# 28th Annual Computational Neuroscience Meeting: CNS*2019

**DOI:** 10.1186/s12868-019-0538-0

**Published:** 2019-11-14

## K1 Brain networks, adolescence and schizophrenia

### Ed Bullmore

#### University of Cambridge, Department of Psychiatry, Cambridge, United Kingdom

##### **Correspondence:** Ed Bullmore (etb23@cam.ac.uk)

*BMC Neuroscience* 2019, **20(Suppl 1)**:K1

The adolescent transition from childhood to young adulthood is an important phase of human brain development and a period of increased risk for incidence of psychotic disorders. I will review some of the recent neuroimaging discoveries concerning adolescent development, focusing on an accelerated longitudinal study of ~ 300 healthy young people (aged 14–25 years) each scanned twice using MRI. Structural MRI, including putative markers of myelination, indicates changes in local anatomy and connectivity of association cortical network hubs during adolescence. Functional MRI indicates strengthening of initially weak connectivity of subcortical nuclei and association cortex. I will also discuss the relationships between intra-cortical myelination, brain networks and anatomical patterns of expression of risk genes for schizophrenia.

## K2 Neural circuits for mental simulation

### Kenji Doya

#### Okinawa Institute of Science and Technology, Neural Computation Unit, Okinawa, Japan

##### **Correspondence:** Kenji Doya (doya@oist.jp)

*BMC Neuroscience* 2019, **20(Suppl 1)**:K2

The basic process of decision making is often explained by learning of values of possible actions by reinforcement learning. In our daily life, however, we rarely rely on pure trial-and-error and utilize any prior knowledge about the world to imagine what situation will happen before taking an action. How such “mental simulation” is implemented by neural circuits and how they are regulated to avoid delusion are exciting new topics of neuroscience. Here I report our works with functional MRI in humans and two-photon imaging in mice to clarify how action-dependent state transition models are learned and utilized in the brain.

## K3 One network, many states: varying the excitability of the cerebral cortex

### Maria V. Sanchez-Vives

#### IDIBAPS and ICREA, Systems Neuroscience, Barcelona, Spain

##### **Correspondence:** Maria V. Sanchez-Vives (msanche3@clinic.cat)

*BMC Neuroscience* 2019, **20(Suppl 1)**:K3

In the transition from deep sleep, anesthesia or coma states to wakefulness, there are profound changes in cortical interactions both in the temporal and the spatial domains. In a state of low excitability, the cortical network, both in vivo and in vitro, expresses it “default activity pattern”, slow oscillations [1], a state of low complexity and high synchronization. Understanding the multiscale mechanisms that enable the emergence of complex brain dynamics associated with wakefulness and cognition while departing from low-complexity, highly synchronized states such as sleep, is key to the development of reliable monitors of brain state transitions and consciousness levels during physiological and pathological states. In this presentation I will discuss different experimental and computational approaches aimed at unraveling how the complexity of activity patterns emerges in the cortical network as it transitions across different brain states. Strategies such as varying anesthesia levels or sleep/awake transitions in vivo, or progressive variations in excitability by variable ionic levels, GABAergic antagonists, potassium blockers or electric fields in vitro, reveal some of the common features of these different cortical states, the gradual or abrupt transitions between them, and the emergence of dynamical richness, providing hints as to the underlying mechanisms.

**Reference**

1. Sanchez-Vives, M, Marcello M, Maurizio M. Shaping the default activity pattern of the cortical network. *Neuron* 94.5 (2017): 993–1001.

## K4 Neural circuits for flexible memory and navigation

### Ila Fiete

#### Massachusetts Institute of Technology, McGovern Institute, Cambridge, United States of America

##### **Correspondence:** Ila Fiete (fiete@mit.edu)

*BMC Neuroscience* 2019, **20(Suppl 1)**:K4

I will discuss the problems of memory and navigation from a computational and functional perspective: What is difficult about these problems, which features of the neural circuit architecture and dynamics enable their solutions, and how the neural solutions are uniquely robust, flexible, and efficient.

## F1 The geometry of abstraction in hippocampus and pre-frontal cortex

### Silvia Bernardi^1^, Marcus K. Benna^2^, Mattia Rigotti^3^, Jérôme Munuera^4^, Stefano Fusi^1^, C. Daniel Salzman^1^

#### ^1^Columbia University, Zuckerman Mind Brain Behavior Institute, New York, United States of America; ^2^Columbia University, Center for Theoretical Neuroscience, Zuckerman Mind Brain Behavior Institute, New York, NY, United States of America; ^3^IBM Research AI, Yorktown Heights, United States of America, ^4^Columbia University, Centre National de la Recherche Scientifique (CNRS), École Normale Supérieure, Paris, France

##### **Correspondence:** Marcus K. Benna (mkb2162@columbia.edu)

*BMC Neuroscience* 2019, **20(Suppl 1)**:F1

Abstraction can be defined as a cognitive process that finds a common feature—an abstract variable, or concept—shared by a number of examples. Knowledge of an abstract variable enables generalization to new examples based upon old ones. Neuronal ensembles could represent abstract variables by discarding all information about specific examples, but this allows for representation of only one variable. Here we show how to construct neural representations that encode multiple abstract variables simultaneously, and we characterize their geometry. Representations conforming to this geometry were observed in dorsolateral pre-frontal cortex, anterior cingulate cortex, and the hippocampus in monkeys performing a serial reversal-learning task. These neural representations allow for generalization, a signature of abstraction, and similar representations are observed in a simulated multi-layer neural network trained with back-propagation. These findings provide a novel framework for characterizing how different brain areas represent abstract variables, which is critical for flexible conceptual generalization and deductive reasoning.

## F2 Signatures of network structure in timescales of spontaneous activity

### Roxana Zeraati^1^, Nicholas Steinmetz^2^, Tirin Moore^3^, Tatiana Engel^4^, Anna Levina^5^

#### ^1^University of Tübingen, International Max Planck Research School for Cognitive and System Neuroscience, Tübingen, Germany; ^2^University of Washington, Department of Biological Structure, Seattle, United States of America; ^3^Stanford University, Department of Neurobiology, Stanford, California, United States of America; ^4^Cold Spring Harbor Laboratory, Cold Spring Harbor, NY, United States of America; ^5^University of Tübingen, Tübingen, Germany

##### **Correspondence:** Roxana Zeraati (roxana.zeraati@tuebingen.mpg.de)

*BMC Neuroscience* 2019, **20(Suppl 1)**:F2

Cortical networks are spontaneously active. Timescales of these intrinsic fluctuations were suggested to reflect the network’s specialization for task-relevant computations. However, how these timescales arise from the spatial network structure is unknown. Spontaneous cortical activity unfolds across different spatial scales. On a local scale of individual columns, ongoing activity spontaneously transitions between episodes of vigorous (On) and faint (Off) spiking, synchronously across cortical layers. On a wider spatial scale, activity propagates as cascades of elevated firing across many columns, characterized by the branching ratio defined as the average number of units activated by each active unit. We asked, to what extent the timescales of spontaneous activity reflect the dynamics on these two spatial scales and the underlying network structure. To this end, we developed a branching network model capable of capturing both the local On-Off dynamics and the global activity propagation. Each unit in the model represents a cortical column, which has spatially structured connections to other columns (Fig. 1A). The columns stochastically transition between On and Off states. Transitions to On-state are driven by stochastic external inputs and by excitatory inputs from the neighboring columns (horizontal recurrent input). An On state can persist due to a self-excitation representing strong recurrent connections within one column (vertical recurrent input). On and Off episode durations in our model follow exponential distributions, similar to the On-Off dynamics observed in single cortical columns (Fig. 1B). We fixed the statistics of On-Off transitions and the global propagation, and studied the dependence of intrinsic timescales on the network spatial structure.

**Fig. 1:**
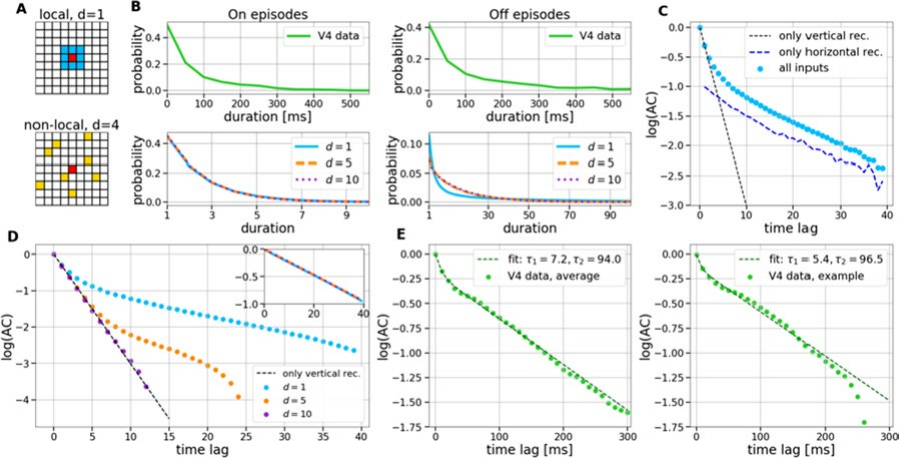
**a** Schematic representation of the model local and non-local connectivity. **b** Distributions of On-Off episode duration in V4 data and model. **c** Representation of different timescales in single columns AC. **d** Average AC of individual columns and the population activity (inset, with the same axes) for different network structures. **e** V4 data AC averaged over all recordings, and an example recording

We found that the timescales of local dynamics reflect the spatial network structure. In the model, activity of single columns exhibits two distinct timescales: one induced by the recurrent excitation within the column and another induced by interactions between the columns (Fig. 1C). The first timescale dominates dynamics in networks with more dispersed connectivity (Fig. 1A, non-local; Fig. 1D), whereas the second timescale is prominent in networks with more local connectivity (Fig. 1A, local; Fig. 1D). Since neighboring columns share many of their recurrent inputs, the second timescale is also evident in cross-correlations (CC) between columns, and it becomes longer with increasing distance between columns.

To test the model predictions, we analyzed 16-channel microelectrode array recordings of spiking activity from single columns in the primate area V4. During spontaneous activity, we observed two distinct timescales in columnar On-Off fluctuations (Fig. 1E). Two timescales were also present in CCs of neural activity on different channels within the same column. To examine how timescales depend on horizontal cortical distance, we leveraged the fact that columnar recordings generally exhibit slight horizontal shifts due to variability in the penetration angle. As a surrogate for horizontal displacements between pairs of channels, we used distances between centers of their receptive fields (RF). As predicted by the model, the second timescale in CCs became longer with increasing RF-center distance. Our results suggest that timescales of local On-Off fluctuations in single cortical columns provide information about the underlying spatial network structure of the cortex.

## F3 Internal bias controls phasic but not delay-period dopamine activity in a parametric working memory task

### Néstor Parga^1^, Stefania Sarno^1^, Manuel Beiran^2^, José Vergara^3^, Román Rossi-Pool^3^, Ranulfo Romo^3^

#### ^1^Universidad Autónoma Madrid, Madrid, Spain; ^2^Ecole Normale Supérieure, Department of Cognitive Studies, Paris, France; ^3^Universidad Nacional Autónoma México, Instituto de Fisiología Celular, México DF, Mexico

##### **Correspondence:** Néstor Parga (nestor.parga@uam.es)

*BMC Neuroscience* 2019, **20(Suppl 1)**:F3

Dopamine (DA) has been implied in coding reward prediction errors (RPEs) and in several other phenomena such as working memory and motivation to work for reward. Under uncertain stimulation conditions DA phasic responses to relevant task cues reflect cortical perceptual decision-making processes, such as the certainty about stimulus detection and evidence accumulation, in a way compatible with the RPE hypothesis [1, 2]. This suggests that the midbrain DA system receives information from cortical circuits about decision formation and transforms it into an RPE signal. However, it is not clear how DA neurons behave when making a decision involves more demanding cognitive features, such as working memory and internal biases, or how they reflect motivation under uncertain conditions. To advance knowledge on these issues we have recorded and analyzed the firing activity of putatively midbrain DA neurons, while monkeys discriminated the frequencies of two vibrotactile stimuli delivered to one fingertip. This two-interval forced choice task, in which both stimuli were selected randomly in each trial, has been widely used to investigate perception, working memory and decision-making in sensory and frontal areas [3]; the current study adds to this scenario possible roles of midbrain DA neurons.

We found that the DA responses to the stimuli were not monotonically tuned to their frequency values. Instead they were controlled by an internally generated bias (contraction bias). This bias induced a subjective difficulty that modulated those responses as well as the accuracy and the response times (RTs). A Bayesian model for the choice explained the bias and gave a measure of the animal’s decision confidence, which also appeared modulated by the bias. We also found that the DA activity was above baseline throughout the delay (working memory) period. Interestingly, this activity was neither tuned to the first frequency nor controlled by the internal bias. While the phasic responses to the task events could be described by a reinforcement learning model based on belief states, the ramping behavior exhibited during the delay period could not be explained by standard models. Finally, the DA responses to the stimuli in short-RT trials and long-RTs trials were significantly different; interpreting the RTs as a measure of motivation, our analysis indicated that motivation affected strongly the responses to the task events but had only a weak influence on the DA activity during the delay interval. To summarize, our results show for the first time that an internal phenomenon (the bias) can control the DA phasic activity similar to the way physical differences in external stimuli do. We also encountered a ramping DA activity during the working memory period, independent of the memorized frequency value. Overall, our study supports the notion that delay and phasic DA activities accomplish quite different functions.

**References**

1. Sarno S, de Lafuente V, Romo R, Parga N. Dopamine reward prediction error signal codes the temporal evaluation of a perceptual decision report. *PNAS*. 201712479 (2017)
2. Lak A, Nomoto K, Keramati M, Sakagami M, Kepecs A. Midbrain dopamine neurons signal belief in choice accuracy during a perceptual decision. *Curr Bio* 27, 821–832 (2017)
3. Romo R, Brody CD, Hernández A, Lemus L. Neuronal correlates of parametric working memory in the prefrontal cortex. *Nature* 399, 470–473 (1999)

## O1 Representations of dissociated shape and category in deep Convolutional Neural Networks and human visual cortex

### Astrid Zeman, J Brendan Ritchie, Stefania Bracci, Hans Op de Beeck

#### KULeuven, Brain and Cognition, Leuven, Belgium

##### **Correspondence:** Astrid Zeman (astrid.zeman@kuleuven.be)

*BMC Neuroscience* 2019, **20(Suppl 1)**:O1

Deep Convolutional Neural Networks (CNNs) excel at object recognition and classification, with accuracy levels that now exceed humans [1]. In addition, CNNs also represent clusters of object similarity, such as the animate-inanimate division that is evident in object-selective areas of human visual cortex [2]. CNNs are trained using natural images, which contain shape and category information that is often highly correlated [3]. Due to this potential confound, it is therefore possible that CNNs rely upon shape information, rather than category, to classify objects. We investigate this possibility by quantifying the representational correlations of shape and category along the layers of multiple CNNs, with human behavioural ratings of these two factors, using two datasets that explicitly orthogonalize shape from category [3, 4] (Fig. 1a, b, c). We analyse shape and category representations along the human ventral pathway areas using fMRI (Fig. 1d) and measure correlations between artificial with biological representations by comparing the output from CNN layers with fMRI activation in ventral areas (Fig. 1e).

**Fig. 1:**
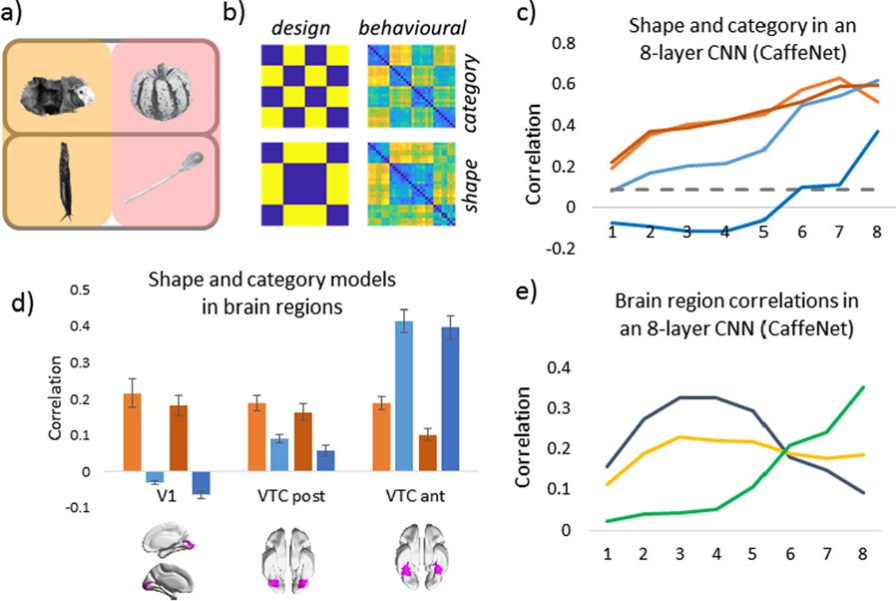
Shape and category models in CNNs vs the brain. **a** Example stimuli **b** Design and behavioral models **c** Shape (orange) and category (blue) correlations in CNNs. Behavioral (darker) and design (lighter) models. Only one CNN shown. **d** Shape (orange) and category (blue) correlations in ventral brain regions. **e** V1 (blue), posterior (yellow) and anterior (green) VTC correlated with CNN layers

First, we find that CNNs encode object category independently from shape, which peaks at the final fully connected layer for all network architectures. At the initial layer of all CNNs, shape is represented significantly above chance in the majority of cases (94%), whereas category is not. Category information only increases above the significance level in the final few layers of all networks, reaching a maximum at the final layer after remaining low for the majority of layers. Second, by using fMRI to analyse shape and category representations along the ventral pathway, we find that shape information decreases from early visual cortex (V1) to the anterior portion of ventral temporal cortex (VTC). Conversely, category information increases from low to high from V1 to anterior VTC. This two-way interaction is significant for both datasets, demonstrating that this effect is evident for both low-level (orientation dependent) and high-level (low vs high aspect ratio) definitions of shape. Third, comparing CNNs with brain areas, the highest correlation with anterior VTC occurs at the final layer of all networks. V1 correlations reach a maximum prior to fully connected layers, at early, mid or late layers, depending upon network depth. In all CNNs, the order of maximum correlations with neural data corresponds well with the flow of visual information along the visual pathway. Overall, our results suggest that CNNs represent category information independently from shape, similarly to human object recognition processing.

**References**

1. He K, Zhang X, Ren S, Sun J. Delving Deep into Rectifiers: Surpassing Human-Level Performance on ImageNet Classification*. 2015 IEEE International Conference on Computer Vision (ICCV), Santiago* 2015, pp 1026–1034.
2. Khaligh-Razavi S-M, Kriegeskorte N. Deep Supervised, but Not Unsupervised, Models May Explain IT Cortical Representation. *PLoS Computational Biology* 2014, 10(11), e1003915.
3. Bracci S, Op de Beeck H. Dissociations and Associations between Shape and Category. *J Neurosci* 2016, 36(2), 432–444.
4. Ritchie JB, Op de Beeck H. Using neural distance to predict reaction time for categorizing the animacy, shape, and abstract properties of objects. *BioRxiv* 2018. Preprint at: 10.1101/496539

## O2 Discovering the building blocks of hearing: a data-driven, neuro-inspired approach

### Lotte Weerts^1^, Claudia Clopath^2^, Dan Goodman^1^

#### ^1^Imperial College London, Electrical and Electronic Engineering, London, United Kingdom; ^2^Imperial College London, Department of Bioengineering, London, United Kingdom

##### **Correspondence:** Dan Goodman (d.goodman@imperial.ac.uk)

*BMC Neuroscience* 2019, **20(Suppl 1)**:O2

Our understanding of hearing and speech recognition rests on controlled experiments requiring simple stimuli. However, these stimuli often lack the variability and complexity characteristic of complex sounds such as speech. We propose an approach that combines neural modelling with data-driven machine learning to determine auditory features that are both theoretically powerful and can be extracted by networks that are compatible with known auditory physiology. Our approach bridges the gap between detailed neuronal models that capture specific auditory responses, and research on the statistics of real-world speech data and its relationship to speech recognition. Importantly, our model can capture a wide variety of well studied features using specific parameter choices, and allows us to unify several concepts from different areas of hearing research.

We introduce a feature detection model with a modest number of parameters that is compatible with auditory physiology. We show that this model is capable of detecting a range of features such as amplitude modulations (AMs) and onsets. In order to objectively determine relevant feature detectors within our model parameter space, we use a simple classifier that approximates the information bottleneck, a principle grounded in information theory that can be used to define which features are “useful”. By analysing the performance in a classification task, our framework allows us to determine the best model parameters and their neurophysiological implications and relate those to psychoacoustic findings.

We analyse the performance of a range of model variants in a phoneme classification task (Fig. 1). Some variants improve accuracy compared to using the original signal, indicating that our feature detection model extracts useful information. By analysing the properties of high performing variants, we rediscover several proposed mechanisms for robust speech processing. Firstly, our result suggest that model variants that can detect and distinguish between formants are important for phoneme recognition. Secondly, we rediscover the importance of AM sensitivity for consonant recognition, which is in line with several experimental studies that show that consonant recognition is degraded when certain amplitude modulations are removed. Besides confirming well-known mechanisms, our analysis hints at other less-established ideas, such as the importance of onset suppression. Our results indicate that onset suppression can improve phoneme recognition, which is in line with the hypothesis that the suppression of onset noise (or “spectral splatter”), as observed in the mammalian auditory brainstem, can improve the clarity of a neural harmonic representation. We also discover model variants that are responsive to more complex features, such as combined onset and AM sensitivity. Finally, we show how our approach lends itself to be extended to more complex environments, by distorting the clean speech signal with noise.

**Fig. 1:**
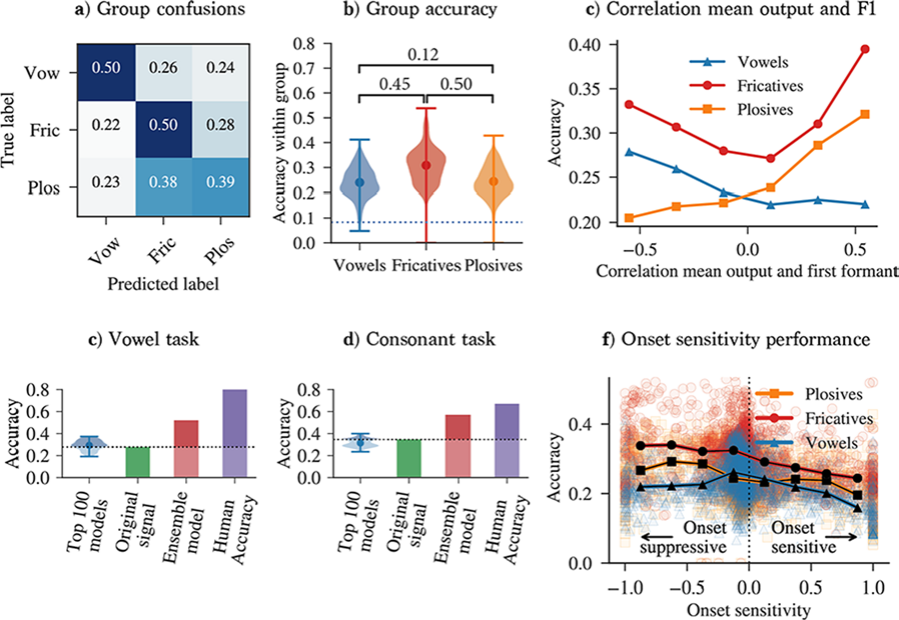
**a** Between-group confusion matrix for best parameters. **b** distribution of within-group accuracies and between-group accuracy correlations. **c** Within-group accuracy and correlation of model output and spectral peaks. **d**, **e** Accuracy achieved with model variants, the original filtered signal, and ensemble models on a vowel (**d**) and consonant (**e**) task. **f** Within-group accuracy versus onset strength

Our approach has various potential applications. Firstly, it could lead to new, testable experimental hypotheses for understanding hearing. Moreover, promising features could be directly applied as a new acoustic front-end for speech recognition systems.

**Acknowledgments:** This work was partly supported by a Titan Xp donated by the NVIDIA Corporation, The Royal Society grant RG170298 and the Engineering and Physical Sciences Research Council (grant number EP/L016737/1).

## O3 Modeling stroke and rehabilitation in mice using large-scale brain networks

### Spase Petkoski^1^, Anna Letizia Allegra Mascaro^2^, Francesco Saverio Pavone^2^, Viktor Jirsa^1^

#### ^1^Aix-Marseille Université, Institut de Neurosciences des Systèmes, Marseille, France; ^2^University of Florence, European Laboratory for Non-linear Spectroscopy, Florence, Italy

##### **Correspondence:** Spase Petkoski (spase.petkoski@univ-amu.fr)

*BMC Neuroscience* 2019, **20(Suppl 1)**:O3

Individualized large-scale computational modeling of the dynamics associated with the brain pathologies [1] is an emerging approach in the clinical applications, which gets validation through animal models. A good candidate for confirmation of brain network causality is stroke and the subsequent recovery, which alter brain’s structural connectivity, and this is then reflected on functional and behavioral level. In this study we use large-scale brain network model (BNM) to computationally validate the structural changes due to stroke and recovery in mice, and their impact on the resting state functional connectivity (FC), as captured by wide-field calcium imaging.

We built our BNM based on the detailed Allen Mouse (AM) connectome that is implemented in The Virtual Mouse Brain [2]. It dictates the strength of the couplings between distant brain regions based on tracer data. The homogeneous local connectivity is absorbed into the neuronal mass model that is generally derived from mean activity of populations of spiking neurons, Fig. 1, and is here represented by the Kuramoto oscillators [3], as canonical model for network synchronization due to weak interactions. The photothrombotic focal stroke affects the right primary motor cortex (rM1). The injured forelimb is daily trained on a custom designed robotic device (M-Platform, [4, 5]) from 5 days after the stroke for a total of 4 weeks. The stroke is modeled by different levels of damage of the links connecting rM1, while the recovery is represented by reinforcing of alternative connections of the nodes initially linked to it [6]. We systematically simulate various impacts of stroke and recovery, to find the best match with the coactivation patterns in the data, where the FC is characterized with the phase coherence calculated for the phases of Hilbert transformed delta frequency activity of pixels within separate regions [6]. Statistically significant changes within the FC of 5 animals are obtained for transitions between the three conditions: healthy, stroke and rehabilitation after 4 weeks of training, and these are compared with the best fits for each condition of the model in the parameter’s space of the global coupling strength and stroke impact and rewiring.

**Fig. 1:**
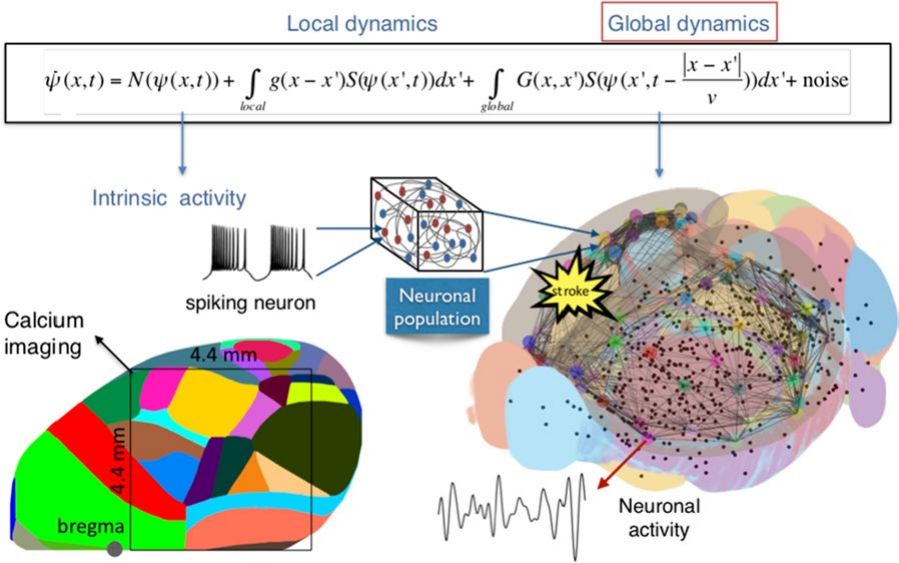
The equation of the mouse BNM shows that the spatiotemporal dynamics is shaped by the connectivity. The brain network (right) is reconstructed from the AMA, showing the centers of sub cortical (small black dots) and cortical (colored circles) regions. On the left, the field of view during the recordings is overlayed on the reconstructed brain, and different colors represent the cortical regions

This approach uncovers recovery paths in the parameter space of the dynamical system that can be related to neurophysiological quantities such as the white matter tracts. This can lead to better strategies for rehabilitation, such as stimulation or inhibition of certain regions and links that have a critical role on the dynamics of the recovery.

**References**

1. Olmi S, Petkoski S, Guye M, Bartolomei F, Jirsa V. Controlling seizure propagation in large-scale brain networks. *PLoS Comp Biol.* [in press]
2. Melozzi F, Woodman MM, Jirsa VK, Bernard C. The Virtual Mouse Brain: A computational neuroinformatics platform to study whole mouse brain dynamics. *eNeuro* 0111-17. 2017.
3. Petkoski S, Palva JM, Jirsa VK. Phase-lags in large scale brain synchronization: Methodological considerations and in-silico analysis. *PLoS Comp Biol*, 14(7), 1–30. 2018.
4. Spalletti C, et al. A robotic system for quantitative assessment and poststroke training of forelimb retraction in mice. *Neurorehabilitation and neural repair* 28, 188–196. 2014.
5. Allegra Mascaro, A et al. Rehabilitation promotes the recovery of distinct functional and structural features of healthy neuronal networks after stroke. *[under review].*
6. Petkoski S, et al. Large-scale brain network model for stroke and rehabilitation in mice. *[in prep].*

## O4 Self-consistent correlations of randomly coupled rotators in the asynchronous state

### Alexander van Meegen^1^, Benjamin Lindner^2^

#### ^1^Jülich Research Centre, Institute of Neuroscience and Medicine (INM-6) and Institute for Advanced Simulation (IAS-6), Jülich, Germany; ^2^Humboldt University Berlin, Physics Department, Berlin, Germany

##### **Correspondence:** Alexander van Meegen (a.van.meegen@fz-juelich.de)

*BMC Neuroscience* 2019, **20(Suppl 1)**:O4

Spiking activity of cortical neurons in behaving animals is highly irregular and asynchronous. The quasi stochastic activity (the network noise) does not seem to root in the comparatively weak intrinsic noise sources but is most likely due to the nonlinear chaotic interactions in the network. Consequently, simple models of spiking neurons display similar states, the theoretical description of which has turned out to be notoriously difficult. In particular, calculating the neuron’s correlation function is still an open problem. One classical approach pioneered in the seminal work of Sompolinsky et al. [1] used analytically tractable rate units to obtain a self-consistent theory of the network fluctuations and the correlation function of the single unit in the asynchronous irregular state. Recently, the original model attracted renewed interest, leading to substantial extensions and a wide range of novel results [2–5].

Here, we develop a theory for a heterogeneous random network of unidirectionally coupled phase oscillators [6]. Similar to Sompolinsky’s rate-unit model, the system can attain an asynchronous state with pronounced temporal autocorrelations of the units. The model can be examined analytically and even allows for closed-form solutions in simple cases. Furthermore, with a small extension, it can mimic mean-driven networks of spiking neurons and the theory can be extended to this case accordingly.

Specifically, we derived a differential equation for the self-consistent autocorrelation function of the network noise and of the single oscillators. Its numerical solution has been confirmed by simulations of sparsely connected networks (Fig. 1). Explicit expressions for correlation functions and power spectra for the case of a homogeneous network (identical oscillators) can be obtained in the limits of weak or strong coupling strength. To apply the model to networks of sparsely coupled excitatory and inhibitory exponential integrate-and-fire (IF) neurons, we extended the coupling function and derived a second differential equation for the self-consistent autocorrelations. Deep in the mean-driven regime of the spiking network, our theory is in excellent agreement with simulations results of the sparse network.

**Fig. 1:**
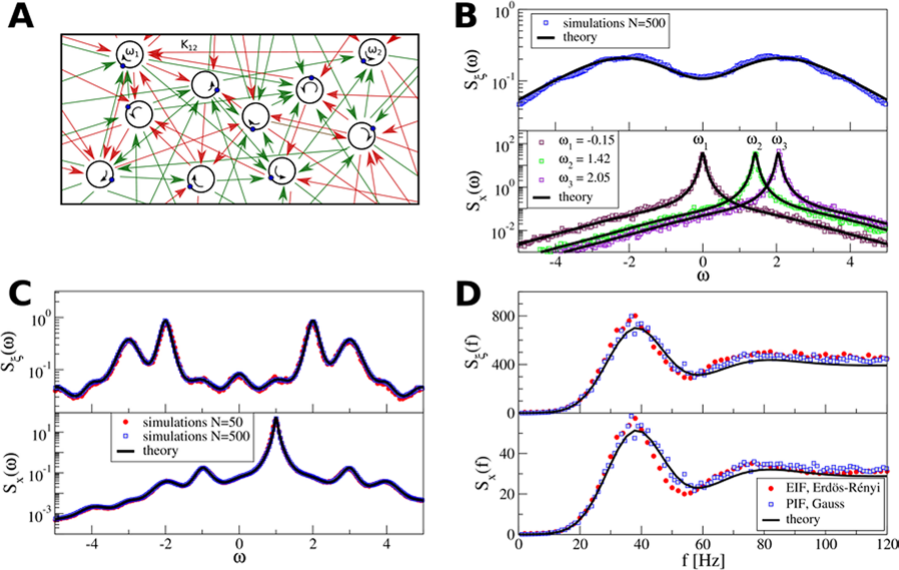
Sketch of a random network of phase oscillators. **a** Self-consistent power spectra of network noise and single units (**b**–**d**), upper and lower plots respectively) obtained from simulations (colored symbols) compared with the theory (black lines): Heterogeneous **b** and homogeneous **c** networks of phase oscillators, and sparsely coupled IF networks (**d**). Panels **b**–**d** adapted and modified from [6]

This work paves the way for more detailed studies of how the statistics of connection strength, the heterogeneity of network parameters, and the form of the interaction function shape the network noise and the autocorrelations of the single element in asynchronous irregular state.

**References**

1. Sompolinsky H, Crisanti A, Sommers HJ. Chaos in random neural networks. *Physical review letters* 1988 Jul 18;61(3):259.
2. Kadmon J, Sompolinsky H. Transition to chaos in random neuronal networks. *Physical Review X* 2015 Nov 19;5(4):041030.
3. Mastrogiuseppe F, Ostojic S. Linking connectivity, dynamics, and computations in low-rank recurrent neural networks. *Neuron* 2018 Aug 8;99(3):609-23.
4. Schuecker J, Goedeke S, Helias M. Optimal sequence memory in driven random networks. *Physical Review X* 2018 Nov 14;8(4):041029.
5. Muscinelli SP, Gerstner W, Schwalger T. Single neuron properties shape chaotic dynamics in random neural networks. *arXiv preprint* arXiv:1812.06925 2018 Dec 17.
6. van Meegen A, Lindner B. Self-Consistent Correlations of Randomly Coupled Rotators in the Asynchronous State. *Physical review letters* 2018 Dec 20;121(25):258302.

## O5 Firing rate-dependent phase responses dynamically regulate Purkinje cell network oscillations

### Yunliang Zang, Erik De Schutter

#### Okinawa Institute of Science and Technology, Computational Neuroscience Unit, Onna-Son, Japan

##### **Correspondence:** Yunliang Zang (zangyl1983@gmail.com)

*BMC Neuroscience* 2019, **20(Suppl 1)**:O5

Phase response curves (PRCs) have been defined to quantify how a weak stimulus shift the next spike timing in regular firing neurons. However, the biophysical mechanisms that shape the PRC profiles are poorly understood. The PRCs in Purkinje cells (PCs) show firing rate (FR) adaptation. At low FRs, the responses are small and phase independent. At high FRs, the responses become phase dependent at later phases, with their onset phases gradually left-shifted and peaks gradually increased, due to an unknown mechanism [1, 2].

Using our recently developed compartment-based PC model [3], we reproduced the FR-dependence of PRCs and identified the depolarized interspike membrane potential as the mechanism underlying the transition from phase-independent responses at low FRs to the gradually left-shifted phase-dependent responses at high FRs. We also demonstrated this mechanism plays a general role in shaping PRC profiles in other neurons.

PC axon collaterals have been proposed to correlate temporal spiking in PC ensembles [4, 5], but whether and how they interact with the FR-dependent PRCs to regulate PC output remains unexplored. We built a recurrent inhibitory PC-to-PC network model to examine how FR-dependent PRCs regulate the synchrony of high frequency (~ 160 Hz) oscillations observed in vivo [4]. We find the synchrony of these oscillations increases with FR due to larger and broader PRCs at high FRs. This increased synchrony still holds when the network incorporates dynamically and heterogeneously changing cellular FRs. Our work implies that FR-dependent PRCs may be a critical property of the cerebellar cortex in combining rate- and synchrony-coding to dynamically organize its temporal output.

**References**

1. Phoka E., et al., A new approach for determining phase response curves reveals that Purkinje cells can act as perfect integrators. *PLoS Comput. Biol* 2010. 6(4): p. e1000768.
2. Couto J., et al., On the firing rate dependency of the phase response curve of rat Purkinje neurons in vitro. PLoS *Comput. Biol* 2015. 11(3): p. e1004112.
3. Zang Y, Dieudonne S, De Schutter E. Voltage- and Branch-Specific Climbing Fiber Responses in Purkinje Cells. *Cell Rep* 2018. 24(6): p. 1536-1549.
4. de Solages C., et al., High-frequency organization and synchrony of activity in the purkinje cell layer of the cerebellum. *Neuron* 2008. 58(5): p. 775-88.
5. Witter L., et al., Purkinje Cell Collaterals Enable Output Signals from the Cerebellar Cortex to Feed Back to Purkinje Cells and Interneurons. *Neuron* 2016. 91(2): p. 312-9.

## O6 Computational modeling of brainstem-spinal circuits controlling locomotor speed and gait

### Ilya Rybak, Jessica Ausborn, Simon Danner, Natalia Shevtsova

#### Drexel University College of Medicine, Department of Neurobiology and Anatomy, Philadelphia, PA, United States of America

##### **Correspondence:** Ilya Rybak (rybak@drexel.edu)

*BMC Neuroscience* 2019, **20(Suppl 1)**:O6

Locomotion is an essential motor activity allowing animals to survive in complex environments. Depending on the environmental context and current needs quadruped animals can switch locomotor behavior from slow left-right alternating gaits, such as walk and trot (typical for exploration), to higher-speed synchronous gaits, such as gallop and bound (specific for escape behavior). At the spinal cord level,the locomotor gait is controlled by interactions between four central rhythm generators (RGs) located on the left and right sides of the lumbar and cervical enlargements of the cord, each producing rhythmic activity controlling one limb. The activities of the RGs are coordinated by commissural interneurons (CINs), projecting across the midline to the contralateral side of the cord, and long propriospinal neurons (LPNs), connecting the cervical and lumbar circuits. At the brainstem level, locomotor behavior and gaitsare controlled by two majorbrainstem nuclei: the cuneiform (CnF) and the pedunculopontine (PPN) nuclei [1]. Glutamatergic neurons in both nuclei contribute to the control of slow alternating-gait movements, whereas only activation of CnF can elicit high-speed synchronous-gait locomotion. Neurons from both regions project to the spinal cord via descendingreticulospinal tracts from thelateral paragigantocellular nuclei (LPGi) [2].

To investigate the brainstem control of spinal circuits involved in the slow exploratory and fast escape locomotion, we built a computational model ofthe brainstem-spinal circuits controlling these locomotor behaviors. The spinal cord circuits in the modelincluded four RGs (one per limb) interacting via cervical and lumbar CINs and LPNs. The brainstem model incorporated bilaterally interacting CnF and PPN circuits projecting to the LPGi nuclei that mediated the descending pathways to the spinal cord.These pathways provided excitation of all RGs to control locomotor frequency and inhibited selected CINs and LPNs, which allowed the model to reproduce the speed-dependent gait transitions observed in intact mice and the loss of particular gaits in mutants lacking some genetically identified CINs [3].The proposed structure of synaptic inputs of the descending (LPGi) pathways to the spinal CINs and LPNs allowed the model to reproduce the experimentally observed effects of stimulation of excitatory and inhibitory neurons within CnF, PPN, and LPGi. The suggests explanations for (a) the speed-dependent expression of different locomotor gaits and the role of different CINs and LPNs in gait transitions, (b) the involvement of the CnF and PPN nuclei in the control of low-speed alternating-gait locomotion and the specific role of the CnF in the control of high-speed synchronous-gait locomotion, and (c) the role of inhibitory neurons in these areas in slowing down and stopping locomotion. The model provides important insights into the brainstem-spinal cord interactions and the brainstem control of locomotor speed and gaits.

**References**

1. Caggiano V, Leiras R, Goñi-Erro H, et al. Midbrain circuits that set locomotor speed and gait selection. *Nature* 2018, 553, 455–460.
2. Capelli P, Pivetta C, Esposito MS, Arber S. Locomotor speed control circuits in the caudal brainstem. *Nature* 2017, 551, 373–377.
3. Bellardita C, Kiehn O. Phenotypic characterization of speed-associated gait changes in mice reveals modular organization of locomotor networks. *Curr Biol* 2015, 25, 1426–1436.

## O7 Co-refinement of network interactions and neural response properties in visual cortex

### Sigrid Trägenap^1^, Bettina Hein^1^, David Whitney^2^, Gordon Smith^3^, David Fitzpatrick^2^, Matthias Kaschube^1^

#### ^1^Frankfurt Institute for Advanced Studies (FIAS), Department of Neuroscience, Frankfurt, Germany; ^2^Max Planck Florida Institute, Department of Neuroscience, Jupiter, FL, United States of America; ^3^University of Minnesota, Department of Neuroscience, Minneapolis, MN, United States of America

##### **Correspondence:** Sigrid Trägenap (traegenap@fias.uni-frankfurt.de)

*BMC Neuroscience* 2019, **20(Suppl 1)**:O7

In the mature visual cortex, local tuning properties are linked through distributed network interactions with a remarkable degree of specificity [1]. However, it remains unknown whether the tight linkage between functional tuning and network structure is an intrinsic feature of cortical circuits, or instead gradually emerges in development. Combining virally-mediated expression of GCAMP6s in pyramidal neurons with wide-field epifluorescence imaging in ferret visual cortex, we longitudinally monitored the spontaneous activity correlation structure—our proxy for intrinsic network interactions- and the emergence of orientation tuning around eye-opening.

We find that prior to eye-opening, the layout of emerging iso-orientation domains is only weakly similar to the spontaneous correlation structure. Nonetheless within one week of visual experience, the layout of iso-orientation domains and the spontaneous correlation structure become rapidly matched. Motivated by these observations, we developed dynamical equations to describe how tuning and network correlations co-refine to become matched with age. Here we propose an objective function capturing the degree of consistency between orientation tuning and network correlations. Then by gradient descent of this objective function, we derive dynamical equations that predict an interdependent refinement of orientation tuning and network correlations. To first approximation, these equations predict that correlated neurons become more similar in orientation tuning over time, while network correlations follow a relaxation process increasing the degree of self-consistency in their link to tuning properties.

Empirically, we indeed observe a refinement with age in both orientation tuning and spontaneous correlations. Furthermore, we find that this framework can utilize early measurements of orientation tuning and correlation structure to predict aspects of the future refinement in orientation tuning and spontaneous correlations. We conclude that visual response properties and network interactions show a considerable degree of coordinated and interdependent refinement towards a self-consistent configuration in the developing visual cortex.

**Reference**

1. Smith GB, Hein B, Whitney DE, Fitzpatrick D, Kaschube M. Distributed network interactions and their emergence in developing neocortex. *Nature Neuroscience* 2018 Nov;21(11):1600.

## O8 Receptive field structure of border ownership-selective cells in response to direction of figure

### Ko Sakai^1^, Kazunao Tanaka^1^, Rüdiger von der Heydt^2^, Ernst Niebur^3^

#### ^1^University of Tsukuba, Department of Computer Science, Tsukuba, Japan; ^2^Johns Hopkins University, Krieger Mind/Brain Institute, Baltimore, United States of America; ^3^Johns Hopkins, Neuroscience, Baltimore, MD, United States of America

##### **Correspondence:** Ko Sakai (sakai@cs.tsukuba.ac.jp)

*BMC Neuroscience* 2019, **20(Suppl 1)**:O8

The responses of border ownership-selective cells (BOCs) have been reported to signal the direction of figure (DOF) along the contours in natural images with a variety of shapes and textures [1]. We examined the spatial structure of the optimal stimuli for BOCs in monkey visual cortical area V2 to determine the structure of the receptive field. We computed the spike triggered average (STA) from responses of the BOCs to natural images (JHU archive, http://dx.doi.org/10.7281/T1C8276W). To estimate the STA in response to figure-ground organization of natural images, we tagged figure regions with luminance contrast. The left panel in Fig 1 illustrates the procedure for STA computation. We first aligned all images to a given cell’s preferred orientation and preferred direction of figure. We then grouped the images based on the luminance contrast of their figure regions with respect to their ground regions, and averaged them separately for each group. By averaging the bright-figure stimuli with weights based on each cell’s spike count, we were able to observe the optimal figure and ground sub-regions as brighter and darker regions, respectively. By averaging the dark-figure stimuli, we obtained the reverse. We then generated the STA by subtracting the average of the dark-figure stimuli from that of the bright-figure stimuli. This subtraction canceled out the dependence of response to contrast. We compensated for the bias due to the non-uniformity of luminance in the natural images by subtracting the simple ensemble average of the stimuli (equivalent to weight = 1 for all stimuli) from the weighted average. The mean STA across 22 BOCs showed facilitated and suppressed sub-regions in response to the figure towards the preferred and non-preferred DOFs, respectively (Fig. 1, the right panel). The structure was shown more clearly when figure and ground were replaced by a binary mask. The result demonstrates, for the first time, the antagonistic spatial structure in the receptive field of BOCs in response to figure-ground organization.

**Fig. 1:**
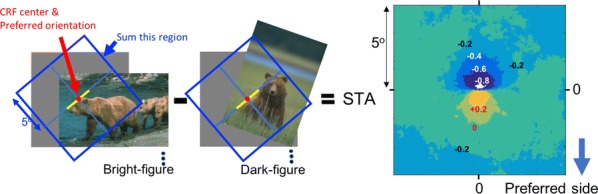
(Left) We tagged figure regions with luminance contrast to compute the STA in response to figure-ground organization. Natural images with bright foreground were weighted by the cell’s spike counts and summed. The analogue was computed for scenes with dark foregrounds and the difference taken. (Right) The computed STA across 22 cells revealed antagonistic sub-regions

**Acknowledgment:** This work was partly supported by JSPS (KAKENHI, 26280047, 17H01754) and National Institutes of Health (R01EY027544 and R01DA040990).

**Reference**

1. Williford JR, Von Der Heydt R. Figure-ground organization in visual cortex for natural scenes. *eNeuro* 2016 Nov; 3(6) 1–15

## O9 Development of periodic and salt-and-pepper orientation maps from a common retinal origin

### Min Song, Jaeson Jang, Se-Bum Paik

#### Korea Advanced Institute of Science and Technology, Department of Biology and Brain Engineering, Daejeon, South Korea

##### **Correspondence:** Min Song (night@kaist.ac.kr)

*BMC Neuroscience* 2019, **20(Suppl 1)**:O9

Spatial organization of orientation tuning in the primary visual cortex (V1) is arranged in different forms across mammalian species. In some species (e.g. monkeys or cats), the preferred orientation continuously changes across the cortical surface (columnar orientation map), while other species (e.g. mice or rats) have a random-like distribution of orientation preference, termed salt-and-pepper organization. However, it still remains unclear why the organization of the cortical circuit develops differently across species. Previously, it was suggested that each type of circuit might be a result of wiring optimization under different conditions of evolution [1], but the developmental mechanism of each organization of orientation tuning still remains unclear. In this study, we propose that the structural variations between cortical circuits across species simply arise from the differences in physical constraints of the visual system—the size of the retina and V1 (see Fig. 1). By expanding the statistical wiring model proposing that the orientation tuning of a V1 neuron is restricted by the local arrangement of ON and OFF retinal ganglion cells (RGCs) [2, 3], we suggest that the number of V1 neurons sampling a given RGC (sampling ratio) is a crucial factor in determining the continuity of orientation tuning in V1. Our simulation results show that as the sampling ratio increases, neighboring V1 neurons receive similar retinal inputs, resulting in continuous changes in orientation tuning. To validate our prediction, we estimated the sampling ratio of each species from the physical size of the retina and V1 [5] and compared with the organization of orientation tuning. As predicted, this ratio could successfully distinguish diverse mammalian species into two groups according to the organization of orientation tuning, even though the organization has not been clearly predicted by considering only a single factor in the visual system (e.g. V1 size or visual acuity; [4]). Our results suggest a common retinal origin of orientation preference across diverse mammalian species, while its spatial organization can vary depending on the physical constraints of the visual system.

**Fig. 1:**
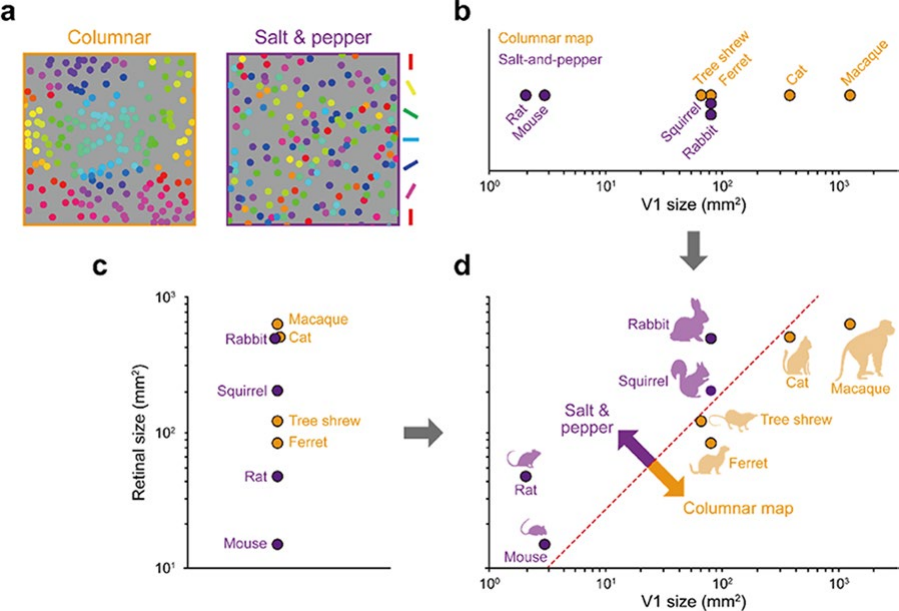
Organization of orientation tuning in a species could be predicted by V1/retinal size

**References**

1. Kaschube M. Neural maps versus salt-and-pepper organization in visual cortex. *Current opinion in neurobiology* 2014, 24: 95-102.
2. Ringach DL. “Haphazard wiring of simple receptive fields and orientation columns in visual cortex.” *Journal of neurophysiology* 2004, 92.1: 468-476.
3. Ringach DL. On the origin of the functional architecture of the cortex. *PloS one* 2007, 2.2: e251.
4. Van Hooser SD, et al. Orientation selectivity without orientation maps in visual cortex of a highly visual mammal. *Journal of Neuroscience* 2005, 25.1: 19-28.
5. Colonnese MT, et al. A conserved switch in sensory processing prepares developing neocortex for vision. *Neuron* 2010, 67.3: 480-498.

## O10 Explaining the pitch of FM-sweeps with a predictive hierarchical model

### Alejandro Tabas^1^, Katharina von Kriegstein^2^

#### ^1^Max Planck Institute for Human Cognitive and Brain Sciences, Research Group in Neural Mechanisms of Human Communication, Leipzig, Germany; ^2^Tesnische Universität Dresden, Chair of Clinical and Cognitive Neuroscience, Faculty of Psychology, Dresden, Germany

##### **Correspondence:** Alejandro Tabas (alextabas@gmail.com)

*BMC Neuroscience* 2019, **20(Suppl 1)**:O10

Frequency modulation (FM) is a basic constituent of vocalisation. FM-sweeps in the frequency range and modulation rates of speech have been shown to elicit a pitch percept that consistently deviates from the sweep average frequency [1]. Here, we use this perceptual effect to inform a model characterising the neural encoding of FM.

First, we performed a perceptual experiment where participants were asked to match the pitch of 30 sweeps with probe sinusoids of the same duration. The elicited pitch systematically deviated from the average frequency of the sweep by an amount that depended linearly on the modulation slope. Previous studies [2] have proposed that the deviance might be due to a fixed-sized-window integration process that fosters frequencies present at the end of the stimulus. To test this hypothesis, we conducted a second perceptual experiment considering the pitch elicited by continuous trains of five concatenated sweeps. As before, participants were asked to match the pitch of the sweep trains with probe sinusoids. Our results showed that the pitch deviance from the mean observed in sweeps was severely reduced in the train stimuli, in direct contradiction with the fixed-sized-integration-window hypothesis.

The perceptual effects may also stem from unexpected interactions between the frequencies spanned in the stimuli during pitch processing. We studied this posibility in two well-established families of mechanistic models of pitch. First, we considered a general spectral model that computes pitch as the expected value of the activity distribution across the cochlear decomposition. Due to adaptation effects, this model fostered the spectral range present at the beginning of the sweep: the exact opposite of what we observed in the experimental data. Second, we considered the predictions of the summary autocorrelation function (SACF) [3], a prototypical model of temporal pitch processing that considers the temporal structure of the auditory nerve activity. The SACF was unable to integrate temporal pitch information quickly enough to keep track of the modulation rate, yielding inconsistent pitch predictions that deviated stochastically from the average frequency.

Here, we introduce an alternative hypothesis based on top-down facilitation. Top-down efferents constitute an important fraction of the fibres in the auditory nerve; moreover, top-down predictive facilitation may reduce the metabolic cost and increase the speed of the neural encoding of expected inputs. Our model incorporates a second layer of neurons encoding FM direction that, after detecting that the incoming inputs are consistent with a rising (falling) sweep, anticipate that neurons encoding immediately higher (lower) frequencies will activate next. This prediction is propagated downwards to neurons encoding such frequencies, increasing their readiness and effectively inflating their weight during pitch temporal integration.

The described mechanism fully reproduces our and previously published experimental results (Fig. 1). We conclude that top-down predictive modulation plays an important role in the neural encoding of frequency modulation even at early stages of the processing hierarchy.

**Fig. 1:**
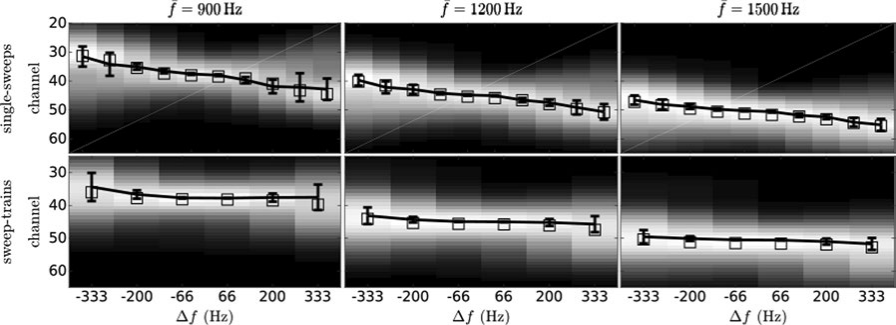
Heatmaps show the distribution of the activation across channels (y-axis) for different sweep frequency gaps (x-axis). Squares printed over the distributions mark the expected value with respect to the distribution. Solid error bars are estimations of the experimental results in the channel space

**References**

1. d’Alessandro C, Castellengo M. The pitch of short‐duration vibrato tones. *The Journal of the Acoustical Society of America* 1994 Mar;95(3):1617-30.
2. Brady PT, House AS, Stevens KN. Perception of sounds characterized by a rapidly changing resonant frequency. *The Journal of the Acoustical Society of America* 1961 Oct;33(10):1357-62.
3. Meddis R, O’Mard LP. Virtual pitch in a computational physiological model. *The Journal of the Acoustical Society of America* 2006 Dec;120(6):3861-9.

## O11 Effects of anesthesia on coordinated neuronal activity and information processing in rat primary visual cortex

### Heonsoo Lee, Shiyong Wang, Anthony Hudetz

#### University of Michigan, Anesthesiology, Ann Arbor, MI, United States of America

##### **Correspondence:** Heonsoo Lee (heonslee@umich.edu)

*BMC Neuroscience* 2019, **20(Suppl 1)**:O11

**Introduction:** Understanding of how anesthesia affects neural activity is important to reveal the mechanism of loss and recovery of consciousness. Despite numerous studies during the past decade, how anesthesia alters spiking activity of different types of neurons and information processing within an intact neural network is not fully understood. Based on prior in vitro studies we hypothesized that excitatory and inhibitory neurons in neocortex are differentially affected by anesthetic. We also predicted that individual neurons are constrained to population activity, leading to impaired information processing within a neural network.

**Methods:** We implanted sixty-four-contact microelectrode arrays in primary visual cortex (layer 5/6, contacts spanning 800 µm depth and 1600 µm width) for recording of extracellular unit activity at three steady-state levels of anesthesia (6, 4 and 2% desflurane) and wakefulness (number of rats = 8). Single unit activities were extracted and putative excitatory and inhibitory neurons were identified based on their spike waveforms and autocorrelogram characteristics (number of neurons = 210). Neuronal features such as firing rate, interspike interval (ISI), bimodality, and monosynaptic spike transmission probabilities were investigated. Normalized mutual information and transfer entropy were also applied to investigate the interaction between spike trains and population activity (local field potential; LFP).

**Results:** First, anesthesia significantly altered characteristics of individual neurons. Firing rate of most neurons was reduced; this effect was more pronounced in inhibitory neurons. Excitatory neurons showed enhanced bursting activity (ISI<9 ms) and silent periods (hundreds of milliseconds) (Fig. 1A). Second, anesthesia disrupted information processing within a neural network. Neurons shared the silent periods, resulting in synchronous population activity (neural oscillations), despite of the suppressed monosynaptic connectivity (Fig. 1B). The population activity (LFP) showed reduced information content (entropy), and was easily predicted by individual neurons; that is, shared information between individual neurons and population activity was significantly increased (Fig. 1C). Transfer entropy analysis revealed a strong directional influence from LFP to individual neurons, suggesting that neuronal activity is constrained to the synchronous population activity.

**Fig. 1:**
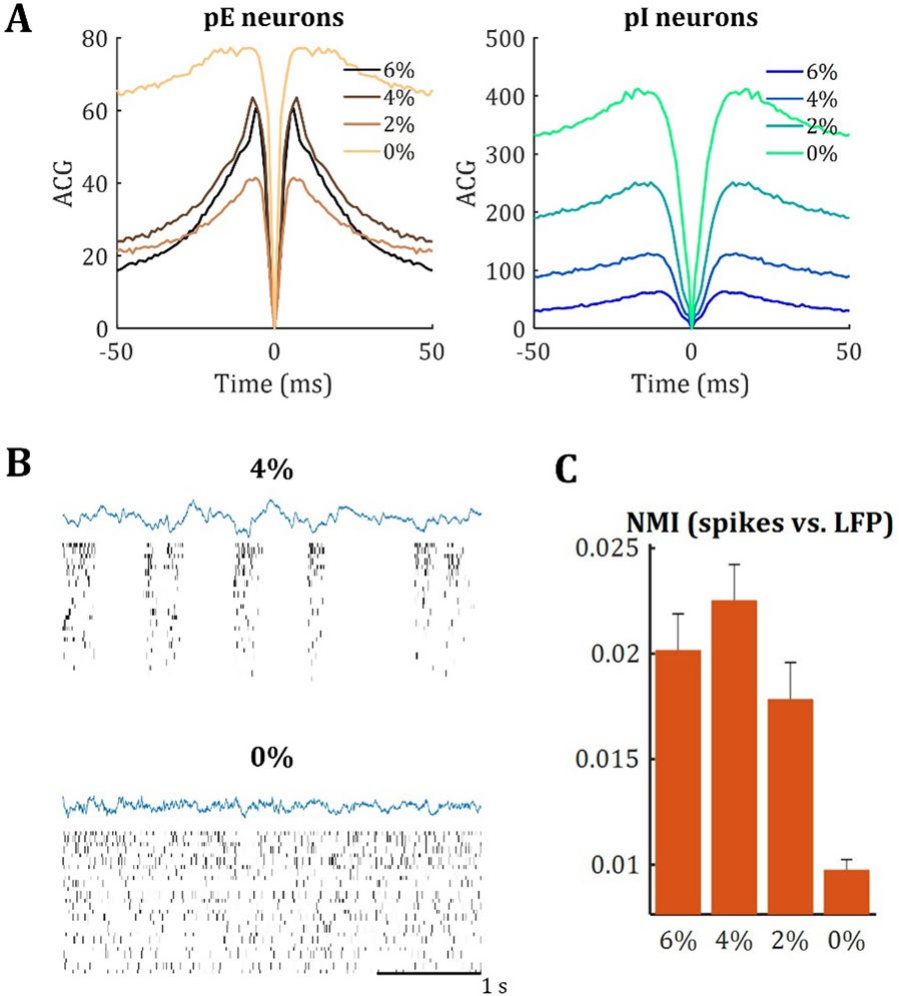
**a** Auto-correlograms (ACG) of putative excitatory (pE) and putative inhibitory (pI) units. **b** Examples of LFP and spiking activity. **c** Normalized mutual information (NMI) between individual spiking activity and LFP

**Conclusions:** This study reveals how excitatory and inhibitory neurons are differentially affected by anesthetic, leading to synchronous population activity and impaired information processing. These findings provide an integrated understanding of anesthetic effects on neuronal activity and information processing. Further study of stimulus evoked activity and computational modeling will provide a more detailed mechanism of how anesthesia alters neural activity and disrupts information processing.

## O12 Learning where to look: a foveated visuomotor control model

### Emmanuel Daucé^1^, Pierre Albigès^2^, Laurent Perrinet^3^

#### ^1^Aix-Marseille Univ, INS, Marseille, France; ^2^Aix-Marseille Univ, Neuroschool, Marseille, France; ^3^CNRS - Aix-Marseille Université, Institut de Neurosciences de la Timone, Marseille, France

##### **Correspondence:** Emmanuel Daucé (emmanuel.dauce@centrale-marseille.fr)

*BMC Neuroscience* 2019, **20(Suppl 1)**:O12

We emulate a model of active vision which aims at finding a visual target whose position and identity are unknown. This generic visual search problem is of broad interest to machine learning, computer vision and robotics, but also to neuroscience, as it speaks to the mechanisms underlying foveation and more generally to low-level attention mechanisms. From a computer vision perspective, the problem is generally addressed by processing the different hypothesis (categories) at all possible spatial configuration through dedicated parallel hardware. The human visual system, however, seems to employ a different strategy, through a combination of a foveated sensor with the capacity of rapidly moving the center of fixation using saccades. Visual processing is done through fast and specialized pathways, one of which mainly conveying information about target position and speed in the peripheral space (the “where” pathway), the other mainly conveying information about the identity of the target (the “what” pathway). The combination of the two pathways is expected to provide most of the useful knowledge about the external visual scene. Still, it is unknown why such a separation exists. Active vision methods provide the ground principles of saccadic exploration, assuming the existence of a generative model from which both the target position and identity can be inferred through active sampling. Taking for granted that (i) the position and category of objects are independent and (ii) the visual sensor is foveated, we consider how to minimize the overall computational cost of finding a target. This justifies the design of two complementary processing pathways: first a classical image classifier, assuming that the gaze is on the object, and second a peripheral processing pathway learning to identify the position of a target in retinotopic coordinates. This framework was tested on a simple task of finding digits in a large, cluttered image (see Fig. 1). Results demonstrate the benefit of specifically learning where to look, and this before actually identifying the target category (with cluttered noise ensuring the category is not readable in the periphery). In the “what” pathway, the accuracy drops to the baseline at mere 5 pixels away from the center of fixation, while issuing a saccade is beneficial in up to 26 pixels around, allowing a much wider covering of the image. The difference between the two distributions forms an “accuracy gain”, that quantifies the benefit of issuing a saccade with respect to a central prior. Until the central classifier is confident, the system should thus perform a saccade to the most likely target position. The different accuracy predictions, such as the ones done in the “what” and the “where” pathway, may also explain more elaborate decision making, such as the inhibition of return. The approach is also energy-efficient as it includes the strong compression rate performed by retina and V1 encoding, which is preserved up to the action selection level. The computational cost of this active inference strategy may thus be way less than that of a brute force framework. This provides evidence of the importance of identifying “putative interesting targets” first and we highlight some possible extensions of our model both in computer vision and modeling.

**Fig. 1:**
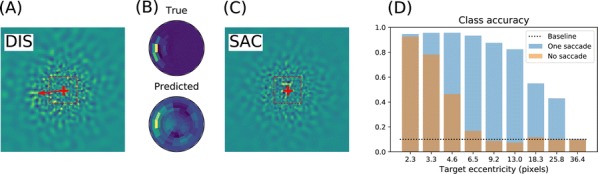
Simulated active vision agent: **a** Example retinotopic input. **b** Example network output (’Predicted’) compared with ground truth (’True’). **c** Accuracy estimation after saccade decision. **d** Orange bars: accuracy of a central classifier w.r.t target eccentricity; Blue bars: classification rate after one saccade (1000 trials average per eccentricity scale)

## O13 A standardized formalism for voltage-gated ion channel models

### Chaitanya Chintaluri^1^, Bill Podlaski^2^, Pedro Goncalves^3^, Jan-Matthis Lueckmann^3^, Jakob H. Macke^3^, Tim P. Vogels^1^

#### ^1^University of Oxford, Centre for Neural Circuits and Behaviour, Oxford, United Kingdom; ^2^Champalimaud Center for the Unknown, Lisbon, Portugal; ^3^Research Center Caesar; Technical University of Munich, Bonn, Germany

##### **Correspondence:** Bill Podlaski (william.podlaski@gmail.com)

*BMC Neuroscience* 2019, **20(Suppl 1)**:O13

Biophysical neuron modelling has become widespread in neuroscience research, with the combination of diverse ion channel kinetics and morphologies being used to explain various single-neuron properties. However, there is no standard by which ion channel models are constructed, making it very difficult to relate models to each other and to experimental data. The complexity and scale of these models also makes them especially susceptible to problems with reproducibility and reusability, especially when translating between different simulators. To address these issues, we revive the idea of a standardised model for ion channels based on a thermodynamic interpretation of the Hodgkin-Huxley formalism, and apply it to a recently curated database of approximately 2500 published ion channel models (ICGenealogy). We show that a standard formulation fits the steady-state and time-constant curves of nearly all voltage-gated models found in the database, and reproduces responses to voltage-clamp protocols with high fidelity, thus serving as a functional translation of the original models. We further test the correspondence of the standardised models in a realistic physiological setting by simulating the complex spiking behaviour of multi-compartmental single-neuron models in which one or several of the ion channel models are replaced by the corresponding best-fit standardised model. These simulations result in qualitatively similar behaviour, often nearly identical to the original models. Notably, when differences do arise, they likely reflect the fact that many of the models are very finely tuned. Overall, this standard formulation facilitates be er understanding and comparisons among ion channel models, as well as reusability of models through easy functional translation between simulation languages. Additionally, our analysis allows for a direct comparison of models based on parameter settings, and can be used to make new observations about the space of ion channel kinetics across different ion channel subtypes, neuron types and species.

## O14 A priori identifiability of a binomial synapse

### Camille Gontier^1^, Jean-Pascal Pfister^2^

#### ^1^University of Bern, Department of Physiology, Bern, France; ^2^University of Bern, Department of Physiology, Bern, Switzerland

##### **Correspondence:** Camille Gontier (gontier@pyl.unibe.ch)

*BMC Neuroscience* 2019, **20(Suppl 1)**:O14

Synapses are highly stochastic transmission units. A classical model describing this transmission is called the binomial model [1], which assumes that there are N independent release sites, each having the same release probability p; and that each vesicle release gives rise to a quantal current q. The parameters of the binomial model (N, p, q, and the recording noise) can be estimated from postsynaptic responses, either by following a maximum-likelihood approach [2] or by computing the posterior distribution over the parameters [3].

But these estimates might be subject to parameter identifiability issues. This uncertainty of the parameter estimates is usually assessed a posteriori from recorded data, for instance by using re-sampling procedure such as parametric bootstrap.

Here, we propose a methodology for a priori quantifying the structural identifiability of the parameters. A lower bound on the error of parameter estimates can be obtained analytically using the Cramer-Rao bound. Instead of simply assessing a posteriori the validity of their parameter estimates, it is thus possible for experimentalists to select a priori a lower bound on the standard deviation of the estimates and to select the number of data points and to tune the level of noise accordingly.

Besides parameter identifiability, another critical issue is the so-called model identifiability, i.e. the possibility, given a certain number of data points T and a certain level of measurement noise, to find the model of synapse that fits our data the best. For instance, when observing discrete peaks on the histogram of post-synaptic currents, one might be tempted to conclude that the binomial model (“multi-quantal hypothesis”) is the best one to fit the data. However, these peaks might actually be artifacts due to noisy or scarce data points, and data might be best explained by a simpler Gaussian distribution (“uni-quantal hypothesis”).

Model selection tools are classically used to determine a posteriori which model is the best one to fit a data set, but little is known on the a priori possibility (in terms of number of data points or recording noise) to discriminate the binomial model against a simpler distribution.

We compute an analytical identifiability domain for which the binomial model is correctly identified (Fig. 1), and we verify it by simulations. Our proposed methodology can be further extended and applied to other models of synaptic transmission, allowing to define and quantitatively assess a priori the experimental conditions to reliably fit the model parameters as well as to test hypotheses on the desired model compared to simpler versions.

**Fig. 1:**
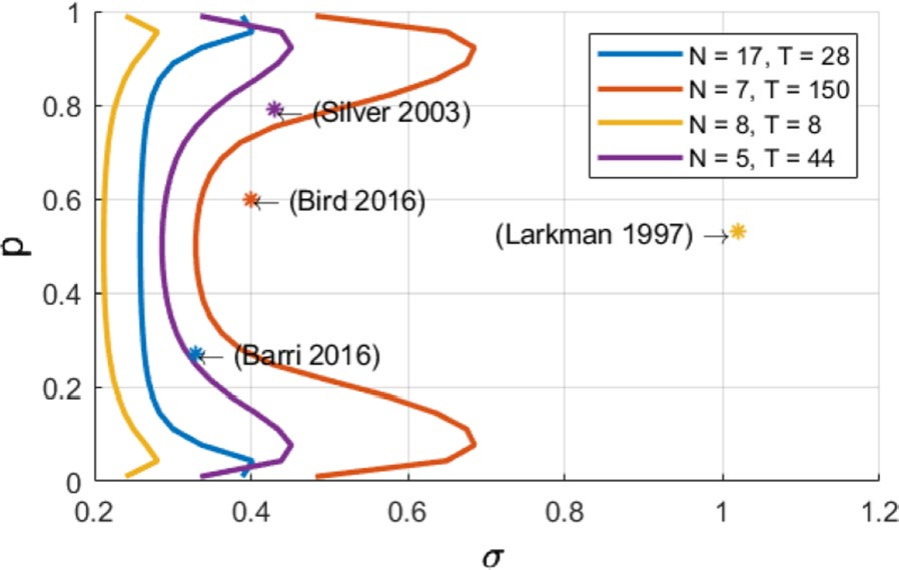
Published estimates of binomial parameters (dots), and corresponding identifiability domains (solid lines: the model is identifiable if, for a given release probability p, the recording noise does not exceed sigma). Applying our analysis to fitted parameters of the binomial model found in previous studies, we find that none of them are in the parameter range that would make the model identifiable

In conclusion, our approach aims at providing experimentalists objectives for experimental design on the required number of data points and on the maximally acceptable recording noise. This approach allows to optimize experimental design, draw more robust conclusions on the validity of the parameter estimates, and correctly validate hypotheses on the binomial model.

**References**

1. Katz B. The release of neural transmitter substances. *Liverpool University Press* (1969): 5–39.
2. Barri A, Wang Y, Hansel D, Mongillo G. Quantifying repetitive transmission at chemical synapses: a generative-model approach. *eNeuro* 2016 Mar;3(2).
3. Bird AD, Wall MJ, Richardson MJ. Bayesian inference of synaptic quantal parameters from correlated vesicle release. *Frontiers in computational neuroscience* 2016 Nov 25; 10:116.

## O15 A flexible, fast and systematic method to obtain reduced compartmental models.

### Willem Wybo, Walter Senn

#### University of Bern, Department of Physiology, Bern, Switzerland

##### **Correspondence:** Willem Wybo (willem.a.m.wybo@gmail.com)

*BMC Neuroscience* 2019, **20(Suppl 1)**:O15

Most input signals received by neurons in the brain impinge on their dendritic trees. Before being transmitted downstream as action potential (AP) output, the dendritic tree performs a variety of computations on these signals that are vital to normal behavioural function [3, 8]. In most modelling studies however, dendrites are omitted due the cost associated with simulating them. Biophysical neuron models can contain thousands of compartments, rendering it infeasible to employ these models in meaningful computational tasks. Thus, to understand the role of dendritic computations in networks of neurons, it is necessary to simplify biophysical neuron models. Previous work has either explored advanced mathematical reduction techniques [6, 10] or has relied on ad-hoc simplifications to reduce compartment numbers [11]. Both of these approaches have inherent difficulties that prevent widespread adoption: advanced mathematical techniques cannot be implemented with standard simulation tools such as NEURON [2] or BRIAN [4], whereas ad-hoc methods are tailored to the problem at hand and generalize poorly. Here, we present an approach that overcomes both of these hurdles: First, our method simply outputs standard compartmental models (Fig 1A). The models can thus be simulated with standard tools. Second, our method is systematic, as the parameters of the reduced compartmental models are optimized with a linear least square fitting procedure to reproduce the impedance matrix of the biophysical model (Fig 1B). This matrix relates input current to voltage, and thus determines the response properties of the neuron [9]. By fitting a reduced model to this matrix, we obtain the response properties of the full model at a vastly reduced computational cost. Furthermore, since we are solving a linear least squares problem, the fitting procedure is well-defined—as there is a single minimum to the error function—and computationally efficient. Our method is not constrained to passive neuron models. By linearizing ion channels around wisely chosen sets of expansion points, we can extend the fitting procedure to yield appropriately rescaled maximal conductances for these ion channels (Fig 1C). With these conductances, voltage and spike output can be predicted accurately (Fig 1D, E). Since our reduced models reproduce the response properties of the biophysical models, non-linear synaptic currents, such as NMDA, are also integrated correctly. Our models thus reproduce dendritic NMDA spikes (Fig 1F). Our method is also flexible, as any dendritic computation (that can be implemented in a biophysical model) can be reproduced by choosing an appropriate set of locations on the morphology at which to fit the impedance matrix. Direction selectivity [1] for instance, can be implemented by fitting a reduced model to a set of locations distributed on a linear branch, whereas independent subunits [5] can be implemented by choosing locations on separate dendritic subtrees. In conclusion, we have created a flexible linear fitting method to reduce non-linear biophysical models. To streamline the process of obtaining these reduced compartmental models, work is underway on a toolbox (https://github.com/WillemWybo/NEAT) that automatizes the impedance matrix calculation and fitting process.

**Fig. 1:**
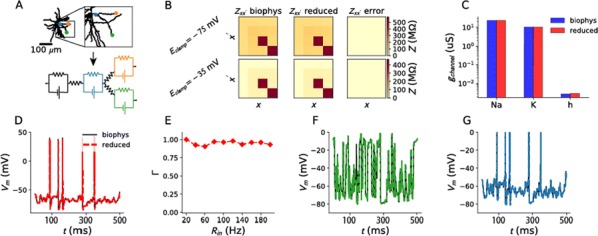
**a** Reduction of branch of stellate cell with compartments at 4 locations. **b** Biophysical (left) and reduced (middle) impedance matrices and error (right) at two holding potentials (top–bottom). **c** Somatic conductances. **d** Somatic voltage. **e** Spike coincidence factor between both models (1: perfect coincidence, 0: no coincidence—4 ms window). F res. **g** Same as **d**, but for green resp. blue site

**References**

1. Branco T, Clark B, Hausser M. Dendritic discrimination of temporal input sequences in cortical neurons. *Science Signaling* 2010, Sept:1671–1675.
2. Carnevale NT, Hines ML. *The NEURON book* 2004.
3. Cichon J, Gan WB. Branch-specific dendritic Ca2+ spikes cause persistent synaptic plasticity. *Nature* 2015, 520(7546):180–185.
4. Goodman DFM, Brette R. The Brian simulator. *Frontiers in neuroscience* 2009, 3(2):192– 7.
5. Häusser M, Mel B. Dendrites: bug or feature? *Current Opinion in Neurobiology* 2003, 13(3):372–383.
6. Kellems AR, Chaturantabut S, Sorensen DC, Cox SJ. Morphologically accurate reduced order modeling of spiking neurons. *Journal of computational neuroscience* 2010, 28(3):477–94.
7. Koch C, Poggio T. A simple algorithm for solving the cable equation in dendritic trees of arbitrary geometry. *Journal of neuroscience methods* 1985, 12(4):303–315.
8. Takahashi N, Oertner TG, Hegemann P, Larkum ME. Active cortical dendrites modulate perception. *Science* 2016, 354(6319):1587–90.
9. Wybo WA, Torben-Nielsen B, Nevian T, Gewaltig MO. Electrical Compartmentalization in Neurons. *Cell Reports* 2019, 26(7):1759–1773.e7.
10. Wybo WAM, Boccalini D, Torben-Nielsen B, Gewaltig MO. A Sparse Reformulation of the Green’s Function Formalism Allows Efficient Simulations of Morphological Neuron Models. *Neural computation* 2015, 27(12):2587–622.
11. Traub RD, Pais I, Bibbig A, et al. Transient depression of excitatory synapses on interneurons contributes to epileptiform bursts during gamma oscillations in the mouse hippocampal slice. *Journal of neurophysiology* 2005 Aug;94(2):1225–35.

## O16 An exact firing rate model reveals the differential effects of chemical versus electrical synapses in spiking networks

### Ernest Montbrió^1^, Alex Roxin^2^, Federico Devalle^1^, Bastian Pietras^3^, Andreas Daffertshofer^3^

#### ^1^Universitat Pompeu Fabra, Department of Information and Communication Technologies, Barcelona, Spain; ^2^Centre de Recerca Matemàtica, Barcelona, Spain; ^3^Vrije Universiteit Amsterdam, Behavioral and Movement Sciences, Amsterdam, Netherlands

##### **Correspondence:** Alex Roxin (aroxin@crm.cat)

*BMC Neuroscience* 2019, **20(Suppl 1)**:O16

Chemical and electrical synapses shape the collective dynamics of neuronal networks. Numerous theoretical studies have investigated how, separately, each of these types of synapses contributes to the generation of neuronal oscillations, but their combined effect is less understood. In part this is due to the impossibility of traditional neuronal firing rate models to include electrical synapses.

Here we perform a comparative analysis of the dynamics of heterogeneous populations of integrate-and-fire neurons with chemical, electrical, and both chemical and electrical coupling. In the thermodynamic limit, we show that the population’s mean-field dynamics is exactly described by a system of two ordinary differential equations for the center and the width of the distribution of membrane potentials —or, equivalently, for the population-mean membrane potential and firing rate. These firing rate equations exactly describe, in a unified framework, the collective dynamics of the ensemble of spiking neurons, and reveal that both chemical and electrical coupling are mediated by the population firing rate. Moreover, while chemical coupling shifts the center of the distribution of membrane potentials, electrical coupling tends to reduce the width of this distribution promoting the emergence of synchronization.

The firing rate equations are highly amenable to analysis, and allow us to obtain exact formulas for all the fixed points and their bifurcations. We find that the phase diagram for networks with instantaneous chemical synapses are characterized by a codimension-two Cusp point, and by the presence of persistent states for strong excitatory coupling. In contrast, phase diagrams for electrically coupled networks is determined by a Takens-Bogdanov codimension-two point, which entails the presence of oscillations and greatly reduces the presence of persistent states. Oscillations arise either via a Saddle-Node-Invariant-Circle bifurcation, or through a supercritical Hopf bifurcation. Near the Hopf bifurcation the frequency of the emerging oscillations coincides with the most likely firing frequency of the network. Only the presence of chemical coupling allows to shift (increase for excitation, and decrease for inhibition) the frequency of these oscillations. Finally, we show that the Takens-Bogdanov bifurcation scenario is generically present in networks with both chemical and electrical coupling.

**Acknowledgement:** We acknowledge support by the European Union’s Horizon 2020 research and innovation programme under the Marie Skłodowska Curie grant agreement No. 642563.

## O17 Graph-filtered temporal dictionary learning for calcium imaging analysis

### Gal Mishne^1^, Benjamin Scott^2^, Stephan Thiberge^4^, Nathan Cermak^3^, Jackie Schiller^3^, Carlos Brody^4^, David W. Tank^4^, Adam Charles^4^

#### ^1^Yale University, Applied Math, New Haven, CT, United States of America; ^2^Boston University, Boston, United States of America; ^3^Technion, Haifa, Israel; ^4^Princeton University, Department of Neuroscience, Princeton, NJ, United States of America

##### **Correspondence:** Gal Mishne (gal.mishne@yale.edu)

*BMC Neuroscience* 2019, **20(Suppl 1)**:O17

Optical calcium imaging is a versatile imaging modality that permits the recording of neural activity, including single dendrites and spines, deep neural populations using two-photon microscopy, and wide-field recordings of entire cortical surfaces. To utilize calcium imaging, the temporal fluorescence fluctuations of each component (e.g., spines, neurons or brain regions) must be extracted from the full video. Traditional segmentation methods used spatial information to extract regions of interest (ROIs), and then projected the data onto the ROIs to calculate the time-traces [1]. Current methods typically use a combination of both a-priori spatial and temporal statistics to isolate each fluorescing source in the data, along with the corresponding time-traces [2, 3]. Such methods often rely on strong spatial regularization and temporal priors that can bias time-trace estimation and that do not translate well across imaging scales.

We propose to instead model how the time-traces generate the data, using only weak spatial information to relate per-pixel generative models across a field-of-view. Our method, based on spatially-filtered Laplacian-scale mixture models [4,5], introduces a novel non-local spatial smoothing and additional regularization to the dictionary learning framework, where the learned dictionary consists of the fluorescing components’ time-traces.

We demonstrate on synthetic and real calcium imaging data at different scales that our solution has advantages regarding initialization, implicitly inferring number of neurons and simultaneously detecting different neuronal types (Fig. 1). For population data, we compare our method to a current state-of-the-art algorithm, Suite2p, on the publicly available Neurofinder dataset (Fig. 1C). The lack of strong spatial contiguity constraints allows our model to isolate both disconnected portions of the same neuron, as well as small components that would otherwise be over-shadowed by larger components. In the latter case, this is important as such configurations can easily cause false transients which can be scientifically misleading. On dendritic data our method isolates spines and dendritic firing modes (Fig. 1D). Finally, our method can partition widefield data [6] in to a small number of components that capture the scientifically relevant neural activity (Fig. 1E-F).

**Fig. 1:**
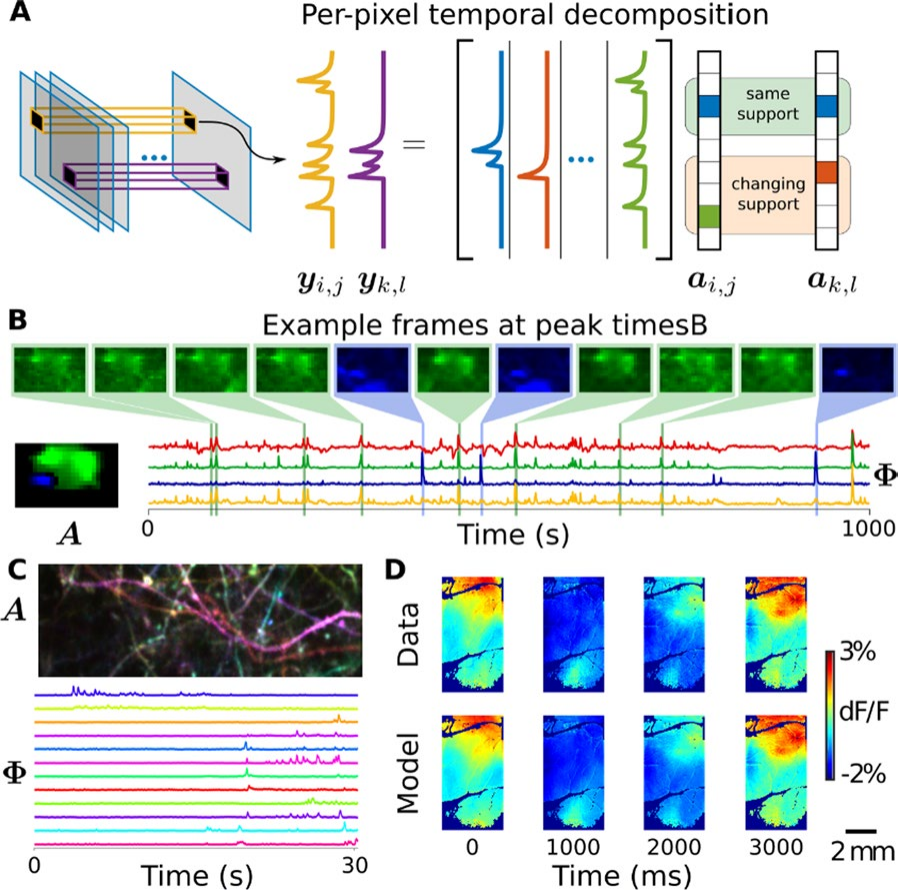
**a** Our method uses a per-pixel generative model with non-local spatially correlated coefficients. **b** Temporal DL finds subtle features in the Neurofinder dataset. For example, shown here is an apical dendrite (blue) significantly overlapping with a soma (green) was isolated. Manually labeled soma (yellow) and Suite2p (red) do not account for the apical, resulting in contaminated time-traces. **c** Applications to dendritic data extracts both dendrite and spine activity (bottom), as seen by the spatial maps where each component is colored differently (top). **d** In widefield imaging, the reconstructed movie recapitulates the behaviorally-triggered dynamics [6], demonstrating that it captures the scientifically-relevant activity

**Acknowledgments:** M is supported by NIH NIBIB and NINDS (grant R01EB026936).

**References**

1. Mukamel EA, Nimmerjahn A, Schnitzer MJ. Automated analysis of cellular signals from large-scale calcium imaging data. *Neuron* 2009, 63, 747–760.
2. Pachitariu M, et al. Suite2p: beyond 10,000 neurons with standard two-photon microscopy. *bioRxiv*, 2016. 061507
3. Pnevmatikakis EA, et al. Simultaneous denoising, deconvolution, and demixing of calcium imaging data. *Neuron* 2016, 89, 285–299.
4. Garrigues P, Olshausen BA. Group sparse coding with a laplacian scale mixture prior. *NIPS* 2010, 676–684.
5. Charles AS, Rozell CJ. Spectral superresolution of hyperspectral imagery using reweighted l1 spatial filtering. *IEEE Geosci. Remote Sens. Lett.* 2014, 11, 602–606.
6. Scott BB, et al. Imaging Cortical Dynamics in GCaMP Transgenic Rats with a Head-Mounted Widefield Macroscope, *Neuron* 2018, 100, 1045–1058.

## O18 Drift-resistant, real-time spike sorting based on anatomical similarity for high channel-count silicon probes

### James Jun^1^, Jeremy Magland^1^, Catalin Mitelut^2^, Alex Barnett^1^

#### ^1^Flatiron Institute, Center for Computational Mathematics, New York, NJ, United States of America; ^2^Columbia University, Department of Statistics, New York, United States of America

##### **Correspondence:** James Jun (jjun@flatironinstitute.org)

*BMC Neuroscience* 2019, **20(Suppl 1)**:O18

Extracellular electrophysiology records a mixture of neural population activity at a single spike resolution. In order to resolve individual cellular activities, a spike-sorting operation groups together similar spike waveforms distributed at a subset of electrodes adjacent to each neuron. Penetrating micro-electrode arrays are widely used to measure the spiking activities from behaving animals, but silicon probes can be drifted in the brain due to animal movements or tissue relaxation following a probe penetration. The probe drift issue results in errors in conventional spike sorting operations that assumes stationarity in spike waveforms and amplitudes. Some of the latest silicon probes [1] offer a whole-shank coverage of closely-spaced electrode arrays, which can continually capture the spikes generated by neurons moving along the probe axis. We introduce a drift-resistant spike sorting algorithm for high channel-count, high-density silicon probe, which is designed to handle gradual and rapid random probe movements. IronClust takes advantage of the fact that a drifting probe revisits the same anatomical locations at various times. We apply a density-based clustering by grouping a temporal subset of the spiking events where the probe occupied similar anatomical locations. Anatomical similarities between a disjoint set of time bins are determined by calculating the activity histograms, which capture the spatial structures in the spike amplitude distribution based on the peak spike amplitudes on each electrode. For each spiking event, the clustering algorithm (DPCLUS [2]) computes the distances to a subset of its neighbors selected by their peak channel locations and the anatomical similarity. Based on the k-nearest neighbors [3], the clustering algorithm finds the density peaks based on the local density values and the nearest distances to the higher-density neighbors, and recursively propagates the cluster memberships toward a decreasing density gradient, The accuracy of our algorithm was evaluated using validation datasets generated using a biophysically detailed neural network simulator (BioNet [4]), which generated three scenarios including stationary, slow monotonic drift, and fast random drift cases. IronClust achieved ~8% error on the stationary dataset, and ~10% error on the gradual or random drift datasets, which significantly outperformed existing algorithms (Fig. 1). We also found that additional columns of electrodes improve the sorting accuracy in all cases. IronClust achieved over 11x of the real-time speed using GPU, and over twice faster than other leading algorithm. In conclusion, we realized an accurate and scalable spike sorting operation resistant to probe drift by taking advantage of an anatomically-aware clustering and parallel computing.

**Fig. 1:**
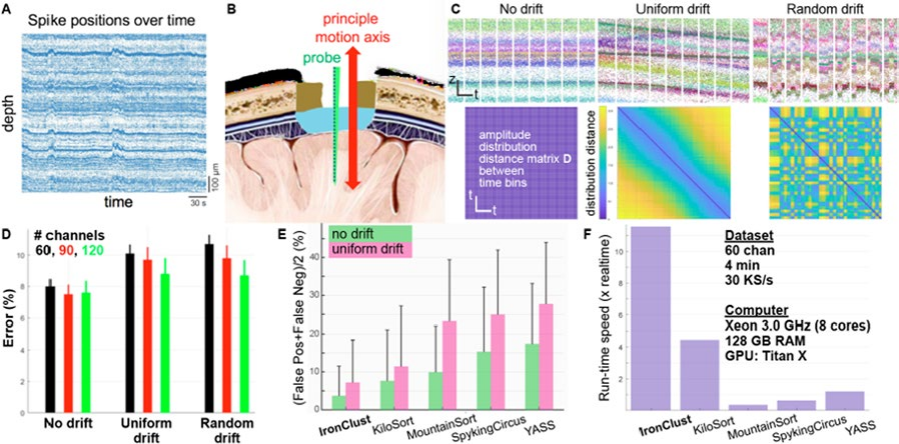
**a** Probe drift causes coherent shifts in the spike positions preserving the anatomical structure. **b** Principal probe movement occurs along the probe axis. **c** Three drift scenarios and the anatomical similarity matrices between time bins. **d** Clustering errors for various drift scenarios and electrode layouts. **e** Accuracy comparison. **f** Speed comparison between multiple sorters

**References**

1. Jun JJ et al. Fully integrated silicon probes for high-density recording of neural activity. *Nature* 2017 Nov;551(7679):232.
2. Rodriguez A, Laio A. Clustering by fast search and find of density peaks. *Science* 2014 Jun 27;344(6191):1492–6.
3. Rodriguez A, d’Errico M, Facco E, Laio A. Computing the free energy without collective variables. *Journal of chemical theory and computation* 2018 Feb 5;14(3):1206–15.
4. Gratiy SL, et al. BioNet: A Python interface to NEURON for modeling large-scale networks. *PloS one* 2018 Aug 2;13(8):e0201630.

## P1 Promoting community processes and actions to make neuroscience FAIR

### Malin Sandström, Mathew Abrams

#### INCF, INCF Secretariat, Stockholm, Sweden

##### **Correspondence:** Malin Sandström (malin.sandstrom@incf.org)

*BMC Neuroscience* 2019, **20(Suppl 1)**:P1

The FAIR data principles were established as a general framework to facilitate knowledge discovery in research. Since the FAIR data principles are only guidelines, it is up to each domain to establish the standards and best practices (SBPs) that fulfill the principles. Thus, INCF is working with the community to develop, endorse, and adopt SBPs in neuroscience.

Develop: Connecting communities to support FAIR(er) practices

INCF provides 3 forums in which community members can come together to develop SBPs: Special Interest Groups (SIGs), Working Groups (WGs), and the INCF Assembly. SIGs are composed of a group of community members with the same interest, who gather and self-organize around tools, data, and community needs in a specific area. The SIGs will also serve as the focus for getting agreement and community buy-in on the use of these standards and best practices. INCF WGs are extensions of SIGs that receive funding from INCF to develop or extend existing SBPs, for example to support additional data types, or the development of a new SBP. The WG plan must include a plan for gathering appropriate input from the membership and the community. for example to support additional data types, or the development of a new SBP.

Endorse: Formalized standards endorsement process

The endorsement process is a continuous loop of feedback from the committee and the community to the developer(s) of the SBPs (e.g. PyNN and NeuroML [1,2]). Developers submit their SBPs for endorsement to the INCF SBP Committee who in turn vets the merit of the SBPs and publishes a report on the proposed standard covering openness, FAIRness, testing and implementation, governance, adoption and use, stability, and support. Then community is invited to comment during a 60-day period before the committee takes the final decision. Endorsed SBPs are then made available on incf.org and promoted to the community, to journals, and to funders through INCF’s training and outreach efforts.

Promote Adoption: Outreach and training

To promote adoption, INCF offers the yearly INCF Assembly where SIGs and WGs can present their work and engage the wider community. Training materials are also integrated into the INCF TrainingSpace, a platform linking world-class neuroinformatics training resources, developed by INCF in collaboration with its partners, and existing community resources. In addition to outreach and training, INCF also developed KnowledgeSpace, a community-based encyclopedia for neuroscience that links brain research concepts to the data, models, and literature that supports them, demonstrating how SBPs can facilitate linking brain research concepts with data, models and literature from around the world. It is an open project and welcomes participation and contributions from members of the global research community. KS is the result of recommendations from a community workshop held by the INCF Program on Ontologies of Neural Structures in 2012.

**References**

1. Martone M, Das S, Goscinski W, et al. Call for community review of NeuroML — A Model Description Language for Computational Neuroscience [version 1; not peer reviewed] *F1000Research* 2019, 8:75 (document) (10.7490/f1000research.1116398.1).
2. Martone M, Das S, Goscinski W, et al. Call for community review of PyNN — A simulator-independent language for building neuronal network models [version 1; not peer reviewed]. *F1000Research* 2019, 8:74 (document) (10.7490/f1000research.1116399.1).

## P2 Ring integrator model of the head direction cells

### Anu Aggarwal

#### Grand Valley State University, Electrical and Computer Engineering, Grand Rapids, MI, United States of America

##### **Correspondence:** Anu Aggarwal (aaagganu@gmail.com)

*BMC Neuroscience* 2019, **20(Suppl 1)**:P2

Head direction (HD) cells have been demonstrated in the post subiculum [1, 2] of the hippocampal formation of the brain. Ensembles of the HD cells provide information about heading direction during spatial navigation. An Attractor Dynamic model [3] has been proposed to explain the unique firing patterns of the head direction cells. Here, we present a novel Ring Integrator model of the HD cells. This model is an improvement over the Attractor Dynamic model as it achieves the same functionality with fewer neurons and explains how the HD cells align to orienting cues.

**References**

1. Taube JS, Muller RU, Ranck JB. Head-direction cells recorded from the postsubiculum in freely moving rats. I. Description and quantitative analysis. *Journal of Neuroscience* 1990 Feb 1;10(2):420–35.
2. Taube JS, Muller RU, Ranck JB. Head-direction cells recorded from the postsubiculum in freely moving rats. II. Effects of environmental manipulations. *Journal of Neuroscience* 1990 Feb 1;10(2):436–47.
3. McNaughton BL, Battaglia FP, Jensen O, Moser EI, Moser MB. Path integration and the neural basis of the ‘cognitive map’. *Nature Reviews Neuroscience* 2006 Aug;7(8):663.

## P3 Parametric modulation of distractor filtering in visuospatial working memory.

### Davd Bestue^1^, Albert Compte^2^, Torkel Klingberg^3^, Rita Almeida^4^

#### ^1^IDIBAPS, Barcelona, Spain; ^2^IDIBAPS, Systems Neuroscience, Barcelona, Spain; ^3^Karolinksa Institutet, Stockholm, Sweden; ^4^Stockholm University, Stockholm, Sweden

##### **Correspondence:** Davd Bestue (davidsanchezbestue@hotmail.com)

*BMC Neuroscience* 2019, **20(Suppl 1)**:P3

Although distractor filtering has been long identified as a fundamental mechanism to achieve an efficient management of working memory, there are not many tasks where distractors are parametrically modulated both in the temporal and the similarity domain simultaneously. Here, 21 subjects participated in a visuospatial working memory task (vsWM) where distractors could be presented prospectively or retrospectively at two different delay times (200 and 7000 ms). Moreover, distractors were presented close or far away from the target. As expected, changes in the temporal and the similarity domain induced different distraction behaviours. In the similarity domain, we observed that close-by distractors induced an attractive bias while far distractors induced a repulsive one. Interestingly, this pattern of biases occurred both for prospective and retrospective distractors, suggesting common mechanisms of interference with the behaviorally relevant target. This result is in line with a previously validated bump-attractor model where diffusing bumps of neural activity attract or repel each other in the delay period [1]. In the temporal domain, we found a stronger effect for prospective distractors and short delays (200ms). Intriguingly, we observed that a retrospective distractor at 7000 ms also affected behavior, suggesting that irrelevant distractor memory traces can last longer than previously considered in computational models. One possibility is that persistent-activity based mechanisms underpin target storage while synaptic-based mechanisms underlie distractor memory traces. To gather support for this idea, we ran the same experiment with a 3T fMRI in 6 participants. Based on previous studies where sensory areas were not resistant to distractors [2], we hypothesized that sensory areas would represent all visual stimuli while associative areas like IPS would subserve memory-for-target function. Importantly, the synaptic hypothesis for distractor storage would predict that despite the behavioral evidence for retrospective distractor memory in this task, retrospective distractors would not be represented in the activity of either area, despite strong representations of the target. To test this, we will map parametric behavioral outputs into physiological activity readouts [3] for the different distractor conditions and we will explore the biological mechanism of distractor storage in working memory by comparing distractor storage in the retrospective 7000 ms condition with target storage in the absence of distractors. All together, these results open the door to an integrative model of working memory where different neural mechanisms and multiple brain regions are taken into account.

**References**

1. Almeida R, Barbosa J, Compte A. Neural circuit basis of visuo-spatial working memory precision: a computational and behavioral study. *Journal of Neurophysiology* 2015 Jul 15;114(3):1806–18.
2. Bettencourt KC, Xu Y. Decoding the content of visual short-term memory under distraction in occipital and parietal areas. *Nature Neuroscience* 2016 Jan;19(1):150.
3. Ester EF, Sprague TC, Serences JT. Parietal and frontal cortex encode stimulus-specific mnemonic representations during visual working memory. *Neuron* 2015 Aug 19;87(4):893–905.

## P4 Dynamical phase transitions study in simulations of finite neurons network

### Cecilia Romaro^1^, Fernando Najman^2^, Morgan Andre^2^

#### ^1^University of São Paulo, Department of Physics, Ribeirão Preto, Brazil; ^2^University of São Paulo, Institute of Mathematics and Statistics, São Paulo, Brazil

##### **Correspondence:** Cecilia Romaro (cecilia.romaro@usp.br)

*BMC Neuroscience* 2019, **20(Suppl 1)**:P4

In [1], Ferrari et al. introduced a continuous time model for network of spiking neurons with binary membrane potential. It consists in an infinite system of interacting point processes. Each neuron in the one-dimensional lattice Z has two post-synaptic neurons, which are its two immediate neighbors. There is only two possible states for a given neuron, which are “active” or “quiescent” (1 or 0), and the neuron goes from “active” to “quiescent” either when it spikes, either when it is affected by the leakage effect, it goes from 0 to 1 when one of its presynaptic neurons spikes. For a given neuron the spikes are modeled as the events of a Poisson process of parameter 1, while the leakage events are modeled as the events of a Poisson process of some positive parameter gamma γ, all the processes being mutually independents. It was shown that this model presents a phase transition with respect to the parameter γ. This means that there exists a critical value for the parameter γ, denoted γc, such that, when γ>γc all neurons will once for all end up in the “quiescent” state with probability one; and when γ<γc there is a positive probability that the neurons will come back to the “active” state infinitely often.

However, when modeling the brain, it is usual to work with a necessarily finite number of neurons. Thus, we consider a finite version of the infinite system: instead of a process defined on entire lattice Z, we consider a version of the process defined on the finite window {−N, −N + 1, …, N − 1 N} (the number of neurons is therefore 2N + 1). When the number of neurons is finite we know by elementary results about Markov chains that the absorbent state, where all neurons are “quiescent”, will necessarily be reached in some finite time for any value of γ. The time t spent to reach the absorbent state depends on the network number of neuron 2N + 1 and the arbitrary parameter γ. For example, around 10^7^ random numbers were picked up until the network reached the absorbent state for N = 100 and γ = 0.375, but around 10^9^ random numbers were required when N was increased to 500 (Fig. 1).

**Fig. 1:**
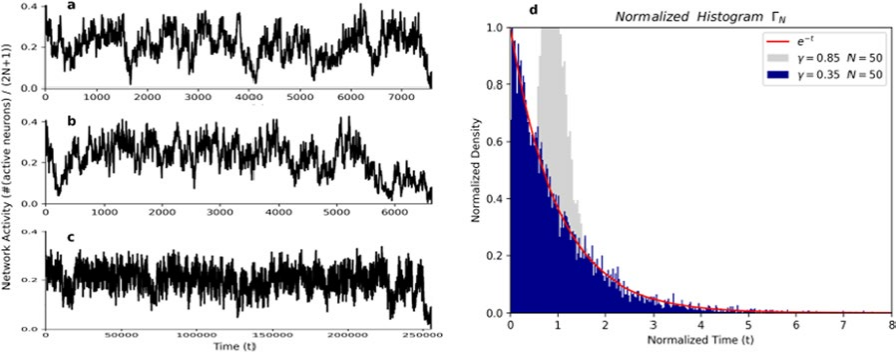
The activity of network with (**a** and **b**) N = 100 or **c** N = 500 and γ = 0.375. Around 10^7^ (**a** and **b** and 10^9^) **c** random numbers were required until the network reaches the absorbent state. **d** Histogram normalized of the time t of extinction for N = 50 and gamma = 0.35 for 10,000 turns compared with the function exp(−t) in red

So, we conjecture that, for a γ less than the critical gamma γc, the finite model presents a dynamical phase transition, as first defined in [2]. By this we mean that for a finite number of neurons, the distribution of the time of extinction (T(N,γ)) re-normalized (divided by its expectation) converges in distribution to an exponential random variable of parameter 1 when the number of neurons grows (N→∞). To back up our conjecture we build up the present model in python and run it 10,000 turns for N = (10, 50, 100, 500, 1000), J = (0.40, 0.35, 0.30) and plot the normalized histogram. The Fig. 1d shows the normalized histogram of the time of extinction for N = 50 (101 neurons) and J = 0.35 for 10,000 simulations and the function e-t in red.

**Acknowledgements:** This work was produced as part of the activities of FAPESP Research, Disseminations and Innovation Center for Neuromathematics (Grant 2013/07699-0, S. Paulo Research Foundation).

**References**

1. Ferrari PA, Galves A, Grigorescu I, Löcherbach E. Phase transition for infinite systems of spiking neurons. *Journal of Statistical Physics* 2018 Sep 1;172(6):1564–75.
2. Cassandro M, Galves A, Picco P. Dynamical phase transitions in disordered systems: the study of a random walk model. *In Annales de l’IHP Physique théorique* 1991 (Vol. 55, No. 2, pp. 689–705).

## P5 Computational modeling of genetic contributions to excitability and neural coding in layer V pyramidal cells: applications to schizophrenia pathology

### Tuomo Mäki-Marttunen^1^, Gaute Einevoll^2^, Anna Devor^3^, William A. Phillips^4^, Anders M. Dale^3^, Ole A. Andreassen^5^

#### ^1^Simula Research Laboratory, Oslo, Norway; ^2^Norwegian University of Life Sciences, Faculty of Science and Technology, Aas, Norway; ^3^University of California, San Diego, Department of Neurosciences, La Jolla, United States of America; ^4^University of Stirling, Psychology, Faculty of Natural Sciences, Stirling, United Kingdom; ^5^University of Oslo, NORMENT, KG Jebsen Centre for Psychosis Research, Division of Mental Health and Addiction, Oslo, Norway

##### **Correspondence:** Tuomo Mäki-Marttunen (tuomo@simula.no)

*BMC Neuroscience* 2019, **20(Suppl 1)**:P5

Layer V pyramidal cells (L5PCs) extend their apical dendrites throughout the cortical thickness of the neocortex and integrate information from local and distant sources [1]. Alterations in the L5PC excitability and its ability to process context- and sensory drive-dependent inputs have been proposed to be a cause for hallucinations and other impairments of sensory perceptions related to mental disease [2]. In line with this hypothesis, genetic variants in voltage-gated ion channel-encoding genes and their altered expression have been associated with the risk of mental disorders [4]. In this work, we use computational models of L5PCs to systematically study the impact of small-effect variants on L5PC excitability and phenotypes associated with schizophrenia (SCZ).

An important aid in SCZ research is the set of biomarkers and endophenotypes that reflect the impaired neurophysiology and—unlike most of the symptoms of the disorder—are translatable to animal models. The deficit in prepulse inhibition (PPI) is one of the most robust endophenotypes. Although statistical genetics and genome-wide association studies (GWASs) have helped to make associations between gene variants and disease phenotypes, the mechanisms of PPI deficits and other circuit dysfunctions related to SCZ are incompletely understood at the cellular level. Following our previous work [3], we here study the effects ofSCZ-associated genes on PPI in a single neuron.

In this work, we aim at bridging the gap of knowledge between SCZ genetics and disease phenotypes by using biophysically detailed models to uncover the influence of SCZ-associated genes on integration of information in L5PCs. L5PC population displays a wide diversity of morphological and electrophysiological behaviours, which has been overlooked in most modeling studies. To capture this variability, we use two separate models for thick-tufted L5PCs with partly overlapping ion-channel mechanisms and modes of input-output relationships. Furthermore, we generate alternative models that capture a continuum of firing properties between those attained by the two models. We show that most of the effects of SCZ-associated variants reported in [3] are robust across different types of L5PCs. Further, to generalize the results to *in vivo*-like conditions, we show that the effects of these model variants on single-L5PC excitability and integration of inputs persist when the model neuron is stimulated with noisy inputs. We also show that the model variants alter the way L5PCs code the input information both in terms of output action potentials and intracellular [Ca2+], which could contribute to both altered activity in the downstream neurons and synaptic long-term potentiation. Taken together, our results show a wide diversity in how SCZ-associated voltage-gated ion channel-encoding genes affect input-output relationships in L5PCs, and our framework helps to predict how these relationships are correlated with each other. These findings indicate that SCZ-associated variants may alter the interaction between perisomatic and apical dendritic regions.

**References**

1. Hay E, Hill S, Schürmann F, Markram H, Segev I. Models of neocortical layer 5b pyramidal cells capturing a wide range of dendritic and perisomatic active properties. *PLoS Comput Biol* 7, 7(2011): e1002107.
2. Larkum M. A cellular mechanism for cortical associations: an organizing principle for the cerebral cortex. *Trends in Neurosciences* 36, 3(2013): 141–151.
3. Mäki-Marttunen T, Halnes G, Devor A, et al. Functional effects of schizophrenia-linked genetic variants on intrinsic single-neuron excitability: a modeling study. *Biol Psychiatry: Cogn Neurosci Neuroim* 1, 1(2016): 49–59.
4. Ripke S, Neale BM, Corvin A, Walters JT, et al. Biological insights from 108 schizophrenia-associated genetic loci. *Nature* 511, 7510(2014): 421.

## P6 Spatiotemporal dynamics underlying successful cognitive therapy for posttraumatic stress disorder

### Marina Charquero^1^, Morten L Kringelbach^1^, Birgit Kleim^2^, Christian Ruff^3^, Steven C.R Williams^4^, Mark Woolrich^5^, Vidaurre Diego^5^, Ehlers Anke^6^

#### ^1^University of Oxford, Department of Psychiatry, Oxford, United Kingdom; ^2^University of Zurich, Psychotherapy and Psychosomatics, Zurich, Switzerland; ^3^University of Zurich, Zurich Center for Neuroeconomics (ZNE), Department of Economics, Zurich, Switzerland; ^4^King’s College London, Neuroimaging Department, London, United Kingdom; ^5^University of Oxford, Wellcome Trust Centre for Integrative NeuroImaging, Oxford Centre for Human Brain Activity (OHBA), Oxford, United Kingdom; ^6^University of Oxford, Oxford Centre for Anxiety Disorders and Trauma, Department of Experimental Psychology, Oxford, United Kingdom

##### **Correspondence:** Marina Charquero (marina.charqueroballester@psych.ox.ac.uk)

*BMC Neuroscience* 2019, **20(Suppl 1)**:P6

Cognitive therapy for posttraumatic stress disorder (CT-PTSD) is one of the evidence-based psychological treatments. However, there are currently no fMRI studies investigating the temporal dynamics of brain network activation associated with successful cognitive therapy for PTSD. In this study, we used a newly developed data-driven approach to investigate the dynamics of brain function [1] underlying PTSD recovery with CT-PTSD [2].

Participants (43 PTSD, 30 remitted (14 pre & post CT-PTSD, 16 only post CT-PTSD), 8 waiting list and 15 healthy controls) underwent an fMRI protocol on a 1.5T Siemens Scanner using an echoplanar protocol (TR/TE 2400/40). The task consisted of trauma-related or neutral pictures presented in a semi-randomised block design. Data was preprocessed using FSL and FIX and nonlinearly registered to MNI space. Mean BOLD timeseries were estimated using the Shen functional atlas [3]. A Hidden Markov Model [1] was applied to estimate 7 states, each defined by a certain pattern of activation. The amount of total time spent in each network state (i.e., fractional occupancy) was computed separately for each of the two conditions: neutral and trauma-related pictures.

The states can be described as patterns of above- and below-average activation overlapping with functional (e.g., visual ventral stream) or resting-state networks (e.g., default mode network (DMN)). Results show that two DMN-related states, anatomically involving the medial temporal and the dorsomedial prefrontal DMN subsystems [4], had decreased fractional occupancies in PTSD in contrast to both healthy controls and remitted PTSD. No other states showed significant differences between groups. Importantly, there were no differences between PTSD before and after a waiting list condition (Fig 1). Furthermore, flashback qualities of intrusive memories were negatively related to the time spent in the medial temporal DMN as well as positively correlated with the time spent in ventral visual and salience states.

**Fig. 1:**
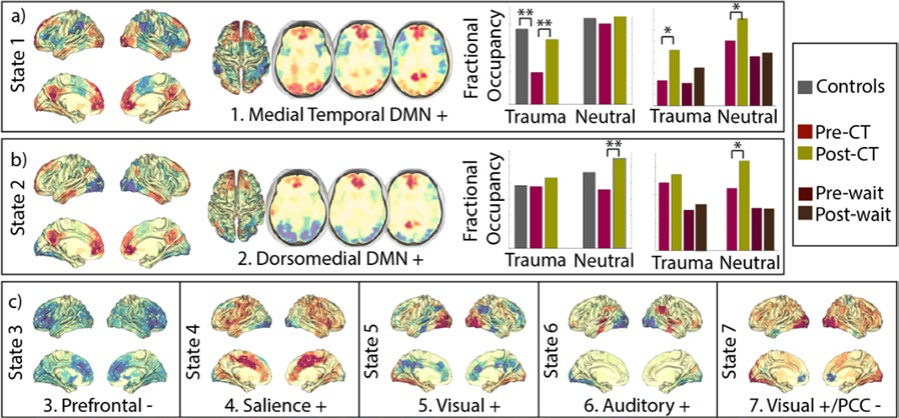
**a**, **b** Participants with PTSD spend less time visiting two DMN-related states in contrast to healthy controls and/or remitted PTSD, but no significant differences were found between visit1 and visit 2 of participants assigned to the waiting list condition. **c** No significant differences were found between groups for any of the other states. *pval < 0.05; **p 0.05 < after FDR

Recent work suggests that two subcomponents of the DMN, the medial temporal DMN and the dorsomedial prefrontal DMN, appear to be related to memory contextualisation and mentalizing about self and others, respectively [e.g. 4]. Our results show that the brains of participants with PTSD spend less time in states related to these two subcomponents before but not after successful therapy. This fits well with the cognitive theory suggested by [5], according to which PTSD results from: 1) disturbance of autobiographical memory characterised by poor contextualisation 2) excessively negative and threatening interpretations of one’s own and other people’s reactions to the trauma.

**References**

1. Vidaurre D, Abeysuriya R, Becker R, et al. Discovering dynamic brain networks from big data in rest and task. *Neuroimage* 2018 Oct 15;180:646–56.
2. Ehlers A, Clark DM, Hackmann A, McManus F, Fennell M. Cognitive therapy for post-traumatic stress disorder: development and evaluation. *Behaviour research and therapy* 2005 Apr 1;43(4):413–31.
3. Shen X, Tokoglu F, Papademetris X, Constable RT. Groupwise whole-brain parcellation from resting-state fMRI data for network node identification. *Neuroimage* 2013 Nov 15;82:403–15.
4. Andrews‐Hanna JR, Smallwood J, Spreng RN. The default network and self‐generated thought: component processes, dynamic control, and clinical relevance. *Annals of the New York Academy of Sciences* 2014 May 1;1316(1):29–52.
5. Ehlers A, Clark DM. A cognitive model of posttraumatic stress disorder. *Behaviour research and therapy* 2000 Apr 1;38(4):319–45.

## P7 Experiments and modeling of NMDA plateau potentials in cortical pyramidal neurons

### Peng Gao^1^, Joe Graham^2^, Wen-Liang Zhou^1^, Jinyoung Jang^1^, Sergio Angulo^2^, Salvador Dura-Bernal^2^, Michael Hines^3^, William W Lytton^2^, Srdjan Antic^1^

#### ^1^University of Connecticut Health Center, Department of Neuroscience, Farmington, CT, United States of America; ^2^SUNY Downstate Medical Center, Department of Physiology and Pharmacology, Brooklyn, NY, United States of America; ^3^Yale University, Department of Neuroscience, CT, United States of America

##### **Correspondence:** Joe Graham (joe.w.graham@gmail.com)

*BMC Neuroscience* 2019, **20(Suppl 1)**:P7

Experiments have shown that application of glutamate near basal dendrites of cortical pyramidal neurons activates AMPA and NMDA receptors, which can result in dendritic plateau potentials: long-lasting depolarizations which spread into the soma, reducing the membrane time constant and bringing the cell closer to the spiking threshold. Utilizing a morphologically-detailed reconstruction of a Layer 5 pyramidal cell from prefrontal cortex, a Hodgkin-Huxley compartmental model was developed in NEURON. Synaptic AMPA/NMDA and extrasynaptic NMDA receptor models were placed on basal dendrites to explore plateau potentials. The properties of the model were tuned to match plateau potentials recorded by voltage-sensitive dye imaging in dendrites and whole-cell patch measurements in somata of prefrontal cortex pyramidal neurons from rat brain slices. The model was capable of reproducing experimental observations: a threshold for activation of the plateau, saturation of plateau amplitude with increasing glutamate application, depolarization of the soma by approximately 20 mV, and back-propagating action potential amplitude attenuation and time delay. The model predicted that membrane time constant is shortened during the plateau, that synaptic inputs are more effective during the plateau due to both depolarization and time constant change, the plateau durations are longer when activated by more distal dendritic segments, and that plateau initiation location can be predicted from somatic plateau amplitude. Dendritic plateaus induced by strong basilar dendrite stimulation can increase population synchrony produced by weak coherent stimulation in apical dendrites. The morphologically-detailed cell model was simplified while maintaining the observed plateau behavior and then utilized in cortical network models along with a previously-published inhibitory interneuron model. The network model simulations showed increased synchrony between cells during induced dendritic plateaus. These results support our hypothesis that dendritic plateaus provide a 200-500 ms time window during which a neuron is particularly excitable. At the network level, this predicts that sets of cells with simultaneous plateaus would provide an activated ensemble of responsive cells with increased firing. Synchronously spiking subsets of these cells would then create an embedded ensemble. This embedded ensemble would demonstrate a temporal code, at the same time as the activated (embedded) ensemble showed rate coding.

## P8 Systematic automated validation of detailed models of hippocampal neurons against electrophysiological data

### Sára Sáray^1^, Christian A Rössert^2^, Andrew Davison^3^, Eilif Muller^2^, Tamas Freund^4^, Szabolcs Kali^4^, Shailesh Appukuttan^3^

#### ^1^Faculty of Information Technology and Bionics, Pázmány Péter Catholic University, Hungary; ^2^École Polytechnique Fédérale de Lausanne, Blue Brain Project, Lausanne, Switzerland; ^3^Centre National de la Recherche Scientifique/Université Paris-Sud, Paris-Saclay Institute of Neuroscience, Gif-sur-Yvette, France; ^4^Institute of Experimental Medicine, Hungarian Academy of Sciences, Budapest, Hungary

##### **Correspondence:** Sára Sáray (saraysari@gmail.com)

*BMC Neuroscience* 2019, **20(Suppl 1)**:P8

Developing biophysically and anatomically detailed data-driven computational models of the different neuronal cell types and running simulations on them is becoming a more and more popular method in the neuroscience community to investigate the behaviour and to understand or predict the function of these neurons in the brain. Several computational and software tools have been developed to build detailed neuronal models, and there is an increasing body of experimental data from electrophysiological measurements that describe the behavior of real cell neurons and thus constrain the parameters of detailed neuronal models. As a result, there are now a large number of different models of many cell types available in the literature.

These published models were usually built to capture some important or interesting properties of the given neuron type, i.e., to reproduce the results of a few selected experiments, and it is often unknown, even by their developers, how they would behave in other situations, outside their original context. Nevertheless, for data-driven models to be predictive, it is important that they are able to generalize beyond their original scope. Furthermore, investigating and developing different hippocampal CA1 pyramidal cell models we experienced that tuning the model parameters so that the model reproduces a specific behaviour often significantly changes previously adjusted behaviours, which can easily remain unrecognized by the modeler. This limits the reusability of these models for different scientific purposes. Therefore, it would be important to test and evaluate the models under different conditions, to explore the changes in model behaviour when its parameters are tuned.

To make it easier for the modeling community to explore the changes in model behavior during parameter tuning, and to systematically compare models of rat hippocampal CA1 pyramidal cells that were developed using different methods and for different purposes, we have developed an automated Python test suite called HippoUnit. HippoUnit is based on the SciUnit framework [1] which was developed for the validation of scientific models against experimental data. The tests of HippoUnit automatically run simulations on CA1 pyramidal cell models built in the NEURON simulator [2] that mimic the electrophysiological protocol from which the target experimental data were derived. Then the behavior of the model is evaluated and quantitatively compared to the experimental data using various feature-based error functions. Current validation tests cover somatic behavior and signal propagation and integration in apical dendrites of rat hippocampal CA1 pyramidal single cell models. The package is open source, available on GitHub (https://github.com/KaliLab/hippounit) and it has been integrated into the Validation Framework developed within the Human Brain Project.

Here we present how we applied HippoUnit to test and compare the behavior of several different hippocampal CA1 pyramidal cell models available on ModelDB [4], against electrophysiological data available in the literature. By providing the software tools and examples on how to validate these models, we hope to encourage the modeling community to use more systematic testing during model development, in order to create neural models that generalize better, and make the process of model building more reproducible and transparent.

**References**

1. Omar C, Aldrich J, Gerkin RC. Collaborative infrastructure for test-driven scientific model validation. In *Companion Proceedings of the 36th International Conference on Software Engineering* 2014 May 31 (pp. 524–527). ACM.
2. Carnevale NT, Hines M. The NEURON Book. *Cambridge, UK: Cambridge University Press;* 2006.
3. Druckmann S, Banitt Y, Gidon AA, Schürmann F, Markram H, Segev I. A novel multiple objective optimization framework for constraining conductance-based neuron models by experimental data. *Frontiers in neuroscience* 2007 Oct 15;1:1.
4. McDougal RA, Morse TM, Carnevale T, et al. Twenty years of ModelDB and beyond: building essential modeling tools for the future of neuroscience. *Journal of computational neuroscience* 2017 Feb 1;42(1):1–0.
5. Appukuttan S, Garcia PE, Sharma BL, Sáray S, Káli S, Davison AP. Systematic Statistical Validation of Data-Driven Models in Neuroscience. *Program No. 524.04. 2018 Neuroscience Meeting Planner* San Diego, CA: Society for Neuroscience, 2018. Online.

## P9 Systematic integration of experimental data in biologically realistic models of the mouse primary visual cortex: Insights and predictions

### Yazan Billeh^1^, Binghuang Cai^2^, Sergey Gratiy^1^, Kael Dai^1^, Ramakrishnan Iyer^1^, Nathan Gouwens^1^, Reza Abbasi-Asl^2^, Xiaoxuan Jia^3^, Joshua Siegle^1^, Shawn Olsen^1^, Christof Koch^1^, Stefan Mihalas^1^, Anton Arkhipov^1^

#### ^1^Allen Institute for Brain Science, Modelling, Analysis and Theory, Seattle, WA, United States of America; ^2^Allen Institute for Brain Science, Seattle, WA, United States of America; ^3^Allen Institute for Brain Science, Neural Coding, Seattle, WA, United States of America

##### **Correspondence:** Yazan Billeh (yazanb@alleninstitute.org)

*BMC Neuroscience* 2019, **20(Suppl 1)**:P9

Data collection efforts in neuroscience are growing at an unprecedented pace, providing a constantly widening stream of highly complex information about circuit architectures and neural activity patterns. We leverage these data collection efforts to develop data-driven, biologically realistic models of the mouse primary visual cortex at two levels of granularity. The first model uses biophysically detailed neuron models with morphological reconstructions fit to experimental data. The second uses Generalized Leaky Integrate and Fire point neuron models fit to the same experimental recordings. Both models were developed using the Brain Modeling ToolKit (BMTK) and will be made freely available upon publication. We demonstrate how in the process of building these models, specific predictions about structure-function relationships in the mouse visual cortex emerge. We discuss three such predictions regarding connectivity between excitatory and non-parvalbumin expressing interneurons; functional specialization of connections between excitatory neurons; and the impact of the cortical retinotopic map on neuronal properties and connections.

## P10 Small-world networks enhance the inter-brain synchronization

### Kentaro Suzuki^1^, Jihoon Park^2^, Yuji Kawai^2^, Minoru Asada^2^

#### ^1^Osaka University, Graduate School of Engineering, Minoh City, Japan; ^2^Osaka University, Suita, Osaka, Japan

##### **Correspondence:** Kentaro Suzuki (kentaro.suzuki@ams.eng.osaka-u.ac.jp)

*BMC Neuroscience* 2019, **20(Suppl 1)**:P10

Many hyperscanning studies have shown that activities of the two brains often synchronize during social interaction (e.g., [1]). This synchronization occurs in various frequency bands and brain regions [1]. Further, Dumas et al. [2] constructed a two-brain model in which Kuramoto oscillators, as brain regions, are connected according to an anatomically realistic human connectome. They showed that the model with the realistic brain structure exhibits stronger inter-brain synchronization than the network with a randomly shuffled structure. However, it remains unclear what properties in the brain anatomical structure contribute to the inter-brain synchronization. Furthermore, since Kuramoto oscillators tend to converge to a specific frequency, the model cannot explain the synchronous activities in different frequency bands which were observed in the hyperscanning studies. In the current study, we propose a two-brain model based on small-world networks proposed by Watts and Strogatz method (WS method) [3] to systematically investigate the relationship between the small-world structure and the degree of inter-brain synchronization. WS method can control the clustering coefficient and shortest path length without changing the number of connections by rewiring probability p (p = 0.0: regular network, p = 0.1: small-world network, and p = 1.0: random network). We hypothesize that the small-world network, which has high clustering coefficient and low shortest path length, is responsible for the inter-brain synchronization owing to its efficient information transmission. The model consists of two networks, each of which network consists of 100 neuron groups composed by 1000 spiking neurons (800 excitatory and 200 inhibitory neurons). The neuron groups in a network are connected according to WS method. Some groups in the two networks are directly connected as inter-brain connectivity, which is in the same manner as the previous model [2]. We evaluated the inter-brain synchronization between neuron groups using Phase Locking Value (PLV). Fig. 1 shows PLVs in each combination of networks with different rewiring probabilities in the gamma band (31-48Hz). The mean PLV of the combination of small-world networks was higher than those of the other combinations.

**Fig. 1:**
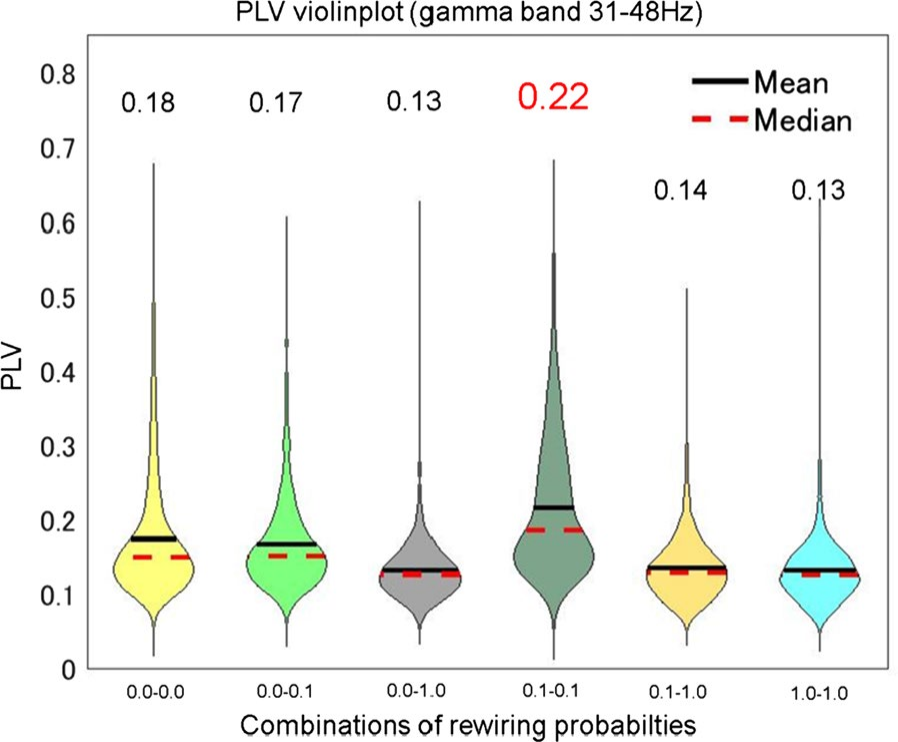
PLVs between the networks in gamma band (31–48Hz), where a higher value indicates stronger synchronization. X-axis indicates the combinations of values of rewiring probability p (p = 0.0: regular network, p = 0.1: small-world network, and p = 1.0: random network). Black lines and red broken lines indicate the mean and the median of the PLVs, respectively

The result implies that the small-world structure in the brains may be a key factor of the inter-brain synchronization. As a future direction, we plan to impose an interaction task on the current model instead of the direct connections to aim to understand the relationship between the social interaction and structure properties of the brains.

**Acknowledgments:** This work was supported by JST CREST Grant Number JPMJCR17A4, and a project commissioned by the New Energy and Industrial Technology Development Organization (NEDO).

**References**

1. Dumas G, Nadel J, Soussignan R, Martinerie J, Garnero L. Inter-brain synchronization during social interaction. *PloS one* 2010 Aug 17;5(8):e12166.
2. Dumas G, Chavez M, Nadel J, Martinerie J. Anatomical connectivity influences both intra-and inter-brain synchronizations. *PloS one* 2012 May 10;7(5):e36414.
3. Watts DJ, Strogatz SH. Collective dynamics of ‘small-world’ networks. *Nature* 1998 Jun;393(6684):440.

## P11 A potential mechanism for phase shifts in grid cells: leveraging place cell remapping to introduce grid shifts

### Zachary Sheldon^1^, Ronald DiTullio^2^, Vijay Balasubramanian^2^

#### ^1^University of Pennsylvania, Philadelphia, PA, United States of America; ^2^University of Pennsylvania, Computational Neuroscience Initiative, Philadelphia, United States of America

##### **Correspondence:** Zachary Sheldon (zsheldon@sas.upenn.edu)

*BMC Neuroscience* 2019, **20(Suppl 1)**:P11

Spatial navigation is a crucial part of survival, allowing an agent to effectively explore environments and obtain necessary resources. It has been theorized that this is achieved by learning an internal representation of space, known as a cognitive map. Multiple types of specialized neurons in the hippocampal formation and entorhinal cortex are believed to contribute to the formation of this cognitive map, particularly place cells and grid cells. These cells exhibit unique spatial firing fields that change in response to changes in environmental conditions. In particular, place cells display remapping of their spatial firing fields across different environments and grid cell display a phase shift in their spatial firing fields. If these cell types are indeed important for spatial navigation, we want to be able to explain the mechanism behind how the firing fields of these cell types change between environments. However, there are currently no suggested models or mechanisms for how this remapping and phase shift occur. Building off of previous work using continuous attractor network (CAN) models of grid cells, we propose a CAN model that incorporates place cell input to grid cells. By allowing for Hebbian learning between place cells and grid cells associated with two distinct environments, our model is able to replicate the phase shifts between environments observed in grid cells. Our model posits the first potential mechanism by which the cognitive map changes between environments, and will hopefully inspire new research into this phenomenon and spatial navigation as a whole.

## P12 Computational modeling of seizure spread on a cortical surface explains the theta-alpha electrographic pattern

### Viktor Sip^1^, Viktor Jirsa^1^, Maxime Guye^2^, Fabrice Bartolomei^3^

#### ^1^Aix-Marseille Universite, Institute de Neurosciences, Marseille, France; ^2^Aix-Marseille Université, Centre de Résonance Magnétique Biologique et Médicale, Marseille, France; ^3^Assistance Publique - Hôpitaux de Marseille, Service de Neurophysiologie Clinique, Marseille, France

##### **Correspondence:** Viktor Sip (viktor.sip@univ-amu.fr)

*BMC Neuroscience* 2019, **20(Suppl 1)**:P12

Intracranial electroencephalography is a standard tool in clinical evaluation of patients with focal epilepsy. Various early electrographic seizure patterns differing in frequency, amplitude, and waveform of the oscillations are observed in intracranial recordings. The pattern most common in the areas of seizure propagation is the so-called theta-alpha activity (TAA), whose defining features are oscillations in the theta-alpha range and gradually increasing amplitude. A deeper understanding of the mechanism underlying the generation of the TAA pattern is however lacking. We show by means of numerical simulation that the features of the TAA pattern observed on an implanted depth electrode in a specific epileptic patient can be plausibly explained by the seizure propagation across an individual folded cortical surface.

In order to demonstrate this, we employ following pipeline: First, the structural model of the brain is reconstructed from the T1-weighted images, and the position of the electrode contact are determined using the CT scan with implanted electrodes. Next, the patch of cortical surface in the vicinity of the electrode of interest is extracted. On this surface, the simulation of the seizure spread is performed using The Virtual Brain framework. As a mathematical model a field version of the Epileptor model is employed. The simulated source activity is then projected to the sensors using the dipole model, and this simulated stereo-electroencephalographic signal is compared with the recorded one.

The results show that the simulation on the patient-specific cortical surface gives a better fit between the recorded and simulated signals than the simulation on generic surrogate surfaces. Furthermore, the results indicate that the spectral content and dynamical features might differ in the source space of the cortical gray matter activity and among the intracranial sensors, questioning the previous approaches to classification of seizure onset patterns done in the sensor space, both based on spectral content and on dynamical features.

In conclusion, we demonstrate that the investigation of the seizure dynamics on the level of cortical surface can provide deeper insight into the large scale spatiotemporal organization of the seizure. At the same time, it highlights the need for a robust technique for inversion of the observed activity from sensor to source space that would take into account the complex geometry of the cortical sources and the position of the intracranial sensors.

**References**

1. Perucca P, Dubeau F, Gotman J. Intracranial electroencephalographic seizure-onset patterns: effect of underlying pathology. *Brain* 2014, 137, 183–196.
2. Sanz Leon P, Knock SA, Woodman MM, et al. The Virtual Brain: a simulator of primate brain network dynamics. *Frontiers in Neuroinformatics* 2013, Jun 11;7:10.
3. Jirsa V, Stacey W, Quilichini P, Ivanov A, Bernard C. On the nature of seizure dynamics. *Brain* 2014, 137, 2110–2113.
4. Proix T, Jirsa VK, Bartolomei F, Guye M, Truccolo W. Predicting the spatiotemporal diversity of seizure propagation and termination in human focal epilepsy. *Nature Communications* 2018, Mar 14;9(1):1088.

## P13 Bistable firing patterns: one way to understand how epileptic seizures are triggered

### Fernando Borges^1^, Paulo Protachevicz^2^, Ewandson Luiz Lameu^3^, Kelly Cristiane Iarosz^4^, Iberê Caldas^4^, Alexandre Kihara^1^, Antonio Marcos Batista^5^

#### ^1^Federal University of ABC, Center for Mathematics, Computation, and Cognition., São Bernardo do Campo, Brazil; ^2^State University of Ponta Grossa, Graduate in Science Program, Ponta Grossa, Brazil; ^3^National Institute for Space Research (INPE), LAC, São José dos Campos, Brazil; ^4^University of São Paulo, Institute of Physics, São Paulo, Brazil; ^5^State University of Ponta Grossa, Program of Post-graduation in Science, Ponta Grossa, Brazil

##### **Correspondence:** Fernando Borges (fernandodasilvaborges@gmail.com)

*BMC Neuroscience* 2019, **20(Suppl 1)**:P13

Excessively high, neural synchronisation has been associated with epileptic seizures, one of the most common brain diseases worldwide. Previous researchers have argued which epileptic and normal neuronal activity are support by the same physiological structure. However, to understand how neuronal systems transit between these regimes is a wide question to be answered. In this work, we study neuronal synchronisation in a random network where nodes are neurons with excitatory and inhibitory synapses, and neural activity for each node is provided by the adaptive exponential integrate-and-fire model. In this framework, we verify that the decrease in the influence of inhibition can generate synchronisation originating from a pattern of desynchronised spikes. The transition from desynchronous spikes to synchronous bursts of activity, induced by varying the synaptic coupling, emerges in a hysteresis loop due to bistability where abnormal (excessively high synchronous) regimes exist. We verify that, for parameters in the bistability regime, a square current pulse can trigger excessively high (abnormal) synchronisation, a process that can reproduce features of epileptic seizures. Then, we show that it is possible to suppress such abnormal synchronisation by applying a small-amplitude external current on less than 10% of the neurons in the network. Our results demonstrate that external electrical stimulation not only can trigger synchronous behaviour, but more importantly, it can be used as a means to reduce abnormal synchronisation and thus, control or treat effectively epileptic seizures.

## P14 Can sleep protect memories from catastrophic forgetting?

### Oscar Gonzalez^1^, Yury Sokolov^2^, Giri Krishnan^2^, Maxim Bazhenov^2^

#### ^1^University of California, San Diego, Neurosciences, La Jolla, CA, United States of America; ^2^University of California, San Diego, Medicine, La Jolla, United States of America

##### **Correspondence:** Oscar Gonzalez (o2gonzalez@ucsd.edu)

*BMC Neuroscience* 2019, **20(Suppl 1)**:P14

Previously encoded memories can be damaged by encoding of new memories, especially when they are relevant to the new data and hence can be disrupted by new training—a phenomenon called “catastrophic forgetting”. Human and animal brains are capable of continual learning, allowing them to learn from past experience and to integrate newly acquired information with previously stored memories. A range of empirical data suggest important role of sleep in consolidation of recent memories and protection of the past knowledge from catastrophic forgetting. To explore potential mechanisms of how sleep can enable continual learning in neuronal networks, we developed a biophysically-realistic thalamocortical network model where we could train multiple memories with different degree of interference. We found that in a wake-like state of the model, training of a “new” memory that overlaps with previously stored “old” memory results in degradation of the old memory. Simulating NREM sleep state immediately after new learning led to replay of both old and new memories—this protected old memory from forgetting and ultimately enhanced both memories. The effect of sleep was similar to the interleaved training of the old and new memories. The study revealed that the network slow-wave oscillatory activity during simulated deep sleep leads to a complex reorganization of the synaptic connectivity matrix that maximizes separation between groups of synapses responsible for conflicting memories in the overlapping population of neurons. The study predicts that sleep may play a protective role against catastrophic forgetting and enables brain networks to undergo continual learning.

## P15 Predicting the distribution of ion-channels in single neurons using compartmental models.

### Roy Ben-Shalom^1^, Kyung Geun Kim^2^, Matthew Sit^3^, Henry Kyoung^3^, David Mao^3^, Kevin Bender^1^

#### ^1^University of California, San-Francisco, Neurology, San-Francisco, CA, United States of America; ^2^University of California, Berkeley, EE/CS, Berkeley, CA, United States of America; ^3^University of California, Berkeley, Computer Science, Berkeley, United States of America

##### **Correspondence:** Roy Ben-Shalom (bens.roy@gmail.com)

*BMC Neuroscience* 2019, **20(Suppl 1)**:P15

Neuronal activity arises from the concerted activity of different ionic currents that are distributed in varying densities across different neuronal compartments, including the axon, soma, and dendrite. One major challenge in understanding neuronal excitability remains understanding precisely how different ionic currents are distributed in neurons. Biophysically detailed neuronal compartmental models allow us to distribute the channels along the morphology of a neuron and simulate resultant voltage responses. One can then use optimization algorithms that fit model’s responses to the neuronal recordings to predict the channels distributions for the model. The quality of predictions generated from such models depends critically on the biophysical accuracy of the model. Depending on how optimization is implemented—both mathematically and experimentally—one can arrive at several solutions that all reasonably fit empirical datasets. However, to generate predictions that can be validated in experiments we need to reach a unique solution that predicts the neuronal activity for a rich repertoire of experimental conditions. As we increase the size of an empirical dataset, the number of model solutions that can accurately account for these empirical observations decreases, theoretically arriving at one unique solution. Here we present a novel approach designed to identify this unique solution in a multi-compartmental model by fitting models to data obtained from a somatic neuronal recording and post-hoc morphological reconstruction. To validate this approach, we began by reverse engineering a classic model of a neocortical pyramidal cell developed by [1], which contains 12 free parameters describing ion channels distributed across dendritic, somatic, and axonal compartments. First, we used the original values of these free parameters (e.g., the target data) to create a dataset of voltage responses that represents a ground truth. Given this target dataset, our goal was to determine whether we could use optimization to arrive at similar parameter values when these values were unknown. We tested over 350 different stimulation protocols and 15 score functions, which compare the simulated data to the ground truth dataset, to determine which combination of stimulation and score functions creates datasets that reliably constrain the model. Then we checked how sensitive each parameter was to different score functions. We found that five of the twelve parameters were sensitive to many different score functions. While these five could be constrained, the other seven parameters were sensitive only to a small set of score functions. We therefore divided the remaining optimization process to several steps, iteratively constraining a subset of the parameters that were sensitive to the same stimulation protocols and score functions. With this approach, were able to constrain 11/12 of the parameters of the model and recover the original values. This suggests that iterative, sensitivity analysis-based optimization could allow for more accurate fitting of model parameters to empirical data. We are currently testing whether similar methods can be used on more recently developed models with more free parameters. Ultimately, our goal is to apply this method to empirical recordings of neurons in acute slice and *in vivo* conditions.

**Reference**

1. Mainen ZF, Sejnowski TJ. Influence of dendritic structure on firing pattern in model neocortical neurons. *Nature* 1996 Jul;382(6589):363.

## P16 The contribution of dendritic spines to synaptic integration and plasticity in hippocampal pyramidal neurons

### Luca Tar^1^, Sára Sáray^2^, Tamas Freund^1^, Szabolcs Kali^1^, Zsuzsanna Bengery^2^

#### ^1^Institute of Experimental Medicine, Hungarian Academy of Sciences, Budapest, Hungary; ^2^Faculty of Information Technology and Bionics, Pázmány Péter Catholic University, Hungary

##### **Correspondence:** Luca Tar (luca.tar04@gmail.com)

*BMC Neuroscience* 2019, **20(Suppl 1)**:P16

The dendrites of cortical pyramidal cells bear spines which receive most of the excitatory synaptic input, act as separate electrical and biochemical compartments, and play important roles in signal integration and plasticity. In this study, we aimed to develop fully active models of hippocampal pyramidal neurons including spines to analyze the contributions of nonlinear processes in spines and dendrites to signal integration and synaptic plasticity. We also investigated ways to reduce the computational complexity of models of spiny neurons without altering their functional properties.

As a first step, we built anatomically and biophysically detailed models of CA1 pyramidal neurons without explicitly including dendritic spines. The models took into account multiple attributes of the cell determined by experiments, including the biophysics and distribution of ion channels, as well as the different electrophysiological characteristics of the soma and the dendrites. For systematic model development, we used two software tools developed in our lab: Optimizer [2] for automated parameter fitting, and the HippoUnit package, based on SciUnit [3] modules, to validate these results. We gradually increased the complexity of our model, mainly by adding further types of ion channels, and monitored the ability of the model to capture both optimized and non-optimized features and behaviors. This method allowed us to determine the minimal set of mechanisms required to replicate particular neuronal behaviors and resulted in a new model of CA1 pyramidal neurons whose characteristics match a wide range of experimental results.

Next, starting from a model which matched the available data on nonlinear dendritic integration [5], we added dendritic spines and moved excitatory synapses to the spine head. Simply adding the spines to the original model significantly changed the propagation of signals in dendrites, the properties of dendritic spikes and the overall characteristics of synaptic integration. This was due mainly to the effective change in membrane capacitance and the density of voltage-gated and leak conductances, and could be compensated by appropriate changes in these parameters. The resulting model showed the correct behavior for nonlinear dendritic integration while explicitly implementing all dendritic spines.

As the effects of spines on dendritic spikes and signal propagation could be largely explained by their effect on the membrane capacitance and conductance, we also developed a simplified version of the model where only those dendritic spines which received synaptic input were explicitly modeled, while the rest of the spines were implicitly taken into account by appropriate changes in the membrane properties. This model behaved very similarly to the one where all spines were explicitly modeled, but ran significantly faster. Our approach generalizes the F-factor method of [4] to active models.

Finally, our models which show realistic electrical behavior in their dendrites and spines allow us to examine Ca dynamics in dendritic spines in response to any combination of synaptic inputs and somatic action potentials. In combination with models of the critical molecular signaling pathways [1], this approach enables a comprehensive computational investigation of the mechanisms underlying activity-dependent synaptic plasticity in hippocampal pyramidal neurons.

**References**

1. Lindroos R, Dorst MC, Du K, et al. Basal Ganglia Neuromodulation Over Multiple Temporal and Structural Scales—Simulations of Direct Pathway MSNs Investigate the Fast Onset of Dopaminergic Effects and Predict the Role of Kv4. 2. *Frontiers in neural circuits* 2018 Feb 6;12:3.
2. Friedrich P, Vella M, Gulyás AI, Freund TF, Káli S. A flexible, interactive software tool for fitting the parameters of neuronal models. *Frontiers in neuroinformatics* 2014 Jul 10;8:63.
3. Omar C, Aldrich J, Gerkin RC. Collaborative infrastructure for test-driven scientific model validation. In *Companion Proceedings of the 36th International Conference on Software Engineering* 2014 May 31 (pp. 524–527). ACM.
4. Rapp M, Yarom Y, Segev I. The impact of parallel fiber background activity on the cable properties of cerebellar Purkinje cells. *Neural Computation* 1992 Jul;4(4):518–33.
5. Losonczy A, Magee JC. Integrative properties of radial oblique dendrites in hippocampal CA1 pyramidal neurons. *Neuron* 2006 Apr 20;50(2):291–307.

## P17 Modelling the dynamics of optogenetic stimulation at the whole-brain level

### Giovanni Rabuffo^1^, Viktor Jirsa^1^, Francesca Melozzi^1^, Christophe Bernard^1^

#### ^1^Aix-Marseille Université, Institut de Neurosciences des Systèmes, Marseille, France

##### **Correspondence:** Giovanni Rabuffo (giovanni.rabuffo@univ-amu.fr)

*BMC Neuroscience* 2019, **20(Suppl 1)**:P17

Deep brain stimulation is commonly used in different pathological conditions, such as Parkinson’s disease, epilepsy, and depression. However, there is scant knowledge regarding the way of stimulating the brain to cause a predictable and beneficial effect. In particular, the choice of the area to stimulate and the stimulation settings (amplitude, frequency, duration) remain empirical [1].

To approach these questions in a theoretical framework, an understanding of how stimulation propagates and influences the *global brain dynamics* is of primary importance.

A precise stimulation (activation/inactivation) of specific cell-types in brain regions of interest can be obtained using *optogenetic methods*. Such stimulation will act in a short-range domain i.e., local in the brain region, as well as on a large-scale network. Both these effects are important to understand the final outcome of the stimulation [2]. Therefore, a *whole brain* approach is required.

In our work we use *The Virtual Brain* platform to model an optogenetic stimulus and to study its global effects on a “virtual” mouse brain [3]. The parameters of our model can be gauged in order to account for the intensity of the stimulus, which is generally controllable during experimental tests.

The functional activity of the mouse brain model can be compared to experimental evidences coming from in vivo *optogenetic fMRI* (ofMRI) [4]. In silico exploration of the parameter space allows then to fit the results of an ofMRI dataset as well as to make predictions on the outcome of a stimulus depending not only by its anatomical location and cell-type, but also by the connection topology.

The theoretical study of the network dynamics emerging from such adjustable and traceable stimuli, provides a step forward in the understanding of the causal relation between structural and functional connectomes.

**References**

1. Sironi VA. Origin and evolution of deep brain stimulation. *Frontiers in integrative neuroscience* 2011 Aug 18;5:42.
2. Fox MD, Buckner RL, Liu H, Chakravarty MM, Lozano AM, Pascual-Leone A. Resting-state networks link invasive and noninvasive brain stimulation across diverse psychiatric and neurological diseases. *Proceedings of the National Academy of Sciences* 2014 Oct 14;111(41):E4367–75.
3. Melozzi F, Woodman MM, Jirsa VK, Bernard C. The virtual mouse brain: a computational neuroinformatics platform to study whole mouse brain dynamics. *eNeuro* 2017 May;4(3).
4. Lee JH, Durand R, Gradinaru V, et al. Global and local fMRI signals driven by neurons defined optogenetically by type and wiring. *Nature* 2010 Jun;465(7299):788.

## P18 Investigating the effect of the nanoscale architecture of astrocytic processes on the propagation of calcium signals

### Audrey Denizot^1^, Misa Arizono^2^, Weiliang Chen^3^, Iain Hepburn^3^, Hédi Soula^4^, U. Valentin Nägerl^2^, Erik De Schutter^3^, Hugues Berry^5^

#### ^1^INSA Lyon, Villeurbanne, France; ^2^Université de Bordeaux, Interdisciplinary Institute for Neuroscience, Bordeaux, France; ^3^Okinawa Institute of Science and Technology, Computational Neuroscience Unit, Onna-Son, Japan; ^4^University of Pierre and Marie Curie, INSERM UMRS 1138, Paris, France; ^5^INRIA, Lyon, France

##### **Correspondence:** Audrey Denizot (audrey.denizot@inria.fr)

*BMC Neuroscience* 2019, **20(Suppl 1)**:P18

According to the concept of the ‘tripartite synapse’ [1], information processing in the brain results from dynamic communication between pre- and post- synaptic neurons and astrocytes. Astrocyte excitability results from transients of cytosolic calcium concentration. Local calcium signals are observed both spontaneously and in response to neuronal activity within fine astrocyte ramifications [2, 3], that are in close contact with synapses [4]. Those fine processes, that belong to the so-called spongiform structure of astrocytes, are too fine to be resolved spatially with conventional light microscopy [5, 6]. However, calcium dynamics in these structures can be investigated by computational modeling. In this study, we investigate the roles of the spatial properties of astrocytic processes on their calcium dynamics. Because of the low volumes and low number of molecules at stake, we use our stochastic spatially-explicit individual-based model of astrocytic calcium signals in 3D [7], implemented with STEPS [8]. We validate our model by reproducing key parameters of calcium signals that we have recorded with high-resolution calcium imaging in organotypic brain slices. Our simulations reveal the importance of the spatial organization of the implicated molecular actors for calcium dynamics. Particularly, we predict that different spatial organizations can lead to very different types of calcium signals, even for two processes displaying the exact same calcium channels, with the same densities. We also investigate the impact of process geometry at the nanoscale on calcium signal propagation. By modeling realistic astrocyte geometry at the nanoscale, this study thus proposes plausible mechanisms for information processing within astrocytes as well as neuron-astrocyte communication.

**References**

1. Araque A, Parpura V, Sanzgiri RP, Haydon PG. Tripartite synapses: glia, the unacknowledged partner. *Trends in neurosciences* 1999 May 1;22(5):208–15.
2. Arizono M, et al. Structural Basis of Astrocytic Ca2+ Signals at Tripartite Synapses. *Social Science Research Network* 2018.
3. Bindocci E, Savtchouk I, Liaudet N, Becker D, Carriero G, Volterra A. Three-dimensional Ca2+ imaging advances understanding of astrocyte biology. *Science* 2017 May 19;356(6339):eaai8185.
4. Ventura R, Harris KM. Three-Dimensional Relationships between Hippocampal Synapses and Astrocytes. *Journal of Neuroscience* 1999, Aug 15;19(16): 6897–6906.
5. Heller JP, Rusakov DA. The nanoworld of the tripartite synapse: insights from super-resolution microscopy. *Frontiers in Cellular Neuroscience* 2017 Nov 24;11:374.
6. Panatier A, Arizono M, Nägerl UV. Dissecting tripartite synapses with STED microscopy. *Phil Trans of the Royal Society B: Biological Sciences* 2014 Oct 19;369(1654):20130597.
7. Audrey D, Misa A, Valentin NU, Hédi S, Hugues B. Simulation of calcium signaling in fine astrocytic processes: effect of spatial properties on spontaneous activity. *bioRxiv* 2019 Jan 1:567388.
8. Hepburn I, Chen W, Wils S, De Schutter E. STEPS: efficient simulation of stochastic reaction–diffusion models in realistic morphologies. *BMC systems biology* 2012 Dec;6(1):36.

## P19 Neural mass modeling of the Ponto-Geniculo-Occipital wave and its neuromodulation

### Kaidi Shao^1^, Nikos Logothetis^1^, Michel Besserve^1^

#### ^1^MPI for Biological Cybernetics, Department for Physiology of Cognitive Processes, Tübingen, Germany

##### **Correspondence:** Kaidi Shao (kdshao@gmail.com)

*BMC Neuroscience* 2019, **20(Suppl 1)**:P19

As a prominent feature of Rapid Eye Movement (REM) sleep and the transitional stage from Slow Wave Sleep to REM sleep (the pre-REM stage), Ponto-Geniculo-Occipital (PGO) waves are hypothesized to play a critical role in dreaming and memory consolidation [1]. During pre-REM and REM stages, PGO waves appear in two subtypes differing in number, amplitude and frequency. However, the mechanisms underlying their generation and propagation across multiple brain structures, as well as their functions, remains largely unexplored. In particular, contrary to the multiple phasic events occurring during non-REM sleep (slow waves, spindles and sharp-wave ripples), computational modeling of PGO waves has to the best of our knowledge not yet been investigated.

Based on experimental evidence in cats, the species were most extensively studied, we elaborated an existing thalamocortical model operating in the pre-REM stage [2], and constructed a ponto-thalamo-cortical neural mass model consisting of 6 rate-coded neuronal populations interconnected via biologically-verified synapses (Fig. 1A). Transient PGO-related activities are elicited by a single or multiple brief pulses, modelling the input bursts that PGO-triggering neurons send to cholinergic neurons in the pedunculopontine tegmentum nucleus (PPT). The effect of acetylcholine (ACh), as the primarily-affecting neuromodulator during the SWS-to-REM transition, was also modelled by tuning several critical parameters with tonically-varying ACh concentration.

**Fig. 1:**
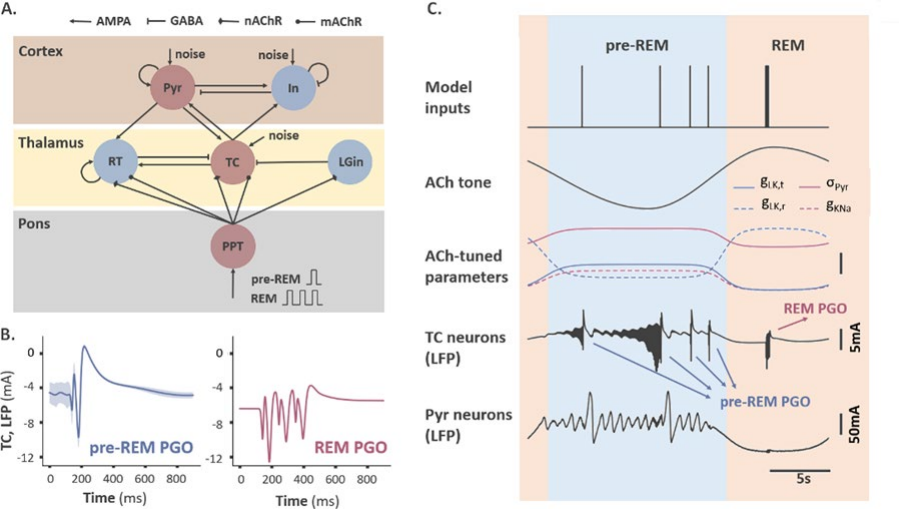
**a** Model structure. TC: thalamocortical neurons. RT: reticular thalamic neurons. Pyr: pyramidal neurons. In: inhibitory neurons. LGin: thalamic interneurons. PPT: PGO-transferring neurons. **b** Typical waveforms of two subtypes of thalamic PGO waves. **c** Example traces of thalamic and cortical LFPs modulated by a cholinergic tone. Unscaled bar: 2 mV for red, 2 mS for dashed red, and 0.01 mS for others

Our simulations are able to reproduce deflections in local field potentials (LFPs), as well as other electrophysiological characteristics consistent in many respects with classical electrophysiological studies (Fig. 1B). For example, the duration of both subtypes of thalamic PGO waves matches that of the PGO recordings with a similar waveform comprised of a sharp negative peak and a slower positive peak. The bursting duration of TC and RT neurons (10ms, 25ms) falls in the range reported by experimental papers (7-15ms, 20-40ms). Consistent with experimental findings, the simulated PGO waves block spindle oscillations that occur during pre-REM stage. By incorporating tonic cholinergic neuromodulation to mimic the SWS-to-REM transition, we were also able to replicate the electrophysiological differences between the two PGO subtypes with an ACh-tuned leaky potassium conductance in TC and RT neurons (Fig. 1C).

These results help clarify the cellular mechanisms underlying thalamic PGO wave generation, e.g., the nicotinic depolarization of LGin neurons, whose role used to be under debate, is shown to be critical for the generation of the negative peak. The model elucidates how ACh modulates state transitions throughout the wake-sleep cycle, and how this modulation leads to a recently-reported difference of transient change in the thalamic multi-unit activities. The simulated PGO waves also provides us a biologically-plausible framework to investigate how they take part in the multifaceted brain-wide network phenomena occurring during sleep and the enduring effects they may induce through plasticity.

**References**

1. Gott JA, Liley DT, Hobson JA. Towards a functional understanding of PGO waves. *Frontiers in human Neuroscience* 2017 Mar 3;11:89.
2. Costa MS, Weigenand A, Ngo HV, et al. A thalamocortical neural mass model of the EEG during NREM Sleep and its response to auditory stimulation. *PLoS computational biology* 2016 Sep 1;12(9):e1005022.

## P20 Oscillations in working memory and neural binding: a mechanism for multiple memories and their interactions

### Jason Pina^1^, G. Bard Ermentrout^2^, Mark Bodner^3^

#### ^1^York University, Physicsand Astronomy, Toronto, Canada; ^2^University of Pittsburgh, Department of Mathematics, Pittsburgh, PA, United States of America; ^3^Mind Research Institute, Irvine, United States of America

##### **Correspondence:** Jason Pina (jay.e.pina@gmail.com)

*BMC Neuroscience* 2019, **20(Suppl 1)**:P20

Working memory is a form of short term memory that seems to be limited in capacity to 3–5 items. It is well known that neurons increase their firing rates from a low baseline state while information is being retained during working memory tasks. However, there is evidence of oscillatory firing rates in the active states, both in individual and in aggregate (for example, LFP and EEG) dynamics. Additionally, each memory may be composed of several different items, such as shape, color, and location. The neural correlate of the association of several items, or neural binding, is not well understood, but may be the synchronous firing of populations of neurons. Thus, the phase information of such oscillatory ensemble activity is a natural candidate to distinguish between bound (synchronous oscillations) and distinct (out-of-phase oscillations) items held actively in working memory.

Here, we explore a population firing rate model that exhibits bistability between a low baseline firing rate and a high, oscillatory firing rate. Coupling several of these populations together to form a firing rate network allows for competitive oscillatory dynamics, whereby different populations may be pairwise synchronous or out-of-phase, corresponding to bound or distinct items in memory, respectively. We find that up to 3 populations may oscillate out-of-phase with plausible modelconnectivitiesand parameter values, a result that is consistent with working memory capacity. The formulation of the model allows us to better examine from a dynamical systems perspective how these states arise as bifurcations of steady states and periodic orbits. In particular, we look at the ranges of coupling strengths and synaptic time scales that allow for synchronous and out-of-phase attracting states. We also explore how varying patterns of selective stimuli can produce and switch between rich sets of dynamics that may be relevant to working memory states and their transitions.

## P21 DeNSE: modeling neuronal morphology and network structure in silico

### Tanguy Fardet^1^, Alessio Quaresima^2^, Samuel Bottani^2^

#### ^1^University of Tübingen, Computer Science Department - Max Planck Institute for Biological Cybernetics, Tübingen, Germany; ^2^Université Paris Diderot, Laboratoire Matière et Systèmes Complexes, Paris, France

##### **Correspondence:** Tanguy Fardet (tanguy.fardet@tuebingen.mpg.de)

*BMC Neuroscience* 2019, **20(Suppl 1)**:P21

Neural systems develop and self-organize into complex networks which can generate stimulus-specific responses. Neurons grow into various morphologies, which influences their activity and the structure of the resulting network. Different network topologies can then display very different behaviors, which suggests that neuronal structure and network connectivity strongly influence the set of functions that can be sustained by a set of neurons. To investigate this, I developed a new simulation platform, DeNSE, aimed at studying the morphogenesis of neurons and networks, and enabling to test how interactions between neurons and their surroundings can shape the emergence of specific properties.

The goal of this new simulator is to serve as a general framework to study the dynamics of neuronal morphogenesis, providing predictive tools to investigate how neuronal structures emerge in complex spatial environments. The software generalizes models present in previous simulators [1, 2], gives access to new mechanisms, and accounts for spatial constraints and neuron-neuron interactions. It has been primarily applied on two main lines of research: a) neuronal cultures or devices, their structures being still poorly defined and strongly influenced by interactions or spatial constraints [3], b) morphological determinants of neuronal disorders, analyzing how changes at the cellular scale affect the properties of the whole network [4].

I illustrate how DeNSE enables to investigate neuronal morphology at different scales, from single cell to network level, notably through cell-cell and cell-surroundings interactions (Fig. 1). At the cellular level, I show how branching mechanisms affect neuronal morphology, introducing new models to account for interstitial branching and the influence of the environment. At intermediate levels, I show how DeNSE can reproduce interactions between neurites and how these contribute to the final morphology and introduce correlations in the network structure. At the network level, I stress how networks obtained through a growth process differ from both simple generative models and more complex network models where the connectivity comes from overlaps of real cell morphologies. Eventually, I demonstrate how DeNSE can provide biologically relevant structures to study spatio-temporal activity patterns in neuronal cultures and devices. In these structures, where the morphologies of the neurons and the network are not well defined but have been shown to play a significant role, DeNSE successfully reproduces experimental setups, predicts the influence of spatial constraints, and enables to predict their electrical activities. Such a tool can therefore be extremely useful to test structures and hypotheses prior to actual experiments, thus saving time and resources.

**Fig. 1:**
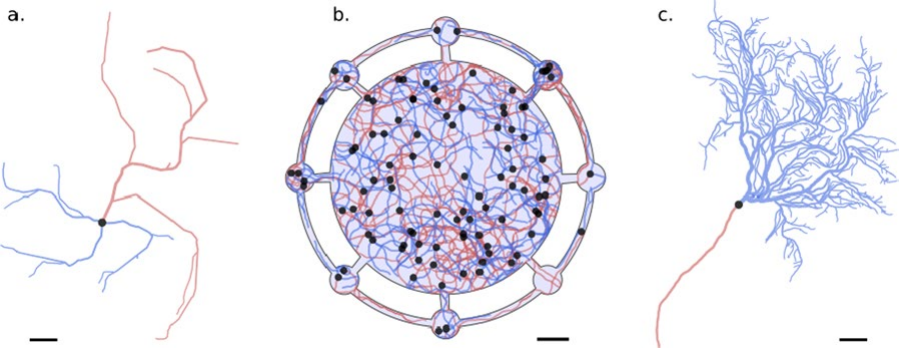
Structures generated with DeNSE; axons are in red, dendrites in blue, and cell bodies in black; scale bars are 50 microns. **a** Multipolar cell. **b** Neuronal growth in a structured neuronal device (light blue background) with a central chamber and small peripheric chambers; interactions between neurites can be seen notably through the presence of some fasciculated axon bundles. **c** Purkinje cell

**References**

1. Koene, R et al. NETMORPH: a framework for the stochastic generation of large-scale neuronal networks with realistic neuron morphologies. *Neuroinformatics* 2009, 7(3), 195–210
2. Torben-Nielsen, B et al. Context-aware modeling of neuronal morphologies. *Frontiers in Neuroanatomy* 2014, 8(92)
3. Renault, R et al. Asymmetric axonal edge guidance: a new paradigm for building oriented neuronal networks. *Lab Chip* 2016, 16(12), 2188–2191
4. Milatovic D et al. Morphometric Analysis in Neurodegenerative Disorder. *Current Protocols in Toxicology* 2010, Feb 1;43(1):12–6.

## P22 Sponge astrocyte model: volume effects in a 2D model space simplification

### Darya Verveyko^1^, Andrey Verisokin^1^, Dmitry Postnov^2^, Alexey R. Brazhe^3^

#### ^1^Kursk State University, Department of Theoretical Physics, Kursk, Russia; ^2^Saratov State University, Institute for Physics, Saratov, Russia; ^3^Lomonosov Moscow State University, Department of Biophysics, Moscow, Russia

##### **Correspondence:** Darya Verveyko (allegroform@mail.ru)

*BMC Neuroscience* 2019, **20(Suppl 1)**:P22

Calcium signaling in astrocytes is crucial for the nervous system function. Earlier we proposed a 2Dastrocytemodelof calcium waves dynamics [1], where waves were driven by local stochastic surges of glutamate which simulated the synaptic activity. The main idea of the model was in reproducing the spatially segregated mechanisms, belonging to regions with different dynamics: (i) the core with calcium exchange mainly with endoplasmic reticulum (ER) and (ii) peripheral compartment with currents through a plasma membrane (PM) with dominating Ca dynamics.

Real astrocytes are obviously not binary. There is a graded transition from thick branches to branchlets and to leaflets, primarily determined via the surface-to-volume ratio (SVR). Moreover, leaflet regions of the template contain not only astrocyte itself, but also the neuropil. We encode the astrocyte structural features by means of its color representation. Let the black color corresponds to astrocyte-free region, and the blue channel color indicate the presence of an astrocyte. Instead of binary leaflet-branch segregation, we introduce the astrocyte volume fraction (AVF) parameter, which indicates how much of the 2D cell volume is occupied by the astrocyte in real 3D effigy (the rest part is neuropil). AVF is encoded by the red channel intensity (Fig. 1A). The soma and thick branches region contain only the astrocyte (AVF = 1). The non-astro content increases from the soma to edges of an astrocyte through the leaflets, so AVF parameter should decrease and the red channel tends to its minimum value equal to 0.1 on the astrocyte border. To describe the relative effect of the exchange through PM and ER, we introduce the SVR parameter, which depends on AVF as a reverse sigmoid form. The SVR value is maximal at the edges of the leaflets and minimal in the soma.

**Fig. 1:**
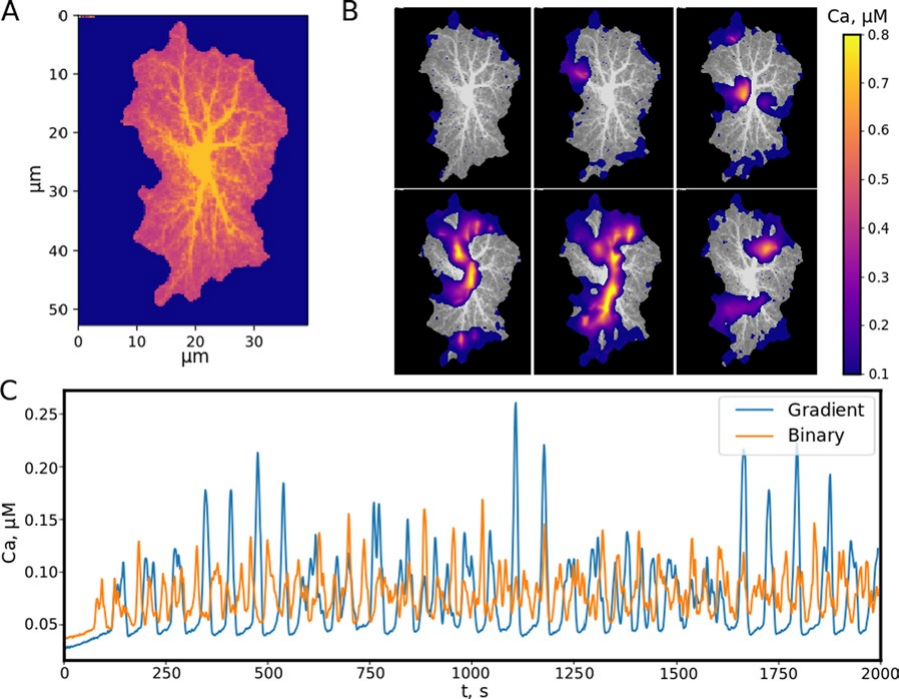
**a** AVR representation of 2D image template obtained as maximum intensity projection of experimentally 3D astrocyte image, numbers from 1 to 6 indicate regions of interest (ROI). **b** Calcium waves in a local astrocyte. **c** The average calcium concentration in the model with binary geometry (red line) and in the proposed model (blue line)

The implementation of AVF and SVR effects is based on the following reasoning: larger AVF (correspondingly, smaller SVR) reflects Ca dynamics dominated by ER exchange (IP3R-mediated) and less input from PM mechanisms (IP3 synthesis and PM-mediated Ca currents). Larger SVR in turn reflects underlying tortuosity of the astrocyte cytoplasm volume by attenuating apparent diffusion coefficients for IP3 and Ca. Finally, small concentration changes in areas with high AVF will cause larger changes in concentration in the neighboring areas with low AVF due to unequal volumes taken up by astrocytic cytoplasm.

Simulations of the proposed model show the formation of calcium waves (Fig. 1B), which propagate throughout the astrocyte template from the borders towards the center. In contrast to the previous binary segmentation model, calcium elevation response in the proposed biophysically more realistic sponge model is greater, i.e. the intensity of the formed waves is higher, but the basal calcium level is lower (Fig. 1C). At the same time, the threshold of stable wave existence grows because increasing AVF works like a blocking barrier for a small glutamate release reducing the number of wave sources. Nevertheless, large enough glutamate release leads to a wide-area wave quickly occupying the leaflets moving to the astrocyte soma.

**Acknowledgements:** This work is supported by the RFBR grant 17-00-00407.

**Reference**

1. Verveyko DV, et al. Raindrops of synaptic noise on dual excitability landscape: an approach to astrocyte network modelling. *Proceedings SPIE* 2018, 10717, 107171S.

## P23 Sodium-calcium exchangers modulate excitability of spatially distributed astrocyte networks

### Andrey Verisokin^1^, Darya Verveyko^1^, Dmitry Postnov^2^, Alexey R. Brazhe^3^

#### ^1^Kursk State University, Department of Theoretical Physics, Kursk, Russia; ^2^Saratov State University, Institute for Physics, Saratov, Russia; ^3^Lomonosov Moscow State University, Department of Biophysics, Moscow, Russia

##### **Correspondence:** Andrey Verisokin (ffalconn@mail.ru)

*BMC Neuroscience* 2019, **20(Suppl 1)**:P23

Previously we proposed two models of astrocytic calcium dynamics modulated by local synaptic activity. The first one [1] is based on the inositol trisphosphate-dependent exchange with the intracellular calcium storage taking into account specific topological features, namely different properties of thick branches and soma with thin branches. The second local model for a separate segment of an astrocyte [2] considers the sodium-calcium exchanger (NCX) and Na+ response to the synaptic glutamate. In this work we combine these two models and proceed to a spatially distributed astrocyte network. Our main goal is to study the process of the cytoplasmic calcium wave initiation and its motion through the astrocyte network.

The astrocyte cell is represented in the model by a 2D projection of a real cell microphotograph and indicated by a blue colour. The intensity of a red channel in each pixel shows the cytoplasm/ neuropil volume ratio. We introduce this volume characteristic to describe the differences in diffusion rates and the contribution of ion currents through endoplasmic reticulum membrane and plasma membrane in soma, branches and leaflets. We further connect various astrocyte cell templates into a network (Fig. 1A).

**Fig. 1:**
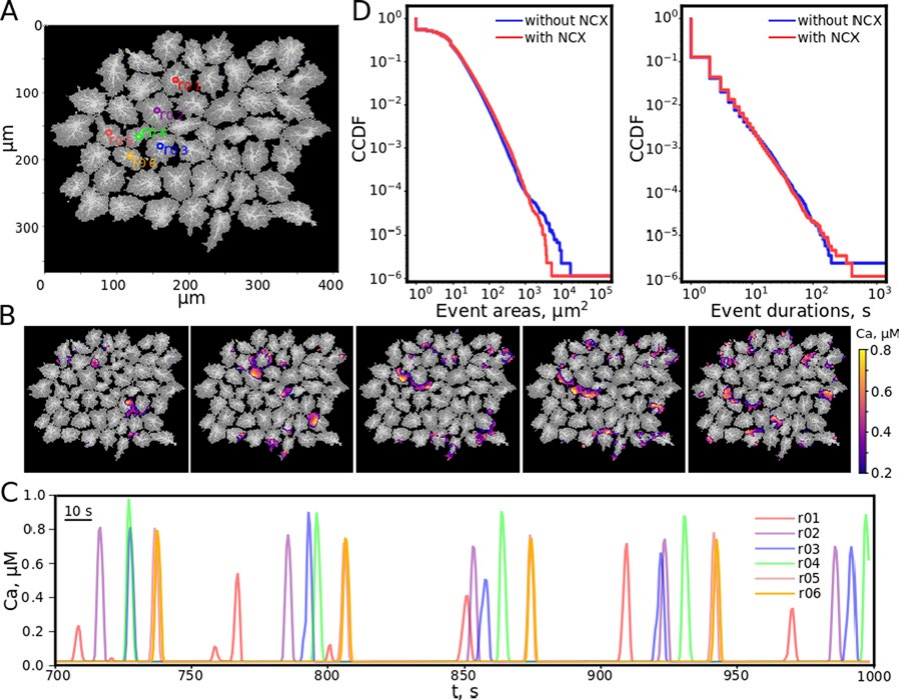
**a** Astrocyte network simulation template, the numbered circles indicate some regions of interest (ROI). **b** The example of spreading calcium wave. **c** Calcium dynamics in ROIs illustrates the quasi-pacemaker behavior. **d** CCDF for areas and durations of calcium excitation for the models with and without (blue and red lines correspondingly) NCX regulation

The proposed mathematical model includes 7 variables: calcium concentrations in cytosol andendoplasmic reticulum, inositol trisphosphate and sodium concentrations incytosol, extracellular glutamate concentration, inositol trisphosphatereceptor and NCX inactivation gating variables *h* and *g*. Synaptic glutamate activity is described by a quantal release triggered by a spike train drawn from a homogeneous Poisson process. A detailed description of the model equations and parameters including its biophysical meaning is provided in [1, 2].

The results of the unified model numerical solution confirm the emergence of calcium waves, which occur due to the synaptic activity and spread over the astrocyte network (Fig. 1B). Depending on the excitation level and the network topology, the combination of two possible scenarios is forming: calcium excitation wave captures the entire astrocyte network, along with local waves, which exist only within one cell and terminate beyond its borders. The first scenario includes the regime when one of the cells acts as a pacemaker, i.e. the source of periodic calcium waves (Fig. 1C). The statistics on area and duration of calcium excitation events in the case of the presence and absence of NCX regulations was obtained using complementary cumulative distribution functions (CCDF). The presence of NCX leads to a decrease in the average areas that are affected by a global calcium wave during excitation, while the number of events with equal duration time is the same on average for both models (Fig. 1D). However, the Na/Ca-exchanger stimulates calcium waves, making possible the formation of more long-lived waves.

**Acknowledgements:** This study was supported by Russian Science Foundation, grant 17-74-20089.

**References**

1. Verveyko DV, et al. Raindrops of synaptic noise on dual excitability landscape: an approach to astrocyte network modelling. *Proceedings SPIE* 2018, 10717, 107171S.
2. Brazhe AR, et al. Sodium–calcium exchanger can account for regenerative Ca2+ entry in thin astrocyte processes. *Frontiers in Cellular Neuroscience* 2018, 12, 250.

## P24 Building a computational model of aging in visual cortex

### Seth Talyansky^1^, Braden Brinkman^2^

#### ^1^Catlin Gabel School, Portland, OR, United States of America; ^2^Stony Brook University, Department of Neurobiology and Behavior, Stony Brook, NY, United States of America

##### **Correspondence:** Seth Talyansky (talyanskys@catlin.edu)

*BMC Neuroscience* 2019, **20(Suppl 1)**:P24

The mammalian visual system has been the focus of countless experimental and theoretical studies designed to elucidate principles of sensory coding. Most theoretical work has focused on networks intended to reflect developing or mature neural circuitry, in both health and disease. Few computational studies have attempted to model changes that occur in neural circuitry as an organism ages non-pathologically. In this work we begin to close this gap, studying how physiological changes correlated with advanced age impact the computational performance of a spiking network model of primary visual cortex (V1).

Senescent brain tissue has been found to show increased excitability [1], decreased GABAergic inhibition [2], and decreased selectivity to the orientation of grating stimuli [1]. While the underlying processes driving these changes with age are far from clear, we find that these observations can be replicated by a straightforward, biologically-interpretable modification to a spiking network model of V1 trained on natural image inputs using local synaptic plasticity rules [3]. Specifically, if we assume the homeostatically-maintained excitatory firing rate increases with “age” (duration of training), a corresponding decrease in network inhibition follows naturally due to the synaptic plasticity rules that shape network architecture during training. The resulting aged network also exhibits a loss in orientation selectivity (Fig. 1).

**Fig. 1:**
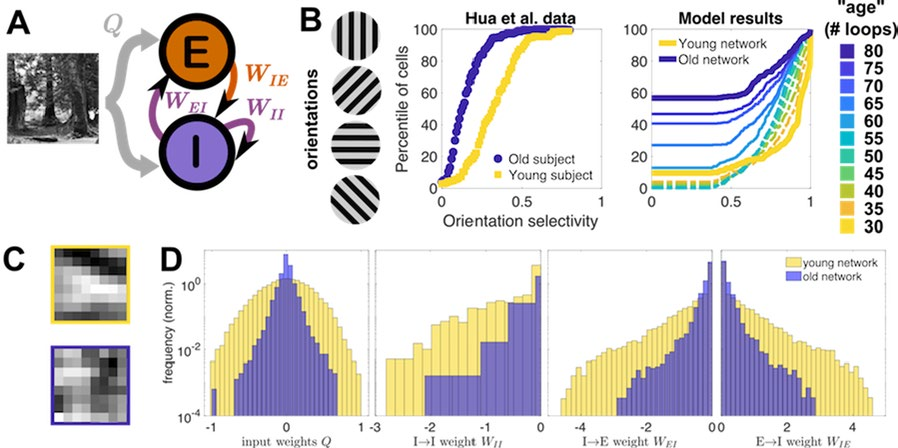
**a** Model schematic (see [3]). **b** Cumulative distribution of experimental [1] and model orientation selectivities. Model “ages” correspond to training loops as target firing increases. Thin dashed (solid) lines correspond to early (late) stages of aging. **c** An example neuron’s young (top) vs. old (bottom) receptive field. **d** Young vs. old distributions of input and lateral weights

In addition to qualitatively replicating previously observed changes, our trained model allows us to probe how the network properties evolve during aging. For example, we statistically characterize how the receptive fields of model neurons change with age: we find that 31% of young model neuron receptive fields are well-characterized as Gabor-like; this drops to 6.5% in the aged network. Only 1.5% of neurons were Gabor-like in both youth and old age, while 5% of neurons that were not classified as Gabor-like in youth were in old age. As one might intuit, these changes are tied to the decrease in orientation selectivity: by remapping the distribution of strengths of the young receptive fields to match the strength distribution of the old receptive fields, while otherwise maintaining the receptive field structure, we can show that orientation selectivity is improved at every age.

Our results demonstrate that deterioration of homeostatic regulation of excitatory firing, coupled with long-term synaptic plasticity, is a sufficient mechanism to reproduce features of observed biogerontological data, specifically declines in selectivity and inhibition. This suggests a potential causality between dysregulation of neuron firing and age-induced changes in brain physiology and performance. While this does not rule out deeper underlying causes or other mechanisms that could give rise to these changes, our approach opens new avenues for exploring these underlying mechanisms in greater depth and making predictions for future experiments.

**References**

1. Hua, Li, He, et al. Functional degradation of visual cortical cells in old cats. *Neurobiol. Aging* 2006, 27, 155–162.
2. Hua, Kao, Sun, et al. Decreased proportion of GABA neurons accompanies age-related degradation of neuronal function in cat striate cortex. *Brain Research Bulletin* 2008, 75, 119–125.
3. King, Zylberberg, DeWeese. Inhibitory Interneurons Decorrelate Excitatory Cells to Drive Sparse Code Formation in a Spiking Model of V1. *Journal of Neuroscience* 2013, 33, 5475–5485.

## P25 Toward a non-perturbative renormalization group analysis of the statistical dynamics of spiking neural populations

### Braden Brinkman

#### Stony Brook University, Department of Neurobiology and Behavior, Stony Brook, NY, United States of America

##### **Correspondence:** Braden Brinkman (braden.brinkman@stonybrook.edu)

*BMC Neuroscience* 2019, **20(Suppl 1)**:P25

Understanding how the brain processes sensory input and performs computations necessarily demands we understand the collective behavior of networks of neurons. The tools of statistical physics are well-suited to this task, but neural populations present several challenges: neurons are organized in a complicated web of connections–rather than crystalline arrangements statistical physics tools were developed for, neural dynamics are often far from equilibrium, and neurons communicate not by gradual changes in their membrane potential but by all-or-nothing spikes. These all-or-nothing spike dynamics render it difficult to treat neuronal network models using field theoretic techniques, though recently Ocker et al. [1] formulated such a representation for a stochastic spiking model and derived diagrammatic rules to calculate perturbative corrections to the mean field approximation. In this work we use an alternate representation of this model that is amenable to the methods of the non-perturbative renormalization group (NPRG), which has successfully elucidated the different phases of collective behavior in several non-equilibrium models in statistical physics. In particular, we use the NPRG to calculate how stochastic fluctuations modify the nonlinear transfer function of the network, which determines the mean neural firing rates as a function of input, and how these changes depend on network structure. Specifically, the mean field approximation of the neural firing rates r receiving current input I and synaptic connections J is r = f(I+J ∗ r), where f(x) is the nonlinear firing rate of a neuron conditioned on its input x. We show exactly that the true mean, accounting for statistical fluctuations, follows the same form of equation, r = U(I+J ∗ r), where U(x) is an effective nonlinearity to be calculated using NPRG approximation methods.

**Reference**

1. Ocker G, Josić K, Shea-Brown E, Buice M. Linking Structure and Activity in Nonlinear Spiking Networks. *PLoS Comput Biology* 2017, 13(6): e1005583.

## P26 Sensorimotor strategies and neuronal representations of whisker-based object recognition in mice barrel cortex

### Ramon Nogueira^1^, Chris Rodgers^1^, Stefano Fusi^2^, Randy Bruno^1^

#### ^1^Columbia University, Center for Theoretical Neuroscience, New York, NY, United States of America; ^2^Columbia University, Zuckerman Mind Brain Behavior Institute, New York, United States of America

##### **Correspondence:** Ramon Nogueira (rnogueiramanas@gmail.com)

*BMC Neuroscience* 2019, **20(Suppl 1)**:P26

Humans and other animals can identify objects by active touch—coordinated exploratory motion and tactile sensation. Rodents, and in particular mice, scan objects by active whisking, which allows them to form an internal representation of the physical properties of the object. In order to elucidate the behavioral and neural mechanisms underlying this ability, we developed a novel curvature discrimination task for head-fixed mice that challenges the mice to discriminate concave from convex shapes (Fig. 1a). On each trial, a curved shape was presented into the range of the mouse’s whiskers and they were asked to lick left for concave and right for convex shapes. Whisking and contacts were monitored with high-speed video. Mice learned the task well and their performance plateaued at 75.7% correct on average (chance 50% correct).

**Fig. 1:**
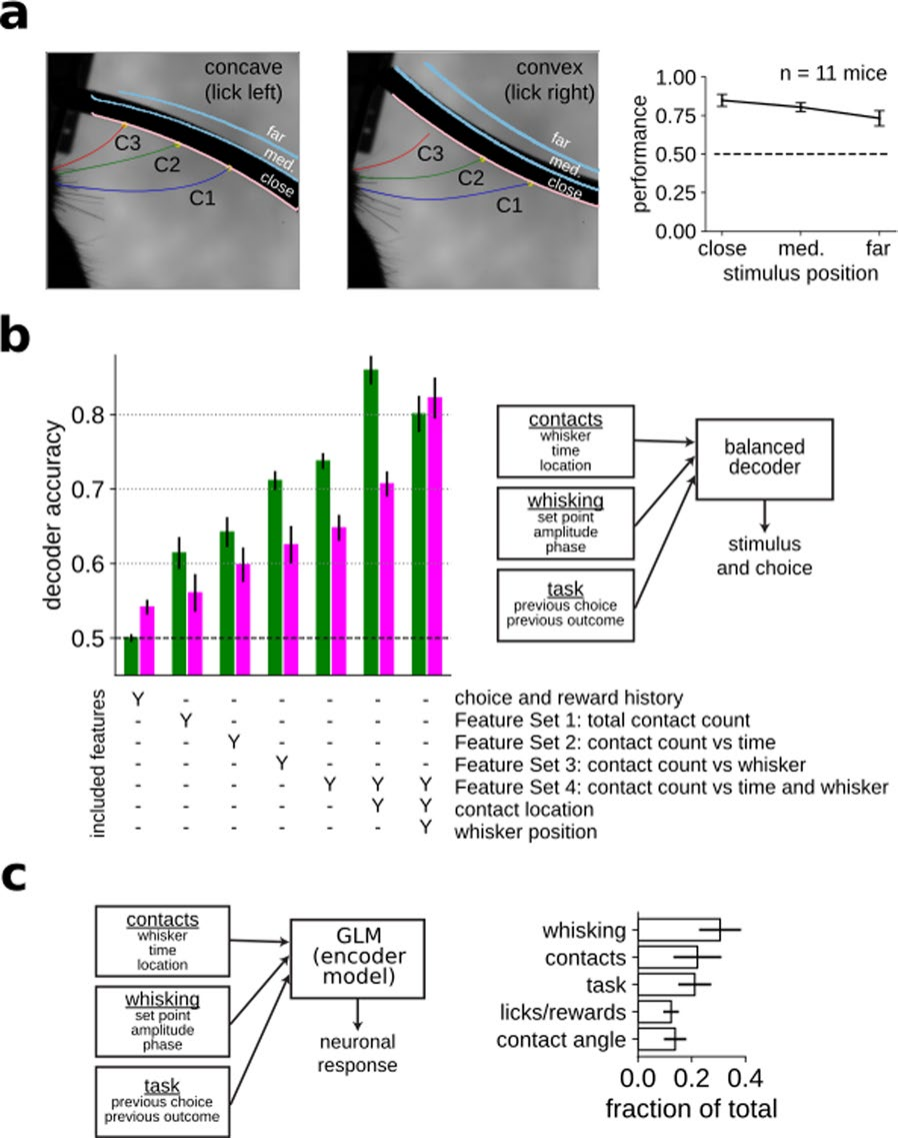
**a** Mice were trained to perform a curvature discrimination task. The identity and position of each whisker was monitored with high-speed video. **b** By increasing the complexity of the regressors used to predict stimulus and choice, we identified the most informative features and the features driving behavior. **c** Neurons in the barrel cortex encode a myriad of sensory and task related variables

Because most previous work has relied on mice detecting the presence or location of a simple object with a single whisker, it is a priori unclear what sensorimotor features are important for more complex tasks such as curvature discrimination. To characterize them, we trained a classifier to identify either the stimulus identity or the mouse’s choice on each trial using the entire suite of sensorimotor variables (whisker position, contact timing and position, contact kinematics, etc.) that could potentially drive behavior, as well as task related variables that could also affect behavior (Fig. 1b). By increasing the complexity and richness of the set of features used to perform the classification of stimulus and choice, we identified what features were most informative to perform the task and what features were driving animal’s decision, respectively. We found that the cumulative number of contacts per trial for each whisker independently was informative about the stimulus and choice identity. Surprisingly, precise contact timings within a trial for the different whiskers was not an important feature in either case. Additionally, the exact angular position of each whisker during contacts was highly predictive of the stimulus identity, suggesting that the mice’s behavior was not fully optimal, since this same feature could not predict mice’s choice accurately on a trial-by-trial basis.

In order to identify how barrel cortex contributes to transforming fine-scale representations of sensory events into high-level representations of object identity, we recorded neural populations in mice performing this task. We fit a generalized linear model (GLM) to each neuron’s firing rate as a function of both sensorimotor (e.g., whisker motion and touch) and cognitive (e.g., reward history) variables (Fig. 1c). Neurons responded strongly to whisker touch and, perhaps more surprisingly for a sensory area, to whisker motion. We also observed widespread and unexpected encoding of reward history and choice.

In conclusion, these results show that mice recognize objects by integrating sensory information gathered by active sampling across whiskers. Moreover, we find that the barrel cortex encodes a myriad of sensory and task related variables, like contacts, motor exploration, and reward and choice history, challenging the classical view of barrel cortex as a purely sensory area.

## P27 Identifying the neural circuits underlying optomotor control in larval zebrafish

### Winnie Lai^1^, John Holman^1^, Paul Pichler^1^, Daniel Saska^2^, Leon Lagnado^2^, Christopher Buckley^3^

#### ^1^University of Sussex, Department of Informatics, Brighton, United Kingdom; ^2^University of Sussex, Department of Neuroscience, Brighton, United Kingdom; ^3^University of Sussex, Falmer, United Kingdom

##### **Correspondence:** Winnie Lai (wl291@sussex.ac.uk)

*BMC Neuroscience* 2019, **20(Suppl 1)**:P27

Most locomotor behaviours require the brain to instantaneously coordinate a continuous flow of sensory and motor information. As opposed to the conventional open-loop approach in the realm of neuroscience, it has been proposed that the brain is better idealised as a closed-loop controller which regulates dynamical motor actions. Studying brain function on these assumptions remains largely unexplored until the recent emergence of imaging techniques, such as the SPIM, which allow brain-wide neural recording at cellular resolution and high speed during active behaviours. Concurrently, larval zebrafish is becoming a powerful model organism in neuroscience due to their great optical accessibility and robust sensorimotor behaviours. Here, we apply control theory in engineering to investigate the neurobiological basis of the optomotor response (OMR), a body reflex to stabilise optic flow in the presence of whole-field visual motion, in larval zebrafish.

Our group recently developed a collection of OMR models based on variations of proportional-integral controllers. Whilst the proportional term allows rapid response to disturbance, the integral term eliminates the steady-state error over time. We will begin by characterising OMR adaption with respect to different speeds and heights, in both free-swimming and head-restrained environments. Data collected will be used to determine which model best captures zebrafish behaviour. Next, we will conduct functional imaging of fictively behaving animals under a SPIM that our group constructed, in an effort to examine how the control mechanism underpinning OMR is implemented and distributed in the neural circuitry of larval zebrafish. This research project will involve evaluating and validating biological plausible models inspired by control theory, as well as quantifying and analysing large behavioural and calcium imaging data sets. Understanding the dynamical nature of brain function for successful OMR control in larval zebrafish can provide unique insight into the neuropathology of diseases with impaired movement and/or offer potential design solutions for sophisticated prosthetics.

## P28 A novel learning mechanism for interval timing based on time cells of hippocampus

### Sorinel Oprisan^1^, Tristan Aft^1^, Mona Buhusi^2^, Catalin Buhusi^2^

#### ^1^College of Charleston, Department of Physics and Astronomy, Charleston, SC, United States of America; ^2^Utah State University, Department of Psychology, Logan, UT, United States of America

##### **Correspondence:** Sorinel Oprisan (oprisans@cofc.edu)

*BMC Neuroscience* 2019, **20(Suppl 1)**:P28

Time cells were recently discovered in the hippocampus and they seem to ramp-up their firing when the subject is at a specific temporal marker in a behavioral test. At cellular level, the spread of the firing interval, i.e. the width of the Gaussian-like activity, for each time cell is proportional to the time of the peak activity. Such a linear relationship is well-known at behavioral level and is called scalar property of interval timing.

We proposed a novel mathematical model for interval timing starting with a population of hippocampal time cells and a dynamic learning rule. We hypothesized that during the reinforcement trials the subject learns the boundaries of the temporal duration. Subsequently, a population of time cells is recruited and coverers the entire to-be-timed duration. At this stage, the population of time cells simply produces a uniform average time field since all time cells contribute equally to the average. We hypothesized that dopamine could modulate the activity of time cells during reinforcement trials by enhancing/depressed their activity. Our numerical simulations of the model agree with behavioral experiments.

**Acknowledgments:** We acknowledge the support of R&D grant from the College of Charleston and support for a Palmetto Academy site from the South Carolina Space Grant Consortium.

**References**

1. Oprisan SA, Aft T, Buhusi M, Buhusi CV. Scalar timing in memory: A temporal map in the hippocampus. *Journal of Theoretical Biology* 2018, 438:133–142.
2. Oprisan SA, Buhusi M, Buhusi CV. A Population-Based Model of the Temporal Memory in the Hippocampus. *Frontiers in Neuroscience* 2018, 12:521.

## P29 Learning the receptive field properties of complex cells in V1

### Yanbo Lian^1^, Hamish Meffin^2^, David Grayden^1^, Tatiana Kameneva^3^, Anthony Burkitt^1^

#### ^1^University of Melbourne, Department of Biomedical Engineering, Melbourne, Australia; ^2^University of Melbourne, Department of Optometry and Visual Science, Melbourne, Australia; ^3^Swinburne University of Technology, Telecommunication Electrical Robotics and Biomedical Engineering, Hawthorn, Australia

##### **Correspondence:** Anthony Burkitt (aburkitt@unimelb.edu.au)

*BMC Neuroscience* 2019, **20(Suppl 1)**:P29

There are two distinct classes of cells in the visual cortex: simple cells and complex cells. One defining feature of complex cells is their phase invariance, namely that they respond strongly to oriented bar stimuli with a preferred orientation but with a wide range of phases. A classical model of complex cells is the energy model, in which the responses are the sum of the squared outputs of two linear phase-shifted filters. Although the energy model can capture the observed phase invariance of complex cells, a recent study has shown that complex cells have a great diversity and only a subset can be characterized by the energy model [1]. From the perspective of a hierarchical structure, it is still unclear how a complex cell pools input from simple cells, which simple cells should be pooled, and how strong the pooling weights should be. Most existing models overlook many biologically important details, e.g., some models assume a quadratic nonlinearity of the linear filtered simple cell activity, use pre-determined weights between simple and complex cells, or use artificial learning rules. Hosoya&Hyvarinen [2] applied strong dimension reduction in pooling simple cell receptive fields trained using independent component analysis. Their approach involves pooling simple cells, but the weights connecting simple and complex cells are not learned and thus it is unclear how this can be biophysically implemented.

We propose a biologically plausible learning model for complex cells that pools inputs from simple cells. The model is a 3-layer network with rate-based neurons that describes the activities of LGN cells (layer 1), V1 simple cells (layer 2), and V1 complex cells (layer 3). The first two layers implement a recently proposed simple cell model that is biologically plausible and accounts for many experimental phenomena [3]. The dynamics of the complex cells involves the linear summation of responses of simple cells that are connected to complex cells, taken in our model to be excitatory. Connections between LGN and simple cells are learned based on Hebbian and anti-Hebbian plasticity, similar to that in our previous work [3]. For connections between simple and complex cells that are learned using natural images as input, a modified version of the Bienenstock, Cooper, and Munro (BCM) rule [4] is investigated.

Our results indicate that the learning rule can describe a diversity of individual complex cells, similar to that observed experimentally, that pool inputs from simple cells with similar orientation but differing phases. Preliminary results support the hypothesis that normalized BCM [5] can lead to competition between complex cells and they thereby pool inputs from different groups of simple cells. In summary, this study provides a plausible explanation for how complex cells can be learned using biologically plasticity mechanisms.

**References**

1. Almasi A. An investigation of spatial receptive fields of complex cells in the primary visual cortex *Doctoral dissertation* 2017.
2. Hosoya H, Hyvärinen A. Learning visual spatial pooling by strong pca dimension reduction. *Neural computation* 2016 Jul;28(7):1249–64.
3. Lian Y, Meffin H, Grayden DB, Kameneva T, Burkitt AN. Towards a biologically plausible model of LGN-V1 pathways based on efficient coding. *Frontiers in Neural Circuits* 2019;13:13.
4. Bienenstock EL, Cooper LN, Munro PW. Theory for the development of neuron selectivity: orientation specificity and binocular interaction in visual cortex. *Journal of Neuroscience* 1982 Jan 1;2(1):32–48.
5. Willmore BD, Bulstrode H, Tolhurst DJ. Contrast normalization contributes to a biologically-plausible model of receptive-field development in primary visual cortex (V1). *Vision research* 2012 Feb 1;54:49–60.

## P30 Bursting mechanisms based on interplay of the Na/K pump and persistent sodium current

### Gennady Cymbalyuk^1^, Christian Erxleben^2^, Angela Wenning-Erxleben^2^, Ronald Calabrese^2^

#### ^1^Georgia State University, Neuroscience Institute, Atlanta, GA, United States of America; ^2^Emory University, Department of Biology, Atlanta, GA, United States of America

##### **Correspondence:** Gennady Cymbalyuk (gcymbalyuk@gmail.com)

*BMC Neuroscience* 2019, **20(Suppl 1)**:P30

Central Pattern Generators produce robust bursting activity to control vital rhythmic functions like breathing and leech heart beating under variable environmental and physiological conditions. Their functional operation under different physiological parameters yields distinct dynamic mechanisms based on the dominance of interactions within different subsets of inward and outward currents. Recent studies provide evidence that the Na+/K+ pump contributes to the dynamics of neurons and is a target of neuromodulation [1, 2]. Recently, we have described a complex interaction of the pump current and h-current that plays a role in the dynamics of rhythmic neurons in the leech heartbeat CPG, where the basic building blocks are half-center oscillators (HCOs): pairs of mutually inhibitory HN interneurons producing alternating bursting activity. In the presence of h-current, application of the H+/Na+ antiporter monensin, which stimulates the pump by diffusively increasing the intracellular Na+ concentration [3], dramatically decreases the period of a leech heartbeat HCO by decreasing both the burst duration (BD) and interburst interval (IBI). If h-current is blocked then monensin decreases BD but lengthens IBI so that there is no net change of period with respect to control. This mechanism shows how each phase of bursting, BD and IBI, can be independently controlled by interaction of the pump and h-currents.

We implemented our model [3] into a hybrid system. We investigated a potential role played by the persistent Na+ current (IP), sodium current which does not inactivate. Our hybrid-system allowed us to upregulate or downregulate the Na+/K+ pump and key ionic currents in real-time models and living neurons. We were able to tune the real time model to support functional-like bursting. We investigated how the variation of the basic physiological parameters like conductance and voltage of half-activation of IP and strength of the Na+/K+ pump affect bursting characteristics in single neurons and HCO. We show that interaction of IP and Ipump constitutes a mechanism which is sufficient to support endogenous bursting activity. We show that this mechanism can reinstate robust bursting regime in HN interneurons recorded intracellularly in ganglion 7. Due to interaction of IPand Ipump, the increase of the maximal conductance of IP can shorten the burst duration and expand the interburst interval. Our data also suggest that the functional alternating bursting regime of the HCO network requires the neurons to be in the parametric vicinity of or in the state of the endogenous bursting. We investigated underlying interaction of the IP and Ipump in a simple 2D model describing dynamics of the membrane potential and intracellular Na+ concentration through instantaneous IP and IPump.

**Acknowledgements:** Supported by NINDS 1 R01 NS085006 to RLC and1 R21 NS111355 to RLC and GSC

**References**

1. Tobin AE, Calabrese R. Myomodulin increases Ih and inhibits the NA/K pump to modulate bursting in leech heart interneurons. *Journal of neurophysiology* 2005 Dec;94(6):3938–50.
2. Picton LD, Nascimento F, Broadhead MJ, Sillar KT, Miles GB. Sodium pumps mediate activity-dependent changes in mammalian motor networks. *Journal of Neuroscience* 2017 Jan 25;37(4):906–21.
3. Kueh D, Barnett W, Cymbalyuk G, Calabrese R. Na(+)/K(+) pump interacts with the h-current to control bursting activity in central pattern generator neurons of leeches. *Elife*, 2016. Sep 2;5:e19322.

## P31 Balanced synaptic strength regulates thalamocortical transmission of informative frequency bands

### Alberto Mazzoni^1^, Matteo Saponati^2^, Jordi Garcia-Ojalvo^3^, Enrico Cataldo^2^

#### ^1^Scuola Superiore Sant’Anna Pisa, The Biorobotics Institute, Pisa, Italy; ^2^University of Pisa, Department of Physics, Pisa, Italy; ^3^Universitat Pompeu Fabra, Department of Experimental and Health Sciences, Barcelona, Spain

##### **Correspondence:** Alberto Mazzoni (a.mazzoni@santannapisa.it)

*BMC Neuroscience* 2019, **20(Suppl 1)**:P31

The thalamus receives information about the external world from the peripheral nervous system and conveys it to the cortex. This is not a passive process: the thalamus gates and selects sensory streams through an interplay with its internal activity, and the inputs from the thalamus, in turn, interact in a non-linear way with the functional architecture of the primary sensory cortex. Here we address the network mechanisms by which the thalamus selectively transmits informative frequency bands to the cortex. In particular, spindle oscillations (about 10 Hz) dominate thalamic activity during sleep but are present in the thalamus also during wake [1, 2], and in the awake state are actively filtered out by thalamocortical transmission [3].

To reproduce and understand the filtering mechanism underlying the lack of thalamocortical transmission of spindle oscillations we developed an integrated adaptive exponential integrate-and-fire model of the thalamocortical network. The network is composed by 500 neurons for the thalamus and 5000 neurons for the cortex, with a 1:1 and 1:4 inhibitory to excitatory ratio respectively. We generated the local field potential (LFP) associated to the two networks to compare our simulation with experimental results [3].

Weobserve, in agreement withexperimental data, both delta and theta oscillations in the cortex, but while the cortical delta band is phase locked to thethalamic delta band [4]– even when we take into account the presence of strong colored cortical noise -, the cortical theta fluctuations are not entrained by thalamocortical spindles. Our simulations show that the spindleLFPoscillationsobserved in experimental recordings are way more pronounced in reticular cells than in thalamocortical relays, thus reducing their potential impact on the cortex. More interestingly, we found that the resonance dynamics in the corticalgamma band, generated by the fast interplay between excitation and inhibition, selectively dampens frequencies in the range of spindle oscillations. Finally, by parametrically varying the properties of thalamocortical connections, we found that the transmission of informative frequency bands depends on the balance of the strength of thalamocortical connections toward excitatory and inhibitory neurons in the cortex, coherently with experimental results [5]. Our results pave the way toward an integrated view of the processing of sensory streams from the periphery system to the cortex, and toward in silico design of thalamic neural stimulation.

**References**

1. Krishnan GP, Chauvette S, Shamiee I et al. Cellular and neurochemical basis of sleep stages in the thalamocortical network, *eLife* 2016, e18607.
2. Barardi A, Garcia-Ojalvo J, Mazzoni A. Transition between Functional Regimes in an Integrate-And-Fire Network Model of the Thalamus. *PLoS One* 2016, e0161934.
3. Bastos AM, Briggs F, Alitto HJ, et al. Simultaneous Recordings from the Primary Visual Cortex and Lateral Geniculate Nucleus Reveal Rhythmic Interactions and a Cortical Source for Gamma-Band Oscillations. *Journal of Neurosci*ence 2014, 7639–7644.
4. Lewis LD, Voigts J, Flores FJ, et al. Thalamic reticular nucleus induces fast and local modulation of arousal state. *eLife* 2015, e08760.
5. Sedigh-Sarvestani M, Vigeland L, Fernandez-Lamo I, et al. Intracellular, in vivo, dynamics of thalamocortical synapses in visual cortex. *Journal of Neurosci*ence 2017,5250–5262.

## P32 Modeling gephyrin dependent synaptic transmission pathways to understand how gephyrin regulates GABAergic synaptic transmission

### Carmen Alina Lupascu^1^, Michele Migliore^1^, Annunziato Morabito^2^, Federica Ruggeri^2^, Chiara Parisi^2^, Domenico Pimpinella^2^, Rocco Pizzarelli^2^, Giovanni Meli^2^, Silvia Marinelli^2^, Enrico Cherubini^2^, Antonino Cattaneo^2^

#### ^1^Institute of Biophysics, National Research Council, Italy; ^2^European Brain Research Institute (EBRI), Rome, Italy

##### **Correspondence:** Carmen Alina Lupascu (carmen.lupascu@pa.ibf.cnr.it)

*BMC Neuroscience* 2019, **20(Suppl 1)**:P32

At inhibitory synapses, GABAergic signaling controls dendritic integration, neural excitability, circuit reorganization and fine tuning of network activity. Among different players, the tubulin-binding protein gephyrin plays a key role in anchoring GABAA receptors to synaptic membranes.

For its properties gephyrin is instrumental in establishing and maintaining a proper excitatory (E)/inhibitory (I) balance necessary for the correct functioning of neuronal networks. A disruption of the E/I balance is thought to be at the origin of several neuropsychiatric disorders including epilepsy, schizophrenia, autism.

In previous studies, the functional role of gephyrin on GABAergic signaling has been studied at post-translational level, using recombinant gephyrin-specific single chain antibody fragments (scFv-gephyrin) containing a nuclear localization signal able to remove endogenous gephyrin from GABAA receptor clusters retargeting it to the nucleus [2]. The reduced accumulation of gephyrin at synapses led to a significant reduction in amplitude and frequency of spontaneous and miniature inhibitory postsynaptic currents (sIPSCs and mIPSCs). This reduction is associated with a decrease in VGAT (the vesicular GABA transporter) and in neuroligin 2 (NLG2), a protein that ensures the cross-talk between the post- and presynaptic sites. Over-expressing NLG2 in gephyrin deprived neurons rescued GABAergic but not glutamatergic innervation, suggesting that the observed changes in the latter were not due to a homeostatic compensatory mechanism. These results suggest a key role of gephyrin in regulating trans-synaptic signaling at inhibitory synapses.

Here, the effects of two different intrabodies against gephyrin have been tested on spontaneous and miniature GABAA-mediated events obtained from cultured hippocampal and cortical neurons. Experimental findings have been used to develop a computational model describing the key role of gephyrin in regulating transynaptic signalingat inhibitory synapses. This represents a further application of a general procedure to study subcellular models of transsynaptic signaling at inhibitory synapses [1]. In this poster we will discuss the statistically significant differences found between the model parameters under control or gephyrin block condition. All computational procedures were carried out using an integrated NEURON and Python parallel code on different systems (JURECA machines, Julich, Germany; MARCONI machine, Cineca, Italy and Neuroscience Gateway, San Diego, USA). The model can be downloaded from the model catalog available on the Collaboratory Portal of Human Brain Project (HBP) (https://collab.humanbrainproject.eu/#/collab/1655/nav/75901?state = model.9f89bbcd-e045-4f1c-97e9-3da5847356c2). The jupyter notebooks used to configure and run the jobs on the HPC machines can be accessed from the Brain Simulation Platform of the HBP (https://collab.humanbrainproject.eu/#/collab/1655/nav/66850).

**References**

1. Lupascu CA, Morabito A, Merenda E, et al. A General Procedure to Study Subcellular Models of Transsynaptic Signaling at Inhibitory Synapses. *Frontiers in Neuroinformatics* 2016;10:23.
2. Marchionni I, Kasap Z, Mozrzymas JW, Sieghart W, Cherubini E, Zacchi P. New insights on the role of gephyrin in regulating both phasic and tonic GABAergic inhibition in rat hippocampal neurons in culture. *Neuroscience* 2009 164: 552–562

## P33 Proprioceptive feedback effects muscle synergy recruitment during an isometric knee extension task

### Hugh Osborne^1^, Gareth York^2^, Piyanee Sriya^2^, Marc de Kamps^3^, Samit Chakrabarty^2^

#### ^1^University of Leeds, Institute for Artificial and Biological Computation, School of Computing, United Kingdomv^2^University of Leeds, School of Biomedical Sciences, Faculty of Biological Sciences, United Kingdom; ^3^University of Leeds, School of Computing, Leeds, United Kingdom

##### **Correspondence:** Hugh Osborne (sc16ho@leeds.ac.uk)

*BMC Neuroscience* 2019, **20(Suppl 1)**:P33

The muscle synergy hypothesis of motor control posits that simple common patterns of muscle behaviour are combined together to produce complex limb movements. How proprioception influences this process is not clear. EMG recordings were taken of the upper leg muscles during an isometric knee extension task (n = 17, male; 9, female; 8). The internal knee angle was held at 0°, 20°, 60° or 90°. Non-negative matrix factorisation (NMF) was performed on the EMG traces and two synergy patterns were identified accounting for over 90% of the variation across participants. The first synergy indicated the expected increase in activity across all muscles which was also visible in the raw EMG. The second synergy showed a significant difference between coefficients of the knee flexors and extensors, highlighting their agonist/antagonist relationship. As the leg was straightened, the flexor-extensor difference in the second synergy became more pronounced indicating a change in passive insufficiency of the hamstring muscles. Changing hip position and reducing the level of passive insufficiency resulted in delayed onset of the second synergy pattern. An additional observation of bias in the Rectus Femoris and Semitendinosus coefficients of the second synergy was made, perhaps indicating the biarticular behaviour of these muscles.

Having demonstrated that static proprioceptive feedback influences muscle synergy recruitment we then reproduced this pattern of activity in a neural population model. We used the MIIND neural simulation platform to build a network of populations of motor neurons and spinal interneurons with a simple Integrate and Fire neuron model. MIIND provides an intuitive system for developing such networks and simulating with an appropriate and well-defined amount of noise. The simulator can handle large, quick changes in activity with plausible postsynaptic potentials. Two mutually inhibiting populations of both excitatory and inhibitory interneurons were connected to five motor neuron populations, each with a balanced descending input. A single excitatory input to the extensor interneuron pool was used to indicate the level of afferent activity due to the static knee angle. By applying the same NMF step to the activity of the motor neuron populations, the same muscle synergies were observed, with increasing levels of afferent activity resulting in changes to agonist/antagonist recruitment. When the trend in afferent activity is taken further such that it is introduced to the flexor interneuron population, extensor synergy coefficients and vectors increase, leaving the flexor coefficients at zero. This shift from afferent feedback in the agonists to antagonists is predicted by the model but has yet to be confirmed with joint angles beyond 90 degrees.

With the introduction of excitatory connections from the flexor interneuron pool to the Rectus Femoris motor neuron population, the biarticular synergy association, which is proportional to the knee angle, was also reproduced in the model. Even with this addition, there is no need to provide a cortical bias to any individual motor neuron population. The synergies arise naturally from the connectivity of the network and afferent input. This suggests muscle synergies could be generated at the level of spinal interneurons wherein proprioceptive feedback is directly integrated into motor control.

## P34 Strategies of dragonfly interception

### Frances Chance

#### Sandia National Laboratories, Department of Cognitive and Emerging Computing, Albuquerque, NM, United States of America

##### **Correspondence:** Frances Chance (fschanc@sandia.gov)

*BMC Neuroscience* 2019, **20(Suppl 1)**:P34

Interception of a target (e.g. a human catching a ball or an animal catching prey) is a common behavior solved by many animals. However, the underlying strategies used by animals are poorly understood. For example, dragonflies are widely recognized as highly successful hunters, with reports of up to 97% success rates [1], yet a full description of their interception strategy, whether it be to head directly at its target (a strategy commonly referred to as pursuit) or instead to maintain a constant bearing-angle relative to the target (sometimes referred to as proportional or parallel navigation) still has yet to be fully developed (see [2]). While parallel navigation is the logical strategy for calculating the shortest time-to-intercept, we find that there are certain conditions (for example if the prey is capable of relatively quick maneuvers) in which parallel navigation is not the optimal strategy for success. Moreover, recent work [2] observed that dragonflies only adopt a parallel-navigation strategy for a short period of time shortly before prey-capture. We propose that alternate strategies, hybrid between pursuit and parallel navigation lead to more successful interception, and describe what constraints (e.g. prey maneuvering) determine which interception strategy is optimal for the dragonfly. Moreover, we compare dragonfly interception strategy to those that might be employed by other animals, for example other predatory insects that may not be capable of flying speeds similar to those of the dragonfly. Finally, we discuss neural circuit mechanisms by which interception strategy, as well as intercept-maneuvers, may be calculated based on prey-image slippage on the dragonfly retina.

This paper describes objective technical results and analysis. Any subjective views or opinions that might be expressed in the paper do not necessarily represent the views of the U.S. Department of Energy or the United States Government.

**Acknowledgements:** Sandia National Laboratories is a multimission laboratory managed and operated by National Technology & Engineering Solutions of Sandia, LLC, a wholly owned subsidiary of Honeywell International Inc., for the U.S. Department of Energy’s National Nuclear Security Administration under contract DE-NA0003525. SAND2019-2782A

**References**

1. Olberg RM, Worthington AH, Venator KR. Prey pursuit and interception in dragonflies. *Journal of Comparative Physiology A* 2000 Feb 1;186(2):155–62.
2. Mischiati M, Lin HT, Herole O, Imler E, Olberg R, Leonardo, A. Internal models direct dragonfly interception steering. *Nature* 2015, 517: 333–338.

## P35 The bump attractor model predicts spatial working memory impairment from changes to pyramidal neurons in the aging rhesus monkey dlPFC

### Sara Ibanez Solas^1^, Jennifer Luebke^2^, Christina Weaver^1^, Wayne Chang^2^

#### ^1^Franklin and Marshall College, Department of Mathematics and Computer Science, Lancaster, PA, United States of America; ^2^Boston University School of Medicine, Department of Anatomy and Neurobiology, Boston, MA, United States of America

##### **Correspondence:** Sara Ibanez Solas (sibanezs@fandm.edu)

*BMC Neuroscience* 2019, **20(Suppl 1)**:P35

Behavioral studies have shown impairment in performance during spatial working memory (WM) tasks with aging in several animal species, including humans. Persistent activity (PA) during delay periods of spatial WM tasks is thought to be the main mechanism underlying spatial WM, since the selective firing of pyramidal neurons in the dorsolateral prefrontal cortex (dlPFC) to different spatial locations seems to encode the memory of the stimulus. This firing activity is generated by recurrent connections between layer 3 pyramidal neurons in the dlPFC, which, as many in vitro studies have shown, undergo significant structural and functional changes with aging. However, the extent to which these changes affect the neural mechanisms underlying spatial WM, and thus cognition, is not known. Here we present the first empirical evidence that spatial WM in the rhesus monkey is impaired in some middle-aged subjects, and show that spatial WM performance is negatively correlated with hyperexcitability (increased action potential firing rates) of layer 3 pyramidal neurons. We used the bump attractor network model to explore the effects on spatial WM of two age-related changes to the properties of individual pyramidal neurons: the increased excitability observed here and previously [1, 2], and a 10-30% loss of both excitatory and inhibitory synapses in middle-aged and aged monkeys [3]. In particular, we simulated the widely used (Oculomotor) Delayed Response Task (DRT) and introduced a simplified model of the Delayed Recognition Span Task-spatial condition (DRST-s) which was administered to the monkeys in this study. The DRST-s task is much more complex than the DRT, requiring simultaneous encoding of multiple stimuli which successively increase in number. Simulations predicted that PA—and in turn WM performance—in both tasks was severely impaired by the increased excitability of individual neurons, but not by the loss of synapses alone. This is consistent with the finding in [3], where no correlations were seen between synapse loss and DRST-s impairment. Simulations also showed that pyramidal neuron hyperexcitability and synapse loss might compensate each other partially: the level of impairment in the DRST-s model with these simultaneous changes was similar to that seen in the DRST-s data from young vs. aged monkeys. The models also predict an age-related reduction in total synaptic input current to pyramidal neurons alongside changes to their f-I curves, showing that the increased excitability of pyramidal neurons we have seen in vitro is consistent with lower firing rates seen during DRT testing of middle-aged and aged monkeys in vivo [4]. Finally, in addition to PA, this study suggests that short-term synaptic facilitation plays an important (if often unappreciated) role in spatial WM.

**Acknowledgments:** We thank National Institute of Health (National Institute on Aging) for supporting the authors with Grant Number R01AG059028.

**References**

1. Chang YM, Rosene DL, Killiany RJ, Mangiamele LA, Luebke JI. Increased action potential firing rates of layer 2/3 pyramidal cells in the prefrontal cortex are significantly related to cognitive performance in aged monkeys. *Cerebral Cortex* 2004 Aug 5;15(4):409–18.
2. Coskren PJ, Luebke JI, Kabaso D, et al. Functional consequences of age-related morphologic changes to pyramidal neurons of the rhesus monkey prefrontal cortex. *Journal of computational neuroscience* 2015 Apr 1;38(2):263–83.
3. Peters A, Sethares C, Luebke JI. Synapses are lost during aging in the primate prefrontal cortex. *Neuroscience* 2008 Apr 9;152(4):970–81.
4. Wang M, Gamo NJ, Yang Y, et al. Neuronal basis of age-related working memory decline. *Nature* 2011 Aug;476(7359):210.

## P36 Brain dynamic functional connectivity: lesson from temporal derivatives and autocorrelations

### Jeremi Ochab^1^, Wojciech Tarnowski^1^, Maciej Nowak^1,2^, Dante Chialvo^3^

#### ^1^Jagiellonian University, Institute of Physics, Kraków, Poland; ^2^Mark Kac Complex Systems Research Center, Kraków, Poland; ^3^Universidad Nacional de San Martín and CONICET, Center for Complex Systems & Brain Sciences (CEMSC^3), Buenos Aires, Argentina

##### **Correspondence:** Jeremi Ochab (jeremi.ochab@uj.edu.pl)

*BMC Neuroscience* 2019, **20(Suppl 1)**:P36

The study of correlations between brain regions in functional magnetic resonance imaging (fMRI) is an important chapter of the analysis of large-scale brain spatiotemporal dynamics. The burst of research exploring momentary patterns of blood oxygen level-dependent (BOLD) coactivations, referred to as dynamic functional connectivity, has brought prospects of novel insights into brain function and dysfunction. It has been, however, closely followed by inquiries into pitfalls the new methods hold [1], and only recently by their systematic evaluation [2].

From among such recent measures, we scrutinize a metric dubbed “Multiplication of Temporal Derivatives” (MTD) [3] which is based on the temporal derivative of each time series. We compare it with the sliding window Pearson correlation of the raw time series in several stationary and non-stationary set-ups, including: simulated autoregressive models with a step change in their coupling, surrogate data [4] with realistic spectral and covariance properties and a step change in their cross- and autocovariance (see, Fig. 1, right panels), and a realistic stationary network detection (with the use of gold standard simulated data; [5]).

**Fig. 1:**
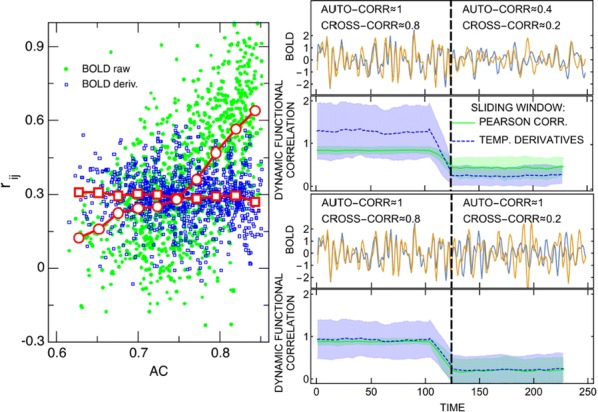
(Left) Cross-correlation of pairs of blood oxygen level-dependent (BOLD) signals and their derivatives versus their common auto-correlation; red markers show binned averages. (Right) a simulated step change in cross- and/or auto- correlations and the effect it has on dynamic functional correlation measures (Pearson sliding window and “multiplication of temporal derivatives”)

The formal comparison of the MTD formula with the Pearson correlation of the derivatives reveals only minor differences, which we find negligible in practice. The numerical comparison reveals lower sensitivity of derivatives to low frequency drifts and to autocorrelations but also lower signal-to-noise ratio. It does not indicate any evident mathematical advantages of the MTD metric over commonly used correlation methods.

Along the way we discover that cross-correlations between fMRI time series of brain regions are tied to their autocorrelations (see, Fig. 1, left panel). We solve simple autoregressive models to provide mathematical grounds for that behaviour. This observation is relevant to the occurrence of false positives in real networks and might be an unexpected consequence of current preprocessing techniques. This fact remains troubling, since similar autocorrelations of any two brain regions do not necessarily result from their actual structural connectivity or functional correlation.

The study has been recently published [6].

**Acknowledgements:** Work supported by the National Science Centre (Poland) grant DEC-2015/17/D/ST2/03492 (JKO), Polish Ministry of Science and Higher Education ”Diamond Grant” 0225/DIA/2015/44 (WT), and by CONICET (Argentina) and Escuela de Ciencia y Tecnología, UNSAM (DRC).

**References**

1. Hindriks R, Adhikari MH, Murayama Y et al. Can sliding-window correlations reveal dynamic functional connectivity in resting-state fMRI? *Neuroimage* 2016, 127, 242–256.
2. Thompson WH, Richter CG, Plavén-Sigray P, Fransson P. Simulations to benchmark time-varying connectivity methods for fMRI. *PLoS Computational Biology* 2018, 14, e1006196.
3. Shine JM, Koyejo O, Bell PT, et al. Estimation of dynamic functional connectivity using multiplication of temporal derivatives. *Neuroimage* 2015, 122, 399–407.
4. Laumann TO, Snyder AZ, Mitra A. et al. On the stability of BOLD fMRI correlations. *Cereb Cortex* 2017, 27, 4719–4732.
5. Smith SM, Miller KL, Salimi-Khorshidi G. et al. Network modelling methods for FMRI. *Neuroimage* 2011, 54, 875–891.
6. Ochab JK, Tarnowski W, Nowak MA, Chialvo DR. On the pros and cons of using temporal derivatives to assess brain functional connectivity. *Neuroimage* 2019, 184, 577–585.

## P37 nigeLab: a fully featured open source neurophysiological data analysis toolbox

### Federico Barban^1^, Maxwell D. Murphy^2^, Stefano Buccelli^1^, Michela Chiappalone^1^

#### ^1^Fondazione Istituto Italiano di Tecnologia, Rehab Technologies, IIT-INAIL Lab, Genova, Italy; ^2^University of Kansas Medical Center, Department of Physical Medicine and Rehabilitation, Kansas City, United States of America

##### **Correspondence:** Federico Barban (federico.barban@iit.it)

*BMC Neuroscience* 2019, **20(Suppl 1)**:P37

The rapid advance in neuroscience research and the related technological improvements have led to an exponential increase in the ability to collect high-density neurophysiological signals from extracellular field potentials generated by neurons. While the specific processing of these signals is dependent upon the nature of the system under consideration, many studies seek to relate these signals to behavioral or sensory stimuli and typically follow a similar workflow. In this context we felt the need for a tool that facilitates tracking and organizing data across experiments and experimental groups during the processing steps. Moreover, we sought to unify different resources into a single hub that could offer standardization and interoperability between different platforms, boosting productivity and fostering the open exchange of experimental data between collaborating groups.

To achieve this, we built an end-to-end signal analysis package based on MATLAB, with a strong focus on collaboration, organization and data sharing . Inspired by the FAIR data policy [1], we propose a hierarchical data organization with copious amount of metadata, to help keep everything organized, easily shareable and traceable. The result is the *neuroscience integrated general electrophysiology lab*, or nigeLab, a unified package for tracking and analyzing electrophysiological and behavioral endpoints in neuroscientific experiments. The pipeline has a lot to offer: data extraction to a standard hierarchical format, filtering algorithms with local field potential (LFP) extraction, spike detection and spike sorting, point process analysis, frequency content analysis, graph theory and connectivity analysis both in the spike domain and in the LFP as well as many data visualizations tools and interfaces.

The source code is freely available and developed to be easily expandable and adaptable to different setups and paradigms. Importantly, nigeLab focuses on ease-of-use through an intuitive interface. We aimed to design an easily deployable toolkit for scientists with a non-technical background, while still offering powerful tools for electrophysiological pre-processing, analysis, and metadata tracking. The whole pipeline is lightweight and optimized to be scalable and parallelizable and can be run on a laptop as well as on a cluster.

**Reference**

1. European Commission. Guidelines on FAIR Data Management in Horizon 2020.

## P38 Neural ensemble circuits with adaptive resonance frequency

### Alejandro Tabas^1^, Shih-Cheng Chien^2^

#### ^1^Max Planck Institute for Human Cognitive and Brain Sciences, Research Group in Neural Mechanisms of Human Communication, Leipzig, Germany; ^2^Max Planck Institute for Human Cognitive and Brain Sciences, Leipzig, Germany

##### **Correspondence:** Alejandro Tabas (alextabas@gmail.com)

*BMC Neuroscience* 2019, **20(Suppl 1)**:P38

Frequency modulation is a ubiquitous phenomenon in sensory processing and cortical communication. Although multiple neural mechanisms are known to operate at cortical and subcortical levels of the auditory hierarchy to encode fast FM-modulation, the neural encoding of low-rate FM-modulation are still poorly understood. In this work, we introduce a potential neural mechanism for low-rate FM selectivity based on a simplified model of a cortical microcolumn following Wilson-Cowan dynamics.

Previous studies have used Wilson-Cowan microcircuits with one excitatory and one inhibitory population to build a system responding selectively to certain rhythms [1]. The excitatory ensemble is connected to the circuit’s input, that usually consists of a sinusoid or a similarly periodic input. The system incorporates synaptic depression through adaptation variables that reduce the effective connectivity weights between the neural populations [2]. By carefully tuning the system parameters, May and Tiitinen showed that this system shows resonant behaviour to a narrow range of frequencies of the oscillatory input, effectively acting as a periodicity detector [1].

Here, we first provide for an approximate analytical expression of the resonance frequency of the system with the system parameters. First, we subdivide the Wilson-Cowan dynamics in two dynamical systems operating at two different temporal scales: the fast system, that operates at the timescale of the cell membrane time constants (tau ~ 10-20ms [3]), and the slow system, that operates at the timescale of the adaptation time constant (tau = 500ms [2]). In the timescale of the fast system, the adaptation dynamics are quasistatic and the connectivity weights can be regarded as locally constant. Under these conditions, we show that the Wilson-Cowan microcircuit behaves as a driven damped harmonic oscillator whose damping factor and resonant frequency depend on the connectivity weights between the populations. We validate the analytical predictions with numerical simulations of the non-approximated system with different sinusoidal inputs and show that our analytical predictions explain the previous results from May and Tiitinen [1].

In the timescale of the slow system, fast oscillations in the firing rate of the excitatory and inhibitory populations are smoothed down by the effective low-pass filtering exerted by the much slower adaptation dynamics. Under these conditions, the connectivity weights decay slowly at a constant rate that depends on the average firing rates of the neural populations and the adaptation strengths. However, since the nominal resonance frequency depends on the connectivity weights, the decay of the latter results in a modulation of the former. We exploit this property to build a series of architectures that potentially show direction selectivity to rising or falling frequency modulated sinusoids. Our analytical predictions are validated by numerical simulations of the non-approximated system, driven by frequency modulated sinusoidal inputs.

**References**

1. May P, Tiitinen H. Human cortical processing of auditory events over time. *NeuroReport* 2001 Mar 5;12(3):573–7.
2. May P, Tiitinen H. Temporal binding of sound emerges out of anatomical structure and synaptic dynamics of auditory cortex. *Frontiers in computational neuroscience* 2013 Nov 7;7:152.
3. McCormick DA, Connors BW, Lighthall JW, Prince DA. Comparative electrophysiology of pyramidal and sparsely spiny stellate neurons of the neocortex. *Journal of neurophysiology* 1985 Oct 1;54(4):782–806.

## P39 Large-scale cortical modes reorganize between infant sleep states and predict preterm development

### James Roberts^1^, Anton Tokariev^2^, Andrew Zalesky^3^, Xuelong Zhao^4^, Sampsa Vanhatalo^2^, Michael Breakspear^5^, Luca Cocchi^6^

#### ^1^QIMR Berghofer Medical Research Institute, Brain Modelling Group, Brisbane, Australia; ^2^University of Helsinki, Department of Clinical Neurophysiology, Helsinki, Finland; ^3^University of Melbourne, Melbourne Neuropsychiatry Centre, Melbourne, Australia; ^4^University of Pennsylvania, Department of Neuroscience, Philadelphia, United States of America; ^5^QIMR Berghofer Medical Research Institute, Systems Neuroscience Group, Brisbane, Australia; ^6^QIMR Berghofer Medical Research Institute, Clinical Brain Networks Group, Brisbane, Australia

##### **Correspondence:** James Roberts (james.roberts@qimrberghofer.edu.au)

*BMC Neuroscience* 2019, **20(Suppl 1)**:P39

Sleep architecture carries important information about brain health but mechanisms at the cortical scale remain incompletely understood. This is particularly so in infants, where there are two main sleep states: active sleep and quiet sleep, precursors to the adult REM and NREM. Here we show that active compared to quiet sleep in infants heralds a marked change from long- to short-range functional connectivity across broad-frequency neural activity. This change in cortical connectivity is attenuated following preterm birth and predicts visual performance at two years. Using eigenmodes of brain activity [1] derived from neural field theory [2], we show that active sleep primarily exhibits reduced energy in a large-scale, uniform mode of neural activity and slightly increased energy in two non-uniform anteroposterior modes. This energy redistribution leads to the emergence of more complex connectivity patterns in active sleep compared to quiet sleep. Preterm-born infants show an attenuation in this sleep-related reorganization of connectivity that carries novel prognostic information. We thus provide a mechanism for the observed changes in functional connectivity between sleep states, with potential clinical relevance.

**Acknowledgments:** A.T. was supported by Finnish Cultural Foundation (Suomen Kulttuurirahasto; 00161034). A.T. and S.V. were also funded by Academy of Finland (276523 and 288220) and Sigrid Jusélius Foundation (Sigrid Juséliuksen Säätiö), as well as Finnish Pediatric Foundation (Lastentautien tutkimussäätiö). J.R., A.Z., M.B., and L.C. are supported by the Australian National Health Medical Research Council (J.R. 1144936 and 1145168, A.Z. 1047648, M.B. 1037196, L.C. 1099082 and 1138711). This work was also supported by the Rebecca L. Cooper Foundation (J.R., PG2018109) and the Australian Research Council Centre of Excellence for Integrative Brain Function (M.B., CE140100007).

**References**

1. Atasoy S, Donnelly I, Pearson J. Human brain networks function in connectome-specific harmonic waves. *Nature communications* 2016 Jan 21;7:10340.
2. Robinson PA, Zhao X, Aquino KM, Griffiths JD, Sarkar S, Mehta-Pandejee G. Eigenmodes of brain activity: Neural field theory predictions and comparison with experiment. *NeuroImage* 2016 Nov 15;142:79–98.

## P40 Reliable information processing through self-organizing synfire chains

### Thomas Ilett, David Hogg, Netta Cohen

#### University of Leeds, School of Computing, Leeds, United Kingdom

##### **Correspondence:** Thomas Ilett (tomilett@gmail.com)

*BMC Neuroscience* 2019, **20(Suppl 1)**:P40

Reliable information processing in the brain requires precise transmission of signals across large neuron populations that is reproducible and stable over time. Exactly how this is achieved remains an open question but a large body of experimental data has pointed to the importance of synchronised firing patterns of cell assemblies in mediating precise sequential patterns of activity. Synfire chains provide an appealing theoretical framework to account for reliable transmission of information through a network, with potential for robustness to noise and synaptic degradation. Here, we use self-assembled synfire chain models to test the interplay between encoding capacity, robustness to noise and flexibility to learning new patterns. We first model synfire chain development as a self-assembly process from a randomly connected network of leaky integrate-and-fire (LIF) neurons subject to a variant of the spike-timing-dependent plasticity (STDP) learning rule (adapted from [1]). We show conditions for these networks to form chains (in some conditions even without external input) and characterise the encoding capacity of the network by presenting different input patterns that result in distinguishable chains of activation. We show that these networks develop different, often overlapping chains in response to different inputs. We further demonstrate the importance of inhibition for the long-term stability of the chains and test the robustness of our network to various degrees of neuronal and synaptic death. Finally, we explore the ability for the network to increase its encoding capacity by dynamically learning new inputs.

**Reference**

1. Waddington A, Appleby PA, De Kamps M, Cohen N. Triphasic spike-timing-dependent plasticity organizes networks to produce robust sequences of neural activity. *Frontiers in Computational Neuroscience* 2012 Nov 12;6:88.

## P41 Acetylcholine regulates redistribution of synaptic efficacy in neocortical microcircuitry

### Cristina Colangelo

#### Blue Brain Project (BBP), Brain Mind Institute, EPFL, Lausanne, Switzerland, geneva, Switzerland

##### **Correspondence:** Cristina Colangelo (cristina.colangelo@epfl.ch)

*BMC Neuroscience* 2019, **20(Suppl 1)**:P41

Acetylcholine is one of the most widely characterized neuromodulatory systems involved in the regulation of cortical activity. Cholinergic release from the basal forebrain controls neocortical network activity and shapes behavioral states such as learning and memory. However, a precise understanding of how acetylcholine regulates local cellular physiology and synaptic transmission that reconfigure global brain states remains poorly understood. To fill this knowledge gap, we analyzed whole-cell patch-clamp recordings from connected pairs of neocortical neurons to investigate how acetylcholine release modulates membrane properties and synaptic transmission. We found that bath-application of 10 µM carbachol differentially redistributes the available synaptic efficacy and the short-term dynamics of excitatory and inhibitory connections. We propose that redistribution of synaptic efficacy by acetylcholine is a potential means to alter content, rather than the gain of information transfer of synaptic connections between specific cell-types types in the neocortex. Additionally, we provide a dataset that can serve as reference to build data-driven computational models on the role of ACh in governing brain states.

## P42 NeuroGym: A framework for training any model on more than 50 neuroscience paradigms

### Manuel Molano-Mazon^1^, Guangyu Robert Yang^2^, Christopher Cueva^2^, Jaime de la Rocha^1^, Albert Compte^3^

#### ^1^IDIBAPS, Theoretical Neurobiology, Barcelona, Spain; ^2^Columbia University, Center for Theoretical Neuroscience, New York, United States of America; ^3^IDIBAPS, Systems Neuroscience, Barcelona, Spain

##### **Correspondence:** Manuel Molano-Mazon (molano@clinic.cat)

*BMC Neuroscience* 2019, **20(Suppl 1)**:P42

It is becoming increasingly popular in systems neuroscience to train Artificial Neural Networks (ANNs) to investigate the neural mechanisms that allow animals to display complex behavior. Important aspects of brain function such as perception or working memory [2, 4] have been investigated using this approach, which has yielded new hypotheses about the computational strategies used by brain circuits to solve different behavioral tasks.

While ANNs are usually tuned for a small set of closely related tasks, the ultimate goal when training neural networks must be to find a model that can explain a wide range of experimental results collected across many different tasks. A necessary step towards that goal is to develop a large, standardized set of neuroscience tasks on which different models can be trained. Indeed, there is a large body of experimental work that hinges on a number of canonical behavioral tasks that have become a reference in the field (e.g. [2,4]) and that makes it possible to develop a general framework encompassing many relevant tasks on which neural networks can be trained.

Here we propose a comprehensive toolkit, NeuroGym, that allows training any network model on many established neuroscience tasks using Reinforcement Learning techniques. NeuroGym currently contains more than ten classical behavioral tasks including, working memory tasks (e.g. [4]), value-based decision tasks (e.g. [3]) and context-dependent perceptual categorization tasks (e.g. [2]). In providing this toolbox our aim is twofold: (1) to facilitate the evaluation of any network model on many tasks and thus evaluate its capacity to generalize to and explain different experimental datasets; (2) to standardize the way computational neuroscientists implement behavioral tasks, in order to promote benchmarking and replication.

Inheriting all functionalities from the machine learning toolkit Gym (OpenAI), NeuroGym allows a wide range of well-established machine learning algorithms to be easily trained on behavioral paradigms relevant for the neuroscience community. NeuroGym also incorporates several properties and functions (e.g. realistic time step or separation of training into trials) that are specific to the protocols used in neuroscience.

Furthermore, the toolkit includes various modifier functions that greatly expand the space of available tasks. For instance, users can introduce trial-to-trial correlations onto any task [1]. Also, tasks can be combined so as to test the capacity of a given model to perform two tasks simultaneously (e.g. to study interference between two tasks [5]).

In summary, NeuroGym constitutes an easy-to-use toolkit that considerably facilitates the evaluation of a network model that has been tuned for a particular task on more than 50 tasks with no additional work, and proposes a framework to which computational neuroscience practitioners can contribute by adding tasks of their interest, using a straightforward template.

**Acknowledgments:** The Spanish Ministry of Science, Innovation and Universities, the European Regional Development Fund (grant BFU2015-65315-R), by Generalitat de Catalunya (grants 2017 SGR 1565 and 2017-BP-00305) and the European Research Council (ERC-2015-CoG–683209_PRIORS).

**References**

1. Hermoso-Mendizabal A, Hyafil A, Rueda-Orozco PE, Jaramillo S, Robbe D, de la Rocha J. Response outcomes gate the impact of expectations on perceptual decisions. *bioRxiv* 2019 Jan 1:433409.
2. Mante V, Sussillo D, Shenoy KV, Newsome WT. Context-dependent computation by recurrent dynamics in prefrontal cortex. *Nature* 2013 Nov;503(7474):78.
3. Padoa-Schioppa C, Assad JA. Neurons in the orbitofrontal cortex encode economic value. *Nature* 2006 May;441(7090):223.
4. Romo R, Brody CD, Hernández A, Lemus L. Neuronal correlates of parametric working memory in the prefrontal cortex. *Nature* 1999 Jun;399(6735):470.
5. Zhang X, Yan W, Wang W, et al. Active information maintenance in working memory by a sensory cortex. *bioRxiv* 2018 Jan 1:385393.

## P43 Synaptic dysfunctions underlying reduced working memory serial bias in autoimmune encephalitis and schizophrenia

### Heike Stein^1^, Joao Barbosa^1^, Adrià Galán^1^, Alba Morato^2^, Laia Prades^2^, Mireia Rosa^3^, Eugenia Martínez^4^, Helena Ariño^4^, Josep Dalmau^4^, Albert Compte^3^

#### ^1^Institut d’Investigacions Biomèdiques August Pi i Sunyer (^2^IDIBAPS), Theoretical Neurobiology, Barcelona, Spain; ^2^IDIBAPS, Neuroscience, Barcelona, Spain; ^3^Hospital Clinic, Pediatric Psychiatry, Barcelona, Spain; ^4^IDIBAPS, Neuroimmunology, Barcelona, Spain

##### **Correspondence:** Heike Stein (heike.c.stein@gmail.com)

*BMC Neuroscience* 2019, **20(Suppl 1)**:P43

Continuity of mnemonic contents in time contributes to forming coherent memory representations. Recently, attractive response biases towards previously memorized features in delayed-response tasks have been reported as evidence for the continuous integration of working memory (WM) contents between trials [1]. In turn, brain disorders with reported executive and memory dysfunction may be characterized by reduced WM serial bias [2], revealing reduced temporal coherence of memory representations. To gain mechanistic insight into this effect, we tested a unique population of patients recovering from anti-NMDAR encephalitis patients, an immune-mediated brain disease causing a drastic reduction of NMDARs, accompanied by WM deficits even as receptors return to normal levels [3]. We hypothesized that potential changes in serial biases found in anti-NMDAR encephalitis should be qualitatively similar to changes in schizophrenia, a disorder associated with hypofunctional NMDARs. We collected behavioral data from anti-NMDAR encephalitis patients, schizophrenic patients, and healthy controls performing a visuospatial WM task. While healthy controls’ responses were significantly biased towards previously remembered locations in the presence of WM requirements (delays of several seconds), attractive serial biases were reduced in encephalitis, and absent in schizophrenic patients. We modeled these findings using a recurrent spiking network with synaptic short-term facilitation in excitatory connections. In this model, memory-sustaining bumps of persistent activity decay after the memory delay but leave stimulus-specific, facilitated synaptic ‘traces’ that affect neural dynamics in the next trial. We systematically explored parameters of synaptic transmission and short-term plasticity to determine the mechanism that could reduce attractive serial bias. By altering the parameters of short-term facilitation, we reproduced reduced and absent attractive biases in patient groups, while maintaining WM precision at a constant level across groups, an intriguing finding from our behavioral analyses. This manipulation of short-term facilitation is in accordance with studies in cortical slices from mouse models of schizophrenia [4]. We thus propose that serial biases in visuospatial WM provide a behavioral readout of short-term facilitation dysfunction in anti-NMDAR encephalitis and schizophrenia.

**Acknowledgements:** Funding provided by Institute Carlos III, Spain (grant PIE 16/00014), Cellex Foundation, the Spanish Ministry of Science, Innovation and Universities (grant BFU 2015-65318-R), the European Regional Development Fund, the Generalitat de Catalunya (grant AGAUR 2017 SGR 1565), “la Caixa” (LCF/BQ/IN17/11620008, H.S.), and the European Union’s Horizon 2020 Marie Skłodowska-Curie grant (713673, H.S.).

**References**

1. Fischer J, Whitney D. Serial dependence in visual perception. *Nature Neuroscience* 2014, 17, 738–743
2. Lieder I, Adam V, Frenkel O, et al. Perceptual bias reveals slow-updating in autism and fast-forgetting in dyslexia. *Nature Neuroscience* 2019, 22, 256–264
3. Dalmau J, Lancaster E, Martinez-Hernandez E, et al. Clinical experience and laboratory investigations in patients with anti-NMDAR encephalitis. *Lancet Neurology* 2011, 10, 63–74
4. Arguello P, Gogos J. Genetic and cognitive windows into circuit mechanisms of psychiatric disease. *Trends in Neuroscience* 2012, 35, 3–13

## P44 Effects of heterogeneity in neuronal electric properties on the intrinsic dynamics of cortical networks

### Svetlana Gladycheva^1^, David Boothe^2^, Alfred Yu^2^, Kelvin Oie^2^, Athena Claudio^1^, Bailey Conrad^1^

#### ^1^Towson University, Department of Physics, Astronomy and Geosciences, Towson, MD, United States of America; ^2^U.S. Army Research Laboratory, Human Research and Engineering Directorate, Aberdeen Proving Ground, MD, United States of America

##### **Correspondence:** Svetlana Gladycheva (sgladycheva@towson.edu)

*BMC Neuroscience* 2019, **20(Suppl 1)**:P44

In previous large-scale models of neural systems, neurons of the same class are typically identical. By contrast, real systems exhibit significant cell-to-cell diversity at different levels, from morphology to intrinsic cell properties [1] to synaptic properties [2]. This heterogeneity may affect neural information processing by, for example, helping to integrate diverse inputs to the network [1], or by positively contributing to the stability of the network activity [3]. However, the exact role of neural heterogeneity in large-scale neural systems is not fully understood.

We examine the impact of neural heterogeneity in large-scale neural models. We use an adaptation of the Traub’s single-column thalamocortical network model [4], adapted to the PGENESIS parallel simulation environment [5]. The model is tuned to eliminate intrinsic neuronal activity and is randomly driven with independent Poisson-distributed excitatory postsynaptic noise potentials with an average firing rate between 1-10 Hz.

Network activity is assessed by calculating the mean local field potential (LFP) and analyzing the neuronal spiking activity. We explored changes in network parameters, including local connectivity probability; the parameters of the noise inputs; and the relative strength of synaptic weights. Observed LFPs can generally be classified into two patterns: an aperiodic low-activity state and a high-activity state involving persistent oscillations associated with periodic neuronal firing. At a broad range of the connectivity probabilities, the network stays in low-activity state until a “threshold” level of connectivity is reached. Further increase in connectivity moves model behavior into high-activity regimes and alters the frequency spectrum. Changes in parameters of noise inputs (frequency range, weight, and percentage of neurons receiving noise) elicit similar threshold-like behavior, as do changes in the ratio of excitatory-to-inhibitory synaptic weights, with high-activity states observed in networks with weak inhibition. We introduce heterogeneity in the intrinsic biophysical parameters by randomizing the values of the anomalous rectifier (AR) channels’ conductance in the model’s pyramidal neurons. Preliminary results from effects of heterogeneity on network activity will be shown. In addition, network responses to pulse train stimuli input to the pyramidal cells at different locations in the column will be studied.

**References**

1. Adams NE, et al. Heterogeneity in neuronal intrinsic properties: a possible mechanism for hub-like properties of the rat anterior cingulate cortex during network activity. *eNeuro* 2017, 0313–16
2. Thomson AM, et al, Single axon IPSPs elicited in pyramidal cells by three classes of interneurons in slices of rat neocortex. *Journal of Physiology* 1996, 496:81–102
3. Mejias JF, Longtin A, Differential effects of excitatory and inhibitory heterogeneity on the gain and asynchronous state of sparse cortical networks. *Frontiers in Computational Neuroscience* 2014, 8:107
4. Traub RD, et al, Single column thalamocortical network model exhibiting gamma oscillations, sleep spindles and epileptic bursts. *Journal of Neurophysiology* 2005, 93(4):2194–232
5. Boothe, et al, Impact of neuronal membrane damage on a local field potential in a large-scale simulation of the neuronal cortex. *Frontiers in Neurology* 2017, 8:236

## P45 Structure–function multi-scale connectomics reveals a major role of the fronto-striato-thalamic circuit in brain aging

### Paolo Bonifazi^1^, Asier Erramuzpe^1^, Ibai Diez^1^, Iñigo Gabilondo^1^, Matthieu Boisgontier^2^, Lisa Pauwels^2^, Sebastiano Stramaglia^3^, Stephan Swinnen^2^, Jesus Cortes^1^

#### ^1^Biocruces Health Research Institute, Computational Neuroimaging, Barakaldo, Spain; ^2^Katholieke Universiteit Leuven, Department of Movement Sciences, Leuven, Belgium; ^3^University of Bari, Physics, Bari, Italy

##### **Correspondence:** Paolo Bonifazi (paol.bonifazi@gmail.com)

*BMC Neuroscience* 2019, **20(Suppl 1)**:P45

Physiological aging affects brain structure and function impacting morphology, connectivity, and performance. However, whether some brain connectivity metrics might reflect the age of an individual is still unclear. Here, we collected brain images from healthy participants (N = 155) ranging from 10 to 80 years to build functional (resting state) and structural (tractography) connectivity matrices, both data sets combined to obtain different connectivity features. We then calculated the brain connectome age—an age estimator resulting from a multi-scale methodology applied to the structure–function connectome, and compared it to the chronological age (ChA). Our results were twofold. First, we found that aging widely affects the connectivity of multiple structures, such as anterior cingulate and medial prefrontal cortices, basal ganglia, thalamus, insula, cingulum, hippocampus, parahippocampus, occipital cortex, fusiform, precuneus, and temporal pole. Second, we found that the connectivity between basal ganglia and thalamus to frontal areas, also known as the fronto-striato-thalamic (FST) circuit, makes the major contribution to age estimation. In conclusion, our results highlight the key role played by the FST circuit in the process of healthy aging. Notably, the same methodology can be generally applied to identify the structural–functional connectivity patterns correlating to other biomarkers than ChA.

## P46 Studying evoked potentials in large cortical networks with PGENESIS 2.4

### David Beeman^1^, Alfred Yu^2^, Joshua Crone^3^

#### ^1^University of Colorado, Department of Electrical, Computer and Energy Engineering, Boulder, CO, United States of America; ^2^U.S. Army Research Laboratory, Human Research and Engineering Directorate, Aberdeen Proving Ground, MD, MD, United States of America; ^3^U.S. Army Research Laboratory, Computational and Information Sciences Directorate, Aberdeen Proving Ground, MD, MD, United States of America

##### **Correspondence:** David Beeman (dbeeman@colorado.edu)

*BMC Neuroscience* 2019, **20(Suppl 1)**:P46

Modern neural simulators have been developed for large scale network models of single-compartment integrate-and-fire neurons that efficiently model millions of neurons. However, accurate modeling of neural activity, including evoked potentials (EPs) recorded from scalp or cortical surface electrodes, requires multicompartmental neuron models with enough realism in the dendritic morphology and location of synapses to account for the major sinks and sources of currents in the extracellular medium. The GENESIS simulator (http://genesis-sim.org) and its parallel computer version PGENESIS were developed over 30 years ago for structurally realistic modeling of large cortical networks. Today, GENESIS continues to be updated with new features and used for implementing such models. Recently Kudela, et al. [1] used a large GENESIS network model to study effects of short-term synaptic plasticity on adaptation of EPs in auditory cortex. Our plans are to increase the size and cell density, extend the model to other cortical layers, and to run simulations on supercomputers such as those available through NSG (the Neuroscience Gateway portal, https://www.nsgportal.org) [2]. Crone, et al. [3] have modified the 2006 release of GENESIS and PGENESIS 2.3 to allow simulations of networks of up to 9 million neurons. Their modifications addressed memory management, reproducibility, and other issues that limited model scalability on high performance computing resources. These improvements are now merged with the current GENESIS/PGENESIS 2.4 development versions. This official release of PGENESIS 2.4 and GENESIS 2.4 are available from the Repository for Continued Development of the GENESIS 2.4 Neural Simulator (https://github.com/genesis-sim/genesis-2.4). We used the new PGENESIS to simulate EPs measured 2 mm above a patch of layer 2/3 primary auditory cortex (Fig. 1), as in [1]. The network was divided into 24 slices simulated in parallel. This model uses 17-compartment pyramidal cells (PCs) based on human cortical PC reconstructions. Inhibition is provided from model basket cells (BCs). Short tone pulses produce excitation to PC distal basal dendrites. Subsequently, PC-PC excitation occurs at oblique apical dendrites. It was shown in [1] that these two excitatory currents produce oppositely oriented electric dipolar charges that are responsible for the initial vertex-positive P1 peak and the following vertex-negative N1 peak in the EP. These results show the effect of varying the strength of the inhibition at the PC proximal apical dendrite from BCs. This occurs later in the N1 peak, and produces a dipole that is oriented oppositely to the one that causes the N1 peak. Therefore, increased inhibition narrows the peak. With PGENESIS available on NSG and other supercomputer resources, we can foster collaborations for using realistic network models to understand human cortical activity.

**Fig. 1:**
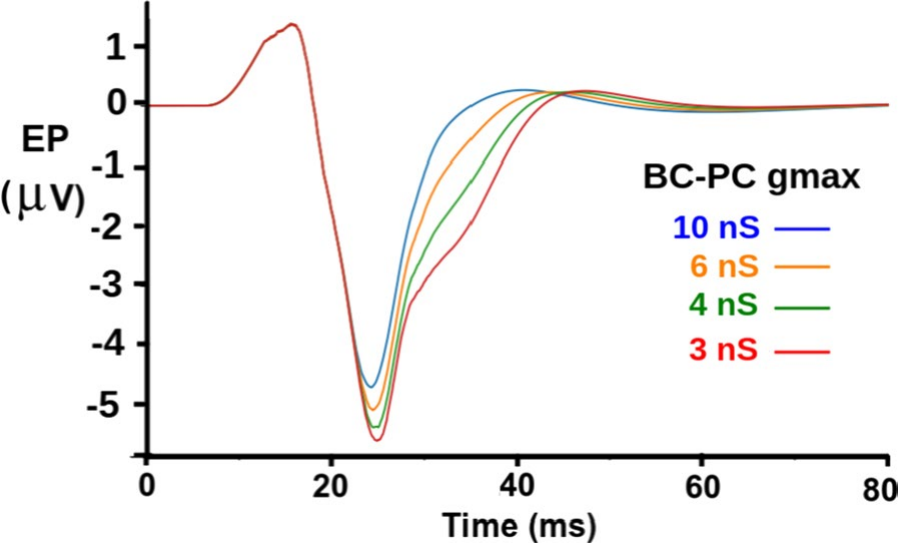
Trial-averaged EPs for the parallel network model, with varying PC maximal inhibitory conductances gmax

**References**

1. Kudela P, Boatman-Reich D, Beeman D and Anderson WS. Modeling Neural Adaptation in Auditory Cortex. *Front. Neural Circuits* 2018, 05 Sept. 10.3389/fncir.2018.00072.
2. Sivagnanam S, Majumdar A, Yoshimoto K, Astakhov V, Bandrowski A, Martone ME, Carnevale NT. Introducing the Neuroscience Gateway. *IWSG, volume 993 of CEUR Workshop Proceedings* 2013 CEUR-WS.org.
3. Crone J, Boothe D, Yu A, Olie K, Franaszczuk P. Time step sensitivity in large scale compartmental models of the neocortex. *BMC Neurosci* 2018 19(Suppl 2):P184.

## P47 Automated assessment and comparison of cortical neuron models

### Justas Birgiolas^1^, Russell Jarvis^1^, Vergil Haynes^2^, Richard Gerkin^1^, Sharon Crook^2^

#### ^1^Arizona State University, School of Life Sciences, Tempe, United States of America; ^2^Arizona State University, School of Mathematical and Statistical Sciences, Tempe, AZ, United States of America

##### **Correspondence:** Sharon Crook (sharon.crook@asu.edu)

*BMC Neuroscience* 2019, **20(Suppl 1)**:P47

Computational models are an indispensable tool for understanding the nervous system. However, describing, sharing, and re-using models with diverse components at many scales represents a major challenge in neuroscience. We have contributed to the development of the NeuroML model description standard [2] and the model sharing platform NeuroML-DB [1] to promote reproducibility and the re-use of data driven neuroscience models. We also have developed the SciDash framework for validating such models against experimental data [4] and sharing the validation outcomes for further scientific discovery at dash.scidash.org, increasing transparency and rigor in the field.

This infrastructure also supports automated pipelines for running large numbers of models shared in the NeuroML format at NeuroML-DB and characterization of these model neurons using simulated “experiments”. These experiments are based on the electrophysiology protocols used by the Allen Cell Type Database [3], which include square, long square, pink noise, ramp, short square and short square triple protocols, and are also the basis for model validation tests. Results are shared in interactive plots at NeuroML-DB. We have characterized over 1000 published cortical neuron models and used the electrophysiological properties of these cortical neuron models to cluster their dynamic behaviors and identify the biophysical properties of models that underlie these clusters. These properties are compared to similar results for experimentally-derived cortical neuron data, providing an overview of how well data-driven models represent the landscape of cortical neuron electrophysiology.

**Acknowledgments:** This research was funded in part by R01MH106674 from NIMH of the National Institutes of Health and R01EB021711 from NIBIB of the National Institutes of Health.

**References**

1. Birgiolas J, et al. Ontology-assisted keyword search for NeuroML models. In Amarnath Gupta and Susan Rathbun, editors. *Proceedings of the 27*^*th*^ *International Conference on Scientific and Statistical Database Management* 2015. New York, NY: ACM; article 37.
2. Gleeson P, et al. NeuroML: a language for describing data driven models of neurons and networks with a high degree of biological detail. *PLoS Computational Biology* 2010, 6, e1000815.
3. Hawrylycz M, et al. Inferring cortical function in the mouse visual system through large-scale systems neuroscience. *PNAS* 2016, 113(27), 7337–44.
4. Omar C, et al. Collaborative infrastructure for test-driven scientific model validation. *In Companion Proceedings of the 36th International Conference on Software Engineering* 2014 May 31 (pp. 524–527). ACM.

## P48 High dimensional ion channel composition enables robust and efficient targeting of realistic regions in the parameter landscape of neuron models

### Marius Schneider^1^, Peter Jedlicka^2^, Hermann Cuntz^3,4^

#### ^1^University of Frankfurt, Institute for Physics, Butzbach, Germany; ^2^Justus Liebig University, Faculty of Medicine, Giessen, Germany; ^3^Frankfurt Institute for Advanced Studies (FIAS), Frankfurt am Main, Germany; ^4^Ernst Strüngmann Institute (ESI), Computational Neuroanatomy, Frankfurt am Main, Germany

##### **Correspondence:** Marius Schneider (marius-s@online.de)

*BMC Neuroscience* 2019, **20(Suppl 1)**:P48

Cellular and molecular sources of variability in the electrical activity of nerve cells are not fully understood. An improved understanding of this variability is the key to predict the response of nerve tissue to pathological changes. We have previously created a robust data-driven compartmental model of the hippocampal granule cell comprising 16 different ion channels and variable dendritic morphologies. Here, we show that it is possible to drastically reduce ion channel diversity while preserving the characteristic spiking behavior of real granule cells. In order to better understand the variability in spiking activity we generated large populations of validated granule cell models with different numbers of ion channels. Unreduced or less reduced models with a higher number of ion channels covered larger and more widely spread regions of the parameter landscape. Moreover, unreduced or less reduced models with a higher number of ion channels were more stable in the face of parameter perturbations. This suggests that ion channel diversity allows for increased robustness and higher flexibility of finding a solution in the complex parameter space. In addition to increasing our understanding of cell-to-cell variability, our models might be of practical relevance. Instead of a one-size-fits-all approach where a computer model simulates average experimental values, the population-based approach reflects the variability of experimental data and therefore might enable pharmacological studies *in silico*.

## P49 Modelling brain folding using neuronal placement according to connectivity requirements

### Moritz Groden^1^, Marvin Weigand^2,3^, Jochen Triesch^3^, Peter Jedlicka^4^, Hermann Cuntz^2,3^

#### ^1^Justus Liebig University Giessen, Faculty of Medicine, Mannheim, Germany; ^2^Ernst Strüngmann Institute (ESI), Computational Neuroanatomy, Frankfurt am Main, Germany; ^3^Frankfurt Institute for Advanced Studies (FIAS), Neuroscience, Frankfurt am Main, Germany; ^4^Institute of Clinical Neuroanatomy Frankfurt, ICAR3R-Justus-Liebig University Giessen, Faculty of Medicine, Giessen, Germany

##### **Correspondence:** Moritz Groden (moritzgromail@gmail.com)

*BMC Neuroscience* 2019, **20(Suppl 1)**:P49

Among different species of animals, the layout of the central nervous system varies extensively from individual clusters of neurons (ganglia) in invertebrates such as worms to solid brains found in mammals that typically exhibit increased folding the larger the animal. Such variations in layout may point to elemental differences in organization of circuitry and connectivity. However, many studies suggest that folding of the brain is a consequence of the restricted volume of the skull exerting mechanical forces on the cortex, which in turn folds to fit a larger surface area into such a confined cavity. In our study we consider a computational model that uses dimension reduction methods to ensure optimal placement of neurons, placing them according to connectivity needs, rather than modelling the forces exerted on the cortex. We assume a simple connectivity that features strong local but weak global (long-range) connections, which mimics the connectivity found in mammalian brains. The predictions made by our model cover all different phenotypes of brains found in animals, ranging from individual ganglia through smooth brains with no gyrification, to extremely convoluted brains for increasing cortical size. Many properties of the cortical morphology found in animals are reproduced by the model, which includes metrics such as the folding index and the fractal dimension. Our model presents a way to combine microscopic inter cellular connectivity with macroscopic morphologies into large-scale brain models that feature its neural network requirements.

## P50 Dynamic neural field modeling of auditory categorization tasks

### Pake Melland^1^, Bob McMurray^2^, Rodica Curtu^1^

#### ^1^University of Iowa, Department of Mathematics, Iowa City, United States of America; ^2^University of Iowa, Psychological and Brain Sciences, Iowa City, United States of America

##### **Correspondence:** Rodica Curtu (rodica-curtu@uiowa.edu)

*BMC Neuroscience* 2019, **20(Suppl 1)**:P50

Categorization is the fundamental ability to treat distinct stimuli similarly; categorization applied to auditory stimuli is crucial for speech perception. For example, phonemes like “t” and “d” are categories that generalize across speakers and contexts. A fundamental question asks what mechanisms form the foundation for auditory category learning. We propose a dynamic neural network framework that combines plausible biological mechanisms and the theory of dynamic neural fields to model this process. The network models a task designed to emulate first language acquisition—a period of unsupervised learning followed by supervised learning. In the unsupervised phase the listener is presented with a sequence of pairs of tones; each pair corresponds to one of four categories defined by their frequencies. During this time the subject engages in a non-distracting task. Then the subject engages in a supervised task and is instructed to associate each tone-pair with a physical object representing one of the four auditory categories. Corrective feedback is given to the subject during the supervised learning. The mathematical model is used to manipulate mechanisms through which hypotheses can be made about the category learning process. We present preliminary results from model simulations of the experiment and compare them with implementations of the experiment on human subjects.

Network Description and Results. We propose a dynamic neural field composed of multiple layers allowing for the manipulation and testing of multiple locations of plasticity involved in the learning process. First, incoming sounds stimulate a one-dimensional tonotopically organized feature space composed of neural units that interact through local excitation with lateral inhibition. Units along this space are associated with sub-cortical auditory fields and respond to specific frequencies in order to capture physical properties of the stimuli. Activity in the feature space feeds forward through excitatory connections, which undergo depression with prolonged stimuli encounters, to regions of primary and secondary auditory cortex. Activity in these regions provide input to the category layer of the network composed of 4 neural units corresponding to the 4 categories defined in the task. These nodes are hypothesized to represent regions in auditory-related temporal cortical regions such as superior temporal gyrus [2] and the inferior frontal gyrus in humans, or the prefrontal cortex in rats [1]. In the theoretical network, the four category nodes are coupled through mutual inhibition and compete in a winner take all setting. Above threshold activation peaks in the category layer are interpreted as experimentally detectable responses. In the supervised portion of the task, synapses between auditory cortex nodes and category layer nodes are updated with via Hebbian processes with a reward/punishment parameter that serves as corrective feedback to the network. Parameters within the model are tuned so that responses in the category layer closely match behavioral results obtained from implementations of the experiment on human subjects that varied stimuli distributions, category prototypes, and category boundaries. The model predicts category learning at rates consistent with those found experimentally.

**Acknowledgments:** NSF CRCNS 151567.

**References**

1. Francis NA, Winkowski DE, Sheikhattar A, Armengol K, Babadi B, Kanold PO. Small networks encode decision-making in primary auditory cortex. *Neuron* 2018 Feb 21;97(4):885–97.
2. Mesgarani N, Cheung C, Johnson K, Chang EF. Phonetic feature encoding in human superior temporal gyrus. *Science* 2014 Feb 28;343(6174):1006–10.

## P51 Role of TRP channels in temperature rate coding by drosophila noxious cold sensitive neurons

### Natalia Maksymchuk, Akira Sakurai, Atit Patel, Nathaniel Himmel, Daniel Cox, Gennady Cymbalyuk

#### Georgia State University, Neuroscience Institute, Atlanta, GA, United States of America

##### **Correspondence:** Natalia Maksymchuk (nmaksymchuk1@gsu.edu)

*BMC Neuroscience* 2019, **20(Suppl 1)**:P51

Noxious cold temperature can cause tissue damage and triggers protective behaviors of animals. Cellular mechanisms of noxious cold temperature coding are not well understood. We focus on *Drosophila* larval cold nociception capitalizing on a diverse array of approaches spanning genetics and animal behavior to electrophysiology and computational models. Larva responds to noxious cold by a well-characterized full-body contraction. Notably, this response is only triggered by a sufficiently fast temperature change. Class III (CIII) multidendritic sensory neurons and specific TRP channels are implicated in noxious cold temperature coding [1]. Based on Ca2+ imaging, specialized roles of Trpm and Pkd2 currents were established and our model explained an apparent paradox of these data [1, 2].

We performed electrophysiological recordings and Ca2+ imaging of CIII neurons along with behavioral analyses. We compared responses of wild type to slow and fast temperature changes from 24oC down to the 10oC. Cold-evoked contraction behavior was potentiated under fast ramping conditions relative to slow. Spiking and [Ca2+]i response at noxious cold were consistent with behavioral data. The CIII neurons exhibited a pronounced peak of spiking rate when the temperature was rapidly decreased and turned silent as the temperature was increased back to 24oC. The response was different when temperature changed slowly: the spiking rate was much smaller during the temperature decrease.

These results suggest that CIII neurons encode rate of temperature decrease. We hypothesize that inactivation processes of certain TRP channels could explain these differences. We focused on comparison of the roles of Pkd2 and Trpm currents as temperature sensors. Our computational model showed that the Ca2+-dependence of the Pkd2 inactivation constant could provide a mechanism of observed rate coding. This mechanism, implemented in the model, allowed us to reproduce recorded electrical activity data—high peak of firing rate in response to the rapid temperature change from 24oC to 10oC and silence during temperature return back to ambient levels. When the noxious cold temperature was held constant after fast ramp, Pkd2 channels inactivated, and low-frequency firing rate was supported through Trpm, responsible for coding temperature. This is consistent with behavioral data as well. In addition, the model shows that increased firing rate at fast temperature decline was accompanied by high [Ca2+]i level, whereas slow ramp resulted in significantly lower Ca2+. We conclude that certain TRP channels, such as Pkd2, could be responsible for high peak of firing rate at rapid temperature fall, whereas Trpm channels could encode the magnitude of temperature.

**Acknowledgements:** This work was supported by NIH R01 NS086082 and a GSU Brains & Behavior Seed Grant (DNC). NJH is a Brains and Behavior and Honeycutt Fellow; AAP is a 2CI Neurogenomics and Honeycutt Fellow.

**References**

1. Turner HN, Armengol K, Patel AA, et al. The TRP Channels Pkd2, NompC, and Trpm Act in Cold-Sensing Neurons to Mediate Unique Aversive Behaviors to Noxious Cold in Drosophila. *Current Biology* 2016, 26(23), 3116–3128.
2. Maksymchuk N, Patel AA, Himmel NJ, Cox DN, Cymbalyuk G. Modeling of TRP channel mediated noxious cold sensation in Drosophila sensory neurons. *BMC* *Neuroscience* 2018, 19(Suppl 2):64, 8–9.

## P52 Role of Na+/K+ pump in dopamine neuromodulation of a mammalian central pattern generator

### Alex Vargas, Gennady Cymbalyuk

#### Georgia State University, Neuroscience Institute, Atlanta, GA, United States of America

##### **Correspondence:** Alex Vargas (avarg453@gmail.com)

*BMC Neuroscience* 2019, **20(Suppl 1)**:P52

CPGs are oscillatory neuronal circuits controlling rhythmic movements across vertebrates and invertebrates [1]. The Na/K pump contributes to the dynamics of bursting activity in variety of CPGs seen across species such as leech, tadpole, and mouse [2, 3, 4, 5, 6]. Movements like locomotion and heartbeat must be continually regulated for an animal to meet environmental and behavioral demands [3]. In vertebrate CPGs, dopamine has been shown to induce a range of subtle to pronounced effects on locomotory and other motor rhythms. Dopamine neuromodulation affects Na/K Pump, GIRK2-, A-, and h-currents through D1 and D2 receptors [7, 8]; this contributes to stabilization of CPG rhythmic activity. We developed a half-center oscillator (HCO) model of a spinal locomotor CPG, which comprises of four populations, two inhibitory and two excitatory. Under a certain parameter regime, the neurons are intrinsically bursting, utilizing a persistent-sodium current mechanism. We investigated activity regimes of single endogenously bursting neurons and HCO. In a range of high modulation level, we found stable periodic bursting, while within some range of low dopamine modulation levels, pronounced intermittent intrinsic patterns. We investigated the hypothesis that dopamine affects the network through activation of inward rectifying potassium currents, IGIRK and IA, and opposing changes of h-current all while interacting with pump current. The reduction in modulatory level of dopamine in the spinal locomotor CPG causes the model to transition from normal periodic bursting into intermittent bursting and then to silence. Our locomotor CPG model highlights the role of the pump and its co-modulation along with GIRK2-, A-, and h-currents in production of robust rhythmic output.

**Acknowledgements:** Supported by NINDS 1 R21 NS111355 to GC.

**References**

1. Marder E, Calabrese RL. Principles of rhythmic motor pattern generation. *Physiological reviews* 1996 Jul 1;76(3):687–717.
2. Picton LD, Zhang H, Sillar KT. Sodium pump regulation of locomotor control circuits. *Journal of neurophysiology* 2017 May 24;118(2):1070–81.
3. Sharples SA, Whelan PJ. Modulation of rhythmic activity in mammalian spinal networks is dependent on excitability state. *eNeuro* 2017 Jan;4(1).
4. Sharples SA, Humphreys JM, Jensen AM, et al. Dopaminergic modulation of locomotor network activity in the neonatal mouse spinal cord. *Journal of neurophysiology* 2015 Feb 4;113(7):2500–10.
5. Kueh D, Barnett WH, Cymbalyuk GS, Calabrese RL. Na+/K+ pump interacts with the h-current to control bursting activity in central pattern generator neurons of leeches. *eLife* 2016 Sep 2;5:e19322.
6. Tobin AE, Calabrese RL. Myomodulin increases I h and inhibits the Na/K pump to modulate bursting in leech heart interneurons. J*ournal of neurophysiology* 2005 Dec;94(6):3938–50.
7. Sharples SA, Whelan PJ. Modulation of rhythmic activity in mammalian spinal networks is dependent on excitability state. *eNeuro* 2017 Jan;4(1).
8. Han P, Nakanishi ST, Tran MA, Whelan PJ. Dopaminergic modulation of spinal neuronal excitability. *Journal of Neuroscience* 2007 Nov 28;27(48):13192–204.

## P53 Hypoxic suppression of Ca2+-ATPase pumps and mitochondrial membrane potential eliminates rhythmic activity of simulated interstitial cells of Cajal

### Sergiy Korogod^1^, Iryna Kulagina^1^, Parker Ellingson^2^, Taylor Kahl^2^, Gennady Cymbalyuk^2^

#### ^1^Bogomoletz Institute of Physiology, National Academy of Sciences of Ukraine, Kiev, Ukraine; ^2^Georgia State University, Neuroscience Institute, Atlanta, GA, United States of America

##### **Correspondence:** Gennady Cymbalyuk (gcymbalyuk@gmail.com)

*BMC Neuroscience* 2019, **20(Suppl 1)**:P53

Neonatal hypoxic**-**ischemic injury is a risk factor for necrotizing enterocolitis (NEC), an inflammatory bowel disease that is often associated with failures of gastrointestinal motility. This motility is driven by a pacemaker action of the interstitial cells of Cajal (ICCs) on intestinal smooth muscle cells (SMCs). The ICC pacemaker activity is determined by interplay of Ca2+channels, pumps, and exchangers present in the endoplasmic reticulum (ER), mitochondria and plasma membrane to form a characteristic Ca2+-handling mechanism. Ca2+-ATPase pumps in ICC are potential targets for injuring action of hypoxia as they operate by consuming energy stored in ATP due to oxidative phosphorylation in mitochondria. In an ICC model, we mimicked effects of hypoxia by reduction of the mitochondrial bulk membrane potential (ΔΨ*) or maximal rates of Ca2+-ATPase pumps in the plasmalemma or ER (PMCA or SERCA, respectively). ICC pacemaker activity (oscillations of the plasma membrane potential Emand intracellular calcium concentration [Ca2+]i) ceased by individual suppression of ΔΨ*, or PMCA, or SERCA and the cessation scenarios were case-specific. Since naturally hypoxia simultaneously affects all these actors, in this study, we explored scenarios of cessation of ICC pacemaker activity depending on combined suppression of ΔΨ*, PMCA, and SERCA. At fixed normal ΔΨ*, equal joint suppression of PMCA and SERCA dramatically reduced amplitude of [Ca2+]iand Emoscillations to “downstate” levels near their basal/rest values. This was similar to the effect of individual suppression of SERCA and dissimilar to that of PMCA, which was characterized by very low-amplitude oscillations about “upstate” levels of depolarized Emand elevated [Ca2+]i. In each case, changes in oscillations frequency were insignificant. Same suppression of PMCA and SERCA accompanied by that of ΔΨ*ceased the ICC pacemaker activity according to scenario observed during isolated reduction of ΔΨ*: the oscillations frequency reduced, duration of oscillatory plateaus of Emand [Ca2+]iextended and, at certain critically low ΔΨ*, the oscillations totally ceased and “downstate” basal [Ca2+]iand rest Emwere established.

Hence, hypoxic suppression of the above considered energy-producing and energy-consuming mechanisms in any combination led the cessation of ICC pacemaker activity and establishment of [Ca2+]iand Em“downstates” near their basal/rest levels without any or with very small oscillations. For the cessation scenario, the main governing factor was suppression of ΔΨ*, and among the Ca2+-ATPase pumps SERCA dominated over PMCA. The observed effects may have crucial pathological consequences for ICC-driven periodic contractions of electrically coupled SMCs manifested as gastrointestinal dysmotility and development of NEC. Since similar Ca2+-handling mechanisms operate in other type excitable cells, particularly in neurons, our model and protocols of computational experiments can be adapted for simulation studies of cellular mechanisms functional consequences of hypoxic injuries of the brain and spinal cord.

## P54 Reconstruction and simulation of the cerebellar microcircuit: a scaffold strategy to embed different levels of neuronal details

### Claudia Casellato^1^, Alice Geminiani^2^, Alessandra Pedrocchi^2^, Elisa Marenzi^1^, Stefano Casali^1^, Chaitanya Medini^1^, Egidio D’Angelo^1^

#### ^1^University of Pavia, Dept. of Brain and Behavioral Sciences - Unit of Neurophysiology, Pavia, Italy; ^2^Politecnico di Milano, Department of Electronics, Information and Bioengineering, Milan, Italy

##### **Correspondence:** Claudia Casellato (claudia.casellato@unipv.it)

*BMC Neuroscience* 2019, **20(Suppl 1)**:P54

Computational models allow propagating microscopic phenomena into large-scale networks and inferencing causal relationships across scales. Here we reconstruct the cerebellar circuit by bottom-up modeling, reproducing the peculiar properties of this structure, which shows a quasi-crystalline geometrical organization well defined by convergence/divergence ratios of neuronal connections and by the anisotropic 3D orientation of dendritic and axonal processes [1].

Therefore, a cerebellum scaffold model has been developed and tested. It maintains scalability and can be flexibly handled to incorporate neuronal properties on multiple scales of complexity. The cerebellar scaffold includes the canonical neuron types: Granular cell, Golgi cell, Purkinje cell, Stellate and Basket cells, Deep Cerebellar Nuclei cell. Placement was based on density and encumbrance values, connectivity on specific geometry of dendritic and axonal fields, and on distance-based probability.

In the first release, spiking point-neuron models based on Integrate & Fire dynamics with exponential synapses were used. The network was run in the neural simulator pyNEST. Complex spatiotemporal patterns of activity, similar to those observed in vivo, emerged [2].

For a second release of the microcircuit model, an extension of the generalized Leaky Integrate & Fire model has been developed, optimized for each cerebellar neuron type and inserted into the built scaffold [3]. It could reproduce a rich variety of electroresponsive patterns with a single set of optimal parameters.

Complex single neuron dynamics and local connectome are key elements for cerebellar functioning.

Then, point-neurons have been replaced by detailed 3D multi-compartment neuron models. The network was run in the neural simulator pyNEURON. Further properties emerged, strictly linked to the morphology and the specific properties of each compartment.

This multiscale tool with different levels of realism has the potential to summarize in a comprehensive way the electrophysiological intrinsic neural properties that drive network dynamics and high-level behaviors.

The model, equipped with ad-hoc plasticity rules, has been embedded in a sensorimotor loop of EyeBlink Classical Conditioning. The network output evolved along repetitions of the task, therefore letting emerge three fundamental operations ascribed to the cerebellum: prediction, timing and learning of motor commands.

**Acknowledgments:** This research was supported by the HBP Neuroinformatics, Brain Simulation, and HPAC Platforms, funded by European Union’s Horizon 2020 under the Specific Grant Agreement No. 785907 (Human Brain Project SGA2), also involving the HBP Partnering Project CerebNEST.

**References**

1. D’Angelo E, Antonietti A, Casali S, et al. Modeling the cerebellar microcircuit: new strategies for a long-standing issue. *Frontiers in cellular neuroscience* 2016 Jul 8;10:176.
2. Casali S, Marenzi E, Medini KC, Casellato C, D‘Angelo E. Reconstruction and Simulation of a Scaffold Model of the Cerebellar Network. *Frontiers in Neuroinformatics* 2019;13:37.
3. Geminiani A, Casellato C, Locatelli F, et al. Complex dynamics in simplified neuronal models: reproducing Golgi cell electroresponsiveness. *Frontiers in Neuroinformatics* 2018, 12, 1–19; 10.3389/fninf.2018.00088

## P55 Simplified and physiologically detailed reconstructions of the cerebellar microcircuit

### Elisa Marenzi^1^, Chaitanya Medini^1^, Stefano Casali^1^, Martina Francesca Rizza^1^, Stefano Masoli^1^, Claudia Casellato^2^, Egidio D’Angelo^2^

#### ^1^University of Pavia, Department of Brain and Behavioural Sciences, Pavia, Italy; ^2^University of Pavia, Dept. of Brain and Behavioral Sciences - Unit of Neurophysiology, Pavia, Italy

##### **Correspondence:** Elisa Marenzi (elisa.marenzi@unipv.it)

*BMC Neuroscience* 2019, **20(Suppl 1)**:P55

The cerebellum is the second largest cortical structure of the brain and contains about half of all brain neurons. Its modeling brings issues reflecting the peculiar properties of the circuit, which has a quasi-crystalline geometrical organization defined by convergence/divergence ratios of neuronal connections and by the anisotropic 3D orientation of dendritic and axonal processes [1]. A data-driven scaffold [2] comprising the granular (GrL), Purkinje (PL), molecular (ML) and Deep Cerebellar Nuclei (DCN) layers has been developed for testing network models with different complexities.

Its reconstruction follows sequential steps. Firstly, cells are placed in the simulation volume through an ad-hoc procedure: the GrL contains glomeruli (glom), granule cells (GrC) and Golgi cells (GoC); somata of Purkinje cells (PC) are in the PL while their dendritic trees are in the ML; here molecular layer interneurons (MLI)—stellate (SC) and basket cells (BC)—are placed whereas the DCN contains only the glutamatergic cells (DCNC).

The connectome stores the IDs of pre- and post-synaptic neurons. Parameters and morphological features derived from physiological experiments and literature data are the basis for its reconstruction, built on geometrical and probability-based rules. When using detailed neuronal morphologies, such rules have been improved to determine dendrites connected also through a touch detection algorithm.

The most typical behaviors of this microcircuit have been tested for both kinds of networks (pyNEST for the point-neuron version and pyNEURON when all detailed morphologies were available). Neuronal discharge of the different neuron populations in response to a mossy fiber burst have been evaluated, showing very similar results between the two simulators. In particular, GoC, SC and BC generate inhibitory bursts that contribute to terminate the GrC and PC bursts and to produce the burst-pause PC response.

Another important behavior regards the PC activation and sensitivity to molecular layer connectivity. The pattern of activity is determined by the various connection properties: particularly, PC inhibition is achieved through a differential orientation between SC and BC axons, while PC excitation depends on both ascending axons (aa) and pf synapses with specific origin from GrC. Their spatial extension reflects the propagation of activity through the MLI network.

The additional details introduced in pyNEURON simulations highlight more complex and physiologically relevant results that cannot be explained with a simplified model without dendrites. Moreover, the integration of the Inferior Olive completes the closed loop of the microcircuit, allowing to embed functional plasticity able to simulate learning processes.

**Acknowledgements:** The research was supported by the EU Horizon 2020 under the Specific Grant Agreements No. 720270 (HBP SGA1) and 785907 (HBP SGA2).

**References**

1. D’Angelo E, Antonietti A, Casali S, et al. Modeling the cerebellar microcircuit: new strategies for a long-standing issue. *Frontiers in cellular neuroscience* 2016 Jul 8;10:176.
2. Casali S, Marenzi E, Medini KC, Casellato C, D‘Angelo E. Reconstruction and Simulation of a Scaffold Model of the Cerebellar Network. *Frontiers in neuroinformatics* 2019;13:37.

## P56 A richness of cerebellar granule cell discharge properties predicted by computational modeling and confirmed experimentally

### Stefano Masoli^1^, Marialuisa Tognolina^1^, Francesco Moccia^2^, Egidio D’Angelo^1^

#### ^1^University of Pavia, Department of Brain and Behavioural Sciences, Pavia, Italy; ^2^University of Pavia, Department of Biology and Biotechnology “L. Spallanzani”, Pavia, Italy

##### **Correspondence:** Stefano Masoli (stefano.masoli@unipv.it)

*BMC Neuroscience* 2019, **20(Suppl 1)**:P56

The cerebellar granule cells (GrCs) are the most common neuron type in the central nervous system. Their highly packed distribution and misleading simple cytoarchitecture, generated the idea of a limited spike generation mechanism. The regular spikes discharge, recorded for short periods of time (<800ms), was the cornerstone for the simulation of realistic [1, 2]. We show that GrCs are capable of diverse patterns response when subjected to prolonged current inject (2s). The somato-dendritic sections were taken from [3], extend with a single section Hillock, an Axon Initial Segment (AIS), an ascending axon and two thin 1mm long parallel fibers. The ionic channels were taken from [1, 2, 4]. The Nav1.6 sodium channel was improved with FHF14 and located in the Hillock and AIS [5]. The calcium buffer was reworked to contain only Calretinin. The models, were automatically fitted with BluePyOpt/NEURON [6]. After 0.8-1s of regular firing, the models predicted three possible outcomes: 1) regular firing, 2) mild adaptation and 3) strong adaptation of firing. Patch-clamp experimental recordings (current-clamp configuration, parasagittal slices obtained from p18-24 Wistar rats) confirmed the modelling predictions on firing adaptation. In a subset of experiments GrCs showed firing acceleration that was not found by the optimization technique. To simulate these GrCs, a TRPM4 channel, known to mediate slow depolarizing currents, was linked to Calmodulin (Cam2C) concentration. This mechanism allowed to reach the accelerated state. These different firing properties impacted on synaptic excitation when the mossy fiber bundle was stimulated at different frequencies (1-100 Hz). Interestingly, a range of different filtering properties emerged, with some cells showing one-to-one responses while others responding faster or slower than the input. This modelling and experimental effort described GrCs properties that show the richness of their encoding capabilities.

**Acknowledgements:** This project has received funding from the Horizon 2020 Framework Programme for Research and Innovation under the Specific Grant Agreement No. 785907 (Human Brain Project SGA2).

**References**

1. D’Angelo E, Nieus T, Maffei A, et al. Theta-frequency bursting and resonance in cerebellar granule cells: experimental evidence and modeling of a slow k+-dependent mechanism. *Journal of Neuroscience* 2001;21:759–70.
2. Diwakar S, Magistretti J, Goldfarb M, Naldi G, D’Angelo E. Axonal Na+ channels ensure fast spike activation and back-propagation in cerebellar granule cells. *Journal of Neurophysiology* 2009;101:519–32.
3. Masoli S, Rizza MF, Sgritta M, Van Geit W, Schürmann F, D’Angelo E. Single Neuron Optimization as a Basis for Accurate Biophysical Modeling: The Case of Cerebellar Granule Cells. *Frontiers in cellular neuroscience* 2017;11:1–14.
4. Masoli S, Solinas S, D’Angelo E. Action potential processing in a detailed Purkinje cell model reveals a critical role for axonal compartmentalization. *Frontiers in cellular neuroscience* 2015;9:1–22.
5. Dover K, Marra C, Solinas S, et al. FHF-independent conduction of action potentials along the leak-resistant cerebellar granule cell axon. *Nature Communications* 2016;7:12895.
6. Van Geit W, Gevaert M, Chindemi G, et al. BluePyOpt: Leveraging Open Source Software and Cloud Infrastructure to Optimise Model Parameters in Neuroscience. *Frontiers in Neuroinformatics* 2016;10:1–30.

## P57 Spatial distribution of Golgi cells inhibition and the dynamic geometry of Cerebellum granular layer activity: a computational study

### Stefano Casali^1^, Marialuisa Tognolina^1^, Elisa Marenzi^1^, Chaitanya Medini^1^, Stefano Masoli^1^, Martina Francesca Rizza^1^, Claudia Casellato^2^, Egidio D’Angelo^1^

#### ^1^University of Pavia, Department of Brain and Behavioural Sciences, Pavia, Italy; ^2^University of Pavia, Department of Brain and Behavioural Sciences - Unit of Neurophysiology, Pavia, Italy

##### **Correspondence:** Stefano Masoli (stefano.masoli@unipv.it)

*BMC Neuroscience* 2019, **20(Suppl 1)**:P57

The cerebellum granular layer (*GL*) has been considered for a long time as a fine-grained spatio-temporal filter, characterized by its main role of delivering the right amount of information at the proper timing to the above molecular layer (*ML*) [1] While this general tenet remains, recent experimental and theoretical works suggest that the *GL* is endowed with a rich and complex variety of spatio-temporal dynamics, empowering the *GL* itself to exert a qualitatively strong influence upon the nature of the signal conveyed to the *ML*.

In the present work, a large-scale computational reconstruction of the *GL* network has been developed, exploiting previously published detailed single cell models of granule cells (*GrCs*, [2]) and Golgi cells [3]. The peculiar structure of synaptic connections has been observed and reproduced by means of geometrical-statistical connectivity rules derived from experimental data, when available [4]. One of the main features of *GL* connectivity, the anisotropic organization of *GoCs* axonal plexus, which is orthogonal to the parallel fibers (*pfs,* coronal axis) and runs along the parasagittal axis, plays a key role in shaping the spatio-temporal dynamics of *GL* activity. Excitatory / inhibitory ratio of *GrCs* response to external stimuli is organized in a center-surround structure, with excitation prevailing in the core and inhibition in the surround area [5] Simulations results show that Golgi cells inhibition is stronger along the parasagittal axis; these computational predictions have been confirmed by a set of experiments in acute slices *in vitro* with high resolution two-photon microscopy. This preferential path for Golgi cells inhibition can also affect how two simultaneously activated distant spots interact: simulations show that spots placed at a 100 or 200mm distance along the parasagittal axis can significantly inhibit each other; on the contrary, when the spots are positioned along the coronal axis, in line with the *pfs,* almost no interaction occurs. Specific synapses modulate the strength of this phenomenon; specifically, when the ascending axon (*aa*) synapses from *GrCs* to *GoCs* are switched-off, inhibitory interaction along the parasagittal axis decreases.

**Acknowledgements:** The research was supported by the EU Horizon 2020 under the Specific Grant Agreements No. 720270 (HBP SGA1) and 785907 (HBP SGA2).

**References**

1. Rössert C, Dean P, Porril J. At the Edge of Chaos: How Cerebellar Granular Layer Network Dynamics Can Provide the Basis for Temporal Filters. *PLoS Computational Biology* 2015. 11(10). 1–28
2. D’Angelo E, Nieus T, Maffei A, et al. Theta-frequency bursting and resonance in cerebellar granule cells: experimental evidence and modeling of a slow K+-dependent mechanism. *Journal of Neuroscience* 2001. 21. 759–770
3. Solinas S, Forti L, Cesana E, et al. Fast-reset of pacemaking and theta-frequency resonance patterns in cerebellar Golgi cells: simulations of their impact in vivo. *Frontiers in Cellular Neuroscience* 2007. 1. 1–9
4. Korbo L, Andresen BB, Ladefoged O, et al. Total numbers of various cell types in rat cerebellar cortex estimated using an unbiased stereological method. *Brain Research* 1993. 609. 262–268
5. Mapelli J, D’Angelo E. The Spatial Organization of Long-Term Synaptic Plasticity at the Input Stage of Cerebellum. *Journal of Neuroscience* 2007. 27. 1285–1296

## P58 Reconstruction and simulation of cerebellum granular layer functional dynamics with detailed mathematical models

### Chaitanya Medini^1^, Elisa Marenzi^1^, Stefano Casali^1^, Stefano Masoli^1^, Claudia Casellato^2^, Egidio D’Angelo^2^

#### ^1^University of Pavia, Department of Brain and Behavioural Sciences, Pavia, Italy; ^2^University of Pavia, Dept. of Brain and Behavioral Sciences - Unit of Neurophysiology, Pavia, Italy

##### **Correspondence:** Elisa Marenzi (elisa.marenzi@unipv.it)

*BMC Neuroscience* 2019, **20(Suppl 1)**:P58

Cerebellum has been widely known to be involved in several cognitive activities, however an elaborate investigation is required to validate known hypotheses and propose new theories. A detailed large-scale scaffold cerebellar circuit was developed with experimental connectivity rules on python NEURON with MPI configuration. An adaptable version of the cerebellar scaffold model [1] is developed on pyNEST and pyNEURON using morphologically-driven cell positions and functional connectivity, inspired from convergence/divergence geometry rules [2]. The reconstruction methodologies used for scaffold network improvises on the existing connectivity literature with Bounded Self-Avoiding Random Walk Algorithm. The simulations revealed a close correspondence to experimental results validating the network reconstruction. Simulations in pyNEURON gave results like those obtained with pyNEST. This is an important validation to ensure that the connectivity generates identical functional dynamics irrespective of the simulator platform. In the current study, pyNEURON scaffold cerebellar model has been extended from point neuron model network to detailed biophysical model network with similar connectome and positions. Detailed multicompartmental models of granule [3], Golgi, Purkinje [4], Stellate and Basket neurons (to be published) are being used for the study. As a first test case, the detailed neuron morphologies are connected using simpleneuronal connectivity rules representing spatially confined convergence/divergence rules. The number of synapses were evenly distributed along the dendritic length of these neuron models to compensate for the absence of computed distance probability between pre and post synaptic neurons. In the second case, a touch-detector based algorithm [5], was used to generate synaptic connectivity in the molecular layer (including Molecular Layer Interneurons and Purkinje Neurons). The network implementation is scalable and flexible to include new types of cell models or to replace the current version with updated models.

**Acknowledgements:** The research was supported by the EU Horizon 2020 under the Specific Grant Agreements No. 720270 (HBP SGA1) and 785907 (HBP SGA2).

**References**

1. Casali S, Marenzi E, Medini KC, Casellato C, D‘Angelo E. Reconstruction and Simulation of a Scaffold Model of the Cerebellar Network. *Frontiers in neuroinformatics* 2019;13:37. 10.1101/532515
2. Solinas S, Nieus T, D‘Angelo E. A realistic large-scale model of the cerebellum granular layer predicts circuit spatio-temporal filtering properties. *Frontiers in cellular neuroscience* 2010 May 14;4:12. 10.3389/fncel.2010.00012
3. Diwakar S, Magistretti J, Goldfarb M, Naldi G, D’Angelo E. Axonal Na+ channels ensure fast spike activation and back-propagation in cerebellar granule cells. *Journal of neurophysiology* 2009 Feb;101(2):519-32. 10.1152/jn.90382.2008
4. Masoli S, Solinas S, D’Angelo E. Action potential processing in a detailed Purkinje cell model reveals a critical role for axonal compartmentalization. *Frontiers in cellular neuroscience* 2015 Feb 24;9:47. 10.3389/fncel.2015.00047
5. Reimann MW, King JG, Muller EB, Ramaswamy S, Markram H. An algorithm to predict the connectome of neural microcircuits. *Frontiers in computational neuroscience* 2015 Oct 8;9:28. 10.3389/fncom.2015.00120

## P59 Reconstruction of effective connectivity in the case of asymmetric phase distributions

### Azamat Yeldesbay^1^, Gereon Fink^2^, Silvia Daun^2^

#### ^1^University of Cologne, Institute of Zoology, Cologne, Germany; ^2^Research Centre Jülich, Institute of Neuroscience and Medicine (INM-3), Jülich, Germany

##### **Correspondence:** Azamat Yeldesbay (azayeld@gmail.com)

*BMC Neuroscience* 2019, **20(Suppl 1)**:P59

The interaction of different brain regions is supported by transient synchronization between neural oscillations at different frequencies. Different measures based on synchronization theory are used to assess the strength of the interactions from experimental data, e.g. the phase-locking index, phase-locking value, phase-amplitude coupling, and cross-frequency coupling. Another approach measuring connectivity based on the reconstruction of the dynamics of phase interactions from experimental data was suggested by [3]. On the basis of this method and the theory of weakly coupled phase oscillators, [2] presented a variant of Dynamic Causal Modelling (DCM) for the analysis of phase-coupled data, where a Bayesian model selection and inversion framework is used to identify the structure and directed connectivity among brain regions from measured time series.

Most of the research on phase analysis relies on the direct association of the phases of the signals with the phases used in the theoretical description of weakly coupled oscillators. However, [1] showed that the phases of the signals measured in experiments are not uniquely defined and an asymmetric distribution of the measured phases (e.g. non-sine form of the signals) could result in a false estimation of the effective connectivity between the network nodes. Furthermore, [1] suggested a solution for this problem by introducing a transformation from an arbitrarily measured phase to a uniquely defined phase variable.

In this work we merge the ideas from the Dynamical Causal Modelling by [2] with the phase dynamics reconstruction by [1] and present a new modelling part that we implemented into DCM for phase coupling. In particular, we extended it with a distortion (a transformation) function that accommodates departures from purely sinusoidal oscillations.

By numerically analysing synthetic data sets with an asymmetric phase distribution, generated from models of coupled stochastic phase oscillators and coupled neural mass models, we demonstrate that the extended DCM for phase coupling with the additional modelling component correctly estimates the coupling functions that do not depend on the distribution of the observables.

The new proposed extension of DCM for phase coupling allows for different intrinsic frequencies among coupled neuronal populations, thereby making it possible to analyse effective connectivity between brain regions within and between different frequency bands, to characterize m:n phase coupling, and to unravel underlying mechanisms of the transient synchronization.

**References**

1. Kralemann B, Cimponeriu L, Rosenblum M, Pikovsky A, Mrowka R. Phase dynamics of coupled oscillators reconstructed from data. *Physical Review E*. 2008 Jun 9;77(6):066205.
2. Penny WD, Litvak V, Fuentemilla L, Duzel E, Friston K. Dynamic causal models for phase coupling. *Journal of neuroscience methods* 2009 Sep 30;183(1):19–30.
3. Rosenblum MG, Pikovsky AS. Detecting direction of coupling in interacting oscillators. *Physical Review E.* 2001 Sep 21;64(4):045202.

## P60 Movement related synchronization affected by aging: A dynamic graph study

### Nils Rosjat, Gereon Fink, Silvia Daun

#### Research Centre Jülich, Institute of Neuroscience and Medicine (INM-3), Jülich, Germany

##### **Correspondence:** Nils Rosjat (n.rosjat@fz-juelich.de)

*BMC Neuroscience* 2019, **20(Suppl 1)**:P60

The vast majority of motor actions, including their preparation and execution, is the result of a complex interplay of various brain regions. Novel methods in computational neuroscience allow us to assess interregional interactions from time series acquired with in-vivo techniques like electro-encephalography (EEG). However, our knowledge of the functional changes in neural networks during non-pathological aging is relatively poor.

To advance our knowledge on this topic, we recorded EEG (64 channels) from 18 right-handed healthy younger subjects (YS, 22–35 years) and 24 right-handed healthy older subjects (OS, 60–79 years) during a simple motor task. The participants had to execute visually-cued low frequency left or right index finger tapping movements. Here, we used the relative phase-locking value (rPLV) [1] to examine whether there is an increase in functional coupling of brain regions during this simple motor task. We analyzed the connectivity for 42 electrodes focusing on connections between electrodes lying above the ipsi- and contralateral premotor and sensorimotor areas and the supplementary motor area.

Widely used approaches for network definition are based on certain functional connectivity measures (e.g. similarity in BOLD time series, phase locking, coherence). These methods typically focus on constructing a single network representation over a fixed time period. However, this approach cannot make use of the high temporal resolution of EEG data and is not able to shed light on the understanding of temporal network dynamics. Here we used graph theory-based metrics that were developed in the last several years that can deal with the analysis of temporally evolving network structures [2].

Our rPLV network analysis revealed four major results: An underlying coupling structure around movement onset in the low frequencies (2–7 Hz) that is present in YS and OS. The network in OS involved several additional connections and showed an overall increased coupling structure (Fig. 1). While the motor related networks of YS mainly involved ipsilateral frontal, contralateral frontal and central electrodes and interhemispheric pairs of electrodes connecting frontal ipsilateral with central contralateral ones, the networks of OS showed especially an increased interhemispheric connectivity. The analysis of hub nodes and communities showed a strong involvement of occipital, parietal, sensorimotor and central regions in YS. While the networks of OS involved similar hub nodes, the first occurrence of sensorimotor regions was clearly delayed and central electrodes played a more important role in the network (Fig. 1). Moreover, the motor related node degrees were significantly increased in OS.

**Fig. 1:**
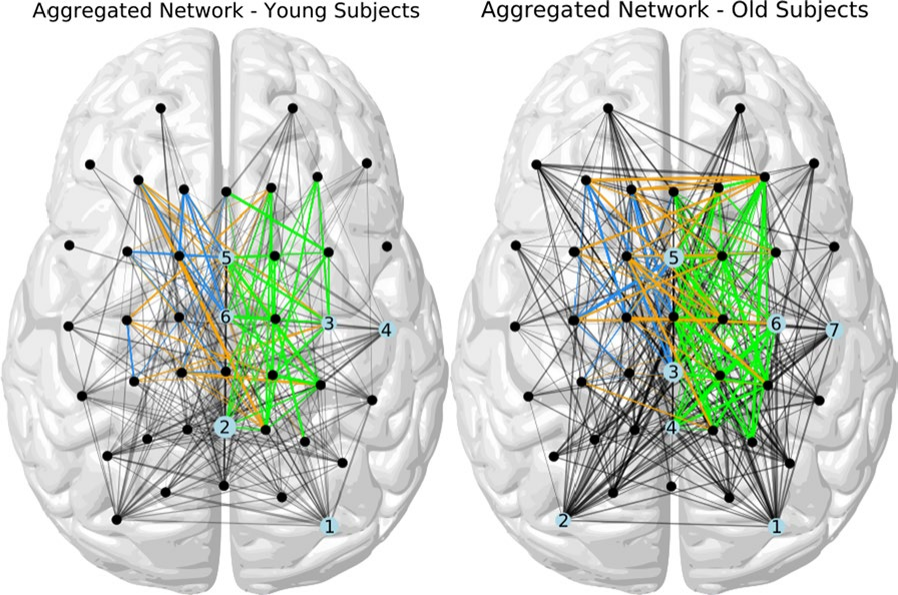
Aggregated networks for younger (left) and older subjects (right) summarizing the network connectivity over the whole time interval. Edges lying above the motor cortex are highlighted in blue (ipsilateral), green (contralateral) and orange (interhemispheric). Hub nodes are marked in the order of first appearance scaled by their frequency

In addition to previously published results [3, 4], we were able to unravel the time-development of specific age-related dynamic network structures that seem to be a necessary prerequisite for the execution of a motor act. The increased interhemispheric connectivity of frontal electrodes fits very well to previous fMRI literature reporting an overactivation in frontal regions in older subjects. Our results also hint at a loss of lateralization via increased connectivity in both hemispheres as well as interhemispheric connections.

**References**

1. Lachaux JP, Rodriguez E, Martinerie J, Varela FJ. Measuring phase synchrony in brain signals. *Human brain mapping* 1999, *8*(4), 194–208.
2. Sizemore AE, Bassett DS. Dynamic graph metrics: Tutorial, toolbox, and tale. *NeuroImage* 2018, 180, 417-427.
3. Dennis NA, Cabeza R. Neuroimaging of healthy cognitive aging. *The handbook of aging and cognition* 2008, 3, 1-54.
4. Cabeza R. Hemispheric asymmetry reduction in older adults: the HAROLD model. *Psychology and aging* 2002, 17(1), 85.

## P61 How a scale-invariant avalanche regime is responsible for the hallmarks of spontaneous and stimulation-induced activity: a large-scale model

### Etienne Hugues, Olivier David

#### Université Grenoble Alpes, Grenoble Institut des Neurosciences, Grenoble, France

##### **Correspondence:** Etienne Hugues (etienne.hugues@upf.edu)

*BMC Neuroscience* 2019, **20(Suppl 1)**:P61

At rest, BOLD fMRI and MEG recordings have revealed the existence of functional connectivity (FC) [1] and of scale-invariant neural avalanches [2], respectively. Under stimulation, neural activity is known to propagate on the brain network and, across trials, firing variability is found to be generically reduced [3]. Understanding the properties of the spontaneous state emerging on the brain network, together with its modifications during stimulation is a fundamental problem in neuroscience, still largely untouched.

A large-scale modeling approach, where the brain network is modeled by local neuronal networks connected through the large-scale connectome, has been previously used. Assuming that the whole brain is in an asynchronous state, the noisy fluctuations reverberating on the network have been found to be responsible for BOLD FC. However, in this fluctuation scenario, stimulation-induced activity is strongly damped while propagating on the network, even when trying to correct for this limitation [4].

We show that low spontaneous firing prevents neural activity to propagate in the fluctuation scenario. Adding neural adaptation, a local node can have two dynamical states, allowing the network dynamics to escape from the fluctuation regime, through brief excursions of individual nodes towards the higher activity state, allowing neural activity to effectively propagate on the network.

In the spontaneous state, the model exhibits neural avalanches, whose size distribution is scale-invariant for some global coupling strength value. BOLD FC is found to originate from the avalanches, therefore from nonlinear dynamics. The best agreement with empirical BOLD FC is found for scale-invariant avalanches.

Stimulation tends to entrain some nodes towards the high activity state, eliciting a reproducible propagation on the network, which simultaneously leads to a decrease of neural variability compared to the spontaneous state where more fluctuations occur, attributing a global origin to this phenomenon. Finally, neural activity is found to propagate optimally in the scale-invariant avalanche regime.

In conclusion, this study demonstrates that, beyond the brain connectome, a spontaneous state in the scale-invariant avalanche regime is crucial to reproduce the hallmarks of spontaneous and stimulation-induced activity. Neural variability decreases wherever activity propagates reliably, going beyond experimental results [3] and previously proposed mechanisms. Overall, the present work proposes a unified theory of the large-scale brain dynamics for a wide range of experimental findings.

**Acknowledgments:** We thank the European Research Council for supporting E.H. and O.D. with E.U.’s 7th Framework Programme / ERC Grant 616268 “F-TRACT”.

**References**

1. Fox MD, Raichle ME. Spontaneous fluctuations in brain activity observed with functional magnetic resonance imaging. *Nature Reviews Neuroscience* 2007, 8, 700–711.
2. Shriki O, Alstott J, Carver F, et al. Neuronal avalanches in the resting MEG of the human brain. *Journal of Neuroscience* 2013, 33, 7079–7090.
3. Churchland MM, Yu BM, Cunningham JP, et al. Stimulus onset quenches neural variability: a widespread cortical phenomenon. *Nature Neuroscience* 2010, 13, 369–378.
4. Joglekar MR, Mejias JF, Yang GR, et al. Inter-areal balanced amplification enhances signal propagation in a large-scale circuit model of the primate cortex. *Neuron* 2018, 98, 222–234.

## P62 Astrocytes restore connectivity and synchronization in dysfunctional cerebellar networks

### Paolo Bonifazi^1^, Sivan Kanner^2^, Miri Goldin^1^, Ronit Galron^2^, Eshel Ben Jacob^3^, Ari Barzilai^2^, Maurizio De Pitta’^4^

#### ^1^Biocruces Health Research Institute, Neurocomputational imaging, Bilbao, Spain; ^2^Tel Aviv University, Department of Neurobiology, Tel Aviv, Israel; ^3^Tel Aviv University, School of Physics and Astronomy, Tel Aviv, Israel; ^4^Basque Center for Applied Mathematics: BCAM, Bilbao, Spain

##### **Correspondence:** Paolo Bonifazi (paol.bonifazi@gmail.com)

*BMC Neuroscience* 2019, **20(Suppl 1)**:P62

In the last two decades, it has become appreciated that glial cells play a critical role in brain degenerative diseases (BDDs). The symptoms of BDDs arise from pathological changes to neuro-glia interactions, leading to neuronal cell death, disrupted neuro-glia communication, and impaired cell function, all of which affect global dynamics of brain circuitry. Astrocytes, a particular glial cell type, play key roles in regulating pathophysiology of neuronal functions. In this work, we tested the hypothesis that neuronal circuit dynamics were impacted as a consequence of disrupted neuron-astrocyte physiology in a mouse model of the BDD that results from a deficiency in the ATM protein. The gene encoding ATM is mutated in the human genetic diseaseAtaxia-Telangiectasia (A-T). One of the most devastating symptoms of A-T is the cerebellar ataxia, with significant loss of Purkinje and granule neurons in the cerebellum, that leads progressively to general motor dysfunction. We used primary cerebellar cultures grown from postnatal wild-type (WT) and *Atm* −/− mice to study how ATM deficiency influences the structure and dynamics of cerebellar neuronal-astrocyte circuits. We hypothesized that ATMdeficiency impairs the neuronal-astrocytic interactions underlying spontaneous neuronal synchronizations, a hallmark activity pattern of the developing nervous system.

We report that the absence of Atm in neurons and astrocytes severely alters astrocyte morphology and the number of pre- and post-synaptic puncta, disrupting the topology and dynamics of cerebellar networks. Functionally, *Atm* −/− networks showed a reduced number of global synchronizations (GSs) which recruited the whole imaged neuronal population, in favor of an increased number of sparse synchronizations (SSs), where only a small subset of neurons of the network fired in together. Structurally, higher numbers of synaptic puncta in *Atm*−/−networks relative to numbers in wild-type cultures were associated with lower levels of autophagy. These reported structural and functional anomalies were all rescued in chimeric neuronal networks composed of *Atm*−/−neurons and WT astrocytes. In contrast, cultures of WT neurons with *Atm*−/−astrocytes led to significant neuronal cell death. Characterizations of adult *Atm*−/−cerebella similarly showed disrupted astrocyte morphology, upregulated GABAergic markers, and dysregulated mTOR-mediated signaling and autophagy.

The apparent contradiction between a larger number of synapses in the Atm−/−circuits and lower occurrence of network synchronizations could result from the presence of non-functional connections (aborted functional connectivity hypothesis) or from the homeostatic downscaling of synaptic weights between neurons (aborted effective connectivity hypothesis). We explore the latter hypothesis extrapolating on its possible consequences on in-vivo cerebellar dynamics. With this regard we presenta spiking neural network model for the above described in-vitro experiments, where increase in connectivity in parallel withscaling of synaptic weightscan account for the increase of SSs in the KO model. Next, we consider the same increase in connectivity yet in relation to GABAergic transmission in a simplified model of cerebellar circuits and we show that an increase of inhibitory connections results in a reduction of functional connections in evoked excitatory activity, suggesting disrupted sensory and motor processing cascade in ataxia.

## P63 Pybrep: Efficient and extensible software to construct an anatomical basis for a physiologically realistic neural network model

### Ines Wichert^1^, Sanghun Jee^2^, Sungho Hong^3^, Erik De Schutter^3^

#### ^1^Champalimaud Center for the Unknown, Champalimaud Research, Lisbon, Portugal; ^2^Korea University, College of Life Science and Biotechnology, Seoul, South Korea; ^3^Okinawa Institute of Science and Technology, Computational Neuroscience Unit, Okinawa, Japan

##### **Correspondence:** Sungho Hong (shhong@oist.jp)

*BMC Neuroscience* 2019, **20(Suppl 1)**:P63

In building a physiologically realistic model of a neural network, one of the first challenges is to determine the positions of neurons and their mutual connectivity based on their anatomic features. Recent studies have shown that cell locations are often distributed in non-random spatial patterns [1–3]. Also, synaptic and gap junction-mediated connectivity is constrained by the spatial geometry of axonal and dendritic arbors. These features have to be taken into account for realistic modeling since they determine convergence/divergence of the input/output of the neurons, respectively, and fundamentally impact their spatiotemporal activity patterns [4,5].

Here we present pybrep, an easily usable and extendable Python tool, designed for efficient generation of cell positions and connectivity based on anatomical data in large neuronal networks, and demonstrate its successful application to our previously published network model of the cerebellar cortex [5] and its extension. In a first step, pybrep generates cell positions by the Poisson disk sampling algorithm [6]: By sampling quasi-random points in a space with a constraint on their mutual distances, it simulates tight packing of spherical cells with given radii. We adapted this to generate multiple cell types sequentially and apply coordinate transformations to compensate for anisotropic geometry. Based on those locations, it generates point clouds representing specified axonal and dendritic morphologies. Using an efficient nearest neighbor search algorithm, it then identifies candidate connections by finding points that satisfy a distance condition. This can be done in 3D, or in some cases even more efficiently with a 2D projection method that exploits morphological regularities such as the long parallel fibers in the cerebellar network.

In the setup process for the cerebellar cortex model, pybrep efficiently produced the positions of more than a million cellular structures, including granule and Golgi cells as well as mossy fiber glomeruli, based on existing data about densities, volume ratios, etc. [7] Notably, applying a physiologically plausible, distance-based connection rule to the generated positions reproduced the well-known 4-to-1 connectivity between glomeruli and granule cells [7]. Pybrep also generated synaptic connectivity, particularly between the granule and Golgi cells, by an order of magnitude faster compared to our previous software for the same task [5]. Finally, the modular structure of pybrep allowed for an easy extension of the existing model by adding a new cell type, the molecular layer interneuron.

Pybrep depends only on a few external packages, but can easily be combined with existing Python tools, such as those for parallelization and scaling-up. These features will make pybrep a useful tool for constructing diverse network models in various sizes.

**References**

1. Töpperwien M, van der Meer F, Stadelmann C, Salditt T. Three-dimensional virtual histology of human cerebellum by X-ray phase-contrast tomography. *PNAS* 2018 Jul 3;115(27):6940–5.
2. Jiao Y, Lau T, Hatzikirou H, Meyer-Hermann M, Corbo JC, Torquato S. Avian photoreceptor patterns represent a disordered hyperuniform solution to a multiscale packing problem. *Physical Review E* 2014 Feb 24;89(2):022721.
3. Haruoka H, Nakagawa N, Tsuruno S, Sakai S, Yoneda T, Hosoya T. Lattice system of functionally distinct cell types in the neocortex. *Science* 2017 Nov 3;358(6363):610–5.
4. Rosenbaum R, Smith MA, Kohn A, Rubin JE, Doiron B. The spatial structure of correlated neuronal variability. *Nature neuroscience* 2017 Jan;20(1):107.
5. Sudhakar SK, Hong S, Raikov I, et al. Spatiotemporal network coding of physiological mossy fiber inputs by the cerebellar granular layer. *PLoS computational biology* 2017 Sep 21;13(9):e1005754.
6. Bridson R. Fast Poisson disk sampling in arbitrary dimensions. *In SIGGRAPH sketches* 2007 Aug 5 (p. 22).
7. Billings G, Piasini E, Lőrincz A, Nusser Z, Silver RA. Network structure within the cerebellar input layer enables lossless sparse encoding. *Neuron* 2014 Aug 20;83(4):960–74.

## P64 3D modeling of complex spike bursts in a cerebellar Purkinje cell

### Alexey Martyushev^1^, Erik De Schutter^2^

#### ^1^Okinawa Institute of Science and Technology (OIST), Erik De Schutter Unit, Onna-son, Okinawa, Japan; ^2^Okinawa Institute of Science and Technology, Computational Neuroscience Unit, Onna-Son, Japan

##### **Correspondence:** Alexey Martyushev (martyushev.alexey@gmail.com)

*BMC Neuroscience* 2019, **20(Suppl 1)**:P64

The cerebellum regulates motor movements through the function of its Purkinje neurons. Purkinje neurons generate electrophysiological activity in the form of firing simple (fast) and complex (slow) spikes differing in the number of spikes, amplitude and duration. The interest in studying the complex spike bursts is based on their role in controlling and learning human body movements.

This study describes a new version of the recently published spatial single Purkinje cell model implemented in the NEURON simulation software by [1]. This model uses a variety of ionic mechanisms to generate simple and complex spike activity. We analyze the difference in modeling results between the NEURON [2] and the Stochastic Engine for Pathway Simulation (STEPS) [3] simulation environments. The NEURON modeling approach idealizes the complex 3D morphology as cylinders (>10 µm scale) with uniform membrane properties and considers only 1D membrane potential propagation, while STEPS treats the neuron morphology in the form of a more detailed (<1 µm scale) tetrahedral 3D mesh [3]. These differences affect channel properties and calcium dynamics in the Purkinje cell model. Additionally, the need of detailed neuronal modeling leverages the increase of using electron microscopy to provide super resolution neuronal reconstructions.

The results of this study will detail our understanding of intrinsic properties and functioning of neurons at the nanoscale. Possible differences between the two software tools may require us to reconsider our approaches to computational modelling of the neuronal activity in the brain [4].

**References**

1. Zang Y, Dieudonne S, De Schutter E. Voltage- and Branch-Specific Climbing Fiber Responses in Purkinje Cells. *Cell Rep.* 2018, 24(6), p. 1536–1549.
2. Carnevale NT, Hines M. The NEURON Book. *Cambridge, UK: Cambridge University Press;* 2006.
3. Hepburn I, et al. STEPS: efficient simulation of stochastic reaction-diffusion models in realistic morphologies. *BMC Syst Biol.* 2012, 6, p. 36.
4. Chen W, De Schutter E. Time to Bring Single Neuron Modeling into 3D. *Neuroinformatics* 2017, 15, p. 1–3.

## P65 Hybrid modelling of vesicles with spatial reaction-diffusion processes in STEPS

### Iain Hepburn, Sarah Nagasawa, Erik De Schutter

#### Okinawa Institute of Science and Technology, Computational Neuroscience Unit, Onna-son, Japan

##### **Correspondence:** Iain Hepburn (ihepburn@oist.jp)

*BMC Neuroscience* 2019, **20(Suppl 1)**:P65

Vesicles play a central role in many fundamental neuronal cellular processes. For example, pre-synaptic vesicles package, transport and release neurotransmitter, and post-synaptic AMPAR trafficking is controlled by the vesicular-endosomal pathway. Therefore, vesicle trafficking underlies crucial brain features such as the dynamics and strength of chemical synapses, yet vesicles have only received limited attention in computational neuronal modelling to now.

Molecular simulation software STEPS (steps.sourceforge.net) applies reaction-diffusion kinetics on realistic tetrahedral mesh structures by tracking the molecular population within tetrahedrons and modelling their local interactions stochastically. STEPS is usually applied to subcellular models such as synaptic plasticity pathways and so is a natural choice for extension to vesicle processes. However, combining vesicle modelling with mesh-based reaction-diffusion modelling poses a number of challenges.

The fundamental issue to solve is the interaction between spherical vesicle objects and the tetrahedral mesh. We apply an overlap library and track local vesicle-tetrahedron overlap, which allows us to modify local diffusion rates and model interactions between vesicular surface proteins and molecules in the tetrahedral mesh such as cytosolic and plasma membrane proteins as the vesicles sweep through the mesh. These interactions open up many modelling applications such as vesicle-endosome interaction, membrane-docking, priming and neurotransmitter release, all solved to a high level of spatial and biochemical detail.

This hybrid modelling, that includes dynamic vesicle processes and dependencies, presents challenges in ensuring accuracy whilst maintaining efficiency of the software, and this is an important focus of our work. Where possible we validate the accuracy of our modelling processes, for example by validating diffusion and binding rates. Optimisation efforts are ongoing but we have had some successes, for example by applying local updates to the dynamic vesicle processes.

We apply this new modelling technology to the post-synaptic AMPAR trafficking pathway. AMPA receptors undergo clatherin-dependent endocytosis and are trafficked to the endosome where they are sorted for either degradation or returned to the membrane via recycling vesicles. Rab GTPases coordinate sorting through the endosomal system.

Due to our new hybrid modelling technology it is possible to simulate this pathway, as well as potentially other areas of cell biology where vesicle trafficking and function play an important role, to high spatial detail. We hope that our current efforts and future additions open up new avenues of modelling research in neuroscience.

## P66 A computational model of social motivation and effort

### Ignasi Cos^1^, Gustavo Deco^2^

#### ^1^Pompeu Fabra University, Center for Brain & Cognition, Barcelona, Spain; ^2^Universitat Pompeu Fabra, Barcelona, Spain

##### **Correspondence:** Ignasi Cos (ignasi.cos@gmail.com)

*BMC Neuroscience* 2019, **20(Suppl 1)**:P66

Although the relationship between motivation and behaviour has been extensively studied, the specifics of how motivation relates to movement and how effort is considered to select specific movements remains largely controversial. Indeed, moving towards valuable states implies investing a certain amount of effort and coming up with appropriate motor strategies. How are these principles modulated by social pressure?

To investigate whether and how motor parameters and decisions between movements were influenced by differentially induced motivated states, we performed a decision-making paradigm where healthy human participants made choices between reaching movements under different conditions. Their goal was to accumulate reward by selecting one of two reaching movements of opposite motor cost, and to perform the selected reaching movement. Reward was contingent upon target arrival precision. All trials had fixed duration to prevent the participants from maximizing reward by minimizing temporal discount.

We manipulated the participants’ motivated state via social pressure. Each experimental session was composed of six blocks, during which subjects could either play alone or accompanied by a simulated co-player. Within this illusion, the amount of reward obtained by the participant and by their companion was reported at the end of each trial. The previous ten trial ranking for the two players was shown briefly every nine trials. However, no specific mention to competition was ever made to the subjects in the instruction, and any such mention reported by the participant was immediately rejected by the experimenter.

The results show that participants increased precision alongside the skill of their co-actor, implying that the participants cared about their own performance. The main behavioural result was an increase of the movement duration between baseline (playing alone) and any other condition (with any co-actor), and a modulation of amplitude as the skill of the co-actor became unattainable. As to provide a quantitative account of the dynamics of social motivation, we developed a generative computational model of decision-making and motor control, based on the optimization of the trade-off between the benefits and costs associated to a movement. Its predictions show that this optimization depends on the motivational context where the movements and the choices between them are performed. Although further research remains to be performed to understand the specific intricacies of this relationship between motor control theory and motivated states, this suggests that this inter-relation between internal physiological dynamics and motor behaviour is more than a simple modulation of the vigour of movement.

**Acknowledgements:** This project was funded by the Marie Sklodowska-Curie Research Grant Scheme (grant number IF-656262).

## P67 Functional inference of real neural networks with artificial neural networks

### Mohamed Bahdine^1^, Simon V. Hardy^2^, Patrick Desrosiers^3^

#### ^1^Laval University, Quebec, Canada; ^2^Laval University, Département d’informatique et de génie logiciel, Quebec, Canada; ^3^Laval University, Département de physique, de génie physique et d’optique, Quebec, Canada

##### **Correspondence:** Mohamed Bahdine (mohamed.bahdine.1@ulaval.ca)

*BMC Neuroscience* 2019, **20(Suppl 1)**:P67

Fast extraction of connectomes from whole-brain functional imaging is computationally challenging. Despite the development of new algorithms that efficiently segment the neurons in calcium imaging data, the detection of individual synapses in whole-brain images remains intractable. Instead, connections between neurons are inferred using time series that describe the evolution of neurons’ activity. We compare classical methods of functional inference such as Granger Causality (GC) and Transfer Entropy (TE) to deep learning approaches such as Convolutional Neural Networks (CNN) and Long Short-Term Memory (LSTM).

Since ground truth is required to compare the methods, synthetic time series are generated from the C. Elegans’ connectome using the leaky-integrate and fire neuron model. Noise, inhibition and adaptation are added to the model to promote richer neuron activity. To mimic typical calcium-imaging data, the time series are down-sampled from 10 kHz to 30 Hz and filtered with calcium and fluorescence dynamics. Additionally, we produce multiple simulations by varying brain and stimulation parameters to test each inference methods on different types of brain activity.

By comparing the mean ROC curves of each method (see Fig. 1) we find that the CNN outperforms all other methods up to a false positive rate of 0.7, while GC has the weakest performance, being on average slightly above random guesses. TE performs better than LSTM for low false positive rates, but these performances are inverted for false positive rates higher than 0.5. Although the CNN has the highest mean curve, it also has the largest width, meaning the CNN is the most variable and therefore least consistent inference method. TE’s mean ROC curve’s width is significantly narrower than other methods for low false positive rates and slowly grows when it meets other curves. The choice of an inference method is therefore dependant on one’s tolerance to false positives and variability.

**Fig. 1:**
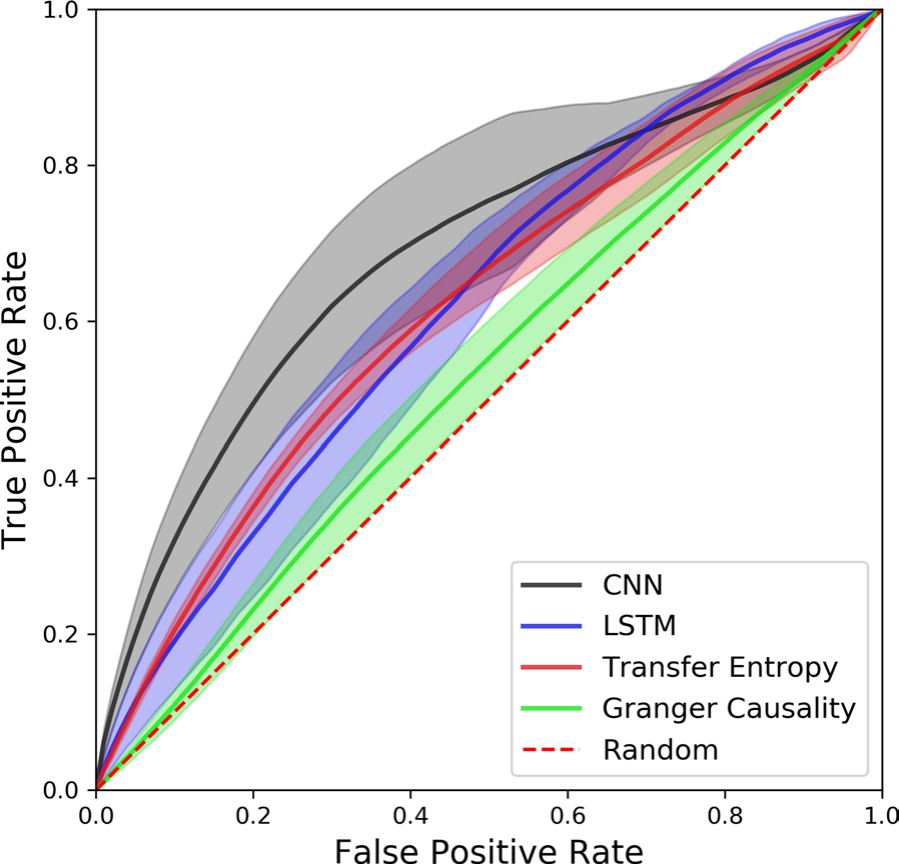
Average Receiver Operating Characteristic (ROC) curves for each functional inference method. The average is computed from 46 ROC curves from as many simulations. The width corresponds to the standard deviation. The red-dotted diagonal corresponds to random guesses

## P68 Stochastic axon systems: A conceptual framework

### Skirmantas Janusonis^1^, Nils-Christian Detering^2^

#### ^1^University of California, Santa Barbara, Department of Psychological and Brain Sciences, Santa Barbara, CA, United States of America; ^2^University of California, Santa Barbara, Department of Statistics and Applied Probability, Santa Barbara, CA, United States of America

##### **Correspondence:** Skirmantas Janusonis (janusonis@ucsb.edu)

*BMC Neuroscience* 2019, **20(Suppl 1)**:P68

The brain contains many “point-to-point” projections that originate in known anatomical locations, form distinct fascicles or tracts, and terminate in well-defined destination sites. This “deterministic brain” coexists with the “stochastic brain,” the axons of which disperse in meandering trajectories, creating meshworks in virtually all brain nuclei and laminae. The cell bodies of this system are typically located in the brainstem, as a component of the ascending reticular activating system (ARAS). ARAS axons (fibers) release serotonin, dopamine, norepinephrine, acetylcholine, and other neurotransmitters that regulate perception, cognition, and affective states. They also play major roles in human mental disorders (e.g., Major Depressive Disorder and Autism Spectrum Disorder).

Our interdisciplinary program [1, 2] seeks to understand at a rigorous level how the behavior of individual ARAS fibers determines their equilibrium densities in brain regions. These densities are commonly used in fundamental and applied neuroscience and can be thought to represent a macroscopic measure that has a strong spatial dependence (conceptually similar to temperature in thermodynamics). This measure provides essential information about the environment neuronal ensembles operate in, since ARAS fibers are present in virtually all brain regions and achieve extremely high densities in many of them.

A major focus of our research is the identification of the stochastic process that drives individual ARAS trajectories. Fundamentally, it bridges the stochastic paths of single fibers and the essentially deterministic fiber densities in the adult brain. Building upon state-of-the-art microscopic analyses and theoretical models, the project investigates whether the observed fiber densities are the result of self-organization, with no active guidance by other cells. Specifically, we hypothesize that the knowledge of the geometry of the brain, including the spatial distribution of physical “obstacles” in the brain parenchyma, provides key information that can be used to predict regional fiber densities.

In this presentation, we focus on serotonergic fibers. We demonstrate that a step-wise random walk, based on the von Mises-Fisher (directional) probability distribution, can provide a realistic and mathematically concise description of their trajectories in fixed tissue. Based on the trajectories of serotonergic fibers in 3D-confocal microscopy images, we present estimates of the concentration parameter (κ) in several brain regions with different fiber densities. These estimates are then used to produce computational simulations that are consistent with experimental results. We also propose that other stochastic models, such as the superdiffusion regime of the Fractional Brownian Motion (FBM), may lead to a biologically accurate and analytically rich description of ARAS fibers, including their temporal dynamics.

**Acknowledgements:** This research is funded by the National Science Foundation (NSF 1822517), the National Institute of Mental Health (R21 MH117488), and the California NanoSystems Institute (Challenge-Program Development Grant).

**References**

1. Janusonis S, Detering N. A stochastic approach to serotonergic fibers in mental disorders. *Biochimie* 2018, in press.
2. Janusonis S, Mays KC, Hingorani MT. Serotonergic fibers as 3D-walks. *ACS Chem. Neurosci.* 2019, in press.

## P69 Replicating the mouse visual cortex using Neuromorphic hardware

### Srijanie Dey, Alexander Dimitrov

#### Washington State University Vancouver, Mathematics and Statistics, Vancouver, WA, United States of America

##### **Correspondence:** Srijanie Dey (srijanie.dey@wsu.edu)

*BMC Neuroscience* 2019, **20(Suppl 1)**:P69

The primary visual cortex is one of the most complex parts of the brain offering significant modeling challenges. With the ongoing development of neuromorphic hardware, simulation of biologically realistic neuronal networks seems viable. According to [1], Generalized Leaky Integrate and Fire Models (GLIFs) are capable of reproducing cellular data under standardized physiological conditions. The linearity of the dynamical equations of the GLIFs also work to our advantage. In an ongoing work, we proposed the implementation of five variants of the GLIF model [1], incorporating different phenomenological mechanisms, into Intel’s latest neuromorphic hardware, Loihi. Owing to its architecture that supports hierarchical connectivity, dendritic compartments and synaptic delays, the current LIF hardware abstraction in Loihi is a good match to the GLIF models. In spite of that, precise detection of spikes and the fixed-point arithmetic on Loihi pose challenges. We use the experimental data and the classical simulation of GLIF as references for the neuromorphic implementation. Following the benchmark in [2], we use various statistical measures on different levels of the network to validate and verify the neuromorphic network implementation. In addition, variance among the models and within the data based on spike times are compared to further support the network’s validity [1, 3]. Based on our preliminary results, viz., implementation of the first GLIF model followed by a full-fledged network in the Loihi architecture, we believe it is highly probable that a successful implementation of a network of different GLIF models could lay the foundation for replicating the complete primary visual cortex.

**References**

1. Teeter C, Iyer R, Menon V, et al. Generalized leaky integrate-and-fire models classify multiple neuron types. *Nature communications* 2018 Feb 19;9(1):709.
2. Trensch G, Gutzen R, Blundell I, Denker M, Morrison A. Rigorous neural network simulations: a model substantiation methodology for increasing the correctness of simulation results in the absence of experimental validation data. *Frontiers in Neuroinformatics* 2018;12.
3. Paninski L, Simoncelli EP, Pillow JW. Maximum likelihood estimation of a stochastic integrate-and-fire neural model. *Advances in Neural Information Processing Systems* 2004; pp 1311–1318.

## P70 Understanding modulatory effects on cortical circuits through subpopulation coding

### Matthew Getz^1^, Chengcheng Huang^2^, Brent Doiron^2^

#### ^1^University of Pittsburgh, Neuroscience, Pittsburgh, PA, United States of America; ^2^University of Pittsburgh, Mathematics, Pittsburgh, United States of America

##### **Correspondence:** Matthew Getz (mpg39@pitt.edu)

*BMC Neuroscience* 2019, **20(Suppl 1)**:P70

Information theoretic approaches have shed light on the brain’s ability to efficiently propagate information along the cortical hierarchy, as well as exposed limitations in this process. One common measure of coding capacity, linear Fisher Information (FI), has also been used to study the neural code within a given cortical region. In particular, we recently used this approach to study the effects of an attention-like modulation on a cortical population model [1]. Previous studies have been largely agnostic as to the class of neuron that encodes a particular sensory variable, assuming little more than stimulus tuning properties. While it is widely accepted that local cortical dynamics involve an interplay between excitatory and inhibitory neurons, there are a large number of anatomical studies showing that excitatory neurons are the dominant projection neurons from one cortical area to the next. This suggests that, rather than maximizing the FI across the full excitatory and inhibitory network, to improve down-stream readout of neural codes the goal of top-down modulation may instead be to modulate the information carried only within the excitatory population, denoted FI-E [1]. In this study we explore this hypothesis using a combined numerical and analytic analysis of population coding in simplified model cortical networks.

We first study this effect in a recurrently coupled, excitatory (E)/inhibitory (I) population pair coding for a scalar stimulus variable (Fig. 1, A). We demonstrate that while the FI of the full E/I network does not change with a top-down modulation (Fig. 1, C; dashed colored lines), FI-E can nevertheless increase (Fig. 1, C; solid colored lines). We derive intuition for this key difference between FI and FI-E by considering the combined influence of input correlation and recurrent connectivity (captured by the ratio a in Fig. 1, C, middle plots. Light points show the ratio a before modulation; dark points, after modulation. Green and purple correspond to two different sets of network parameters. Fig. 1, Ci corresponds to input correlations = 0.9; Cii, to input correlations = 0.5).

**Fig. 1:**
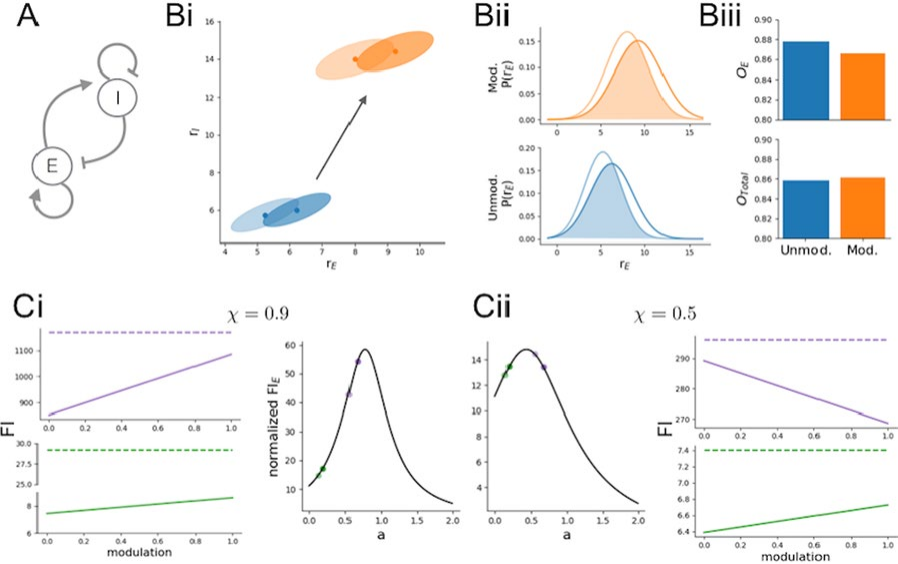
**a** Network schematic. **b** (i, ii) Distribution of firing rates for E and I before (blue) and after (orange) modulation at a given contrast c (light ellipse) and c+dc (dark ellipse) where dc is a small perturbation in the input. (iii) Calculated overlap of the rate distributions for E and E/I (Total). **c** The effects of modulation depend on the input correlations and recurrent connectivity

Finally, we will further extend these ideas to a distributed population code by considering a framework with multiple E/I populations encoding a periodic stimulus variable [2]. In total, our results develop a new framework in which to understand how top-down modulation may exert a positive effect on cortical population codes.

**References**

1. Kanashiro T, Ocker GK, Cohen MR, Doiron B. Attentional modulation of neuronal variability in circuit models of cortex. *Elife* 2017 Jun 7; 6:e23978.
2. Getz M P, Huang C, Dunworth J, Cohen M R, Doiron B. Attentional modulation of neural covariability in a distributed circuit-based population model. *Cosyne Abstracts 2018*, Denver, CO, USA.

## P71 Stimulus integration and categorization with bump attractor dynamics

### Jose M. Esnaola-Acebes^1^, Alex Roxin^1^, Klaus Wimmer^1^, Bharath C. Talluri^2^, Tobias Donner^2,3^

#### ^1^Centre de Recerca Matemàtica, Computational Neuroscience group, Barcelona, Spain; ^2^University Medical Center Hamburg-Eppendorf, Department of Neurophysiology & Pathophysiology, Hamburg, Germany; ^3^University of Amsterdam, Department of Psychology, Amsterdam, The Netherlands

##### **Correspondence:** Jose M. Esnaola-Acebes (jmesnaola@crm.cat)

*BMC Neuroscience* 2019, **20(Suppl 1)**:P71

Perceptual decision making often involves making categorical judgments based on estimations of continuous stimulus features. It has recently been shown that committing to a categorical choice biases a subsequent report of the stimulus estimate by selectively increasing the weighting of choice-consistent evidence [1]. This phenomenon, known as confirmation bias, commonly results in a suboptimal performance in people’s perceptual decisions. The underlying neural mechanisms that give rise to this phenomenon are still poorly understood.

Here we develop a computational network model that can integrate a continuous stimulus feature such as motion direction and can also account for a subsequent categorical choice. The model, a ring attractor network, represents the estimate of the integrated stimulus direction in the phase of an activity bump. A categorical choice can then be achieved by applying a decision signal at the end of the trial forcing the activity bump to move to one of two opposite positions. We reduced the network dynamics to a two-dimensional equation for the amplitude and the phase of the bump which allows for studying evidence integration analytically. The model can account for qualitatively distinct decision behaviors, depending on the relative strength of sensory stimuli compared to the amplitude of the bump attractor. When sensory inputs dominate over the intrinsic network dynamics, later parts of the stimulus have a higher impact on the final phase and the categorical choice than earlier parts (“recency” regime). On the other hand, when the internal dynamics are stronger, the temporal weighting of stimulus information is uniform. The corresponding psychophysical kernels are consistent with experimental observations [2]. We then simulated how stimulus estimation is affected by an intermittent categorical choice [1] by applying the decision signal after the first half of the stimulus. We found that this biases the resulting stimulus estimate at the end of the trial towards larger values for stimuli that are consistent with the categorical choice and towards smaller values for stimuli that are inconsistent, resembling the experimentally observed confirmation bias.

Our work suggests bump attractor dynamics as a potential underlying mechanism of stimulus integration and perceptual categorization.

**Acknowledgments:** Funded by the Spanish Ministry of Science, Innovation and Universities and the European Regional Development Fund (grants RYC-2015-17236, BFU2017-86026-R and MTM2015-71509-C2-1-R) and by the Generalitat de Catalunya (grant AGAUR 2017 SGR 1565).

**References**

1. Talluri BC, Urai AE, Tsetsos K, Usher M, Donner TH. Confirmation bias through selective overweighting of choice-consistent evidence. *Current Biology* 2018 Oct 8;28(19):3128–35.
2. Wyart V, De Gardelle V, Scholl J, Summerfield C. Rhythmic fluctuations in evidence accumulation during decision making in the human brain. *Neuron* 2012 Nov 21;76(4):847–58.

## P72 Topological phase transitions in functional brain networks

### Fernando Santos^1^, Ernesto P Raposo^2^, Maurício Domingues Coutinho-Filho^2^, Mauro Copelli^2^, Cornelis J Stam^3^, Linda Douw^4^

#### ^1^Universidade Federal de Pernambuco, Departamento de Matemática, Recife, Brazil; ^2^Universidade Federal de Pernambuco, Departamento de Física, Recife, Brazil; ^3^Vrije University Amsterdam Medical Center, Department of Clinical Neurophysiology and MEG Center, Amsterdam, Netherlands; ^4^Vrije University Amsterdam Medical Center, Department of Anatomy & Neurosciences, Amsterdam, Netherlands

##### **Correspondence:** Fernando Santos (fansantos@dmat.ufpe.br)

*BMC Neuroscience* 2019, **20(Suppl 1)**:P72

Functional brain networks are often constructed by quantifying correlations between time series of activity of brain regions. Their topological structure includes nodes, edges, triangles and even higher-dimensional objects. Topological data analysis (TDA) is the emerging framework to process datasets under this perspective. In parallel, topology has proven essential for understanding fundamental questions in physics. Here we report the discovery of topological phase transitions in functional brain networks by merging concepts from TDA, topology, geometry, physics, and network theory. We show that topological phase transitions occur when the Euler entropy has a singularity, which remarkably coincides with the emergence of multidimensional topological holes in the brain network, as illustrated in Fig. 1. The geometric nature of the transitions can be interpreted, under certain hypotheses, as an extension of percolation to high-dimensional objects. Due to the universal character of phase transitions and noise robustness of TDA, our findings open perspectives towards establishing reliable topological and geometrical markers for group and possibly individual differences in functional brain network organization.

**Fig. 1:**
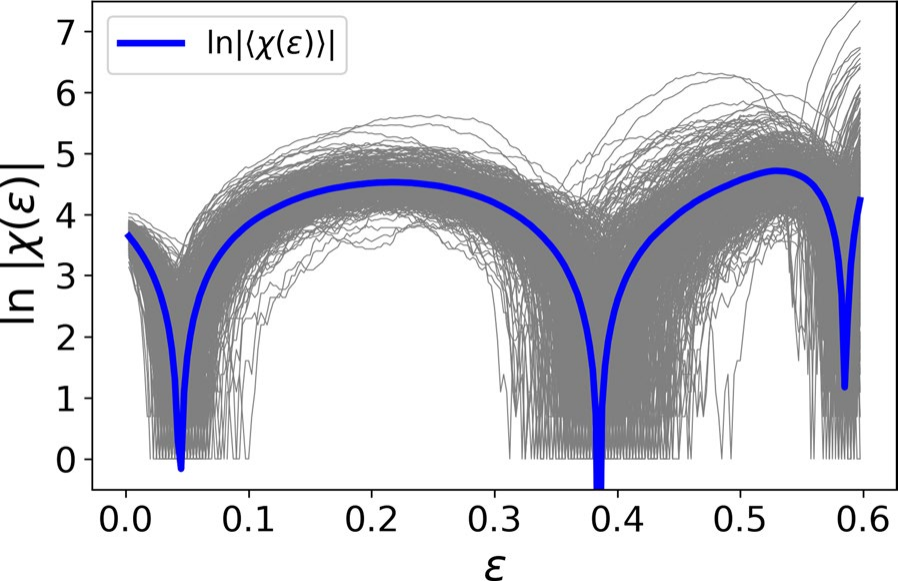
Topological phase transitions in functional brain networks. Euler entropy as a function of the correlation threshold level ε of functional brain networks from the HCP dataset. Each thin gray line represents an individual’s brain network, whereas the thick blueline depicts their average

## P73 A whole-brain spiking neural network model linking basal ganglia, cerebellum, cortex and thalamus

### Carlos Gutierrez^1^, Jun Igarashi^2^, Zhe Sun^2^, Hiroshi Yamaura^3^, Tadashi Yamazaki^4^, Markus Diesmann^5^, Jean Lienard^1^, Heidarinejad Morteza^2^, Benoit Girard^6^, Gordon Arbuthnott^7^, Hans Ekkehard Plesser^8^, Kenji Doya^1^

#### ^1^Okinawa Institute of Science and Technology, Neural Computation Unit, Okinawa, Japan; ^2^Riken, Computational Engineering Applications Unit, Saitama, Japan; ^3^The University of Electro-Communications, Tokyo, Japan; ^4^The University of Electro-Communications, Graduate School of Informatics and Engineering, Tokyo, Japan; ^5^Jülich Research Centre, Institute of Neuroscience and Medicine (INM-6) & Institute for Advanced Simulation (IAS-6), Jülich, Germany; ^6^Sorbonne Universite, UPMC Univ Paris 06, CNRS, Institut des Systemes Intelligents et de Robotique (ISIR), Paris, France; ^7^Okinawa Institute of Science and Technology, Brain Mechanism for Behaviour Unit, Okinawa, Japan; ^8^Norwegian University of Life Sciences, Faculty of Science and Technology, Aas, Norway

##### **Correspondence:** Carlos Gutierrez (carlos.gutierrez@oist.jp)

*BMC Neuroscience* 2019, **20(Suppl 1)**:P73

The neural circuit linking the basal ganglia, the cerebellum and the cortex through the thalamus plays an essential role in motor and cognitive functions. However, how such functions are realized by multiple loop circuits with neurons of multiple types is still unknown. In order to investigate the dynamic nature of the whole-brain network, we built biologically constrained spiking neural network models of the basal ganglia [1, 2, 3], cerebellum, thalamus, and the cortex [4, 5] and ran an integrated simulation on K supercomputer [8] using NEST 2.16.0 [6, 7, 9].

We replicated resting state activities of 1 biological second of time in models with increasing scales, from 1x1mm^2^ to 9x9mm^2^ of cortical surface, the latter of which includes 35 million neurons and 66 billion synapses in total. Simulations using a hybrid parallelization approach showed a good weak scaling performance in simulation time lasting between 15–30 minutes, but identified a problem of long time (between 6–9 hours) required for network building.

We also evaluated the properties of action selection with realistic topographic connections in the basal ganglia circuit in 2-D target reaching task and observed selective activation and inhibition of neurons in preferred directions in every nucleus leading to the output. Moreover, we performed tests of reinforcement learning based on dopamine-dependent spike-timing dependent synaptic plasticity.

**References**

1. Liénard J, Girard B. A biologically constrained model of the whole basal ganglia addressing the paradoxes of connections and selection. *Journal of computational neuroscience* 2014 Jun 1;36(3):445–68.
2. Liénard J, Girard B, Doya K, et al. Action selection and reinforcement learning in a Basal Ganglia model. *In Eighth International Symposium on Biology of Decision Making* 2018 (Vol. 6226, pp. 597–606). Springer.
3. Gutierrez CE, et al. Spiking neural network model of the basal ganglia with realistic topological organization. *Advances in Neuroinformatics* 2018, 10.14931/aini2018.ps.15 [Poster].
4. Igarashi J, Moren K, Yoshimoto J, Doya K. Selective activation of columnar neural population by lateral inhibition in a realistic model of primary motor cortex. *In Neuroscience 2014, the 44th Annual Meeting of the Society for Neuroscience (SfN 2014)* Nov 15th. [Poster].
5. Zhe S, Igarashi J. A Virtual Laser Scanning Photostimulation Experiment of the Primary Somatosensory Cortex. *In The 28th Annual Conference of the Japanese Neural Network Society* 2018 Oct (pp. 116). Okinawa Institute of Science and Technology.
6. Gewaltig MO, Diesmann M. Nest (neural simulation tool). *Scholarpedia* 2007 Apr 5;2(4):1430.
7. Linssen C, et al. NEST 2.16.0. *Zenodo* 2018. 10.5281/zenodo.1400175.
8. Miyazaki H, Kusano Y, Shinjou N, et al. Overview of the K computer system. *Fujitsu Sci. Tech. J.* 2012 Jul 1;48(3):302–9.
9. Jordan J, Ippen T, Helias M, et al. Extremely scalable spiking neuronal network simulation code: from laptops to exascale computers. *Frontiers in neuroinformatics* 2018 Feb 16;12:2.

## P74 Graph theory-based representation of hippocampal dCA1 learning network dynamics

### Giuseppe Pietro Gava^1^, Simon R Schultz^2^, David Dupret^3^

#### ^1^Imperial College London, Biomedical Engineering, London, United Kingdom; ^2^Imperial College London, London, United Kingdom; ^3^University of Oxford, Medical Research Council Brain Network Dynamics Unit, Department of Pharmacology, Oxford, United Kingdom

##### **Correspondence:** Giuseppe Pietro Gava (giuseppepietrogava@gmail.com)

*BMC Neuroscience* 2019, **20(Suppl 1)**:P74

Since the discovery of place and grid cells, the hippocampus has been attributed a particular sensitivity to the spatial-contextual features of memory and learning. A crucial area in these processes is the dorsal CA1 hippocampus region (dCA1), where both pyramidal cells and interneurons are found. The former are excitatory cells that display tuning to spatial location (place fields), whilst the latter regulate the network with inhibitory inputs. Graph theory gives us powerful tools for studying such complex systems by representing, analyzing and modelling the dynamics of hundreds of components (neurons) interacting together. Graph theory-based methods are employed by network neuroscience to yield insightful descriptions of neural networks dynamics [1].

Here, we propose a graph theory-based analysis of the dCA1 network, recorded from mice engaged in a condition place preference task. In this protocol the animals first explore a familiar environment (*fam*). Afterwards, it is introduced to two novel arenas (*pre/post*), which are later individually associated with different reward dispensers. We analyse electro-physiological data from 2 animals, 7 recording days combined, for a total of 617 putative pyramidal cells and 38 putative interneurons. To investigate the dynamics of the recorded network, we apply directed weighted graphs using a directional biophysically-inspired measure of the functional connectivity between each neuron.

As of now, we have limited our analysis to the dynamics of putative pyramidal cells in the network. As the task progresses and the animal learns the reward associations, we observe an overall increase in the average strength (*S*) of the network (S_pre = 0.41±0.08 / S_post = 0.78±0.09, mean ± s.e.m. normalized units). The average firing rate (*FR*), instead, peaks only during the first exploration of the novel environment and decreases thereafter—(FR_fam = 0.78±0.02 / FR_pre = 0.95±0.02 / FR_post = 0.82±0.02). Together with *S*, an overall decrease in the shortest path length (*PL*) in the network suggests that the system shifts towards a more small-world structure (PL_fam = 1±0 / PL_pre = 0.76±0.09 / PL_post = 0.61±0.10). This topology has been described to be more adaptive and efficient, thus fit to encode new information [2]. The evolution of the network during learning is also indicated by its Riemannian distance from the activity patterns evoked in *fam*. This measure increases from the exposition to *pre* (0.88±0.02) to the end of learning (0.98±0.01), decreases in *post* (0.91±0.02) and is at its minimum when *fam* is recalled (0.78±0.06). These results suggest that the evoked patterns in *pre* and *post* are similar, as they represent the same environment, even if they display different network activity measures (*S, FR, PL*). We hypothesize that these metrics might indicate the learning-related dynamics that favor the encoding of new information.

We are to integrate these findings with information measures at the individual neuron level. The finer structure of the network may be investigated: from changes in pyramidal cells’ spatial tuning, to diverse regulatory action of the interneuron population. Together, these analyses will provide us with an insightful picture of the dCA1 network dynamics during learning.

**References**

1. Bassett DS, Sporns O. Network neuroscience. *Nature neuroscience* 2017 Mar;20(3):353.
2. Bassett DS, Bullmore ED. Small-world brain networks. *The neuroscientist* 2006 Dec;12(6):512–23.

## P75 Measurement-oriented deep-learning workflow for improved segmentation of myelin and axons in high-resolution images of human cerebral white matter

### Predrag Janjic^1^, Kristijan Petrovski^1^, Blagoja Dolgoski^2^, John Smiley^3^, Panče Zdravkovski^2^, Goran Pavlovski^4^, Zlatko Jakjovski^4^, Natasa Davceva^4^, Verica Poposka^4^, Aleksandar Stankov^4^, Gorazd Rosoklija^5^, Gordana Petrushevska^2^, Ljupco Kocarev^6^, Andrew Dwork^5^

#### ^1^Macedonian Academy of Sciences and Arts, Research Centre for Computer Science and Information Technologies, Skopje, Macedonia; ^2^School of Medicine, Ss. Cyril and Methodius University Skopje, Institute of Pathology, Skopje, Macedonia; ^3^Nathan S. Kline Institute for Psychiatric Research, New York, United States of America; ^4^School of Medicine, Ss. Cyril and Methodius University, Institute of Forensic Medicine, Skopje, Macedonia; ^5^New York State Psychiatric Institute, Columbia University, Division of Molecular Imaging and Neuropathology, New York, United States of America; ^6^Macedonian Academy of Sciences and Arts, Skopje, Macedonia

##### **Correspondence:** Predrag Janjic (predrag.a.janjic@gmail.com)

*BMC Neuroscience* 2019, **20(Suppl 1)**:P75

**Background:** In CNS, the relationship between axon diameter and myelin thickness is more complex than in peripheral nerve. Standard segmentation of high-contrast electron micrographs (EM) segments the myelin accurately, but even in studies of regular, parallel fibers, this does not translate easily into measurements of individual axons and their myelin sheaths, Quantitative morphology of myelinated axons requires measuring the diameters of thousands of axons and the thickness of each axon’s myelin sheath. We describe here a procedure for automated refinement of segmentation and measurement of each myelinated axon and its sheath in EMs (11 nm/pixel) of arbitrarily oriented prefrontal white matter (WM) from human autopsies (Fig. 1A).

**Fig. 1:**
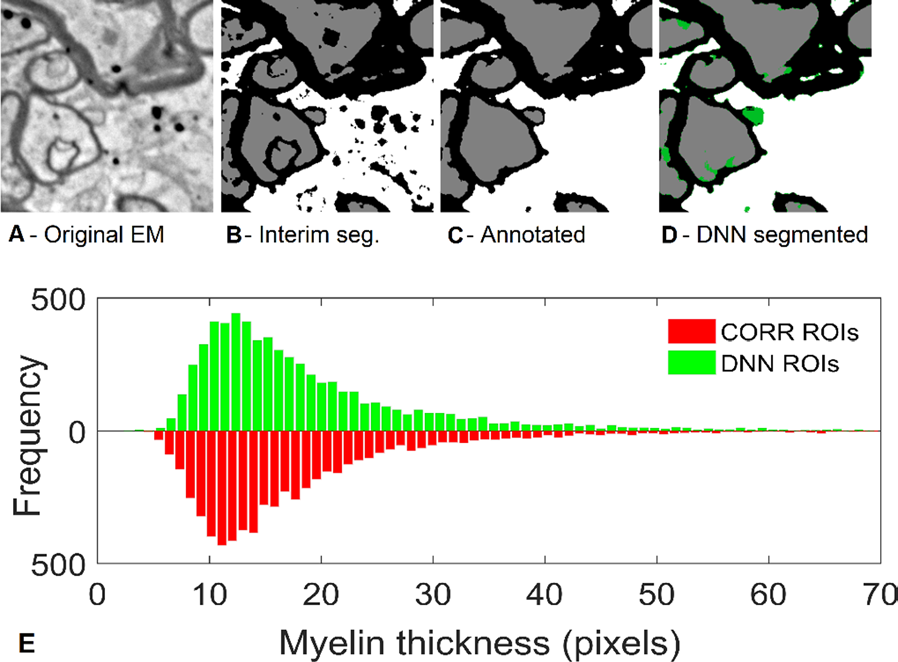
(Upper) **a** Fragment of original EM image has gone through automated pre-segmentation and automated post-processing producing Interim image **b** used as DNN input. **c** Fully corrected and annotated version used as “ground truth”. **d** DNN segmented fragment, with green pixels marking pixel errors compared to **c**. (lower) Histogram of myelin thickness measurements of a same dataset

**New methods:** Preliminary segmentation of myelin, axons and background in the original images, using ML techniques based on manually selected filters (Fig. 1B), are postprocessed for correcting of typical, systematic errors in the preliminary-segmentation. Final, refined and corrected segmentation is achieved by deep neural networks (DNN) which classify the central pixel of an input fragment (Fig. 1D). We use two DNN architectures: (i) *Denoising auto-encoder* using *convolutional neural network* (CNN) layers for initialization of weights to the first receptive layer of the main DNN, which is built in (ii) classical multilayer CNN architecture Automated routine gives radial measurements of each putative axon and its myelin sheath, after it rejects measures encountering predefined artifacts and excludes fibers that fail to satisfy certain predefined conditions. The ML processing, after a working dataset of 30 images, 2048x2048 pixel is preprocessed, takes ~ 1h 40min. for complete pixel-based segmentation of ~ 8,000 ÷ 9,000 fiber ROIs per set, on a commercial PC equipped with a single GTX-1080 class GPU.

**Results**: This routine improved segmentation of three sets of 30 annotated images (sets 1 and 2 from prefrontal white matter, while set 3 was from optic nerve), with DNN trained only with a subset of set 1 images. Total number of myelinated axons identified by the DNN differed from the human segmentation by 0.2%, 2.9%, and − 5.1% for sets 1–3, respectively. G-ratios differed by 2.96%, 0.74% and 2.83%. Myelin thickness measurements were even closer, Fig. 1E. Intraclass correlation coefficients between DNN and annotated segmentation, were mostly>0.9, indicating nearly interchangeable performance.

**Comparison with existing method(s):** Measurement-oriented studies of arbitrarily oriented fibers (appearing in single images) from human frontal white matter are rare. Published studies of spinal cord white matter or peripheral nerve typically measure *aggregated area* of myelin sheaths, allowing only an aggregate estimation of average g-ratio, assuming counterfactually that g-ratio is the same for all fibers. Thus, our method fulfills an important need.

**Conclusions:** Automated segmentation and measurement of axons and myelin is more complex than it appears initially. We have developed a feasible approach that has proven comparable to human segmentation in our tests so far, and the trained networks generalize very well on datasets other than those used in training.

**Acknowledgements:** This work has been funded by National Institutes of Health, NIMH under MH98786.

## P76 Spike latency reduction generates efficient encoding of predictions

### Pau Vilimelis Aceituno^1^, Juergen Jost^2^, Masud Ehsani^1^

#### ^1^Max Planck Institute for Mathematics in the Sciences, Cognitive group of Juergen Jost, Leipzig, Germany; ^2^Max Planck Institute for Mathematics in the Sciences, Leipzig, Germany

##### **Correspondence:** Pau Vilimelis Aceituno (pau.aceituno@mis.mpg.de)

*BMC Neuroscience* 2019, **20(Suppl 1)**:P76

Living organisms make predictions in order to survive, posing the question of how do brains learn to make those predictions. General models based on classical conditioning [1] assume that prediction performance is feed back into the predicting neural population. However, recent studies have found that sensory neurons without feedback from higher brain areas encode predictive information [4]. Therefore, a bottom-up process without explicit feedback should also generate predictions. Here we present such a mechanism through latency reduction, an effect of Synaptic Time-Dependent Plasticity (STDP) [3].

We study leaky-integrate and fire neurons with a refractory period (LIF), each one getting a fixed input spike train that is repeated many times. The weights of the synapses change following the Synaptic Time-Dependent Plasticity (STDP) with soft bounds. From this we use a variety of mathematical tools and simulations to create the following argument:

- Short Temporal Effects: We analyze how do postsynaptic spikes evolve, showing that a single postsynaptic spike reduces its latency
- Long Temporal Effects: We prove that the postsynaptic spike train becomes very dense at input onset and that the number of postsynaptic spikes reduces with the stimulus repetition.
- Coding: The concentration of inputs makes the code more efficient in metabolic and decoding terms.
- Predictions: STDP makes postsynaptic neurons fire at the onset of the input spike train, which might be before the stimulus if the input spike train includes a pre-stimulus clue, thus generating predictions.

We show here (Fig. 1) that STDP in combination with regularly timed presynaptic spikes generates postsynaptic codes that are efficient and explain how forecasting are phenomena that emerge in an unsupervised way with a simple mechanistic interpretation. We believe that this idea offers an interesting complement to classical supervised predictive coding schemes in which prediction errors are feed back into the coding neurons. Furthermore, the concentration of postsynaptic spikes at stimulus onset can be interpreted in information theoretical terms as a way to improve the code in terms of error-resilience. Finally, we speculate that the fact that the same mechanism can be used to generate predictions as well as improve the effectiveness and metabolic efficiency of the neural code might give insights into how the ability of the nervous system to forecast might have evolved.

**Fig. 1:**
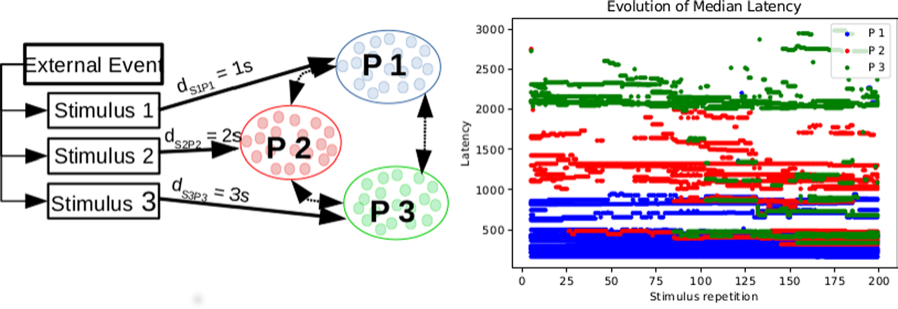
We show that STDP can lead to predictions through a schema where a single event generates stimulus S1, S2, S3 which trigger spikes on the neural populations P1 P2 P3. By STDP the spikes in P3 and P2 appear before the stimuli S2, S3

**References**

1. Dayan P, Abbott LF. Theoretical neuroscience: computational and mathematical modeling of neural systems. *MIT Press* 2001.
2. Song S, Miller KD, Abbott LF. Competitive Hebbian learning through spike-timing-dependent synaptic plasticity. *Nature neuroscience* 2000 Sep;3(9):919.
3. Guyonneau R, VanRullen R, Thorpe SJ. Neurons tune to the earliest spikes through STDP. *Neural Computation* 2005 Apr 1;17(4):859–79.
4. Palmer SE, Marre O, Berry MJ, Bialek W. Predictive information in a sensory population. *Proceedings of the National Academy of Sciences* 2015 Jun 2;112(22):6908–13.

## P77 Differential diffusion in a normal and a multiple sclerosis lesioned connectome with building blocks of the peripheral and central nervous system

### Oliver Schmitt^1^, Christian Nitzsche^1^, Frauke Ruß^1^, Lena Kuch^1^, Peter Eipert^1^

#### ^1^University of Rostock, Department of Anatomy, Rostock, Germany

##### **Correspondence:** Oliver Schmitt (schmitt@med.uni-rostock.de)

*BMC Neuroscience* 2019, **20(Suppl 1)**:P77

The structural connectome (SC) of the rat nervous system has been built by collating neuronal connectivity information from tract tracing publications [1]. In most publications semi quantitative estimates of axonal densities are indicated. These connectivity weights and the orientation of connections (source-target of action potentials) were imported into neuroVIISAS [2].

The connectivity of the peripheral nervous system and of the spinal cord allows a continuous reconstruction of the transfer of afferent signals from the periphery, respectively, dorsal root ganglions via intraspinal or medullary secondary neurons. As opposed to this the efferent pathway from the central peripheral nervous system (PNS) through primary vegetative neurons as well as α-motoneurons is available, too. This thorough connectome data allows the investigation of complete peripheral-central-afferents pathways as well as central-peripheral-efferent pathways by dynamic analyses.

The propagation of signals derived from basic diffusion processes [3], the Gierer-Meinhardt [4] and Mimura-Murray [5] diffusion models (DM) was investigated. The models have been adapted to a weighted and directed connectome. The application of DM in SCs exhibit a lower complexity by contrast with coupled single neuron models (FitzHugh Nagumo (FHN)) [3] or models of spiking LIF populations. To compare outcomes of DM the FHN model has been realized in the same SC (Fig. 1).

**Fig. 1:**
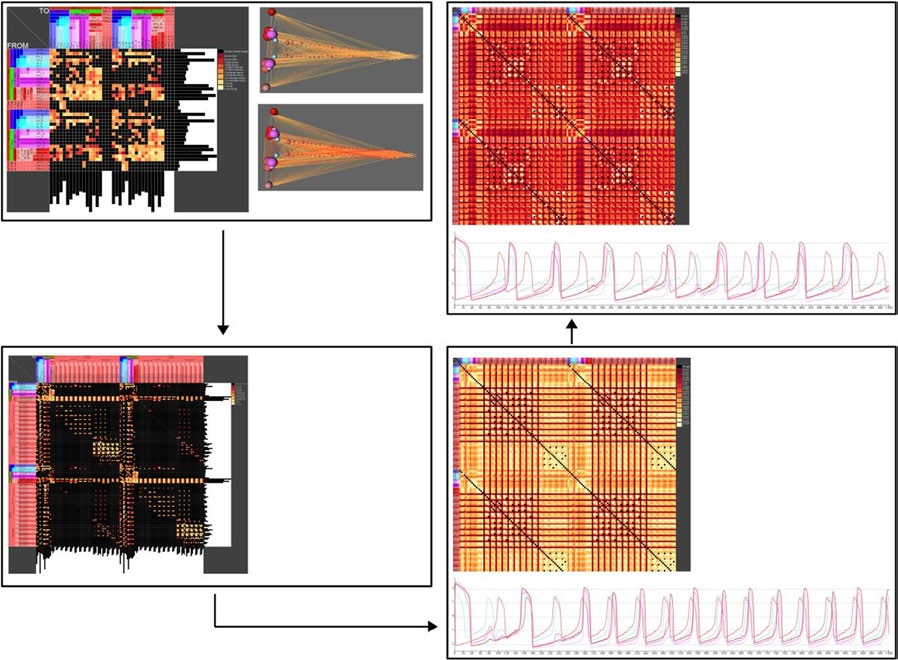
Visualization of bilateral weighted connectivity (upper left: adjacency matrix) of spinal and supraspinal regions (spherical 3D reconstruction). Upper right: Coactivation matrix of an FHN simulation. FHN oscillations of an afferent pathway. Lower left: Adjacency matrix of complete bilateral system. Coactivation matrix after simulating a MS demyelination

Modeling of diseases like Alzheimer and Parkinson as well as multiple sclerosis (MS) in SC helps to understand spreading of pathology and predicting changes of white and gray matter [6-8]. The reduction of connection weights by modelling reflect the effect of myelin degeneration in MS. The change [9] of diffusibility of a lesioned afferent-efferent loop in the rat PNS-ZNS has been analyzed. A reduction of diffusion was observed in the GM and MM models following linear and nonlinear reduction of connectivity weights of central processes of the dorsal root ganglion neurons, cuneate and gracile nuclei. The change of diffusibility shows slight effects in the motoric pathway.

The effects of the two models coincides with clinical observations with regard to paresthesias and spaticity because changes of diffusion were most prominent in the somatosensory and somatomotoric system. Further investigations will be performed to analyze functional effects of local white matter lesions as well as long term functional changes.

**References**

1. Schmitt O, Eipert P, Kettlitz R, Leßmann F, Wree A. The connectome of the basal ganglia. *Brain Structure and Function* 2016 Mar 1;221(2):753–814.
2. Schmitt O, Eipert P. neuroVIISAS: approaching multiscale simulation of the rat connectome. *Neuroinformatics* 2012 Jul 1;10(3):243–67.
3. Messé A, Hütt MT, König P, Hilgetag CC. A closer look at the apparent correlation of structural and functional connectivity in excitable neural networks. *Scientific reports* 2015 Jan 19;5:7870.
4. Gierer A, Meinhardt H. A theory of biological pattern formation. *Kybernetik* 1972 Dec 1;12(1):30–9.
5. Nakao H, Mikhailov AS. Turing patterns in network-organized activator–inhibitor systems. *Nature Physics* 2010 Jul;6(7):544.
6. Ji GJ, Ren C, Li Y, Sun J, et al. Regional and network properties of white matter function in Parkinson’s disease. *Human brain mapping* 2019 Mar;40(4):1253–63.
7. Ye C, Mori S, Chan P, Ma T. Connectome-wide network analysis of white matter connectivity in Alzheimer’s disease. *NeuroImage: Clinical* 2019 Jan 1;22:101690.
8. Mangeat G, Badji A, Ouellette R, et al. Changes in structural network are associated with cortical demyelination in early multiple sclerosis. *Human brain mapping* 2018 May;39(5):2133–46.
9. Schwanke S, Jenssen J, Eipert P, Schmitt O. Towards Differential Connectomics with NeuroVIISAS. *Neuroinformatics* 2019 Jan 1;17(1):163–79.

## P78 Linking noise correlations to spatiotemporal population dynamics and network structure

### Yanliang Shi^1^, Nicholas Steinmetz^2^, Tirin Moore^3^, Kwabena Boahen^4^, Tatiana Engel^1^

#### ^1^Cold Spring Harbor Laboratory, Cold Spring Harbor, NY, United States of America; ^2^University of Washington, Department of Biological Structure, Seattle, United States of America; ^3^Stanford University, Department of Neurobiology, Stanford, California, United States of America; ^4^Stanford University, Departments of Bioengineering and Electrical Engineering, Stanford, United States of America

##### **Correspondence:** Yanliang Shi (shi@cshl.edu)

*BMC Neuroscience* 2019, **20(Suppl 1)**:P78

Neocortical activity fluctuates endogenously, with much variability shared among neurons. These co-fluctuations are generally characterized as correlations between pairs of neurons, termed noise correlations. Noise correlations depend on anatomical dimensions, such as cortical layer and lateral distance, and they are also dynamically influenced by behavioral states, in particular, during spatial attention. Specifically, recordings from laterally separated neurons in superficial layers find a robust reduction of noise correlations during attention [1]. On the other hand, recordings from neurons in different layers of the same column find that changes of noise correlations differ across layers and overall are small compared to lateral noise-correlation changes [2]. Evidently, these varying patterns of noise correlations echo the wide-scale population activity, but the dynamics of population-wide fluctuations and their relationship to the underlying circuitry remain unknown.

Here we present a theory which relates noise correlations to spatiotemporal dynamics of population activity and the network structure. The theory integrates vast data on noise correlations with our recent discovery that population activity in single columns spontaneously transitions between synchronous phases of vigorous (On) and faint (Off) spiking [3]. We develop a network model of cortical columns, which replicates cortical On-Off dynamics. Each unit in the network represents one layer—superficial or deep—of a single column (Fig. 1a). Units are connected laterally to their neighbors within the same layer, which correlates On-Off dynamics across columns. Visual stimuli and attention are modeled as external inputs to local groups of units. We study the model by simulations and also derive analytical expressions for distance-dependent noise correlations. To test the theory, we analyze linear microelectrode array recordings of spiking activity from all layers of the primate area V4 during an attention task.

**Fig. 1:**
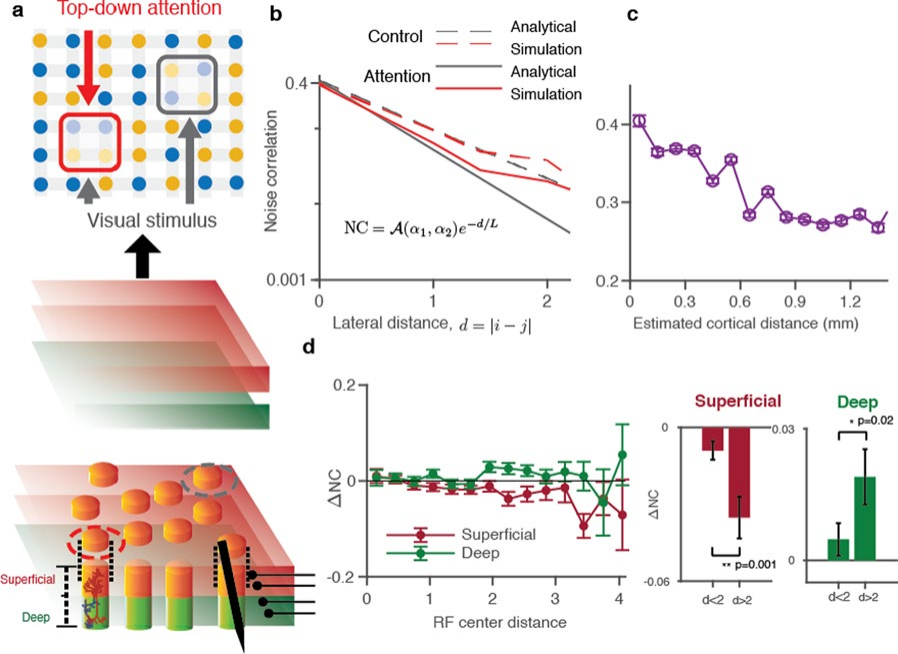
**a** Model architecture. A network of columns with lateral interactions represents one layer of cortical area V4. **b** The theory predicts that noise correlations decay exponentially with lateral distance. **c** Decrease of noise correlations with lateral distance in the laminar recordings. **d** Recordings show that during attention, noise correlations decrease in superficial and increase in deep layers

First, at the scale of single columns, the theory accurately predicts the broad distribution of attention-related changes of noise-correlations in our laminar recordings, indicating that they largely arise from the On-Off dynamics. Second, the network model mechanistically explains differences in attention-related changes of noise-correlations at different lateral distances. Due to spatial connectivity, noise correlations decay exponentially with lateral distance, characterized by the decay-constant called correlation length (Fig. 1b) . Correlation length depends on the strength of lateral connections, but it is also modulated by attentional inputs, which effectively regulate the relative influence of lateral inputs. Thus changes of lateral noise-correlations mainly arise from changes in the correlation length. The model predicts that at intermediate lateral distances (<1mm), noise-correlation changes decrease or increase with distance, when the correlation-length increases or decreases, respectively. To test these predictions, we used distances between receptive-field centers to estimate lateral shifts in our laminar recordings (Fig. 1c). We found that during attention, correlation length decreases in superficial and increases in deep layers, indicating differential modulation of superficial and deep layers. (Fig. 1d). Our work provides a unifying framework that links network mechanisms shaping noise correlations to dynamics of population activity and underlying cortical circuit structure.

**References**

1. Cohen MR, Maunsell JH. Attention improves performance primarily by reducing interneuronal correlations. *Nature neuroscience* 2009 Dec;12(12):1594.
2. Nandy AS, Nassi JJ, Reynolds JH. Laminar organization of attentional modulation in macaque visual area V4. *Neuron* 2017 Jan 4;93(1):235–46.
3. Engel TA, Steinmetz NA, Gieselmann MA, Thiele A, Moore T, Boahen K. Selective modulation of cortical state during spatial attention. *Science* 2016 Dec 2;3.

## P79 Modeling the link between optimal characteristics of saccades and cerebellar plasticity

### Hari Kalidindi^1^, Lorenzo Vannucci^1^, Cecilia Laschi^1^, Egidio Falotico^1^

#### ^1^Scuola Superiore Sant’Anna Pisa, The BioRobotics Institute, Pontedera, Italy

##### **Correspondence:** Hari Kalidindi (h.kalidindi@santannapisa.it)

*BMC Neuroscience* 2019, **20(Suppl 1)**:P79

Plasticity in cerebellar synapses is important for adaptability and fine tuning of fast reaching movements. The perceived sensory errors between the desired and actual movement outcomes are commonly considered to induce plasticity in the cerebellar synapses, with an objective to improve the desirability of the executed movements. In fast goal-directed eye movements called saccades, the desired outcome is to reach a given target location in minimum-time, with accuracy. However, an explicit encoding of this desired outcome is not observed in the cerebellar inputs prior to the movement initiation. It is unclear how the cerebellum is able to process only partial error information, that is the final reaching error signal obtained from sensors, to control both the reaching time as well as the precision of fast movements in an adaptive manner. We model the bidirectional plasticity at the parallel fiber to Purkinje cell synapses that can account for the mentioned saccade characteristics. We provide a mathematical and robot experimental demonstration of how the equations governing the cerebellar plasticity are determined by the desirability of the behavior. In the experimental results, the model output activity displays a definite encoding of eye speed and displacement during the movement. This is in line with the corresponding neurophysiological recordings of Purkinje cell populations in the cerebellar vermis of rhesus monkeys. The proposed modeling strategy, due to its mechanistic form, is suitable for studying the link between motor learning rules observed in biological systems and their respective behavioral principles.

## P80 Attractors and flows in the neural dynamics of movement control

### Paolo Del Giudice^1^, Gabriel Baglietto^2^, Stefano Ferraina^3^

#### ^1^Istituto Superiore di Sanità, Rome, Italy; ^2^IFLYSIB Instituto de Fisica de Liquidos y Sistemas Biologicos (UNLP-CONICET), La Plata, Argentina; ^3^Sapienza University, Dept Physiology and Pharmacology, Rome, Italy

##### **Correspondence:** Paolo Del Giudice (paolo.delgiudice@iss.it)

*BMC Neuroscience* 2019, **20(Suppl 1)**:P80

Density-based clustering (DBC) [1] provides efficient representations of a multidimensional time series, allowing to cast it in the form of the symbolic sequence of the labels identifying the cluster to which each vector of instantaneous values belong. Such representation naturally lends itself to obtain compact descriptions of data from multichannel electrophysiological recordings.

We used DBC to analyze the spatio-temporal dynamics of dorsal premotor cortex in neuronal data recorded from two monkeys during a ‘countermanding’ reaching task: the animal must perform a reaching movement to a target on a screen (‘no-stop trials’), unless an intervening stop signal prescribes to withhold the movement (‘stop-trials’); no-stop (~70%) and stop trials (~30%) were randomly intermixed, and the stop signal occurred at variable times within the reaction time.

Multi-unit activity (MUA) was extracted from signals recorded using a 96-electrodes array. Performing DBC on the 96-dimensional MUA time series, we derived the corresponding discrete sequence of clusters’ centroid.

Through the joint analysis of such cluster sequences for no-stop and stop trials we show that reproducible cluster sequences are associated with the completion of the motor plan in no-stop trials, and that in stop trials the performance depends on the relative timing of such states and the arrival of the Stop signal.

Besides, we show that a simple classifier can reliably predict the outcome of stop trials from the cluster sequence preceding the appearance of the stop signal, at the single-trial level.

We also observe that, consistently with previous studies, the inter-trial variability of MUA configurations typically collapses around the movement time, and has minima corresponding to other behavioral events (Go signal; Reward); comparing the time profile of MUA inter-trial variability with the cluster sequences, we are led to ask whether the neural dynamics underlying the clusters sequence can be interpreted as attractor hopping. For this purpose we analyze the flow in the MUA configuration space: for each trial, and each time, the measured MUA values identify a point in the 96-dimensional space, such that each trial corresponds to a trajectory in this space, and a set of repeated trials to a bundle of trajectories, of which we can compute individual or average properties. We measure quantities suited to discriminate between a dynamics of convergence of the trajectories to a point attractor, from different flows in the MUA configuration space. We tentatively conclude that convergent attractor relaxation dynamics (in attentive wait conditions, as before the Go or the Reward events) coexist with coherent flows (associated with movement onset), in which low inter-trial variability of MUA configurations corresponds to a collapse in the directions of velocities (with high magnitude of the latter), like the system entering a funnel.

The ‘delay task’ (Go signal comes with a variable delay after the visual target), allows to further check our interpretation of specific MUA configurations (clusters) as being associated with the completion of the motor plan. Preliminary analysis shows that pre-movement-related MUA cluster sequences during delay trials are consistent with those from other trial types, though their time course qualitatively differs in the two monkeys, possibly reflecting different computational options.

**Reference**

1. Baglietto G, Gigante G, Del Giudice P. Density-based clustering: A ‘landscape view’ of multi-channel neural data for inference and dynamic complexity analysis. *PloS one* 2017 Apr 3;12(4):e0174918.

## P81 Information transmission in delay-coupled neuronal circuits

### Jaime Sánchez Claros^1^, Claudio Mirasso^1^, Minseok Kang^2^, Aref Pariz^1^, Ingo Fischer^1^

#### ^1^Institute for Cross-Disciplinary Physics and Complex Systems, Palma de Mallorca, Spain; ^2^Institute for Cross-Disciplinary Physics and Complex Systems, Osnabrck University, Osnabrck, Germany

##### **Correspondence:** Jaime Sánchez Claros (js.claros27@gmail.com)

*BMC Neuroscience* 2019, **20(Suppl 1)**:P81

The information that we receive through our sensory system (e.g. sound, vision, pain, etc), needs to be transmitted to different regions of the brain for its processing. When these regions are sufficiently separated from each other, the latency in the communication can affect the synchronization state; it is possible that the regions synchronize in phase or out of phase, or even not synchronize [1]. These types of synchronization, when occur, can have important consequences in information transmission and processing [2].

Here we study the information transmission in a V and a circular motif (see Fig. 1). We initially use the Kuramoto model to describe the nodes dynamics and derive analytical stability solutions for the V-motif for different delays and coupling strengths among the neurons as well as different spiking frequencies. We then analyze the effect that a third connection would have on the stable solutions as we change its axonal delay and synaptic strength. For a more realistic model, we simulate the Hodgkin-Huxley neuron model. For the V-motif we find that the delay can play an important role in the efficiency of the signal transmission. When we introduce a direct connection between 1 and 3, we find changes in the stability conditions and so the efficacy of the information transmission. To distinguish between rate and temporal coding, we modulate one of the elements with low and high frequency signals, respectively, and investigate the signal transmission to the other neurons using delayed mutual information and delayed transfer entropy [3].

**Fig. 1:**
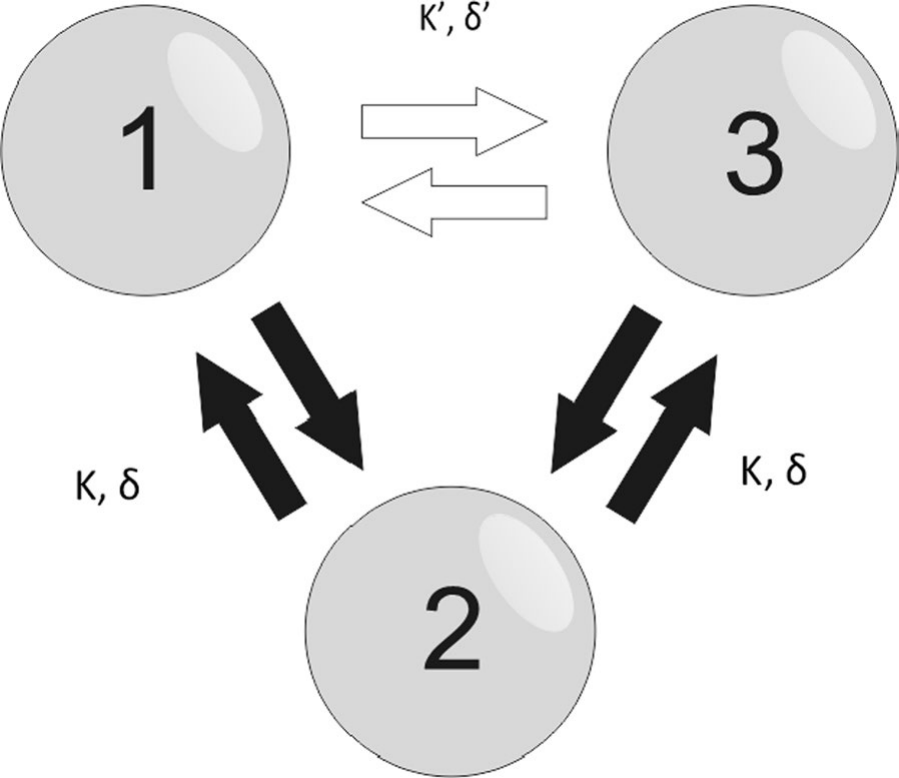
Three bidirectionally connected neurons. Two outer nodes (1 and 3) are bidirectionally connected to a middle node (2) with the same synaptic strength K and delay δ thus creating the V-motif. The addition of third bidirectional connection (white arrows) with synaptic strength K’ and delay δ’ between two outer nodes gives rise to the circular motif

**References**

1. Sadeghi S, Valizadeh A. Synchronization of delayed coupled neurons in presence of inhomogeneity. *Journal of Computational Neuroscience* 2014, 36, 55–66.
2. Mirasso CR, Carelli PV, Pereira T, Matias FS, Copelli M. Anticipated and zero-lag synchronization in motifs of delay-coupled systems. *Chaos* 2017, 27,114305.
3. Kirst C, Timme M, Battaglia D. Dynamic information routing in complex networks. *Nature communication* 2016, 7.

## P82 A Liquid State Machine pruning method for identifying task specific circuits

### Dorian Florescu

#### Coventry University, Coventry, United Kingdom

##### **Correspondence:** Dorian Florescu (dorian.florescu@coventry.ac.uk)

*BMC Neuroscience* 2019, **20(Suppl 1)**:P82

The current lack of knowledge on the precise neural circuits responsible for performing sensory and motor tasks, despite the large amounts of neuroscience data available, significantly slows down the development of new treatments for impairments caused by neurodegenerative diseases.

The Liquid State Machine (LSM) is one of the widely used paradigms for modelling brain computation. This model consists of a fixed recurrent spiking neural network, called Liquid, and a linear Readout unit with adjustable synapses. The model possesses, under idealised conditions, universal real-time computing power [1]. It was shown that, when the connections in the Liquid are modelled as dynamical synapses, this model can reproduce accurately the behaviour of the rat cortical microcircuits [1]. However, it is still largely unknown which neurons and synapses in the Liquid play a key role in a task performed by the LSM. Several proposed methods train the Liquid in addition to the Readout [2], which leads to improvements in accuracy and network sparsity, but offers little insight into the functioning of the original Liquid.

In the typical LSM architecture, the spike trains generated by the Liquid neurons are filtered before being processed by the Readout. It was shown that using the exact spike times generated by the Liquid neurons, rather than the filtered spike times, results in a much better performance of LSMs on training tasks. The algorithm introduced, called the Orthogonal Forward Regression with Spike Times (OFRST), leads to higher accuracy and fewer Readout connections than the state-of-the-art algorithm [3].

This work proposes an analysis of the underlying mechanisms used by the LSM to perform a computational task by searching for the key neural circuits involved. Given an LSM trained on a classification task, a new algorithm is introduced that identifies the corresponding task specific circuit (TSC), defined as the set of neurons and synapses in the Liquid that have a contribution to the Readout output. Thorough numerical simulations, I show that the TSC computed with the proposed algorithm has fewer neurons and higher performance when the training is done with OFRST compared with other state-of-the-art training methods (Fig. 1).

**Fig. 1:**
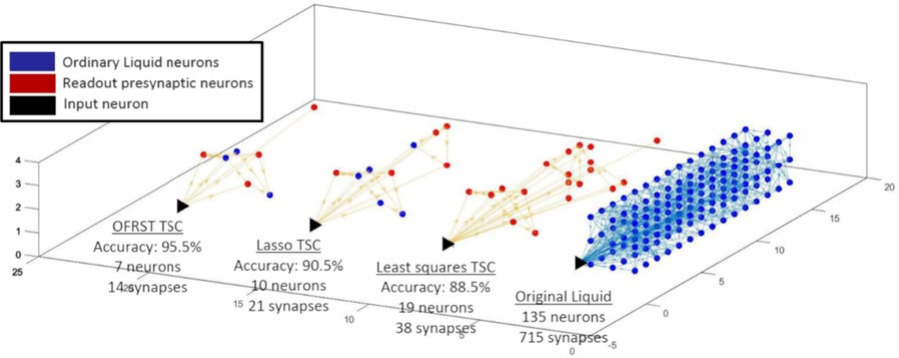
The task specific circuits (TSCs), computed with the proposed algorithm, corresponding to the classification task of discriminating jittered spike trains belonging to two classes. The training is done with three methods: OFRST, Least Squares, and Lasso. OFRST, the only method processing exact spike times, leads to the smallest circuit and the best performance on the validation dataset

I introduce a new representation for the Liquid dynamical synapses, which demonstrates that they can be mapped onto operators on the Hilbert space of spike trains. Based on this representation, I develop a novel algorithm that removes iteratively the synapses of a TSC based on the exact spike times generated by the Liquid neurons. Additional numerical simulations show that the proposed algorithm improves the LSM classification performance and leads to a significantly sparser representation. For the same initial Liquid, but different tasks, the proposed algorithm results in different TSCs that, in some cases, have no neurons in common. These results can lead to new methods to synthesize Liquids by interconnecting dedicated neural circuits.

**References**

1. Maass W, Natschläger T, Markram H. Computational models for generic cortical microcircuits. *Computational neuroscience: A comprehensive approach* 2004;18:575–605.
2. Yin J, Meng Y, Jin Y. A developmental approach to structural self-organization in reservoir computing. *IEEE transactions on autonomous mental development* 2012 Dec;4(4):273–89.
3. Florescu D, Coca D. Learning with precise spike times: A new approach to select task-specific neurons. *In Computational and Systems Neuroscience (COSYNE)* 2018 Mar 2. COSYNE.

## P83 Cross-frequency coupling along the soma-apical dendritic axis of model pyramidal neurons

### Melvin Felton^1^, Alfred Yu^2^, David Boothe^2^, Kelvin Oie^2^, Piotr Franaszczuk^2^

#### ^1^U.S. Army Research Laboratory, Computational and Information Sciences Division, Adelphi, MD, United States of America; ^2^U.S. Army Research Laboratory, Human Research and Engineering Directorate, Aberdeen Proving Ground, MD, United States of America

##### **Correspondence:** Piotr Franaszczuk (pfranasz@gmail.com)

*BMC Neuroscience* 2019, **20(Suppl 1)**:P83

Cross-frequency coupling (CFC) has been associated with mental processes like perceptual and memory-related tasks, and is often observed via EEG and LFP measurements [1]. There are a variety of physiological mechanisms believed to produce CFC, and different types of network properties can yield distinct CFC signatures [2]. While it is widely believed that pyramidal neurons play an important role in the occurrence of CFC, the detailed nature of the contribution of individual pyramidal neurons to CFC detected via large-scale measures of brain activity is still uncertain.

As an extension of our single model neuron resonance analysis [3], we examined CFC along the soma-apical dendrite axis of realistic models of pyramidal neurons. We configured three models to capture some variety that exists among pyramidal neurons in the neocortical and limbic regions of the brain. Our baseline model had the least amount of regional variation in conductance densities of the Ih and high- and low-threshold Ca2+ conductances. The second model had an exponential gradient in Ih conductance density along the soma-apical dendrite axis, typical of some neocortical and hippocampal pyramidal neurons. The third model contained both the exponential gradient in Ih conductance density and a distal apical “hot zone” where the high- and low-threshold Ca2+conductances had densities 10 and 100 times higher, respectively, than anywhere else in the model (cf., [3]). We simulated two current injection scenarios: 1) perisomatic 4 Hz modulation with perisomatic, mid-apical, and distal apical 40 Hz injections; and 2) distal 4 Hz modulation with perisomatic, mid-apical, and distal 40 Hz injections. We used two metrics to quantify the strength of CFC—height ratio and modulation index [4].

We found that CFC strength can be predicted from the passive filtering properties of the model neuron. Generally, regions of the model with much larger membrane potential fluctuations at 4 Hz than at 40 Hz (high Vm4Hz/Vm40Hz) had stronger CFC. The strongest CFC values were observed in the baseline model, but when the exponential gradient in Ih conductance density was added, CFC strength decreased by almost 50% at times. On the other hand, including the distal hot zone increased CFC strength slightly above the case with only the exponential gradient in Ih conductance density.

This study can potentially shed light on which configurations of fast and slow input to pyramidal neurons can produce the strongest CFC, and where exactly within the neuron CFC is strongest. In addition, this study can illuminate the reasons why there may be differences between CFC strength observed in different regions of the brain and between different populations of neurons.

**References**

1. Tort AB, Komorowski RW, Manns JR, Kopell NJ, Eichenbaum H. Theta–gamma coupling increases during the learning of item–context associations. *Proceedings of the National Academy of Sciences* 2009 Dec 8;106(49):20942–7.
2. Hyafil A, Giraud AL, Fontolan L, Gutkin B. Neural cross-frequency coupling: connecting architectures, mechanisms, and functions. *Trends in neurosciences* 2015 Nov 1;38(11):725–40.
3. Felton Jr MA, Alfred BY, Boothe DL, Oie KS, Franaszczuk PJ. Resonance Analysis as a Tool for Characterizing Functional Division of Layer 5 Pyramidal Neurons. *Frontiers in Computational Neuroscience* 2018;12.
4. Tort AB, Komorowski R, Eichenbaum H, Kopell N. Measuring phase-amplitude coupling between neuronal oscillations of different frequencies. *Journal of neurophysiology* 2010 May 12;104(2):1195–210.

## P84 Regional connectivity increases low frequency power and heterogeneity

### David Boothe, Alfred Yu, Kelvin Oie, Piotr Franaszczuk

#### U.S. Army Research Laboratory, Human Research and Engineering Directorate, Aberdeen Proving Ground, MD, United States of America

##### **Correspondence:** David Boothe (david.l.boothe7.civ@mail.mil)

*BMC Neuroscience* 2019, **20(Suppl 1)**:P84

The relationship between neuronal connectivity and frequency in the power spectrum of calculated local field potentials is poorly characterized in models of cerebral cortex. Here we present a simulation of cerebral cortex based on the Traub model [1] implemented in the GENESIS neuronal simulation environment. We found that this model tended to produce high neuronal firing rates and strongly rhythmic activity in response to increases in neuronal connectivity. In order to simulate spontaneous brain activity with a 1/f power spectrum as observed using electroencephalogram (EEG) (cf. [2]), and to faithfully recreate the sparse nature of cortical neuronal activity we re-tuned the original Traub parameters to eliminate intrinsic neuronal activity and removed the gap junctions. While gap junctions are known to exist in adult human cortex, their exact functional role in generating spontaneous brain activity is at present poorly characterized. Tuning out intrinsic neuronal activity allows changes to the synaptic connectivity to be central to changing overall model activity.

The model we present here consists of 16 simulated cortical regions each containing 976 neurons (15,616 neurons total). Simulated cortical regions are connected via short association fibers between adjacent cortical regions originating from pyramidal cells in cortical layer 2/3 (P23s). In the biological brain these short association fibers connect local cortical regions that tend to share a function like the myriad of visual areas of the posterior, parietal and temporal cortices [4]. Because of their ubiquity across cortex short association fibers were a natural starting point for our simulations. Long range layer 2/3 pyramidal cell connections terminated on neurons in other cortical regions with the same connectivity probabilities that they have locally within a region. We then varied the relative levels of long range and short-range connectivity and observed the impact on overall model activity. Because model dynamics were very sensitive to the overall number of connections we had to be careful that the simulations we were comparing only varied in proportion of long and short range connections and not in terms of total connectivity.

Our starting point for these simulations was a model with relatively sparse connectivity, which exhibited 1/f power spectrum with strong peaks in power spectral density at 20 Hz and 40 Hz ((Fig. 1), black line). We found that increases in long range connectivity increased power across the entire 1 to 100 Hz range of the overall local field potential of the model ((Fig. 1), blue line) and also increased heterogeneity in the power spectra of the 16 individual cortical regions. Increasing short range connectivity had the opposite effect, with overall power in the low frequency range (1 to 10 Hz) being reduced while the relative intensity at 20 Hz and 40 Hz remained constant (Fig. 1, red line). We will explore how consistent this effect is across varying levels of short- and long-range connectivity and model configuration.

**Fig. 1:**
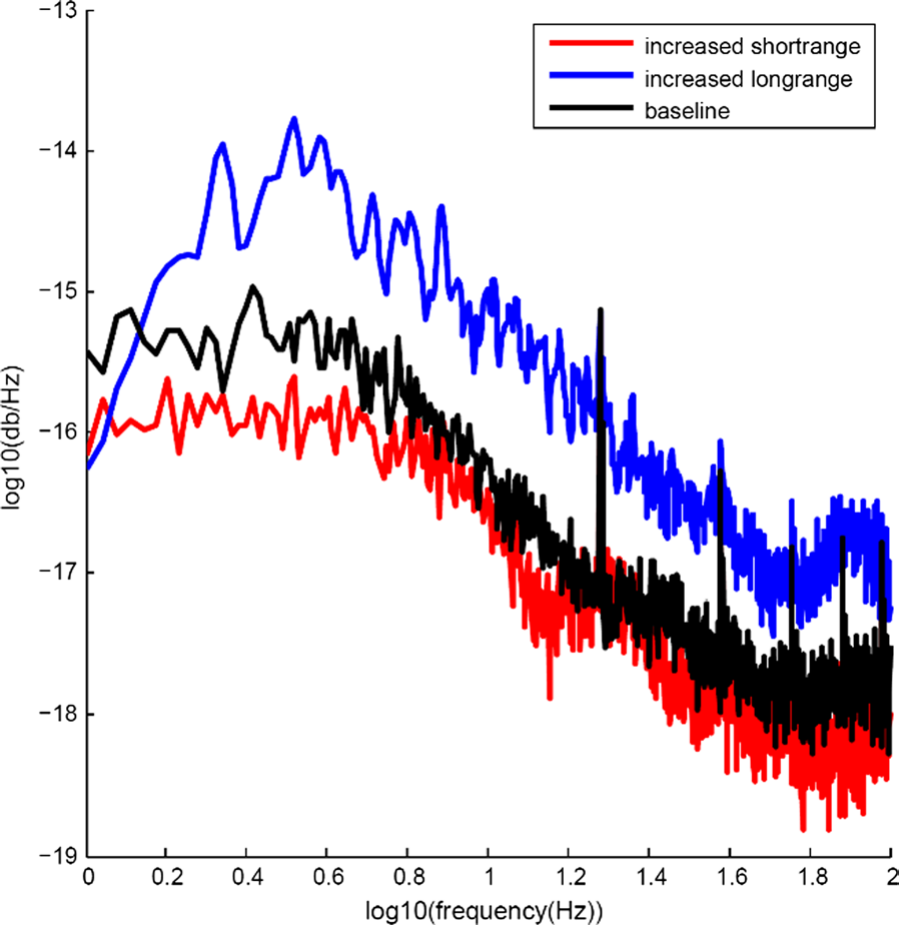
Differential impact of changes to short- and long-range connectivity. Black line shows power spectrum of model LFP. Blue line shows increase in LFP power across 1 to 100 Hz frequency range when long range connectivity is increased. Red line shows reduction in model power in 1 to 10 Hz range due to increase in short range connectivity

**References**

1. Traub RD, Contreras D, Cunningham MO, et al. Single-column thalamocortical network model exhibiting gamma oscillations, sleep spindles, and epileptogenic bursts. *Journal of neurophysiology* 2005 Apr;93(4):2194–232.
2. Le Van Quyen M. Disentangling the dynamic core: a research program for a neurodynamics at the large-scale. *Biological research* 2003;36(1):67–88.
3. Salin PA, Bullier J. Corticocortical connections in the visual system: structure and function. *Physiological reviews* 1995 Jan 1;75(1):107–54.

## P85 Cortical folding modulates the effect of external electrical fields on neuronal function

### Alfred Yu, David Boothe, Kelvin Oie, Piotr Franaszczuk

#### U.S. Army Research Laboratory, Human Research and Engineering Directorate, Aberdeen Proving Ground, MD, United States of America

##### **Correspondence:** David Boothe (david.l.boothe7.civ@mail.mil)

*BMC Neuroscience* 2019, **20(Suppl 1)**:P85

Transcranial electrical stimulation produces an electrical field that propagates through cortical tissue. Finite element modeling has shown that individual variation in spatial morphology can lead to variability in field strength within target structures across individuals [1]. Using GENESIS, we simulated a 10x10 mm network of neurons with spatial arrangements simulating microcolumns of a single cortical region spread across sulci and gyri. We modeled a transient electrical field with distance-dependent effects on membrane polarization, simulating the nonstationary effects of electrical fields on neuronal activity at the compartment level. In previous work, we have modeled applied electrical fields using distant electrodes, resulting in uniform orientation and field strength across all compartments. In this work, we examine a more realistic situation with distance- and orientation-dependent drop-off in field strength. As expected, this change resulted in a greater degree of functional variability between microcolumns and reduced overall network synchrony. We show that the spatial arrangement of cells within sulci and gyri yields sub-populations that are differentially susceptible to externally applied electric fields, in both their firing rates and the functional connectivity with adjacent microcolumns. In particular, pyramidal cell populations with inconsistently oriented apical dendrites produce less synchronized activity within an applied external field. Further, we find differences across cell types, such that cells with reduced dendritic arborization had greater sensitivity to orientation changes due to placement within sulci and gyri. Given that there is individual variability in the spatial arrangement of even primary cortices [2], our findings indicate that individual differences in outcomes of neurostimulation can be the result of variations in local topography. In summary, aside from increasing cortical surface area and altering axonal connection distances, cortical folding may additionally shape the effects of spatially local influences such as electrical fields.

**References**

1. Datta A. Inter-individual variation during transcranial direct current stimulation and normalization of dose using MRI-derived computational models. *Frontiers in psychiatry* 2012 Oct 22;3:91.
2. Rademacher J, Caviness Jr VS, Steinmetz H, Galaburda AM. Topographical variation of the human primary cortices: implications for neuroimaging, brain mapping, and neurobiology. *Cerebral Cortex* 1993 Jul 1;3(4):313–29.

## P86 Data-driven modeling of mouse CA1 and DG neurons

### Paola Vitale^1^, Carmen Alina Lupascu^1^, Luca Leonardo Bologna^1^, Mala Shah^2^, Armando Romani^3^, Jean-Denis Courcol^3^, Stefano Antonel^3^, Werner Alfons Hilda Van Geit^3^, Ying Shi^3^, Julian Martin Leslie Budd^4^, Attila Gulyas^4^, Szabolcs Kali^4^, Michele Migliore^1^, Rosanna Migliore^1^, Maurizio Pezzoli^5^, Sara Sáray^6^, Luca Tar^6^, Daniel Schlingloff^7^, Peter Berki^4^, Tamas F. Freund^4^

#### ^1^Institute of Biophysics, National Research Council, Palermo, Italy; ^2^UCL School of Pharmacy, University College London, School of Pharmacy, London, United Kingdom; ^3^École Polytechnique Fédérale de Lausanne, Blue Brain Project, Lausanne, Switzerland; ^4^Institute of Experimental Medicine, Hungarian Academy of Sciences, Budapest, Hungary; ^5^Laboratory of Neural Microcircuitry (LNMC),Brain Mind Institute, EPFL, Lausanne, Switzerland; ^6^Hungarian Academy of Sciences and Pázmány Péter Catholic University, Institute of Experimental Medicine and Information Technology and Bionics, Budapest, Hungary; ^7^Hungarian Academy of Sciences and Semmelweis University, Institute of Experimental Medicine and János Szentágothai Doctoral School of Neurosciences, Budapest, Hungary

##### **Correspondence:** Paola Vitale (paola.vitale@pa.ibf.cnr.it)

*BMC Neuroscience* 2019, **20(Suppl 1)**:P86

Implementing morphologically and biophysically accurate single cell models, capturing the electrophysiological variability observed experimentally, is the first crucial step to obtain the building blocks to construct a brain region at the cellular level.

We have previously applied a unified workflow to implement a set of optimized models of CA1 neurons and interneurons of rats [1]. In this work, we apply the same workflow to implement detailed single cell models of CA1 and DG mouse neurons. An initial set of kinetic models and dendritic distributions of the different ion channels present on each type of cells studied neurons was defined, consistently with the available experimental data. Many electrophysiological features were then extracted from a set of experimental traces obtained under somatic current injections. For this purpose, we used the eFEL tool available on the Brain Simulation Platform of the HBP (https://collab.humanbrainproject.eu/#/collab/1655/nav/66850). Interestingly, for both cell types we observed rather different firing patterns within the same cell population, suggesting that a given population of cells in the mouse hippocampus cannot be considered as belonging to a single firing type. For this reason, we have chosen to cluster the experimental traces on the basis of the number of spikes as a function of the current injection and optimize each group independently from the others. We identified four different types of firing behavior for both DG’s granule cells and CA1’s pyramidal neurons. To create the optimized models, we used the BluePyOpt Optimization library [2] with several different accurate morphologies. Simulations were run on HPC systems at Cineca, Jülich, and CSCS. The results of the models for CA1 and DG will be discussed also in comparison with the models obtained for the rat.

**References**

1. Migliore R, Lupascu CA, Bologna LL, et al. The physiological variability of channel density in hippocampal CA1 pyramidal cells and interneurons explored using a unified data-driven modeling workflow. *PLoS computational biology* 2018 Sep 17;14(9):e1006423.
2. Van Geit W, Gevaert M, Chindemi G, et al. BluePyOpt: leveraging open source software and cloud infrastructure to optimize model parameters in neuroscience. *Frontiers in neuroinformatics* 2016 Jun 7;10:17.

## P87 Memory compression in the hippocampus leads to the emergence of place cells

### Marcus K. Benna, Stefano Fusi

#### Columbia University, Center for Theoretical Neuroscience, Zuckerman Mind Brain Behavior Institute, New York, NY, United States of America

##### **Correspondence:** Marcus K. Benna (mkb2162@columbia.edu)

*BMC Neuroscience* 2019, **20(Suppl 1)**:P87

The observation of place cells in the hippocampus has suggested that this brain area plays a special role in encoding spatial information. However, several studies show that place cells do not only encode position in physical space, but that their activity is in fact modulated by several other variables, which include the behavior of the animal (e.g. speed of movement or head direction), the presence of objects at particular locations, their value, and interactions with other animals. Consistent with these observations, place cell responses are reported to be rather unstable, indicating that they encode multiple variables, many of which are not under control in experiments, and that the neural representations in the hippocampus may be continuously updated. Here we propose a memory model of the hippocampus that provides a novel interpretation of place cells and can explain these observations. We hypothesize that the hippocampus is a memory device that takes advantage of the correlations between sensory experiences to generate compressed representations of the episodes that are stored in memory. We have constructed a simple neural network model that can efficiently compress simulated memories. This model naturally produces place cells that are similar to those observed in experiments. It predicts that the activity of these cells is variable and that the fluctuations of the place fields encode information about the recent history of sensory experiences. Our model also suggests that the hippocampus is not explicitly designed to deal with physical space, but can equally well represent any variable with which its inputs correlate. Place cells may simply be a consequence of a memory compression process implemented in the hippocampus.

## P88 The information decomposition and the information delta: A unified approach to disentangling non-pairwise information

### James Kunert-Graf, Nikita Sakhanenko, David Galas

#### Pacific Northwest Research Institute, Galas Lab, Seattle, WA, United States of America

##### **Correspondence:** James Kunert-Graf (kunert@uw.edu)

*BMC Neuroscience* 2019, **20(Suppl 1)**:P88

Neurons in a network must integrate information from multiple inputs, and how this information is encoded (e.g. redundantly between multiple sources, or uniquely by a single source) is crucial to the understanding of how neuronal networks transmit information. Information theory provides robust measures of the interdependence of multiple variables, and recent work has attempted to disentangle the different types of interactions captured by these measures (Fig 1A).

**Fig. 1:**
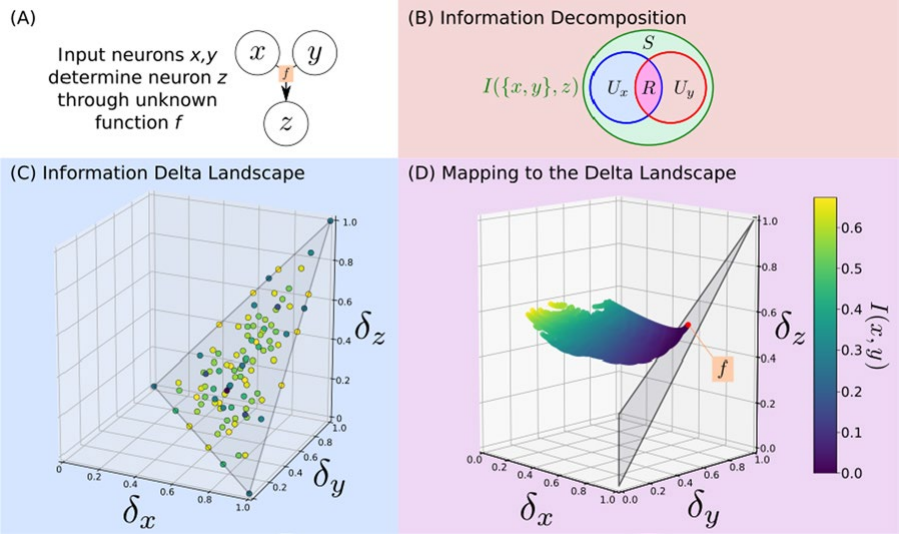
**a** Let x,y be neurons which determine z. **b** The Information Decomposition (ID) breaks information into unique, redundant and synergistic components. **c** Delta theory maps functions onto a space which encodes the ID. **d** [3] calculates the ID via an optimization which we map to delta-space, and is solved by the points from [6]. This identifies the function by which z integrates information

The Information Decomposition of Williams and Beer proposed decomposing the mutual information into unique, redundant, and synergistic components [1, 2]. This has been fruitfully applied, particularly in computational neuroscience, but there is no generally accepted method for its computation. Bertschinger et al. [3] developed one particularly rigorous approach, but it requires an intensive optimization over probability space (Fig 1B).

Independently, the quantitative genetics community has developed the Information Delta measures for detecting non-pairwise interactions for use in genetic datasets [4, 5, 6]. This has been exhaustively characterized for the discrete variables often found in genetics, yielding a geometric interpretation of how an arbitrary discrete function maps onto delta-space, and what its location therein encodes about the interaction (Fig 1C); however, this approach still lacks certain generalizations.

In this paper, we show that the Information Decomposition and Information Delta frameworks are largely equivalent. We identify theoretical advances in each that can be immediately applied towards answering questions open in the other. For example, we find that the results of Bertschinger et al. answer an open question in the Information Delta framework, specifically how to address the problem of linkage disequilibrium dependence in genetic data. We develop a method to computationally map the probability space defined by Bertschinger et al. into the space of delta measures, in which we can define a plane to which it is constrained with a well-defined optimum (Fig 1D). These optima occur at points in delta space which correspond to known discrete functions. This geometric mapping can thereby both side-step an expensive optimization and characterize the functional relationships between neurons. This unification of theoretical frameworks provides valuable insights for the analysis of how neurons integrate upstream information.

**References**

1. Williams PL, Beer RD. Nonnegative decomposition of multivariate information. *arXiv* 2010, arXiv:1004.2515.
2. Lizier JT, Bertschinger N, Jost J, Wibral M. Information decomposition of target effects from multi-source interactions: Perspectives on previous, current and future work. *Entropy* 2018, 20, 307.
3. Bertschinger N, Rauh J, Olbrich E, Jost J, Ay N. Quantifying unique information. *Entropy* 2014, 16, 2161–2183.
4. Galas D, Sakhanenko NA, Skupin A, Ignac T. Describing the complexity of systems: Multivariable “set complexity” and the information basis of systems biology. *J Comput Biol.* 2014, 2, 118–140.
5. Sakhanenko NA, Galas DJ. Biological data analysis as an information theory problem: Multivariable dependence measures and the shadows algorithm. *J Comput Biol.* 2015, 22, 1005–1024.
6. Sakhanenko NA, Kunert-Graf JM, Galas DJ. The information content of discrete functions and their application in genetic data analysis. *J Comp Biol.* 2017, 24, 1153–1178.

## P89 Homeostatic mechanism of myelination for age-dependent variations of axonal conductance speed in the pathophysiology of Alzheimer’s disease

### Maurizio De Pittà^1^, Giulio Bonifazi^1^, Tania Quintela-López^2^, Carolina Ortiz-Sanz^2^, María Botta^2^, Alberto Pérez-Samartín^2^, Carlos Matute^2^, Elena Alberdi^2^, Adhara Gaminde-Blasco^2^

#### ^1^Basque Center for Applied Mathematics, Group of Mathematical, Computational and Experimental Neuroscience, Bilbao, Spain; ^2^Achucarro Basque Center for Neuroscience, Leioa, Spain

##### **Correspondence:** Giulio Bonifazi (gbonifazi@bcamath.org)

*BMC Neuroscience* 2019, **20(Suppl 1)**:P89

The structure of white matter in patients affected by Alzheimer’s disease (AD) and age-related dementia, typically reveals aberrant myelination, suggesting that ensuing changes in axonal conduction speed could contribute to cognitive impairment and behavioral deficits observed in those patients. Experiments ex vivo in a murine model of AD confirm these observations but also pinpoint to multiple, coexisting mechanisms that could intervene in regulation and maintenance of integrity of myelinated fibers. Density of myelinated fibers in the corpus callosum indeed appears not to be affected by disease progression in transgenic mice whereas density of myelinating oligodendrocyte is increased with respect to wild-type animals. Significantly, this enhancement correlates with an increased expression of myelin basic protein (MBP); as well as with nodes of Ranvier that are shorter and more numerous; and a decrease in axonal conduction speed. We show that these results can be reproduced by a classical model of action potential propagation in myelinated axons by the combination of three factors that are: (i) a reduction of node length in association with (ii) an increase of both internode number and (iii) myelin thickness. In the simple scenario of two interacting neural populations where a recently-observed inhibitory feedback on the degree of myelination is incorporated as a function of synaptic connection disrupted by extracellular amyloid beta oligomers (Aβ1-42), we show that the reduction of axonal conduction speed by the concerted increase of Ranvier’s node number and myelin thickness accounts for minimizing the energetic cost of interacting population activity.

## P90 Collective dynamics of a heterogeneous network of active rotators

### Pablo Ruiz, Jordi Garcia-Ojalvo

#### Universitat Pompeu Fabra, Department of Experimental and Health Sciences, Barcelona, Spain

##### **Correspondence:** Pablo Ruiz (pabloruizibarrechevea@gmail.com)

*BMC Neuroscience* 2019, **20(Suppl 1)**:P90

We analyze the behavior of a network of active rotators [1] containing both oscillatory and excitable elements. We assume that the oscillatory character of the elements is continuously distributed. The system exhibits three main dynamical behaviors (i) a quiescent phase in which all elements are stationary, (ii) global oscillations in which all elements oscillate in a synchronized manner, and (iii) partial oscillations in which a fraction of the units oscillates, partially synchronized among them (analogous to the case in [2]). We also observe that the pulse duration is shorter for the excitable units than for the oscillating ones, even though the former has smaller intrinsic frequencies than the latter. Apart from the standard usage of the Kuramoto order parameter (or its variance) as a measure of synchrony, and consequently, as a measure of the macroscopic state of the system, we are interested in finding an observable that helps gain insight on what is the position within a hierarchy of states. We can call this measure the potential or energy of the system, and define it as the integral over the phases, by gradient dynamics [3]. This variable can be considered as a measure of multistability. We also study more complex coupling situations, included the existence of negative links between coupled elements in a whole-brain network, mimicking the inhibitory connections present in the brain.

**References**

1. Sakaguchi H, Shinomoto S, Kuramoto Y. Phase transitions and their bifurcation analysis in a large population of active rotators with mean-field coupling. *Progress of Theoretical Physics* 1988 Mar 1;79(3):600–7.
2. Pazó D, Montbrió E. Universal behavior in populations composed of excitable and self-oscillatory elements. *Physical Review E* 2006 May 31;73(5):055202.
3. Ionita F, Labavić D, Zaks MA, Meyer-Ortmanns H. Order-by-disorder in classical oscillator systems. *The European Physical Journal B* 2013 Dec 1;86(12):511.

## P91 A hidden state analysis of prefrontal cortex activity underlying trial difficulty and erroneous responses in a distance discrimination task

### Danilo Benozzo^1^, Giancarlo La Camera^2^, Aldo Genovesio^1^

#### ^1^Sapienza University of Rome, Department of Physiology and Pharmacology, Rome, Italy; ^2^Stony Brook University, Department of Neurobiology and Behavior, Stony Brook, NY, United States of America

##### **Correspondence:** Danilo Benozzo (danilo.benozzo@uniroma1.it)

*BMC Neuroscience* 2019, **20(Suppl 1)**:P91

Previous studies have established the involvement of prefrontal cortex (PFC) neurons in the decision process during a distance discrimination task. However, no single-neuron correlates of important task variables such as trial difficulty was found. Here, we perform a trial-by-trial analysis of ensembles of simultaneously recorded neurons, specifically, multiple single-unit data from two rhesus monkeys performing the distance discrimination task. The task consists in the sequential presentation of two visual stimuli (S1 and S2, in this order) separated by a temporal delay. The monkeys had to report which stimulus was farthest from a reference point after a GO signal consisting in the presentation of the same two stimuli in the two sides of the screen. Six stimulus distances were tested (from 8 to 48mm), generating five levels of difficulty, each measured as the difference |S2-S1| between the relative positions of the stimuli (difficulty increases with |S2-S1|).

We analyzed the neural ensemble data with a Poisson hidden Markov model (HMM). A Poisson-HMM describes the activity of each single trial by a sequence of vectors of firing rates across simultaneously recorded neurons. Each vector of firing rates is a metastable ‘state’ of the neural activity. HMM allows to identify changes in neural state independently of external triggers, which have previously been linked to states of attention, expectation and decision making, to name a few.

For each experimental session, we fit the HMM to the neural ensemble starting from random initial conditions and different numbers of states (between 2 and 5) using maximum likelihood (Baum-Welch algorithm). The fitting procedure was repeated 5 times with new random initial conditions until a convergence criterion was reached (capped at 500 iterations). The model with the smallest BIC was selected as the best model. Post-fitting, a state was assigned to each 5ms bin of data if its posterior probability given the data exceeded 0.8. To further avoid overfitting, only states exceeding 0.8 for at least 50 consecutive ms were kept.

First, we looked for a relationship between trial difficulty and the first state transition time after S2 presentation. We found that faster state transitions occurred in easier trials (Fig. 1a), but no correlation was found with first transition times after the GO signal. This demonstrates that task difficulty modulates the neural dynamics during decisions, and this modulation occurs in the deliberation phase and is absent when the monkeys convert the decision into action.

**Fig. 1:**
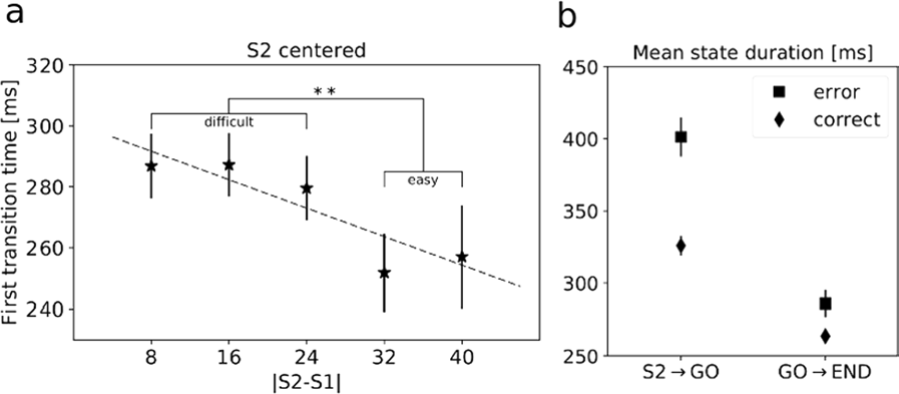
**a** First transition time after S2 evaluated across trial difficulties |S2-S1|. Higher |S2-S1| means lower difficulty. Dashed line is the linear regression interpolation (p<0.05). **=p<0.01, Welch’s t-test. **b** Mean state duration computed before and after the GO signal on correct and incorrect trials (2-way ANOVA, significant interaction and effects of trial type and time interval; p<0.001)

Second, we found that RTs were significantly longer in error trials compared to correct trials overall (p<0.001, t-test). Thus, we investigated the relationship between reaction times and neural dynamics. We focused on a larger time window, from 400 ms before S2 until the beginning of the following trial, in order to better capture the whole dynamics of state transitions. We found longer mean state durations after S2 in error trials compared to correct trials (Fig. 1b), a signature of a slowing down of cortical dynamics during error trials. The effect was largest in the period from S2 to the GO signal, i.e., during the deliberation period (2-way ANOVA, interaction term, p<0.001). These results indicate a global slowdown of the neural dynamics prior to errors as the neural substrate of longer reaction times during incorrect trials.

## P92 Neural model of the visual recognition of social interactions

### Mohammad Hovaidi-Ardestani, Martin Giese

#### Hertie Institute for Clinical Brain Research, Centre for Integrative Neuroscience, Department of Cognitive Neurology, University Clinic Tübingen, Tübingen, Germany

##### **Correspondence:** Mohammad Hovaidi-Ardestani (mohammad.hovaidi-ardestani@uni-tuebingen.de)

*BMC Neuroscience* 2019, **20(Suppl 1)**:P92

Humans are highly skilled at interpreting intent or social behavior from strongly impoverished stimuli [1]. The neural circuits that derive such judgements from image sequences are entirely unknown. It has been hypothesized that this visual function is based on high-level cognitive processes, such as probabilistic reasoning. Taking an alternative approach, we show that such functions can be accomplished by relatively elementary neural networks that can be implemented by simple physiologically plausible neural mechanisms, forming a hierarchical (deep) neural model of the visual pathway.

**Methods:** Extending classical biologically-inspired models for object and action perception [2, 3] and alternatively a front-end that exploits a deep learning model (VGG16) for the construction of low and mid-level feature detectors, we built a hierarchical neural model that reproduces elementary psychophysical results on animacy and social perception from abstract stimuli. The lower hierarchy levels of the model consist of position-variant neural feature detectors that extract orientation and intermediately complex shape features. The next-higher level is formed by shape-selective neurons that are not completely position-invariant, which extract the 2D positions and orientation of moving agents. A second pathway analyses the 2D motion of the moving agents, exploiting motion energy detectors. Exploiting a gain-field network, we compute the relative positions of the moving agents and analyze their relative motion. The top layers of the model combine the mentioned features that characterize the speed and smoothness of motion, and spatial relationships of the moving agents. The highest level of the model consists of neurons that compute the perceived agency of the motions, and that classify different categories of social interactions.

**Results:** Based on input video sequences, the model successfully reproduces results of [4] on the dependence of perceived animacy on motion parameters, and its dependence on the alignment of motion and body axis. The model reproduces the fact that a moving figure that with a body axis, like a rectangle, result in stronger perceived animacy than a moving circle if the body axis is aligned with the motion direction. In addition, the model classifies different interactions from abstract stimuli, including six categories of social interactions that have been frequently tested in the psychophysical literature (following, fighting, chasing, playing, guarding, and flirting) (e.g. [5, 6]).

**Conclusion:** Using simple physiologically plausible neural circuits, the model accounts simultaneously for a variety of effects related to animacy and social interaction perception. Even in its simple form the model proves that animacy and social interaction judgements partly can be derived by very elementary operations within a hierarchical neural vision system, without a need of sophisticated probabilistic inference mechanisms. The model makes precise predictions about the tuning properties of different types of neurons that should be involved in the visual processing of such stimuli. Such predictions might serve as starting point for physiological experiments that investigate the correlate of the perceptual processing of animacy and interaction at the single-cell level.

**References**

1. Heider F, Simmel M. An experimental study of apparent behavior. *The American journal of psychology* 1944 Apr 1;57(2):243–59. 10.2307/1416950
2. Riesenhuber M, Poggio T. Hierarchical models of object recognition in cortex. *Nature neuroscience* 1999 Nov;2(11):1019. 10.1038/14819
3. Giese MA, Poggio T. Cognitive neuroscience: neural mechanisms for the recognition of biological movements*. Nature Reviews Neuroscience* 2003 Mar;4(3):179. 10.1038/nrn1057
4. Tremoulet PD, Feldman J. Perception of animacy from the motion of a single object. *Perception* 2000 Aug;29(8):943–51.
5. Gao T, Scholl BJ, McCarthy G. Dissociating the detection of intentionality from animacy in the right posterior superior temporal sulcus. *Journal of Neuroscience* 2012 Oct 10;32(41):14276–80.
6. McAleer P, Pollick FE. Understanding intention from minimal displays of human activity. *Behavior Research Methods* 2008 Aug 1;40(3):830–9. 10.3758/BRM.40.3.830

## P93 Learning of generative neural network models for EMG data constrained by cortical activation dynamics

### Alessandro Salatiello, Martin Giese

#### Center for Integrative Neuroscience & University Clinic Tübingen, Dept of Cognitive Neurology, Tübingen, Germany

##### **Correspondence:** Alessandro Salatiello (alessandro.salatiello@uni-tuebingen.de)

*BMC Neuroscience* 2019, **20(Suppl 1)**:P93

Recurrent Artificial Neural Networks (RNNs) are popular models for neural structures in motor control. A common approach to build such models is to train RNNs to reproduce the input-output mapping of biological networks. However, this approach suffers from the problem that the internal dynamics of such networks are typically highly under-constrained: even though they correctly reproduce the desired input-output behavior, their internal dynamics are not under control and usually deviate strongly from those of real neurons. Here, we show that it is possible to accomplish the dual goal of both reproducing the target input-output behavior and constraining the internal dynamics to be similar to the ones of real neurons. As a test-bed, we simulated an 8-target reaching task; we assumed that a network of 200 primary motor cortex (M1) neurons generates the necessary activity to perform such tasks in response to 8 different inputs and that this activity drives the contraction of 10 different arm muscles. We further assumed to have access to only a sample of M1 neurons (30%) and relevant muscles (40%). In particular, we first generated multiphasic EMG-like activity by drawing samples from a Gaussian process. Secondly, we generated ground truth M1-like activity by training a stability-optimized circuit (SOC) network [2] to reproduce the EMG activity through gain modulation [1]. Finally, we trained two RNN models with the full-FORCE method [3] to reproduce the subset of observed EMG activity; critically, while one of the networks (FF) was free to reach such a goal through the generation of arbitrary dynamics, the other (FFH) was constrained to do so by generating, through its recurrent dynamics, activity patterns resembling those of the observed SOC neurons. To assess the similarity between the activities of FF, FFH and SOC neurons, we applied canonical correlation analysis (CCA) on the latent factors extracted through PCA. This analysis revealed that while both the FF and FFH network were able to reproduce the EMG activities accurately, the FFH network, that is the one with constrained internal dynamics, showed a greater similarity in the neural response space with the SOC network. Such similarity is noteworthy since the sample used to constrain the internal dynamics was small. Our results suggest that this approach might facilitate the design of neural network models that bridge multiple hierarchical levels in motor control, at the same time including details of available single-cell data.

**Acknowledgements:** Funding from BMBF FKZ 01GQ1704, DFG GZ: KA 1258/15-1; CogIMon H2020 ICT-644727, HFSP RGP0036/2016, KONSENS BW Stiftung NEU007/1

**References**

1. Stroud JP, Porter MA, Hennequin G, Vogels TP. Motor primitives in space and time via targeted gain modulation in cortical networks. *Nature Neuroscience* 2018 Dec;21(12):1774.
2. Hennequin G, Vogels TP, Gerstner W. Optimal control of transient dynamics in balanced networks supports generation of complex movements. *Neuron* 2014 Jun 18;82(6):1394–406.
3. DePasquale B, Cueva CJ, Rajan K, Abbott LF. full-FORCE: A target-based method for training recurrent networks. *PloS One* 2018 Feb 7;13(2):e0191527.

## P94 A neuron can make reliable binary, threshold gate like, decisions if and only if its afferents are synchronized.

### Timothee Masquelier^1^, Matthieu Gilson^2^

#### ^1^CNRS, Toulouse, France; ^2^Universitat Pompeu Fabra, Center for Brain and Cognition, Barcelona, Spain

##### **Correspondence:** Timothee Masquelier (timothee.masquelier@cnrs.fr)

*BMC Neuroscience* 2019, **20(Suppl 1)**:P94

Binary decisions are presumably made by weighing and comparing evidence, which can be modeled using the threshold gate formalism: the decision depends on whether or not a weighted sum of input variables *S* exceeds a threshold *θ*. Incidentally, this is exactly how the first neuron model proposed by McCulloch and Pitts in 1943, and later used in the perceptron, worked. But can biological neurons implement such a function, assuming that the input variables are the afferent firing rates? This matter is unclear, because biological neurons deal with spikes, not firing rates.

We investigated this issue through analytical calculations and numerical simulations, using a leaky integrate-and-fire (LIF) neuron (with *τ*  =  10 ms). The goal was to adjust the LIF’s threshold so that it fires at least one spike over a period *T* if *S*>*θ* (“positive condition”), and none otherwise (“negative condition”). We considered two different regimes: input spikes were either asynchronous (*i.e.*, latencies were uniformly distributed over [0; *T*]), or synchronous. In the latter case, the spikes arrived in discrete periodic volleys (with frequency *fo*), and with a certain dispersion inside each volley (*σ*). As Fig. 1 Top shows, in the asynchronous regime any threshold will lead to false alarms and/or misses. Conversely, in the synchronous regime, it is possible to set a threshold that will be reached in the positive condition, but not in the negative one.

**Fig. 1:**
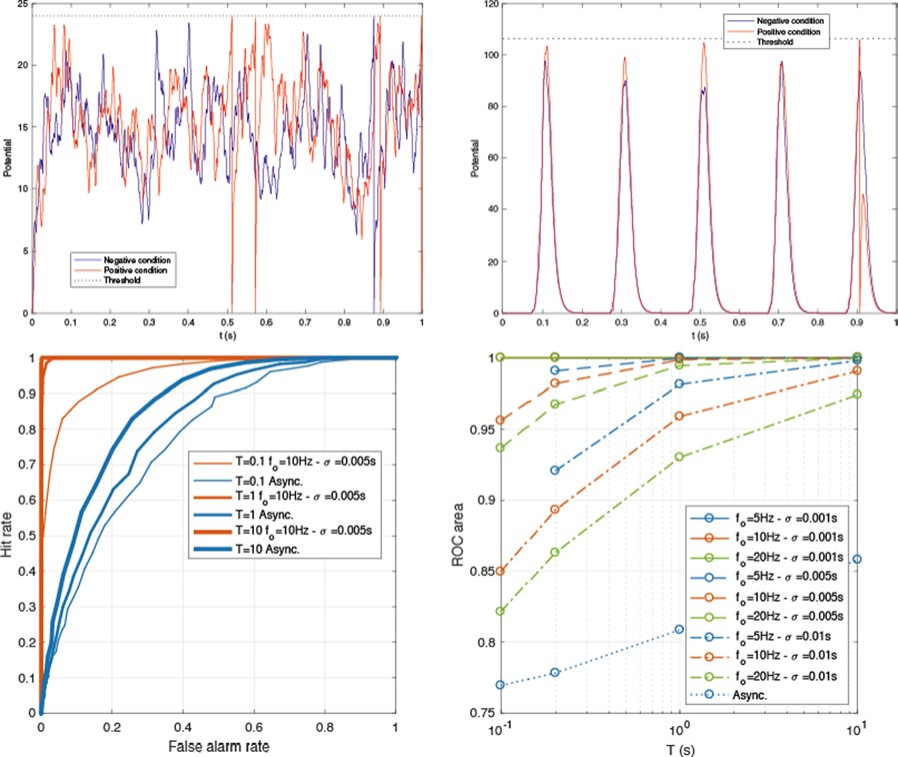
(Top left) The asynchronous regime. Threshold = 24 causes a hit for the positive condition, but also a false alarm for the negative one. (Top right) The synchronous regime. Threshold = 105 causes a hit for the positive condition, and no false alarm for the negative one. (Bottom left) Examples of ROC curves. (Bottom right) ROC area as a function of T, in the asynchronous and synchronous conditions

To demonstrate this more rigorously, we computed the receiver operating characteristic (ROC) curve as a function of *T* in both regimes (Fig. 1 Bottom). For the synchronous regime, we varied *f*o and *σ*. In short, the asynchronous regime leads to poor accuracy, which increases with *T*, but very slowly. Conversely, the synchronous regime leads to much better accuracy, which increases with *T*, but decreases with *σ* and *fo*.

In conclusion, if the decision needs to be taken in a reasonable amount of time, only the synchronous regime is viable, and the precision of the synchronization should be in the millisecond range. We are now exploring more biologically realistic regimes in which only a subset of the afferents is synchronized, in between the two extreme examples in Fig. 1. In the brain, the required synchronization could come from abrupt changes in the environment (*e.g.*, stimulus onset), active sampling (*e.g.*, saccades and microsaccades, sniffs, licking, touching), or endogenous brain oscillations. For example, rhythms in the beta or gamma ranges that correspond to different values for *f*o lead to different efficiency in our scheme for transmitting information, which implies constraints on the volley precision *σ*.

## P95 Unifying network descriptions of neural mass and spiking neuron models and specifying them in common, standardized formats

### Jessica Dafflon^1^, Angus Silver^2^, Padraig Gleeson^2^

#### ^1^King’s College London, Centre for Neuroimaging Sciences, London, United Kingdom; ^2^University College London, Dept. of Neuroscience, Physiology & Pharmacology, London, United Kingdom

##### **Correspondence:** Padraig Gleeson (p.gleeson@ucl.ac.uk)

*BMC Neuroscience* 2019, **20(Suppl 1)**:P95

Due to the inherent complexity of information processing in the brain, many different approaches have been taken to creating models of neural circuits, each making different choices about the level of biological detail to incorporate and the mathematical/analytical tractability of the models. Some approaches favour investigating large scale, brain wide behaviour with interconnected populations, each representing the activity of many neurons. Others include many of the known biophysical details of the constituent cells, down to the level of ion channel kinetics. These different approaches often lead to disjointed communities investigating the same system from very different perspectives. There is also an important issue of different simulation technologies being used in each of these communities (e.g. The Virtual Brain; NEURON), further preventing exchange of models and theories.

To address these issues, we have extended the NeuroML model specification language [1, 2], which already supports descriptions of networks of biophysically complex, conductance based cells, to allow descriptions of population units where the average activity of the cells is given by a single variable. With this, it is possible to describe classic models such as that of Wilson and Cowan [3] in the same format as more detailed models. To demonstrate the utility of this approach we have converted a recent large-scale network model of the macaque cortex [4] into NeuroML format. This model features the interaction between the feedforward and feedback signalling across multiple scales. In particular, interactions inside cortical layers, between layers, between areas and at the whole cortex level are simulated. With the NeuroML implementation we were able to replicate the main findings described in the original paper.

Compatibility with NeuroML comes with other advantages, particularly the ability to visualise the structure of models in the format in 3D on Open Source Brain [5] as well as analyse the network connectivity and run and replay simulations (http://www.opensourcebrain.org/projects/mejiasetal2016). This extension to NeuroML for neural mass models, the support in compatible tools and platforms and example networks in this format will help enable sharing, comparison and reuse of models between researchers taking diverse approaches to understanding the brain.

**References**

1. Cannon RC, Gleeson P, Crook S, Ganapathy G, Marin B, Piasini E, et al. LEMS: A language for expressing complex biological models in concise and hierarchical form and its use in underpinning NeuroML 2*. Frontiers in neuroinformatics* 2014;8. 10.3389/fninf.2014.00079
2. Gleeson P, Crook S, Cannon RC, Hines ML, Billings GO, Farinella M, et al. NeuroML: A Language for Describing Data Driven Models of Neurons and Networks with a High Degree of Biological Detail*. PLoS Comput Biol. Public Library of Science* 2010;6: e1000815.
3. Wilson HR, Cowan JD. Excitatory and inhibitory interactions in localized populations of model neurons. *Biophys J.* 1972;12: 1–24.
4. Mejias JF, Murray JD, Kennedy H, Wang X-J. Feedforward and feedback frequency-dependent interactions in a large-scale laminar network of the primate cortex. *Science advances* 2016;2: e1601335.
5. Gleeson P, Cantarelli M, Marin B, Quintana A, Earnshaw M, Piasini E, et al. Open Source Brain: a collaborative resource for visualizing, analyzing, simulating and developing standardized models of neurons and circuits. *bioRxiv* 2018; 229484.

## P96 NeuroFedora: a ready to use Free/Open Source platform for Neuroscientists

### Ankur Sinha^1^, Luiz Bazan^2^, Luis M. Segundo^2^, Zbigniew Jędrzejewski-Szmek^2^, Christian J. Kellner^2^, Sergio Pascual^2^, Antonio Trande^2^, Manas Mangaonkar^2^, Tereza Hlaváčková^2^, Morgan Hough^2^, Ilya Gradina^2^, Igor Gnatenko^2^

#### ^1^University of Hertfordshire, Biocomputation Research Group, Hatfield, United Kingdom; ^2^Fedora Project

##### **Correspondence:** Ankur Sinha (a.sinha2@herts.ac.uk)

*BMC Neuroscience* 2019, **20(Suppl 1)**:P96

Modern Neuroscience relies heavily on software. From the gathering of data, simulation of computational models, analysis of large amounts of information, to collaboration and communication tools for community development, software is now a necessary part of the research pipeline.

While the Neuroscience community is gradually moving to the use of Free/Open Source Software (FOSS) [11], our tools are generally complex and not trivial to deploy. In a community that is as multidisciplinary as Neuroscience, a large chunk of researchers hails from fields other than computing. It, therefore, often demands considerable time and effort to install, configure, and maintain research tool sets.

In NeuroFedora, we present a ready to use, FOSS platform for Neuroscientists. We leverage the infrastructure resources of the FOSS Fedora community [3] to develop a ready to install operating system that includes a plethora of Neuroscience software. All software included in NeuroFedora is built in accordance with modern software development best practices, follows the Fedora community’s Quality Assurance process, and is well integrated with other software such as desktop environments, text editors, and other daily use and development tools.

While work continues to make more software available in NeuroFedora covering all aspects of Neuroscience, NeuroFedora already provides commonly used Computational Neuroscience tools such as the NEST simulator [12], GENESIS [2], Auryn [8], Neuron [1], Brian (v1 and 2) [5], Moose [4], Neurord [10], Bionetgen [9], COPASI [6], PyLEMS [7], and others.

With up to date documentation atneuro.fedoraproject.org, we invite researchers to use NeuroFedora in their research and to join the team to help NeuroFedora better aid the research community.

**References**

1. Hines ML, Carnevale NT. The NEURON simulation environment. *Neural computation* 1997 Aug 15;9(6):1179–209.
2. Bower JM, Beeman D, Hucka M. The GENESIS simulation system, 2003.
3. RedHat. *Fedora Project*, 2008.
4. Dudani N, Ray S, George S, Bhalla US. Multiscale modeling and interoperability in MOOSE. *BMC Neuroscience* 2009 Sep;10(1):P54.
5. Goodman DF, Brette R. The Brian simulator. *Frontiers in neuroscience* 2009 Sep 15; 3:26.
6. Mendes P, Hoops S, Sahle S, et al. Computational modeling of biochemical networks using COPASI. *Methods in molecular biology (Clifton, N.J.)* 2009 500, 17–59. ISSN: 1064–3745.
7. Vella M, Cannon RC, Crook S, et al. libNeuroML and PyLEMS: using Python to combine procedural and declarative modeling approaches in computational neuroscience. *Frontiers in neuroinformatics* 2014 Apr 23;8:38.
8. Zenke F, Gerstner W. Limits to high-speed simulations of spiking neural networks using general-purpose computers. *Frontiers in neuroinformatics* 2014 Sep 11;8:76.
9. Harris LA, Hogg JS, Tapia JJ, Sekar JA, Gupta S, Korsunsky I, Arora A, Barua D, Sheehan RP, Faeder JR. BioNetGen 2.2: advances in rule-based modeling. *Bioinformatics* 2016 Jul 8;32(21):3366–8.
10. Jȩdrzejewski-Szmek Z, Blackwell KT. Asynchronous τ-leaping. *The Journal of chemical physics* 2016 Mar 28;144(12):125104.
11. Gleeson P, Davison AP, Silver RA, Ascoli GA. A commitment to open source in neuroscience. *Neuron* 2017 Dec 6;96(5):964–5.
12. Linssen C, Lepperød ME, Mitchell J, et al. *NEST 2.16.0* Aug. 2018-08. 10.5281/zenodo.1400175.

## P97 Flexibility of patterns of avalanches in source-reconstructed magnetoencephalography

### Pierpaolo Sorrentino^1^, Rosaria Rucco^2^, Fabio Baselice^3^, Carmine Granata^4^, Rosita Di Micco^5^, Alesssandro Tessitore^5^, Giuseppe Sorrentino^2^, Leonardo L Gollo^1^

#### ^1^QIMR Berghofer Medical Research Institute, Systems Neuroscience Group, Brisbane, Australia; ^2^University of Naples Parthenope, Department of movement science, Naples, Italy; ^3^University of Naples Parthenope, Department of Engineering, Naples, Italy; ^4^National Research Council of Italy, Institute of Applied Sciences and Intelligent Systems, Pozzuoli, Italy; ^5^University of Campania Luigi Vanvitelli, Department of Neurology, Naples, Italy

##### **Correspondence:** Pierpaolo Sorrentino (ppsorrentino@gmail.com)

*BMC Neuroscience* 2019, **20(Suppl 1)**:P97

**Background:** In many complex systems, when an event occurs, other units follow, giving rise to a cascade. The spreading of activity can be quantified by the branching ratio σ, defined by the number of active units at the present time over the one at the previous time step [1]. If σ = 1, the system is critical. In neuroscience, a critical system is believed to be more efficient [2]. For a critical branching, the system will visit a higher number of states [3]. Utilizing MEG recordings, we characterize patterns of activity at the whole brain level, and we compare the flexibility of patterns observed in healthy controls and Parkinson’s disease (PD). We hypothesize that the damages to the neuronal circuitry will move the network to a less efficient and flexible state, and that this may indicate clinical disability.

**Methods:** We recorded five minutes of closed eyes resting-state MEG in two cohorts: thirty-nine PD patients (20 males and 19 females, age 64.87 ± 9.12 years) matched with thirty-eight controls (19 males and 19 females, age 62.35 ± 8.74 years). The source-level time series of neuronal activity were reconstructed in 116 regions, by a beamformer approach based on the native MRI. The time series were filtered in the classical frequency bands. An avalanche was defined as a continuous sequence of time bins with activity on any region. T σ was estimated based on the geometric mean. We then counted the number of different patterns of avalanches that were present in each subject, and compared them between groups by permutation testing (Fig. 1). Finally, the clinical state was evaluated using the UPDRS-III scale. The relationship occurring between the number of patterns a patient visited and its clinical phenotype was assessed using linear correlation.

**Fig. 1:**
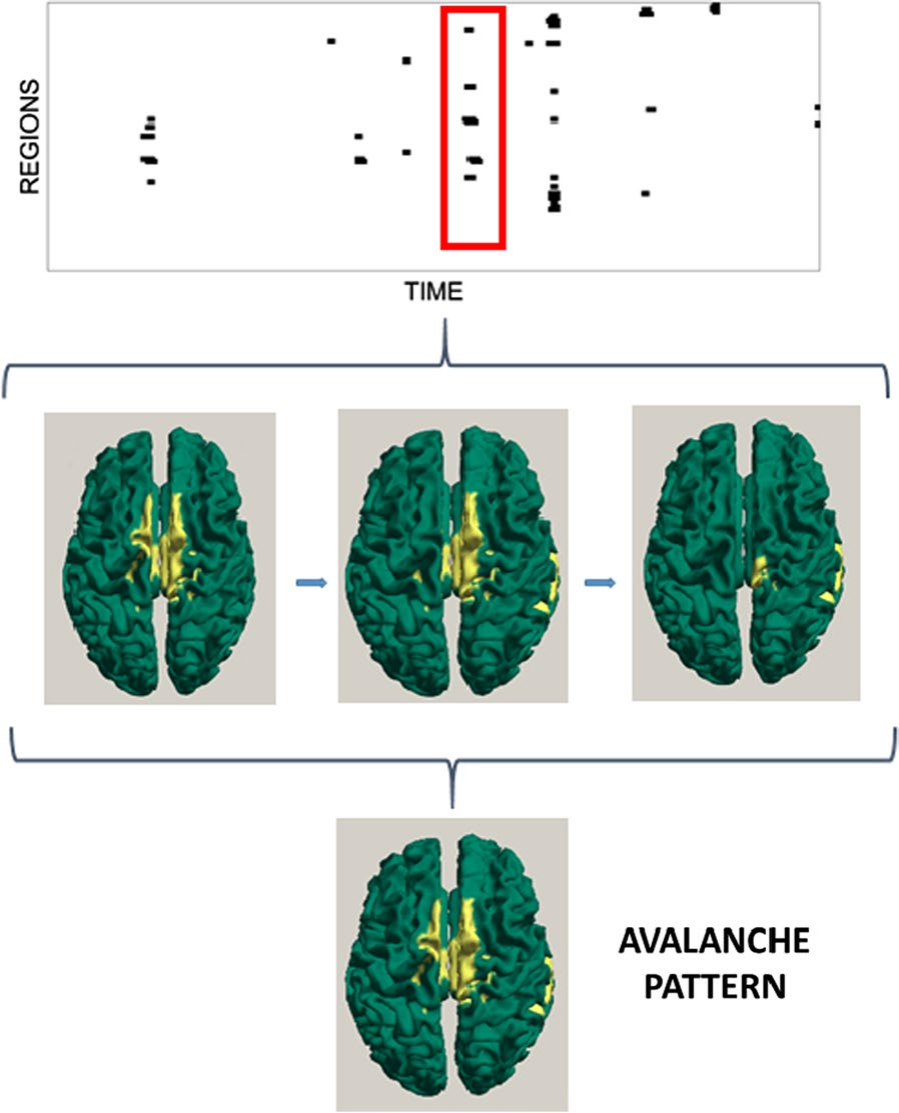
**a** Reconstructed MEG time series. **b** Z-scores of each time series, binarized as abs(z) > 3. **c** Binarized time series, red rectangle is an avalanche. **d** Active regions (yellow) in an avalanche. **e** Avalanche pattern: any area active in any moment during the avalanche. **f** All individual patterns that have occurred (i.e. no pattern repetition is shown in this plot)

**Results:** Firstly, the analysis of sigma shows that MEG signals are in the critical state. Furthermore, the frequency band analysis showed that criticality is not a frequency-specific phenomenon. However, the contribution of each region to the avalanche patterns was frequency specific A comparison between healthy controls and PD patients shows that the latter tend to visit a lower number of patterns (for broad band p = 0.0086). The lower the number of visited patterns, the greater their clinical impairment.

**Discussion:** Here we put forward a novel way to identify brain states and quantify their flexibility. The contribution of regions to the diversity of patterns is heterogeneous and frequency specific, giving rise to frequency specific topologies Although the number of patterns of activity observed across participants is varied, we found that they are substantially reduced in the PD patients. Moreover, the amount of such reduction is significantly associated with the clinical disability.

**References**

1. Kinouchi O, Copelli M. Optimal dynamical range of excitable networks at criticality. *Nature physics* 2006 May;2(5):348.
2. Cocchi L, Gollo LL, Zalesky A, Breakspear M. Criticality in the brain: A synthesis of neurobiology, models and cognition. *Progress in neurobiology* 2017 Nov 1;158:132-52.
3. Haldeman C, Beggs JM. Critical branching captures activity in living neural networks and maximizes the number of metastable states. *Physical review letters* 2005 Feb 7;94(5):058101.

## P98 A learning mechanism in cortical microcircuits for estimating the statistics of the world

### Jordi-Ysard Puigbò Llobet^1^, Xerxes Arsiwalla^1^, Paul Verschure^2^, Miguel Ángel González-Ballester^3^

#### ^1^Institute for Bioengineering of Catalonia, Barcelona, Spain; ^2^Institute for BioEngineering of Catalonia (IBEC), Catalan Institute of Advanced Studies (ICREA), SPECS Lab, Barcelona, Spain; ^3^UPF, ICREA, DTIC, Barcelona, Spain

##### **Correspondence:** Jordi-Ysard Puigbò Llobet (jysard@ibecbarcelona.eu)

*BMC Neuroscience* 2019, **20(Suppl 1)**:P98

We know that the brain can estimate what is the expected value of an input signal. Up to some extent, signals that differ slightly from this expectation will be ignored, whereas errors that exceed some particular threshold will unavoidably convey a behavioral or physiological response. In this work, we assume that this threshold should be variable and therefore dependent on the input uncertainty. Consequently, we present here a biologically plausible model of how the brain can estimate uncertainty in sensory signals. In the predictive coding framework, our model will attempt to assess the validity of sensory predictions and regulate learning accordingly. In this work, we use gradient ascent to derive the formulation that defines a dynamical system which provides estimations of input data while also estimating their variance. We start with the assumption that the probability of our sensory input being explained by internal parameters of the model and other external signals follows a normal distribution. Similar to the approach of [1], we minimize the error in predicting the input signal but, instead of fixing the standard deviation to one static value, we estimate the variance of the input online, as a parameter in our dynamical system. The resulting model is presented as a simple recurrent neural network in Fig. 1C (nodes change with the weighted sum of inputs and vertices follow Hebbian-like learning rules). This microcircuit becomes a model of how cortical networks use expectation maximization to estimate mean and variance of the input signals simultaneously (Fig. 1D). We carefully analyze the implications of estimating uncertainty in parallel to minimizing prediction error to observe that the computation of the variance results in the minimization of the relative error (absolute error divided by variance). While classical models of predictive coding assume the variance to be a fixed constant extracted from the data once, we observe that estimating the variance online increases considerably learning speed at the cost of sometimes converging to less accurate estimations (Fig. 1E). The learning process becomes more resilient to input noise than previous approaches while requiring accurate estimates of the expected input variance. We discuss that this system can be implemented under biological constraints. In that case, our model predicts that two different classes of inhibitory interneurons in the Neocortex must play a role in either estimating mean or variance and that external modulation of the variance-computing interneurons results in the modulation of learning speed, promoting the exploitation of existing models versus the adaptation of existing ones.

**Fig. 1:**
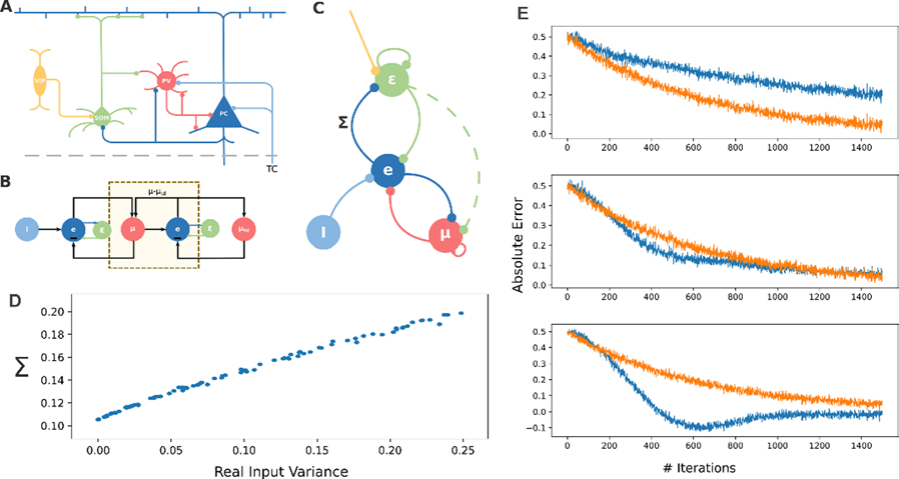
**a** Cortical representation of our microcircuit model, drawn schematically in **c**. **b** extension beyond the Rao-Ballard model of predictive coding by our model. **d** A fairly linear profile in model estimated variance and real variance. **e** Shows the prediction error over time comparing our model (blue) and a standard gradient descent method (orange) for 3 initial estimates of variance

## P99 Generalization of frequency mixing and temporal interference phenomena through Volterra analysis

### Nicolas Perez Nieves, Dan Goodman

#### Imperial College London, Electrical and Electronic Engineering, London, United Kingdom

##### **Correspondence:** Nicolas Perez Nieves (np1714@ic.ac.uk)

*BMC Neuroscience* 2019, **20(Suppl 1)**:P99

It has been recently shown that it is possible to use sinusoidal electric fields at kHz frequencies to enable focused, yet non-invasive, neural stimulation at depth by delivering multiple electric fields to the brain at slightly different frequencies (f1and f2) that are themselves too high to recruit effective neural firing, but for which the offset frequency is low enough to drive neural activity. This is called **temporal interference (TI)** [1]. However, it is not yet known the mechanism by which these electric fields are able to depolarise the cell membrane at the difference frequency despite the lack of depolarization by the individual kHz fields. There is some theoretical analysis into showing how neural stimulation at f1, f2<150Hz generates activity at the difference (f1-f2) and sum (f1+f2) of the frequencies due to the non-linearity of the spiking mechanism in neurons [2] via f**requency mixing (FM)**. Yet, this approach is not general enough to explain why at higher frequencies we still see activity at the difference (f1-f2) with no activity present at any other frequency. To model the non-linearity present in neurons we propose using a Volterra expansion. First, we show that any non-linear system of order P when stimulated by N sinusoids will output a linear combination of sinusoids at frequencies given by all the possible linear combinations of the original frequencies with coefficients ±{0, 1, …, P}. This is consistent with [2] who give output frequencies at fout = nf1+mf2for n, m = ±{0, 1, 2}. We also show that the amplitude of each of the sinusoidal components at the output depends on the P-dimensional Fourier transforms of the Pth order kernel of the Volterra expansion evaluated at the stimulation frequencies (e.g. for a P = 2 system **Ψ**(±f{1, 2}, ±f{1, 2})) We simulate a population of leaky integrate and fire neurons stimulated by two sinusoidal currents at f1and f2and record the average population firing rate. For low frequencies (Fig. 1a), we see all combinations nf1+mf2as in [2]. For high frequencies (Fig. 1b), we only find f1-f2as in [1].

**Fig. 1:**
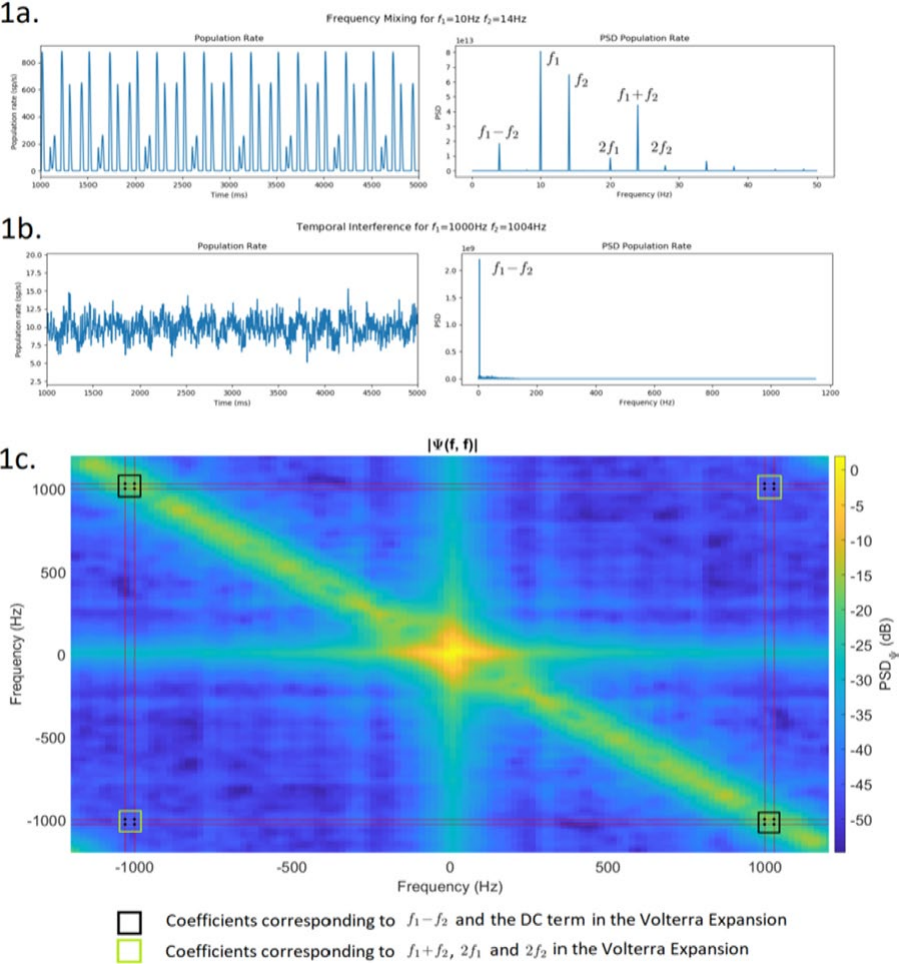
**a**, **b** FM and TI respectively on 1000 LIF neurons. **c** PSD of the 2D-Fourier Transform of the second order Volterra kernel of a non-linear system consisting of the LIF neurons used in **a** and **b** explaining both FM and TI

We then obtain the second order Volterra kernel using the Lee-Schetzen method [3]. The 2D Fourier transform of the kernel is shown in Fig. 1c. The dots show the 16 coefficients corresponding to |**Ψ**(±f{1, 2}, ±f{1, 2})|. As shown, for high stimulation frequencies, only the coefficients corresponding to f1-f2and the DC term are high enough to generate a response in the network, thus explaining TI stimulation. For low stimulation frequencies (<150Hz), all coefficients are high enough to produce a significant response at all nf1+mf2.

We have generalised previous experimental and theoretical results on temporal interference and frequency mixing. Understanding the mechanism of temporal interference stimulation will facilitate its clinical adoption, help develop improvement strategies and may reveal new computational principles of the brain.

**References**

1. Grossman N, et al. Non-invasive Deep Brain Stimulation via Temporally Interfering Electric Fields. *Cell* 2017, Vol.169, Issue 6, pp.1029–1041.e16
2. Haufler D, Pare D. Detection of Multiway Gamma Coordination Reveals How Frequency Mixing Shapes Neural Dynamics. *Neuron* 2019 Vol. 101, Issue 4, pp.603-614.e6
3. van Drongelen, W. Signal Processing for Neuroscientists. *Elsevier* 2010, pp.39-90.

## P100 Neural topic modelling

### Pamela Hathway, Dan Goodman

#### Imperial College London, Department of Electrical and Electronic Engineering, London, United Kingdom

##### **Correspondence:** Pamela Hathway (p.hathway16@imperial.ac.uk)

*BMC Neuroscience* 2019, **20(Suppl 1)**:P100

Recent advances in neuronal recording techniques have led to the availability of large datasets of neuronal activity. This creates new challenges for neural data analysis methods: 1) scalability to larger numbers of neurons, 2) combining data on different temporal and spatial scales e.g. single units and local field potentials and 3) interpretability of the results.

We propose a new approach to these challenges: Neural Topic Modelling, a neural data analysis tool based on Latent Dirichlet Allocation (LDA), a method routinely used in text mining to find latent topics in texts. For Neural Topic Modelling, neural data is converted into the presence or absence of discrete events (e.g. neuron 1 has a higher firing rate than usual), which we call “neural words”. A recording is split into time windows that reflect stimulus presentation (“neural documents”) and the neural words present in each neural document are used as input to LDA. The result is a number of topics—probability distributions over words—which best explain the given occurrences of neural words in the neural documents.

To demonstrate the validity of Neural Topic Modelling we analysed an electrophysiological dataset of visual cortex neurons recorded with a Neuropixel electrode. The spikes were translated into four simple neural word types: 1) increased firing rate in neuron i, 2) decreased firing rate in neuron i, 3) small inter-spike intervals in neuron i, 4) neurons i and j are simultaneously active.

Neural Topic Modelling identifies topics in which the neural words are similar in their preferences for stimulus location and brightness. Five out of ten topics exhibited a clear receptive field (RF)—a small region to which the words in the topic responded preferentially (positive RF, see Fig. 1 D) or non-preferentially (negative RF, see Fig. 1 C) as measured by weighted mean probabilities of the appearance of topic words given the stimulus location. The topic receptive fields overlap with the general mean probability of a word occurring given the stimulus location (see Fig. 1 A), but the topics responded to different subregions (see Fig. 1 B) and some were brightness-sensitive (see Fig. 1 D, right). Additionally, topics seem to reflect proximity on the recording electrode. We confirmed that topic groupings were not driven by word order or overall word count.

**Fig. 1:**
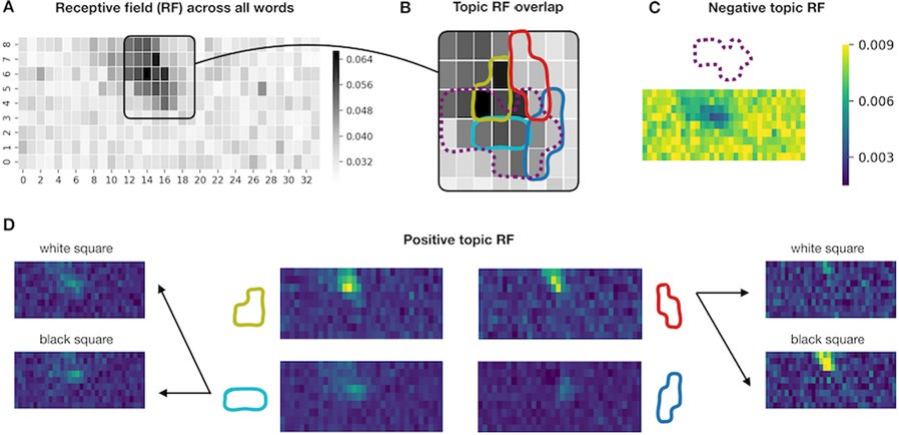
Topic receptive fields. **a** Probability of a word happening given stimulus location on the 9x34 grid. **c**, **d** Weighted mean probabilities for five topics with negative (**c**) and positive (**d**) receptive fields (RF). Colormap applies to C and D. Brightness sensitivity is shown for two topics (**d** left & right). **b** Overlap of pos. and neg. (dashed) RFs from topics in C & D masked at 0.8 of max value

Neural Topic Modelling is an unsupervised analysis tool that receives no knowledge about the cortex topography nor about the spatial structure of the stimuli, but is nonetheless able to recover these relationships. The neural activity patterns used as neural words are interpretable by the brain and the resulting topics are interpretable by researchers. Converting neural activity into relevant events makes the method scalable to very large datasets and enables the analysis of neural data recordings on different spatial or temporal scales. It will be interesting to apply the model to more complex datasets e.g. in behaving mice, or to datasets where the neural representation of the stimulus structure is less clear e.g. for auditory or olfactory experiments.

The combination of scalability, applicability across temporal and spatial scales and the biological interpretability of Neural Topic Modelling sets this approach apart from other machine learning approaches to neural data analysis. We will make Neural Topic Modelling available to all researchers in the form of a Python software package.

## P101 An attentional inhibitory feedback network for multi-label classification

### Yang Chu, Dan Goodman

#### Imperial College London, Electrical Engineering, London, United Kingdom

##### **Correspondence:** Yang Chu (y.chu16@imperial.ac.uk)

*BMC Neuroscience* 2019, **20(Suppl 1)**:P101

It’s not difficult for people to distinguish the sound of a piano and a bass in a jazz ensemble, or to recognize an actor under unique stage lighting, even if these combinations have never been experienced before. However, these multi-label recognition tasks remain challenging for current machine learning and computational neural models. The first challenge is to learn to generalize along with the combinatorial explosion of novel combinations, in contrast to brute-force memorization. The second challenge is to infer the multiple latent causes from mixed signals.

Here we present a new attentional inhibitory feedback model as a first step to address both these challenges and study the impact of feedback connections on learning. The new model outperforms baseline feedforward-only networks in an overlapping-handwritten-digits recognition task. Our simulation results also provide new understanding of feedback guided synaptic plasticity and complementary learning systems theory.

The task is to recognize two overlapping digits in an image (Fig 1A). The advantage of this for comparing neuro-inspired and machine learning approaches is that it is easy for humans but challenging for machine learning models, as they need to learn individual digits from combinations. Recognizing single handwritten digits, by contrast, can easily be solved by modern deep learning models.

**Fig. 1:**
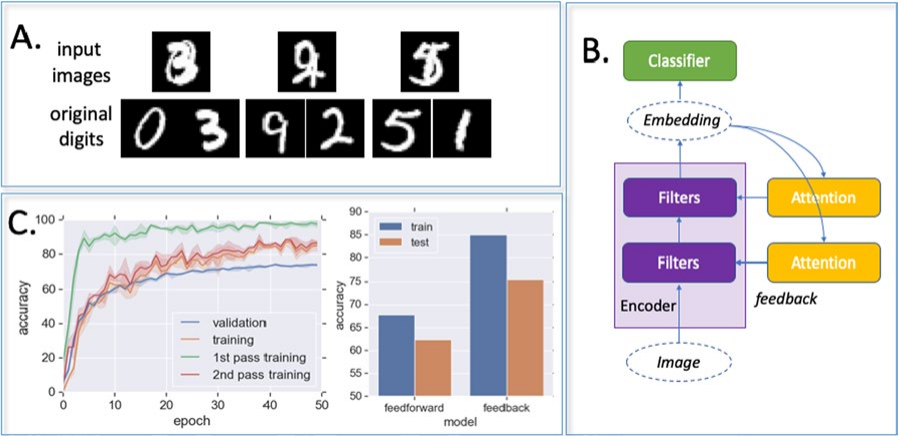
**a** Samples of input images for overlapping handwritten digit recognition task. **b** Attentional feedback network structure. **c** Left: Attentional feedback model learning process. Right: Performance comparison to feedforward-only baseline network

The proposed model (Fig 1B) has a feature encoder built on a multi-layer fully connected neural network. Each encoder neuron receives an inhibitory feedback connection from a corresponding attentional neural network. During recognition, an image is first fed through the encoder, yielding a first guess. Then, based on the most confidently recognized digit, the attention module feeds back a multiplicative inhibitory signal to each encoder neuron. In the following time step, the image is processed again, but by the modulated encoder, resulting in a second recognition result. This feedback loop can carry on several times.

In our model, attention modulates the effective plasticity of different synapses based on the predicted contributions. While the attention networks learn to select more distinctive features, the encoder learns better with synapse-specific guidance from attention. Our feedback model achieves significantly higher accuracy comparing with the feedforward baseline network on both training and validation datasets (Fig 1C), despite having fewer neurons (2.6M compared to 3.7M). State of the art machine learning models can outperform our model but requires five to ten times as many parameters and more than a thousand times training data. Finally, we found intriguing dynamics during the co-learning process among attention and encoder networks, suggesting further links to neural development phenomena and memory consolidation in the brain.

**Acknowledgements:** This work was partly supported by a Titan Xp donated by the NVIDIA Corporation, and The Royal Society (grant RG170298).

## P102 Closed-loop sinusoidal stimulation of ventral hippocampal terminals in prefrontal cortex preferentially entrains circuit activity at distinct frequencies

### Maxym Myroshnychenko^1^, David Kupferschmidt^2^, Joshua Gordon^3^

#### ^1^National Institutes of Health, National Institute of Neurological Disorders and Stroke, Bethesda, MD, United States of America; ^2^National Institute of Neurological Disorders and Stroke, Integrative Neuroscience Section, Bethesda, MD, United States of America; ^3^National Institutes of Health, National Institute of Mental Health, Bethesda, United States of America

##### **Correspondence:** Maxym Myroshnychenko (mmyros@gmail.com)

*BMC Neuroscience* 2019, **20(Suppl 1)**:P102

Closed-loop interrogation of neural circuits allows for causal description of circuit properties. Recent developments in recording and stimulation technology brought about the ability to stimulate or inhibit activity in one brain region conditional on the activity of another. Furthermore, the advent of optogenetics made it possible to control the activity of discrete, anatomically defined neural pathways. Normally, optogenetic excitation is induced using narrow pulses of light of the same intensity. To better approximate endogenous neural oscillations, we used continuously varied sinusoidal open- and closed-loop optogenetic stimulation of ventral hippocampal terminals in prefrontal cortex in awake mice. This allowed us to investigate the dynamical relationship between the two brain regions, which is critical for higher cognitive functions such as spatial working memory. Open-loop stimulation at different frequencies and amplitudes allowed us to map the response of the circuit over a range of parameters, revealing that response power in prefrontal and hippocampal field potentials was maximal in two tongue-shaped regions centered respectively at 8 Hz and 25–35 Hz, resembling resonant properties of coupled oscillators. Coherence between them was also maximal at these two frequency ranges. This suggests that neural activity in the circuit became entrained to the laser-induced oscillation, and the entrainment was not limited to the region near the stimulating laser. Further, adding frequency-filtered feedback based on the hippocampal field potential enhanced or suppressed synchronization depending on the amount of delay introduced to the feedback procedure. Specifically, delaying the optical stimulation relative to the hippocampal signal by about half of its period enhanced the entrainment of the prefrontal and hippocampal field potential responses to the stimulation frequency and enhanced prefrontal spikes’ phase locking to hippocampal field potential. On the other hand, closed-loop feedback without delay resulted in little enhancement and even decreased firing rate of prefrontal neurons. This is to our knowledge the first demonstration of an oscillatory phase-dependent bias in hippocampal-prefrontal communication based on an active closed-loop intervention. These results stand to inform computational models of communication between brain regions, and guide the use of continuously varying, closed-loop stimulation to assess effects of enhancing endogenous long-range neuronal communication on behavioral measures of cognitive function.

## P103 The shape of thought: data-driven synthesis of neuronal morphology and the search for fundamental parameters of form

### Joe Graham

#### SUNY Downstate Medical Center, Department of Physiology and Pharmacology, Brooklyn, NY, United States of America

##### **Correspondence:** Joe Graham (joe.w.graham@gmail.com)

*BMC Neuroscience* 2019, **20(Suppl 1)**:P103

Neuronal morphology is critical in the form and function of nervous systems. Morphological diversity in and between populations of neurons contributes to functional connectivity and robust behavior. Morphologically-realistic computational models are an important tool in improving our understanding of nervous systems. Continual improvements in computing make large-scale, morphologically-realistic, biophysical models of nervous systems increasingly feasible. However, reconstructing large numbers of neurons experimentally is not scalable. Algorithmic generation of neuronal morphologies (“synthesis” of “virtual” neurons) holds promise for deciphering underlying patterns in branching morphology as well as meeting the increasing need in computational neuroscience for large numbers of diverse, realistic neurons.

There are many ways to quantify neuronal form, not all are useful. [1] proposed that “from the mass of quantitative information available” a small set of “fundamental parameters of form” and their intercorrelations could be measured from reconstructed neurons which could potentially “completely describe” the population. A parameter set completely describing the original data would be useful for classification of neuronal types, exploring embryological development of neurons, and for understanding morphological changes following illness or intervention. [2] realized that virtual dendritic trees could be generated by stochastic sampling from a set of fundamental parameters (a synthesis model). Persistent differences between the reconstructed and virtual trees guided model refinement. [3] realized entire virtual neurons could be created by synthesizing multiple dendritic trees from a virtual soma. [3] implemented the models of Hillman and Burke et al. and made the code and data publicly available. Both groups used the same data set: a population of six fully-reconstructed cat alpha motoneurons. They were able to generate virtual motoneurons that were similar to the reconstructed ones, however, persistent, significant differences remained unexplained.

Exploration of these motoneurons and novel synthesis models led to two major insights into dendritic form. 1) Parameter distributions correlate with local properties, and these correlations must be accounted for in synthesis models. Dendritic diameter is an important local property, correlating with most parameters. 2) Parent branch parameters correlate differently than those of terminal branches, requiring setting a branch’s type before synthesizing it. Inclusion of these findings in a synthesis model produces virtual motoneurons that are far more similar to the reconstructions than previous models and which are statistically indistinguishable across most measures. These findings hold true across a variety of neuronal types, and may constitute a key to the elusive “fundamental parameters of form” for neuronal morphology.

**References**

1. Hillman DE. Neuronal shape parameters and substructures as a basis of neuronal form. *In: The Neurosciences, 4th Study program. Cambridge: MIT Press;* 1979, pp. 477–498.
2. Burke RE, Marks WB, Ulfhake B. A parsimonious description of motoneuron dendritic morphology using computer simulation. *Journal of Neuroscience* 1992, 12(6), pp. 2403–2416.
3. Ascoli GA, Krichmar JL, Scorcioni R, Nasuto SJ, Senft SL, Krichmar GL. Computer generation and quantitative morphometric analysis of virtual neurons. *Anatomy and Embryology* 2001 Oct 1;204(4):283–301.

## P104 An information-theoretic framework for examining information flow in the brain

### Praveen Venkatesh, Pulkit Grover

#### Carnegie Mellon University, Electrical and Computer Engineering, Pittsburgh, PA, United States of America

##### **Correspondence:** Praveen Venkatesh (vpraveen@cmu.edu)

*BMC Neuroscience* 2019, **20(Suppl 1)**:P104

We propose a formal, systematic methodology for examining information flow in the brain. Our method is based on constructing a graphical model of the underlying computational circuit, comprising nodes that represent neurons or groups of neurons, which are interconnected to reflect anatomy. Using this model, we provide an information-theoretic definition for information flow, based on conditional mutual information between the stimulus and the transmissions of neurons. Our definition of information flow organically emphasizes what the information is *about*: typically, this information is encapsulated in the stimulus or response of a specific neuroscientific task. We also give pronounced importance to distinguishing the *defining* of information flow from the act of *estimating* it.

The information-theoretic framework we develop provides theoretical guarantees that were hitherto unattainable using statistical tools such as Granger Causality, Directed Information and Transfer Entropy, partly because they lacked a theoretical foundation grounded in neuroscience. Specifically, we are able to guarantee that if the “output” of the computational system shows stimulus-dependence, then there exists an “information path” leading from the input to the output, along which stimulus-dependent information flows. This path may be identified by performing statistical independence tests (or sometimes, conditional independence tests) at every edge. We are also able to obtain a fine-grained understanding of information geared towards understanding computation, by identifying which transmissions contain unique information and which are derived or redundant.

Furthermore, our framework offers consistency-checks, such as statistical tests for detecting hidden nodes. It also allows the experimentalist to examine how information about independent components of the stimulus (e.g., color and shape of a visual stimulus in a visual processing task) flow individually. Finally, we believe that our structured approach suggests a workflow for informed experimental design: especially, for purposing stimuli towards specific objectives, such as identifying whether or not a particular brain region is involved in a given task.

We hope that our theoretical framework will enable neuroscientists to state their assumptions more clearly and hence make more confident interpretations of their experimental results. One caveat, however, is that statistical independence tests (and especially, conditional independence tests) are often hard to perform in practice, and require a sufficiently large number of experimental trials.

## P105 Detection and evaluation of bursts and rate onsets in terms of novelty and surprise

### Junji Ito^1^, Emanuele Lucrezia^1^, Guenther Palm^2^, Sonja Gruen^1,3^

#### ^1^Jülich Research Centre, Institute of Neuroscience and Medicine (INM-6), Jülich, Germany; ^2^University of Ulm, Institute of Neural Information Processing, Ulm, Germany; ^3^Jülich Research Centre, Institute of Neuroscience and Medicine (INM-10), Jülich, Germany

##### **Correspondence:** Junji Ito (j.ito@fz-juelich.de)

*BMC Neuroscience* 2019, **20(Suppl 1)**:P105

The detection of bursts and also of response onsets is often of relevance in understanding neurophysiological data, but the detection of these events is not a trivial task. Building on a method that was originally designed for burst detection using the so-called burst surprise as a measure [1], we extend it to a significance measure, the strict burst surprise [2, 3]. Briefly, the strict burst surprise is based on a measure called (strict) burst novelty, which is defined for each spike in a spike train as the greatest negative logarithm of the p-values for all ISI sequences ending at the spike. The strict burst surprise is defined as the negative logarithm of the p-value of the cumulative distribution function for the strict burst novelty. The burst detection method based on these measures consists of two stages as follows. In the first stage we model the neuron’s inter-spike interval (ISI) distribution and make an i.i.d. assumption to formulate our null hypothesis. In addition, we define a set of ‘surprising’ events that signify deviations from the null hypothesis in the direction of ‘burstiness’. Here the (strict) burst novelty is used to measure the size of this deviation. In the second stage we determine the significance of this deviation. The (strict) burst surprise is used to measure the significance, since it represents (the negative logarithm of) the significance probability of burst novelty values. We first show how a non-proper choice of null hypothesis affects burst detection performance, and then we apply the method to experimental data from macaque motor cortex [4, 5]. For this application the data are divided into a period for parameter estimation to express a proper null-hypothesis (model of the ISI distribution), and the rest of the data is analyzed by using that null hypothesis. We find that assuming a Poisson process for experimental spike data from motor cortex is rarely a proper null hypothesis, because these data tend to fire more regularly and thus a gamma process is more appropriate. We show that our burst detection method can be used for rate change onset detection (see Fig. 1), because a deviation from the null-hypothesis detected by (strict) burst novelty also covers an increase of firing rate.

**Fig. 1:**
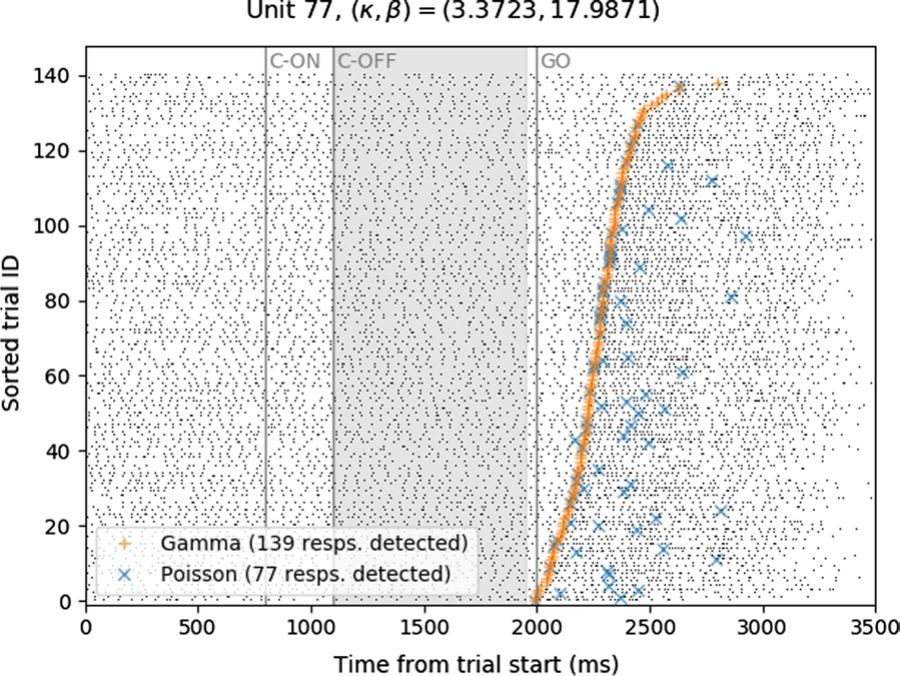
Raster plot of an example single unit (black dots), shown together with rate change onset detection results (orange and blue marks, for gamma and Poisson null hypotheses, respectively). The Poisson null hypothesis fails to detect a lot of rate changes in this case, where the baseline spike train is highly regular (the shape factor k of the spike train is 3.3723, corresponding to a CV of 0.5445)

**References**

1. Legendy CR, Salcman M. Bursts and recurrences of bursts in the spike trains of spontaneously active striate cortex neurons. *Journal of neurophysiology* 1985 Apr 1;53(4):926–39.
2. Palm G. Evidence, information, and surprise. *Biological Cybernetics* 1981 Nov 1;42(1):57–68.
3. Palm G. Novelty, information and surprise. *Springer Science & Business Media*; 2012 Aug 30.
4. Riehle A, Wirtssohn S, Grün S, Brochier T. Mapping the spatio-temporal structure of motor cortical LFP and spiking activities during reach-to-grasp movements. *Frontiers in neural circuits* 2013 Mar 27;7:48.
5. Brochier T, Zehl L, Hao Y, et al. Massively parallel recordings in macaque motor cortex during an instructed delayed reach-to-grasp task. *Scientific data* 2018 Apr 10;5:180055.

## P106 Precise spatio-temporal spike patterns in macaque motor cortex during a reach-to-grasp task

### Alessandra Stella^1^, Pietro Quaglio^1^, Alexa Riehle^2^, Thomas Brochier^2^, Sonja Gruen^1^

#### ^1^Jülich Research Centre, Institute of Neuroscience and Medicine (INM-6 and INM-10), Jülich, Germany; ^2^CNRS - Aix-Marseille Université, Institut de Neurosciences de la Timone (INT), Marseille, France

##### **Correspondence:** Alessandra Stella (a.stella@fz-juelich.de)

*BMC Neuroscience* 2019, **20(Suppl 1)**:P106

The Hebbian hypothesis [1] states that neurons organize in assemblies of co-active neurons acting as information processing units. We hypothesize that assembly activity is expressed by the occurrence of precise spatio-temporal patterns (STPs) of spikes—with temporal delays between the spikes—emitted by neurons being members of the assembly. We developed a method, called SPADE [2, 3], that detects significant STPs in massively parallel spike trains. SPADE involves three steps: it first identifies repeating STPs using Frequent Itemset Mining [4]; second, it evaluates the detected patterns for significance; third, it removes the false positive patterns that are a byproduct of true patterns and background activity. SPADE is implemented in the Python library Elephant [5].

Here we aim to evaluate if cell assemblies are active in relation to motor behavior [6]. Therefore, we analyzed parallel spike data recorded in the pre-/motor cortex of a macaque monkey performing a reach-to-grasp task. The experimental paradigm is the following: after an instructed preparatory period, the monkeys had to pull and hold an object by using either a side or a precision grip, and using either high or low force (four behavioral conditions). We segmented the data into 500ms periods and analyzed them separately for the occurrence of STPs (an extension of [2]). We then registered for each significant STP its neuron composition, its number of occurrences and the times of the spikes involved in the pattern (see an example pattern in Fig. 1). This enabled us to investigate the time-resolved occurrences of each pattern across trials. Furthermore, we can make statistics of patterns’ characteristics in relation to the behavioral condition.

**Fig. 1:**
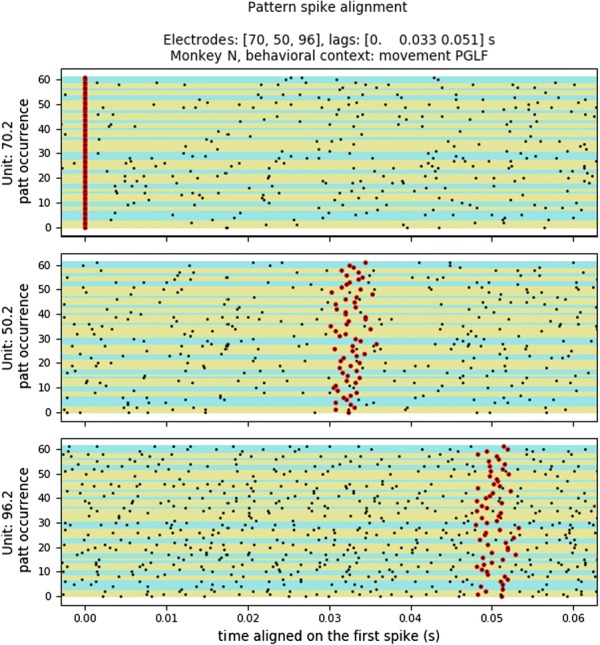
Raster plot of one specific pattern of size 3 (composed of neurons 70.2, 50.2 and 96.2), detected during the trial type PGLF and movement epoch for monkey N. The STP repeated occurrences are aligned to the first spike of the pattern. Spikes belonging to the pattern are marked in red. Different colored bands represent the pattern occurrence within one trial. Trials are ordered along the y-axis

We find that STPs occur in all phases of the behavior, but are more frequent during the movement period. The patterns are specific to the behavioral conditions (different grip and force type combinations) during movement, suggesting that different assemblies are active for the performance of the different behavior. Interestingly, there is a high tendency that the same neurons participate in different STPs, however with different temporal lag constellations, i.e. as different STPs. This means that individual neurons are involved in different patterns at different points in time. These neurons may be interpreted as hub neurons [7]. We also find that individual spikes of some neurons may take part in different patterns. We are currently exploring if that indicates the existence of larger patterns, not detected because of our strict definition on the exact timing and constellation of spikes and neurons in the pattern. This may be too strict given the insights from modeling work [8].

**References**

1. Hebb DO. The organization of behavior; a neuropsychological theory. *A Wiley Book in Clinical Psychology* 1949:62–78.
2. Torre E, Quaglio P, Denker M, Brochier T, Riehle A, Grün S. Synchronous spike patterns in macaque motor cortex during an instructed-delay reach-to-grasp task. *Journal of Neuroscience* 2016 Aug 10;36(32):8329–40.
3. Quaglio P, Yegenoglu A, Torre E, Endres DM, Grün S. Detection and evaluation of spatio-temporal spike patterns in massively parallel spike train data with spade. *Frontiers in computational neuroscience* 2017 May 24;11:41.
4. Picado-Muiño D, Borgelt C, Berger D, Gerstein GL, Grün S. Finding neural assemblies with frequent item set mining. *Frontiers in neuroinformatics* 2013 May 31;7:9.
5. Elephant - Electrophysiology Analysis Toolkit, http://www.python-elephant.org/, RRID:SCR_003833
6. Brochier T, Zehl L, Hao Y, et al. Massively parallel recordings in macaque motor cortex during an instructed delayed reach-to-grasp task. *Scientific data* 2018 Apr 10;5:180055.
7. Dann B, Michaels JA, Schaffelhofer S, Scherberger H. Uniting functional network topology and oscillations in the fronto-parietal single unit network of behaving primates. *Elife* 2016 Aug 15;5:e15719.
8. Diesmann M, Gewaltig MO, Aertsen A. Stable propagation of synchronous spiking in cortical neural networks. *Nature* 1999 Dec;402(6761):529.

## P107 Translating mechanisms of theta rhythm generation from simpler to more detailed network models

### Alexandra Chatzikalymniou^1^, Frances Skinner^2^, Melisa Gumus^3^

#### ^1^Krembil Research Institute and University of Toronto, Department of Physiology, Toronto, Canada; ^2^Krembil Research Institute, Division of Fundamental Neurobiology, Toronto, Canada; ^3^Krembil Research Institute and University of Toronto, Institute of Medical Sciences, Toronto, Canada

##### **Correspondence:** Alexandra Chatzikalymniou (alex4@windowslive.com)

*BMC Neuroscience* 2019, **20(Suppl 1)**:P107

Theta oscillations in the hippocampus are important functional units for phase-coding in the brain [5]. However, how the interactions of the multiple inhibitory cell types and pyramidal cells give rise to these rhythms is far from clear. Recently, Bezaire and colleagues [1] built a full-scale CA1 hippocampus model with 8 inhibitory cell types and pyramidal cells using cellular, synaptic and connectivity characteristics based on a plethora of experimental data. Among other aspects, theirmodel identified interneuronal diversity and parvalbumin positive (PV) cell types as important factors for theta generation. In another recent modeling study [2], a network of PV fast-firing inhibitory and pyramidal cells revealed the importance of post-inhibitory rebound (PIR) as a network property requirement for the emergence of theta. As both models generated theta rhythms intrinsic to the hippocampus [3], we undertook comparisons to both leverage their advantages and overcome their limitations. An analysis of the Bezaire et al network model showed a consistency with the experimental excitatory/inhibitory current balances [4]. Also, the Ferguson et al model [2] predictions of connection probability requirements for theta, were consistent with the empirically determined connections in the Bezaire et al model [3]. Given this, we extracted a network `chunk’ of the later of a size similar to the model in [2], to facilitate comparisons and efficient computational investigations. Since it is known that the CA1 contains multiple theta generators across the septotemporal axis [3], our chunk network representsone of the many oscillators in this area. Without any model parameter adjustments, a chunk of the Bezaire et al no longer produces theta. After taking advantage of the balances exposed in the [2] model using high performance computing, we find that it is possible to generate theta in our chunk model. These rhythms occur preferentially for decreased pyramidal-pyramidal synaptic conductances relative to [1], suggesting that PIR plays a fundamental role in intrinsic theta. Moving forward, our models can be used to extract cell-type specific pathways critical for the theta rhythm.

**References**

1. Bezaire MJ, Raikov I, Burk K, et al. Interneuronal mechanisms of hippocampal theta oscillations in a full-scale model of the rodent CA1 circuit. *ELife Sciences* 2016, 5, e18566.
2. Ferguson KA, Chatzikalymniou AP, Skinner FK. Combining Theory, Model, and Experiment to Explain How Intrinsic Theta Rhythms Are Generated in an In Vitro Whole Hippocampus Preparation without Oscillatory Inputs. *Eneuro*. 2017,4 (4) ENEURO.0131-17.2017
3. Goutagny R, Jackson J, Williams S. Self-generated theta oscillations in the hippocampus. *Nature Neuroscience* 2009, 12, 1491–1493.
4. Huh CYL, Amilhon B, Ferguson KA, et al. Excitatory Inputs Determine Phase-Locking Strength and Spike-Timing of CA1 Stratum Oriens/Alveus Parvalbumin and Somatostatin Interneurons during Intrinsically Generated Hippocampal Theta Rhythm. *Journal of Neuroscience* 2016, 36, 6605–6622.
5. Wilson MA, Varela C, Remondes M. Phase organization of network computations. *Current Opinions Neurobiology* 2015, 31: 250–253.

## P108 NeuroViz: A web platform for visualizing and analyzing neuronal databases

### Elizabeth Haynie^1^, Kidus Debesai^1^, Edgar Juarez Cabrera^1^, Anca Doloc-Mihu^2^, Cengiz Gunay^1^

#### ^1^Georgia Gwinnett College, School of Science and Technology, Lawrenceville, United States of America; ^2^Georgia Gwinnett College, Information Technology/ SST, Decatur, GA, United States of America

##### **Correspondence:** Cengiz Gunay (cengique@users.sf.net)

*BMC Neuroscience* 2019, **20(Suppl 1)**:P108

Computer modeling of neuronal circuits has become a valuable tool in neuroscience. For a neuron model to be useful, its many free parameters need to be properly tuned using various exploration methods. These methods can illustrate a complete picture of all possible model outcomes and have been extremely valuable in understanding the principles of neuronal circuit function. These explorations yield large-scale neuron model simulation results databases, which provide opportunities for further investigations and new discoveries.

Many examples of such databases of simulation results already exist ([1, 2]; for a review, see [3]) and more will be available as new neuronal models are constructed and computing platforms get less expensive and more powerful. Simulation results databases are either publicly unavailable, or only available upon request [4]. However, the data are often stored in custom formats whose size increase exponentially with the number of parameters varied, making collaborations difficult. There is no central repository with a common format to store these databases. As computer simulation technologies and neuron models are advancing, parameter exploration methods are becoming more accessible to many researchers. Therefore, a common location to store and analyze model databases is needed more than ever.

To serve this need, we are proposing an online portal, called NeuroViz, which hosts neuronal simulation and recording databases. NeuroViz is planned to be an open freely available website where researchers can submit new model databases, and visualize and analyze existing databases. At first stage, we plan to provide only tabular data formats that contain parameter values and metrics already extracted from raw electrophysiology data. Here, we are presenting our initial designs of the software interface to validate and explore usage scenarios and receive feedback from potential users. Fig. 1 shows our first step into building this tool. For demonstration purposes, we have incorporated the HCO-DB [1] database from the leech.

**Fig. 1:**
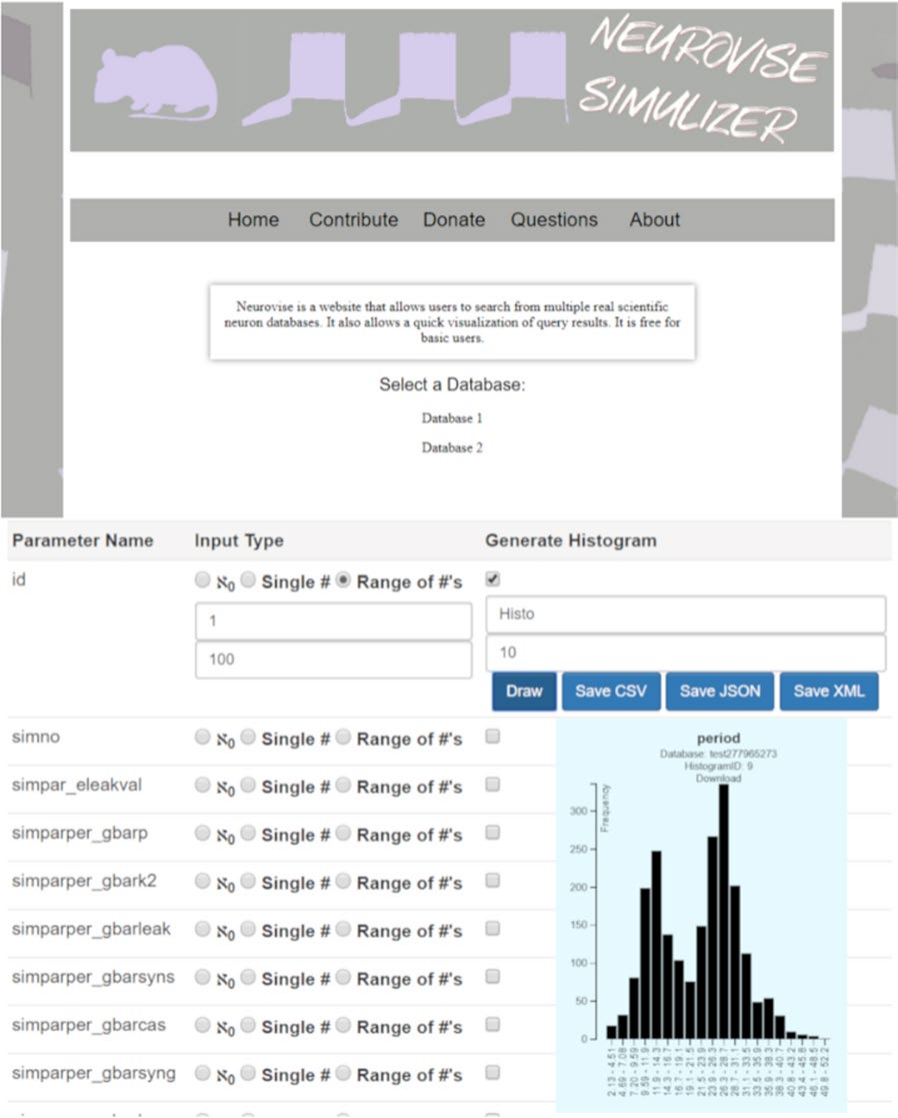
Our proposed web portal NeuroViz is a central repository that can host recorded and model simulation results databases, and provide support and tools to conduct or enhance model parameter exploration research

**References**

1. Doloc-Mihu A, Calabrese RL. A database of computational models of a half-center oscillator for analyzing how neuronal parameters influence network activity. *Journal of biological physics* 2011 Jun 1;37(3):263–83.
2. Sekulić V, Lawrence JJ, Skinner FK. Using multi-compartment ensemble modeling as an investigative tool of spatially distributed biophysical balances: application to hippocampal oriens-lacunosum/moleculare (O-LM) cells. *PLoS One* 2014 Oct 31;9(10):e106567.
3. Günay C. Neuronal model databases. *Encyclopedia of Computational Neuroscience* 2015:2024–8.
4. Günay C, Prinz AA. Model calcium sensors for network homeostasis: sensor and readout parameter analysis from a database of model neuronal networks. *Journal of Neuroscience* 2010 Feb 3;30(5):1686–98.

## P109 Computational analysis of disinhibitory and neuromodulatory mechanisms for induction of hippocampal plasticity

### Ines Guerreiro^1^, Zhenglin Gu^2^, Jerrel Yakel^2^, Boris Gutkin^1^

#### ^1^École Normale Supérieure, Paris, France; ^2^NIEHS, Department of Health and Human Services, Research Triangle Park, United States of America

##### **Correspondence:** Ines Guerreiro (ines.completo@gmail.com)

*BMC Neuroscience* 2019, **20(Suppl 1)**:P109

Since first observed in the rabbit hippocampus [1], LTP has remained a key subject of research, and the hippocampus continues to serve as a model structure for the study of plasticity. Studies of induction of hippocampal plasticity have shown that blockade of GABA inhibition can greatly facilitate the induction of LTP in excitatory synapses [2]. More specifically, recent studies show that repeated inhibition of hippocampal CA1 somatostatin-positive interneurons can induce lasting potentiation of Schaffer collateral (SC) to CA1 EPSCs, suggesting that repeated dendritic disinhibition of CA1 pyramidal cells plays a role in the induction of synaptic plasticity. It was also shown experimentally that repeated cholinergic activation enhanced the SC-evoked EPSCs through a7-containing nicotinic acetylcholine receptors (a7 nAChRs) expressed in oriens lacunosum-moleculare (OLMa2) interneurons. However, it is not clear how these circuits and neuromodulatory factors interplay to result in synaptic plasticity.

To analyse the plasticity mechanisms, first we used a biophysically-realistic computational model to examine mechanistically how inhibitory inputs to hippocampal pyramidal neurons can modulate the plasticity of the SC-CA1 excitatory synapses. We found that locally-reduced GABA release (disinhibition) could lead to increased NMDAR activation and intracellular calcium concentration sufficient to upregulate AMPAR permeability. Repeated disinhibition of the excitatory synapses could lead to larger and longer lasting increase of the AMPAR permeability, i.e. synaptic plasticity. We then used our model to show how repeated cholinergic activation of a7 nAChR in stratum oriens OLMa2 interneurons paired with SC stimulation can induce synaptic plasticity at the SC-CA1 excitatory synapses. Activation of pre-synaptic a7 nAChRs in OLM cells activates these interneurons which, in turn, inhibit fast-spiking stratum radiatum interneurons that provide feed-forward inhibition onto pyramidal neurons after SC excitation, and thus disinhibiting the CA1 pyramidal neurons. Repeated cholinergic activation then leads to repeated feed-forward disinhibition of the pyramidal cell, which can modulate the SC-CA1 synapses by the method previously described.

Our modelling work thus unravels the intricate interplay of the hierarchal inhibitory circuitry and cholinergic neuromodulation as a mechanism for hippocampal plasticity.

**References**

1. Lømo T. Frequency potentiation of excitatory synaptic activity in the dentate area of the hippocampal formation. *Acta Physiol. Scand* 1966, 68(277), 128.
2. Wingstrom H, Gustafsson B. Facilitation of hippocampal long-lasting potentiation by GABA antagonists. *Acta Physiol. Scand* 1985, 125, 159–172

## P110 Coherence states of inter-communicating gamma oscillatory neural circuits

### Gregory Dumont, Boris Gutkin

#### École Normale Supérieure, Paris, France

##### **Correspondence:** Gregory Dumont (gregory.dumont@ens.fr)

*BMC Neuroscience* 2019, **20(Suppl 1)**:P110

Macroscopic oscillations of different brain regions show multiple phase relationships that are persistent across time [3]. Such phase locking is believed to be implicated in a number of cognitive functions and is key to the so-called Communication Through Coherence theory for neural information transfer [3]. Multiple cellular level mechanisms influence the network dynamic and structure the macroscopic firing patterns. Key question is to identify the biophysical neuronal and synaptic properties that permit such motifs to arise and how the different coherence states determine the communication between the neural circuits. We use a semi-analytic approach to investigate the emergence of phase locking within two bidirectionally delayed-coupled spiking circuits with emergent global gamma oscillations. Internally the circuits consist of excitatory and inhibitory quadratic integrate-and-fire neurons coupled synaptically in an all-to-all fashion. Importantly the neurons are heterogeneous and are not all intrinsic oscillators. The circuits can show global pyramidal-interneuron or interneuron gamma rhythms. Using a mean-field approach and an exact reduction method [1, 4], we break down each gamma network into a low dimensional nonlinear system. We then derive the macroscopic phase resetting-curves (mPRCs) [1, 2] that determine how the phase of the global oscillation responds to incoming perturbations. We find that depending on the gamma type and perturbation target (excitatory of inhibitory neurons), the mPRC can be either class I (purely positive) or class II (by-phasic). We then study the emergence of macroscopic coherence states (phase locking) of two weakly synaptically-coupled gamma-networks. We derive a phase equation that links the synaptic mechanisms to the coherence state of the system; notably the determinant part played by the delay and coupling strength in the emergent variety of coherence modes. We show that the delay is a necessary condition for symmetry breaking, i.e. a non-symmetric phase lag between the macroscopic oscillations. We find that a whole host of phase-locking relationships exist, depending on the coupling strength and delay, potentially giving an explanation to the experimentally observations [8]. Our analysis, see Fig. 1, further allows us to understand how signal transfer between the gamma circuits may depend on the nature of their mutual coherence states [2].

**Fig. 1:**
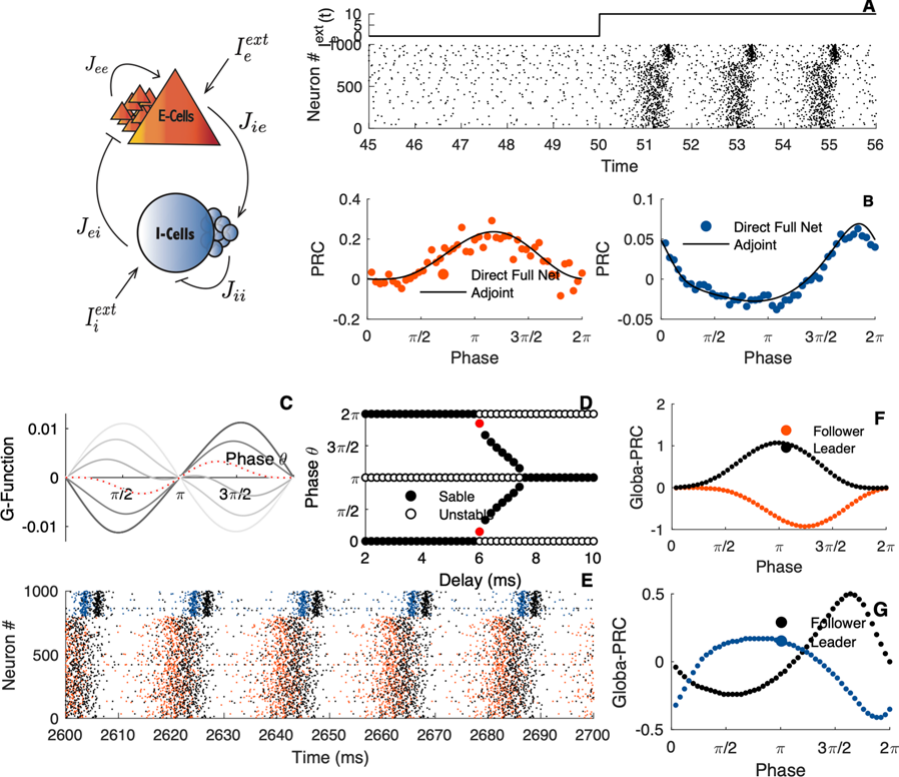
Emergent phase locking and signal flow. **a** Emergent oscillations **b** PRC obtained via direct method (dots) and the adjoint (black line). In red the perturbation are on the E-cells, in blue, on the I-cells. **c** Interaction function for different delays. **d** Diagram locking modes. **e** Spiking activity of the networks is presented. The locking mode corresponds to the prediction. **f**, **g** Global-PRCs

**Acknowledgement:** This study was supported by the Russian Science Foundation grant (contract number: 17-11-01273).

**References**

1. Dumont G, Ermentrout B, Gutkin B. Macroscopic Phase-resetting curves for spiking neural networks. *Phys. Rev. E.* 2017, 96,
2. Dumont G, Gutkin B. Macroscopic Phase-resetting curves determine oscillatory coherence and signal transfer. arXiv:1812.03455[q-bio.NC*]* 2018
3. Maris E, Fries P, van Ede F. Diverse phase relations among neuronal rhythms and their potential function. *Trends in Neurosciences* 2015 39(2), 86–99
4. Montbrio E, Pazo D, Roxin A. Macroscopic description for networks of spiking neurons. *Phys. Rev. X* 2015, 5, 021,028

## P111 Mechanisms of working memory stabilization by an external oscillatory input

### Nikita Novikov^1^, Boris Gutkin^2^

#### ^1^Higher School of Economics, Centre for cognition and decision making, Moscow, Russia; ^2^École Normale Supérieure, Paris, France

##### **Correspondence:** Nikita Novikov (nikknovikov@gmail.com)

*BMC Neuroscience* 2019, **20(Suppl 1)**:P111

Working memory (WM) is the ability to retain information not currently presented from sensory systems. WM retention is accompanied by self-sustained elevation of firing rates, which is usually modelled as transition of a bistable system from the “background” to the “active” state after a brief stimulus presentation [1]. Besides firing rates, the beta oscillations are usually enhanced, supposedly stabilizing WM retention [2]. In this study, we propose mechanisms for such stabilization. Key to these mechanisms is that beta input could provide additional excitation due to non-linear properties of the neurons.

First, we identified the regimes where non-specific beta input affects more strongly populations in the active (memory) state, compared to the background state (due to their different resonant properties). We considered a system of two mutually inhibiting populations, one that (S) actively maintains a stimulus, and the other (D) is selective to a distractor and stays in the background state. Non-selective beta input to both populations provides stronger excitation to S (compared to D), impeding activation of D by the distractor and decreasing the chance that its presentation will erase the stimulus from WM (Fig. 1a, b).

**Fig. 1:**
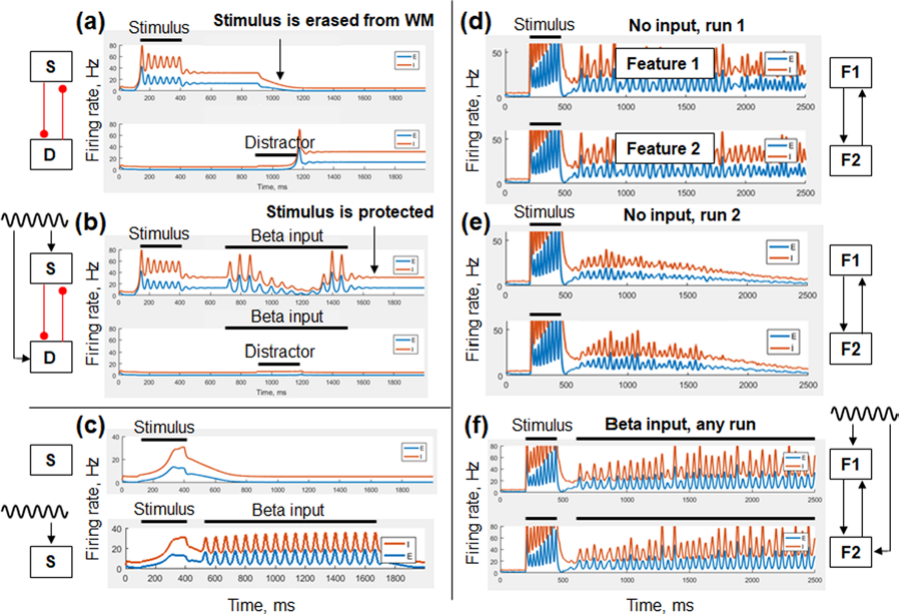
Simulation results. **a**, **b** Two populations with mutual inhibition; **a** no input; **b** beta-band input. **c** Single population with unstable active state; upper panel no input, lower panel beta-band input. **d**–**f** Two populations with mutual excitation; **d**, **e** no input, different noise realizations; **f** beta-band input

Second, we considered models where the WM-holding population does not have a “true” attractor active state, but reacts to the stimulus by a slowly decaying firing rate increase. We found that an external beta input can provide enough excitation to make the memory retention stable (Fig. 1c) (similar mechanism was explored in [3]). Then we considered two such populations with excitatory coupling that could be considered as parts of a distributed single object representation. In the post-stimulus high firing rate regime, populations generate beta-band quasi-oscillations and could synchronize with certain probability, providing mutual excitation that supports stable joint activity (Fig. 1d,e). Weak external beta input to both populations increases the chance for synchronization, thus stabilizing WM retention (Fig. 1f).

We successfully tested the proposed mechanisms with the Wilson-Cowan-like population models. In summary, we demonstrated that WM retention could be stabilized by an external beta-band input via increasing competition between active and background populations, as well as via promoting cooperation between parts of a distributed active population. This is in line with the ideas that beta activity promotes status quo and helps forming of distributed functional ensembles in the cortex.

**Acknowledgements:** Supported by Russian Science Foundation grant (contract No: 17-11-01273).

**References**

1. Amit DJ, Brunel N. Model of global spontaneous activity and local structured activity during delay periods in the cerebral cortex. *Cerebral Cortex* 1997, 7(3):237–252.
2. Engel AK, Fries P. Beta-band oscillations - signaling the status quo? *Current Opinion Neurobiology* 2010, 20(2):156–165
3. Schmidt H, Avitabile D, Montbrio E, Roxin A. Network mechanisms underlying the role of oscillations in cognitive tasks. *PLoS Computational Biology* 2018, 14(9):e1006430.

## P112 Prediction of mean firing rate shift induced by externally applied oscillations in a spiking network model

### Kristina Vodorezova^1^, Nikita Novikov^1^, Boris Gutkin^2^

#### ^1^Higher School of Economics, Center for Cognition and Decision Making, Moscow, Russia; ^2^École Normale Supérieure, Paris, France

##### **Correspondence:** Kristina Vodorezova (trustyourt@gmail.com)

*BMC Neuroscience* 2019, **20(Suppl 1)**:P112

One presumable role of neural oscillations is stabilization or destabilization of neural codes, which promotes retention or updating of the encoded information, respectively [1, 2]. We hypothesize that such functions could stem from the ability of oscillations to differentially affect neural populations that actively retain information and those that stay in the background state [3]. To explore this mechanism, we considered a bistable excitatory-inhibitory network of leaky-integrate-and-fire (LIF) neurons with external sinusoidal forcing. The two steady states differ by their average firing rates and correspond to the active retention and to the background, respectively. We wanted to understand how periodic beta-band input affects time-averaged firing rates in both states. In order to systematically address this question, we developed a method for semi-numerical prediction of the oscillation-induced average firing rate shifts. We simulated single LIF neurons under various combinations of the input mean, variance, and oscillation amplitude. This time-consuming step was performed once; then its results were interpolated during the parameter space exploration. We considered a discrete grid in re-ri coordinates (time-averaged presynaptic excitatory and inhibitory firing rates, respectively). For each (re, ri) combination, we calculated the corresponding input mean and variance, as well as the linear response coefficient (input-output amplitude relation) for each population. Then, in a reverse-engineering way, we derived the amplitudes of the total (external + recurrent) oscillatory inputs. The pre-calculated data was used to determine the time-averaged postsynaptic firing rates (re1, ri1). The curves re = re1 and ri = ri1 defined the nullclines for the time-averaged forced system, and their intersections—the corresponding equilibria. In order to predict the stimulation effect, we visualized the nullclines both for the models with and without external periodic input (Fig. 1a). Using the described method, we found parameters, for which the oscillatory input produced an increase in the average firing rate of the excitatory population in the active memory state, but not in the background state. Our predictions were confirmed by spiking network simulations (Fig. 1b,c). Given the obtained results, we suggest that our method would be useful for further investigation of oscillatory control in multi-stable systems such as working memory or decision-making models.

**Fig. 1:**
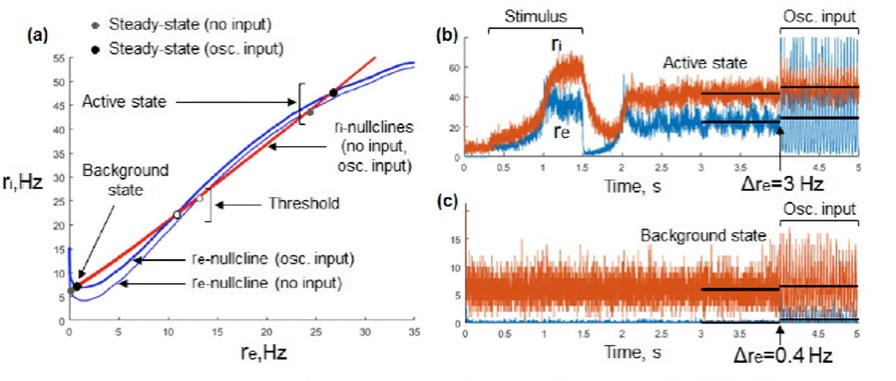
**a** Phase plane for the unforced and for the periodically forced time-averaged system. **b**, **c** Results of the spiking network simulation. In **b** stimulus switches the network into the active state, in **c** the network stays in the background state. Horizontal black lines denote average firing rates with and without the input oscillations

**Acknowledgements:** Supported by a Russian Science Foundation grant (contract No: 17-11-01273).

**References**

1. Engel AK, Fries P. Beta-band oscillations–signalling the status quo? *Current opinion in neurobiology* 2010;20(2):156–65.
2. Brittain JS, Sharott A, Brown P. The highs and lows of beta activity in cortico-basal ganglia loops. *The European journal of neuroscience* 2014;39(11):1951–9.
3. Schmidt H, Avitabile D, Montbrio E, Roxin A. Network mechanisms underlying the role of oscillations in cognitive tasks. *PLoS computational biology* 2018, 14(9):e1006430.

## P113 Augmenting the source-level EEG signal using structural connectivity

### Katharina Glomb^1^, Joana Cabral^2^, Margherita Carboni^3,4^, Maria Rubega^4^, Sebastien Tourbier^1^, Serge Vulliemoz^3,4^, Emeline Mullier^1^, Morten L Kringelbach^5^, Giannarita Iannotti^4^, Martin Seeber^4^, Patric Hagmann^1^

#### ^1^Centre Hospitalier Universitaire Vaudois, Department of Radiology, Lausanne, Switzerland; ^2^University of Minho, Life and Health Sciences Research Institute, Braga, Portugal; ^3^University Hospital of Geneva, Geneva, Switzerland; ^4^University of Geneva, Department of Fundamental Neurosciences, Geneva, Switzerland; ^5^University of Oxford, Department of Psychiatry, Oxford, United Kingdom

##### **Correspondence:** Katharina Glomb (katharina.glomb@upf.edu)

*BMC Neuroscience* 2019, **20(Suppl 1)**:P113

Due to its high temporal resolution, EEG, in principle, can be used to characterize the dynamics of how remote brain regions communicate with each other on a millisecond scale. Recent advances have also made it possible to project the time series recorded at the scalp into the gray matter, localizing the sources of the activity. The main limitations one faces when analyzing such source-level time series are that the source signal has low SNR and spatial resolution and is polluted by volume conduction of the electromagnetic field between the sources. The latter leads to signals appearing functionally connected (i.e., statistically dependent) simply due to their proximity. Here we propose a new approach that addresses these issues: We combine source-level EEG data (resting state, 18 subjects) with structural connectivity (SC; number of streamlines found via diffusion imaging and fiber tracking). Thereby we exploit the fact that functional connectivity (FC) is partially mediated by anatomical white matter connections. We define a graph which consists of N nodes corresponding to N brain regions. Edges between nodes are defined by the SC. The source-projected activity measured at each point in time is taken to be the activity of the graph’s nodes over time. Each node in this graph has a set of nearest neighbors (NNs), i.e. nodes to which it is directly connected according to the SC, and we smooth our data using these NNs. In particular, for each point in time, a weighted average of each node’s NNs’ activity is added to its own activity. The contribution of the NNs is scaled by a factor G, controlling the level of smoothing. This procedure corresponds to convolving the electric signal with a low-pass filter, in graph space.

To test whether this method reduces the effects of volume conduction, we correlate EEG-FC to FC derived from fMRI, a method which does not suffer from volume conduction (fig. left). We compute envelope correlation-based EEG-FC matrices in three different frequency bands (alpha, beta, gamma; See Fig. 1, middle). We find that the EEG-FCs derived from smoothed data (fig. right) have a better fit to the fMRI-FC (0.28 vs 0.46 at G = 200). Importantly, the increase in fit is significantly stronger than when using NNs purely derived from Euclidean distance (Wilcoxon signed-rank test, corrected alpha = 0.05).

**Fig. 1:**
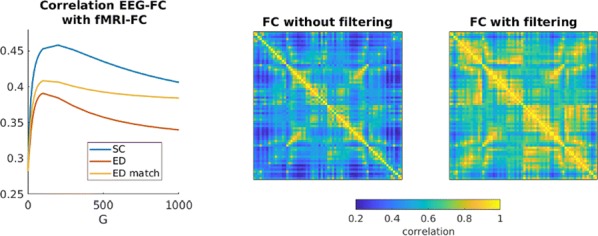
Left: Fits of EEG-FC (beta) to fMRI-FC depending on the strength G of SC-based smoothing, when using nearest neighbors defined: blue: the SC, red: the Euclidean distance, yellow: the Euclidean distance masked by the SC (same pairs of brain regions as in the SC are connected, but with weights derived from Euclidean distance). Middle: EEG-FC (beta) at G = 0. Right: EEG-FC at G = 0

To further validate our technique, we fit the EEG-FCs to simulated data which are free of volume conduction effects. We use a network of N Kuramoto oscillators, coupled according to the empirical SC scaled by a global factor K. This model includes time delays tau, which are proportional to the length of the fibers connecting brain regions. We compute FCs from simulated data in the same way as from empirical data and assess the correlations between simulated and empirical FCs across a parameter space spanned by K and tau. Without filtering the empirical data, the maximum fit is 0.38; with filtering, this value increases to 0.49, again at G = 200. This is in line with the interpretation that graph filtering removes spurious correlations between nearby regions and boosts FC between far-away pairs of regions, demonstrating the merit of combining structural (SC) and functional data in EEG.

## P114 Inferring birdsong neural learning mechanisms from behavior

### Hazem Toutounji^1^, Anja Zai^1^, Ofer Tchernichovski^2^, Dina Lipkind^3^, Richard Hahnloser^1^

#### ^1^Institute of NeuroInformatics, UZH/ETH, Zurich, Switzerland; ^2^Hunter College, New York, United States of America; ^3^City University of New York, York College, New York, United States of America

##### **Correspondence:** Hazem Toutounji (hazem@ini.ethz.ch)

*BMC Neuroscience* 2019, **20(Suppl 1)**:P114

Learning complex skills requires continuous coordination between several behaviors. Juvenile male zebra finches, for instance, learn to produce songs by imitating the songs of adult male tutors. These songs consist of a sequence of syllables with distinct spectral features (e.g., pitch). The juvenile’s task is twofold: matching their syllable repertoire to the tutor’s (syllable assignment), and producing syllables in the right temporal order (syntax learning). It was previously shown that in learning a new song that involves both pitch and syntax change, juveniles first assign syllables to targets, followed by syntax learning [1]. Our work aims at identifying potential neural mechanisms of syllable assignment through data-driven computational modelling within reinforcement learning (RL) theory.

RL theory states that skill acquisition proceeds by learning the behaviors that maximize future rewards. This theoretical framework is often invoked as a working hypothesis for explaining songbird behavior when an aversive auditory stimulus is presented to an adult as reinforcement, but no formal models are yet developed in this context. Furthermore, dopaminergic neurons in the Ventral Tegmental Area (VTA) of adult songbird brains provide the learning signal necessary for escaping the aversive stimulus [2]. Juveniles, however, learn syllable assignment in the absence of external reinforcement, leading us to posit that an inner critic system drives learning during development. Sincedepleting dopamine in juveniles impairs tutor song learning [3], we suggest that the critic evaluates how well syllables in the juvenile’s repertoire match those in the target song.

Here we show that syllable assignment learning as observed experimentally [1] can be reproduced by assuming an intrinsic, global reward function that evaluates similarity in pitch between sung and target syllables. We develop an RL model in which an independent agent represents the motor program for each syllable pitch, assuming that both action and reward are continuous functions. Each agent aims at maximizing the global reward by adjusting its mean syllable pitch toward a target. We infer model parameters from one set of experimental data, including pitch and reward variances, and the time when a juvenile switches its attention toward a new target. Simulations with data-inferred parameters illustrate accurate qualitative agreement between data not involved in the fitting procedure and model. Finally, we make model-based, quantitative predictions on changes in dopaminergic VTA neuronal activity during juvenile song learning. These predictions are empirically verifiable and will be the basis for future investigations in the songbird brain.

**Acknowledgments:** Hazem Toutounji acknowledges the financial support of the German Academic Exchange Service (DAAD).

**References**

1. Lipkind D, Zai, AT, Hanuschkin, A, et al. Songbirds work around computational complexity by learning song vocabulary independently of sequence. *Nature Communications* 2017, 8(1247).
2. Gadagkar V, Puzerey PA, Chen R, et al. Dopamine neurons encode performance error in singing birds. *Science* 2016, 354(6317), 1278–1282.
3. Hisey E, Kearney MG, Mooney, R. A common neural circuit mechanism for internally guided and externally reinforced forms of motor learning. *Nature Neuroscience* 2018 21(4), 589–597.

## P115 A two-compartment neuron model with ion conservation and ion pumps

### Marte J. Sætra^1^, Gaute Einevoll^2^, Geir Halnes^2^

#### ^1^University of Oslo, Department of Physics, Oslo, Norway; ^2^Norwegian University of Life Sciences, Faculty of Science and Technology, Aas, Norway

##### **Correspondence:** Marte J. Sætra (m.j.satra@fys.uio.no)

*BMC Neuroscience* 2019, **20(Suppl 1)**:P115

In most computational models of neurons, the membrane potential is the key dynamic variable. A common model assumption is that the intra- and extracellular concentrations of the main charge carriers (K+, Na+, Cl-) are effectively constant during the simulated period. On the time scale relevant for synaptic integration and the firing of a few action potentials (<1s), this is often a good approximation, since the transmembrane ion exchange is too small to impose significant concentration changes on this short timescale. The approximation is often valid also on a longer time scale due to the work done by uptake mechanisms (ion pumps and co-transporters) to restore baseline concentrations. However, in cases of neuronal hyperactivity or pump dysfunction, the re-uptake may become too slow, and ion concentrations may change over time. This occurs in several pathological conditions, including epilepsy, stroke and spreading depression.

To explore conditions involving shifts in ion concentrations, one needs neuron models that fully keep track of all ions and charges in the intra- and extracellular space. To accommodate this, we propose a version of the two-compartment (soma + dendrites) Pinsky-Rinzel model of a CA3 pyramidal cell [1], which is expanded so that it (i) includes two additional compartments for the extracellular space outside the soma and dendrite compartment, (ii) keeps track of all ion concentrations (K+, Na+, Cl- and Ca2+) in the intra- and extracellular compartments, and (iii) adds additional membrane mechanisms for ion pumps and co-transporters. The additional membrane mechanisms were taken from a previous model [2], and ion transports in the intra- and extracellular space were modelled using an electrodiffusive formalism that ensures ion conservation and a consistent relationship between ion concentrations and membrane voltages [3].

We tuned the new model aiming to preserve the characteristic firing properties of the original model, and at the same time obtain realistic ion-concentration dynamics, i.e., concentrations that remained close to physiological baseline values during normal working conditions, but diverged from baseline during neural hyperactivity. We analyzed the model by performing a sensitivity analysis using Uncertainpy [4]. With its reduced morphology, we envision that the model will be a useful building block in large network simulations of pathological conditions associated with ion concentration shifts in the extracellular space, such as stroke, spreading depression, and epilepsy.

**References**

1. Pinsky PF, Rinzel J. Intrinsic and network rhythmogenesis in a reduced Traub model for CA3 neurons. *Journal of Computational Neuroscience* 1994, 1(1–2):39–60.
2. Wei Y, Ullah G, Schiff SJ. Unification of neuronal spikes, seizures, and spreading depression. *Journal of Neuroscience* 2014, 34(35):11733–11743.
3. Halnes G, Østby I, Pettersen KH, Omholt SW, Einevoll GT. Electrodiffusive model for astrocytic and neuronal ion concentration dynamics. *PLoS Computational Biology* 2013, 9(12).
4. Tennøe S, Halnes G, Einevoll GT. Uncertainpy: A Python toolbox for uncertainty quantification and sensitivity analysis in computational neuroscience. *Frontiers in Neuroinformatics* 2018, 12.

## P116 Endogenously oscillating motoneurons produce undulatory output in a connectome-based neuromechanical model of C. elegans without proprioception

### Haroon Anwar^1,2^, Lan Deng^2^, Soheil Saghafi^3^, Jack Denham^4^, Thomas Ranner^4^, Netta Cohen^4^, Casey Diekman^3^, Gal Haspel^2^

#### ^1^Princeton Neuroscience Institute, Princeton, NJ, United States of America; ^2^Federated Department of Biological Sciences, New Jersey Institute of Technology and Rutgers University - Newark; ^3^Department of Mathematical Sciences, New Jersey Institute of Technology, Newark; ^4^University of Leeds, School of Computing, Leeds, United Kingdom; ^5^New Jersey Institute of Technology, Mathematics Sciences, Newark, United States of America

##### **Correspondence:** Haroon Anwar (hanwar@njit.edu)

*BMC Neuroscience* 2019, **20(Suppl 1)**:P116

Neural circuits producing rhythmic behavior are often driven by pacemaker neurons. The endogenous pacemaker activity is often modulated by proprioceptive or descending signal. Although all the components of the compact locomotion circuit of *Caenorhabditis elegans* are identified and their connectivity has been deduced from electron micrographs, the neural mechanisms underlying rhythm generation and undulatory locomotion are still unknown. In *C. elegans*, undulation is produced by a propagation of alternating activation of 95 dorsal and ventral muscle cells along the animal body, opposite to the direction of movement. Past studies have mainly focused on two hypotheses: 1) Sensory feedback suffices to generate and propagate the rhythm: There are no pacemaker neurons and the neural circuit merely integrates over proprioceptive inputs to generate and propagate appropriate muscle activity [1, 2]. 2) Head oscillator model: A dorsoventral alternating pattern is generated in the neck by an oscillator, which drives the sensory feedback propagation along the animal [6-9]. Gjorgjieva et al [4] revisit a third hypothesis: Dorsoventral alternations are produced locally by oscillating pacemaker neurons and the orchestrations of appropriate phase relations are mediated by the finely tuned neuronal circuitry. In this study, we chose a computational approach to test the conditions for generation of locomotion patterns relying on pacemakers in the known connectivity in the absence of proprioceptive feedback.

We use our previously described neuromuscular network [5] that spans the full length of an animal and includes seven classes of motoneurons, muscle cells, and synaptic connections, both chemical and electrical. Using two kinds of motoneuron classes and muscle cells: leaky (passive) and endogenously oscillating (pacemaker), we systematically screened all 2^7 = 128 configurations of passive and pacemaker motoneuron classes. For each configuration, we screened parameter space and used parameter optimization approach to search for synaptic weights that produce a propagating dorsoventral alternation of muscular activity in forward or backward directions. The opposing directions of locomotion were induced by adding a tonic current to forward or backward motoneurons. We scored the dorsoventral alternation phases to evaluate simulation outputs, and used the same scoring algorithm on biological animals to assess biologically realistic undulation patterns. In the second stage, to see how fictive patterns translate in an embodied scenario, successful neuromuscular outputs were fed into a neuromechanical model [3] to test for realistic forward and backward locomotion.

When motoneuron classes were either all passive or all endogenous oscillators, an undulatory pattern in both forward and backward directions was not generated. We found that several configurations in which some excitatory motoneurons were oscillators produced undulatory-like activity pattern in both forward and backward directions. Moreover, implementation of these motor programs in the neuromechanical model produced multiple trajectories with varying speed and waveform, and clear wave propagation during both forward and backward locomotion depending on descending drive.

**References**

1. Boyle JH, Berri S, Cohen N. Gait Modulation in C. elegans: An Integrated Neuromechanical Model. *Frontiers in Computational Neuroscience* 2012;6: 10. 10.3389/fncom.2012.00010
2. Cohen N, Sanders T. Nematode locomotion: dissecting the neuronal-environmental loop. *Current* *Opinions Neurobiology* 2014; 25: 99–106. 10.1016/jconb.2013.12.003
3. Denham JE, Ranner T, Cohen N. Signatures of proprioceptive control in Caenorhabditis elegans locomotion. *Phil Trans Royal Society B.* 2018; 10.1098/rstb.2018.0208
4. Gjorgjieva J, Biron D, Haspel G. Neurobiology of Caenorhabditis elegans Locomotion: Where Do We Stand? *BioScience* 2014;64: 476. 10.1093/biosci/biu058
5. Haspel G, O’Donovan MJ. A Perimotor Framework Reveals Functional Segmentation in the Motoneuronal Network Controlling Locomotion in Caenorhabditis elegans. *Journal of Neuroscience* 2011;31: 14611–14623. 10.1523/jneurosci.2186-11.2011
6. Karbowski J, Schindelman G, Cronin CJ, Seah A, Sternberg PW. Systems level circuit model of C. elegans undulatory locomotion: mathematical modelling and molecular genetics. *Journal of Computational Neuroscience* 2008;24: 253–276. 10.1007/s10827-007-0054-6
7. Kunert JM, Proctor JL, Brunton SL, Kutz JN. Spatiotemporal feedback and network structure drive and encode Caenorhabditis elegans locomotion. *PLoS Computational Biology* 2017; e1005303. 10.1371/journal.pcbi.1005303
8. Niebur E, Erdös P. Theory of the locomotion of nematodes: control of the somatic motor neurons by interneurons. *Mathematical Biosciences* 1993;118: 51–82.
9. Wen Q, Po MD, Hulme E, Chen S, Liu X, Kwok SW, et al. Proprioceptive Coupling within Motor Neurons Drives C. elegans Forward Locomotion. *Neuron* 2012;76: 750–761. 10.1016/j.neuron.2012.08.039

## P117 Optimized reservoir computing with stochastic recurrent networks

### Sandra Nestler, Chriastian Keup, David Dahmen, Moritz Helias

#### Jülich Research Centre, Institute of Neuroscience and Medicine (INM-6), Jülich, Germany

##### **Correspondence:** Sandra Nestler (s.nestler@fz-juelich.de)

*BMC Neuroscience* 2019, **20(Suppl 1)**:P117

Cortical networks are strongly recurrent, and neurons have intrinsic temporal dynamics. This sets them apart from deep networks. Reservoir computing [1, 2] is an approach that takes these features into account. Inputs are here mapped into a high dimensional space spanned by a large number of typically randomly connected neurons; the network acts like a kernel in a support vector machine (Fig. 1). Functional tasks on the time-dependent inputs are realized by training a linear readout of the network activity.

**Fig. 1:**
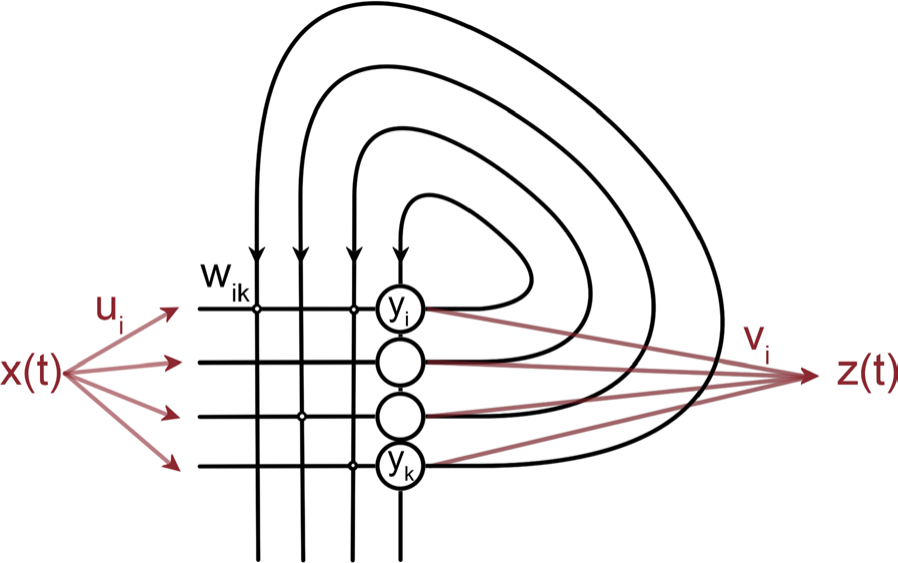
Reservoir Computing Scheme. A neural network with random connectivity (middle) is stimulated with an input via an input vector (left). A linear readout transforms the high dimensional signal into a one-dimensional quantity (right). While the performance dependence on the properties of the connectivity is well studied, we aim at quantifying the effects of input modulation and readout generation

It has been extensively studied how the performance of the reservoir depends on the properties of the recurrent connectivity; the edge of chaos has been found as a global indicator of good computational properties [3, 4].

However, the interplay of recurrence, nonlinearities, and stochastic neuronal dynamics may offer optimal settings that are not described by such global parameters alone. We here set out to systematically analyze the kernel properties of recurrent time-continuous stochastic networks in a binary time series classification task. We derive a learning rule that maximizes the classification margin. The interplay between the signal and neuronal noise determines a single optimal readout direction. Finding this direction does not require a training process; it can be directly calculated from the network statistics. This technique is reliable and yields a measure of linear separability that we use to optimize the remainder of the network. We show that the classification performance crucially depends on the input projection; random projections will lead to significantly suboptimal readouts.

We generalize these results to nonlinear networks. With field theoretical methods [5] we derive systematic corrections due to neuronal nonlinearities, which decompose the recurrent network into an effective bilinear time-dependent kernel. The expressions expose how the network dynamics separates a priori linearly non-separable time-series, and thus explain how recurrent nonlinear networks acquire capabilities beyond a linear perceptron.

**Acknowledgements:** Partly supported by HGF young investigator’s group VH-NG-1028 and European Union Horizon 2020 grant 785907 (Human Brain Project SGA2).

**References**

1. Maass W, Natschlaeger T, Markram H. Real-time computing without stable states: A new framework for neural computation based on perturbations. *Neural Computation* 2002, 2531–2560.
2. Jaeger H, Haas H. Harnessing nonlinearity: Predicting chaotic systems and saving energy in wireless communication. *Science* 2004, 304, 78–80
3. Bertschinger N, Natschlaeger T, Legenstein R. At the Edge of Chaos: Real-time Computations and Self-Organized Criticality in Recurrent Neural Networks. *Conference: Advances in Neural Information Processing System*s 17, 2005. (pp. 145–152).
4. Toyoizumi T, Abbott L. Beyond the edge of chaos: Amplification and temporal integration by recurrent networks in the chaotic regime. *Phys. Rev. E* 2004, 84, 051908.
5. Helias M, Dahmen D. Statistical field theory for neural networks. 2019, arXiv:1901.10416.

## P118 Coordination between individual neurons across mesoscopic distances

### David Dahmen^1^, Moritz Layer^1^, Lukas Deutz^2^, Paulina Dabrowska^1,3^, Nicole Voges^1,3^, Michael von Papen^1,3^, Sonja Gruen^1,4^, Markus Diesmann^1,3^, Moritz Helias^1^

#### ^1^Jülich Research Centre, Institute of Neuroscience and Medicine (INM-6), Jülich, Germany; ^2^University of Leeds, School of Computing, Leeds, Germany; ^3^Jülich Research Centre, Institute for Advanced Simulation (IAS-6), Jülich, Germany; ^4^Jülich Research Centre, Institute of Neuroscience and Medicine (INM-10), Jülich, Germany

##### **Correspondence:** David Dahmen (d.dahmen@fz-juelich.de)

*BMC Neuroscience* 2019, **20(Suppl 1)**:P118

The cortex is a network of networks that is organized on various spatial scales [1, 2]. On the largest scale, coordination of activity is mediated by specific white matter connectivity patterns of small-world character, allowing for short path lengths between any two cortical areas. In contrast, on the scale of small groups of neurons (<100 microns), connection patterns are seemingly random, offering the potential communication between any two cells. For the intermediate, mesoscopic scale inside a cortical area one finds that the majority of connections is governed by connection probabilities that fall off with distance on characteristic length scales of a few hundred microns. Neurons that are a few millimeters apart therefore most likely lack any synapse that would be required for coordination.

Yet, in massively parallel recordings of motor cortex spiking activity in awake and resting macaque monkey we find strongly correlated neurons almost across the whole Utah array, which covers an area of 4 × 4 mm^2^. Positive and negative correlations form salt-and-pepper patterns in space that are seemingly unrelated to the underlying short-range connectivity profiles. Whilst additional complex connection and input structures could potentially give rise to such patterns, we here show that the latter emerge naturally in a dynamically balanced network near criticality [3] where interactions are mediated by a multitude of parallel paths through the network. As a consequence of multi-synaptic interactions via excitatory and inhibitory neurons, spatial profiles of correlations are much wider than those expected from structured connectivity, giving rise to long-distance coordination between individual cells. Using methods from statistical physics and disordered systems [4], we discover a relation between the distance to criticality and the spatial dependence of the statistics of correlations. For networks close to the critical point, individual neuron pairs show significant long-range correlations even though average correlations decay much faster than the connectivity. The operation point of the network, for example its overall firing rate, controls the spatial range on which neurons cooperate, thus offering a potential dynamic mechanism that adapts the circuit to different computational demands.

**Acknowledgements**: upported by HGF young investigator’s group VH-NG-1028 and European Union Horizon 2020 grant 785907 (Human Brain Project SGA2).

**References**

1. Abeles M. Corticonics: Neural circuits of the cerebral cortex. *Cambridge University Press,* 1991.
2. Braitenberg V, Schüz A. Cortex: statistics and geometry of neuronal connectivity. *Springer Science & Business Media,* 2013.
3. Dahmen D, Grün S, Diesmann M, Helias M. Two types of criticality in the brain. *arXiv*, 1711.10930, 2017.
4. Hertz JA, Roudi Y, Sollich P. Path integral methods for the dynamics of stochastic and disordered systems. *Journal of Physics A: Mathematical and Theoretical* 2017, 50(3):033001.

## P119 Learning to learn on high performance computing

### Sandra Diaz-Pier^1^, Alper Yegenoglu^2^, Wouter Klijn^1^, Alexander Peyser^1^, Wolfgang Maass^3^, Anand Subramoney^4^, Giuseppe Visconti^4^, Michael Herty^4^

#### ^1^Jülich Research Centre, SimLab Neuroscience, Jülich, Germany; ^2^Jülich Research Centre, Institute of Neuroscience and Medicine (INM-6) & Institute for Advanced Simulation (IAS-6), Jülich, Germany; ^3^Graz University of Technology, Institute of Theoretical Computer Science, Graz, Austria; ^4^RWTH Aachen University, Institute of Geometry and Practical Mathematics, Department of Mathematics, Aachen, Germany

##### **Correspondence:** Sandra Diaz-Pier (s.diaz@fz-juelich.de)

*BMC Neuroscience* 2019, **20(Suppl 1)**:P119

Simulation of biological neural networks has become an essential part of neuroscience. The complexity of the structure and activity of the brain, combined with the limited access we have to measurements of in-vivo function of this organ, has led to the development of computational simulations which allows us to decompose, analyze and understand its elements and the interactions between them.

Impressive progress has recently been made in machine learning where brain-like learning capabilities can now be produced in non-spiking artificial neural networks [1, 3]. A substantial part of this progress arises from computing-intense learning-to-learn (L2L) [2, 4, 5] or meta-learning methods. L2L is a specific solution for acquiring constraints to improve learning performance.

The L2L conceptual world can be decomposed into an optimizee which learns specific tasks and an optimizer which searches for generalized hyperparameters for the optimizee. The optimizer learns to improve the optimizee’s performance over distinct tasks as measured by a fitness function (see Fig. 1).

**Fig. 1:**
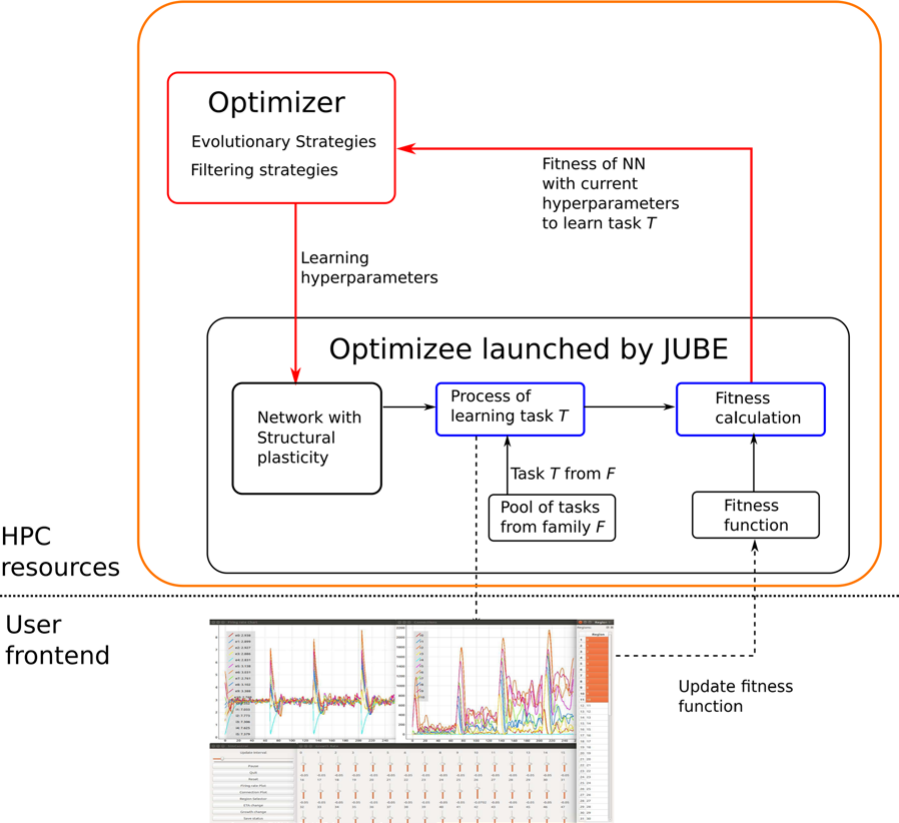
Learning-to-learn loop: Optimizee is an ensemble of machine learning instances over sets of hyperparameters and training samples from tasks

In this work we present an implementation of L2L which works on High Performance Computing (HPC) [6] for hyperparameter optimization of spiking neural networks. First, we discuss how the software works on a supercomputing environment. Taking advantage of the large parallelization which can be achieved by deploying independent instances of the optimizees on HPC, our L2L framework becomes a powerful tool for understanding and analyzing mathematical models of the brain. We also present preliminary results on optimizing NEST simulations with structural plasticity using a variety of optimizer algorithms e.g. gradient descent, cross entropy, evolutionary strategies. Finally, we discuss initial results on optimization algorithms designed specifically to work with spiking neural networks.

The L2L framework is flexible and can be also used for finding optimal configurations of generic programs, not only neural network simulations. Because of this, it can be applied in and outside of neuroscience.

**Acknowledgments:** This work has been partially funded by the Helmholtz Association through the Helmholtz Portfolio Theme “Supercomputing and Modeling for the Human Brain”. In addition, this work has received funding from the European Union’s Horizon 2020 research and innovation programme under grant agreement No 720270 (HBP SGA1) and No 785907 (HBP SGA2).

**References**

1. Lake BM, Ullman TD, Tenenbaum JB, Gershman SJ. Building machines that learn and think like people. *Behavioral and brain sciences* 2017;40.
2. Thrun S, Pratt L, editors. Learning to learn. *Springer Science & Business Media*; 2012 Dec 6.
3. Hutter F, Kotthoff L, Vanschoren J. Automatic machine learning: methods, systems, challenges. *Springer* 2018.
4. Andrychowicz M, Denil M, Gomez S, et al. Learning to learn by gradient descent by gradient descent. *In Advances in Neural Information Processing Systems* 2016 (pp. 3981–3989).
5. Jordan MI, Mitchell TM. Machine learning: Trends, perspectives, and prospects. *Science* 2015 Jul 17;349(6245):255–60.
6. Subramoney A, Diaz-Pier S, Rao A, et al. (2019, March 11). IGITUGraz/L2L: v1.0.0-beta (Version v1.0.0-beta). *Zenodo* http://doi.org/10.5281/zenodo.2590760

## P120 A novel method to encode sequences in a computational model of speech production

### Meropi Topalidou, Emre Neftci, Gregory Hickok

#### University of California, Irvine, Department of Cognitive Sciences, Irvine, CA, United States of America

##### **Correspondence:** Meropi Topalidou (mtopalid@uci.edu)

*BMC Neuroscience* 2019, **20(Suppl 1)**:P120

The ability to sequence at the phoneme, syllable, and word level is essential to speech production. Speech production models generally contain buffers or working-memory modules to encode sequences [2, 4] or use slots to label the kind of the unit [3]. The goal of this work is to propose a simple computational model of speech production that produces sequences using a biological plausible method, but also it has reduced spatial and temporal complexity compared to the existing ones. We propose a novel method where the sequences are encoded by the synaptic weights of the network, a feature shared by many connectionist models. The organization of the model is derived from psycholinguistic models that propose a higher-level lexical (abstract word) system and a lower-level phonological system. Accordingly, the proposed computational model contains a lexical and a motor-phonological structures bidirectionally connected to each other. These components map onto the cortical regions of posterior superior temporal sulcus/middle temporal gyrus (pSTS/pMTG) for the lexical component, and posterior inferior frontal gyrus (pIFG) for the motor-phonological component. Additionally, the model contains an inhibitory mechanism that simulates the interneurons in pIFG. The basic idea of the model is that the ``word’’ at the lexical level, and its ``phonemes’’ at the motor level are connected by synaptic weights, where the first element of the sequence is more strongly connected with the word than the second one and so on. This is essentially equivalent to what Karl Lashley proposed in 1951; the serial order of the sequence is encoded into the activity level of each unit. The architecture of the model eliminates the need for buffers or position slots used by other models [2, 3].

Another advantage of our model is that it does not include a separate working memory to explicitly store symbolic information. Both layers include a winner-take-all mechanism to ensure that only one unit will remain active. However, the inhibitory mechanism is an essential part of the model for producing sequences. The role of this mechanism is to be a “puppet master” during the production of each phoneme by inhibiting the more active unit so the second more active unit will be expressed.

Saying it differently, each neuron representing a phoneme should stay active until the production has been completed, but be silent after. Analysis of the network behavior showed that with this simple architecture, the model is sufficient to produce any word as a sequence of phonemes. Furthermore, this method can be embedded in a broader model of sensorimotor planning for speech production. A limitation of the model is that cannot represent a sequence with duplicated elements; although, this can be overstepped by adding hierarchical organization at the lexical layer. For example, the lower level will include all the known syllables in the language, and in upper levels will include the combinations of these syllables to more complex words. The different levels at the lexical layer can be linked by using the same mechanism presented here.

**References**

1. Hickok G. Computational neuroanatomy of speech production. *Nature Reviews Neuroscience* 2012 Feb;13(2):135.
2. Bohland JW, Bullock D, Guenther FH. Neural representations and mechanisms for the performance of simple speech sequences. *Journal of cognitive neuroscience* 2010 Jul;22(7):1504–29.
3. Foygel D, Dell GS. Models of impaired lexical access in speech production. *Journal of Memory and Language* 2000 Aug 1;43(2):182–216.
4. Grossberg S. A theory of human memory: Self-organization and performance of sensory-motor codes, maps, and plans. *In Studies of Mind and Brain* 1982 (pp. 498–639). Springer, Dordrecht.
5. Wilson DE, Smith GB, Jacob AL, et al. GABAergic neurons in ferret visual cortex participate in functionally specific networks. *Neuron* 2017 Mar 8;93(5):1058–65.

## P121 Origin of 1/f^β noise structure in M/EEG power spectra

### Rick Evertz^1^, David Liley^2^, Damien Hicks^3^

#### ^1^Swinburne University, Centre for Human Psychopharmacology, North Melbourne, Australia; ^2^University of Melbourne, Department of Medicine, Melbourne, Australia; ^3^Swinburne University, Department of Physics and Astronomy, Hawthorn, Australia

##### **Correspondence:** Rick Evertz (revertz@swin.edu.au)

*BMC Neuroscience* 2019, **20(Suppl 1)**:P121

Spectral analysis of magneto/electroencephalography (M/EEG) time series presents with a clearly pronounced alpha-band peak followed by a distinct S(f) = 1/f^β noise profile. The mechanistic origin of the alpha peak and its progenitor oscillation is an unresolved question in M/EEG research often thought to be dynamically unrelated to the S(f) = 1/f^β noise structure present in power spectra. Assuming that the measured M/EEG power spectrum can be modeled as a superposition of alpha-band relaxation processes with a distribution of dampings, the origin of the alpha peak and S(f) = 1/f^β noise profile can thus be explained via a singular generative mechanism. Within this framework, changes to the alpha peak and spectral noise profile are hypothesized to be a consequence of changes in the underlying damping distribution. We estimated the damping distributions for M/EEG power spectra computed from time series data that was recorded for multiple participants across a range of conditions. In practice this required solving a Fredholm integral equation of the first kind which was achieved through the use of second order Tikhonov regularization. The estimated damping distributions shared several robust features across multiple participants. The damping distributions were found to be multimodal with changes in EEG alpha peak between eyes closed and eyes open resting state, the result of a shift in the first mode of the distributions to a more heavily damped mode. The same were found for MEG power spectra where reductions in the alpha peak between resting and anesthesia (Xenon) states were observed. The shift in the most weakly damped distribution mode to more heavily damped one resulted in a direct reduction in the alpha peak. Furthermore, the bulk S(f) = 1/f^β properties of the M/EEG power spectra was replicated by using the regularized damping distributions in the forward model to generate an estimated power spectrum which fit the measured data remarkably well. The results demonstrate that the alpha peak and the S(f) = 1/f^β noise profile can be explained by a singular mechanism and changes to the spectral properties are a direct consequence of changes in the underlying damping distributions.

## P122 A neural mechanism for predictive optokinetic eye movement

### Ruben-Dario Pinzon-Morales, Shuntaro Miki, Yutaka Hirata

#### Chubu University, Robotics Science and Technology, Kasugai, Aichi, Japan

##### **Correspondence:** Yutaka Hirata (yutaka@isc.chubu.ac.jp)

*BMC Neuroscience* 2019, **20(Suppl 1)**:P122

This work deals with a sufficient mechanism for reproducing predictive eye velocity control known as predictive optokinetic response (OKR).

## P123 Evaluation of context dependency in VOR motor learning using artificial cerebellum

### Shogo Takatori, Keiichiro Inagaki, Yutaka Hirata

#### Chubu University, Robotics Science and Technology, Kasugai, Aichi, Japan

##### **Correspondence:** Shogo Takatori (tr18009-0255@sti.chubu.ac.jp)

*BMC Neuroscience* 2019, **20(Suppl 1)**:P123

The vestibuloocular reflex (VOR) maintains stable vision during head motion by counter rotating the eyes in the orbit. The VOR has been a popular model system to investigate neural mechanism of motor learning as its gain defined as eye velocity / head velocity is easily modifiable by visual-vestibular mismatch stimuli. When visual stimulation is given in-phase or out-of-phase with head motion for 10 min or longer, VOR gain measured in darkness w/o visual stimulation decreases or increases, respectively. As many other biological adaptive motor control systems, VOR motor learning is context dependent [1]. For example, VOR gain increase and decrease can be induced simultaneously for different head rotation directions. Namely, by applying visual stimulus out-of-phase with leftward head rotation and in-phase with rightward head rotation (L-Enh/R-Sup stimulus), VOR gain in dark during left and rightward head rotation respectively increases and decreases. It has been shown that long-term depression (LTD) and long-term potentiation (LTP) at the parallel fiber (PF)–Purkinje cell (PC) synapses in the cerebellum play major roles in VOR motor learning. However, how cerebellar neuronal circuitry incorporating those LTD and LTP achieves head direction dependent VOR motor learning is still unknown. Presently, we investigated the effect of directional context in the VOR motor learning, using the artificial cerebellum that we have been developing and modifying for the past decade [2]. Our artificial cerebellum having a bihemispheric structure was utilized for simulations of head direction dependent VOR motor learning. For that, the non-cerebellar neural pathways subserving VOR are described by transfer functions based on physiological experimental results in squirrel monkey. The cerebellar flocculus neuronal network was constructed based on the known anatomical and physiological evidence by spiking neuron models. LTD and LTP between PF–PC were described in spike timing dependent plasticity. Directional dependent VOR motor learning was induced in the model after 2-hour L-Enh/R-Sup training. A simple possible mechanism to achieve this head direction selective VOR motor learning is that the cerebellar left hemisphere is responsible for VOR gain increase during leftward head rotation and the right hemisphere is for gain decrease during rightward rotation. We showed that this scenario is unlikely because substituting PF-PC synaptic weights in the left hemisphere with those acquired by ordinary VOR gain increase training and those in the right hemisphere with those after ordinary gain decrease training did not reproduce the directional dependent VOR gain changes. These results suggest that the need of learning of directional context to achieve directional context dependent VOR gain change. Our results also indicated that mechanism for context dependent VOR motor learning differs from ordinary VOR gain increase and decrease learning.

**Acknowledgement:** A part of this work was supported by JSPS KAKENHI Grant Number 17K12781.

**References**

1. Yoshikawa A, Hirata Y. Different mechanisms for gain-up and gain-down vestibuloocular reflex motor learning revealed by directional differential learning tasks. *The IEICE transactions on information and systems* 2009, J92-D, pp.176–185.
2. Takatori S, Inagaki K, Hirata Y. Realization of direction selective motor learning in the artificial cerebellum: simulation on the vestibuloocular reflex adaptation. *IEEE EMBC* 2018.

## P124 A computational model of the spontaneous activity of gonadotropin-releasing cells in the teleost fish medaka

### Geir Halnes^1^, Simen Tennøe^2^, Gaute Einevoll^1^, Trude M. Haug^3^, Finn-Arne Weltzien^4^, Kjetil Hodne^4^

#### ^1^Norwegian University of Life Sciences, Faculty of Science and Technology, Aas, Norway; ^2^University of Oslo, Department of Informatics, Oslo, Norway; ^3^University of Oslo, Institute of Oral Biology, Oslo, Norway; ^4^Norwegian University of Life Sciences, Department of Basic Sciences and Aquatic Medicine, Aas, Norway

##### **Correspondence:** Geir Halnes (geih@nmbu.no)

*BMC Neuroscience* 2019, **20(Suppl 1)**:P124

Pituitary hormone producing gonadotrope cells can fire spontaneous action potentials (APs). The hormone-release rate is proportional to the cytosolic Ca2+ concentration, which is regulated by release from intracellular stores (ER), and/or influx through Ca2+ channels on the plasma membrane. While ER-Ca2+ release normally requires G-protein activation, Ca2+ influx through the plasma membrane relies largely on the intrinsic firing properties of the cell. The spontaneous activity is partly important for the re-filling of ER, but may also give rise to a basal hormone secretion rate [1]. Pituitary APs are typically generated by TTX-sensitive Na+ currents (INa), high-voltage activated Ca2+ currents (ICa), or by a combination of the two [1]. Previous computational models have focused on conditions where spontaneous APs are predominantly mediated by ICa. This is representative for many pituitary cells, but not all (see [2] and refs. therein).

Here, we present a computational model of a gonadotrope cell in the teleost fish medaka, which fire INa-dependent spontaneous APs. The model contains a leak conductance, two depolarizing channels (INa and ICa) that mediate the AP upstroke, and three hyperpolarizing K+-channels that shape the downstroke of the AP. The leakage- and K+- channels were adapted from a previous study [3], while the kinetics of INa and ICa were adapted to new voltage-clamp data. The channel conductances were constrained to current-clamp recordings under control conditions, after TTX application, and after application of the BK-channel blocker paxilline. We compare the model to previous pituitary cell models (based on data from rats and mice), and perform a sensitivity analysis of the model by using the toolbox UncertainPy [4]. Although the model was constrained to experimental data from gonadotrope cells in medaka, we anticipate that modified versions of it will be useful for describing also other pituitary cells that fire INa-mediated APs.

**Acknowledgements**: This work was funded by the Research Council of Norway via the BIOTEK2021 project “DigiBrain”, grant no 248828, and the Aquaculture program, grant no 244461.

**References**

1. Stojilkovic SS, Tabak J, Bertram R. Ion channels and signaling in the pituitary gland. *Endocrine reviews* 2010 Dec 1;31(6):845–915.
2. Halnes G, Tennøe S, Haug TM, Einevoll GT, Weltzien FA, Hodne K. BK channels have opposite effects on sodium versus calcium-mediated action potentials in endocrine pituitary cells. *bioRxiv* 2018 Jan 1:477976.
3. Tabak J, Tomaiuolo M, Gonzalez-Iglesias AE, Milescu LS, Bertram R. Fast-activating voltage-and calcium-dependent potassium (BK) conductance promotes bursting in pituitary cells: a dynamic clamp study. *Journal of Neuroscience* 2011 Nov 16;31(46):16855–63.
4. Tennøe S, Halnes G, Einevoll GT. Uncertainpy: A Python toolbox for uncertainty quantification and sensitivity analysis in computational neuroscience. *Frontiers in neuroinformatics* 2018;12.

## P125 Neural transmission delays and predictive coding: Real-time temporal alignment in a layered network with Hebbian learning

### Anthony Burkitt^1^, Hinze Hogendoorn^2^

#### ^1^University of Melbourne, Department of Biomedical Engineering, Melbourne, Australia; ^2^University of Melbourne, Melbourne School of Psychological Sciences, Melbourne, Australia

##### **Correspondence:** Anthony Burkitt (aburkitt@unimelb.edu.au)

*BMC Neuroscience* 2019, **20(Suppl 1)**:P125

The transmission of information in neural systems inherently involves delays, which results in our awareness of sensory events necessarily lagging behind the occurrence of those events in the world. In the absence of some mechanism to compensate for these delays, our visual perception would consistently mislocalize moving objects behind their actual position. Anticipatory mechanisms that might compensate for these delays have been hypothesized to underlie perceptual effects in humans such as the Flash-Lag Effect. However, there has been no consistent neural modelling framework that captures these phenomena.

By extending the predictive coding framework to take account of the delays inherent in neural transmission, we have proposed a real-time temporal alignment hypothesis [1]. In this framework both the feed-forward and feedback extrapolation mechanisms realign the feedback predictions to minimize prediction error. The consequence is that neural representations across all hierarchical stages become aligned in real-time.

In order to demonstrate real-time temporal alignment in a layered network of neurons, we consider a network architecture in which the location of a moving stimulus is encoded at each layer of the network by a population code for both the position and velocity of the stimulus. There are N position sub-populations at each layer, each with an identical Gaussian distribution and each containing M velocity sub-populations. The sub-populations are connected by both feed-forward and feedback weights. The excitatory feed-forward weights between the neural populations at each layer and subsequent layer are learned by a Hebbian rule and normalization is imposed.

Using this model, we explore the key mechanisms of neural coding and synaptic plasticity necessary to generate real-time alignment of neural activityin a layered network. We demonstrate how a moving stimulus generates a representation of the position and velocity of the stimulus in the higher levels of the network that maintains the real-time representation of the stimulus, accounting for the neural processing delay associated with the transmission of information through the network. This neural population code alignment provides a solution to the temporal binding problem, since the neural population activity remains in real-time temporal alignment with the moving stimulus that generates the input to the network. Second, we show that this real-time population neural code can prime the appropriate neural sub-population that is consistent with a constantly moving stimulus. This priming of the neural activity in alignment with a moving stimulus provides a parsimonious explanation for several known motion-position illusions [2].

In summary, this study uses visual motion as an example to illustrate a neurally plausible model of real-time temporal alignment. This model is consistent with evidence of extrapolation mechanisms throughout the visual hierarchy, it predicts several known motion-position illusions in human observers, and that it provides a solution to the temporal binding problem.

**References**

1. Hogendoorn H, Burkitt AN. Predictive coding with neural transmission delays: a real-time temporal alignment hypothesis. *bioArxiv* 2018, doi:http://dx.doi.org/10.1101/453183
2. Hogendoorn H, Burkitt AN. Predictive coding of visual object position ahead of moving objects revealed by time-resolved EEG decoding, *NeuroImage* 2018 171: 55–61.

## P126 Emergence of ‘columnette’ orientation map in mouse visual cortex

### Peijia Yu, Brent Doiron, Chengcheng Huang

#### University of Pittsburgh, Department of Mathematics, Pittsburgh, PA, United States of America

##### **Correspondence:** Peijia Yu (yupeijia.qbio@gmail.com)

*BMC Neuroscience* 2019, **20(Suppl 1)**:P126

The orientation selectivity of neurons in the primary visual cortex (V1) of higher mammals, such as primates and cats, are spatially arranged in columnar maps. In contrast, the V1 of rodents are believed to have no clear spatial organization, and rather form a ‘salt-and-pepper’ style organization. However, [1] recently showed that the tuning similarity of pyramidal neurons in mouse V1 decreases with cortical distance, indicating a weak spatial clustering of tuning, instead of a strict salt-and-pepper map (Fig. 1a) [1].

**Fig. 1:**
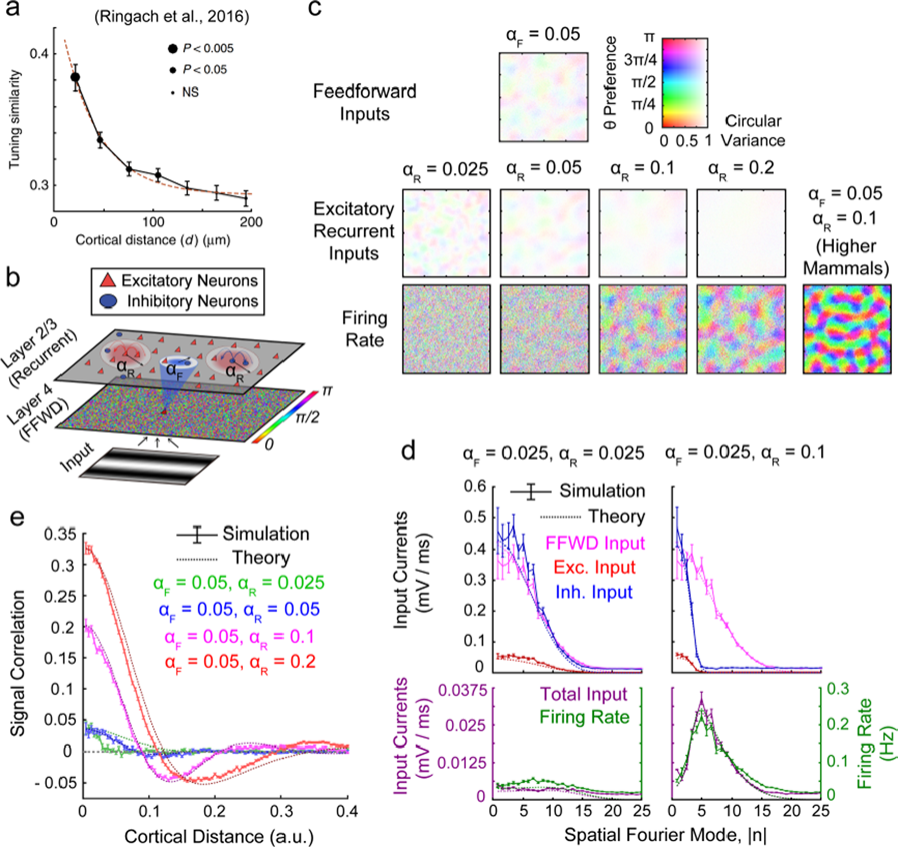
**a** Tuning similarity of pyramidal neurons in mouse V1 [1]. **b** Schematic of the network model. **c** Spatial patterns of preferred orientations of excitatory neurons, under different alpha_R. **d** Input currents and firing rate of excitatory neurons as a function of the magnitude of spatial Fourier mode. **e** Signal correlation as a function of cortical distance

To study the emergence of spatial organization of orientation tuning, we model the layer (L) 4 and layer (L) 2/3 of rodent V1 with a network of spiking neurons (Fig. 1b). The tuning curves of L4 neurons are homogeneous, with preferred orientations randomly assigned without any spatial correlation (i.e. ‘salt-and-pepper’). The L2/3 network consists of excitatory and inhibitory neurons receiving feedforward input from L4 neurons, and lateral recurrent inputs. The probability of both feedforward and recurrent connectivity decays with physical distance, which obey 2D Gaussian-shaped function with average widths alpha_F and alpha_R, respectively.

We found that when the network has strong, yet balanced, excitatory and inhibitory interactions, even though feedforward and recurrent inputs to L2/3 neurons are weakly tuned due to spatial filtering, L2/3 neurons can be orientation selective. This is consistent with previous studies [2, 3]. Surprisingly, spatial clustering of similarly tuned neurons emerges in L2/3 when recurrent connections are broader than feedforward connections (alpha_R>alpha_F), which resembles the columnar maps of higher mammals, though weaker. We name this pattern ‘columnette’ (Fig. 1c).

This result could be intuitively interpreted in the spatial Fourier space: Both feedforward and recurrent input currents have low-pass structure in the Fourier domain, due to the spatial filtering of a Gaussian-shaped connectivity footprint. Their summation can be either low-pass when alpha_R< = alpha_F (Fig. 1d, left panel), or band-pass when alpha_R>alpha_F (Fig. 1d, right panel), which corresponds to a clustered pattern in the physical space.

Furthermore, we predict that the signal correlation between neurons decreases with distance (Fig. 1e). Especially, when alpha_R>alpha_F and ‘columnette’ emerges, the signal correlation shows non-monotonic dependence on distance.

Previous models of orientation maps typically use long-range lateral inhibition which gives rise to strong columnar periodicity [4]. In contrast, we show that in networks with spatially balanced excitatory and inhibitory connections, ‘columnette’, a weak columnar structure, can emerge without any feature based spatial organization of either feedforward inputs or recurrent coupling.

**References**

1. Ringach DL, Mineault PJ, Tring E, et al. Spatial clustering of tuning in mouse primary visual cortex. *Nature communications* 2016, 7, 12270.
2. Hansel D, van Vreeswijk C. The mechanism of orientation selectivity in primary visual cortex without a functional map. *Journal of Neuroscience* 2012, 32(12), 4049–4064.
3. Pehlevan C, Sompolinsky H. Selectivity and sparseness in randomly connected balanced networks. *PLoS One* 2014, 9(2), e89994.
4. Kaschube M, Schnabel M, Lowel S, et al. Universality in the evolution of orientation columns in the visual cortex. *Science* 2010, 330(6007), 1113–1116.

## P127 Decoupled reaction times and choices in expectation-guided perceptual decisions

### Lluís Hernández-Navarro^1^, Ainhoa Hermoso-Mendizabal^1^, Jaime de la Rocha^2^, Alexandre Hyafil^3^

#### ^1^IDIBAPS, Barcelona, Spain; ^2^IDIBAPS, Theoretical Neurobiology, Barcelona, Spain; ^3^UPF, Center for Brain and Cognition, Barcelona, Spain

##### **Correspondence:** Lluís Hernández-Navarro (llhernandez@clinic.cat)

*BMC Neuroscience* 2019, **20(Suppl 1)**:P127

In perceptual categorization tasks, both reaction times (RTs) and choices not only depend on current stimulus information, but also on urgency and prior expectations. To study them, we trained 10 rats in a two-alternative forced choice auditory discrimination task in free-response paradigm. The standard Drift Diffusion Model (DDM) for evidence accumulation up-to-threshold predicts a modulation of RTs by evidence strength. However, rats showed stimulus-independent RTs for fast, ‘express’ responses (RT<80ms,≈35% of trials). On the other hand, rats’ express choices were clearly modulated by stimuli because their express performance was significantly above chance (also for unbiased trials), and increased with RT. Additionally, in≈20% of trials, rats aborted fixation close to the onset of the stimulus, i.e. fixation break (FB, unrewarded).

The stimulus-independent express RTs, FBs and the increase of performance with RT for unbiased trials are inconsistent with standard DDMs for decision-making. Therefore, we propose a novel variant in which rats’ responses are triggered by independently integrating time and evidence. In this Dual DDM (2DM), time is tracked by a single-threshold DDM with constant bound initiating before the stimulus onset. This time integrator acts as both, an anticipation signal and an urgency signal. The evidence integrator is a standard DDM (two-threshold, constant-bound) that starts integrating sensory evidence some sensory delay after stimulus onset. The response of the rat is triggered when a bound is reached (either time-bound or evidence-bound). The choice of the rat is always set by the accumulated evidence at response time. The unconstrained fit of the 2DM to the full RT distributions provides initial, strong and consistent evidence across rats for the dual nature of their decision process.

We also introduced correlations in the stimulus sequence to induce trial-dependent expectations to repeat or alternate the previous response. We first found that, surprisingly, post-error slowing arose from two distinct phenomena: a slowing of the time integrator, and a lower stimulus sensitivity (i.e. slower integration to threshold) of the evidence integrator. Also, as expected, the evidence integrator was strongly influenced by history biases. We were able to decouple the contribution of the ‘lateral bias’ (i.e. accumulated ‘win-stay’ side bias) and the ‘transition bias’ (i.e. accumulated bias to repeat or alternate the previous response) on rats’ decisions. By maximum likelihood fitting (with L2 regularization) of the 2DM to rats’ choices, we consistently found that the lateral bias arises as a constant bias in the drift of the evidence integrator, whereas the transition bias is implemented as an initial offset of the evidence integrator.

We also found an unexpected modulation of the time integrator with history biases: it was slower under an expectation to repeat, while it became faster under an expectation to alternate. Preliminary results seem to support a distinct impact of the lateral and the transition bias also on the time integrator.

In conclusion, current standard models of decision making predict a direct relation between evidence accumulation and RTs, which is inconsistent with experimental observations in rats. A novel dual model, grounded on an independent integration of time and evidence, is able to capture rat’s behavior, and even decouple the impact of distinct history biases on RTs and choices.

## P128 V1 visual neurons: receptive field types vs spike shapes

### Syeda Zehra^1^, Hamish Meffin^2^, Damien Hicks^3^, Tatiana Kameneva^1^, Michael Ibbotson^4^

#### ^1^Swinburne University of Technology, Telecommunication Electrical Robotics and Biomedical Engineering, Melbourne, Australia; ^2^University of Melbourne, Department of Optometry and Visual Science, Melbourne, Australia; ^3^Swinburne University, Department of Physics and Astronomy, Hawthorn, Australia; ^4^National Vision Research Institute, University of Melbourne, Melbourne, Australia

##### **Correspondence:** Tatiana Kameneva (tkameneva@swin.edu.au)

*BMC Neuroscience* 2019, **20(Suppl 1)**:P128

People with retinitis pigmentosa (RP) and age-related macular degeneration (AMD) lose retinal cells called photoreceptors that convert light energy into electro-chemical signals. However, many other types of retinal neurons survive in RP and AMD. It is possible to return a rudimentary vision to people with these diseases by stimulating remaining neurons in the retina with small electrical currents via an implanted electrode array. To improve efficacy of visual prostheses it is important to understand electrophysiology of different classes of visual neurons.

We used machine learning techniques to divide previously recorded data into clusters. We analyzed if the clusters discovered using the machine learning technique corresponded to the cell receptive field classifications. Extracellular recordings with 32 electrode array were collected from 189 V1 cortical neurons in anaesthetised cats. For each cell, a spike with the largest amplitude (out of 32 recordings) was analysed. White noise light stimulation protocol was implemented to classify receptive field size for each cell. Recorded extracellular spikes were spike sorted and used for clustering analysis. Wavelet decomposition was used to decompose experimentally recorded data into coefficients at five levels (the number of levels was based on the number of samples in the data). The coefficients at levels 3, 4 and 5 were used as input into K-means algorithm to classify data into clusters. The number of clusters in the algorithm was chosen to match six receptive field types.

Results show that clusters found by wavelet decomposition have some overlap with the receptive field types, i.e. cells with the same receptive field types have similar shapes of extracellular spikes. The clusters can be divided into triphasic slow, triphasic fast, double spikes, upwards, biphasic and fast spikes. In addition, the extracellular spikes were clustered into fast and slow groups which corresponded to previously published results for cortical visual neurons.

Understanding the differences in electrophysiological properties between V1 neurons is important for the advancement of basic neuroscience. In addition, our results may have an important implication on the development of stimulation strategies for visual prostheses.

## P129 Synaptic basis for contrast-dependent shifts in functional cell identity in mouse primary visual cortex

### Molis Yunzab^1^, Veronica Choi^2^, Hamish Meffin^1^, Shaun Cloherty^3^, Nicholas Priebe^2^, Michael Ibbotson^1^

#### ^1^National Vision Research Institute, Melbourne, Australia; ^2^University of Texas Austin, Centre for Learning and Memory, Austin, United States of America; ^3^Monash University, Department of Physiology, Clayton, Australia

##### **Correspondence:** Molis Yunzab (molisyunzab@me.com)

*BMC Neuroscience* 2019, **20(Suppl 1)**:P129

Neurons in the mammalian primary visual cortex (V1) are classically labelled as either simple or complex based on their response linearity. A fundamental transformation that occurs in the mammalian visual cortex is the change from linear, polarity-sensitive responses of simple cells to nonlinear, polarity-insensitive responses of complex cells. While the difference between simple and complex responses is clear when the stimulus strength is high, reducing stimulus strength (e.g. contrast) diminishes the differences between the two cell types and causes some complex cells to respond as simple cells. This contrast-dependent transformation has been observed in extracellularly recorded spiking responses in V1 of mouse, cat and monkey. However, the mechanism underlying the phenomenon is unclear. In this study, we first explored two models that could potentially explain the contrast-dependent transformation and then examined the signature of the potential models by recording both the spiking and subthreshold responses of mouse V1 neurons using in vivo whole cell recordings. In the first candidate model the contrast-dependent shifts in complex cell responses emerge due to the “iceberg” effect, generated by the biophysical spike threshold, in which not all synaptic responses are converted into spikes at low contrast. However, we found systematic shifts in the degree of complex cell responses in mouse V1 at the subthreshold level, demonstrating that synaptic inputs change in concert with the shifts in response linearity and that this change cannot be explained with a simple threshold nonlinearity model. In the second candidate model recurrent amplification of the network acts as a critical component in generating linear or nonlinear responses in complex cells when input gain is low or high, respectively [1]. This model predicts that both spiking and subthreshold responses undergo contrast-dependent shifts in response linearity. Our experimental data confirms that this is the case in mouse V1 neurons. In conclusion, while the threshold nonlinearity may play an additional role in altering the response linearity of neurons [2], there is a clear synaptic component to the shift in response linearity that is likely driven by the changing recurrent inputs received from the cortical network.

**References**

1. Chance FS, Nelson SB, Abbott LF. Complex cells as cortically amplified simple cells. *Nature Neuroscience* 1999, 2, 277–282.
2. Priebe NJ, Mechler F, Carandini M, Ferster D. The contribution of threshold to the dichotomy of cortical simple and complex cells. *Nature Neuroscience* 2004, 7, 1113–1122.

## P130 An encoding mechanism for translating between temporal sequences and spatial patterns

### Nathalia Cristimann^1^, Gustavo Soroka^2^, Marco Idiart^1^

#### ^1^Universidade Federal do Rio Grande do Sul, Institute of Physics, Porto Alegre, Brazil; ^2^Universidade Federal do Rio Grande do Sul, Instituto de Ciências Básicas da Saúde, Porto Alegre, Brazil

##### **Correspondence:** Nathalia Cristimann (nmcristimann@gmail.com)

*BMC Neuroscience* 2019, **20(Suppl 1)**:P130

There are evidences that different brain networks may have distinct forms of holding information, both in terms of mechanism and coding. In particular, when modeling memory function in the brain, two theoretical frameworks have been used: recurrent attractor networks and bistability based working memory buffers. Recurrent attractor networks store information in the synaptic connections, and memory is a network property. On the other hand, working memory buffers may rely on short-lived changes, sometimes at single cell level, and have a much lower storage capacity that can be circumvent, for instance, by a multiplexing code like the theta-gamma temporal code. Moreover, while it is likely that recurrent networks could present an irregular asynchronous state the same may not be true of the working memory buffers of the theta-gamma kind where synchrony is an essential feature. But ultimately if both networks are to be present in the brain they need to communicate to exchange information. In this work we propose a mechanism using inhibitory competition that provides a satisfactory functional coupling between such different forms of information storage and processing. We focus in the simpler case of a neural architecture comprised of two working memory buffers that interact via a recurrent neural network (RNN) that is capable of holding long term memories as attractors. In the network the temporal sequence coming from the input buffer is stored as a spatial pattern in the RNN, and subsequently decoded as a temporal pattern in the output buffer. We investigate its encoding and decoding capabilities in presence of noise and incomplete information. We also address the question of whether a random network structure in RNN could be sufficient to guarantee information transfer between the two buffers. We explore 4 models of random connectivity: Erdos-Renyi (ER), Watts-Strogatz (WS), Newmann-Watts-Strogatz (NWS) and Barabasi-Albert (BA). Using as a metric for the encoding/decoding error the edit distance between the output and input sequences, we show that the WS and NWS models, which correspond to networks that have small-world properties, are more efficient than the other models. When compared to the ER model, the WS and NWS models present a smaller error for almost every value of the connectivity parameters.

## P131 Building Python interactive neuroscience applications using Geppetto

### Matteo Cantarelli^1,2^, Padraig Gleeson^5^, Adrian Quintana^1,3^, Angus Silver^5^, William W Lytton^6^, Salvador Dura-Bernal^6^, Facundo Rodriguez^1^, Bóris Marin^7,5^, Robert Court^4^, Matt Earnshaw^5^, Giovanni Idili^1,2^

#### ^1^MetaCell Ltd. LLC, Oxford, UK/Boston, USA; ^2^OpenWorm Foundation, Delaware, USA; ^3^EyeSeeTea Ltd., London, UK; ^4^Institute for Adaptive and Neural Computation, University of Edinburgh, UK; ^5^University College London, Dept. of Neuroscience, Physiology & Pharmacology, London, United Kingdom; ^6^State University of New York Downstate Medical Center, Brooklyn, NY, USA; ^7^Universidade Federal do ABC, São Bernardo do Campo, Brazil

##### **Correspondence:** Matteo Cantarelli (matteo@metacell.us)

*BMC Neuroscience* 2019, **20(Suppl 1)**:P131

Geppetto [1] is an open-source platform for building web applications for visualizing neuroscience models and data, as well as managing simulations. Geppetto underpins a number of neuroscience applications available to the research community, including Open Source Brain (OSB) [2], Virtual Fly Brain (VFB) [3], NetPyNE-UI [4] and HNN-UI [5]. While Geppetto traditionally employed a Java backend we have now augmented it to also support Python. This means that applications built with Geppetto now also offer their users the ability to interact directly with any underlying Python APIs, while seamlessly keeping the user interface synchronized. To make this possible we developed a series of Javascript-Python Connectors that let developers easily build a user interface, whose state can be controlled from a Python model and vice versa. Neuroscience applications built with Python Geppetto have the advantage of bridging the beginner/advanced user usability gap. Beginner users will be able to interact with a user interface that will simplify the accessibility of the underlying APIs. Expert users will be able from the same GUI to programmatically interact with the underlying data models and Python APIs while the user interface will be kept updated graphically reflecting any programmatic changes. Python Geppetto applications can be deployed locally, installed using standard Python packages (accessible from PyPI) or Docker and deployed remotely on the web using Kubernetes and Jupyter Hub.

**Fig. 1:**
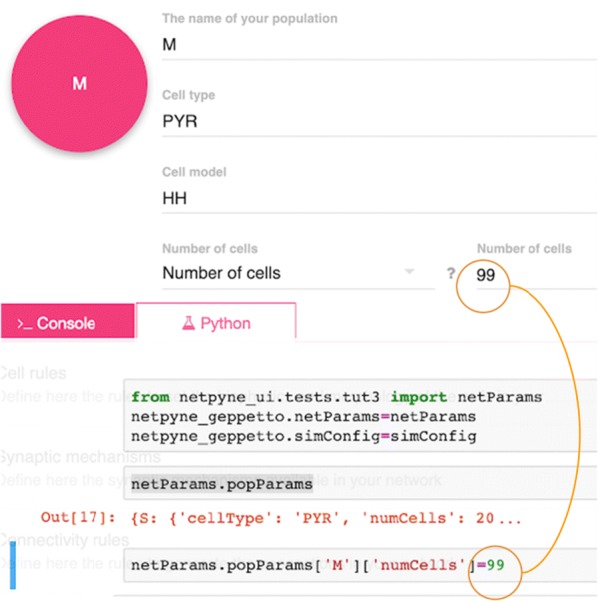
NetPyNE-UI [4] as an example of an application built with Python Geppetto. In the screenshot the number of cells for population M was programmatically changed via an integrated Jupyter notebook, causing the GUI to automatically update

**References**

1. Cantarelli M, et al. Geppetto: a reusable modular open platform for exploring neuroscience data and models. *Philosophical Transactions of the Royal Society B: Biological Sciences* 2018 Sep 10;373(1758):20170380.
2. Gleeson P, et al. Open Source Brain: a collaborative resource for visualizing, analyzing, simulating and developing standardized models of neurons and circuits. *bioRxiv* 2018 Jan 1:229484. (10.1101/229484)
3. Armstrong JD, et al. Towards a virtual fly brain. *Philosophical Transactions of the Royal Society A: Mathematical, Physical and Engineering Sciences* 2009 Jun 13;367(1896):2387–97. (10.1098/rsta.2008.0308)
4. Dura-Bernal S, et al. NetPyNE: a tool for data-driven multiscale modeling of brain circuits. *bioRxiv* 461137(2018) [Preprint]; (Under review in eLife)
5. Neymotin S, et al. Human Neocortical Neurosolver. 2018. 10.5281/zenodo.1446517

## P132 Hierarchy of inhibitory circuit acts as a switch key for network function in a model of the primary motor cortex

### Heidarinejad Morteza^1^, Zhe Sun^1^, Jun Igarashi^1^

#### ^1^Riken, Computational Engineering Applications Unit, Saitama, Japan

##### **Correspondence:** Heidarinejad Morteza (s.heidarinejadmohamm@riken.jp)

*BMC Neuroscience* 2019, **20(Suppl 1)**:P132

The primary motor cortex (M1) is the core region for body action and movements. Here we have been constructed a large-scale real spiking neural network of M1, based on anatomical and electrophysiological data [1, 2]. The model includes 5 layers L1, L23, L5A, L5B, and L6 and 19 different cell types. Spatial extents and connection probabilities among the neurons were estimated from experimental Laser Scanning Photo Stimulation (LSPS) data and unitary synaptic connections.

First, the virtual LSPS experiment was conducted. Those already reported by experiments [3], repeated by our results, while other non-reported maps were reported by our stimulation.

Also, we conducted columnar shape stimulation to inhibitory neurons. To elucidate the application of such a structure, we assumed a vertical cylinder and applied the stimulation on neurons inside. All neurons of 5 populations of L1-ENGC, L1-SBC, L2/3-PV, L2/3-SST, and L2/3-VIP were stimulated and spiking activity of all neurons was recorded.

As a result, SBC, PV, and SST interneurons were categorized as local inhibitors. Projection of all these 3 interneurons were confined to the inside of assumed column. In contrast, stimulation of layer 1 ENGC and layer 2/3 VIP interneurons showed both vertical and horizontal propagation. ENGC cells almost inhibited all neurons of layer 1 and 2/3, plus SST neurons of all layers and VIP interneurons activated all neuron types including PV neurons in all layers (excluding layer 1) except SST neurons which are inhibited by VIPs.

Inhibition of inhibition is a well-known logic to control the activity of cortex. ENGCs and SBCs in layer 1 and SSTs of layer 2/3 inhibit VIP neurons, while VIP interneurons themselves have a versatile impact on others. The results suggest that VIP neurons may act as a switch for activation of inhibition on entire cortical network.

**References**

1. Fino E, Yuste R. Dense inhibitory connectivity in neocortex. *Neuron* 2011 Mar 24;69(6):1188–203.
2. Jiang X, Shen S, Cadwell CR, et al. Principles of connectivity among morphologically defined cell types in adult neocortex. *Science* 2015 Nov 27;350(6264):aac9462.
3. Hooks BM, Hires SA, Zhang YX, et al. Laminar analysis of excitatory local circuits in vibrissal motor and sensory cortical areas. *PLoS Biology* 2011 Jan 4;9(1):e1000572.

## P133 Spatially organized connectivity for signal processing in a model of the rodent primary somatosensory cortex

### Zhe Sun^1^, Heidarinejad Morteza^1^, Jun Igarashi^1^

#### ^1^Riken, Computational Engineering Applications Unit, Saitama, Japan

##### **Correspondence:** Zhe Sun (zhe.sun.vk@riken.jp)

*BMC Neuroscience* 2019, **20(Suppl 1)**:P133

Understanding the structure and function of S1 is critical for figuring out the information process mechanism in the sensory nervous system. Spatial organization of connections, layers, and columns in the somatosensory cortex is considered to work as information processing device for integration of inputs and selection of outputs. However, it remains unknown how different types of connections with different spatial extent work for sensory processing in the primary somatosensory cortex (S1). To investigate it, we developed a three-dimensional model of spiking neural network model of the S1 based on anatomical and electrophysiological experiment results [1, 2]. The S1 model comprised 7 layers, with 1 excitatory neuron and 5 inhibitory neuron types (L1: 2 inhibitory neuron types; L2 and L3: 3 inhibitory and 1 excitatory neuron types; L4, L5A, L5B&L6: 2 inhibitory and 1 excitatory neuron types). We used the layer thicknesses and the cell densities of the mouse’s S1 data. Leaky integrate-and-fire neuron model was used for all neuron types. We used the information of spatial extents, probabilities, and connectivity from the reports of laser-scanning photo-stimulation (LSPS) experiments and patch clamp recordings. We used Gaussian function as the connection probability function. All simulations were performed using pyNEST 2.16 on HOKUSAI supercomputer in RIKEN. The simulation time step was set to 0.1 ms. When we performed a size of 1 mm^2^ S1 simulation on one compute node with 40 CPU cores, it took 6 minutes to construct the network. And it also took 0.5 minute to complete the simulation of 1 second of neuronal network activity in real biological time. Total neuron number in the 1mm^2^ microcircuit is 94396.By adjusting external Poisson input for each kind of neuron, we realized resting state firing rate for all neuron types in our S1 model. We made a virtual slice of the S1 whose shape was a cube of 1600 × 400 × 1400 micron. We first performed virtual LSPS experiments for excitatory and inhibitory connections to all neuron types. The responses of neurons to LSPS were qualitatively similar to those in real LSPS experiments. Most importantly, to investigate the relation between excitatory and inhibitory signals, we compared the excitatory and inhibitory conductance with changing distances between neurons with external stimulation and recorded neurons. The excitatory and inhibitory synaptic conductance of L2/3 and L5 excitatory neurons similarly decayed with increasing in the horizontal distance between stimulation sites and positions of recorded neurons, which is similar to real experimental results [3]. These results suggest that spatial extents of different connections may cause spatially coupled excitation and inhibition in L2/3 and L5A, which may lead to cooperative information processing by excitation and inhibition.

**Fig. 1:**
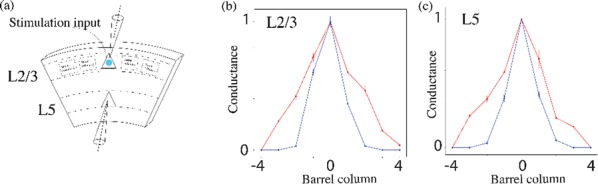
**a** Experiment of spatial interaction between excitatory and inhibitory signals in S1. The width of a virtual column is around 200 microns. **b** The excitatory and inhibitory synaptic conductance of L23 pyramidal cells in different barrel columns. **c** The excitatory and inhibitory synaptic conductance of L5 pyramidal cells

**Acknowledgement:** This work was supported by the Ministry of Education, Culture, Sports, Science and Technology(MEXT) as ”Exploratory Challenge 4 on Post-K Computer”.

**References**

1. Kätzel D, Zemelman BV, Buetfering C, Wölfel M, Miesenböck G. The columnar and laminar organization of inhibitory connections to neocortical excitatory cells. *Nature Neuroscience* 2011 Jan;14(1):100.
2. Hooks BM, Hires SA, Zhang YX, et al. Laminar analysis of excitatory local circuits in vibrissal motor and sensory cortical areas. *PLoS Biology* 2011 Jan 4;9(1):e1000572.
3. Adesnik H, Scanziani M. Lateral competition for cortical space by layer-specific horizontal circuits. *Nature* 2010 Apr;464(7292):1155.

## P134 Probing the association between axonal sprouting and seizure activity using a coupled neural mass model

### Jaymar Soriano^1^, Takatomi Kubo^2^, Kazushi Ikeda^2^

#### ^1^University of the Philippines, Department of Computer Science, Quezon City, Philippines; ^2^Nara Institute of Science and Technology, Ikoma, Japan

##### **Correspondence:** Jaymar Soriano (jbsoriano@gmail.com)

*BMC Neuroscience* 2019, **20(Suppl 1)**:P134

Initiation of seizure activity in the brain is generally believed to be caused by an alteration in excitation-inhibition balance such as when dendritic inhibition is impaired. Alternatively, it is also believed that seizure activity can arise due to a synaptic reorganization of neural networks such as the emergence of axonal sprouting in which axonal processes of a neuron grow out and create synaptic connections with the dendritic processes of other neurons. In fact, co-occurrence of seizure activity and axonal sprouting has been established in epilepsy and lesion models. For example, Cavazos et al. [1] report that alterations in terminal projections of mossy fiber pathway progressed with the evolution of kindled seizures. It remains unclear, however, whether axonal sprouting is a cause or effect of seizure activity or how and when it contributes to brain dysfunction and initiation of seizure activity. In this study, we used a coupled neural mass model to demonstrate that epileptic discharge activity can initiate from non-pathologic brain regions, reciprocally coupled to simulate an emergence of axonal sprouting. As axonal sprouting progresses and creates stronger connections between the brain regions, the discharge activity transitions into different types of seizure activity such as high frequency discharges, periodic oscillations, and low-amplitude high-frequency rhythms; increasing in beta-activity component (Fig. 1). These transitions can also be brought by an increase in post-synaptic gain, possibly concurrent with an increase in number of synaptic connections. Such increase in post-synaptic gain captures observed aberrant post-synaptic morphologies like the formation of multiple spine buttons similar to those observed with long term potentiation. The results delve into the possibility that axon sprouting maybe a primary mechanism, possibly concomitant with impaired inhibition, which can provide insights on how networks of brain regions are recruited and give rise to the generalization of seizure activity. In the future, we aim to construct a generative model for seizure activity initiation and propagation for diagnosis and treatment of patients with primary or secondary generalized epilepsy [2].

**Fig. 1:**
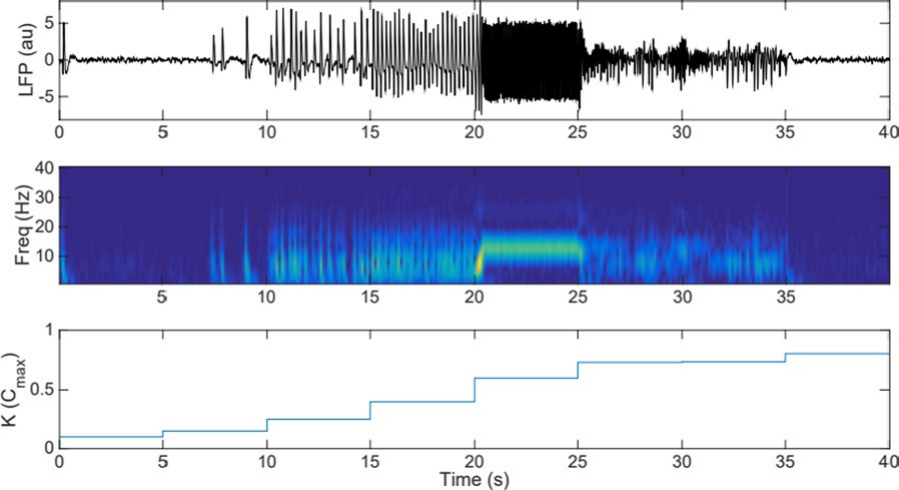
Seizure activity initiates as ictal discharges in a reciprocally coupled non-pathological neural masses. As coupling (axonal sprouting) increases, discharge activity increases in frequency and a transition to different types of seizure activity is observed such as low-amplitude high-frequency rhythms and waxing and waning. After further increase in coupling, baseline activity is recovered

**References**

1. Cavazos JE, Golarai G, Sutula TP. Mossy fiber synaptic reorganization induced by kindling: time course of development, progression, and permanence. *Journal of Neuroscience* 1991 Sep 1;11(9):2795–803.
2. Proix T, Bartolomei F, Guye M, Jirsa VK. Individual brain structure and modelling predict seizure propagation. *Brain* 2017. 140(3), 641–654.

## P135 Evaluation of signal processing of Golgi cells and Basket cells in vestibular ocular reflex motor learning using artificial cerebellum

### Taiga Matsuda, Keiichiro Inagaki

#### Chubu University, Kasugai, Japan

##### **Correspondence:** Taiga Matsuda (tr18016-4399@sti.chubu.ac.jp)

*BMC Neuroscience* 2019, **20(Suppl 1)**:P135

The vestibuloocular reflex (VOR) is one of the most popular model systems to study motor learning due to its clear function (stabilization of our vision) and ease in recording its input (head rotation) and output (eye movement) signals. The motor learning of the VOR requires the cerebellar flocculus. The flocculus receives sensory and motor information through mossy and climbing fibers, and outputs motor related activities to vestibular nuclei via Purkinje cell axons. Between these inputs and outputs lies a rich network of interneurons, most of them inhibitory (GABAergic). While most of the previous studies on VOR motor learning have focused on responses of Purkinje cells, little attention has paid to roles of cerebellar inhibitory interneurons due to a difficulty in identifying and recording those neurons in cerebellar cortex in behaving animals. Herein, we have constructed a computational model of the VOR that explicitly implements the anatomically realistic floccular neuronal network structure so that activities of each inhibitory interneuron can be evaluated. The model also allows us to knocked-out any specific interneuron(s) at any timing of VOR motor learning. The model consists of 20 Purkinje, 10000 granular, 900 Golgi, and 60 basket/stellate cells each of which is described as a conductance based spiking neuron model. These neuron models are connected, preserving convergence/divergence ratios between neuron types [1]. As bases of VOR motor learning, climbing fiber spike timing dependent LTD and LTP have been implemented at parallel fiber—Purkinje cell synapses. To induce VOR motor learning we simulated continuous application of visual-vestibular mismatch paradigm: VOR enhancement (VORe) and VOR suppression. In the VORe, the head and visual stimulus was applied out of phase, while those are given in phase in the VORs. Continuous application of VORe and VORs stimulus, VOR gain measured in darkness without visual stimulation increases or decreases, respectively. Furthermore, knock-out of Golgi and/or basket/stellate cells is simulated to investigate roles of those cells in the VOR motor learning.

We confirmed that the model reproduces adaptive changes of VOR gain with/without Golgi cells or basket/stellate cells. When the Golgi cell knocked-out in the motor learning, increase of VOR gain was slightly impaired while its decrease was enhanced. When the basket/stellate cell knocked-out, decrease of VOR gain was slightly impaired, while its increase was enhanced. Interestingly, retention of acquired VOR performance was affected for only low gain condition by elimination of Golgi cell or Basket/Stellate cell after the VOR motor learning. Those results indicated that the inhibitory interneurons play key roles in the high and low gain VOR motor learning, and retention of those memories.

**Acknowledgement:** A part of this work supported by JSPS KAKENHI Grant Number 17K12781 (KI)

**Reference**

1. Inagaki K, Hirata K. Computational theory underlying acute vestibulo-ocular reflex motor learning with cerebellar long-term depression and long-term potentiation. *The cerebellum* 2017, vol.16, pp.827–839.

## P136 Development of a self-motivated treadmill task that quantifies differences in learning behavior in mice for optogenetic studies of basal ganglia

### Po-Han Chen, Dieter Jaeger

#### Emory University, Department of Biology, Atlanta, GA, United States of America

##### **Correspondence:** Po-Han Chen (chenabe97@gmail.com)

*BMC Neuroscience* 2019, **20(Suppl 1)**:P136

The basal ganglia (BG) is involved in various cognitive functions, including stimulus-response associative learning and decision making. The two major pathways that connect the striatum and the output nuclei of the basal ganglia are direct and indirect, with the substantia nigra pars reticulata (SNr) / globus pallidus internus (GPi) inhibitory projections providing the final output. Signals through these pathways converge to inhibit the glutamatergic thalamic nuclei, which output onto the cortex. GPi activity suppresses inappropriate motor activity that may conflict with the movement being performed, making it an important integrator of learned reward-related behaviors [3]. Recent innovations in genetic technology resulted in the ability to stimulate distinct neural populations with light through the insertion of light sensitive ion channels. Optogenetic manipulations to the basal ganglia has shown to alter behavioral execution in mice, but what exactly are these behaviors that are being altered and to what degree? In our studies we have designed a self-paced treadmill task that will help better our understanding of how movement planning intersects with a self-motivational task. Ultimately, the goal is to use this task with optogenetic methods to activate GPi and determine how it reduces learned reward-seeking behaviors.

We designed an open field environment with a horizontal treadmill and a simple water delivery system. Mice are water-restricted to increase motivation. During training, once the mouse runs above a 200 cm distance threshold on the treadmill, an associated visual light cue signals that the water reward is ready. Water delivery is triggered by the breaking of an IR bream at the spout. To investigate the motivation aspect, we manipulate the reward sizes, which become a function of the run distance in a set period (15-60 s) and are delivered at the end of each period. Higher motivation is signaled by higher run speeds or run distances, as well as by between-trial response time. With this task, the effect of GPi activation on learned behavior and motivation can be elucidated with expression of Channelrhodopsin (ChR2) through AAV injection in GPi. A glass fiber will be implanted to allow for light stimulation of GPi. Optogenetic stimulation of GPi will be delivered at various intervals, such as right before cue delivery or during the behavioral response, to observe the effect of BG control has on learned behavior at different parts of execution. Because GPi provides inhibitory input to motor planning circuits in the cortex, we expect a reduction in learned behavior across all levels of baseline motivation. Future investigation of BG circuits and their movement effects can also use this task for examination into reward-seeking behavior and self-motivation.

**References**

1. Albin RL, Young AB, Penney JB. The functional anatomy of basal ganglia disorders. *Trends in Neurosciences* 1989; 12:366–375.
2. DeLong MR. Primate models of movement disorders of basal ganglia origin. *Trends in Neurosciences* 1990; 13:281–285.
3. Turner RS, Desmurget M. Basal Ganglia Contributions to Motor Control: A Vigorous Tutor. *Current Opinions Neurobiology* 2010; 20(6): 704–716.
4. Aravanis AM, Wang LP, Zhang F, et al. An optical neural interface: in vivo control of rodent motor cortex with integrated fibreoptic and optogenetic technology. *Journal of Neural Engineering* 2007; 4:143–156.
5. Sanders TH, Jaeger D. Optogenetic stimulation of cortico-subthalamic projections is sufficient to ameliorate bradykinesia in 6-ohda lesioned mice. *Neurobiology of Disease* 2016; 95:225–237.

## P137 Compensatory effects of dendritic retraction on excitability and induction of synaptic plasticity

### Martin Mittag^1^, Manfred Kaps^2^, Thomas Deller^3^, Hermann Cuntz^4^, Peter Jedlicka^5^

#### ^1^Justus Liebig University Giessen, Giessen, Germany; ^2^Justus Liebig University Giessen, Department of Neurology, Giessen, Germany; ^3^Goethe University Frankfurt, Institute of Clinical Neuroanatomy, Neuroscience Center, Frankfurt am Main, Germany; ^4^Frankfurt Institute for Advanced Studies (FIAS) & Ernst Strüngmann Institute (ESI), Computational Neuroanatomy, Frankfurt/Main, Germany; ^5^Justus Liebig University, Faculty of Medicine, Giessen, Germany

##### **Correspondence:** Martin Mittag (martin.mittag@gmx.de)

*BMC Neuroscience* 2019, **20(Suppl 1)**:P137

How can a neuron maintain its function under changed physiological or pathological conditions? Brain lesions affect not only the locally damaged area but have an impact also on postsynaptic regions. Lesion-induced denervation of connections from the entorhinal cortex causes significant loss of synapses in the hippocampal dentate gyrus. Subsequently, dendritic retraction occurs in the postsynaptic target area containing hippocampal dentate granule cells. Our previous models showed that dendritic retraction is capable of increasing the excitability of neurons thus compensating for the denervation-evoked loss of synapses. The firing rate remains similar in healthy and denervated neurons despite weaker synaptic input upon denervation (firing rate homeostasis). However, this effect was computed only for stochastically stimulated AMPA synapses [1] but not for more realistic AMPA/NMDA synapses. Furthermore, a boost in backpropagating action potentials (bAPs) in denervated granule cells might affect homeostasis of synaptic plasticity. Therefore, here we investigated the consequences of dendritic retraction for (1) firing rate homeostasis and (2) NMDA receptor-dependentsynaptic plasticity in biologically realistic compartmental models driven by AMPA/NMDA synapses. Our simulations predict that dendritic retraction supports firing rate homeostasis and partially also synaptic plasticity homeostasis.

**Acknowledgement:** The work was supported by BMBF (No. 01GQ1406 – Bernstein Award 2013 to H.C.), University Medical Center Giessen and Marburg (UKGM; to P.J. and M.K.), LOEWE CePTER – Center for Personalized Translational Epilepsy Research (to P.J. and T.D.)

**Reference**

1. Platschek S, Cuntz H, Vuksic M, Deller T, Jedlicka P. A general homeostatic principle following lesion induced dendritic remodeling, *Acta Neuropathologica Communications* 2016

## P138 Lognormal distribution of spine sizes is preserved following homo- and heterosynaptic plasticity in the dentate gyrus

### Nina Rößler^1^, Tassilo Jungenitz^2^, Stephan Schwarzacher^2^, Peter Jedlicka^3^

#### ^1^Goethe University Frankfurt, Frankfurt, Germany; ^2^Goethe University Frankfurt, Institute of Clinical Neuroanatomy, Frankfurt, Germany; ^3^Justus Liebig University, Faculty of Medicine, Giessen, Germany

##### **Correspondence:** Nina Rößler (nina.roessler@gmx.net)

*BMC Neuroscience* 2019, **20(Suppl 1)**:P138

The dentate gyrus is one of two brain regions that exhibit adult neurogenesis. It was shown to be important for hippocampal learning and memory processes, which are based on synaptic plasticity. We have recently reported structural homo- and heterosynaptic long-term synaptic plasticity emerging in adult-born dentate granule cells at a cell age of 35 days [1]. High frequency stimulation of the medial perforant path in this and later stages of adult neurogenesis led to spine enlargement in stimulated dendritic regions (homosynaptic structural LTP) and a concurrent spine shrinkage in neighboring non stimulated dendritic segments (heterosynaptic structural LTD).

Here we perform a follow-up systematic analysis of spine plasticity data. Our results show that spine sizes follow a lognormal distribution, both in dendritic segments undergoing homosynaptic spine enlargement as well as heterosynaptic spine shrinkage, suggesting that the overall distribution of spine sizes does not change. We are currently developing computational models, which should account for the observed spine changes in adult-born granule cells and provide new insights into plasticity rules in the dentate gyrus.

**Reference**

1. Jungenitz T, et al. Structural homo- and heterosynaptic plasticity in mature and adult newborn rat hippocampal granule cells. *PNAS* 2018, 115(20):e4670e–4679.

## P139 Inferring the dynamic of personalized large-scale brain network models using Bayesian framework

### Meysam Hashemi^1^, Anirudh Vattikonda^1^, Viktor Sip^1^, Maxime Guye^2^, Marmaduke Woodman^1^, Viktor Jirsa^1^

#### ^1^Aix-Marseille Université, Institut de Neurosciences des Systèmes, Marseille, France; ^2^Aix-Marseille Université, Institut de Neurosciences de la Timone, Marseille, France

##### **Correspondence:** Meysam Hashemi (meysam.hashemi@univ-amu.fr)

*BMC Neuroscience* 2019, **20(Suppl 1)**:P139

Despite the importance and common use of Bayesian inference in brain network modelling to understand how experimental modalities result from the dynamics of coupled neural populations, many challenges remain to be addressed in this context. The recent successful personalized strategies towards epilepsy treatment [1] motivated us to focus on Bayesian parameter estimation of Virtual Epileptic Patient (VEP) brain model. The VEP is based on personalized brain network models derived from non-invasive structural data of individual patients. Using VEP as generative model, and the recently developed Bayesian algorithms implemented in probabilistic programming languages [2], our aim is to infer the dynamics of brain network model from the patient’s empirical data. We estimate the spatial dependence of excitability and provide a heat map capturing an estimate of epileptogenicity and our confidence thereof. The Bayesian framework taken in this work proposes an appropriate patient-specific strategy to infer epileptogenicity of the brain regions to improve outcomes after epilepsy surgery.

**References**

1. Jirsa VK, Proix T, Perdikis D, et al. The virtual epileptic patient: individualized whole-brain models of epilepsy spread. *Neuroimage* 2017 Jan 15;145:377–88.
2. The Stan Development Team. Stan: A C ++ Library for Probability and Sampling, 2015.

## P140 Personalized brain network model for deep brain stimulation on treatment-resistant depression: Spatiotemporal network organization by stimulation

### Sora An^1^, Jan Fousek^1^, Vineet Tiruvadi^2^, Filomeno Cortese^3^, Gwen van der Wijk^4^, Laina McAusland^5^, Rajamannar Ramasubbu^6^, Zelma Kiss^7^, Andrea Protzner^8^, Viktor Jirsa^1^

#### ^1^Aix Marseille Universite, Institute de Neurosciences, Marseille, France; ^2^Emory University School of Medicine, Department of Psychiatry and Behavioral Sciences, Atlanta, Georgia, United States of America; ^3^University of Calgary, Seaman Family MR Centre, Foothills Medical Centre, Hotchkiss Brain Institute, Calgary, Alberta, Canada; ^4^University of Calgary, Department of Psychology, Calgary, Alberta, Canada; ^5^University of Calgary, Department of Clinical Neurosciences, Calgary, Alberta, Canada; ^6^University of Calgary, Hotchkiss Brain Institute, Cumming School of Medicine, Calgary, Alberta, Canada; ^7^University of Calgary, Hotchkiss Brain Institute, Department of Clinical Neurosciences, Calgary, Alberta, Canada; ^8^University of Calgary, Hotchkiss Brain Institute, Department of Psychology, Calgary, Alberta, Canada

##### **Correspondence:** Sora An (an.sora@univ-amu.fr)

*BMC Neuroscience* 2019, **20(Suppl 1)**:P140

Deep brain stimulation (DBS) is a surgical technology in which fine electrodes are implanted into the brain and connected to a type of pacemaker. This applies chronic high frequency electrical stimulation to the brain 24 hours a day for years. It has revolutionized the treatment of movement disorders, such as Parkinson disease, and is being studied as potential treatment for several other disorders, including treatment resistant depression (TRD). In TRD, the subcallosal cingulate gyrus (SCG) is most commonly used as a DBS target because it shows hyperactivity in patients with depression, normalization of activity in the context of positive response to other antidepressant treatments, and because the SCG has structural connections with several key regions involved in mood regulation.DBS treatment outcome has been variable, with some studies failing to find effects, and others finding positive outcomes in up to 80% of patients. Potential reasons for these inconsistent findings are that the ideal stimulation target location and ideal stimulation parameters are currently unknown. DBS for TRD is therefore still applied on a trial-and-error basis, which, especially considering the invasive nature of this treatment, is far from ideal. Determining the exact stimulation conditions that generate good treatment outcome is thus crucial for applying DBS to TRD.

In this study, we propose a computational modeling approach for identifying the ideal stimulation location. Toward this end, we have built personalized brain network models based on neuroimaging data obtained from each patient, using The Virtual Brain (TVB) platform. Then, spatiotemporal brain activation patterns following the stimulation are simulated. In the simulations, electrical stimulation is systematically applied to each electrode contact (8 contacts per patient), and the fiber tracts activated in each case are determined from the voltage distribution across each fiber tract. The voltage distribution is calculated based on patient-specific contact positions and anatomical locations of fiber tracts, by employing the finite difference method. Source activity from each brain node is projected to 65-channel electroencephalography (EEG) sensor space, through the forward solution. In order to verify the validity of the proposed model, the simulated EEG signals are compared with empirical data, i.e., the event-related potentials recorded by means of EEG from the individual patient. The results show that brain network models based on fiber tract activation are able to reproduce the spatiotemporal response patterns according to the stimulation location, which can be useful to optimize the active contact positions in individual patients. This study sets the stage forapplying computational modeling in the context of personalized medicine, where an in-silico brain platform allows clinicians to test andoptimize DBS strategies for individual patients, prior to implantation.

**Acknowledgements**: We wish to acknowledge the financial support of the following agencies; Fondation pour la Recherche Médicale (FRM) (grant number DIC20161236442), European Commission’s Human Brain Project (grant agreement H2020-720270), the SATT Sud-Est (TVB-Epilepsy) to VJ; Alberta Innovates Health Solutions (previously, Alberta Heritage Foundation for Medical Research) to ZK and RR; Natural Sciences and Engineering Council of Canada (NSERC; grant number 418454-2013) to ABP.

## P141 Transmission time delays organize the brain network synchronization dynamics

### Spase Petkoski, Viktor Jirsa

#### Aix-Marseille University, Institut de Neurosciences des Systèmes, Marseille, France

##### **Correspondence:** Spase Petkoski (spase.petkoski@univ-amu.fr)

*BMC Neuroscience* 2019, **20(Suppl 1)**:P141

Timings of the activity at brain regions, which can be described by their phases for oscillatory processes, are of crucial importance for the brain functioning. The structure of the brain constrains its dynamics through the delays due to propagation and the strengths of the white matter tracts [1]. Rhythms and their synchronization, as one of the key mechanisms of brain function [2] are particularly sensitive to delays, which become notably long in large-scale brain models with biologically realistic connectivity [3].

We show theoretical and in-silico numerical results for phase coherence between signals from different brain regions. For this we build on the Kuramoto model with spatially distributed time delays [4], where the network connectivity strengths and distances are defined by the connectome. Phase relations and their regions of stability are derived and numerically confirmed, showing that besides in-phase, clustered delays can induce anti-phase synchronization for certain frequencies, while the sign of the lags is determined by the inhomogeneous network interactions [5]. For in-phase synchronization faster oscillators always phase lead, while stronger connected nodes lag behind the weaker during frequency depression, which consistently arises for in-silico results (See Fig. 1). The statistics of the phases is calculated from the phase locking values, as in many empirical studies, and we scrutinize the method’s impact. The choice of surrogates does not affect the mean of the observed phase lags, but higher significance levels that are generated by some surrogates, cause decreased variance and might fail to detect the generally weaker coherence of the interhemispheric links. These links are also affected by the non-stationary and intermittent synchronization, which causes multimodal phase lags that can be misleading if averaged [5].

**Fig. 1:**
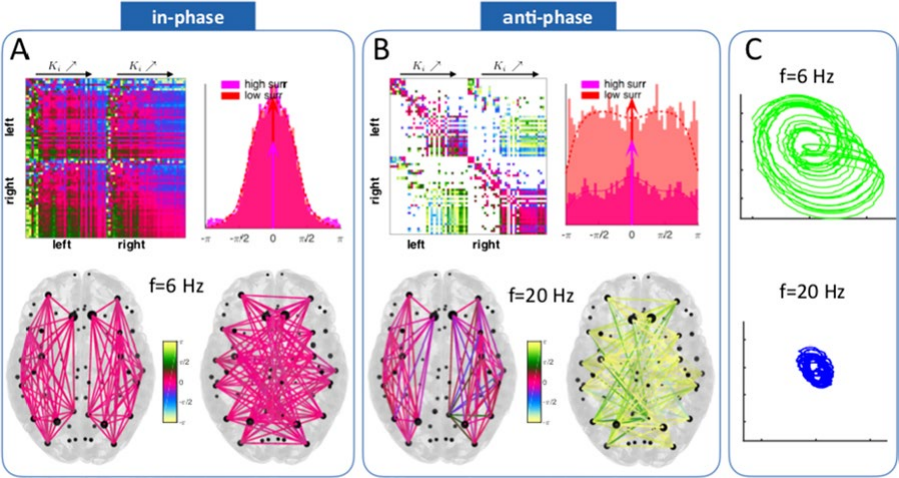
**a** In- and **b** anti-phase interhemispheric synchronization for different frequencies. Matrices show phase lags between brain regions, ordered by in-strength within each hemisphere, and upper right are histograms of phase lags for the whole brain. (bottom) Intra- and inter-hemispheric lags for links between 10 strongest regions. **c** Amplitude reduction of the neural activity due to delays

The architecture of the phase lags are confirmed for non-isochronous, nonlinearly damped, and chaotic oscillators, which show a robust switching from in to anti-phase synchronization by increasing the frequency, with a consistent lagging of the stronger connected regions [6]. Increased frequency and coupling are also shown to distort the oscillators by decreasing their amplitude, and stronger regions have lower, but more synchronized activity [6]. Taken together, the results indicate specific features in the phase relationships within the brain that need to hold for a wide range of local oscillatory dynamics, given that the time-delays of the connectome are proportional to the lengths of the structural pathways [5, 6].

**References**

1. Sanz-Leon P et al. Mathematical framework for large-scale brain network modeling in the virtual brain. *Neuroimage* 2015, 111, 385–430
2. Varela F, Lachaux J, Rodriguez E, Martinerie J. The brainweb: Phase synchronization and large-scale integration. *Nature Review Neuroscience* 2001, 2(4): 229–239
3. Deco G, Jirsa V, McIntosh AR, Sporns O, Kötter R. Key role of coupling, delay, and noise in resting brain fluctuations. *PNAS USA* 2009, 106 (25): 10302–10307
4. Petkoski S, et al. Heterogeneity of time delays determines synchronization of coupled oscillators. *Physical Review E* 2016, 94, 012209
5. Petkoski S, et al. Phase-lags in large scale brain synchronization: Methodological considerations and in-silico analysis. *PLoS Computational Biology* 2018, 14(7), 1–30.
6. Petkoski S, et al. Transmission time delays organize the brain network synchronization dynamics. *Philosophical Transactions of the Royal Society A* [in review]

## P142 Mutual information vs. transfer entropy in spike-based neuroscience

### Mireille Conrad, Renaud Jolivet

#### University of Geneva, Nuclear and Corpuscular Physics Department, Genève, Switzerland

##### **Correspondence:** Mireille Conrad (mireille.conrad@unige.ch)

*BMC Neuroscience* 2019, **20(Suppl 1)**:P142

Energetic constraints might limit and shape information processing in the brain, and it has been shown previously that synapses maximize energy efficiency of information transfer rather than information transfer itself. To investigate computation by neural systems, measuring the amount of information transferred between stimuli and neural responses is essential. Information theory offers a range of tools to calculate information flow in neural networks. Choosing the appropriate method is particularly important in experimental contexts, where technical limitations can complicate or limit the use of information theory.

Here, we will discuss the comparative advantages of two different metrics: mutual information and transfer entropy. We will compare their performance on biologically plausible spike trains and discuss their accuracy depending on various parameters, and on the amount of available data, a critical limiting factor in all practical applications of information theory. We will first demonstrate these metrics’ performances using synthetic random spike trains before moving on to more realistic spike-generating models. Those realistic models focus on the generation of input spike trains with a statistical structure similar to biological spike trains, and also on the generation of output spike trains with an experimentally-calibrated Hodgkin-Huxley-type model. We will conclude by discussing how these metrics can be used to study brain function, especially the effect of neuromodulators and learning rules as ways for synapses to maximize energy efficiency.

## P143 Plasticity rules for learning sequential inputs under energetic constraints

### Dmytro Grytskyy^1^, Renaud Jolivet^1,2^

#### ^1^University of Geneva, Geneva, Switzerland; ^2^CERN, DPNC, Genève, Switzerland

##### **Correspondence:** Dmytro Grytskyy (dmytro.grytskyy@etu.unige.ch)

*BMC Neuroscience* 2019, **20(Suppl 1)**:P143

Information measures are often used to assess the efficacy of neural networks and learning rules can be derived through optimization procedures on such measures [3, 8, 10]. There has also been recent interest for sequence learning for specific tasks [2], or with specific network configurations [7]. In biological neural networks, computation is restricted by the amount of available resources [4, 11]. Considering energy restrictions, it is reasonable to balance information processing efficacy with energy consumption [1]. Here, we obtain such an energy-constrained learning rule for inputs described as sequence of events.

We studied networks of non-linear Hawkes neurons and assessed information flow using mutual information. We then applied gradient descent for a combination of mutual information and energetic costs to obtain a learning rule. The rule obtained contains a sliding threshold similar to the Bienenstock-Cooper-Munro rule [5]. It contains terms local in time and in space, plus one global variable common to the whole network. The rule thus belongs to so-called three-factors rules, and the global variable could be related to neuromodulation [6]. Because that global variable integrates over time, consecutive inputs can influence synaptic changes triggered by preceding events. We additionally investigated the relation between that rule and STDP, and obtained different STDP-like learning windows for excitatory and inhibitory neurons.

Constraining energy consumption results in a rearrangement of the correspondence between inputs and respective outputs, with more frequent input patterns mapped to lower energy orbits. Taking into account unreliability of neural transmission results in an additional negative term in the learning rule proportional to the synaptic weight. This has the effect that extremely rare events aren’t learned, while moderately rare inputs evoke maximal network activity.

When different neurons respond to different inputs that are predictive of each other, synaptic weights between these neurons will be reinforced. Different inputs regularly occurring in close temporal relation to each other can be defined as a context, which can lead to the appearance of subnetworks coding for the whole context rather than for components of it, lowering energetic costs of that representation. For almost strict sequences, neurons representing late inputs in the sequence might be inhibited, reducing energy costs.

**Acknowledgements**: Supported by the Swiss National Science Foundation (31003A_170079) and by the Australian Research Council (DP180101494).

**References**

1. Bourdoukan R, Barrett D, Deneve S, Machens CK. Learning optimal spike-based representations*. In Advances in neural information processing systems* 2012 (pp. 2285–2293).
2. Brea J, Senn W, Pfister JP. Sequence learning with hidden units in spiking neural networks. *In Advances in neural information processing systems* 2011 (pp. 1422–1430).
3. Chechik G. Spike-timing-dependent plasticity and relevant mutual information maximization. *Neural Computation* 2003 Jul 1;15(7):1481–510.
4. Harris JJ, Jolivet R, Attwell D. Synaptic energy use and supply. Neuron. 2012 Sep 6;75(5):762–77.
5. Intrator N, Cooper LN. Objective function formulation of the BCM theory of visual cortical plasticity: Statistical connections, stability conditions. *Neural Networks* 1992 Jan 1;5(1):3–17.
6. Isomura T, Toyoizumi T. A local learning rule for independent component analysis. *Scientific reports* 2016 Jun 21;6:28073.
7. Kappel D, Nessler B, Maass W. STDP installs in winner-take-all circuits an online approximation to hidden Markov model learning. *PLoS Computational Biology* 2014 Mar 27;10(3):e1003511.
8. Linsker R. Local synaptic learning rules suffice to maximize mutual information in a linear network. *Neural Computation* 1992 Sep;4(5):691–702.
9. Toyoizumi T, Pfister JP, Aihara K, Gerstner W. Spike-timing dependent plasticity and mutual information maximization for a spiking neuron model. *In Advances in neural information processing systems* 2005 (pp. 1409–1416).
10. Yu L, Yu Y. Energy‐efficient neural information processing in individual neurons and neuronal networks. *Journal of Neuroscience Research* 2017 Nov;95(11):2253–66.

## P144 Hierarchical inference interactions in dynamic environments

### Zachary Kilpatrick^1^, Tahra Eissa^1^, Nicholas Barendregt^1^, Joshua Gold^2^, Kresimir Josic^3^

#### ^1^University of Colorado Boulder, Applied Mathematics, Boulder, Colorado, United States of America; ^2^University of Pennsylvania, Neuroscience, Philadelphia, Pennsylvania, United States of America; ^3^University of Houston, Mathematics, Houston, United States of America

##### **Correspondence:** Zachary Kilpatrick (zpkilpat@colorado.edu)

*BMC Neuroscience* 2019, **20(Suppl 1)**:P144

In a constantly changing world, accurate decisions require flexible evidence accumulation. As old information becomes less relevant, it should be discounted at a rate adapted to the frequency of environmental changes. However, sometimes humans and other animals must simultaneously infer the state of the environment and its volatility (hazard rate). How do such inference processes interact when performed hierarchically? To address this question, we developed and analyzed a model of an ideal observer who must report either the state or the hazard rate. We find that the speed of both state and hazard rate inference is mostly determined by information integration across change points.

Our observer infers the state and hazard rate by integrating noisy observations and discounting them according to an evolving hazard rate estimate. To analyze this model and its variants, we developed a new method for computing the observer’s state and hazard rate beliefs. Instead of sampling, we solve a set of nonlinear partial differential equations (PDEs), leading to faster and more accurate estimates. We characterize how optimal and suboptimal (those with mistuned evidence discounting rates or other discounting functions) observers infer the state and hazard rate and compare their performance in tasks with varying difficulty. Suboptimal observers may possess mistuned evidence discounting rates or even different functional forms of discounting.

Evidence near change points strongly perturbs an observer’s posterior by altering the state belief and supports higher hazard rates. Thus, state and hazard rate inference are linked, and the speed of hazard rate learning is primarily determined by how well the observer accounts for change points. Early in a trial, changes may not be well tracked, as the observer’s hazard rate estimate is poor, but this estimate improves as the trial evolves, and environmental changes are better tracked.

We measure how biases in hazard rate learning influence an observer’s state inference process. Our setup can therefore be used to improve dynamic decision task design by identifying parameterizations that reveal hierarchical inference strategies.

**Acknowledgements**: We thank a grant from NIH in Collaborative Research in Computational Neuroscience for supporting Zachary Kilpatrick, Tahra Eissa, and Nicholas Barendregt with R01MH115557-01.

## P145 Optimizing sequential decisions in the drift-diffusion model

### Khanh Nguyen^1^, Zachary Kilpatrick^2^, Kresimir Josic^1^

#### ^1^University of Houston, Mathematics, Houston, TX, United States of America; ^2^University of Colorado Boulder, Applied Mathematics, Boulder, CO, United States of America

##### **Correspondence:** Khanh Nguyen (kpnguyen@math.uh.edu)

*BMC Neuroscience* 2019, **20(Suppl 1)**:P145

Natural environments change over many different timescales. To make the best decisions organisms must therefore flexibly accumulate information, accounting for what is relevant, and ignoring what is not. However, many experimental and modeling studies of decision-making focus on sequences of independent trials. In such studies, both the evidence gathered to make a choice and the resulting actions are irrelevant to future decisions. To understand decision-making under more natural conditions, we propose and analyze models of observers who accumulate evidence to freely make choices across a sequence of correlated trials, and receive uncertain feedback.

Two alternative forced choice tasks are often used to identify strategies humans and other animals use to make decisions. Experiments have shown that subjects can learn the latent probabilistic structure of the environment to increase their performance. However, a lack of systematic analyses of normative models makes it difficult to study whether and how subjects’ decision-making strategies deviate from optimality. To address this problem, we extend drift-diffusion models to obtain the normative form of evidence accumulation in serial trials whose correct choice evolves as a two-state Markov process. Ideal observers integrate noisy evidence within a trial until reaching a decision threshold. Their initial belief is biased by their choice and feedback on previous trials. If observers use fixed decision thresholds, their bias decreases decision times, but leaves the probability of correct answers unchanged. To optimize reward rate in trial sequences, ideal observers adjust their thresholds over trials to deliberate longer on early decisions, and respond more quickly later in the sequence. We show how conflicts between unreliable feedback and evidence from previous trials are resolved by marginalization. Our findings are consistent with experimentally observed response trends, suggesting humans often assume correlations in task environments even when none exist.

## P146 Degeneracy in hippocampal CA1 neurons

### Rosanna Migliore^1^, Carmen Alina Lupascu^1^, Luca Leonardo Bologna^1^, Armando Romani^2^, Jean-Denis Courcol^2^, Werner Alfons Hilda Van Geit^2^, Alex M Thomson^3^, Audrey Mercer^3^, Sigrun Lange^3^, Christian A Rössert^2^, Ying Shi^2^, Olivier Hagens^2^, Maurizio Pezzoli^2^, Tamas Freund^4^, Eilif Muller^2^, Felix Schuermann^2^, Henry Markram^2^, Michele Migliore^1^, Stefano Antonel^2^, Joanne Falck^3^, Szabolcs Kali^4^

#### ^1^Institute of Biophysics, National Research Council, Italy; ^2^École Polytechnique Fédérale de Lausanne, Blue Brain Project, Lausanne, Switzerland; ^3^University College London, London, United Kingdom; ^4^Institute of Experimental Medicine, Hungarian Academy of Sciences, Hungary

##### **Correspondence:** Rosanna Migliore (rosanna.migliore@cnr.it)

*BMC Neuroscience* 2019, **20(Suppl 1)**:P146

Every neuron of a network exerts its function by transforming multiple spatiotemporal synaptic input patterns into a single spiking output. During development and during the entire lifetime of a neuron, its input/output function is adapted to realize ongoing refinement of the function of the neuron and circuit, or maintain functional robustness in the face of constant protein turnover or an evolving pathological condition. This process results in a high variability in the observed peak conductance of ion channels across neurons. The mechanisms responsible for this variability are not well understood, although there are clear experimental and modeling indications that correlation and degeneracy among a variety of conductances can be involved.

Here, using a unified data-driven simulation workflow [1, 2], we studied this issue in -detailed models of hippocampal CA1 pyramidal cells and interneurons with morphological and electrophysiological properties explicitly constrained with experimental data from rats [3].

The models and their analysis show that the set of conductances expressed in any given hippocampal neuron may be considered as belonging to two groups: one subset is responsible for the major characteristics of the firing behavior in each population and the other more involved in degeneracy. It is also possible to conceive several experimentally testable predictions related to the combination and relative proportion of the different conductances that should be expressed on the membrane of different types of neurons for them to fulfill their role in the hippocampus circuitry.

**References**

1. This modeling effort has been carried out using the Brain Simulation Platform (https://collab.humanbrainproject.eu/#/collab/1655/nav/28538) and two open-source packages, the Electrophys Feature Extraction Library (eFEL, https://github.com/BlueBrain/eFEL) and the Blue Brain Python Optimization Library (BluePyOpt) developed within the Human Brain Project (https://www.humanbrainproject.eu/en/).
2. Van Geit W, Gevaert M, Chindemi G, et al. BluePyOpt: Leveraging Open Source Software and Cloud Infrastructure to Optimise Model Parameters in Neuroscience. *Frontiers in Neuroinformatics* 2016, 10: 17. 10.3389/fninf.2016.00017
3. Migliore R, Lupascu CA, Bologna LL, et al. The physiological variability of channel density in hippocampal CA1 pyramidal cells and interneurons explored using a unified data-driven modeling workflow. *PLoS Computational Biology* 2018. 14(9): e1006423. 10.1371/journal.pcbi.1006423

## P147 Mechanisms of combined electrical and optogenetic costimulation

### William Hart^1^, Paul Stoddart^1^, Tatiana Kameneva^2^

#### ^1^Swinburne University of Technology, ARC Centre for Biodevices, Melbourne, Australia; ^2^Swinburne University of Technology, Telecommunication Electrical Robotics and Biomedical Engineering, Hawthorn, Australia

##### **Correspondence:** Tatiana Kameneva (tkameneva@swin.edu.au)

*BMC Neuroscience* 2019, **20(Suppl 1)**:P147

Neuroprosthetic devices are reaching a level of maturity and have benefited many people who suffer from neurological conditions such as deafness and blindness. However, the perception outcome that they provide is significantly less than normal function. In part, this is due to the current spread, neural adaptation and inability to selectively activate different classes of neurons when using electrical stimulation. Optogenetic neural stimulation may provide an alternative to conventional electrical pulse stimulation by delivering more targeted stimulation with higher spatial resolution. A novel way to stimulate neurons is to combine conventional electrical stimulation with targeted optogenetic stimulation. The mechanisms of neural activation in response to the combined electrical and optogenetic costimulation are not clear.

To investigate the mechanisms of neural activation in response to electrical and optogenetic costimulation, we used computer simulations in the NEURON environment. We simulated single compartment neurons and used Hodgkin-Huxley type formalism to study how costimulation and a combination of ionic channels affect the neuronal response. To simulate an optogenetically modified neuron, we combined voltage-activated currents with a model of channelrodopsin-2 ion channel responsive to voltage, temperature and light. We systematically applied different levels of intracellular current pulse stimulation and optical stimulation to bring the membrane potential close to firing threshold. We also applied mock-electrical current stimulation that approximates the response of neurons to optical-alone stimulation and studied the activation of ionic channels in this case. To isolate the mechanisms during costimulation, the maximum sodium conductance in the NEURON model was set to zero, simulating total blockage of sodium channels.

Our results showed that the membrane is initially depolarised by a small inward channelrhodopsin current during the optical stimulation, followed by a rapid sodium current following the electrical trigger. During costimulation, the channelrhodopsin current transiently reduced during the action potential due to its voltage sensitivity. This result matched modelling and experimental data reported by [1] in cardiomyocytes.

Our results support the interpretation of a costimulation mechanism involving two separate families of ion channels. Our results may have implications for the development of stimulation strategies in novel neurosprosthetic devices that have electrical and optogenetic stimulation capabilities.

**Reference**

1. Williams JC, Xu J, Lu Z, et al. Computational optogenetics: empirically-derived voltage-and light-sensitive channelrhodopsin-2 model. *PLoS computational biology* 2013 Sep 12;9(9):e1003220.

## P148 Real-time Bayesian decoding of taste from neural populations in gustatory cortex

### Daniel Svedberg, Bradly Stone, Donald Katz

#### Brandeis University, Department of Neuroscience, Waltham, MA, United States of America

##### **Correspondence:** Daniel Svedberg (dsvedberg@brandeis.edu)

*BMC Neuroscience* 2019, **20(Suppl 1)**:P148

The activity of neural ensembles in gustatory cortex encodes various features of gustatory stimuli in a temporally dynamic fashion, using adaptive coding schemes. Although it is well-established that electrophysiological activity of neural ensembles in gustatory cortex differentiates the identities of basic tastes over many taste exposures, it is unknown if taste can be statistically and reliably identified from individual trials, on an instantaneous basis. Rats were implanted with a multielectrode drive in gustatory cortex and were given oral deliveries of liquids with one of each of the basic tastes. Here we demonstrate that a naïve Bayesian decoder can reliably decode tastes from populations of neurons on an instantaneous basis, evaluate various strategies for establishing sampling periods, and compare dynamics of Bayesian decoding against dynamic state transitions identified by Hidden Markov Modeling.

## P149 Effects of value on early sensory activity and motor preparation during rapid sensorimotor decisions

### L. Alexandra Martinez-Rodriguez, Simon P. Kelly

#### University College Dublin, School of Electrical & Electronic Engineering, Dublin, Ireland

##### **Correspondence:** L. Alexandra Martinez-Rodriguez (l.martinezrodriguez@ucdconnect.ie)

*BMC Neuroscience* 2019, **20(Suppl 1)**:P149

Various computational accounts have been proposed to explain how sensorimotor decisions are biased by value. Although the longstanding dominant account has been the Starting Point Bias model, where the starting point of an evidence-accumulating decision variable is shifted towards the higher value decision bound, our group recently showed that fast biased decisions are best explained by a Drift Rate Bias model when one allows for temporally increasing drift rate [1] Such drift rate biases may arise directly from the modulation of the sensory representations of evidence in low-level sensory cortex. Alternatively, they may be implemented through post-sensory modulations, in which case they would be expressed in motor preparation dynamics but not in sensory encoding. Our study examines this by recording EEG (Biosemi), eye-position (Eyelink) and EMG of the flexor policis brevis muscle during a value-biased orientation discrimination task under a strict deadline, where a correct response to one orientation was worth more (40 points) than the other (10).

As expected, errors were more frequent (p<.001) and were committed with greater haste (p<.01) on low value trials such that the fastest responses were purely value driven and the slowest entirely sensory-driven (hence correct). Replicating Afacan et al, a Dynamic Drift Rate Bias model, in which drift rate is biased by value and is non-stationary, increasing over the short decision time frame, fit the behavioural data better than models with stationary evidence and/or starting point bias.

Neurophysiological analyses revealed that the initial “C1” component of the visual evoked potential (VEP), thought to reflect primary visual cortical activation, showed no signs of significant value modulation (p>.1) for either correct responses or for errors. A Starting Point bias mechanism around target onset was observed in the Lateralized Readiness Potential (LRP), across the different value conditions. Interestingly, this starting point bias seemed to increase with time (Fig. 1).

**Fig. 1:**
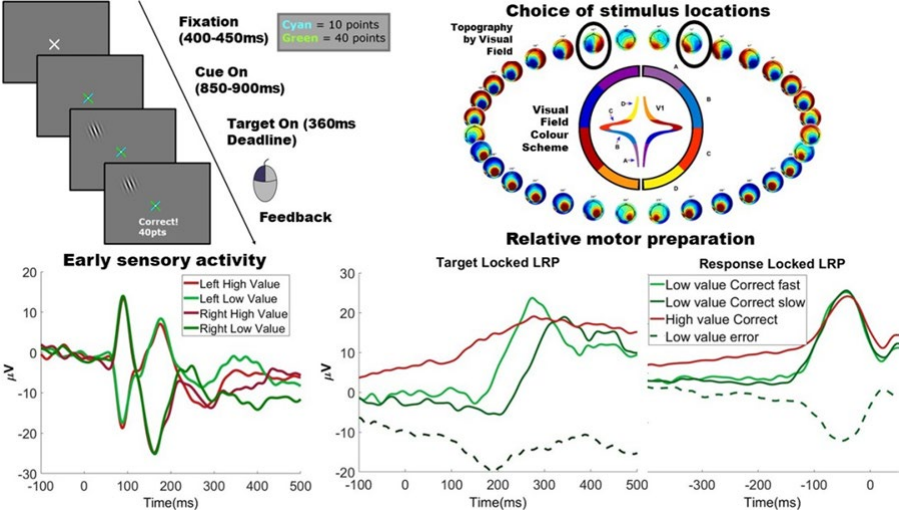
Orientation discrimination task, average retinotopic organization of the V1 region and neurophysiological results

Given that the best fitting model included a drift rate bias but our results showed that value does not modulate the earliest sensory evidence, this bias must be implemented at a later processing stage. It could be that these biases are implemented at the motor level, taking the form of urgency signals. The slopes of the urgency signals and their onsets could vary according to the value of the stimulus. In order to test this hypothesis, a new model was created which included an urgency signal with a different start time and rate of increase for each value condition. This Dynamic Urgency model proved to fit the data as well as the Dynamic Drift Rate Bias model did. Further, these urgency signals can account for an initially negative drift rate as well as the temporally growing Starting Point Bias observed in the Lateralized Readiness Potential. These findings further demonstrate the inadequacy of standard stationary-accumulation models in explaining highly time-constrained, value-biased decisions, and highlight novel computational architectures that may explain the more complex decision formation dynamics unfolding in such scenarios, which are prevalent in real life.

**Reference**

1. Afacan-Seref K, Steinemann NA, Blangero A, Kelly SP. Dynamic interplay of value and sensory information in high-speed decision making. *Current Biology* 2018 Mar 5;28(5):795–802.

## P150 A real-time model fitting method for single individual neurons

### Felix B. Kern^1^, Thomas Nowotny^2^, George Kemenes^1^

#### ^1^University of Sussex, School of Life Sciences, Brighton, United Kingdom; ^2^University of Sussex, School of Engineering and Informatics, Brighton, United Kingdom

##### **Correspondence:** Felix B. Kern (fbk21@sussex.ac.uk)

*BMC Neuroscience* 2019, **20(Suppl 1)**:P150

Most currently available methods of constructing conductance-based biophysical models of neuronal activity are not suitable for finding valid descriptions of live individual neurons. Often, data from several neurons of a given type, e.g. pharmacologically separated currents, are pooled to yield a single model, ignoring the cells’ unique morphology and, as has been shown to be the case in at least some invertebrate neurons, individual differences in current expression. Alternatively, electrophysiological data from a single neuron are combined with morphological reconstruction of that same neuron to yield a highly accurate model of a single cell at the price of a process spanning many days or weeks. In neither case does the resulting model match a living, experimentally accessible neuron.

In order to probe e.g. the dynamics of small neuronal circuits, or to track changes in neuronal properties over time induced e.g. by intrinsic plasticity, cellular homoeostasis, or extrinsic modulation of ion channels, it would be desirable to have an accurate model of the cell under investigation while the experiment is ongoing. Here, we present a novel method that attempts to solve this problem. Rather than separating currents pharmacologically, we use a machine learning approach to design highly informative voltage clamp stimulation protocols in advance of an experiment, guided by an informed guess about how the model might be structured and parametrised. Using these stimulations and a massively parallel optimisation algorithm, we rapidly arrive at model parameters that accurately reflect the properties of the membrane and its active conductances. We show a proof of concept for single-compartment models in simulation and in a model system and discuss advantages and limitations of the method.

## P151 S1 neurons process spontaneous pain information using nonlinear distributed coding

### Sa-Yoon Park^1^, Heera Yoon^2^, Sun Kwang Kim^2^, Sang Jeong Kim^3^, Chang-Eop Kim^4^

#### ^1^Gachon University, Department of Physiology, Seongnam, South Korea; ^2^Kyung Hee University, Department of Physiology, Seoul, South Korea; ^3^Seoul National University, Department of Physiology, Seoul, South Korea; ^4^Gachon Uniersity, Department of Physiology, Seoul, South Korea

##### **Correspondence:** Sa-Yoon Park (sayou92@naver.com)

*BMC Neuroscience* 2019, **20(Suppl 1)**:P151

The coding principle of pain has been debated between two opposing ideas for decades—labeled line coding and distributed coding. Further, according to the way of distribution, distributed coding can be categorized as linear distributed and non-linear distributed coding. Noxious information is well known to be conveyed at a peripheral level via independent neural pathways, such as C and A-delta fibers. However, it remains largely unknown how the neural circuits at the spinal and supraspinal level process pain information. Here, we examined whether primary somatosensory (S1) neurons process various properties of spontaneous pain and further, how S1 neurons encode pain information in terms of computational coding principle. We recorded activities of neural populations in the S1 cortex from awake head-fixed mice during formalin-induced pain using in vivo two-photon Ca2+ imaging (n = 7). Subcutaneously injected formalin elicits a biphasic pain-related behavior, i.e. a short early phase around 0–5 min, a longer period of late phase around 20–60 min, and interphase showing minimum pain-related behavior between them. To capture the dynamics of neural activities during each phase, Ca2+ imaging was performed before injection, 1–3 min, 7–9 min, 25–27 min, and 43–45 min after injection periods (basal, early, inter, late 1, and late 2 phase). First, we computed the preference index (PI) of neurons for each phase and compared those between different phases using PI scatter plots. A distinct population of neurons with different preference for each phase was observed, implying the response of S1 neurons to noxious information from the periphery during each phase is not one-dimensional. To establish whether the neuronal population of S1 cortex supports labeled line coding or distributed coding, we conducted multiple linear regression analysis with Ca2+ signals of each neuron as the outcome and binary phase information as the predictors. Most neurons (446/468) showed more than two significant beta coefficients for phases, suggesting that S1 neurons encode multiple phase information (distributed coding) rather than tuned to a specific phase. To examine the way S1 neurons distribute pain information, we first applied support vector machine (SVM) classifier with linear kernel, and successfully decoded the phase information using the neural activity of the population (average multiclass classification accuracy of 0.88, 10-fold cross-validation). We then removed all the linear components of the neural signals with respect to the phase information by orthogonalizing them. SVM with the non-linear kernel using this orthogonalized residuals still achieved significant decoding performance for each phase (average performance of decoding was 0.77, 10-fold cross-validation), while linear SVM did not. Taken together, these results suggest that S1 neurons process each phase of the spontaneous pain information using both linear and non-linear distributed coding schemes, rather than labeled line coding scheme.

## P152 Dynamic Worm: Computational investigation of locomotion through integration of connectomics, neural dynamics and biomechanics in C. elegans

### Eli Shlizerman^1,2^, Jimin Kim^2^

#### ^1^University of Washington, Departments of Applied Mathematics, Seattle, WA, United States of America; ^2^University of Washington, Electrical & Computer Engineering, Seattle, United States of America

##### **Correspondence:** Eli Shlizerman (shlizee@uw.edu)

*BMC Neuroscience* 2019, **20(Suppl 1)**:P152

The ability to discern how the brain orchestrates behavior relies on the development of successful computational approaches to link and analyze outcomes of multi-pronged investigations of the nervous system. We have developed such an integrative approach for the nematode *Caenorhabditis elegans (C. elegans)*. Specifically, we propose a model which emulates the full somatic nervous system and its response to stimuli. The model incorporates the anatomical wiring diagram, *connectome* of 279 neurons, and intra-cellular and extra-cellular *neural dynamics*. In addition, it includes layers which translate neural activity to *muscle* forces and muscle impulses to *body postures*. The model also implements an *inverse integration* procedure which modulates neural dynamics according to external forces on the body. We validate the model by injecting currents into sensory- and inter- neurons and by applying external forces to the body.

We are able to generate locomotion behaviors typical to the nematode (forward and backward) from neural stimuli or body forces. The characteristics of the movements are similar to experimentally identified ones. Furthermore, the neural dynamics associated with distinct movements can be mapped and classified using low dimensional embeddings. Accordingly, these results show that our model, and thus the connectome along with neural dynamics, encompass rhythmic activity and locomotion behaviors. Utilizing the inverse integration approach, we simulate the effect of environmental forces acting on the body through proprioceptive feedback and show that feedback can entrain and sustain movements initiated by neural or mechanical triggers. Taken together, our results show that the structure of the connectome sets specific movement patterns for the organism. These patterns are enabled by neural dynamics guided by the connectome.

The success of the dynamic worm framework to generate robust directional locomotion warrants the development of computational approaches utilizing it for identification of neural circuits and pathways associated with specific neural stimuli. We demonstrate that such studies are plausible. We apply neural stimuli experimentally known to modulate locomotion and trigger behavioral responses such as turns, escape and avoidance and show that our model supports them. We then propose computational ablation strategies, particularly (i) *ablation survey* and (ii) *conditional ablation*, for inferring neural pathways associated with these stimuli. Specifically, we demonstrate how ablation survey of all inter-neurons reveals command neurons that facilitate sensory-motor responses, such as SMDV in a sharp turn response to RIV stimulation. We also show how conditional ablation can identify alternative pathways to known command neurons and facilitate steering away behavior in the case of olfactory asymmetric neural stimuli.

In conclusion, our study provides a novel computational approach to study the interaction between the brain and behavior in *C. elegans*. Specifically, the dynamic worm framework incorporates models of interacting layers of the nervous system and bio mechanics and shows their critical role in generating locomotion movements. The outcomes of our study show that such framework can be utilized to identify brain circuits which control, mediate and generate natural behavior.

## P153 Inhibitory structures may interrupt the coherence of slow activity in deep anesthesia

### Pangyu Joo, Seunghwan Kim

#### Pohang University of Science and Technology, Physics Department, Pohang, South Korea

##### **Correspondence:** Pangyu Joo (pangyu32@postech.ac.kr)

*BMC Neuroscience* 2019, **20(Suppl 1)**:P153

EEG has been utilized as an important means of investigation on anesthetized brain because it instantaneously reflects the change in distinct brain dynamics during anesthesia. EEG during deep anesthesia is usually characterized by prominent slow oscillatory activities: slow oscillation and burst suppression. Slow oscillation and burst suppression have significantly different EEG patterns and therefore it may seem inconsistent with the studies that slow oscillation and burst suppression have qualitatively similar active-inactive patterns in neuronal level.

Our assumption is that the difference comes from synchronization of slow activity of the brain. Slow oscillation is known to be fragmentized across cortex. On the other hand, burst suppression is mainly global phenomena although it has some significant local characteristics. Moreover, the cortex is known to be hyper-excitable so that a small excitation can propagate over the cortex during burst suppression period. To sum up, we can hypothesize that spatially incoherent slow oscillation may turn into spatially coherent burst suppression due to the cortical hyper-excitability caused by reduced inhibition during burst suppression period.

We suggest two inhibitory structures which may prohibit spatial synchronization of slow activities. The two possible inhibitory structures we suggest are overlapped inhibitions and inter-inhibitory connectivity and we constructed a computational model to verify the effect of the structures. First of all, we studied the two possible inhibitory structure on a 1D chain of neurons which is divided into two modules. We found that the additional inhibitory structures on the model can disturb the coherence of slow activities. With these inhibitory structures, we constructed more realistic model where the neurons are distributed in finite space and the neurons are linked each other with probabilities depending on the distance between each neuron. In the models, the inhibitory structure could interrupt the spatial coherence of slow activities, and the degree of incoherence was dependent on the interaction range of inhibitory neurons. Also simulated EEG derived from the model exhibits several characteristics which can be generally observed in transition period between slow oscillation and burst suppression (see Fig. 1). These results suggest that inhibitory neuron can disturb the coherence of slow activities in deep anesthesia and the transition between slow oscillation and burst suppression may governed by disturbance of synchronization by inhibitory structures.

**Fig. 1:**
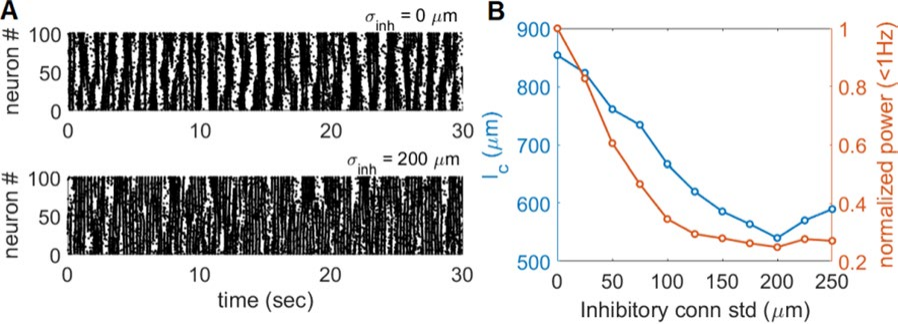
Slow activity desynchronization in 1D chain model. **a** Raster plot for no inhibition case (upper) and widely inhibited case (lower). **b** Characteristic length of correlation (l_c) and slow oscillation power

## P154 Data-driven predictive models for information processing in the small brain of Caenorhabditis elegans

### Chentao Wen, Kotaro Kimura

#### Nagoya City University, Graduate School of Natural Sciences, Nagoya, Japan

##### **Correspondence:** Chentao Wen (chintou.on@gmail.com)

*BMC Neuroscience* 2019, **20(Suppl 1)**:P154

The brain perceives information from external environments, extracts critical aspects of the information and integrates them to make decisions and regulates behaviors. How can the network of multiple neurons in the brain substantiate such sophisticated functions? Previous modelling studies focused on those large brains, e.g. in human and rodents, which are too complex to obtain accurate understanding. In contrast, recent fast 3D optical monitoring of all neuronal activities (whole brain imaging) in small brains such as zebrafish and the nematode *Caenorhabditis elegans* has allowed us to model brain activities accurately. However, these studies were mostly based on “compressed” data from principal component analysis, or simply applied correlation analyses, which cannot reveal accurate “information flow” in the brain. Here we modelled activities of all head neurons (~150) in *C. elegans*acquired by whole brain imaging [1]. Our data-driven models using machine learning techniques were able to predict individual neuronal activities by its own history and effects from other neurons, thus accurately described the potential ways of information processing in the brain. We found the tree-based models and support vector regression model are more accurate than simple linear model, while the long short-term memory (LSTM) predicts poor. Furthermore, we estimated the “causality” from our models by estimating the importance of predictors in each model. Although the estimated importance from different models are somewhat variant in details, usually those most important predictors were commonly chosen. To reveal the commonality and differences between anatomical and functional neural network, we will compare those “causality” patterns with connectome information [2] and will test our results by experimentally perturbing neuronal activities with optogenetics techniques. Our studies suggest that machine learning-based modeling of neuronal activities can reveal the patterns of information processing in small brains and may be used for understanding large brains in future.

**References**

1. Wen C, Miura T, Fujie Y, Teramoto T, Ishihara T, Kimura KD. Deep-learning-based flexible pipeline for segmenting and tracking cells in 3D image time series for whole brain imaging. *bioRxiv* 2018 Jan 1:385567.
2. White JG, Southgate E, Thomson JN, Brenner S. The structure of the nervous system of the nematode Caenorhabditis elegans. *Philos Trans R Soc Lond B Biol Sci.* 1986 Nov 12;314(1165):1–340.

## P155 A neural network model of naming impairment and treatment response in bilingual speakers with aphasia

### Claudia Penaloza^1^, Uli Grasemann^2^, Maria Dekhtyar^1^, Risto Miikkulainen^2^, Swathi Kiran^1^

#### ^1^Boston University, Department of Speech, Language and Hearing Sciences, Boston, United States of America; ^2^University of Texas at Austin, Department of Computer Science, Austin, United States of America

##### **Correspondence:** Claudia Penaloza (penaloza@bu.edu)

*BMC Neuroscience* 2019, **20(Suppl 1)**:P155

Bilinguals with aphasia (BWA) may present varying degrees of impairment in their two languages, yet both languages have potential for recovery [1]. Computational models that simulate treatment outcomes in BWA can help predicting which language should be targeted in treatment to observe the maximum potential treatment gains in both languages. Here we aimed to simulate (i) naming impairment in the native (L1) and the second language (L2) and (ii) treatment effects in the treated language and cross-language transfer effects in the untreated language in BWA. Based on our previous DISLEX model [1] we developed BiLex, a neural network model of bilingual lexical access based on self-organizing maps SOMS, that can simulate L1 and L2 naming ability in healthy bilinguals with varying degrees of language proficiency [2]. First, we trained an individual instance of the BiLex model for 13 Spanish-English BWA (mean age = 55.61 years) whose data have been previously reported elsewhere [1, 3] to simulate their naming abilities prior to stroke while accounting for their age at testing, L2 age of acquisition and L1 and L2 prestroke exposure. Next, these individual models were lesioned by gradually applying increasing amounts of damage to simulate independently the L1 and L2 naming deficits observed in standardized language tests in each BWA. Finally, each individual model was retrained to simulate treatment outcomes in the treated and the untreated language (Fig. 1). All BWA received naming treatment based on semantic feature analysis in English (n = 6) or Spanish (n = 7), and their treatment outcomes were estimated by computing effect sizes [4] on the treated and the untreated language. Significant treatment gains were observed in 10 BWA in the treated language (English n = 4; Spanish n = 6) and 3 BWA also presented significant cross-language transfer effects to the untreated language. Cross-correlations between behavioral treatment and computational model times-series data for the treated (range: 0.48 to 0.96) and the untreated language (range: −0.15 to 0.63) suggest that overall, BiLex can capture treatment effects in the language targeted in therapy for most BWA, and cross-language transfer for BWA presenting treatment gains in the untreated language. In future research, these simulations can be employed to evaluate potential treatment gains in each language and to guide clinical decisions on the language that should be targeted in treatment with BWA.

**Fig. 1:**
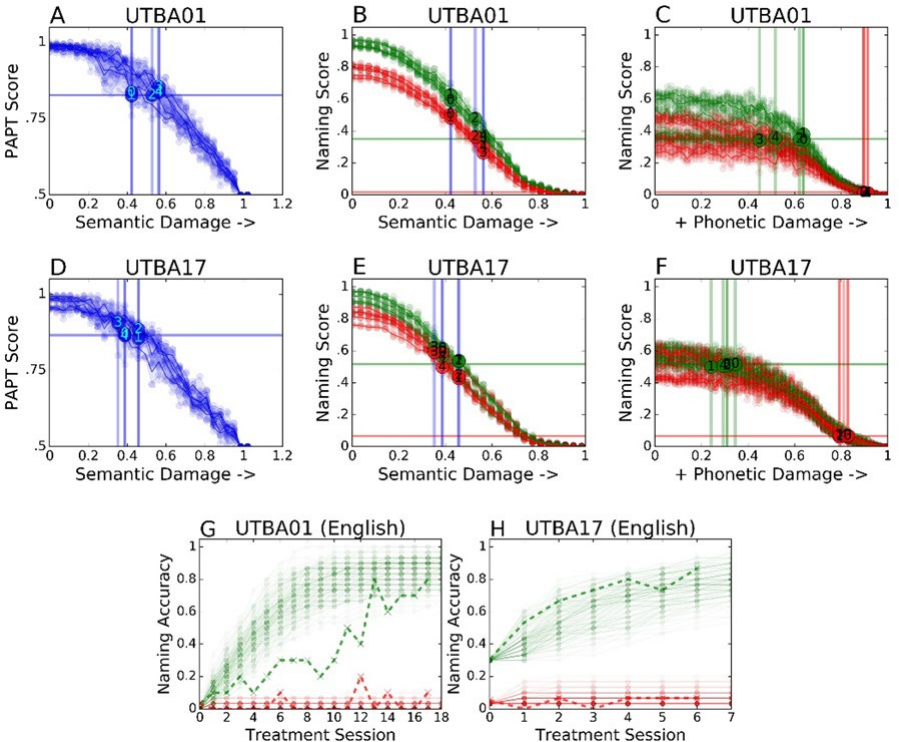
Simulations of language impairment and recovery. **a**–**f** Real (horizontal line) vs simulated (dotted lines) performance of patients UTBA01 and UTBA17 where BiLex matches (vertical line intersection) semantic (**a**, **d**) and naming deficits (**b**, **c**, **e**, **f**) in English (green) and Spanish (red). **g**–**h** Simulations (dotted lines) of treatment response (solid lines) in the treated (English) vs the untreated language

**Acknowledgments:** We thank the National Institute on Deafness and Other Communication Disorders of the National Institutes of Health for supporting Swathi Kiran with grant U01DC014922.

**References**

1. Kiran S, Grasemann U, Sandberg C, Miikkulainen R. A computational account of bilingual aphasia rehabilitation. *Bilingualism: Language and Cognition* 2013 Apr;16(2):325–42.
2. Peñaloza C, Grasemann U, Dekhtyar M, et al. BiLex: A computational approach to the effects of age of acquisition and language exposure in bilingual lexical access. *Brain and Language, In press.*
3. Kiran S, Sandberg C, Gray T, Ascenso E, Kester E. Rehabilitation in bilingual aphasia: Evidence for within-and between-language generalization. *American journal of speech-language pathology* 2013.
4. Beeson PM, Robey RR. Evaluating single-subject treatment research: Lessons learned from the aphasia literature. *Neuropsychology Review* 2006 Dec 1;16(4):161–9.

## P156 Modeling hindlimb elevation angles during intact locomotion and locomotion evoked by MLR- and epidural spinal stimulation in decerebrate cats

### Boris Prilutsky^1^, Ilya Rybak^2^, Sergey Markin^2^, Pavel Zelenin^3^, Tatiana Deliagina^3^, Yury Gerasimenko^4^, Pavel Musienko^5^, Alexander Klishko^6^

#### ^1^Georgia Institute of Technology, Biological Sciences, Atlanta, GA, GA, United States of America; ^2^Drexel University, Anatomy and Neurobiology, Philadelphia, United States of America; ^3^Karolinska Institutet, Department of Neuroscience, Stockholm, Sweden; ^4^University of California, Los Angeles, Integrative Biology and Physiology, Los Angeles, United States of America; ^5^Pavlov Institute of Physiology, Motor Physiology Laboratory, St. Petersburg, Russia; ^6^Georgia Tech, Marietta, GA, United States of America

##### **Correspondence:** Boris Prilutsky (boris.prilutsky@ap.gatech.edu)

*BMC Neuroscience* 2019, **20(Suppl 1)**:P156

It has been suggested that in order to overcome the motor redundancy of the mammalian musculoskeletal system and simplify motor control, the nervous system imposes neural constraints on redundant degrees of freedom. For example, during the cycle of locomotion, the three elevation angles of leg segments (Fig. 1B) are constrained to form a loop in a plane—the kinematic synergy of planar covariation of elevation angles. The origin of these neural constraints in the nervous system is unknown.

**Fig. 1:**
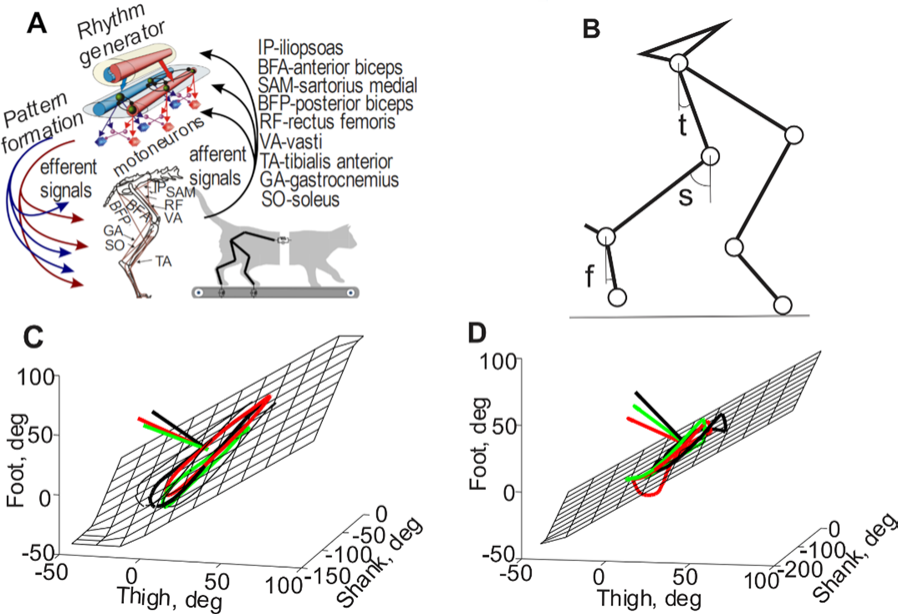
**a** Neuromechanical model of cat hindlimb spinal locomotor control. **b** Definitions of segment elevation angles. **c**, **d** Elevation angles obtained experimentally and reproduced by the neuromechanical model. Black, red and green lines are the mean trajectories for intact, MLR- and ES-evoked walking

Here, we addressed this question by computational and experimental studies of planar covariation of hindlimb segmental elevation angles during intact locomotion and during locomotion of decerebrate cats evoked by electrical stimulation of the mesencephalic locomotor region (MLR) in the brainstem and by epidural stimulation (ES) of the spinal cord at L6–L7. Possible differences in the planar covariation between intact and MLR-evoked locomotion could indicate the potential contribution of the forebrain in imposing the neural constraints. Differences in planarity between MLR- and ES-evoked locomotion would suggest the role of motion-dependent sensory feedback, which is partially disrupted by ES. If a neuromechanical model of spinal control of locomotion (Fig. 1A) reproduced the elevation angle planarity, this result would suggest that spinal locomotor circuits may be responsible for the neural constraints on the hindlimb elevation angles. We modelled electrical MLR and ES stimulations as excitatory tonic inputs to spinal interneurons representing the CPG network and afferent motion-dependent pathways, respectively. We tuned these inputs to reproduce EMG activity of major hindlimb muscles recorded during MLR-evoked and ES-evoked locomotion. Planarity of elevation angle trajectories was quantified by the percentage of total data variation (PV) accounted for by the first two principal components describing the data. Thus, the complete planarity occurred when PV = 100%.

We found that all three walking conditions exhibited planar covariation of elevation angles (Fig. 1^1^C). There was no significant difference in planarity between the MLR-evoked (PVMLR = 99%) and ES-evoked (PVES = 99%) walking and intact walking (PVInt = 98%; Fig. 1C). Since the circuitry in the spinal cord and brainstem lead to planar covariation, the forebrain does not appear to contribute to the neural constraints on the elevation angles. The neuromechanical model of spinal locomotor control reproduced planar trajectories of the elevation angles for MLR-evoked (PVMLR_m = 97%), ES-evoked (PVES_m = 98%) and intact (PVInt_m = 98%) walking (Fig. 1D). Analysis of the model suggested that neural constraints on elevation angles may originate from the CPG periodic activity.

**Acknowledgements:** Supported by NIH grantR01 NS100928.

## P157 How do local neural populations know about the predictability of sound sequences

### Shih-Cheng Chien^1^, Burkhard Maess^1^, Thomas Knoesche^2^

#### ^1^Max Planck Institute for Human Cognitive and Brain Sciences, Leipzig, Germany; ^2^MPI for Human Cognitive and Brain Sciences, Department of Neurophysics, Leipzig, Germany

##### **Correspondence:** Shih-Cheng Chien (vchien@cbs.mpg.de)

*BMC Neuroscience* 2019, **20(Suppl 1)**:P157

The brain recognizes regularity of trains of sound sequences soon after a few repetitions. A MEG study showed that the predictability of sound sequences is positively correlated to the root mean square (RMS) amplitude of the MEG signals: the participants showed higher RMS amplitude when passively listening to *regular* than *random*, and *short* than *long* sound sequences [1]. This finding is counterintuitive in the context of predictive-coding because the prediction error is assumed to decrease under more predictable conditions (i.e., *regular* and *short sequences*). Auksztulewicz and colleagues included synaptic gain modulation in their dynamic causal modeling (DCM) to account for the change in RMS amplitude [2]. However, how the synaptic gain is modulated according to the predictability is not clear. In order to clarify this, we investigate how the dynamics of neural activities and change in synaptic strength in a local neural network (representing the primary auditory cortex) may be reshaped by sound sequences under conditions of different predictability.

We construct a rate-based primary auditory cortex model that consists of excitatory (E) and inhibitory (I) neural populations, where all E populations receive external inputs in tonotopic manner. Three short-term plasticity rules are considered: (1) synaptic adaptation on E-to-E connections, (2) Hebbian learning on E-to-E connections, and (3) Hebbian learning on E-to-I connections. The 1st plasticity rule reduces the synaptic strength according to the pre-synaptic activity [3]. The 2nd and 3rd plasticity rules increase/reduce the synaptic strength according to the temporal order of firing activities between the populations [4]. The simulation results show that the 1st plasticity rule (i.e. adaptation) alone can account for the higher neural activity in *regular* than *random* sequences, but cannot account for the higher neural activity in *short* than *long* sequences, if the 2nd and 3rd plasticity rules (i.e. Hebbian learning) are not considered.

We demonstrate how synaptic gain is modulated (as suggested by [2]) during predictable sequences, which potentially links RMS amplitude and predictability of sound sequences. This study provides a possible mechanism of reshaping the synaptic strength and firing activities when only local information is available.

**References**

1. Barascud N, Pearce MT, Griffiths TD, Friston KJ, Chait M. Brain responses in humans reveal ideal observer-like sensitivity to complex acoustic patterns. *Proceedings of the National Academy of Sciences* 2016 Feb 2;113(5):E616–25.
2. Auksztulewicz R, Barascud N, Cooray G, Nobre AC, Chait M, Friston K. The cumulative effects of predictability on synaptic gain in the auditory processing stream. *Journal of Neuroscience* 2017 Jul 12;37(28):6751–60.
3. May PJ, Tiitinen H. Mismatch negativity (MMN), the deviance‐elicited auditory deflection, explained. *Psychophysiology* 2010 Jan;47(1):66–122.
4. Caporale N, Dan Y. Spike timing–dependent plasticity: a Hebbian learning rule. *Annu. Rev. Neurosci.* 2008 Jul 21;31:25–46.

## P158 Action potential propagation in long-range axonal fiber bundles

### Helmut Schmidt^1^, Thomas Knoesche^2^

#### ^1^Max Planck Institute for Human Cognitive and Brain Sciences, Leipzig, Germany; ^2^MPI for Human Cognitive and Brain Sciences, Department of Neurophysics, Leipzig, Germany

##### **Correspondence:** Helmut Schmidt (hschmidt@cbs.mpg.de)

*BMC Neuroscience* 2019, **20(Suppl 1)**:P158

With the advent of advanced MRI techniques, it has become possible to study the white matter non-invasively and in great detail. Estimating important parameters of long-range connections, such as axon diameters and myelin thickness, enables building and refining computational models of the brain that incorporate detailed effects of long-range transmission, such as distributed delays and synchronisation. If one wants to study large networks (possibly of the entire brain) and systematically investigate their parameter spaces, we need a mathematical description of action potential propagation that is sufficiently simple, yet still biologically plausible. We developed a mathematical framework that can achieve this by using a leaky integrate-and-fire framework with passive sub-threshold dynamics and explicit threshold-activated ion currents. We study different types of ion current profiles ranging from instantaneous currents modelled by delta functions, to combinations of exponential functions describing ion current profiles found experimentally. This framework allows us to derive explicit solutions for the depolarisation / hyperpolarisation profiles of action potentials. We use these to study in detail the influence of the various, potentially MRI derived, model parameters on action potential velocity. We also use this framework to study the synchronisation of action potentials between ephaptically coupled axons.

Specifically, we recover known results regarding the influence of axon diameter, node of Ranvier length and internode length on the velocity of action potentials. The velocity scales approximately linearly with the diameter in myelinated axons, and with the square root of the diameter in unmyelinated axons [1]. The velocity shows a maximum at values of node and internode length in myelinated axons that correspond to experimentally observed values [2, 3], thus suggesting that the axonal microstructure is optimised for high velocities. Furthermore, we find that the velocity depends more strongly on the thickness of the myelin sheath than was suggested by previous theoretical studies [1]. In addition, we explain the slowing down and synchronisation of action potentials in ephaptically coupled fibers through changes in the effective electrotonic length constant. We see the same effect of threshold perturbation within passive fibres by action potentials passing through neighbouring axons that has been observed experimentally [3]. Finally, we use our results to derive the delay distribution between brain regions given a specific distribution of axonal diameters within a fibre bundle. In summary, we present a solution for incorporating detailed axonal properties into a whole-brain modelling framework.

**References**

1. Rushton WAH. A theory of the effects of fiber size in medullated nerve. *J. Physiol*. 1951, 115, 101–122.
2. Hursh JB. Conduction velocity and diameter of nerve fibers. *Am. J. Physiol.* 1939, 127, 131–139.
3. Arancibia-Carcamo IL, et al. Node of Ranvier length as a potential regulator of myelinated conduction speed. *eLife* 2017, 6, e23329.
4. Katz B, Schmitt OH. Electric interaction between two adjacent nerve fibers. *J. Physiol.* 1940, 97, 471–488.

## P159 From damped oscillations to synchronous bursting: Describing the effects of synaptic depression on the collective behavior of spiking neurons

### Richard Gast^1^, Helmut Schmidt^2^, Harald Möller^3^, Nikolaus Weiskopf^3^, Thomas Knoesche^3^

#### ^1^MPI for Human Cognitive and Brain Sciences, MEG and Cortical Networks Group, Nuclear Magnetic Resonance Group, Neurophysics Department, Leipzig, Germany; ^2^Max Planck Institute for Human Cognitive and Brain Sciences, Leipzig, Germany; ^3^MPI for Human Cognitive and Brain Sciences, Department of Neurophysics, Leipzig, Germany

##### **Correspondence:** Richard Gast (rgast@cbs.mpg.de)

*BMC Neuroscience* 2019, **20(Suppl 1)**:P159

From a dynamical systems point of view, the brain can be described as a complex system consisting of a vast number of interacting, non-linear units, i.e. neurons. One prominent approach to study this system is to describe the collective behavior of large neural populations instead of the behavior of each single neuron [1, 2].Crucial to this approach is the availability of proper macroscopic descriptions of the collective behavior of interacting neurons. In a recent study, Montbrio and colleagues analytically derived a mathematically exact description of the macroscopic firing rate and membrane potential dynamics of a fully coupled population of quadratic integrate-and-fire neurons (QIF) [3]. While simple excitatory-inhibitory circuits of these QIF populations have been shown to exhibit sustained oscillatory activity [4], a single QIF population as described by Montbrio and colleagues cannot do so without periodic forcing. In this work, we extended the QIF model by a synaptic depression mechanism which weakens post-synaptic efficacies following pre-synaptic activity. The mechanism is independent of the specific synaptic model that is used and, in a globally coupled QIF network, can be coupled to the mean field such that each spike in the network triggers post-synaptic depression at all synapses. We demonstrate, that this synaptic depression mechanism can be well integrated into the macroscopic population description derived by Montbrio and colleagues and investigate its impact on the population dynamics. We show that, depending on the parametrization of synaptic depression, the QIF population can exhibit various forms of sustained oscillatory activity such as sinusoidal oscillations and bursting behavior. Furthermore, we provide a detailed description of the model’s bifurcation structure with regard to the synaptic depression. Finally, we provide an outlook in which scenarios this model could find application, i.e. which neurodynamic phenomena in the brain it could be linked to.

**References**

1. Freeman, WJ. Mass Action in the Nervous System: Examination of the Neurophysiological Basis of Adaptive Behavior through the EEG. *Academic Press London*, 1975.
2. Haken, H. Principles of Brain Functioning. *Springer Berlin Heidelberg*, 1996.
3. Montbrió E, Pazó D, Roxin A. Macroscopic Description for Networks of Spiking Neurons. *Physical Review X5 American Physical Society (APS*) 2015.
4. Schmidt H, Avitabile D, Montbrió E, Roxin A. Network mechanisms underlying the role of oscillations in cognitive tasks. *Cold Spring Harbor Laboratory* 2018.

## P160 Prefrontal oscillations modulate the propagation of neuronal activity required for working memory

### Jason Sherfey^1^, Salva Ardid^2^, Michael Hasselmo^1^, Earl Miller^3^, Nancy Kopell^2^

#### ^1^Boston University, Psychological and Brain Sciences, Boston, MA, United States of America; ^2^Boston University, Mathematics and Statistics, Boston, MA, United States of America; ^3^Massachusetts Institute of Technology, The Picower Institute for Learning and Memory, Cambridge, United States of America

##### **Correspondence:** Jason Sherfey (sherfey@bu.edu)

*BMC Neuroscience* 2019, **20(Suppl 1)**:P160

Cognition involves using attended information (e.g., stimuli, rules, responses), maintained in working memory (WM), to guide action. During a cognitive task, a correct response requires flexible, selective gating so that only the appropriate information flows at the proper time from WM to downstream effectors that carry out the response. Much evidence suggests that WM information is encoded in the firing rates of populations of neurons in prefrontal cortex (PFC). At the same time, many experiments have demonstrated separate, task-related modulation of oscillatory dynamics in PFC networks. In this work, we used biophysically-detailed modeling to explore the hypothesis that network oscillations, leveraging lateral inhibition, can independently gate responses to rate-coded items in working memory. Consistent with recent data, we modeled the superficial layers of PFC as a WM buffer that stores task-relevant information and the deep layers of PFC as an output gate that flexibly governs which information in the WM buffer is propagated downstream to guide action. We investigated two models of the WM buffer: one where items were stored in persistent spiking and one where items were stored synaptically. In the former “classic” model, item-encoding populations were in an asynchronous or rhythmic state modulated at fast beta/gamma oscillation frequencies. In the latter model, motivated by recent findings, items were stored in distributed connectivity patterns among populations with anatomically-motivated architecture; the synaptically-stored items were then made available for downstream propagation through the output gate by transient reactivation in a gamma-frequency rhythmic burst. In both cases, we found that whichever WM item induced a response in the output gate with the shortest period between spike volleys would be most reliably propagated through the output gate. Furthermore, the output gate exhibited network resonance capable of selectively propagating items with resonant oscillatory modulation. We found that network resonance of the deep layer gate can be flexibly tuned by varying the excitability of deep layer principal cells. Our results demonstrate that the propagation of WM-associated neuronal activity can be modulated by tuning either the oscillatory properties of populations encoding WM items, themselves, or the resonant properties of the output gate through which item-encoding activity must propagate to reach downstream effectors. In our PFC model, these dynamics reveal how population rate-coded items embedded in superficial beta and gamma oscillations can be alternately selected by tuning network resonance in the deep layers of PFC depending on task demands. Thus, our model predicts that the experimentally-observed modulation of PFC beta and gamma oscillations could leverage network resonance and lateral inhibition to govern the flexible routing of signals in service of cognitive processes like gating outputs from working memory and the selection of rule-based actions.

## P161 **Model of respiration’s projections to the brain reproduces physiological changes and predicts emotion’s cognitive influences**

### Julius Cueto^1^, Hoshinori Kanazawa^1^, Shogo Yonekura^1^, Satoko Hirabayashi^2^, Noritoshi Atsumi^2^, Masami Iwamoto^2^, Yasuo Kuniyoshi^1^

#### ^1^The University of Tokyo, Department of Mechano-Informatics, Tokyo, Japan; ^2^TOYOTA CENTRAL R&D LABS., INC., Nagakute, Japan

##### **Correspondence:** Julius Cueto (cueto@isi.imi.i.u-tokyo.ac.jp)

*BMC Neuroscience* 2019, **20(Suppl 1)**:P161

Recent studies show that cognition is biased by (nasal) respiration and presented emotional stimuli, providing insight to the physical origins of emotion’s influences. However, the mechanism for this influence remains uncertain, with neuro-modulation through the locus coeruleus (LC), or influences from the piriform cortex being suggested as possible candidates [1]. To compensate for the difficulty of measuring the activity of LC in humans while performing tasks, a combined approach of both human behavioral experimentation and computational modeling was taken.

In the human behavioral experiments, participants were presented with emotional faces (fearful or surprised) and were asked to identify the emotions exhibited or respond to a cue presented subsequently [2, 3]. The change in the time taken to identify the emotions, or respond to the cues, were used as a measure for the change in cognition. Respiration during task performance was measured as a phase calculated from the chest movements of participants.

The computational model was constructed to incorporate, the influence of LC activity on human cognition, the mechanical, chemical and neurological activities involved in respiration, and the influence of respiration/emotion on the human brain. The influence of respiration and emotion was newly formed in the form of connections to the LC, combining experimental findings of the involved brain regions and in vitro connections.

Results from the behavioral experiments showed different emotional stimuli having a different influence on the responses. Furthermore, observations on how this influence changed with respiration, in each task, showed the presented emotions’ influence on cognition to change at the level of specific respiration phases, with each emotion showing different influences.

The computational model showed activity that matches experimental findings, for example, LC activity increasing in magnitude and synchrony during task performance. In addition to the physiological resemblance, the model showed different responses when provided with different emotional inputs, which corresponds to the presentation of fearful and surprised faces. Furthermore, this change in response created by the introduction of emotion, was observed to change at the level of respiration phases. By changing the model’s threshold to produce a response, the model was able to reproduce the influences of the emotions at the respiration phase level, matching the experimental results closely for both tasks.

Our findings implicate that changes in human cognition through respiration and presentation of emotion, can be reproduced by using neuro-modulation/LC, indicating the importance of this mechanism in the human body. Furthermore, our model shows an ability to match humans, making it possible to be used to predict human responses, or to provide machines the ability to respond to emotional stimuli in a human-like manner.

**References**

1. Heck D, McAfee S, Liu Y, et al. Breathing as a Fundamental Rhythm of Brain Function. *Frontiers in Neural Circuits* 2017, 10, 115.
2. Zelano C, Jiang H, Zhou G, et al. Nasal Respiration Entrains Human Limbic Oscillations and Modulates Cognitive Function. *Journal of Neuroscience* 2016, 36 (49), 12448–12467.
3. Park G, Thayer J. From the heart to the mind: cardiac vagal tone modulates top-down and bottom-up visual perception and attention to emotional stimuli. *Frontiers in Psychology* 2014, 5, 278.

## P162 EEG simulation reveals that changes in cortical morphology and global connectivity during development affect neonatal EEG

### Tomoaki Morioka^1^, Hoshinori Kanazawa^2^, Keiko Fujii^2^, Yasuo Kuniyoshi^2^

#### ^1^The University of Tokyo, Tokyo, Japan; ^2^The University of Tokyo, Graduate School of Information Science and Technology, Department of Mechano-Informatics, Tokyo, Japan

##### **Correspondence:** Tomoaki Morioka (morioka@isi.imi.i.u-tokyo.ac.jp)

*BMC Neuroscience* 2019, **20(Suppl 1)**:P162

Obtaining electroencephalograms (EEGs) from neonates is important for the clinical assessment of several pathologies, such as neonatal seizures and hypoxic ischemic encephalopathy. However, because multiple factors mature simultaneously during neonatal development, how the specific factors cause developmental changes in EEG remain unclear. Two potential factors are cortical morphology and global connectivity, as both have been reported to affect spectral features of EEG signal during early development. In this study, we constructed EEG simulations based on real cortical morphology and global connectivity data and described how their developmental changes affected EEG signals. We first constructed a simple geometric model to confirm the relationship between cortical morphology and the EEG signal. In a simple model, neural activity propagated along a two-dimensional sine curve, and EEG signals are estimated at model electrode positions. To simulate developmental changes in cortical morphology, we manipulated spatial frequency and the amplitude of the sine curve, which correspond to the number of sulci and the depth of the sulcus, respectively. Next, we constructed a thalamocortical model based on cortical morphology and global connectivity that was acquired from fetal and neonatal magnetic resonance imaging at 28 and 37 weeks after conception, respectively. The model neuronal activity was generated based on a spiking neural network composed of about one million neurons. We approximated postsynaptic potentials with electric dipoles and generated EEG signals as a superposition of these electric field potentials. The EEG signals obtained from the data at 28 and 37 weeks after conception was verified using the following three features: (1) the power spectral density at each electrode, (2) the activation synchrony index (ASI) between EEG signal pairs of each electrode, and (3) the global structure formed by a minimum spanning-tree graph network. By using the simple geometric model, we confirmed the influence of cortical morphology on EEG signals. The peak frequency of the EEG signal became higher as the morphological frequency increased. Additionally, we found that as the amplitude of the cortical morphology increased, the absolute value of the power spectral density at the peak frequency of the EEG signal became large, which affected the frequency distribution of the EEG signal. The same effect of cortical morphology on EEG characteristics was observed in the thalamocortical simulation. We also examined the influence of global connectivity on the EEG signal using the thalamocortical simulation. We compared the EEG signal obtained using the global connectivity at 28 weeks with that using the connectivity at 37 weeks. Although the frequency characteristics did not change, the synchrony between neurons pairs did, as indicated by differences in ASI, the index for quantitatively measuring the synchrony of signals between two electrodes. In conclusion, we constructed a biologically based EEG simulation that enabled us to independently examine the influence of two developmental factors on neonatal EEG. Furthermore, our simulation suggests that cortical morphology influences EEG frequency distribution, while global connectivity influences the functional connectivity.

## P163 Distinct temporal structure of ACh receptor activation determines responses of VTA activity to endogenous ACh and nicotine

### Ekaterina Morozova^1^, Phiilippe Faure^2^, Boris Gutkin^3^, Christopher Lapish^4^, Alexey Kuznetsov^5^

#### ^1^Brandeis University, Volen Center for Complex systems, Waltham, United States of America; ^2^Sorbonne Université, UPMC Univ Paris 06, INSERM, CNRS, Neuroscience Paris Seine - Institut de Biologie Paris Seine, Paris, France; ^3^École Normale Supérieure, Paris, France; ^4^Indiana University-Purdue University, Department of Psychology, Indianapolis, IN, United States of America; ^5^Indiana University-Purdue University at Indianapolis, Department of Mathematical Sciences, Indianapolis, United States of America

##### **Correspondence:** Boris Gutkin (boris.gutkin@ens.fr)

*BMC Neuroscience* 2019, **20(Suppl 1)**:P163

Tobacco use is a worldwide leading cause of preventable mortality. The addictive component of tobacco, nicotine, exerts its effects through nicotinic acetylcholine receptors (nAChRs). Among the different nAChRs, the β2-containing nAChRs (β2-nAChRs) have been shown to play a crucial role in the positive rewarding properties of nicotine and to be particularly densely expressed in the mesolimbic reward system. Specifically, nAChRs regulate dopamine (DA) which is released by the mesolimbic system. nAChRs are expressed on DA neurons in the ventral tegmental area (VTA) as well as neighboring GABA neurons that modulate DA neuron activity. ACh and nicotinic regulation of DA neuron activity is complex and its understanding is incomplete. With our model, we provide mechanisms for several apparently contradictory experimental results. First, systemic knock out of ß2-nAChRs drastically reduces DA neurons bursting even though the major glutamatergic (Glu) afferents that have been shown to evoke this bursting stay intact. Second, the most intuitive way to rescue this bursting—by re-expressing the nAChRs on VTA DA neurons—fails. Third, nAChR re-expression on VTA GABA neurons rescues DA neurons bursting and increases their firing rate under the influence of ACh input, whereas nicotinic application results in the opposite changes in VTA DA neurons firing. The model shows that, first, without ACh receptors, Glu excitation of VTA DA and GABA neurons remains balanced and cancel each other. Second, re-expressing the ACh receptors on the DA neurons provides an input that impedes membrane repolarization and is ineffective in restoring firing of DA neurons. Third, the distinct responses to ACh and nicotine are due to distinct temporal patterns of these inputs: pulsatile vs. continuous. All together this study highlights how β2-nAChRs influence co-activation of VTA DA and GABA neurons required for DA neuron bursting and, thus, motivation and saliency signals carried by these bursts.

## P164 Basal Ganglia role in learning reward actions and executing previously learned choices

### Alexey Kuznetsov

#### IUPUI, Indianapolis, IN, United States of America

##### **Correspondence:** Alexey Kuznetsov (alexey@math.iupui.edu)

*BMC Neuroscience* 2019, **20(Suppl 1)**:P164

The basal ganglia (BG), a collection of nuclei located deep beneath the cerebral cortex is involved in learning and selection of rewarded actions. We analyze the mechanism by which the BG enable learning of the rewarded action. We have implemented a rate model of a BG-thalamo-cortical loop and simulated its performance in a standard instrumental conditioning task, in which an animal is rewarded for choosing one of two options. We have shown that potentiation of cortico-striatal synapses enables learning of the rewarded option (Fig. 1). However, later these synapses become redundant as direct connections between prefrontal and premotor cortices (PMC-PFC) potentiate by Hebbian learning. After we switch the reward to the previously unrewarded option (reversal), the BG are again responsible for switching to the new option. Due to the potentiated direct cortical connections, the system is bias to the previously rewarded choice, and establishing new choice requires greater number of trials. We then modified our model to reproduce pathological physiology of mild Parkinson (PD) and Huntington (HD) diseases. We have found that, in PD, the PMC activity levels become extremely variable, which is caused by oscillations arising in the BG-thalamo-cortical loop. The model reproduces severe impairment of learning and predicts that it is caused by these oscillations as well as a reduced reward prediction signal. By contrast, in HD, the potentiation of the PFC-PMC connections shows much better learning, but the altered BG output disrupts expression of the rewarded choices. This results in random switching between rewarded and unrewarded choices resembling an exploratory phase that never ends. Our results reconcile the apparent contradiction between the BG involvement in execution of previously learned, in particular habitual actions and no impairment of these actions after the BG output is ablated by lesions or deep brain stimulation: The model predicts that BG-thalamo-cortical loop simply conforms to the previously learned choice in the healthy state, but impedes the choice in the disease states.

**Fig. 1:**
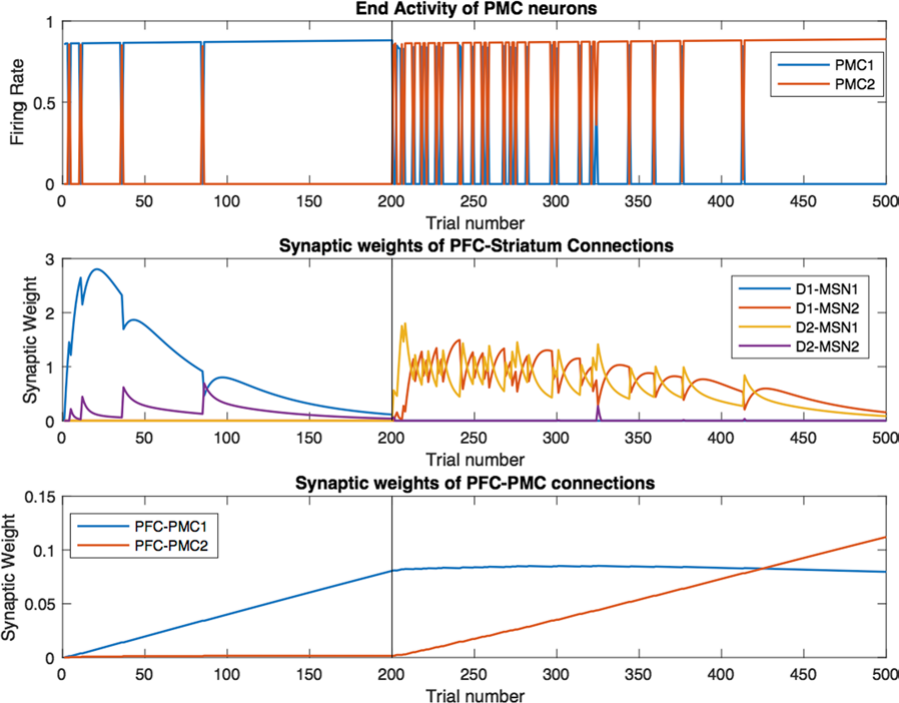
Trial-by-trial dynamics of the choices and underlying modulation of synaptic weights in the Healthy BG model. Trials 1–199:initial learning; trials 200-500: reversal **a** A higher activity of PMC1 (blue) manifests choice 1, whereas higher activity of PMC2 manifests choice 2. **b** Synaptic weights of the PFC to striatum connections. **c** Synaptic weights of the PFC to PMC connections

## P165 Short-term plasticity in PV and SST interneurons enhances neural code propagation in the feedforward network model

### Jeongheon Gwak, Jeehyun Kwag

#### Korea University, Brain and Cognitive Engineering, Seoul, South Korea

##### **Correspondence:** Jeongheon Gwak (john.gwak@ncl.korea.ac.kr)

*BMC Neuroscience* 2019, **20(Suppl 1)**:P165

Neural codes, such as rate code (spike firing rate) and temporal code (precise spike timing), should be reliably propagated across the feedforward network (FFN) of the brain for successful neural information processing. However, how distinct subtypes of inhibitory interneurons modulate the propagation of rate and temporal codes is unclear. Especially, excitatory inputs onto parvalbumin-positive (PV) and somatostatin-positive interneurons (SST) show different short-term plasticity (STP) characteristics where excitatory postsynaptic potentials (EPSPs) onto PV display short-term depression, while EPSPs onto SST display short-term facilitation. To address such question, we built four different types of 5-layer FFN models, composed of Hodgkin-Huxley–type excitatory neurons only (EX-FFN), EX-FFN with PV (EX-PV-FFN) or SST (EX-SST-FFN) or both (EX-PV-SST-FFN) to investigate how STP characteristics of PV and SST modulate the propagation of rate and temporal codes. In investigating the propagation of rate code, different asynchronous spike firing rates (5-100 Hz) were given to the input layer of each FFN model and the firing rate in the final layer was used to analyze input-output ratio of firing rate (I/O-ratio). I/O-ratio in EX-FFN was above 1 for all input firing rates, indicating failure of rate code propagation. However, I/O-ratio was close to 1 for input firing rates at high gamma-frequency ranges (50-100 Hz) in EX-PV-FFN while I/O-ratio was close to 1 for input firing rates at alpha/beta-frequency ranges (10-30 Hz) in EX-SST-FFN. Since PV and SST had differential effects on propagating firing rates, we investigated how different ratios of PV and SST would influence rate code propagation.

We found that PV and SST ratio of 6:4 in EX-PV-SST-FFN could reliably propagate firing rates across all frequency ranges, indicating that distinct ratio of PV and SST with differential STP properties could selectively enhance the propagation of rate code. In investigating the propagation of temporal code, we designed a spike train with spike timing pattern that monotonically decreased or increased in inter-spike interval (ISI) to investigate firing rate-dependent temporal code propagation. Temporal similarity (SR), which measures synchronization between two spike trains with reliable propagation indicated as 1, was used to analyze propagation of input spike trains. SR values in EX-FFN were less than 1 for all ISIs, indicating failure of temporal code propagation. However, SR values were close to 1 for ISIs that had instantaneous firing rate (IFR) at gamma-frequency ranges (30-50 Hz) in EX-PV-FFN while SR values were close to 1 for ISIs that had IFR at alpha/beta-frequency ranges (10-20 Hz) in EX-SST-FFN. We again examined the effect of different ratios of PV and SST on temporal code propagation. We found that PV and SST ratio of 6:4 in EX-PV-SST-FFN could reliably propagate input spike trains across all IFR ranges. Overall, our results indicate that PV and SST with distinct STP characteristics activated at 6:4 ratio could allow for reliable propagation of both rate and temporal codes, suggesting that STP mechanisms of distinct interneurons might play important roles in reliable transmission of neural information in the cortical network.

**Acknowledgment**: This study was supported by Human Frontier Science Program (RGY0073/2015) and the National Research Foundation of Korea (NRF) grant funded by the Korea government (MIST) (NRF-2016R1A1A1A05921614).

## P166 Differential roles of PV and SST interneurons in spike-timing pattern propagation in the cortical feedforward network

### Hyun Jae Jang^1^, James M. Rowland^2^, Hyowon Chung^1^, Blake A Richards^3^, Michael M. Kohl^2^, Jeehyun Kwag^1^

#### ^1^Korea University, Department of Brain and Cognitive Engineering, Seoul, South Korea; ^2^University of Oxford, Department of Physiology, Anatomy, and Genetics, Oxford, United Kingdom; ^3^University of Toronto Scarborough, Department of Biological Sciences, Toronto, Canada

##### **Correspondence:** Hyun Jae Jang (dragon88hj@gmail.com)

*BMC Neuroscience* 2019, **20(Suppl 1)**:P166

In primary somatosensory cortex (S1), tactile information is believed to be encoded in temporally complex spike-timing patterns using two different types of neural codes: rate code (spike firing rate) and temporal code (precise timing of spikes). For effective sensory information processing, such neural code-carrying spike-timing patterns should reliably propagate to downstream neurons across multiple layers of the feedforward network (FFN) of the cortex. Inhibitory neural circuits have been suggested to gate the propagation of spikes in FFN. However, how distinct subtypes of interneurons, such as parvalbumin-positive (PV) and somatostatin-positive (SST) interneurons, recruit distinct neural circuit motifs such as feedforward inhibition (FFI) and feedback inhibition (FBI), to gate the propagation of neural code-carrying spike-timing pattern is unclear. Here, to address this question, we performed *in vivo* single-unit recording in S1 during whisker stimulation with optogenetic modulation of PV and SST interneurons and compared the *in vivo*-recorded results with that from *in silico* three-layer FFN model with FFI or FBI.

By analyzing layer-wise spike-timing coherence similarity, we found that *in vivo* spike-times recorded in layer 4, the main recipient layer of S1, reliably propagated across downstream layers 2/3, 5, and 6. Optogenetic activation of blue-light sensitive opsin (channelrhodopsin2, ChR2)-expressing PV or SST interneurons during whisker stimulation revealed that activation of ChR2-expressing PV interneuron preferentially facilitated the propagation of spike-times with low firing rate (<12 Hz) while activation of ChR2-expressing SST interneuron facilitated the propagation of spike-times with high firing rate (>12 Hz). By comparing*in vivo*optogenetic modulation results with the*in silico* FFN model with FFI and FBI network, we found that ChR2-expressing PV interneuron *in vivo* preferentially recruited FFI while ChR2-expressing SST interneuron *in vivo* preferentially recruited FBI. These results suggest that PV and SST interneurons preferentially recruit distinct inhibitory network motifs to function as complementary frequency-selective gates, which may have critical roles in neural information processing in the cortex.

**Acknowledgements**: This study was supported by Human Frontier Science Program (RGY0073/2015) and the National Research Foundation of Korea (NRF) grant funded by the Korea government (MIST) (NRF-2016R1A1A1A05921614).

## P167 The State of the MIIND Simulator

### Marc de Kamps^1^, Hugh Osborne^2^, Lukas Deutz^3^, Mikkel Elle Lepperød^4^, Yi Ming Lai^5^

#### ^1^University of Leeds, School of Computing, Leeds, United Kingdom; ^2^University of Leeds, Institute for Artificial and Biological Computation, School of Computing, United Kingdom; ^3^University of Leeds, School of Computing, Leeds, Germany; ^4^University of Oslo, Institute of Basic Medical Sciences & Center for Integrative Neuroplasticity, Oslo, Norway; ^5^University of Nottingham, School of Mathematical Sciences, Nottingham, United Kingdom

##### **Correspondence:** Marc de Kamps (m.dekamps@leeds.ac.uk)

*BMC Neuroscience* 2019, **20(Suppl 1)**:P167

MIIND is a population level simulator, centered around population density techniques (PDTs). Populations are modeled by a density function, representing the distribution of neurons across their state space. By modeling the evolution of the density function in response to individual neurons receiving Poisson spike trains from elsewhere, their subthreshold dynamics can be captured, and the fraction of neurons crossing the threshold can be estimated, which leads to a reliable prediction of the population firing rate in response to external input. PDTs are a main technique for calculating so-called transfer functions that are used in linear response theory. Here, we present an extension of PDTs that allows the point model neurons to be two dimensional [1], e.g. Izhikevich, adaptive-exponential-integrate-and-fire, Fitzhugh-Nagumo and others, and that facilitates grouping individual populations into large networks. The method is agnostic regarding the neural model, which can be presented to the simulator as a mesh, and the method is universal in that sense: if a model can be represented accurately by a mesh in state space, the method will work. Stationary points and nullcline crossings require extra care, but can be handled. We are not restricted to the diffusion limit: arbitrarily large synapses can be considered. For large networks, we have shown that the 2D techniques are as fast as direct simulation, but use an order of magnitude less memory. A CUDA implementation allows the simulation of networks that otherwise must be simulated on an HPC cluster to be moved on a single PC equipped with GPGPU. MIIND provides an open source implementation of the PDTs. It allows users to configure simulations using an XML script. Python scripts convert this into C++ and CUDA. A Docker container simplifies installation. We will present several examples of population and network simulations. (Fig. 1) describes a population of conductance-based neurons with a two-dimensional state space, spanned by the membrane potential and the conductance variable g.

**Fig. 1:**
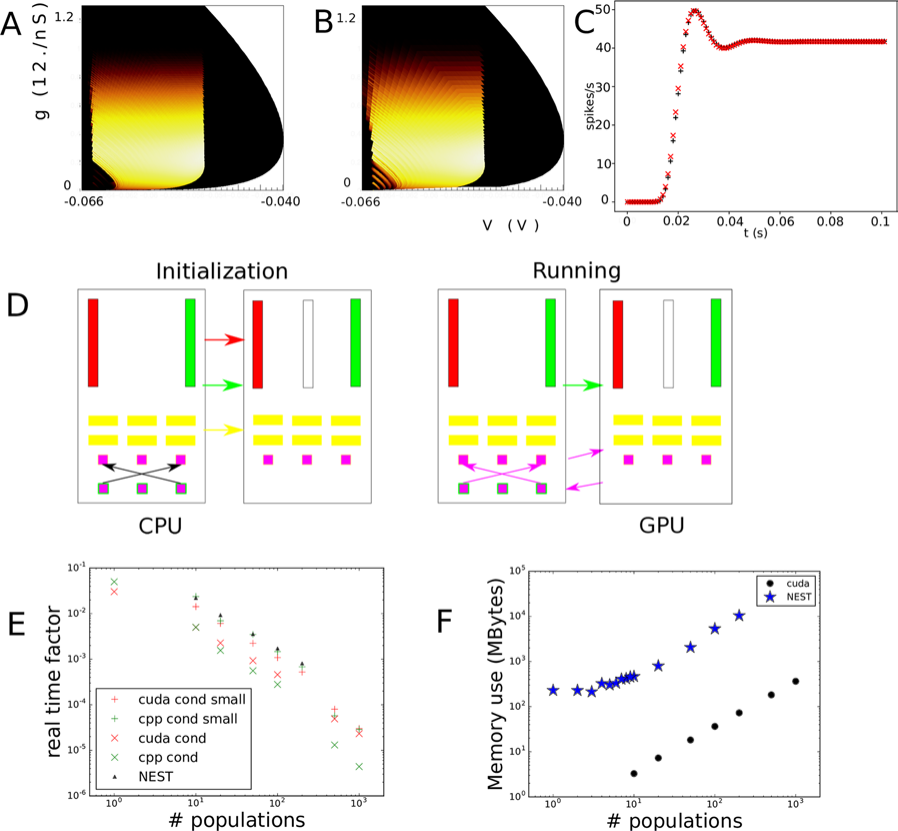
Population state (fine: A, coarse B). The firing rate caused by neurons pushed over threshold (sharp edge in **a**, **b**). Connectivity is applied to output firing rates, and converted to input firing rates on the CPU. **c** The density itself is maintained on the GPU to minimize data transfer (**d**). The method is about as fast as a NEST simulation (**e**), but uses an order of magnitude less memory (**f**)

**Reference**

1. de Kamps M, Lepperød M, Lai YM. Computational geometry for modeling neural populations: From visualization to simulation. *PLoS Computational Biology* 2019 15(3): e1006729.

## P168 Optimal conditions for reliable representation of asynchronous spikes in feed-forward neural networks

### Sayan Faraz^1^, Milad Lankarany^2^

#### ^1^University of Toronto, Toronto, Canada; ^2^The Krembil Research Institute - University Health Network, Computational Neuroscience Program, Toronto, Canada

##### **Correspondence:** Milad Lankarany (milad.lankarany@gmail.com)

*BMC Neuroscience* 2019, **20(Suppl 1)**:P168

While the stable propagation of synchronous spikes is well understood and relatively easy to implement by neuronal models, reliable propagation of firing rate—specifically slow modulation of asynchronous spikes in fairly short time windows [20–500] ms—across multiple layers of a feedforward network (FFN) receiving background synaptic noise has proven difficult to capture in spiking models [1]. Particularly, the firing rate of asynchronous spikes is attenuated in the first layer of an FFN. In this study, we explore how information of asynchronous spikes is disrupted in the first layer of a typical FFN, and which factors can enable reliable information representation. In a typical FFN, each layer comprises a certain number (network size) of excitatory neurons—modeled by leaky integrate and fire (LIF) model—receiving correlated input (common stimulus (modeled by Ornstein–Uhlenbeck process of time constant 100 msec) from the upstream layer) plus independent background synaptic noise. We develop a reduced network model of FFN which captures main features of a conventional all-to-all connected FFN. Exploiting the reduced network model, synaptic weights are calculated using a closed-form optimization framework that minimizes the mean squared error between reconstructed stimulus (by spikes of the first layer of FFN) and the original common stimulus. We further explore how representation of asynchronous spikes in an FFN changes with respect to other factors like the network size and the level of background synaptic noise while synaptic weights are optimized for each scenario. We found that not only synaptic weights but also the network size and the level of background synaptic noise are crucial to preserve a reliable representation of asynchronous spikes in the first layer of an FFN. This work sheds light in better understanding of how information of slowly time-varying fluctuations of the firing rate can be transmitted in multi-layered FFNs.

**Reference**

1. Kumar A, Rotter S, Aertsen A. Spiking activity propagation in neuronal networks: reconciling different perspectives on neural coding. *Nature Reviews Neuroscience* 2010 Sep;11(9):615.

## P169 Optogenetic data mining with empirical mode decomposition

### Sorinel Oprisan^1^, Xandre Clementsmith^1^, Tams Tompa^2^, Antonieta Lavin^3^

#### ^1^College of Charleston, Department of Physics and Astronomy, Charleston, SC, United States of America; ^2^University of Miskolc, Miskolc, Hungary; ^3^Medical University of South Carolina, Charleston, SC, United States of America

##### **Correspondence:** Sorinel Oprisan (oprisans@cofc.edu)

*BMC Neuroscience* 2019, **20(Suppl 1)**:P169

Optogenetically evoked local field potentials (LFPs) were recorded from the medial prefrontal cortex (mPFC) of male PV-Cre mice infected with a viral vector [1]. We recorded multiple basal conditions followed by a systemic injection with D1 receptors antagonist SCH23390 and/or D2 antagonist sulpiride. Optical stimulation was provided by a blue laser (473 nm) stimulus delivered to mPFC through a fiber optic every 2 seconds and each trial was repeated 100 times. The extracellular signals were sampled at 10 kHz and stored for offline analysis.

We previously used nonlinear dynamics tools, such as delay embedding and false nearest neighbors, to estimate the embedding dimension and the delay time for attractor reconstruction from LFPs. Delay embedding has relatively limited use in the case of short, non-stationary, and nonlinear time series.

To alleviate these concerns, we also used the Empirical Mode Decomposition (EMD) to extracts the Intrinsic Mode Functions (IMFs) from our data [2]. The first IMF represents the fast variations of data and capture the short-term processes in the system. The second IMF contains information regarding the next longer temporal scale present in the data. We compared the phase resetting determined using the delay embedding dendrogam against the Hilbert transform of IMFs and found a good agreement between the two methods.

**Acknowledgments:** We acknowledge the support of R&D grant from the College of Charleston and support for a Palmetto Academy site from the South Carolina Space Grant Consortium.

**References**

1. Dilgen JE, Tompa T, Saggu S, Naselaris T, Lavin A. Optogenetically evoked gamma oscillations are disturbed by cocaine administration. *Frontiers in cellular neuroscience* 2013, 7:213.
2. Huang NE, Shen Z, Long SR, et al. The Empirical Mode Decomposition and the Hilbert Spectrum for Nonlinear and Nonstationary Time Series Analysis. *Proceedings of the Royal Society of London A*. 1998, 454: 903–995.

## P170 High channel count electrophysiological recordings in prefrontal cortex in a novel spatial memory task.

### Claudia Böhm, Albert Lee

#### HHMI Janelia Research Campus, Ashburn, United States of America

##### **Correspondence:** Claudia Böhm (boehmc@janelia.hhmi.org)

*BMC Neuroscience* 2019, **20(Suppl 1)**:P170

Past research on spatial working memory has largely focused on simple tasks with a binary choice, such as the T-maze. In these tasks, the animal can memorize the location of the goal or the route to the goal from the start location. In addition, such tasks tend to result in stereotyped behaviors for each goal that may themselves produce goal-associated neural correlates. Thus, the animal’s strategy to solve the task and the interpretation of neural correlates can be ambiguous. We have devised a novel spatial memory task where rats are required to flexibly encode three spatially distinct goals on a trial-by-trial basis. The goals can be reached via multiple routes, one of which is available in each trial. Knowledge of the available route is only gained after a delay period in which the animal has to perform a nose poke in one of three randomly chosen start positions. This design forces the animal to memorize the spatial location of the goal instead of planning a route from start position to goal position. This allows us to dissociate between neural correlates of route planning and goal representation as well as investigate the nature of egocentric and allocentric spatial goal representations in working memory. In such cognitively demanding tasks a large population of neurons in several brain regions, including prefrontal areas, are expected to be required to coordinate their activity and encode task-relevant variables and rules. Sampling a sufficiently large number of neurons at high temporal accuracy poses a challenge to current electrophysiological recording technology. Here we have employed Neuropixels probes, a new type of high channel count silicon probe featuring nearly a thousand recording sites along a single 10 mm shank, of which 384 can be recorded simultaneously. These probes are ideally suited for our experiments as the shank spans multiple task-relevant brain regions including anterior cingulate cortex, prelimbic cortex and infralimbic cortex. This technology has allowed us to record activity from 100-200 frontal cortical neurons simultaneously in freely behaving rats performing the complex spatial working memory task described above. The high number of neurons also facilitates single trial analysis, which is highly relevant since the working memory content must be updated from trial to trial and be instantly accessible despite other cognitive requirements, distractions or activities of the subject. Furthermore, especially in higher cognitive areas, working memory might not be statically encoded and instead could be constantly reshaped by ongoing local and non-local network dynamics, limiting the success of trial averaged analysis. We have found that current spatial location at the start positions can be clearly and unambiguously decoded by the combined firing rates of multiple neurons over a range of timescales, ranging from hundreds of milliseconds to seconds. The representation of goal location in our well-controlled task is more complex. Ongoing analysis is focused on other biologically plausible forms of representation, such as sequences of neural activity or coordinated ensemble activity.

## P171 Exploring the machine learning model space commonly used in Neuroimaging using Automated Machine Learning

### Jessica Dafflon, Federico Turkheimer, James Cole, Robert Leech

#### King’s College London, Centre for Neuroimaging Sciences, London, United Kingdom

##### **Correspondence:** Jessica Dafflon (jessica.dafflon@gmail.com)

*BMC Neuroscience* 2019, **20(Suppl 1)**:P171*BMC Neuroscience* 2019, **20(Suppl 1)**:P17

**Introduction:** A plethora of machine learning models have been trained to learn the relationship between age and brain structure. However, the creation and analysis of optimal machine learning models is a cumbersome and time-consuming process. It not only requires expert knowledge of the used tools, their limitation and assumptions but it is also subject to experimental bias.To overcome these limitations and extensively search the parameter space, we used automated machine learning to extract the best age prediction models from the data and analysed their similarities. Automated machine learning consists of extensively testing different algorithms combinations with various parameters to find the model with the appropriate combination that maximises the predicted accuracy. In this project, we have used a tree-based genetic programming algorithm (TPOT [1]) to find the best set of models and evaluate their prediction accuracy.

**Methods:** Using data from N = 1227 healthy individuals (aged 18–98 years, mean age = 36.89), we trained different algorithms which included among others k-nearest Neighbours, Random Forrest Regression and Gaussian Processor Regression. For each of the analysed subjects, we used 140 features that describe the volume and thickness of different brain areas and were obtained using Freesurfer recon-all [2] on the T1 images. TPOT uses genetic programming to find the best pipeline with the highest accuracy. It does so not only by combining pipelines evaluated in the previous generation but also by performing synthetic feature construction. We then run the analysis multiple times and explored the accuracy and the similarities of the models suggested by TPOT over the different runs.

**Results:** For each run TPOT suggested a pipeline that has a MAE similar to the current literature [3]. We also systematically explored the importance of feature generation, population size and cross-over and mutation rate on the final pipeline suggested by TPOT. By mutating previous models TPOT analyses a large pool of models with a high variability on prediction, however, only the best models are passed to the next generations. Although the initial population size is crucial to the ending model, Gaussian Process regressors tend to lead to a higher accuracy.

**Conclusion:** We showed that TPOT can build machine learning pipelines that achieve MAE similar to the current state of the art [3], and that it also creates innovative pipelines consisting of the combination of different models. Therefore, our study shows the potential of using automatic machine learning to reduce prior assumptions, broaden the range of models used for predicting brain age, and improving the generalisability and reproducibility of the findings.

**References**

1. Olson RS, Urbanowicz RJ, Andrews PC, Lavender NA, Moore JH. Automating biomedical data science through tree-based pipeline optimization. *In European Conference on the Applications of Evolutionary Computation* 2016 Mar 30 (pp. 123–137). Springer, Cham.
2. Dale AM, Fischl B, Sereno MI. Cortical surface-based analysis: I. Segmentation and surface reconstruction. *Neuroimage* 1999 Feb 1;9(2):179–94.
3. Cole JH, Franke K. Predicting age using neuroimaging: innovative brain ageing biomarkers. *Trends in neurosciences* 2017 Dec 1;40(12):681–90.

## P172 Effects of cellular excitatory-inhibitory composition on neuronal dynamics

### Oleg Vinogradov^1^, Nirit Sukenik^2^, Elisha Moses^2^, Anna Levina^3^

#### ^1^University of Tübingen, Department of Computer Science, Tübingen, Germany; ^2^Weizmann Institute of Science, Physics of Complex Systems, Rehovot, Israel; ^3^Universtity of Tübingen, Tübingen, Germany

##### **Correspondence:** Oleg Vinogradov (oleg.vinogradov@tuebingen.mpg.de)

*BMC Neuroscience* 2019, **20(Suppl 1)**:P172

Various brain regions have distinct and highly conserved ratios of excitatory and inhibitory neurons. For instance, cerebral cortex typically includes around 20% of inhibitory neurons. However, it is not clear whether unphysiological ratios would change collective neuronal dynamics or jeopardize the balance of excitation and inhibition on a synaptic level. To investigate this question, we developed a platform that allowed us to culture hippocampal networks with various fractions of inhibitory neurons. We also study how cellular composition affects neuronal dynamics in finite network models with balanced excitation/inhibition currents and neuronal adaptation.

We used fluorescence-activated cell sorting to isolate inhibitory and excitatory neurons and seeded them while keeping prescribed inhibitory percentages. We recorded the calcium dynamics of these cultures. All of them developed spontaneous network activity manifested in full network bursts. The cultures with 10–80% of inhibitory cells showed surprisingly similar mean inter-burst intervals, which were indistinguishable from unsorted control cultures that usually contain 20–30% of inhibitory neurons. Fully excitatory and fully inhibitory cultures had significantly longer inter-burst intervals. The coefficient of variation of inter-burst intervals grew with the number of inhibitory neurons.

To model the observed effects, we developed a set of networks with various fractions of excitatory and inhibitory neurons. The networks were comprised of adaptive leaky integrate-and-fire neurons driven by slow Poisson input. The relative strength of inhibitory and excitatory synapses was kept at balance. The model showed that the stable mean but increasing variance of inter-burst intervals can be achieved by the balance of excitation and inhibition that regulates effects of the adaptation. In a fully excitatory network, inter-burst intervals are determined by the adaptation alone. Adding inhibition to the network results in stopping bursts before the adaptation completely silences the activity. This, in turn, allows the next burst to start earlier, leading to shorter inter-burst intervals with higher variance. To further compare the behavior of the model and cultures, we disrupted the excitation/inhibition balance by decreasing the strength of inhibitory synapses. In the experimental setup, this corresponded to the application of bicuculline. In the cultures with 10–80% of inhibitory neurons application of bicuculline led to prolonged interburst-intervals and decreased variability. Under maximum concentration, the activity of these cultures was generally similar to the fully excitatory cultures. Similarly, in the model, blocking of inhibition resulted in stronger adaptation after a burst that led to longer and less variable inter-burst intervals.

Overall, our results suggest that developed hippocampal cultures with artificial cellular excitatory and inhibitory composition tend to maintain the excitation/inhibition balance. This result in a constant mean activity but a growing variability of bursting in cultures with increasing numbers of inhibitory neurons.

**Fig. 1:**
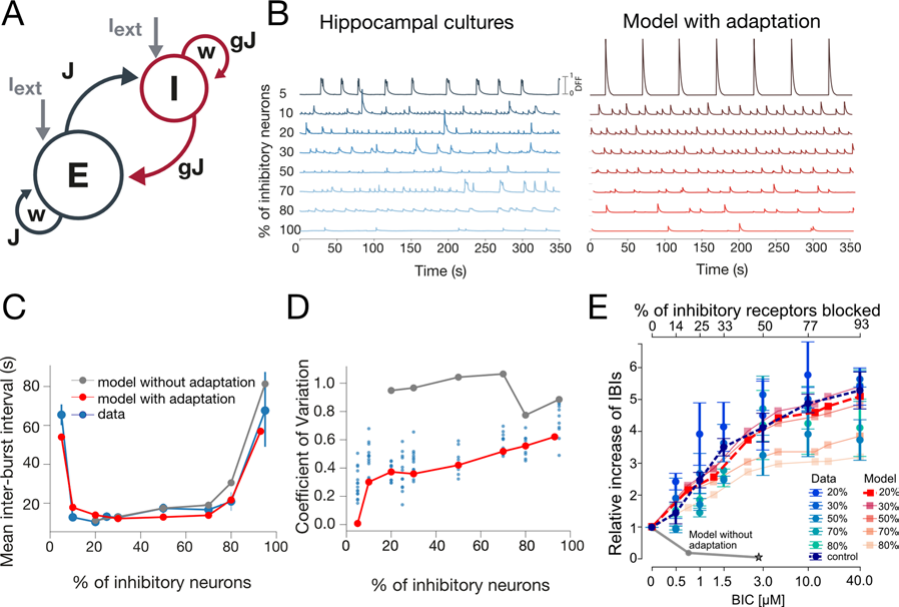
**a** Diagram of the model with adaptation. **b** Activity of hippocampal cultures and of the model with adaptation. **c** Changes in the mean inter-burst intervals with various fractions of inhibitory neurons. **d** Coefficient of variation of inter-burst intervals. **e** Effects of blocking inhibitory receptors

## P173 Towards a unified definition of the clustering coefficient for brain networks.

### Mehrdad Hasanpour^1^, Paolo Massobrio^2^, Anna Levina^3^

#### ^1^University of Tübingen, Institute for Physics, Tübingen, Germany; ^2^University of Genova, Department of Informatics, Bioengineering, Robotics, System Engineering (DIBRIS), Genova, Italy; ^3^Universtity of Tübingen, Tübingen, Germany

##### **Correspondence:** Mehrdad Hasanpour (mehrdad.hasanpour@tuebingen.mpg.de)

*BMC Neuroscience* 2019, **20(Suppl 1)**:P173

The brain is undoubtedly the most complex system known to humanity. Networks constitute the backbone of systems dynamics and there is a big arsenal of tools to describe relevant network properties. These tools can be employed to further study micro- and mesoscale organizations of the brain. Our goal here is to find out how different measures borrowed from graph theory influence structure inference in neuronal networks.It is widely expected that brain networks possess a small-world property, and that is important for fast and reliable information processing [1]. In order to calculate the small-worldness, we need two other measures: the characteristic path length and the clustering coefficient. In this study, we focus on the clustering coefficient, its different definitions, and their attributes. The local clustering coefficient of a node quantifies the tendency for edge formation between vertices connected to this node. For the case of undirected and unweighted networks, the definition of clustering coefficient is straightforward. However, the real cortical networks are both directed and weighted. To consider neuronal networks simply binary and undirected inevitably leads to misinterpretations of the network’s structure and, possibly, function. Multiple methods of computing a weighted clustering coefficient have been proposed, each capturing a particular aspect of the weighted network [2]. However, which of them is most suitable for brain networks is not established.In order to capture the differences among definitions, first, we go through simulated networks (random, small-world, and scale-free). We draw weights from a lognormal distribution that represents well the distribution of synaptic strength in a brain. We compute the weighted local clustering coefficient distributions for each definition. The difference between each method’s range and order of magnitude is shown in (Fig. 1, A). This perceptible diversity in ranges is rooted within either the slightly different property that it captures or its normalization method.The brain networks have a highly complex structure, that is not captured by the simple random networks we consider in theoretical studies. To account for it, we investigate functional networks extracted from the developed cultures using High-Density Multi-Electrode Array (HD-MEA). We pre-process the recordings using SpiCoDyn package and employ transfer entropy to capture information flow. We define functional connectivity by conventional thresholding the transfer entropy matrix at the level of different percentages of strongest connections. In the end, we compare different weighted clustering coefficient distributions (Fig. 1, B). Our result indicates the tangible difference in weighted clustering coefficient distributions which can noticeably influence inferred small-worldness. This also states the necessity of modifying a clustering coefficient appropriate for neuronal networks.We present a list of properties that a definition of clustering coefficient suitable for subsampled and arbitrarily thresholded neuronal network should satisfy. We discuss why the existing methods are not optimal for the task and how to modify them.

**Fig. 1:**
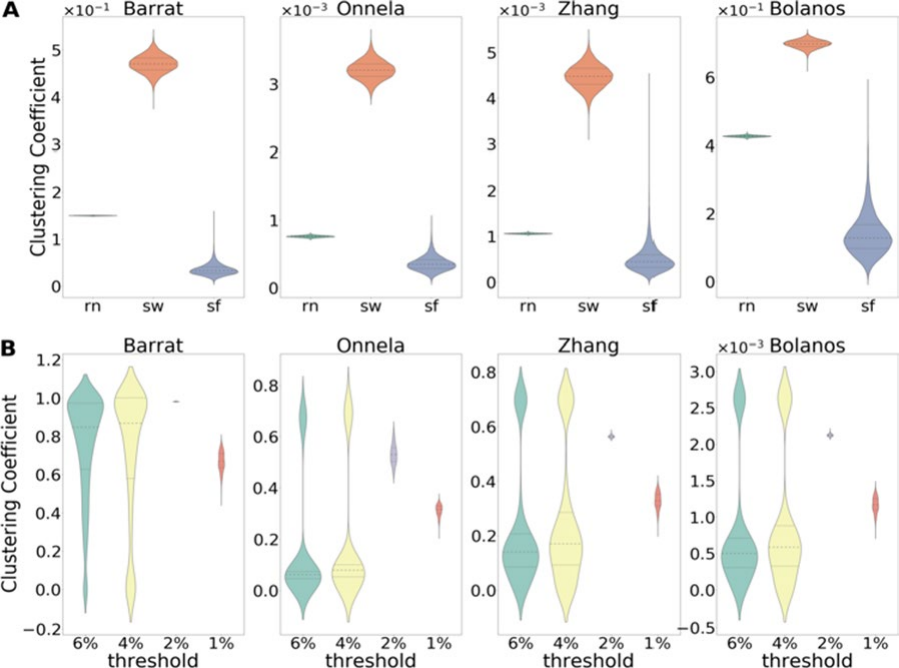
Different definitions result in extensively different values of clustering coefficients. **a** Weighted clustering coefficient computed for the simulated networks (rn: random, sw: small-world, sf: scale-free) of size 10000. **b** Weighted clustering coefficient computed for the extracted network of developed cortical culture. Quartile value and median are shown by the dashed lines in both panels

**References**

1. Bassett DS, Bullmore ET. Small-world brain networks revisited. *The Neuroscientist* 2017 Oct;23(5):499–516.
2. Opsahl T, Panzarasa P. Clustering in weighted networks. *Social networks* 2009 May 1;31(2):155–63.

## P174 Electric field effects improve resynchronization in the sparsely connected pacemaker nucleus of weakly electric fish.

### Aaron Regan Shifman, John Lewis

#### University of Ottawa, Department of Biology, Ottawa, Canada

##### **Correspondence:** Aaron Regan Shifman (ashif060@uottawa.ca)

*BMC Neuroscience* 2019, **20(Suppl 1)**:P174

The precise timing of neuronal activity is critical to normal brain function, be it for sound localization, escape responses, or plasticity and learning. The medullary pacemaker network (PN) of the wave-type weakly electric fish sets the timing for a high-frequency (~1000Hz) electric organ discharge (EOD) used in electric sensing. The PN is composed of ~150 cells which are sparsely coupled via gap junctions (~4% connectivity). Despite this weak coupling, the PN is the most precise biological oscillator known, with sub-microsecond period variation and highly synchronous activity across the network. Interestingly, oscillator precision and synchrony are under behavioural control: fish produce communication signals called “chirps” that involve a transient increase in PN frequency and a subsequent desynchronization of the PN. Previous work has suggested that the rapid recovery to a synchronized state after a chirp, and the high temporal precision of ongoing oscillations, is inconsistent with the sparse gap junctional connectivity of the PN. We hypothesize that electric field effects feeding back from the EOD underlie PN dynamics. We suggest that the bi-directional coupling between the EOD and the PN serves to stabilize oscillations and decrease resynchronization times after a perturbation from the synchronized state. As a first step towards testing this hypothesis, we first develop a new biophysical model that better-describes the dynamics of PN cells. We then construct networks using these model cells and show that while electric field feedback (from the EOD) produces only modest effects on oscillator precision, such feedback can significantly decrease network resynchronization times. This suggests that electric field effects may increase functional connectivity in some neuronal networks.

## P175 A self-consistent theory of autocorrelations in sparse networks of spiking neurons

### Sebastian Vellmer^1^, Benjamin Lindner^2^

#### ^1^Bernstein Center for Computational Neuroscience, Complex Systems and Neurophysics, Berlin, Germany; ^2^Humboldt University Berlin, Physics Department, Berlin, Germany

##### **Correspondence:** Sebastian Vellmer (sebastian.vellmer@bccn-berlin.de)

*BMC Neuroscience* 2019, **20(Suppl 1)**:P175

To study the dynamics of large and sparse recurrent networks of spiking neurons, Brunel [1] applied a stochastic mean field theory that considers a single representative neuron. Neural input, i.e. a large sum of independent spikes in the network, is approximated by temporally uncorrelated (white) Gaussian noise with a flat power spectrum. As a consequence, the problem can be formulated as a one-dimensional Fokker-Planck equation (FPE) with self-consistent coefficients; its stationary solution yields the firing rate. This theory is by construction only consistent with respect to the first-order statistics. Spike-trains of cortical neurons, however, exhibit nontrivial second-order statistics, i.e. non-flat power spectra [2] and the neural input maintains these temporal correlations [3]. Hence, the condition of self-consistence requires that input and output spike-train power spectra coincide. So far, this self-consistency condition has only been exploited in iterative schemes to determine these spectra numerically (e.g. in [4]). A complete self-consistent theory of spike-train correlations in a sparse network is still missing.

Here we present a theoretical framework for the temporal correlations of spike trains in networks of integrate-and-fire neurons. Neural input is approximated by a Markovian embedding, provided by the projection of an N-dimensional Ornstein-Uhlenbeck process. We can formulate a corresponding (N+1)-dimensional FPE, that describes the time evolution of a neuron ensemble driven by colored noise (open loop problem) and use it to derive a partial differential equation for the spike train power spectrum. The numerical solution of this equation displays strongly different spectral shapes depending on the color of the (prescribed) input noise.

In a second step, we can achieve self-consistence by solving equations for the output spectrum such that it coincides with the input noise spectrum. More specifically, the numerical solution of the equation for the power spectrum at selected frequencies in combination with a Pade expansion of the input spectrum is utilized to obtain the self-consistent solution. The results are presented in Fig. 1. For N = 0 (red curve) self-consistence is only achieved at the high-frequency limit as in [1]. For N = 1 it is achieved additionally at f = 0 and at the firing rate (thin gray line). For N = 2 we determined the solution that is self-consistent at f = ∞ and f = 0 and that minimizes the difference between input and output spectrum. Clearly, with increasing N, our self-consistent spike-train power spectrum approximates the network spectrum with increasing accuracy.

**Fig. 1:**
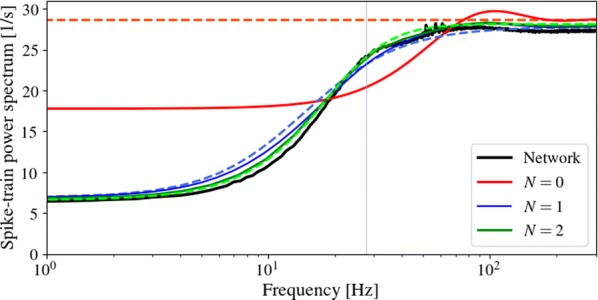
Self-consistent spike-train power spectrum from a network of LIF neurons with high synaptic weights and dominating inhibition (black line) and self-consistent solutions of N+1-dimensional FPEs (output solid, input dashed lines). We obtain a better approximation with increasing N

**Acknowledgements**: This work was developed within the scope of IRTG 1740/TRP 2015/50122-0, funded by DFG/FAPESP.

**References**

1. Brunel N. Dynamics of sparsely connected networks of excitatory and inhibitory spiking neurons. *Journal of Computational Neuroscience* 2000, 183–208.
2. Bair W, Koch C, Newsome W, et al. Power spectrum analysis of bursting cells in area MT in the behaving monkey. *Journal of Neuroscience* 1994, 2870–2892.
3. Lindner B. Superposition of many independent spike trains is generally not a Poisson process. *Physical Review E* 2006, 22901.
4. Dummer B, Wieland S, Lindner B. Self-consistent determination of the spike-train power spectrum in a neural network with sparse connectivity. *Frontiers in Computational Neuroscience* 2014, 104.

## P176 Stationary statistics and linear response of nonlinear Drift-Diffusion model across long sequences of trials

### Sebastian Vellmer^1^, Benjamin Lindner^2^

#### ^1^Bernstein Center for Computational Neuroscience, Complex Systems and Neurophysics, Berlin, Germany; ^2^Humboldt University Berlin, Physics Department, Berlin, Germany

##### **Correspondence:** Sebastian Vellmer (sebastian.vellmer@bccn-berlin.de)

*BMC Neuroscience* 2019, **20(Suppl 1)**:P176

To model decision making on a low level of cognition, the Drift-Diffusion model (DDM) was introduced in [1]. There, perceived evidence for a decision is modelled by a one-dimensional diffusion process and accumulated by an abstract variable x. When x exceeds the threshold xA or xB, the decision A or B is made, respectively. Afterwards the system is quiet for a time t0and, subsequently, the variable is reset to the value x0 [cf. Fig. 1a]. For a constant bias in x the diffusion process is analytically tractable using the Fokker-Planck equation (FPE). The model and slight variations were extensively used for data analysis and to gain insights in neuropsychological mechanisms. From another perspective neural networks were used as models of decision making. Biologically plausible networks are nonlinear. It was shown in [2] that the dynamics of nonlinear neural networks can be reduced to a nonlinear one-dimensional DDM. However, in contrast to the original model no analytic solution for the statistics of the nonlinear diffusion processes is known in general.

**Fig. 1:**
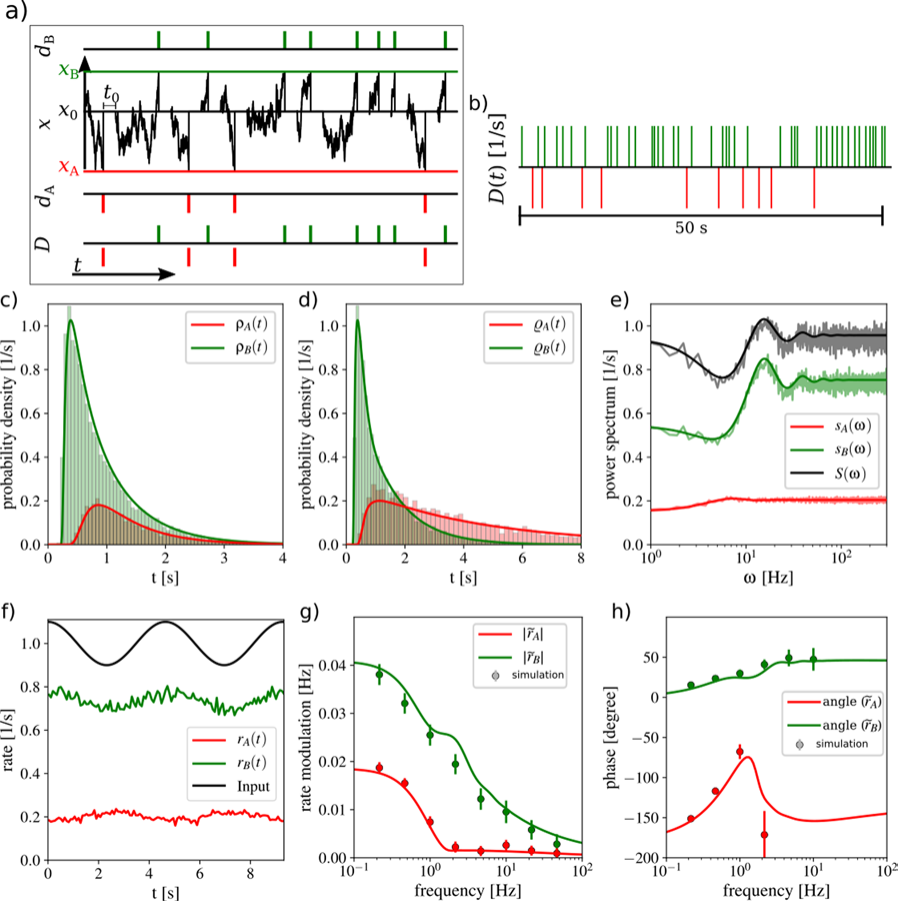
Nonlinear Drift-Diffusion model **a** and decision train for chosen parameters **b**. **c**: First passage time density and **d**: the inter-decision interval for same kind of decision. Solid lines show results of our method, histograms of simulations of the model. **e** Decision-train power spectra. **f** Modulated input and rate modulations. **g**, **h** Linear response theory (lines) and simulations (dots)

Here we present an effective numerical tool to calculate the statistics and the linear response to a weak sinusoidal input modulation of nonlinear DDMs, that is based on the method in [3]. The FPE and the continuity equation are written as two first-order differential equations and integrated numerically from the two thresholds.

We introduce the decision train, that marks decisions in a series of subsequent trials by a sum of delta functions at the decision times with negative (A decision) or positive signs (B decision); cf. Fig. 1b. The solution of the FPE yields the first-passage time densities for both thresholds (Fig. 1c), being the distribution of time intervals between sequential decisions, where the kind of the previous decision yields no relevance. Considering inter-decision intervals of only one decision type, the reset of the other decision has to be included in the FPE [Fig. 1d]. From these distributions, or directly from a corresponding FPE, we derived the decision-train power spectra in Fig. 1e.

Besides the stationary statistics, we use the threshold integration method to calculate the linear response to a sinusoidal modulated input (Fig. 1e). For weak stimuli the amplitude and phase of the decision rate modulation can be calculated (Fig. 1f and g). This might be an interesting tool for the analyzes of experiments in which decisions are influenced by known perturbations across trials.

The model without perturbation generates a renewal point process that cannot show correlations among decision times. However, in measurements these correlations were measured [4]. Future extensions of our model should incorporate mechanisms (slow adaptation variables, colored noise) that can explain such correlations.

**Acknowledgments:** This work was developed within the scope of IRTG 1740/TRP 2015/50122-0, funded by DFG/FAPESP.

**References**

1. Ratcliff R. A theory of memory retrieval. *Psychological review* 1978 Mar;85(2):59.
2. Roxin A, Ledberg A. Neurobiological models of two-choice decision making can be reduced to a one-dimensional nonlinear diffusion equation. *PLoS Computational Biology* 2008 Mar 28;4(3):e1000046.
3. Richardson MJ. Spike-train spectra and network response functions for non-linear integrate-and-fire neurons. *Biological Cybernetics* 2008 Nov 1;99(4–5):381-92.
4. Fründ I, Wichmann FA, Macke JH. Quantifying the effect of intertrial dependence on perceptual decisions. *Journal of vision* 2014 Jun 1;14(7):9-.

## P177 Model order reduction of multiscale models in neuroscience

### Ippa Seppälä, Mikko Lehtimäki, Lassi Paunonen, Marja-Leena Linne

#### Tampere University, Tampere, Finland

##### **Correspondence:** Ippa Seppälä (iseppaelae@gmail.com)

*BMC Neuroscience* 2019, **20(Suppl 1)**:P177

The current trend in computational neuroscience is to incorporate multiple physical levels of the brain into mathematical models, which often results in large networks of interconnected neural cells. Comprehensive models with accurate system dynamics are necessary in order to increase understanding of different mechanisms in the whole brain, but these models are analytically intractable. Additionally, their numerical simulation is very resource intensive. Useful ways of mitigating the computational burden include using a mean-field approach, as well as mathematical model order reduction (MOR).

Using mean-field approximation, one can account for the random fluctuations of variables by replacing them by their mean averages. The cells are grouped together into populations based on their statistical similarities, in order to represent the dynamics of the system in terms of the averaged out ensemble behaviour. These populations can then be described by a probability density function expressing the distribution of neuronal states at a given time. This approach ensures that the essential system dynamics converge to a stationary attractor consistent with the steady-state dynamics of the original system. Here we use the Fokker-Planck formalism, which results in a nonlinear system of partial differential equations (PDEs).

PDE systems can be difficult to solve analytically, and thus discretisation for numerical analysis is necessary. This discretisation often leads to very high-dimensional numerical models that correspond to equally high computational demand. Discretised PDE systems can be reduced using mathematical model order reduction methods [1]. MOR methods are well established in engineering sciences, such as control theory, as they improve computational efficiency of simulations of large-scale nonlinear mathematical models. In computational neuroscience MOR is underutilised, although the potential benefits in enabling multilevel simulations are obvious [2].

In this study we use mathematical MOR methods to reduce the dimensions of a PDE model derived using the mean-field approach. The system can be reduced with minimal information loss, by deriving a subspace that approximates the entire system and its dynamics with a smaller number of dimensions compared to the original model. Here we use Proper Orthogonal Decomposition with Discrete Empirical Interpolation Method (POD+DEIM), a subspace projection method for reducing the dimensionality of general nonlinear systems [1]. By applying these methods, the simulation time of the model is radically shortened, albeit not without dimension-dependent approximation error. The tolerated amount of inaccuracy depends on the final application of the model.

Due to being well-suited for depicting mesoscopic behaviour, the mean-field approach in combination with the POD+DEIM method allows us to describe the behaviour of any large multiscale brain model with a relatively low computational burden. This can be particularly useful when attempting to model whole-brain connectivity, for which there is an immediate demand in clinical and robotic applications.

**References**

1. Chaturantabut S, Sorensen, DC. Nonlinear model reduction via discrete empirical interpolation. *SIAM Journal on Scientific Computing* 2010, 32(5), pp.2737–2764.
2. Lehtimäki M, Paunonen L, Pohjolainen S, Linne ML. Order reduction for a signaling pathway model of neuronal synaptic plasticity. *IFAC Papers OnLine* 2017, 50-1:7687–7692.

## P178 Modeling the influence of neuron-astrocyte interactions on signal transmission in neuronal networks

### Jugoslava Acimovic^1^, Tiina Manninen^1^, Heidi Teppola^1^, Sacha van Albada^2^, Markus Diesmann^2^, Marja-Leena Linne^3^

#### ^1^Tampere University, Faculty of Medicine and Health Technology, Tampere, Finland; ^2^Jülich Research Centre, Institute of Neuroscience and Medicine (INM-6) and Institute for Advanced Simulation (IAS-6), Jülich, Germany; ^3^Tampere University, Tampere, Finland

##### **Correspondence:** Jugoslava Acimovic (jugoslava.acimovic@gmail.com)

*BMC Neuroscience* 2019, **20(Suppl 1)**:P178

Understanding the influence of glial cells on brain functions is of fundamental interest for neuroscience and theoretical neuroscience. We focus on one type of glial cells, the astrocyte, that has been found to contribute to signal transmission in neural networks. Astrocytes are a non-homogeneous cell type whose structural and functional properties vary across different brain areas. A number of computational efforts have been presented to explore selected biophysical mechanisms and their roles in various neurophysiological phenomena [1,2]. Models focusing on astrocytic regulation of synaptic transmission are however scarce. Further efforts are needed to extend these models with other relevant biophysical mechanisms. In addition, simple yet dynamically correct models suitable for addressing the role of neuron-astrocyte interactions in larger systems, neural networks and brain circuits, are rare. While the experimental evidence of astrocytes’ roles in neural activity regulation accumulate, adequate network-level models could help to explore the role of neuron-astrocyte interactions in cognition.

Here we focus on one specific mechanism of neuron-astrocyte regulation that, according to the experimental evidence, supports network synchrony. In [3], glutamate released from astrocytes has been shown to activate extrasynaptic N-methyl-D-aspartate receptors (NMDARs) in neighboring neurons, causing slow inward currents and increased capacity for synchronization at the circuit level. This experimental finding is first explored in combination with our previous work [4] that focused on impact of ionotropic glutamatergic and GABAergic receptors to synaptic transmission and activity in cortical networks. Using a data-driven modeling framework, we integrated the model with the experimental data and quantified accuracy of data representation. Here, we extend this previous work by equipping the model with the minimal description for the extrasynaptic NMDARs and the related cellular and synaptic mechanisms.

The results obtained from this initial model will be further used as a guideline to extend a well-established theoretical model [5] with the same mechanism of neuron-astrocyte interactions, and to test how this mechanism contributes to dynamical regimes in a model incorporating the realistic size and organization of cortical circuits.

The proposed work provides a step towards developing of theoretical methods for describing neuron-astrocyte interactions and more generally glial contributions in activity regulation, information transfer, synchronization, and learning in neuron-glia circuits. Furthermore, it will establish guiding principles for implementation of simplified, generic astrocyte models in neuromorphic technologies for engineering applications.

**Acknowledgements:** The work is supported by Human Brain Project (785907) and Academy of Finland (297893, 315795, 320072).

**References**

1. Manninen T, Havela R, Linne ML. Computational models for calcium-mediated astrocyte functions. *Frontiers in computational neuroscience* 2018 Apr 4;12:14.
2. Manninen T, Havela R, Linne ML. Computational Models of Astrocytes and Astrocyte–Neuron Interactions: Characterization, Reproducibility, and Future Perspectives. *In Computational Glioscience* 2019 (pp. 423–454). Springer, Cham.
3. Pirttimaki TM, Sims RE, Saunders G, Antonio SA, Codadu NK, Parri HR. Astrocyte-mediated neuronal synchronization properties revealed by false gliotransmitter release. *Journal of Neuroscience* 2017 Oct 11;37(41):9859-70.
4. Acimovic J, Teppola H, Mäki-Marttunen TM, Linne M-L. Data-driven study of synchronous population activity in generic spiking neuronal networks: How much do we capture using the minimal model for the considered phenomena? *BMC Neuroscience* 2018 Oct 29;19(Suppl 2):68–69.
5. Potjans TC, Diesmann M. The cell-type specific cortical microcircuit: relating structure and activity in a full-scale spiking network model. *Cerebral cortex* 2012 Dec 2;24(3):785–806.

## P179 Topological analysis of LFP data

### Leonid Fedorov^1^, Tjeerd Dijkstra^2^, Yusuke Murayama^1^, Christoph Bohle^3^, Nikos Logothetis^1^

#### ^1^Max Planck Institute for Biological Cybernetics, Department Physiology of Cognitive Processes, Tübingen, Germany; ^2^Center for Integrative Neuroscience, Hertie Institute, Department Cognitive Neurology, Tübingen, Germany; ^3^University of Tübingen, Mathematisch-Naturwissenschaftliche Fakultät, Tübingen, Germany

##### **Correspondence:** Leonid Fedorov (leonidf87@gmail.com)

*BMC Neuroscience* 2019, **20(Suppl 1)**:P179

The Local Field Potential (LFP) summarizes synaptic and somato-dendritic currents in a bounded ball around the electrode and is dependent on the spatial distribution of neurons. Both fine-grained properties and the temporal distribution of typical waveforms in spontaneous LFP have been used to identify global brain states (see e.g. [1] for P-waves in stages of sleep). While some LFP signatures have been studied in detail (in addition to Pons, see e.g. sleep spindles in the Thalamus and areas of the cortex [2], sharp-wave-ripples [3] in the Hippocampus and k-complexes [4]), it stands to understand the relationship between simultaneous signaling in cortical and subcortical areas. To characterize the mesoscale spontaneous activity, we quantify data-driven properties of LFP and use them to describe different brain states. Inspired by [5], we treat frequency-localized temporary increases in LFP power simultaneously recorded from Cortex, Hippocampus, Pons and LGN as Neural Events that carry information about the brain state. Here, we give a fine-grained characterization of events in the 0-60Hz frequency range that differentiates the onset and offset intervals from the ongoing short-term oscillation within the event’s duration. For example, a fixed-amplitude oscillatory interval can be conceptually thought of as a temporally resolved sample from a circle, whereas the onset and offset can be regarded as samples from spirals. Thus, the change within an event corresponds to a topological change of the trajectory in phase space. We use topological data analysis to detect this change in topology. In detail, we look at barcodes computed using persistence homology [6] of the delay embedding [7, 8] of consecutive windows within a neural event. A persistence barcode can be seen as a topological signature [9] of the reconstructed trajectory. We rely on the difference between a circle and a spiral in homology when this qualitative change is inferred from looking at consecutive barcodes. This feature (Fig. 1) describes the onset-duration-offset intervals for each oscillation, yet is agnostic to event type, recording site or brain state.

**Fig. 1:**
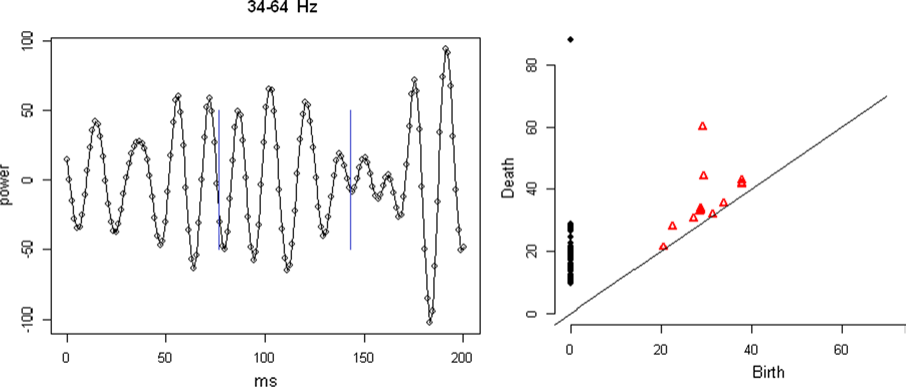
Left panel: example neural event localized to 34-64Hz frequency range from a hippocampal site and, highlighted in blue—an interval within it. Right panel: persistence diagram of the point cloud obtained via delay-embedding of that interval; black dots correspond to birth and death times of zeroth homology groups, and red triangles—of first homology groups (cycles in data)

**References**

1. Gott JA, Liley DTJ, Hobson AJ. Towards a Functional Understanding of PGO Waves. *Frontiers in Human Neuroscience* 2017, 11–89.
2. Contreras D et al. Spatiotemporal patterns of spindle oscillations in cortex and thalamus. *Journal of Neuroscience* 1997, 17:1179–96.
3. Buzsáki G. Hippocampal sharp wave-ripple: A cognitive biomarker for episodic memory and planning. *Hippocampus* 2015, 25, 1073–188.
4. Amzica F, Steriade M. Cellular substrates and laminar profile of sleep K-complex. *Neuroscience* 1997, 82, 671–686.
5. Logothetis NK, et al. Hippocampal–cortical interaction during periods of subcortical silence. *Nature* 2012, 491, 547–553.
6. Edelsbrunner H, Letscher D, Zomorodian A. Topological persistence and simplification. I*n Proceedings 41st Annual Symposium on Foundations of Computer Science* 2000 (pp. 454–463). IEEE.
7. Perea JA, Harer J. Sliding windows and persistence: An application of topological methods to signal analysis. *Foundations of Computational Mathematics* 2015, 15, 799–838.
8. Sanderson N, et al. Computational Topology Techniques for Characterizing Time-Series Data. *In International Symposium on Intelligent Data Analysis* 2017 Oct 26 (pp. 284–296). Springer.
9. Chazal F, Michel B. An introduction to Topological Data Analysis: fundamental and practical aspects for data scientists. 2017, arXiv:1710.04019v1

## P180 Mechanisms of stimulus-induced broadband Gamma Oscillations in a stochastic Wilson-Cowan model

### Arthur Powanwe, Andre Longtin

#### University of Ottawa, Department of Physics and Centre for Neural Dynamics, Ottawa, Canada

##### **Correspondence:** Arthur Powanwe (apowa074@uottawa.ca)

*BMC Neuroscience* 2019, **20(Suppl 1)**:P180

Stimuli can induce gamma oscillations in primary visual cortex (V1) of monkeys [1]. The resulting oscillations appear as discrete events with short durations (gamma bursts) . A single recording does not easily link stimulus properties to the induced LFP. However, trial averaged recordings capture well the stimulus-induced gamma oscillations by means of a transient epoch of approximately 100 ms after the stimulus onset where the power of gamma oscillations is high and the peak frequency spans a large band [1]. Trial averaged analysis also shows how the stimulus properties like contrast or orientation tuning shape the resulting oscillations [2].

The goal of this study is to propose a robust mechanism of stimulus-induced gamma oscillations. Specifically we are interested in a model with the ability to explain the sharp increase in the power and the corresponding large frequency band observed in the trial averaged spectrogram immediately after the stimulus onset. It should also explain the increase in the peak frequency as the stimulus contrast increases and the modulation of the peak frequency with the time-varying contrast as observed in vivo [2].

We address this issue from a nonlinear dynamical point of view. We consider a stochastic spiking network [3], whose corresponding mean field description leads to the stochastic Wilson-Cowan model. Linear stability analysis with the external currents (impinging on the excitatory and inhibitory populations respectively) shows many stable domains, namely the asynchronous state (real negative eigenvalues), the transient synchrony (complex conjugate eigenvalues with negative real parts) and the high synchrony (complex conjugate eigenvalues with positive real parts). When there is no contrast, the system is in the asynchronous state and there is no oscillation. Increasing the contrast leads to a transition from the asynchronous state to the transient synchrony state. At the transition between the two domains, oscillations appear, and the peak frequency increases upon entering the transient synchrony domain. This explains the increase of the peak frequency as the contrast increases. The modulation of the oscillations with the stimulus contrast is also observed in the transient synchrony regime. Finally, the transient amplification observed in the spectrogram can be explained by a strong non-normal amplification [4] of the network when the system enters the transient synchrony state from the asynchronous state. These mechanisms can also be implemented in a similar network with a ring architecture.

**References**

1. Xing D, Shen Y, Burns S, Yeh CI, Shapley R, Li W. Stochastic generation of gamma-band activity in primary visual cortex of awake and anesthetized monkeys. *Journal of Neuroscience* 2012 Oct 3;32(40):13873–80a.
2. Ray S, Maunsell JH. Differences in gamma frequencies across visual cortex restrict their possible use in computation. *Neuron* 2010 Sep 9;67(5):885–96.
3. Wallace E, Benayoun M, Van Drongelen W, Cowan JD. Emergent oscillations in networks of stochastic spiking neurons. *PLoS One* 2011 May 6;6(5):e14804.
4. Henrici P. Bounds for iterates, inverses, spectral variation and fields of values of non-normal matrices. *Numerische Mathematik* 1962 Dec 31;4(1):24–40.

## P181 Phase synchronization and information transfer between coupled bursty-oscillatory neural networks in the gamma band.

### Arthur Powanwe, Andre Longtin

#### University of Ottawa, Department of Physics and Centre for Neural Dynamics, Ottawa, Canada

##### **Correspondence:** Arthur Powanwe (apowa074@uottawa.ca)

*BMC Neuroscience* 2019, **20(Suppl 1)**:P181

Oscillations in the gamma-band frequency (30-90) Hz are ubiquitous in the brain and are believed to be useful for perceptual and cognitive behaviour, coding properties or communication between brain areas [1]. To be a good candidate for these tasks gamma oscillations need to show sufficient coherence in their amplitude, phase and peak frequency. However, usually recorded LFP or EEG in vivo show oscillations at gamma band frequency which exhibit epochs of high amplitude (gamma bursts) alternating with epochs of low amplitude, random phase dynamics and highly variable peak frequency. The incidences and durations of gamma bursts are stochastic and the peak frequency inside bursts also shows high variability.

Despite this stochastic behaviour, a recent computational work has showed that such gamma bursts can efficiently transmit information between brain areas [2]. Our work has showed that the dynamic and statistics of such gamma band activity can be efficiently described by simple nonlinear coupled stochastic differential equations representing the amplitude and phase dynamics of the oscillations [3]. However, the network model used to derive equations for the amplitude and phase dynamics in this study is a simple stochastic Wilson-Cowan model, without any level of complexity (no propagation delays, no heterogeneity,…). Further, the network model in [2] uses a more detailed single neuron model (Wang-Buzsaki) and high level of complexity (delays propagation, synaptic heterogeneity, sparseness,…). A central question then appears since both network types generate gamma band activity similar to what is recorded in vivo. Is flexible communication between brain areas with gamma bursts just due to the oscillation generation mechanism which is similar for both networks type (The network needs to be noisy and to work at the onset of oscillatory synchrony)? Or it is due to a more complex phenomenon due to the high complexity of the neural network?

To answer this question, we first extend our previous method to derive coupled phase-amplitude equations for a single network [3] to two E-I networks coupled by long range excitatory (i.e. E-to-E) connections with inter-areal propagation delay. We focus here on phase synchronization between two coupled networks and information transfer between them. We used a recent method based on Phase Transfer Entropy (PTE) [4] which only requires the phase dynamics we have derived for the two networks. This allows us to infer the amount of information transmitted between coupled networks and its directionality. Results show phase synchronization and the presence of two routing states for information transfer, similar to what has been showed in the detailed computational study [2]. However, the location of the routing states and efficiency of phase synchronization depend respectively on delay propagation and the noise level and coupling strength. This suggests that the higher network complexity is not necessary; all that is required for flexible information transfer is operation near the onset of synchrony in the presence of generic additive noise on the rate equations

**References**

1. Buzsaki G. Rhythms of the Brain. *Oxford University Press*; 2006 Aug 3.
2. Palmigiano A, Geisel T, Wolf F, Battaglia D. Flexible information routing by transient synchrony. *Nature neuroscience* 2017 Jul;20(7):1014.
3. Powanwe A, et al. *In preparation.*
4. Lobier M, Siebenhühner F, Palva S, Palva JM. Phase transfer entropy: a novel phase-based measure for directed connectivity in networks coupled by oscillatory interactions. *Neuroimage* 2014 Jan 15;85:853–72.

## P182 Data-driven jump-diffusion modelling with application to electric fish

### Alexandre Melanson^1^, Andre Longtin^2^

#### ^1^University of Moncton, Physique et Astronomie, Moncton, Canada; ^2^University of Ottawa, Department of Physics and Centre for Neural Dynamics, Ottawa, Canada

##### **Correspondence:** Alexandre Melanson (amela093@uottawa.ca)

*BMC Neuroscience* 2019, **20(Suppl 1)**:P182

The emergent activity of biological systems, such as neural networks or ion channel assemblies, can often be represented as low-dimensional, Langevin-type stochastic differential equations. Reconstructing these equations from experimental time series is possible by estimating the coefficients of the associated Fokker-Planck equation. In certain systems, however, large and abrupt events can occur and violate the assumptions of this Langevin approach. We address this situation here by providing a novel method that reconstructs a jump-diffusion stochastic differential equation based solely on a realization of the original process. We use threshold-crossing of the increments to detect jumps in the time series. This is followed by an iterative scheme that compensates for the presence of diffusive fluctuations that are falsely detected as jumps. Our approach is based on probabilistic calculations associated with these fluctuations, and on the use of the Fokker-Planck and the differential Chapman-Kolmogorov equations.

We show that this inference procedure can be successfully applied to two unrelated types of data from pulse-type electric fish: electrophysiological time series of membrane noise in pyramidal neurons of the electrosensory line lobe, and recordings of the electric organ discharge rate during rest and exploratory locomotion. In this first case, we show that membrane potential fluctuations display large, jump-like depolarization events that occur at random times, the biophysics of which is unknown. After applying our inference procedure to these data, we find that some pyramidal cells increase their jump rate and noise intensity as the membrane potential approaches spike threshold, while their drift function and jump amplitude distribution remain unchanged. As for the second case, previous studies have demonstrated that fish exhibit abrupt increases in their electrosensory sampling rate. By reconstructing a jump-diffusion process from these data, we show the abrupt events occur more frequently during rest than during exploratory locomotion. We also show that the remaining fluctuations in electrosensory sampling rate evolve in a wider potential well during movement than during rest. We conclude that our inference approach is applicable to a variety of situations where abrupt events occur among diffusive fluctuations, and that it provides a means to investigate the functional role of these events without relying on poorly understood biophysical or neural mechanisms.

## P183 Alcohol influence on fear conditioning and extinction in a new amygdala model

### Alexey Kuznetsov^1^, Adam Lonnberg^2^

#### ^1^IUPUI, Indianapolis, IN, United States of America; ^2^University of Evansville, Department of Mathematics, Evansville, United States of America

##### **Correspondence:** Alexey Kuznetsov (alexey@math.iupui.edu)

*BMC Neuroscience* 2019, **20(Suppl 1)**:P183

The processes of fear conditioning and extinction are dependent on the amygdala circuitry [1, 2], and the effects of alcohol on these processes are extensively documented [3, 4]. However, the connections between the changes in amygdala structure and function induced by alcohol and fear conditioning are not well established. We introduce a computational model to test the mechanistic relationship between amygdala functional and structural adaptation during fear learning and the impact of acute vs. repeated alcohol. We hypothesize that acute and prior repeated alcohol exposure impedes fear extinction.

We use firing rate formalism to model a total of five neuronal populations following earlier models [5] with modifications that improve robustness. The model includes the lateral nucleus (LA) of the amygdala, which is crucial to fear acquisition, the basal nucleus (BA) and the lateral subdivision of the central nucleus (CeL). Further, BA and CeL are subdivided into two groups responsible for fear expression and extinction (BAf/BAe; CeLOn/CeLOff). Learning in the model is mediated by the potentiation of inputs to LA and BAf from the thalamus and hippocampus respectively (for acquisition) and to BAe from the prefrontal cortex (for extinction). The behavioral output of the model is activity of the CeLOn neurons, which underlies fear-related behaviors. We calibrate the model to reproduce changes in amygdala connectivity in acute and after prior repeated alcohol exposure measured in vitro [6, 7] and connect this data with in vivo alterations in fear behavior and learning [3, 4].

We determine that, in accordance with experiments [6, 7], alcohol disrupts fear extinction greater than fear conditioning. In conditioning, however, the model predicts that both acute and prior repeated alcohol exposure changes the contribution of context and stimuli (Fig. 1A BAf activity above LA in both alcohol cases). While this does not affect the speed of fear conditioning, both acute and prior repeated alcohol negatively affect speed and robustness of fear extinction in simulations (Fig. 1). The model predicts that the mechanism for this negative effect is related to the above restructuring of amygdala activity that changes the contributions of context and stimuli during conditioning. Furthermore, our results predict that alcohol, especially its prior repeated exposure, move the system to the threshold for spontaneous renewal (relapse) of fear expression. Indeed, in our simulations, both acute and prior repeated alcohol lead to greater activation of the fear pathway after conditioning and shifts the system from robust extinction to relapses. (Fig. 1). Thus, we show how structural changes induced by alcohol in amygdala may affect fear behaviors.

**Fig. 1:**
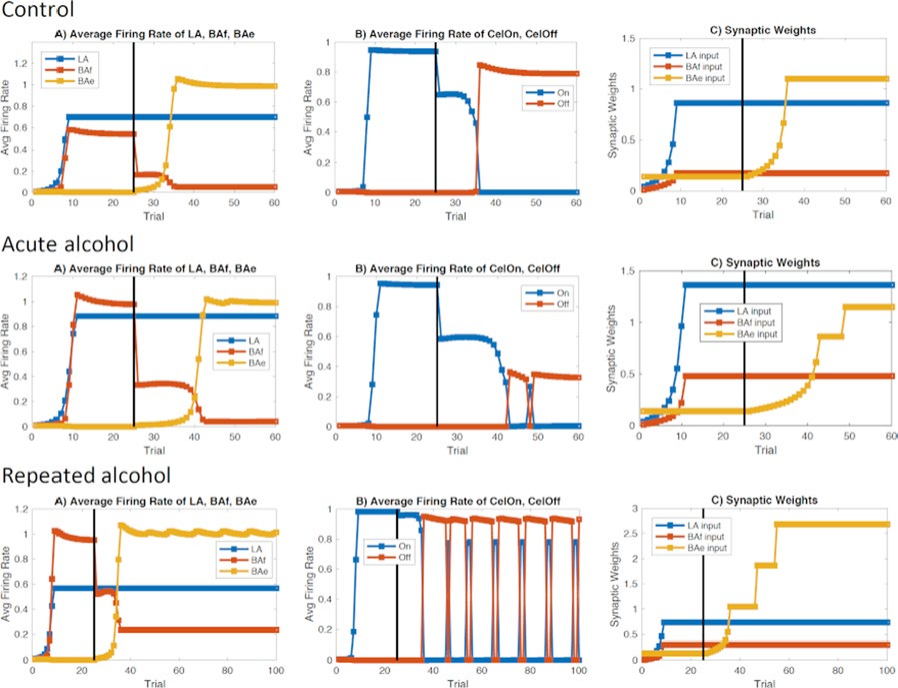
Dynamics of amygdala activity and synaptic inputs during conditioning and extinction in control (top), acute alcohol (middle) and prior repeated alcohol (bottom) conditions. LA = Lateral Amygdala; BAf/BAe = fear/extinction-activated Basal Amygdala; CeL On/Off = fear/extinction-related Central amygdala nucleus. High activity of CeL On signifies behavioral fear response

**References**

1. Likhtik E, Popa D, Apergis-Schoute J, Fidacaro GA, Paré D. Amygdala intercalated neurons are required for expression of fear extinction. *Nature* 2008 Jul;454(7204):642.
2. Walker DL, Ressler KJ, Lu KT, Davis M. Facilitation of conditioned fear extinction by systemic administration or intra-amygdala infusions of D-cycloserine as assessed with fear-potentiated startle in rats. *Journal of Neuroscience* 2002 Mar 15;22(6):2343–51.
3. Gilpin NW, Herman MA, Roberto M. The central amygdala as an integrative hub for anxiety and alcohol use disorders. *Biological psychiatry* 2015 May 15;77(10):859–69.
4. Roberto M, Gilpin NW, Siggins GR. The central amygdala and alcohol: role of γ-aminobutyric acid, glutamate, and neuropeptides. *Cold Spring Harbor perspectives in medicine* 2012 Dec 1;2(12):a012195.
5. Carrere M, Alexandre F. A Pavlovian model of the amygdala and its influence within the medial temporal lobe. *Frontiers in systems neuroscience* 2015 Mar 18;9:41.
6. Bisby JA, King JA, Sulpizio V, Degeilh F, Curran HV, Burgess N. Extinction learning is slower, weaker and less context specific after alcohol. *Neurobiology of learning and memory* 2015 Nov 1;125:55–62.
7. Lattal KM. Effects of ethanol on encoding, consolidation, and expression of extinction following contextual fear conditioning. *Behavioral Neuroscience* 2007 Dec;121(6):1280.

## P184 Whole-brain network modelling of psilocybin treatment for depression

### Jakub Vohryzek^1^, Robin Carhart-Harris^2^, Gustavo Deco^3^, Morten Kringelbach^4^, Joana Cabral^5^, Louis-David Lord^4^

#### ^1^University of Oxford, Hedonia Group, Oxford, Czechia; ^2^Imperial College London, Psychedelic Research Group, London, United Kingdom; ^3^Universitat Pompeu Fabra, Barcelona, Spain; ^4^University of Oxford, Department of Psychiatry, Oxford, United Kingdom; ^5^University of Minho, Life and Health Sciences Research Institute, Braga, Portugal

##### **Correspondence:** Jakub Vohryzek (jakub.vohryzek@queens.ox.ac.uk)

*BMC Neuroscience* 2019, **20(Suppl 1)**:P184

Despite showing significant promise for treating patients with neuropsychiatric conditions, the underlying mechanisms of psychedelics have remained elusive. However, recently new light has been shed on the causal mechanisms of how psychedelics work through the 5HT-2A receptors of the serotonergic system in the brain [3]. Treatment-resistant depression has been treated with psilocybin—an active component of magic mushrooms—and shown promising outcomes [2]. Here, we analysed the neuroimaging data (fMRI) from this pilot study comprising 15 patients with treatment-resistant depression grouped into 7 responders and 8 non-responders based on their QIDS depressive score 5 weeks after the treatment [2]. We firstly explore the dynamical landscape of the brains of patients using leading eigenvector dynamics analysis (LEiDA) [1] (Fig. 1A, B). We further model the patients’ pre- and post-psilocybin intervention brain states using super-critical Hopf bifurcation model connected in a network defined by a group structural connectome. Subsequently, by perturbing the model, we identify possible brain regions and mechanisms leading to the treatment outcomes between groups (Fig. 1C). Our experimental analysis shows the Functional Connectivity Dynamics (FCD) histograms to be significantly different between pre- and post-treatment conditions and between responders and non-responders post-treatment conditions. Clustering the leading eigenvectors of BOLD phase coherence, we identified k = 3 recurrent patterns, with k chosen by the most appropriate combination of Davies–Bouldin score and Silhouette criterion. We showed a significant difference between the conditions for responders in the Probabilistic State Space (PMS) of state three. Furthermore, we constructed and validated the Hopf model to both FCD histograms and PMS for the pre-psilocybin condition for both the responders and non-responders. Through a principled perturbation of the expected psilocybin effects on the brain dynamics we were able to predict the significant functional differences between the responder and non-responder groups. In conclusion, we demonstrate that an increased difference in the FCD as well as the PMS of the third functional state are suggestive of the re-organisation of the brain dynamics for the two-groups of responders/non-responders between the pre- and post-conditions. Moreover, the ability to mechanistically explore the changes in brain dynamics between the conditions through the deployment of a whole-brain model leads to a systematic prediction of the successful treatment between the two groups. In terms of long-term goals, this approach has a potential to address the appropriate dynamical transition of the brain and thus tailor a specific treatment to fit an individual patient’s need.

**Fig. 1:**
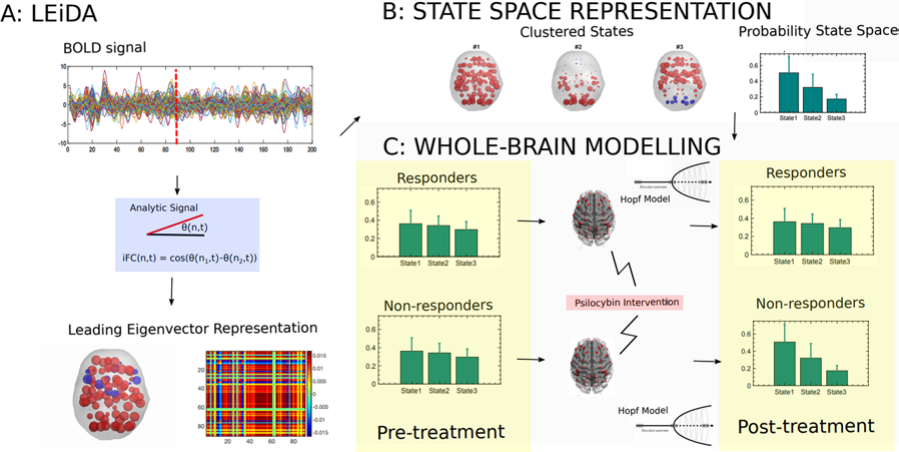
Study Overview: **a** Leading Eigenvector Dynamics Analysis (LEiDA) of the fMRI dataset. **b** State-space representation for three cluster solution of the k-means algorithm. **c** Super-critical Hopf network model fitted to the pre-treatment and perturbed according to psilocybin neuropharmacology to predict the post-treatment results

**References**

1. Cabral J, Vidaurre D, Marques P, et al. Cognitive performance in healthy older adults relates to spontaneous switching between states of functional connectivity during rest. *Scientific reports* 2017 Jul 11;7(1):5135.
2. Carhart-Harris RL, Roseman L, Bolstridge M, et al. Psilocybin for treatment-resistant depression: fMRI-measured brain mechanisms. *Scientific reports* 2017 Oct 13;7(1):13187.
3. Deco G, Cruzat J, Cabral J, et al. Whole-brain multimodal neuroimaging model using serotonin receptor maps explains non-linear functional effects of LSD. *Current biology* 2018 Oct 8;28(19):3065–74.

## P185 Thalamo-cortical microcircuit model of β-rhythm generation in Parkinson’s disease and attenuation during deep brain stimulation

### AmirAli Farokhniaee, John Fleming, Karthik Sridhar, Madeleine Lowery

#### University College Dublin, Neuromuscular Systems Lab, School of Electrical & Electronic Engineering, Dublin, Ireland

##### **Correspondence:** AmirAli Farokhniaee (aafarokh@gmail.com)

*BMC Neuroscience* 2019, **20(Suppl 1)**:P185

Information flow within the cortical areas responsible for motor planning and motor execution is disrupted in Parkinson’s disease (PD). This disruption and information loss is associated with exaggeration of beta oscillations (~13-30 Hz) observed in the LFP and ECoG recordings from the cortex. The underlying mechanism of the generation of these enhanced beta rhythms is still in debate and remains unclear. Recent studies hypothesize that alterations in synaptic connections both within and between the cortex and thalamus play a critical role in the generation of pathological beta rhythms in PD. To examine this hypothesis, we developed a spiking neuronal network model of the thalamo-cortex, the thalamo-cortical microcircuit (TCM). The TCM contains reciprocal synaptic connections that generate low frequency oscillations in the microcircuit in healthy conditions. Alterations of specific connections is shown to lead to high beta rhythm within the TCM. The model was compared and validated against neural firing patterns recorded in rodent models of PD from the literature. The TCM model was then used to investigate the effects of deep brain stimulation (DBS) on exaggerated beta oscillations in the cortical network. The beta rhythm within the TCM was attenuated by the application of monophasic DBS pulses that travel antidromically to the deep layer of cortex. The TCM model provides several advantages over mean field and neural mass models. It enables us to examine individual neural spiking patterns and synchronization within and between the populations under study, both with and without stimulation. In addition, it enables short term synaptic plasticity to be incorporated. Finally, the model presented provides with a biophysical pathway for integration of results from different studies and sources, to better understand changes in cortical information flow in PD.

## P186 Phase-amplitude coupled oscillations and information flow in a multiscale model of M1 microcircuits

### Salvador Dura-Bernal^1^, Benjamin A Suter^2^, Samuel A Neymotin^3^, Gordon MG Shepherd^4^, William W Lytton^1^

#### ^1^SUNY Downstate Medical Center, Department of Physiology and Pharmacology, Brooklyn, NY, United States of America; ^2^Institute of Science and Technology (IST), Austria; ^3^Nathan Kline Institute for Psychiatric Research, United States of America; ^4^Northwestern University, United States of America

##### **Correspondence:** Salvador Dura-Bernal (salvadordura@gmail.com)

*BMC Neuroscience* 2019, **20(Suppl 1)**:P186

We developed a model of primary motor cortex (M1) microcircuits [1] with over 10,000 biophysically detailed neurons and 30 million synaptic connections. It simulates a cylindric cortical volume with a depth of 1350 μm and a diameter of 300 μm. Neuron densities, classes, morphology and biophysics, and connectivity at the long-range, local and dendritic scale were derived from experimental data published in over 30 studies. The model was developed using the NetPyNE tool [2], which facilitated the integration of this complex experimental data at multiple scales. Our model exhibited spontaneous neural activity patterns and oscillations consistent with M1 data. Neural activity depended on cell class, cortical layer and sublaminar location. Different output dynamics were seen when the network was driven by brief activation of particular long-range inputs, or in the setting of different neuromodulatory conditions. Results yielded insights into circuit information pathways, oscillatory coding mechanisms and the role of HCN in modulating corticospinal output.

LFP revealed physiological oscillations in delta (0.5-4 Hz) and high beta to low gamma (25-40 Hz) ranges across layers and populations. Oscillations occurred in the absence of rhythmic external inputs, emergent from neuronal biophysical properties and circuit connectivity. Filtering the LFP signal from the electrode located in upper L5B revealed phase-amplitude coupling of fast oscillations on delta wave phase. LFP spectrogram demonstrated that the fast oscillations occurred robustly during the time course of simulations. Strong LFP beta and gamma oscillations are characteristic of motor cortex activity, and have been found to enhance signal transmission in mouse neocortex. Phase-amplitude coupling may help integrate information across temporal scales and across networks.

Analysis of firing dynamics and information flow in our model confirmed and extended our understanding of information flow in cortical microcircuits. Consistent with existing models, sensory-related long-range inputs targeted superficial layers which in turn projected to deeper layers. Our simulations, however, provided further details: information flow was cell-class specific, going unidirectionally from IT to PT cells; sublaminar-specific, with superficial ITs targeting primarily the upper portion of L5B PT cells; and oscillation frequency-specific, with Granger causality peaks occurring at shifted beta/gamma range frequencies for different internal connections.

Our work provides insights into oscillatory mechanisms and information flow in M1 microcircuits. Our detailed computational model provides a useful tool for researchers in the field to evaluate novel hypothesis, understand motor disorders and develop novel pharmacological or neurostimulation treatments.

**Acknowledgments:** Research supported by NIH grant U01EB017695, DOH01-C32250GG-3450000, NIH R01EB022903.

**References**

1. Dura-Bernal S, Neymotin SA, Suter BA, Shepherd GMG, Lytton WW. Long-range inputs and H-current regulate different modes of operation in a multiscale model of mouse M1 microcircuits. *bioRxiv* 2017, 07 [Preprint]; 10.1101/201707
2. Dura-Bernal S, Suter B, Gleeson P, et al. NetPyNE: a tool for data-driven multiscale modeling of brain circuits. *bioRxiv* 2018, 461137; 1101/461137 (Under Review in eLife)

## P187 Avalanche power-law values by layer and cell type in simulated mouse primary motor cortex (M1)

### Donald Doherty^1^, Salvador Dura-Bernal^2^, William W Lytton^2^

#### ^1^SUNY Downstate Medical Center, Department of Physiology and Pharmacology, Pittsburgh, PA, United States of America; ^2^SUNY Downstate Medical Center, Department of Physiology and Pharmacology, Brooklyn, NY, United States of America

##### **Correspondence:** Donald Doherty (donald.doherty@actionpotential.com)

*BMC Neuroscience* 2019, **20(Suppl 1)**:P187

[1] reported avalanches in local field potential recordings from organotypic cultures and in unit recordings from acute slices of rat somatosensory cortex with a power-law value of − 1.5. Since then, a range of avalanche size distribution values has been reported from about − 2.05 to − 1.25. Some teams have reported strongly curved avalanche size distributions rather than a power law.

Cortical layer, cell type, and connectivity of individual cells are unavailable for in vitro and in vivo recordings. In addition, sample size and temporal resolution are necessarily limited. By comparison, simulation offers access to all of this information. We therefore investigated the circuitry underlying avalanche propagation in a computer model of area M1 of cerebral cortex. This allowed us to distinguish the characteristics of avalanches along different routes defined by either layer or cell type, as well as throughout the column. The M1 model is a moderately detailed simulation of a full-depth cylindrical column of 300μm diameter, containing 10,074 neurons with about 18 million connections. Activation was with a 0.57nA intracellular square-wave current applied to all cells in a 40μm cylindrical subvolume.

Avalanche sizes from all neuron types and layers of the M1 cortical column showed a linear fit to power-law value − 1.72. Avalanches in all excitatory neurons fit − 1.67 and the population of M1 inhibitory neurons fit to the power-law value − 1.60 for avalanche size. Avalanche sizes in layer 2/3 fit a relatively steep − 1.90 and similarly in layer 4 they fit − 1.87 . The power-law value became sharply less negative in layer 5A at − 1.37 and became more negative again in layer 5B and layer 6 at − 1.67. Excitatory neurons appeared to form two groups. One group includes four neuron types: IT2/3 neurons carried avalanche sizes that fit power-law value of − 1.68, IT5A also neurons fit − 1.68, PT5B fit − 1.63, and IT6 fit − 1.66. Two other excitatory neurons form the second group: IT4 neurons with avalanche sizes that fit − 1.87 and IT5B neurons at − 1.81. Inhibitory neurons also formed two groups. One group had very steep power-law curves and the size of their avalanches did not get very large. This group included PV2/3 neurons with a power-law value of − 2.38 and SOM6 at − 2.36. The second group had somewhat less steep curves and included SOM2/3 − 1.88, PV5A − 1.87, and PV5B − 1.76. SOM5A, SOM5B, CT6, and PV6 neurons had low response rates and therefore did not generate enough data to determine the shape of their avalanche size distributions.

In conclusion, many excitatory neuron types, particularly IT2/3, IT5A, PT5B, and IT6, may operate at criticality in our simulated M1 cortical column. Our data also suggest that some inhibitory neurons, especially PV2/3 and SOM6, may not operate in the critical state. Avalanche size distribution is differentially expressed across different layers and different cell types in our simulated M1 cortical column.

**Acknowledgements:** Supported by NIH U01EB017695, DOH01-C32250GG-3450000, and NIH Brain Initiative Grant R01 EB022903.

**Reference**

1. Beggs JM, Plenz D. Neuronal avalanches in neocortical circuits. *Journal of neuroscience* 2003 Dec 3;23(35):11167–77.

## P188 Mathematical tools for phase control and their role in neural communication

### Gemma Huguet, Alberto Pérez-Cervera, Tere M-Seara

#### Universitat Politecnica de Catalunya, Departament de Matematiques, Barcelona, Spain

##### **Correspondence:** Gemma Huguet (gemma.huguet@upc.edu)

*BMC Neuroscience* 2019, **20(Suppl 1)**:P188

Oscillations are ubiquitous in the brain [1]. Although the functional role of oscillations is still unknown, some studies have conjectured that the information transmission between two oscillating neuronal groups depends on the relative phases between them. Thus, the theory of Communication Through Coherence (CTC) proposes that effective communication occurs when the inputs sent by the emitting population arrive at the phases of maximal excitability of the receiving population [2]. In this context, phase-locking between neural populations is relevant for understanding neuronal communication [3].

The phase response curve (PRC) is a powerful and classical tool to study the effect of a perturbation on the phase of an oscillator, assuming that all the dynamics can be explained by the phase variable. However, factors like the rate of convergence to the oscillator, strong forcing or high stimulation frequency may invalidate the above assumption and raise the question of how is the phase variation away from an attractor [4].

We present powerful computational techniques to perform the effective computation of the phase advancement and phase locking properties beyond weak perturbations. In particular, we consider a population rate model consisting of excitatory and inhibitory cells modeling the receiving population and perturb it with a time-dependent periodic function modeling the input from the emitting population. We consider the stroboscopic map for this system and study its fixed and periodic points and their bifurcations as the amplitude and the frequency of the perturbation are varied. The techniques that we use to do the bifurcation analysis have no restriction neither on the amplitude nor on the frequency of the perturbation. From the bifurcation diagram, we can identify the phase-locked states as well as different areas of bistability. We explore carefully the dynamics on these invariant objects and we discuss the implications of these results for the CTC theory, paying attention to the implications in terms of phase-locking and amplitude of the response of the oscillatory neuronal population to the external input. Our results show that naturally an optimal phase locking for CTC emerges, providing a mechanism by which the receiving population can implement selective communication, as well as a mechanism by which different communication regimes between areas can be established (communication can be turned on and off) without changing the connectivity of the network.

**References**

1. Buzsaki G. Rhythms of the Brain. *Oxford University Press* 2006
2. Fries P. A mechanism for cognitive dynamics: neuronal communication through neuronal coherence. *Trends in cognitive sciences* 2005, 9(10), 474–480.
3. Tiesinga PH, Sejnowski TJ. Mechanisms for phase shifting in cortical networks and their role in communication through coherence. *Frontiers in human neuroscience* 2010 Nov 2;4:196.
4. Ashwin P, Coombes S, Nicks R. Mathematical frameworks for oscillatory network dynamics in neuroscience. *The Journal of Mathematical Neuroscience* 2016 Dec; 6(1):2.

## P189 Emergence of binding capabilities in generic spiking neural networks

### Michael Müller^1^, Robert Legenstein^1^, Christos H. Papadimitriou^2^, Wolfgang Maass^1^

#### ^1^Graz University of Technology, Institute of Theoretical Computer Science, Graz, Austria; ^2^UC Berkeley, EECS, Berkeley, United States of America

##### **Correspondence:** Michael Müller (mueller@igi.tugraz.at)

*BMC Neuroscience* 2019, **20(Suppl 1)**:P189

Language understanding requires the structured encoding of sentence contents in the human brain. A recent fMRI study by Frankland and Greene [1] has shed some light on the representation of such structured information in human cortex: there exist specific subareas in the left mid-superior temporal cortex (lmSTC) which encode semantic variables for thematic roles (such as the agent or patient in a sentence). Furthermore, the authors were able to reliably decode the content of these semantic variables from the corresponding lmSTC subregions.

Assignment of values to semantic variables can be viewed as a form of binding. Existing models addressing this problem either rely on detailed assumptions such as specifically constructed circuitry or strict connectivity assumptions, or they cannot reproduce the findings of Frankland and Greene due to their inner workings. We present a model which avoids both issues.

The proposed model shows how binding capabilities emerge in a generic spiking neural network using minimal assumptions and experimentally well-established neural mechanisms. The network consists of sparsely connected populations of spiking neurons with divisive inhibition, where connections are subject to spike-timing dependent plasticity (STDP). In particular, we make no assumptions on specific wiring or symmetric connectivity. The control over the binding processes is implemented through disinhibition of neural populations.

We show through extensive computer simulations that the assignment of semantic variables (like agent or patient in a sentence) to words or values robustly emerges through STDP in this model. Values are encoding by elevated responses of sparse assemblies in the “content space” (similar to the concept cells of the MTL, see [2]). These values can be assigned to different variables, each encoded by a population of neurons (“variable spaces”). If a variable space is disinhibited while some value is active in the content space (Fig. 1A–B), an assembly forms in the variable space (Fig. 1C). This assembly is strongly linked to the neurons in the content space encoding its value (though sparse, random connectivity).

**Fig. 1:**
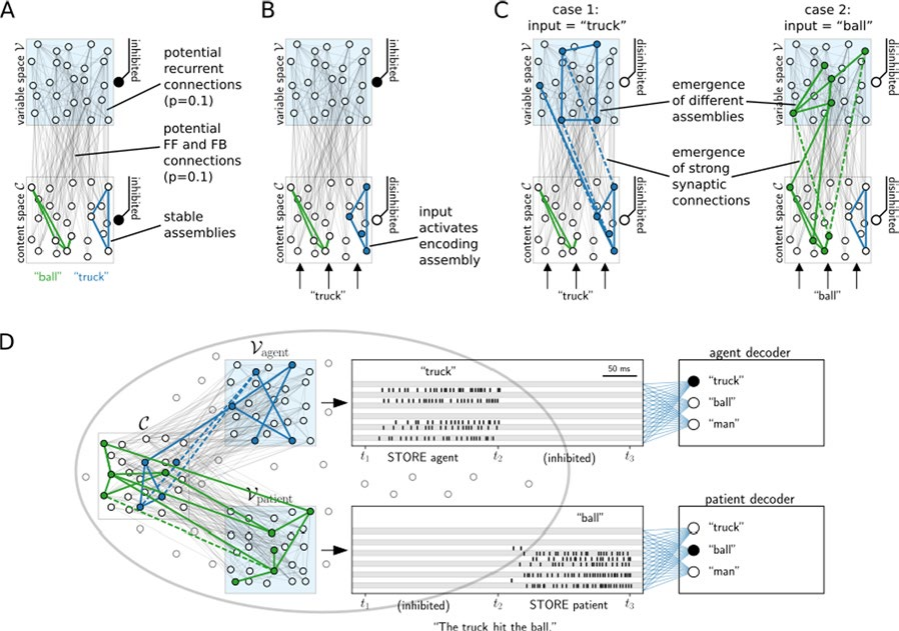
**a** Network architecture with a single variable space for one variable. **b** Input (representing the word “truck”) activates the encoding assembly (blue). **c** Assigning different values (left: “truck”, right: “ball”) gives rise to different encoding assemblies (blue, green) in the variable space. **d** Reproducing lmSTC data: variable contents can be read out from the variable space network dynamics

Such a value assignment is consistent with the findings of Frankland and Greene as the activity of a variable is shaped by its content (Fig. 1C-D). Assigning different values (agent, patient of a sentence) to two separate variables allows reading out the values of the semantic variables from the network activity of the corresponding variable space (with high accuracy, >97% under severe noise conditions), thus reproducing the experimental findings in lmSTC.

The proposed model further allows performing a number of elementary cognitive operations which have been proposed as fundamental primitives of symbolic computation in the brain: recalling the contents of a variable (by disinhibiting the content space and the variable space after a delay, the variable space drives the target assembly in the content space to fire), copying the contents of one variable to another (by performing a recall and simultaneously assigning the recalled value to a new variable space), and comparing the contents of two variables (by performing two consecutive recall operations with a readout assembly connected to the content space via depressing synapses). The model thus can serve as a building block for models performing more demanding cognitive tasks.

**References**

1. Frankland SM, Greene, JD. An architecture for encoding sentence meaning in left mid-superior temporal cortex. *Proceedings of the National Academy of Sciences* 2015, 112.37: 11732–11737.
2. Quiroga RQ. Neuronal codes for visual perception and memory. *Neuropsychologia* 2016 Mar 1;83:227–41.

## P190 Joint effect of Spike-timing-dependent and short-term plasticity in a network of Hodgkin-Huxley neurons

### Ewandson Luiz Lameu^1^, Fernando Borges^2^, Kelly Cristiane Iarosz^3^, Antonio Marcos Batista^4^, Elbert E. N. Macau^1^

#### ^1^National Institute for Space Research (INPE), LAC, São José dos Campos, Brazil; ^2^Federal University of ABC, Center for Mathematics, Computation, and Cognition., São Bernardo do Campo, Brazil; ^3^University of São Paulo, Institute of Physics, São Paulo, Brazil; ^4^State University of Ponta Grossa, Program of Post-graduation in Science, Ponta Grossa, Brazil

##### **Correspondence:** Ewandson Luiz Lameu (ewandson.ll@gmail.com)

*BMC Neuroscience* 2019, **20(Suppl 1)**:P190

Neuronal plasticity, also called brain plasticity, is the capability of the brain to change its function and structure. The plasticity occurs due to external environment, recovery from brain injury, and modifications within the body. We study the effect of both spike-timing dependent (STDP) and short-term (STP) plasticity in the synaptic strength between coupled excitatory Hodgkin-Huxley neurons as a function of their natural frequencies. The STDP rule changes the intensity of the synaptic coupling considering the time interval between the spikes of postsynaptic and presynaptic neurons. The STP is related to the release of neurotransmitters into the synaptic cleft and the recovery time. Previous works reported that the STDP rule induces the appearance of directed connections from the high to low frequency neurons. In our simulations, we observe that the presence of STP with high recovery time allows the existence of connections only when the neurons have close spiking frequencies. We show that, depending on the STP recovery time and the neuronal frequencies distribution, the neuronal network can form clusters of connected neurons with different sizes.

## P191 Transcending model limitations via empirically-tuned parameters

### Alexandre René^1^, Andre Longtin^1,2^, Jakob H. Macke^3^

#### ^1^University of Ottawa, Department of Physics, Ottawa, Canada; ^2^University of Ottawa, Centre for Neural Dynamics, Ottawa, Canada; ^3^Technical University of Munich, Munich, Germany

##### **Correspondence:** Alexandre René (arene010@uottawa.ca)

*BMC Neuroscience* 2019, **20(Suppl 1)**:P191

In order to build interpretable models of neuronal networks, it is necessary to retain only a subset of their biological features. Population models, where the activity of several neurons is averaged and treated as a single functional unit, are a common way of achieving this [1,2]. Such models also address both the experimental limitations in identifying large numbers of individual cells, and the computational limitations in simulating them. What then is the cost of such an approximation, and how can we mitigate it ? We argue that even in models which attempt to account for a biological structure, that cost is substantial, and consequently that any theoretically predicted parameter is unlikely to be better than an order of magnitude estimate. We propose using machine learning methods to close the gap between model and data, by tuning model parameters towards effective values which account for neglected biological features. We show that this provides a substantially better match to data, and can recover behaviours typically lost in population models, such as bursting (Fig. 1A). The use of Bayesian methods also enables us to compute rich posteriors over the model parameters (Fig. 1B).

**Fig. 1:**
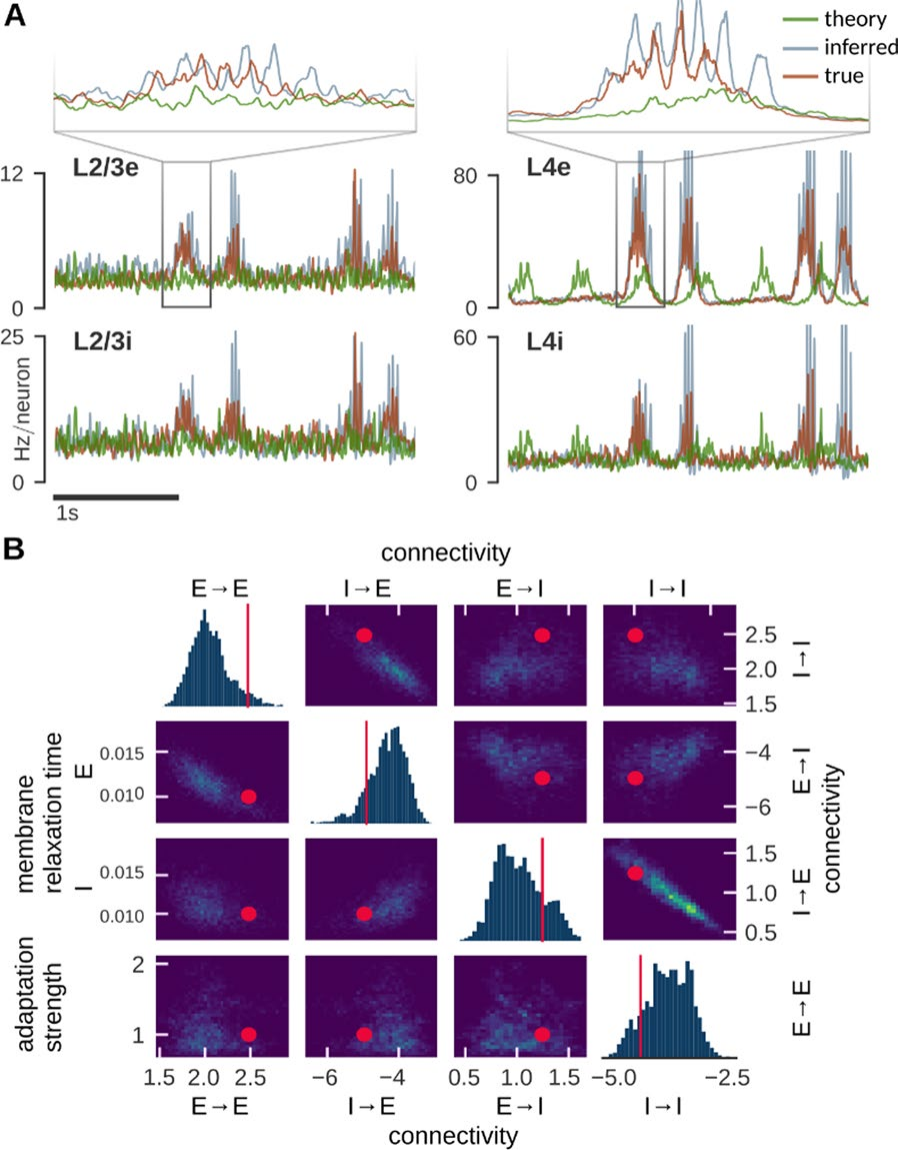
**a** A population model can partially reproduce bursting behavior observed in a spiking model, but only when using inferred effective parameters. Shown are simulations of a putative cortical column containing four populations. Inferred parameters were obtained on non-bursting data. **b** Posterior over parameters for a smaller two-population model. Red dots and lines indicate ground-truth values

Inference methods have been very successful at fitting statistical models to neuron population dynamics. Such models are usually either relatively simple [3], or generic function approximators based on neural networks [4]. In both cases the dynamical equations are chosen to facilitate inference and may be simple caricatures of the underlying biology—they rely on the inference procedure adjusting their parameters in order to provide good predictions. These models are consequently difficult to interpret and may not extrapolate well outside the regime in which they were fit. By extending inference methods to mechanistic models, we are able to combine their explanatory power with the predictive power of statistical models. Moreover, because the dynamical equations are better aligned with the underlying biology, we expect them to generalize to a wider range of dynamical regimes—and this is exactly what we find with respect to bursting behaviour.

We anticipate that empirically tuning mesoscopic models will be a powerful method for further understanding large-scale neural processes, and are releasing software to facilitate its implementation. Compared to simulating an entire network, population models tuned to compensate for unaccounted features provide a simpler mechanistic representation of the population dynamics that remains quantitatively reliable and yet is amenable to theoretical and computational analysis.

**Acknowledgments:** We thank the National Sciences and Engineering Research Council of Canada for supporting Alexandre René and André Longtin.

**References**

1. Schwalger T, Deger M, Gerstner W. Towards a theory of cortical columns: From spiking neurons to interacting neural populations of finite size. *PLOS Computational Biology* 2017, 13.
2. Nykamp DQ, Tranchina D. A Population Density Approach That Facilitates Large-Scale Modeling of Neural Networks: Analysis and an Application to Orientation Tuning. *Journal of Computational Neuroscience* 2000, 8, 19–50.
3. Macke JH, et al. Empirical models of spiking in neural populations. *Advances in Neural Information Processing Systems* 2011, 24, 1350–1358.
4. Pandarinath C, et al. Inferring single-trial neural population dynamics using sequential auto-encoders. *Nature Methods* 2018, 15, 805.

## P192 Hippocampal volume and functional connectivity transitions during the early stage of Alzheimer’s disease: a Spiking Neural Network-based study.

### Gianluca Susi^1^, Isabel Suárez Méndez^1^, David López Sanz^1^, Maria Eugenia López García^1^, Emanuele Paracone^2^, Ernesto Pereda^5^, Fernando Maestu^1^

#### ^1^Center for Biomedical Technology, Technical University of Madrid, Laboratory of Cognitive and Computational Neuroscience, Madrid, Spain; ^2^University of Rome “Tor Vergata”, Department of civil engineering and computer science, Rome, Italy; ^3^University of La Laguna, Industrial Engineering, San Cristobal de La Laguna, Spain

##### **Correspondence:** Gianluca Susi (gianluca.susi@ctb.upm.es)

*BMC Neuroscience* 2019, **20(Suppl 1)**:P192

Acting as a dynamical relaying center between different cortical areas, the hippocampus is known to significantly contribute in shaping the functional connectivity (FC) profile of the cortex. Many works in the field are being addressed to understand how the hippocampus has an impact on other cortical regions, both during motor action and at rest, but also from single areas to larger subnetworks, such as the default mode network (DMN), a resting state (RS) network, more strongly active during idling states than during task performance. On one hand, some studies have reported a progressive decrease of hippocampal volume during the time course of early-stage Alzheimer’s disease (AD), which has been attributed to the loss of neurons and the deterioration of the related connections. On the other hand, the DMN appears to be functionally impaired in AD and even in earlier stages, as in mild cognitive impairment (MCI).

In light of this, the present work aims at understanding the mechanistic underpinnings of FC transitions that take place during MCI. Specifically, we wanted to unveil whether the transitions of the DMN FC observed in this early phase of the AD can be attributed to the deterioration of the hippocampus as a dynamical relaying center between cortical areas. To this purpose, we investigated the RS interactions between the hippocampus and the remaining areas of the DMN (identified in our work by 12 areas (right and left): precuneus, isthmus cingulate, inferior parietal cortex, superior frontal gyrus, middle temporal gyrus, and anterior cingulate cortex). Functional and structural connectivity (SC) profiles have been extracted from n = 9 healthy controls (HC). We generated spiking-neuron based personalized models of the DMN of these subjects, including the hippocampal relay network (HRN, i.e. the star-like network composed of the hippocampus and its connections to the remaining 12 cortical areas of the DMN). We generated two different versions of the HRN: HRNHC (the healthy version, sized with data from healthy participants) and HRNMCI (the degraded version, based on volumetric data from a group of MCI participants). Then, we have simulated 30 s of RS activity and calculated the DMN FC profiles of each subject under the two conditions (i.e., using the HRNHC first, and then the HRNMCI). We compared the FC transitions caused by the degradation of the HRN in the model, with those that have emerged from a previous comparative study (HC vs. MCI) carried out in our laboratory with real subjects [1]. Differences were evaluated in the alpha band, where the reference study had reported significant results.

Simulation results show that the structural modification of the HRN is able to predict up to 80% of the FC variations on the whole DMN, found in the reference study.

Our findings suggest that the misadjustment of the hippocampus as a relaying center between cortical areas could be playing a pivotal role on the disruption of the FC at the initial stage of the disease, when the SC is not yet considerably damaged. It could be the hippocampus malfunction that subsequently triggers a plasticity-driven reconfiguration process, causing over time the structural and functional disruptions that characterize most advanced AD stages.

**Reference**

1. Garcés P, et al. The Default Mode Network is Functionally and Structurally Disrupted in Amnestic Mild Cognitive Impairment - A Bimodal MEG-DTI Study. *NeuroImage: Clinical* 2014 Jan 1;6:214–21.

## P193 A pipeline integrating high-density EEG analysis and graph theory: a feasibility study on resting state functional connectivity

### Riccardo Iandolo^1^, Michela Chiappalone^2^, Jessica Samogin^3^, Federico Barban^4^, Stefano Buccelli^1^, Gaia Taberna^3^, Marianna Semprini^1^, Dante Mantini^3^

#### ^1^Fondazione Istituto Italiano di Tecnologia, Rehab Technologies, Genova, Italy; ^2^Istituto Italiano di Tecnologia, Genova, Italy; ^3^Katholieke Universiteit Leuven, Research Center for Motor Control and Neuroplasticity, Leuven, Belgium; ^4^Fondazione Istituto Italiano di Tecnologia, Rehab Technologies, IIT-INAIL Lab, Genova, Italy

##### **Correspondence:** Riccardo Iandolo (riccardo.iandolo@iit.it)

*BMC Neuroscience* 2019, **20(Suppl 1)**:P193

Recent advances in the field of human brain imaging by electrophysiological recordings provide novel tools to investigate the connectivity patterns during spontaneous oscillatory activity. As a consequence, we could reliably explore, also by means of electrophysiological data, the mechanisms underlying complex brain mechanisms, such as the functional reorganization after brain injury. As a first step towards this goal, we developed a pipeline integrating high-density electroencephalography (hdEEG) and graph theory and we tested it on the resting-state activity of 16 healthy subjects. The goal of our analysis is to explore, by using weighted graphs, the frequency-specific organization of different graph-derived metrics such as the clustering coefficient, the path length and the recent small world propensity index [1]. To reach the above goal, we employed state-of-the-art techniques to process hdEEG channels, to build the head volume conductor model and to estimate thesources location in the gray matter [2]. Once the sources were estimated, we mapped them onto the 384 ROIs of the AICHA atlas [3]. The spectral estimates of each ROI were derived using Morlet wavelet (number of cycles = 5.83). We then employed the power spectra envelope orthogonalization method to unravel the connectivity value between the estimated sources that, otherwise, would be masked by the source leakage problem. Thus, ROIs power envelopes were firstly orthogonalized, log-transformed and then correlated as reported in [4]. The obtained adjacency matrices, containing the strength of the connections, were statistically thresholded to further obtain the graph-based metrics (see Fig. 1). We tested the pipeline on resting state recordings, by showing the behavior of the graph parameters in the different Morlet wavelets’ carrier frequency describing the frequency-specific changes of the brain functional organization. Our future plan is to exploit this pipeline to compute hdEEG derived electrophysiological biomarkers of the sensorimotor recovery in patients with neurological disease enrolled in neurorehabilitation programs.

**Fig. 1:**
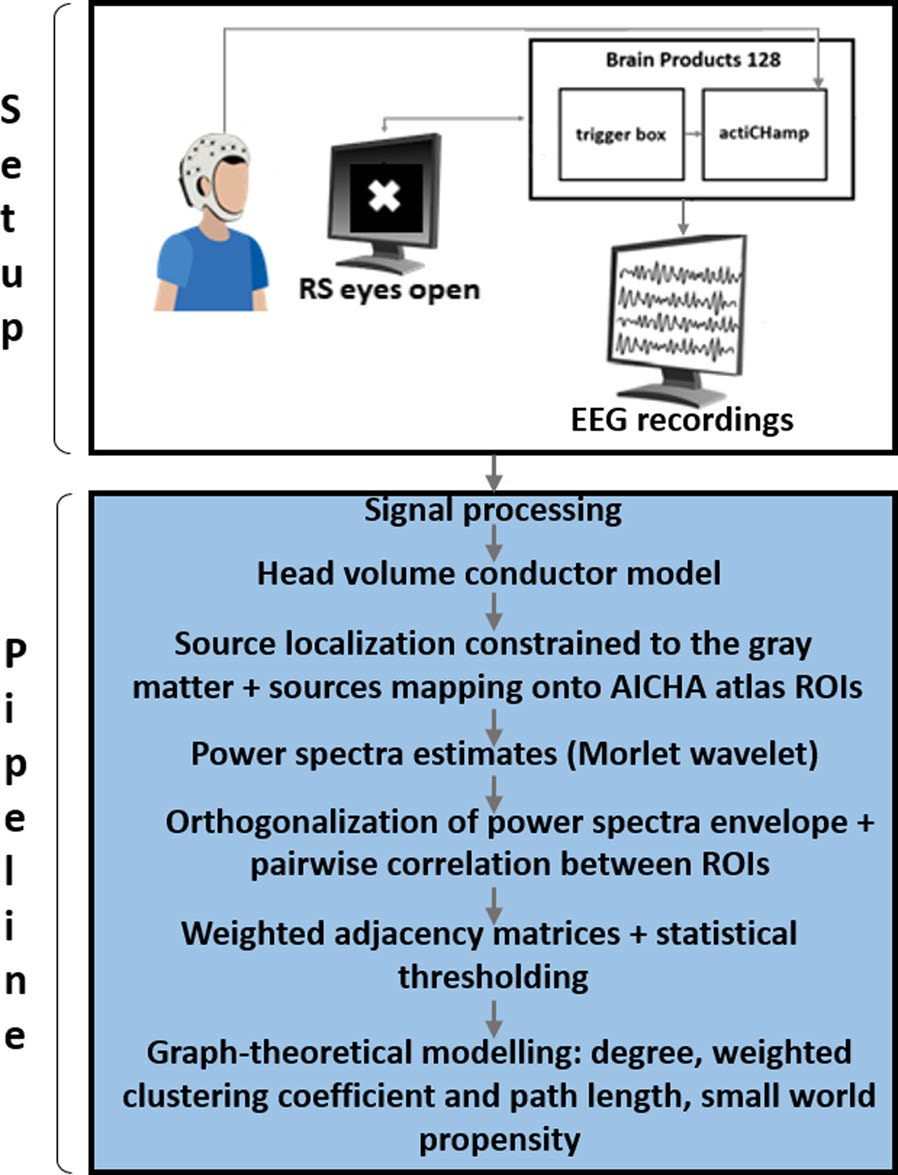
Overview of the experimental setup and of the developed pipeline

**References**

1. Muldoon SF, Bridgeford EW, Bassett DS. Small-world propensity and weighted brain networks. *Scientific report*. 2016 Feb 25;6:22057.
2. Liu Q, Farahibozorg S, Porcaro C, Wenderoth N, Mantini D. Detecting large‐scale networks in the human brain using high‐density electroencephalography. *Human brain mapping* 2017 Sep;38(9):4631–43.
3. Joliot M, Jobard G, Naveau M, et al. AICHA: An atlas of intrinsic connectivity of homotopic areas. *Journal of neuroscience methods* 2015 Oct 30;254:46–59.
4. Siems M, Pape AA, Hipp JF, Siegel M. Measuring the cortical correlation structure of spontaneous oscillatory activity with EEG and MEG. *NeuroImage* 2016 Apr 1;129:345–55.

## P194 Channelrhodopsin-2 model with improved computational efficiency

### Ruben Schoeters^1^, Thomas Tarnaud^1^, Wout Joseph^1^, Robrecht Raedt^2^, Emmeric Tanghe^1^, Luc Martens^1^

#### ^1^University of Ghent, Waves - Information technology, Ghent, Belgium; ^2^University of Ghent, 4Brain lab - Department of Neurology, Ghent, Belgium

##### **Correspondence:** Ruben Schoeters (ruben.schoeters@ugent.be)

*BMC Neuroscience* 2019, **20(Suppl 1)**:P194

Optogenetics is a neuromodulation technique that uses light to control neuronal activity. To this end, light sensitive ion channels or pumps (termed opsins) are genetically expressed into neurons. Channelrhodopsin-2 (ChR2) is an excitatory opsin consisting of seven transmembrane helices covalently bound with a retinal chromophore. Illumination of the opsin triggers a retinal 13 trans-cis isomerization followed by opening of the pore. UV/vis and difference infrared spectroscopy identified at least five different states in a single photocycle. Furthermore, electrophysiological recordings, retinal extraction and Raman measurements provide evidence for the existence of a second photocycle, which is widely adopted [1]. The place of transition between this dark- and light adapted photocycle is however still under debate (Fig 1, left). In-silico, the whole photocycle is predominantly modelled with a four-state branched model that consists of two open and closed states (Fig 1, middle). Moreover, an extra state-variable is typically used to model the time- and irradiance dependent activation [2]. Consequently, the model consists of four differential equations making it quite computational demanding.We proposed an alternative model that is based on the fast transient sodium model of Hodgkin and Huxley. However, instead of inactivation in the Hodgkin and Huxley model, the second state pair represents the light-dark adaptation (Fig 1, right). This model requires only two differential equations, thus reducing the number of equations with fifty percent. Furthermore, by using two light dependent rates in the light-dark adaptation cycle, we hypothesized no loss of ChR2 current features (i.e. a transient peak followed by a steady-state plateau and slow recovery from light adaptation, under voltage-clamp conditions). This hypothesis was tested and confirmed, by fitting our model to voltage-clamp recordings reported by [2]. For both the equilibrium and time constants, a logistics relationship was used to incorporate intensity and voltage dependence. However, these dependences were on a logarithmic and linear scale, respectively. Subsequently, the obtained model was compared against the 4-state branched model created by [2]. The computational efficiency was addressed in a cortex network model, consisting of 36 excitatory neurons containing the ChR2 current model and 12 inhibitory neurons. The simulation was solved with a global variable step and variable order solver (ode15s) in MATLAB. For a two second simulation containing one second of optical stimulation, an average (n = 10) of 220.73 s and 175.32 s computation time was required, for the configuration with the 4-state branched and own model, respectively. The proposed model results thus in a significant increase of computational efficiency.

**Fig. 1:**
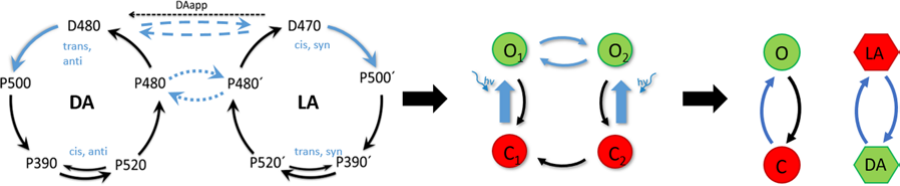
The ChR2 photocycle based on UV/Vis and difference infrared spectroscopic measurements (left). The transition between the dark adapted (DA) and light adapted (LA) occurs either at the parent states (dashed step) or at the late intermediates (dotted step). A four state branching model (middle). The proposed model with opening and light-dark adaptation separately (right)

**References**

1. Bruun S, Stoeppler D, Keidal A, et al. Light-Dark Adaptation of Channelrhodopsin Involves Photoconversion between the all-trans and 13-cis Retinal Isomers. *Biochemistry* 2015, 54, 5389–5400.
2. Williams JC, Xu J, Lu Z, et al. Computational Optogenetics: Empirically-Derived Voltage- and Light-Sensitive Channelrhodopsin-2 Model. *PLoS Computational Biology* 2013, 9.

## P195 A model of presynaptic KV7 channel function in hippocampal mossy fiber bouton

### Elisabetta Giacalone^1^, Michele Migliore^1^, David Anthony Brown^2^, Mala Shah^3^, Katiuscia Martinello^3^

#### ^1^CNRS, Biophysics Institute, Palermo, Italy; ^2^Neuroscience Physiology and Pharmacology, University College London, Neuroscience Physiology and Pharmacology, London, United Kingdom; ^3^UCL School of Pharmacy, University College London, School of Pharmacy, London, United Kingdom

##### **Correspondence:** Elisabetta Giacalone (elisabetta.giacalone@pa.ibf.cnr.it)

*BMC Neuroscience* 2019, **20(Suppl 1)**:P195

In hippocampus, a type of slowly-activated and non-inactivating K+ channels, belonging to the Kv7 family, are highly localized on initial segments of myelinated and unmyelinated axons where they influence neuronal excitability. Interestingly, immunohistochemistry shows that the Kv7.2 and Kv7.3 subunits are localized throughout hippocampal mossy fibers. Electrophysiological recordings from mature synaptic boutons showed that the Kv7/M- current is also present here, is active at rest and enhances the membrane conductance. The current also reduces the spike half-width and after depolarization (ADP) following a presynaptic spike. This is likely to have significant consequences for the modulation of excitatory neurotransmitter release from these boutons, and thus signal transmission, at DG-CA3 synapses.

In this poster, using a biophysical computational model of a mossy fiber bouton (MFB), we will discuss the mechanisms underlying the ADP observed after Kv7 channels block. The model is able to reproduce a number of experimental findings under control and after Kv7 current block by XE991. The results suggest that Kv7 conductance limits spike-induced rise in Ca2+concentration and regulates spike width and ADP amplitude. The model suggests that the ADP is caused by a relatively slow Ca2+-dependent mechanism, which can be conveniently modelled as a slow deactivation time constant of the Ca2+current in MFB. This is a new feature that has not been previously observed experimentally. Taken together, these results suggest that presynaptic Kv7 channels expression at the mossy fiber-CA3 synapse may have an important role in modulating synaptic transmission and signal coding in the hippocampus network.

## P196 Characterization of the network dynamics of interconnected brain regions on-a-chip

### Martina Brofiga, Paolo Massobrio, Sergio Martinoia

#### University of Genova, Department of Informatics, Bioengineering, Robotics, System Engineering (DIBRIS), Genova, Italy

##### **Correspondence:** Martina Brofiga (martina.brofiga@dibris.unige.it)

*BMC Neuroscience* 2019, **20(Suppl 1)**:P196

Neuronal networks are composed of different cell types precisely arranged into organizational schemes and connected via complex electrochemical signaling mechanisms [1]. Since the high brain organization complexity, the use of simplified in vitro experimental models is a valid choice to better investigate how dynamics originate and propagate in the different assemblies. In this work, we investigated the mutual interactions between cortical and hippocampal neuronal networks an extremely important communication pathway for understanding the mechanism behind many pathologies, like epilepsy and depression.

In this work, we used a polydimethylsiloxane (PDMS) device to drive the connectivity of three sub-populations of cortical and hippocampal neurons. The device consists of a compartment with a larger area (width = 6.5 mm, length = 4.3 mm). The two smaller compartments have a diameter of 3.4 mm. There are 20 micro-channels (width = 10mm, length = 100mm and height = 5 μm) for each branch. Such a device is coupled to Micro-Electrode Arrays (MEAs) by splitting into three interconnected regions the recorded area where the neurons can recreate modular connectivity [2].

Cortical-hippocampal cultures were grown on MEAs with 120 electrodes to investigate network activity during their development. We carried out recordings of homogeneous interconnected cortical or hippocampal neurons (controls) and heterogeneous interconnected hippocampal and cortical neurons. Spiking and bursting features have been characterized using: Mean Firing Rate, Mean Bursting Rate, Burst Duration, Inter Burst Interval and percentage of random spikes. Kruskal-Wallis test was used to statistically assess the behaviour of the cortical cell with and without the interaction with the hippocampal cells. We demonstrated the functional separation of the two neuronal populations by evaluating the similarity between the spike trains [3]. In the homogeneous cultures, we observed that the similarity takes medium-high values regardless of compartment of belonging. From the functional point of view, this result revealed that there is a high probability to have the same types of neurons in the three compartments. In the heterogeneous cultures we observed that the recorded cortical activity was different respect to hippocampal cells. This outcome suggests the functional separation between the different compartments. To verify the functional connection between the compartments we studied the propagation of the electrophysiological signals in the cultures with and without compartmentalization. We assessed the ignition sites where the Network Bursts (NBs) started from as the sites where bursts initiate in at least 3% of all the NBs [4]. We observed that the compartments are mutually connected. Finally, using the graph theory, we studied the functional connectivity that evidenced a small world network topology.

**References**

1. Forró C, et al. Modular microstructure design to build neuronal networks of defined functional connectivity. *Biosensors and Bioelectronics* 2018 Dec 30;122:75–87.
2. Meunier D, Lambiotte R, Bullmore ET. Modular and hierarchically modular organization of brain networks. *Frontiers in neuroscience* 2010 Dec 8;4:200.
3. Victor JD, Purpura KP. Nature and precision of temporal coding in visual cortex: a metric-space analysis. *Journal of neurophysiology* 1996 Aug 1;76(2):1310–26.
4. Frega M, Tedesco M, Massobrio P, Pesce M, Martinoia S. Network dynamics of 3D engineered neuronal cultures: a new experimental model for in-vitro electrophysiology. *Scientific reports* 2014 Jun 30;4:5489.

## P197 SpykeTorch: Efficient simulation of convolutional spiking neural networks with at most one spike per neuron

### Milad Mozafari^1^, Mohammad Ganjtabesh^1^, Abbas Nowzari-Dalini^1^, Timothee Masquelier^2^

#### ^1^University of Tehran, Department of Computer Science, Tehran, Iran; ^2^CNRS, Toulouse, France

##### **Correspondence:** Timothee Masquelier (timothee.masquelier@cnrs.fr)

*BMC Neuroscience* 2019, **20(Suppl 1)**:P197

Deep convolutional spiking neural networks (DCSNNs) are the next generation of neural networks that are hardware-friendly and energy-efficient. They are able to function in both spatial and temporal domains which makes them potentially more computationally powerful than deep convolutional neural networks (DCNNs).

Despite the recent advances of DCSNNs, their performance has not been better than DCNNs yet. One of the main reasons for the rapid improvements of DCNNs is the existence of efficient and user-friendly simulation frameworks that facilitate the implementation of new ideas. On the other hand, most of the current DCSNN simulators not only need dealing with complex details of neural mechanisms, but also are not efficient enough for examining ideas on large neural networks.

Here, we introduce SpykeTorch which is an open-source PyTorch-based package for efficient simulation of DCSNNs with at most one spike per neuron and time-to-first-spike information coding. Compatible learning rules can be easily added to SpykeTorch, however, spike-timing-dependent plasticity (STDP) and reward-modulated STDP (R-STDP) are already provided. The proposed package is fully integrated with PyTorch and makes use of its high-speed tensor-based operations. Due to this integration, models implemented in SpykeTorch can be easily launched on CPU, GPU, or Multi-GPU platforms. Apart from the efficiency, implementing models with SpykeTorch is almost similar to PyTorch’s workflow which is familiar to deep learning communities.

Temporal domain is crucial to DCSNNs. Here, the concept of time is implemented by adding an extra dimension to the tensors. In other words, spikes and potentials are stored in four-dimensional tensors of the shape (time-steps, features, height, width), filled with binary and floating-point values, respectively. Besides, spikes are kept in accumulative format: if a neuron fires at a particular time-step, we keep its spike flag “on” till the final time-step (Fig. 1). This accumulative structure enables SpykeTorch to simultaneously operate on and returning the outputs of all time-steps.

**Fig. 1:**
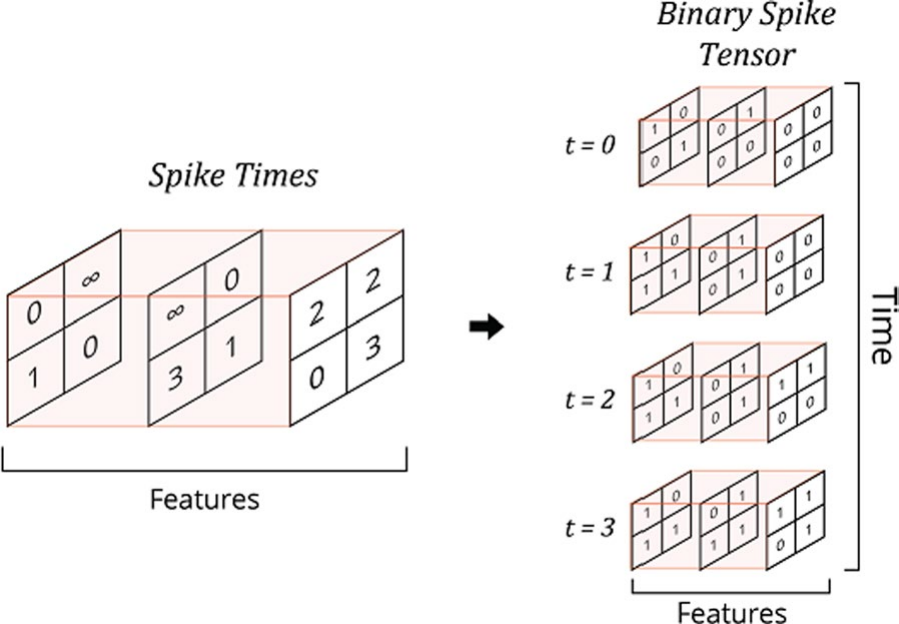
An example of generating binary spike tensor from spike times. There are three feature maps, each constitutes a 2 × 2 grid of neurons. If the maximum number of time-steps is 4, then the result is a four-dimensional tensor of size 4 × 3 × 2 × 2. ∞ means the neuron will never fire

SpykeTorch is already fully functional, and we re-implemented models proposed in [1, 2] (available on GitHub [3]). We are now developing further facilities such as automation modules and batch processing or utilities to work with modalities other than vision to enhance the user experience.

**References**

1. Mozafari M, Kheradpisheh SR, Masquelier T, Nowzari-Dalini A, Ganjtabesh M. First-spike-based visual categorization using reward-modulated STDP. IEEE transactions on neural networks and learning systems. 2018 May 8(99):1–3.
2. Mozafari M, Ganjtabesh M, Nowzari-Dalini A, Thorpe SJ, Masquelier T. Bio-inspired digit recognition using spike-timing-dependent plasticity (stdp) and reward-modulated stdp in deep convolutional networks. arXiv preprint arXiv:1804.00227. 2018 Mar 31.
3. Mozafari M. SpykeTorch – High-speed simulator of convolutional spiking neural networks with at most one spike per neuron. *GitHub repository* 2019, https://github.com/miladmozafari/SpykeTorch

## P198 Slow-wave activity enrichment across brain states in the mouse

### Antonio Pazienti^1^, Miguel Dasilva^2^, Andrea Galluzzi^1^, Maria V. Sanchez-Vives^2,3^, Maurizio Mattia^1^

#### ^1^Istituto Superiore di Sanità, National Center for Radiation Protection and Computational Physics, Rome, Italyv^2^IDIBAPS, Systems Neuroscience, Barcelona, Spain; ^3^ICREA, Systems Neuroscience, Barcelona, Spain

##### **Correspondence:** Maurizio Mattia (maurizio.mattia@iss.it)

*BMC Neuroscience* 2019, **20(Suppl 1)**:P198

Slow-wave activity is a hallmark of sleep and deep anesthesia and constitutes a default spatiotemporal pattern invariantly expressed by cortical networks upon which cognitive functions emerge during wakefulness [1]. The transition from slow-wave activity to wakefulness is thus an ideal experimental framework to understand how complex network dynamics underlying brain computations emerge.

With this aim, we studied the generation and propagation of slow waves under different anesthesia (isoflurane) levels, by means of an array of 32 surface electrodes covering a large part of a mouse brain hemisphere. The propagation of slow waves was detected relying on the multisite, multiunit activity extending a methodology introduced in [2]. For each wave, the estimated matrix of time lags between local activation onsets (transitions from almost quiescent Down to high-firing Up states) reflected the shape of the propagating activity wave fronts. We quantified the properties of these spatiotemporal patterns, as well as their frequency and degree of diversity in time and space. At the deepest anesthesia level, we observed two modes: back-to-front and front-to-back waves [3]. With fading anesthesia, the complexity of the propagation modes (quantified with the entropy computed on the plane of the first two PC components) increases, until a continuum of wave front shapes arises. Besides, both the frequency of Up/Down slow oscillations and the regularity of the rhythm (as measured by the coefficient of variation cv of Up/Down cycles) increase together such that under light anesthesia the highest frequency (≈0.5 Hz) occurs with the lowest variability (cv≈0.2). As a result, wave entropy tightly correlates with the slow oscillation frequency allowing us to work out an anesthesia depth index (ADI), which turns out to correlate with the cv and the average Up and Down state durations too. Intriguingly, the condition where cortical travelling waves show poor diversity (low entropy) and large variability of the rhythm, are characterized by increased network memory, measured as similarity of two successive wave fronts. This is in contrast with lighter levels of anesthesia, where the waves display memory-lessness. It is worthy to mention that despite all these changes, the wave velocity does not vary significantly (10.3±1.6 mm/s, mean± SD, n = 19).

In the observed brain state transition two, apparently incoherent phenomena emerge: increase of temporal regularity/memory and of spatial complexity. To unravel this paradox, we resorted to simulations of spiking neuron networks modeling the probed cortical areas by extending [2]. In order to reproduce the properties of the in vivo slow-wave activity, the model had to incorporate a balanced competition between an increase of global cortical excitability and the presence of a state-dependent inhibition/refractoriness related to more local fatigue processes. As a result, the starting low complexity and propagation mode memory can be due to fatigue effects in the network: waves tend to be stereotypical and follow ‘known tracks’.

**Acknowledgements**: We thank the EU Horizon 2020 Research and Innovation Programme under HBP SGA2 for supporting M.V.S.-V. and M.M. with grant no. 785907.

**References**

1. Sanchez-Vives MV, Massimini M, Mattia M. Shaping the default activity pattern of the cortical network. *Neuron* 2017 Jun 7;94(5):993-1001.
2. Capone C, Rebollo B, Muñoz A, et al. Slow waves in cortical slices: How spontaneous activity is shaped by laminar structure. *Cerebral Cortex* 2017 Nov 28;29(1):319-35.
3. Greenberg A, Abadchi JK, Dickson CT, Mohajerani MH. New waves: Rhythmic electrical field stimulation systematically alters spontaneous slow dynamics across mouse neocortex. *Neuroimage* 2018 Jul 1;174:328-39.

## P199 A spiking neural network model of the N400 congruency effect

### Nirav Porwal^1^, Howard Bowman^2^, Maxine Lintern^1^, Kimron Shapiro^1^, Eirini Mavritsaki^1^

#### ^1^Birmingham City University, Department of Psychology, Birmingham, United Kingdom; ^2^University of Kent, Centre for Cognitive Neuroscience and Cognitive Systems and the School of Computing, Canterbury, United Kingdom

##### **Correspondence:** Nirav Porwal (nirav.porwal@mail.bcu.ac.uk)

*BMC Neuroscience* 2019, **20(Suppl 1)**:P199

Alzheimer’s disease (AD) is a chronic progressive neurodegenerative disorder afflicting millions worldwide. Abnormal N400 Event-Related Potentials (ERP) are biomarkers indicative of AD progression [1]. Specifically, in the semantic category judgment task, N400 congruency and repetition effects diminish with AD progression in Mild Cognitive Impairment Patients. Aberrant neuronal properties in AD such as Calcium (Ca2+) concentrations and N-methyl-D-aspartate (NMDA) receptor dysfunction could be the underlying cause of these ERP abnormalities [2, 3]. However, there is no consensus in the literature on the cognitive functions or specific neural generators of the N400 nor detailed neuronal models that account for these factors.

Here we propose, to our knowledge, the first biologically detailed and plausible connectionist spiking neural network architecture to model the semantic category judgment task. The architecture’s neuronal characteristics are based on the spiking Selection over Time and Space (sSoTS) model that encompasses gamma aminobutyric acid (GABA), alpha-amino-3-hydroxy-5-methyl-4-isoxazolepropionic acid (AMPA), NMDA, and the spike frequency adaptation currents [4]. AMPA&GABA account for fast excitatory and inhibitor currents, respectively. NMDA accounts for slow magnesium ion dependent currents. The spike frequency adaptation current is a Ca2+ ion dependent after-hyperpolarization mechanism. The architecture has groups of neurons divided into pools arranged in layers as seen in Fig. 1. Each layer represents a specific feature type i.e. auditory, visual, or semantic. Pools within a layer represent stimulus properties. The connectivity between and within pools and layers is approximated by converting the architecture into a population coded model using the Mean Field Approach, permitting the exploration of a large parameter space.

**Fig. 1:**
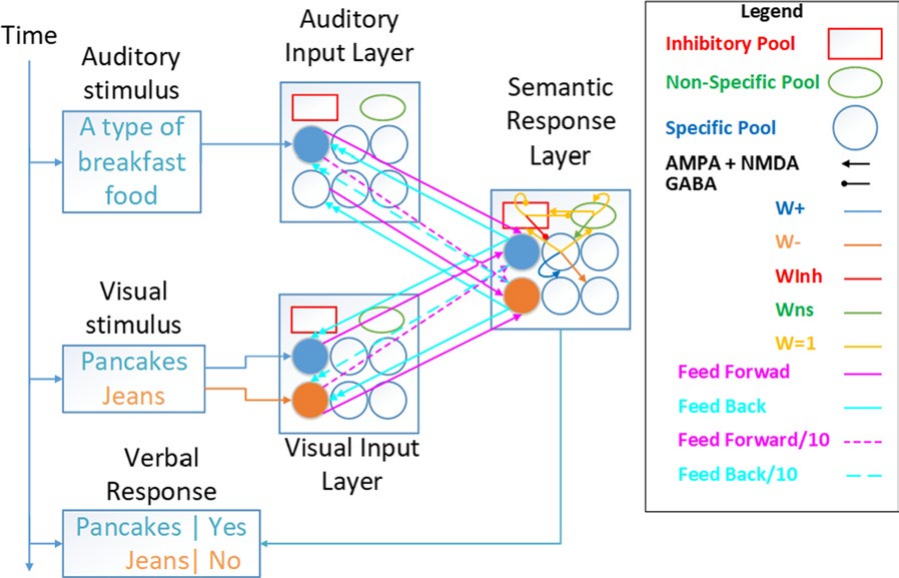
Implementation of the semantic category judgement task in the model

The proposed model architecture successfully generates spiking activity in the semantic layer similar in time course as found in the N400 literature. The results suggest that N400s for individual stimuli are generated by stimulus-induced spiking activity in the semantic layer through AMPA and NMDA currents. The N400 congruency effect is generated by the spike frequency adaptation mechanism. This model provides a biologically detailed and plausible account of the N400 ERP in the semantic judgement task and is the first step in understanding the N400 biomarker in AD patients.

**References**

1. Olichney JM, Yang JC, Taylor J, Kutas M. Cognitive event-related potentials: biomarkers of synaptic dysfunction across the stages of Alzheimer’s disease. *Journal of Alzheimer’s Disease* 2011 Jan 1;26(s3):215–28.
2. Kuchibhotla KV, Goldman ST, Lattarulo CR, Wu HY, Hyman BT, Bacskai BJ. Aβ plaques lead to aberrant regulation of calcium homeostasis in vivo resulting in structural and functional disruption of neuronal networks. *Neuron* 2008 Jul 31;59(2):214–25.
3. Danysz W, Parsons CG. Alzheimer’s disease, β‐amyloid, glutamate, NMDA receptors and memantine–searching for the connections. *British journal of pharmacology* 2012 Sep;167(2):324–52.
4. Mavritsaki E, Heinke D, Allen H, Deco G, Humphreys GW. Bridging the gap between physiology and behavior: Evidence from the sSoTS model of human visual attention. *Psychological review* 2011 Jan;118(1):3.

## P200 Maximizing transfer entropy promotes spontaneous formation of assembly sequences in recurrent spiking networks

### David Kappel^1^, Christian Tetzlaff^1^, Florentin Wörgötter^1^, Christian Mayr^2^

#### ^1^University of Göttingen, Bernstein Center for Computational Neuroscience, Göttingen, Germany; ^2^TU Dresden, Chair of Highly-Parallel VLSI Systems and Neuro-Microelectronics, Dresden, Germany

##### **Correspondence:** David Kappel (david.kappel@uni-goettingen.de)

*BMC Neuroscience* 2019, **20(Suppl 1)**:P200

Experimental data and theoretical considerations suggest that strongly interconnected groups of active neurons, called neural assemblies, constitute the ‘language’ of the brain. Chaining such assemblies leads to the formation of *assembly sequences*, which are commonly found in experimental data and have been related to cognitive processes such as memory recall, decision making and planning [1]. However, the mechanisms that allow neural networks to spontaneously form assembly sequences without supervision are not fully understood. We present a new mathematical framework grounded in information theory, that sheds light on the principles for self-organization that may underlie assembly sequence formation. We hypothesize that a viable objective for learning in recurrent neural networks is to maximize its ability to predict the future continuation of transient stimuli while keeping a minimum information about the past. We formulate this goal in terms of the *transfer entropy* [2], measuring the amount of information about future stimuli that is gained by adding additional neural responses to the assembly sequence. Neurons therefore learn to contribute to the sequence only if their activity helps to increase the predictive power of the network.

The learning rules that emerge from the transfer entropy framework are local and resemble commonly observed STDP curves. In contrast to previously considered Hebbian plasticity, we find that learning rules derived from our theoretical framework include competition between neurons that trades off expressiveness against redundancy. More precisely, we identify a new mechanism that depresses synapses if neurons reverberate information that is already present in the network. We show that this mechanism leads to robust learning where neurons preferably respond with transient activity to external stimuli, and the automatic formation of stable cell assemblies without a global supervisor. To do so we conduct experiments for learning sequence memory and prediction in recurrent neural networks. Furthermore, we find that the competition induced by the transfer entropy learning rule preferably leads to hierarchically organized assembly sequences, where single neurons learn to fill niches of missing information in the spike train, e.g. to close a gap for sequence memory.

The new theoretical framework has interesting correspondences to previously considered learning models. It provides a new view on the predictive coding paradigm [3] and allows us to pinpoint learning rules that enable a network to develop neural codes that minimize surprise. Moreover, transfer entropy learning generalizes the previously considered spiking information bottleneck model [4]. In summary, our approach provides a new theory towards understand the ability of spiking neurons to self-organize into robust assembly sequences.

**References**

1. Buzsáki G. Neural syntax: cell assemblies, synapsembles, and readers. *Neuron* 2010 Nov 4;68(3):362–85.
2. Schreiber T. Measuring information transfer. *Physical review letters* 2000 Jul 10;85(2):461.
3. Friston K. Does predictive coding have a future? *Nature neuroscience* 2018 Aug;21(8):1019.
4. Klampfl S, Maass W, Legenstein RA. Information bottleneck optimization and independent component extraction with spiking neurons. *In Advances in neural information processing systems* 2007 (pp. 713–720).

## P201 Accelerating 3D intracellular NEURON simulations

### Cameon Conte^1^, Adam JH Newton^2^, Lia Eggleston^3^, Michael Hines^4^, William W Lytton^2^, Robert McDougal^4^

#### ^1^Yale University, Neuroscience and Medical Informatics, New Haven, United States of America; ^2^SUNY Downstate Medical Center, Department of Physiology and Pharmacology, Brooklyn, United States of America; ^3^Yale University, Yale College, New Haven, CT, United States of America; ^4^Yale University, Department of Neuroscience, CT, United States of America

##### **Correspondence:** Robert McDougal (robert.mcdougal@yale.edu)

*BMC Neuroscience* 2019, **20(Suppl 1)**:P201

The NEURON simulator (neuron.yale.edu) provides a computational framework for studying networks of neurons and the interplay between electrophysiology and chemical dynamics. While NEURON has supported 3D intracellular dynamics for years, the computational requirements previously put this out of reach for many investigators. To make these studies practical for more researchers, we have developed a new 3D intracellular engine—planned for widespread release with NEURON 7.7—that accelerates serial simulations by an order of magnitude and can take advantage of parallel hardware while preserving NEURON’s traditional reaction-diffusion interface.

The new 3D intracellular compute engine adapts the Douglas-Gunn Alternating Direction Implicit method [1] used for NEURON’s 3D extracellular simulation [2] to the irregularly shaped geometry of a neuron. Voxelization proceeds from an abstract neuron reconstruction using rules adapted from [3], but by explicitly incorporating the connectivity information, improving voxel-NEURON segment mapping and accelerating the discretization. Additionally, the new engine yields a speedup of approximately 10 times for serial simulations compared to the previous Python implementation. This speedup can be further enhanced using threads, exploiting the fundamentally parallel nature of the algorithm; we are currently achieving a speedup of approximately 3 times with 4 threads. With careful cache management, we expect to achieve a performance improvement that scales with the number of threads.

We consider two examples: first, diffusion of a messenger from a spine into the dendrite with emphasis on the local microdomain near the spine in the dendrite. Second, we compare 1D, 3D, and hybrid 1D-3D simulations of a propagating calcium wave in a 3D reconstruction of a CA1 pyramidal cell. Hybrid 1D-3D simulation reduces compute time by continuing to solve mostly 1D domains like dendrites using a simpler 1D approximation while preserving 3D details for non-linear areas like near the soma.

**Acknowledgments:** Research supported by NIH MH 086638.

**References**

1. Douglas J. Alternating direction methods for three space variables. *Numerische Mathematik* 1962 Dec 31;4(1):41–63.
2. Newton AJ, McDougal RA, Hines ML, Lytton WW. Using NEURON for reaction-diffusion modeling of extracellular dynamics. *Frontiers in neuroinformatics* 2018;12.
3. McDougal RA, Hines ML, Lytton WW. Water-tight membranes from neuronal morphology files. *Journal of neuroscience methods* 2013 Nov 15;220(2):167–78.

## P202 How do stimulus statistics change the receptive fields of cells in primary visual cortex?

### Ali Almasi^1^, Shi Sun^2^, Molis Yunzab^1^, Michael Ibbotson^1^, Hamish Meffin^1^

#### ^1^National Vision Research Institute, Melbourne, Melbourne, Australia; ^2^The University of Melbourne, Vision Science, Melbourne, Australia

##### **Correspondence:** Ali Almasi (aalmasi@student.unimelb.edu.au)

*BMC Neuroscience* 2019, **20(Suppl 1)**:P202

Our understanding of sensory coding in the visual system is largely derived from the use of basic stimuli (e.g. bars/gratings) to parameterize responses with a restricted range of stimulus parameters (e.g. orientation, spatial frequency etc.). Such techniques provide only partial descriptions of the full response functions. More recently, mathematical tools for comprehensively characterizing responses to arbitrary stimuli (e.g. white noise) have emerged, such as probabilistic frameworks where model estimation is performed by maximizing the likelihood of the recorded responses to particular stimuli. In this case, characterization is achieved by estimating the parameters of a receptive field (RF) model, which are typically a cascade of linear filters on the stimulus, followed by static nonlinearities that map the output of the linear filters to the neuronal spike rates (e.g. the general linear model). However, how much do these characterizations depend on the choice of the stimulus?

Here, we studied the changes that neuronal receptive field models undergo due to the change in the statistics of the visual input. We applied the nonlinear input model (NIM) to the recordings of single cells in cat primary visual cortex in response to white Gaussian noise (WGN) and natural scenes (NS), with a fixed global RMS contrast. We estimated for each cell the spatial filters constituting the neuronal RF, and their corresponding pooling mechanism. The NIM framework makes minimal assumptions about the underlying neuronal processing and is able to fit a diverse range of nonlinearities. The number of spatial filters for each cell was determined by performing cross-validation techniques over a test dataset. Fig. 1 shows model fits to an example cell under the two stimulation regimes.

**Fig. 1:**
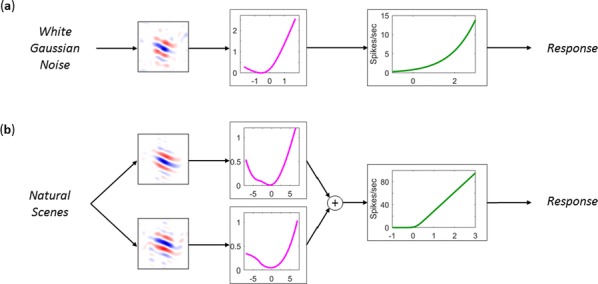
Schematic diagram of the nonlinear input model estimated for an example cell in cat primary visual cortex, using responses to **a** WGN and **b** NS stimuli. The estimated filters represent the neuronal spatial receptive field. The pink curves indicate the uncovered functions that are applied to the output of the filters, prior to pooling. The green curves are the estimated spiking nonlinearities

The most striking finding was that NS resulted in around twice as many significant filters as WGN. We also compared the identified RF filters under the two stimulation regimes in terms of preferred orientation and preferred bandwidths for spatial frequency, orientation and spatial frequency. Due to unequal numbers of RF filters, we computed and compared the mean of each of the above characteristics across RF filters within the same cell. The preferred orientation was highly preserved between filters estimated using WGN or NS, with a correlation coefficient (r) of 0.97. Other characteristics were preserved to a lesser degree: preferred spatial frequency (r = 0.73), orientation bandwidth (r = 0.53), and spatial frequency bandwidth (r = 0.45). Population analysis of the above characteristics revealed a statistically significant shift towards higher spatial frequency filters under the NS stimulation regime (t-test; p-value>0.99).

We compared the response function (i.e. function relating the filter’s output to the cell’s spike rate) of those cells that had the same number of spatial filters when stimulated by WGN and NS. We found profound differences in the relationship between the filter’s output and the cell’s spike rate: notably for gain, input scaling, output scaling, or a combination of all three. An interesting observation was that feature-contrast covered a wider range when using NS, which suggests that higher local contrast features embedded in the NS stimuli have a major impact on the recovered RF filters. A thorough investigation of the changes introduced in the response functions under different stimulation regimes are to be undertaken.

## P203 Extracellular spike waveform predicts whether single units recorded in visual cortex are tuned to orientation

### Shi Sun^1^, Ali Almasi^2^, Molis Yunzab^2^, Michael Ibbotson^2^, Hamish Meffin^2^

#### ^1^The University of Melbourne, Vision Science, Melbourne, Australia; ^2^National Vision Research Institute, Melbourne, Australia

##### **Correspondence:** Hamish Meffin (hmeffin@yahoo.com)

*BMC Neuroscience* 2019, **20(Suppl 1)**:P203

Extracellular spike waveforms from recordings in the visual cortex have been classified into either regular-spiking (RS) or fast-spiking (FS) units and are often associated with excitatory and inhibitory neurons, respectively. While both these types of spikes have waveforms with negative-going first-phases, we show that there are also distinct classes with positive-going first phases, which have not previously been reported. We estimated the spatial receptive fields (RFs) of these different spike waveform types and found that they have distinctly different structures.

237 single units were classified into five categories by the shape of their spike waveforms: RS units (33%, 78/237) which are biphasic, have a dominant negative peak, and a slow declining slope at the end of the waveform; FS units (32%, 75/237) which are biphasic, have a dominant negative peak, and a fast declining slope at the end of the waveform; triphasic spiking (TS) units (11%, 25/237) which have a positive first peak that is>10% of the negative peak, a large negative second peak and smaller positive third peak; compound spiking (CS) units (4%, 9/237) which are also triphasic but a significantly longer waveform; and positive spiking (PS) units (21%, 50/237) which have a dominant positive peak.

RFs were classified as either oriented and Gabor-like (orientation bandwidth <85°) or non-oriented and blob-like (orientation bandwidth >110°). PS units had mostly non-oriented RFs (65%, 24/37). RS and FS units had mostly oriented RFs (94%, 66/70; 99%, 71/72, respectively). TS and CS units are also mostly non-oriented RFs (56 %, 14/25; 75%, 6/8, respectively).

The non-oriented blob-like RFs are similar to the centre-surround RFs reported in the lateral geniculate nucleus. Thus, PS units may correspond to recordings from thalamic axons projecting to visual cortex, while the other spike types correspond to cortical neurons, which are orientation-tuned in the great majority of cases. This would allow cortically implanted electrodes to record activity from thalamus and cortex simultaneously.

## P204 Visual alpha generators in a spiking thalamocortical microcircuit model

### Renan O. Shimoura^1^, Antônio C. Roque^1^, Markus Diesmann^2^, Sacha van Albada^2^

#### ^1^University of São Paulo, Department of Physics, Ribeirão Preto, Brazil; ^2^Jülich Research Centre, Institute of Neuroscience and Medicine (INM-6) and Institute for Advanced Simulation (IAS-6), Jülich, Germany

##### **Correspondence:** Renan O. Shimoura (renanshimoura@usp.br)

*BMC Neuroscience* 2019, **20(Suppl 1)**:P204

The brain displays various oscillatory rhythms across scales that are related to one or multiple cognitive functions. One of the most prominent features in waking electroencephalograms of a variety of mammals, mainly observed at eyes-closed rest, is the alpha rhythm (around 10 Hz). This oscillation is observed in different areas of the cerebral cortex, standing out in occipitoparietal regions. Although alpha is strongly associated with reduced visual attention, it is also related to other functions such as the regulation of the timing and temporal resolution of perception, and transmission facilitation of predictions to visual cortex [1]. Understanding how and where this rhythm is generated can elucidate its functions. Even today there is no definitive answer on this question, though several hypotheses put forward the thalamus and the cortex as possible protagonists. In this work, two possible mechanisms responsible for alpha rhythm generation were studied: 1) pyramidal cortical neurons of layer 5 (L5) producing rhythmic bursts after stimulation by a short current pulse [2]; 2) a thalamocortical loop delay around 100 ms, as proposed before in mean-field models [3]. Here we investigate these hypotheses by implementing a full-scale computational model of a primary visual layered cortical microcircuit connected to a thalamic network, both containing excitatory and inhibitory neurons modeled by the adaptive exponential integrate-and-fire model (AdEx). The mechanisms were evaluated separately to isolate the role of each in generating the alpha rhythm. For hypothesis 1, the isolated cortical network was used and the AdEx model was adjusted so that the firing pattern resembled experimental data from a specific electrophysiological class of L5 neurons [2]. As observed experimentally, the addition of these neurons in the isolated L5 network was able to generate oscillations close to 10 Hz. To test hypothesis 2, thalamus was connected with its main cortical entry pathway to cortical layers 4 and 6. In turn, closing the thalamocortical loop, L6 neurons sent feedback connections to thalamus. Different combinations of thalamocortical and corticothalamic delays were evaluated adding up to the total delay mentioned. The results show alpha oscillations when the thalamocortical delay is sufficiently smaller than the corticothalamic one. Thus, both mechanisms potentially contribute to generating and sustaining the alpha rhythm in the thalamocortical network.

**Acknowledgements**: This work is part of the activities of FAPESP RIDC for Neuromathematics (Grant 2013/07699-0, S. Paulo Research Foundation). ROS is recipient of FAPESP scholarships: 2017/07688-9 and 2018/08556-1. ACR is partially supported by the CNPq fellowship Grant 306251/2014-0. ACR is also part of the IRTG 1740/TRP 2015/50122-0, funded by DFG/FAPESP. Supported by the European Union’s Horizon 2020 Framework Programme for Research and Innovation (grant 785907, Human Brain Project SGA2), the Jülich-Aachen Research Alliance (JARA), and DFG SPP 2041 “Computational Connectomics”.

**References**

1. Clayton MS, Yeung N, Cohen Kadosh R. The many characters of visual alpha oscillations. European *Journal of Neuroscience* 2018 Oct;48(7):2498-–508. 10.1111/ejn.13747
2. Silva LR, Amitai Y, Connors BW. Intrinsic oscillations of neocortex generated by layer 5 pyramidal neurons. *Science* 1991 Jan 25;251(4992):432–5. 10.1126/science.1824881
3. Roberts JA, Robinson PA. Modeling absence seizure dynamics: implications for basic mechanisms and measurement of thalamocortical and corticothalamic latencies. *Journal of theoretical biology* 2008 Jul 7;253(1):189–201. 10.1016/j.jtbi.2008.03.005

## P205 Neuronal avalanches in developing networks of Hawkes spiking neurons

### Sven Goedeke^1^, Felipe Yaroslav Kalle Kossio^2^, Raoul-Martin Memmesheimer^1^

#### ^1^University of Bonn, Neural Network Dynamics and Computation, Institute of Genetics, Bonn, Germany; ^2^University of Bonn, Institute for Genetics, Bonn, Germany

##### **Correspondence:** Sven Goedeke (sgoedeke@uni-bonn.de)

*BMC Neuroscience* 2019, **20(Suppl 1)**:P205

Neuronal avalanches are activity bursts with approximately power-law distributed sizes and durations. They have been observed on different scales in various neural systems. A possible explanation for their generation is that the underlying dynamics operate close to a critical state in which responses to small perturbations (or inputs) occur on all scales. Experimental studies indicate that neuronal avalanches may emerge during network development [e.g. 1, 2]. We present a simple model based on activity-dependent growth for developing networks of spiking neurons and demonstrate how these can “grow into” criticality [3]. Neurons spike stochastically with an instantaneous rate, which is excited by input spikes from other neurons as in a system of Hawkes processes. The growth mechanism is regulated homeostatically: If a neuron’s average spike rate is below a target rate, its neurites grow to increase the excitatory input. If its average spike rate is above the target rate, its neurites shrink.

Using mathematical analysis and simulations we show that our networks grow into a stationary state at which growth and shrinkage of neurites balance while the neurons are active at their target rates (Fig. 1). The characteristics of the stationary state are determined by the ratio of the target spike rate to the neurons’ spontaneous spike rate. If this ratio is large, every spike in the network causes on average almost one additional spike, and the network self-organizes into a nearly critical state (Fig. 1, rightmost column). Identifying the network’s total spiking dynamics with a self-exciting Hawkes process we analytically derive the size and duration distributions for nearly critical as well as subcritical states.

**Fig. 1:**
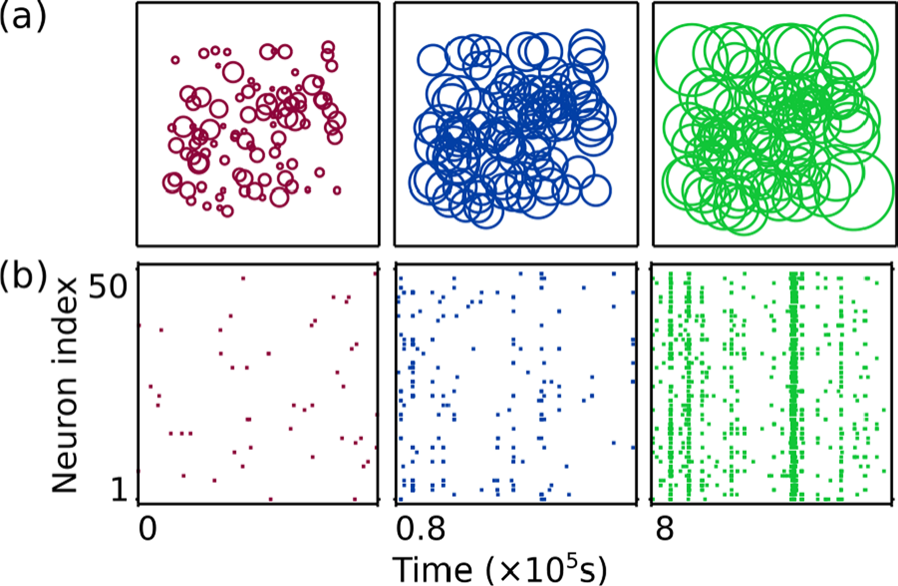
Network growth and activity dynamics (adapted from [3]). **a** Similar to previous models for network development extents of neurites are represented by disks arranged in a plane; their overlaps determine synaptic connection strength. Radii grow slowly and shrink upon spike generation. **b** Spiking activity during initial (red), growing (blue) and stationary state (green)

In neuroscientific experiments, avalanches may overlap and form complexes. The observer of an experiment will usually only have access to the latter. Using the duration distribution of single avalanches, we therefore compute the probability that avalanches overlap in our model as well as the duration distribution of avalanche complexes.

**Acknowledgments:** Supported by the German Federal Ministry of Education and Research (BMBF) via the Bernstein Network (Bernstein Award 2014, 01GQ1501 and 01GQ1710).

**References**

1. Hennig MH, Adams C, Willshaw D, Sernagor, E. Early-stage waves in the retinal network emerge close to a critical state transition between local and global functional connectivity. *Journal of Neuroscience* 2009, 29, 1077–1086.
2. Yada Y, et al. Development of neural population activity toward self-organized criticality. *Neuroscience* 2017, 343, 55–65.
3. Kossio FY, Goedeke S, van den Akker B, Ibarz B, Memmesheimer RM. Growing critical: self-organized criticality in a developing neural system. *Physical review letters* 2018 Aug 3;121(5):058301.

## P206 Quantifying transfer learning in mice and machines

### Ildefons Magrans de Abril, Doug Ollerenshaw, Marina Garrett, Peter Groblewski, Shawn Olsen, Stefan Mihalas

#### Allen Institute for Brain Science, Modelling, Analysis and Theory, Seattle, WA, United States of America

##### **Correspondence:** Stefan Mihalas (stefanm@alleninstitute.org)

*BMC Neuroscience* 2019, **20(Suppl 1)**:P206

Animals are considered to have a great capacity to generalize when learning a task, and transfer the learned information from a variant of the task to another, more so than standard machine learning methods. Here we systematically quantify the capacity of mice to transfer the learning of different stages of a task and compare it to several machine learning models. We used 24 mice in a standardized behavioural pipeline trained on go/no-go detection of change task (Fig. 1a). We quantify the speed of learning following a transition from oriented square-wave gratings to natural images. We compare the hit rate during the first few trials of the session prior to the change with the same number of trials in the following session. We observe a significant difference in hit distribution between the 30th and the 60th trial, indicating that mice are able to quickly transition to the new task (Fig. 1b).

**Fig. 1:**
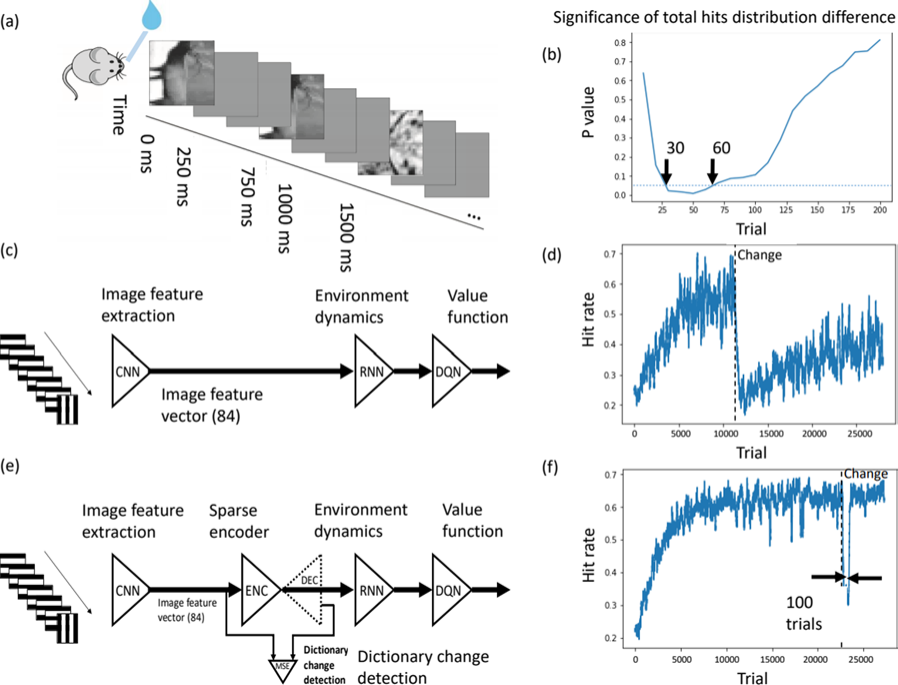
**a** Mice are presented a sequence of images (initially gratings, later natural images) and are trained to respond to identity changes. **b** Significance of hit distribution difference between the initial trials of the session prior and after transition. **c** The baseline model **d** recovers from transitions slowly. **e** New model with “higher order concept mapping” **f** recovers normal faster

We constructed a model of the computations involved, in which a pre-trained CNN (representing the visual processing stages) is followed by an RNN (which implements the memory) followed by a two-layer feedforward neural network acting as value function, trained with deep reinforcement learning (DQN). It matches well a number of existing models describing how reinforcement learning is realized in the brain (Fig. 1c). The transition between training phases of this model is significantly slower than the mice (~ 10000s trails; see Fig. 1d).

We hypothesize that the superior ability to transfer knowledge in animals is due to the ability to quickly recycle partial knowledge from past experiences. Similarly to how primates split visual processing between the “what” or ventral pathway associated with the ability to recognize an object and the “where” or dorsal pathway associated with the ability to reach that object, we suggest a first separation between environment representation and task control logic. Our model uses a sparse autoencoder trained to represent the input vector generated by the pre-trained CNN. The encoder translates an arbitrary feature vector to a very sparse encoding of “higher order concepts”. This code is sent to an RNN to capture the environment dynamic features which are finally forwarded to the DQN network. Encoder and decoder participate in the computation of a reconstruction error which is used to detect a change in the images used. The encoder acts as “firewall” by guaranteeing a constant encoding despite dictionary changes. When a change is detected, the RL algorithm stops and the autoencoder training proceeds to minimize the reconstruction error and to maximize the sparsity of the encoding. Assuming the dimensionality of the encoding vector is equal to the number of images in the dictionary, the task learned by the DQN network can be transferred to a new set of images by simply retraining the sparse autoencoder (Fig. 1e). We performed simulations with dictionaries of 2 images. Fig. 1f) shows that a few hundred trials suffice to detect and update the autoencoder weights which is two orders of magnitude quicker than the baseline model.

We hypothesise that computing predictions at intermediate stages, monitoring these predictions and using them to improve the local computations is a general organization principle of biological nervous systems, which would allow for rapid transfer of knowledge. Studying how they are built in biological structure can help us understand the biological systems and make better machine intelligence models.

## P207 Modeling and building a disinhibitory circuit in V1 that achieves context-switching

### Doris Voina^1^, Stefano Recanatesi^2^, Eric Shea-Brown^1^, Stefan Mihalas^3^

#### ^1^University of Washington, Department of Applied Mathematics, Seattle, WA, United States of America; ^2^University of Washington, Department of Physiology and Biophysics, Seattle, WA, United States of America; ^3^Allen Institute for Brain Science, Modelling, Analysis and Theory, Seattle, WA, United States of America

##### **Correspondence:** Doris Voina (dorisvoina@gmail.com)

*BMC Neuroscience* 2019, **20(Suppl 1)**:P207

We investigate minimally complex circuits that perform related but different tasks by using knowledge common to both tasks (transfer learning) and apply them to visual processing of images and visual processing of movies. Experimental evidence suggests that such a circuit exists in the brain’s visual area V1, where in addition to excitatory Pyr neurons and inhibitory SST neurons that interact with each other, an additional inhibitory VIP population enables switching between processing of different input types (static and moving). This circuit performs context-dependent computations, which means that Pyr neurons receive contextual information from the surround that could be different for the two input modalities and thus could lead to different neuronal interactions. Instead of requiring two circuits to solve the two separate tasks for visual processing, the brain processes scenes using a single network, where VIP neurons act as switching units that become activated as soon as the animals are running.

Could such a flexible circuit operate in both static and moving contexts to perform optimal processing? We use a model for optimal integration of context to predict neuronal connectivities (weights) that achieve optimal visual processing in the static and movement conditions separately. These weights are essentially lateral connections between Pyr and SST neurons that provide prior information about the (past) surround. As different input types can have very different statistics, whether the inputs are static or moving changes the value of the priors (and hence the value of weights). Instead of using these two different sets of weights in two separate circuits performing visual processing, we attempt to find one circuit where VIP neurons interact in a switch-like manner with the Pyr and SST populations. Here, Pyr and SST are connected by the weights found optimal in the static condition. To find a circuit with the capability of doing both visual processing tasks, we need to find the VIP contribution during movement that produces the same firing rate statistics that the set of optimal weights for the movement condition would generate.

We find that having feedforward connections (either additive or multiplicative) from VIP neurons to SST and Pyr is not enough to explain the firing rates statistics during movement. However, providing additional feedback connections from Pyr to VIP makes the circuit capable of reproducing firing rates for movement. Applying this network to natural images, we confirm that the network does optimal visual processing of images and, when the VIP units are active, optimal visual processing of videos. Our network predicts realistic connectivity patterns and firing rates of Pyr and other neural populations, while also finding the minimal number of VIP neurons that are necessary to still achieve an optimal switching circuit.

Using these findings, we are able to build such a circuit that achieves transfer learning by switching between different, but related, visual processing tasks. This provides a concrete example of an artificial neural network (ANN) that models a biological circuit—complete with cell type specifications—capable of performing multiple tasks, further paving the way for such bio-inspired ANNs.

## P208 Quantifying the dynamic effects of conceptual combination on word meanings using neural networks

### Nora E Aguirre-Celis^1^, Risto Miikkulainen^2^

#### ^1^ITESM and The University of Texas at Austin, Computer Sciences, Austin, TX, United States of America; ^2^The University of Texas at Austin, Computer Sciences, Austin, United States of America

##### **Correspondence:** Nora E Aguirre-Celis (naguirre@cs.utexas.edu)

*BMC Neuroscience* 2019, **20(Suppl 1)**:P208

How do people understand concepts such as bird, yellow bird, or early bird? The meaning of a concept depends largely on the concepts around it. While this hypothesis has existed for a long time, only recently it has become possible to test it based on neuroimaging and quantify it using computational modeling. Embodiment approaches to knowledge representation suggest that words are represented as a set of features that are the basic components of meaning. In particular, Binder et al. [1] grounded this idea by mapping semantic attributes to different brain networks and creating the Concept Attribute Representations (CAR) theory.

In CAR theory, words are represented as a set of weighted features stimulated by context. People weigh concept features based on context to construct a new representation specific to the combination of the concepts around it. For example, listeners understand that red apple is a fruit with certain color by selecting salient features that dominate the combination. Apple is defined by color, size, etc. and its color dimension is modified during conceptual combination.

This study focuses on the attribute combination process by quantifying such dynamic construction of concepts in the brain. Previous work showed (1) that words in different contexts have different representations, and (2) these differences are determined by context [2, 3]. These effects were demonstrated by analyzing individual sentence cases across multiple fMRI subjects (Fig. 1). The analysis was extended in this study to verify these same conclusions in the aggregate through a statistical analysis across an entire corpus of sentences and semantic roles. It measured how the CAR representation of a word changes in different sentences, and correlates these changes to the CAR representations of the other words in the sentence.

**Fig. 1:**
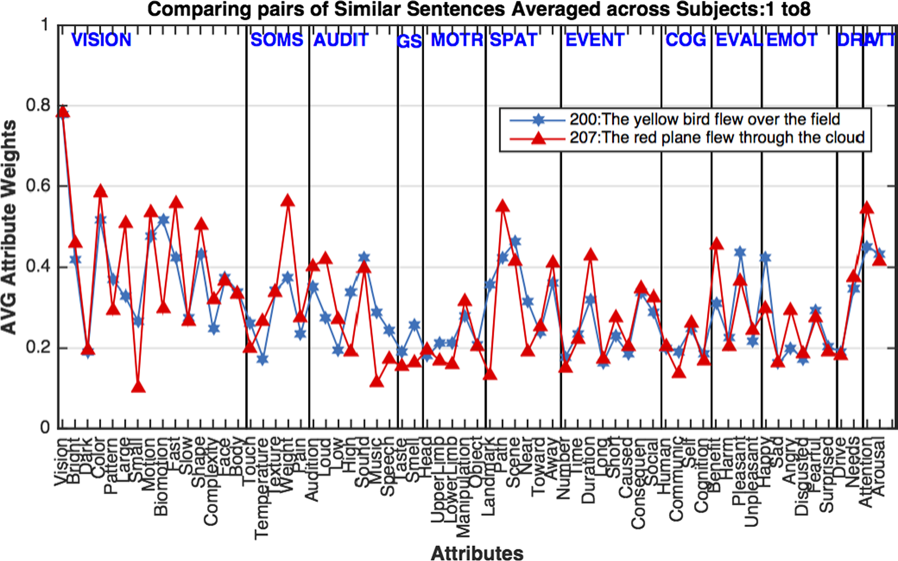
A detailed individual example of the conceptual combination effect. The conceptual combination effect in two sentences. In Sentence 200 (blue lines), the CAR representation modified by FGREP for the word flew has salient activations on animate features, presumably denoting bird properties like Small, Biomotion, Music, Smell and Taste, Pleasant and Happy. In Sentence 207 (red lines), it has high activations on inanimate object features, describing a Large, Fast, heavy Weight, and Loud object with some spatial and evaluation properties such as Path, Away, and Benefit which resembles a plane. The strengths of the attributes in the CARs changed according to context

The analysis was based on a neural network trained to map brain-based semantic representations of words (CARs) into fMRI data of subjects reading everyday sentences. Backpropagation was then repeated separately for each sentence, reducing the remaining error by modifying only the CARs at the input of the network (the FGREP method). As a result, the strengths of the attributes in the CARs changed according to context. The correlations are significantly higher for new CARs than for the original CARs across all subjects and all roles; the average correlation was 0.3201 for original CARs and 0.3918 for new CARs. Hence, the new CARs are more similar to the other words in the sentence than to the original CARs. These results indeed demonstrated that the effect is robust, and can be quantified by analyzing fMRI images through the FGREP mechanism. In the future such dynamic conceptual combination effects could be included in natural language processing systems by making the word embeddings susceptible to the semantic meanings that humans truly perceive.

**Acknowledgments:** J. Binder, R. Raizada, and M. Aguilar. Supported in part by IARPA-FA8650-14-C-7357 and by NIH 1U01DC014922 grants.

**References**

1. Binder J, Desai R, Graves W, et al. Where is the semantic system? *Cereb. Cortex* 2009, vol. 19, pp. 2767–2769.
2. Aguirre-Celis N, Miikkulainen R. From Words to Sentences&Back. *Annual Meeting of the Cog. Sc. Society, London, UK*. 2017, 1513–1518.
3. Aguirre-Celis N, Miikkulainen R. Combining fMRI Data and NN to Quantify Context Effects in the Brain. Brain Informatics. *Lecture Notes in Computer Science. Springer* 2018, 11309:129–140.

## P209 On-line decoding of attempted movements from source reconstructed potentials.

### Timothée Proix^1^, Christoph Pokorny^2^, Martin Seeber^1^, Stephanie Trznadel^3^, Andrea Galvez^2^, David D’Croz-Baron^4^, Aude Yulzari^3^, Simon Duboue^2^, Ruxandra Iancu-Ferfoglia^5^, Ammar Kassouha^5^, Karin Diserens^6^, Anne-Chantal Heritier Barras^5^, Jean-Paul Janssens^5^, Ujwal Chaudhary^7^, Niels Birbaumer^7^, Christoph Michel^2^, Tomislav Milekovic^2^

#### ^1^University of Geneva, Departement des Neurosciences Fondamentales, Geneve, Switzerland; ^2^University of Geneva, Geneva, Switzerland; ^3^Wyss Center for Bio and Neuroengineering, Geneva, Switzerland; ^4^Texas Tech University, Lubbock, United States of America; ^5^University Hospital Geneva, Geneva, Switzerland; ^6^Vaud University Hospital, Lausanne, Switzerland; ^7^University of Tübingen, Tübingen, Germany

##### **Correspondence:** Timothée Proix (timothee.proix@unige.ch)

*BMC Neuroscience* 2019, **20(Suppl 1)**:P209

People with locked-in syndrome lose the ability to effectively communicate due to the loss of limb movement and capacity to talk. This condition has a drastic impact on their quality of life, entailing emotional, social and financial costs. Restoring independent communication for people with locked-in syndrome remains a challenging clinical problem with no viable solution. Brain-computer interfaces (BCIs) can help them regain independence by providing the ability to control communication interfaces, interact with ICT technologies (brain-controlled computer cursor). Non-invasive BCIs have been used to restore communication to people with paralysis. Yet, the communication rates were low and BCIs required frequent (daily) recalibration by highly skilled engineers. We designed a method that aims to achieve stable performance of EEG-based BCIs using reconstructed activity of sources from the cortical motor regions. Subjects performed a delayed instructed movement task, where each movement instruction was followed by a go cue after a fixed delay. We reconstructed activity of motor cortical sources from high-density EEG recordings made while the subjects performed the task. We then calibrated a regularized linear discriminant analysis decoder on movement-related cortical potentials (MRCPs), and event-related desynchronization and synchronization (ERDS) responses in the beta frequency bands. Our decoder successfully detects feet, wrist and finger movements in an asynchronous test scenario. These results open the way for developing efficient, stable and user-friendly communication BCI using high-density EEG and source reconstructed responses for people with locked-in syndrome.

**Acknowledgements**: This study was supported by the Swiss National Science Foundation Ambizione grant (PZ00P2_168103), Wings for Life Foundation, Friedrich Flick Förderungsstiftung, Bertarelli Foundation and the Wyss Center for Bio- and Neuroengineering.

## P210 Nonlinear functional co-activations: dynamical, directed and delayed.

### Ignacio Cifre^1^, Jeremi Ochab^2^, Dante Chialvo^3,4^, Tatiana Miller^4^

#### ^1^Universit Ramon Llull, Barcelona, Spain; ^2^Jagiellonian University, Institute of Physics, Kraków, Poland; ^3^Universidad Nacional de San Martín, Buenos Aires, Argentina; ^4^Center for Complex Systems & Brain Sciences (CEMSC^3), Buenos Aires, Argentina

##### **Correspondence:** Ignacio Cifre (ignaciocl@blanquerna.url.edu)

*BMC Neuroscience* 2019, **20(Suppl 1)**:P210

Linear Pearson correlation, is the most frequently used framework in most of the research about functional and dynamic functional connectivity between brain areas in task conduced and in resting state studies. An alternative, introduced in previous work [1, 2], emphasized that the relatively stronger Blood Oxygen Level Dependent (BOLD) activations contain most of the information relevant to brain functional connectivity. Here we study the correlation properties of these relatively strong BOLD activations investigating novel features that further characterize the brain functional and dynamical connectivity. These include characteristics of the brain network, such a) directionality in the influence between areas, resulting in non-symmetric correlations; b) temporal latency of the events, i.e., a sequence of activations across brain regions; c) detection of negative correlations between areas, focusing in relevant high and low amplitude events; and d) the possibility of using this method on task conducted studies by considering the input signal in the chain of events.

As a test-bed of the method, here we use the Autism ABIDE database [3], which is an open database of fMRI with more than a thousand resting state scans of patients (AU) and healthy subjects (HS). Our method [2, 1] starts by selecting from each BOLD time-series those events which surpass a given threshold of intensity (usually 1 SD), defining a vector than contains few points before and after this trigger (usually ~ 8-15 seconds). The average value of these vectors for each time-series (termed source) is stored with the mean signal of all other voxels’ time-series evaluated at the same times (termed target). Further calculations used these vectors in order to compute the correlation between source and target (Fig. 1A), its direction (Fig. 1B), its temporal delay (Fig. 1C), etc.

**Fig. 1:**
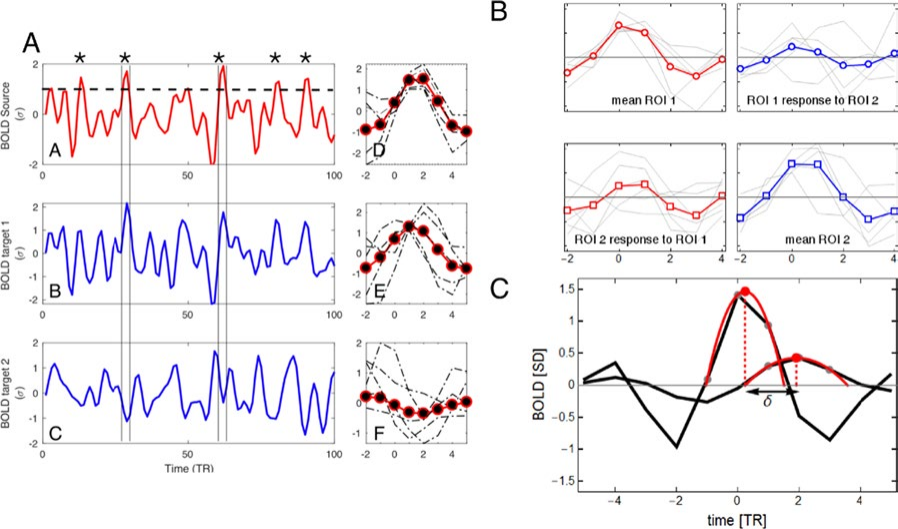
**a** Selection method for the relevant events; **b** Representation of asymmetry calculation; **c** Representation of delay calculation

Here we replicated the findings reported in [4] in which HS showed higher correlation between ventral agranular insula L and Precuneus L and R, and revealed that identical results are obtained by correlation from the relevant strong events. In addition, we find a clear asymmetry in the direction of the co-activations between Insula and Fusiform. The computation of the delays of the events shows that AU have higher delays between the events of Insula and Precuneus. These results show the ability of our method for inspecting relevant events going beyond the classical measures of resting state functional connectivity.

**Acknowledgements**: Work supported by the MICINN (Spain) grant PSI2017-82397-R, the NSC (Poland) grant DEC-2015/17/D/ST2/03492, and by CONICET (Argentina) and Escuela de Ciencia y Tecnología, UNSAM.

**References**

1. Tagliazucchi E, Balenzuela P, Fraiman D, Chialvo DR. Criticality in large-scale brain fMRI dynamics unveiled by a novel point process analysis. *Frontiers in physiology* 2012 Feb 8;3:15.
2. Tagliazucchi E, Balenzuela P, Fraiman D, Montoya P, Chialvo DR. Spontaneous BOLD event triggered averages for estimating functional connectivity at resting state. *Neuroscience letters* 2011 Jan 20;488(2):158–63.
3. Chen H, Nomi JS, Uddin LQ, Duan X, Chen H. Intrinsic functional connectivity variance and state‐specific under‐connectivity in autism. *Human brain mapping* 2017 Nov;38(11):5740–55.
4. Xu J, Wang H, Zhang L, Xu Z, Li T, Zhou Z, Zhou Z, Gan Y, Hu Q. Both Hypo-Connectivity and Hyper-Connectivity of the Insular Subregions Associated With Severity in Children With Autism Spectrum Disorders. *Frontiers in neuroscience* 2018 Apr 11;12:234.

## P211 A co-evolving model for synaptic pruning

### Ana Paula Millán Vidal

#### University of Granada, Granada, Spain

##### **Correspondence:** Ana Paula Millán Vidal (apmillan@ugr.es)

*BMC Neuroscience* 2019, **20(Suppl 1)**:P211

Most real-world networks present non-trivial topological features, such as high clustering and short minimum paths, a modular structure or cost-efficient wiring [1]. Remarkably, the evolution of the topology is typically linked to the activity state and vice-versa, giving rise to a co-evolving or adaptive network. This interplay between form and function affects the emerging behavior of the system in a critical way, and certain dynamic phenomena appear repeatedly: the formation of complex topologies, robust dynamical self-organization, and complex mutual dynamics in both activity and topology. Here we propose an adaptive network model inspired in synaptic pruning. This consists in the extensive elimination of synapses during infancy [2], and it plays a major role in the development of the mammalian brain. It is believed to provide an optimum compromise between network efficiency and the amount of energy it consumes, whereas minimizing the amount of genetic information needed. Recent studies have also pointed at its implications on high-level brain functions, and its relation with the emergence of some neurological disorders such as autism and schizophrenia [3].

Given that synaptic growth and death depend on neural activity, here we propose a biologically inspired co-evolving model for neural network development which couples a classical attractor neural network with a preferential attachment model for network evolution that reproduces experimental profiles of synaptic density [4]. This coupling gives rise to a feedback loop between activity and structure that strongly increases noise tolerance and ensues the existence of a region of bistability between heterogeneous networks that are capable of memory, and homogeneous ones goberned by noise. Moreover, the inclusion of a transient time of high connectivity, as it occurs during brain development, enhances the memory capabilities of the system. Interestingly, there is an optimal intermediate connectivity leading to efficient networks with minimum energy consumption, so that it is not necessary -and may in fact be detrimental- to start out with an overly connected network. Finally, depending on parameters there appear oscillations among different memories, reminding mind dynamical processes, which originate due to the destabilization of memory attractors due to synaptic pruning.

These results could explain why experimental pruning curves present their characteristic temporal profiles and, eventually, anomalies such as autism and schizophrenia associated, respectively, with a deficit or an excess of pruning. The basic mechanism illustrated here is not restricted to neural networks, but may play a role in shaping other systems, such as protein interaction networks. In fact, almost all biological networks change in time, so pruning may be a general mechanism for network optimization in an environment of limited resources.

**References**

1. Eguiluz VM, Chialvo DR, Cecchi GA, Baliki M, Apkarian AV. Scale-free brain functional networks. *Physical review letters* 2005 Jan 6;94(1):018102.
2. Iglesias J, Eriksson J, Grize F, Tomassini M, Villa AE. Dynamics of pruning in simulated large-scale spiking neural networks. *Biosystems* 2005 Jan 1;79(1–3):11–20.
3. Faludi G, Mirnics K. Synaptic changes in the brain of subjects with schizophrenia. *International Journal of Developmental Neuroscience* 2011 May 1;29(3):305–9.
4. Millán AP, Torres JJ, Johnson S, Marro J. Concurrence of form and function in developing networks and its role in synaptic pruning. *Nature communications* 2018 Jun 8;9(1):2236.

## P212 A theoretical approach to intrinsic timescales in spiking neural networks

### Alexander van Meegen, Sacha van Albada

#### Jülich Research Centre, Institute of Neuroscience and Medicine (INM-6) and Institute for Advanced Simulation (IAS-6), Jülich, Germany

##### **Correspondence:** Alexander van Meegen (a.van.meegen@fz-juelich.de)

*BMC Neuroscience* 2019, **20(Suppl 1)**:P212

Cortical neural dynamics unfolds over multiple timescales. Beyond a simple heterogeneity of timescales, in vivo electrophysiological recordings in the resting state reveal a hierarchical structure that matches anatomical hierarchies remarkably well [1]. This structure could arise either from systematic variations in single-neuron properties or from the intricate network structure. We focus on the latter and investigate intrinsic timescales, characterized by single-unit autocorrelation times, in spiking neural network models with biologically constrained connectivity [2, 3].

For networks of rate units, dynamic mean field theory (DMFT) has yielded significant insights into the interrelation between network structure and intrinsic timescales [4, 5]. In a first step, DMFT reduces the dynamics of the recurrent network to a set of self-consistent stochastic differential equations. Technically, the starting point of the theory is the system’s characteristic functional and it proceeds with a disorder average, a Hubbard-Stratonovich transformation, and a saddle point approximation. Although mathematically involved, the result is intuitive: The massive recurrent input to each neuron is replaced by an effective stochastic process. In a second step, the self-consistency problem has to be solved. To this end, the output statistics of a neuron driven by anon-Markovian Gaussian process have to be calculated. The full non-Markovian problem has to be considered because a Markovian approximation neglects the quantity of interest: the temporal correlations. For sufficiently simple rate neurons, the problem is analytically solvable [4, 6]. However, for spiking neuron models, this is an open challenge. In the low firing rate regime where the mean inter-spike interval exceeds the correlation time of the input, a renewal approximation is admissible. A renewal process is fully characterized by its hazard function, i.e. its instantaneous firing rate given that no previous firing occurred. Thus, we derive a novel approximation for the hazard function of a leaky integrate-and-fire neuron driven by a non-Markovian Gaussian process. This enables us to obtain a closed system of self-consistent equations for the autocorrelation functions of single neurons. From the autocorrelation functions we can finally obtain the intrinsic timescales (Fig. 1).

**Fig. 1:**
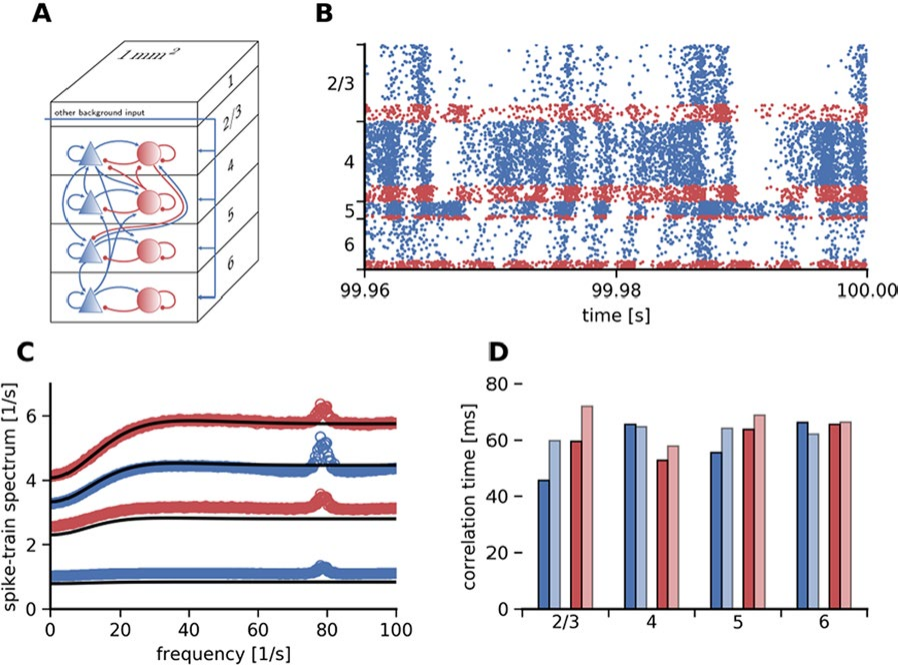
Sketch of the spiking neural network model (**a**, figure adapted from [2]). A raster plot **b** shows asynchronous irregular dynamics. Spike-train power spectra obtained from our theory (**c**, black lines) agree well with simulations (**c**, colored symbols). Accordingly, predicted intrinsic timescales (**d**, light bars) are also in good agreement with simulations (**d**, solid bars)

Establishing a direct link between the connectivity and the emergent intrinsic timescales allows for a thorough investigation of the effect of network architecture. From a modeler’s point of view, such mechanisms could be used to fine-tune network models to match the experimentally observed hierarchy of timescales. Focusing on computational aspects, diverse time scales strongly enhance the computational capacity of a recurrent network [7].

**Acknowledgments:** Supported by the European Union’s Horizon 2020 Framework Programme for Research and Innovation under Specific Grant Agreement785907 (Human Brain Project SGA2), the Jülich-Aachen Research Alliance (JARA), and DFG Priority Program “Computational Connectomics”(SPP 2041).

**References**

1. Murray JD, Bernacchia A, Freedman DJ, et al. A hierarchy of intrinsic timescales across primate cortex. *Nature neuroscience* 2014 Dec;17(12):1661.
2. Potjans TC, Diesmann M. The cell-type specific cortical microcircuit: relating structure and activity in a full-scale spiking network model. *Cerebral cortex* 2012 Dec 2;24(3):785–806.
3. Schmidt M, Bakker R, Hilgetag CC, Diesmann M, van Albada SJ. Multi-scale account of the network structure of macaque visual cortex. *Brain Structure and Function* 2018 Apr 1;223(3):1409–35.
4. Sompolinsky H, Crisanti A, Sommers HJ. Chaos in random neural networks. *Physical review letters* 1988 Jul 18;61(3):259.
5. Schuecker J, Goedeke S, Helias M. Optimal sequence memory in driven random networks. *Physical Review X* 2018 Nov 14;8(4):041029.
6. van Meegen A, Lindner B. Self-Consistent Correlations of Randomly Coupled Rotators in the Asynchronous State. *Physical review letters* 2018 Dec 20;121(25):258302.
7. Bellec G, Salaj D, Subramoney A, Legenstein R, Maass W. Long short-term memory and learning-to-learn in networks of spiking neurons. *In Advances in Neural Information Processing Systems* 2018 (pp. 787–797).

## P213 Pontine mechanisms of abnormal breathing

### William Barnett^1^, Lucas Koolen^2^, Thomas Dick^3^, Ana Abdala^2^, Yaroslav Molkov^1^

#### ^1^Georgia State University, Department of Mathematics & Statistics, Atlanta, GA, United States of America; ^2^University of Bristol, School of Physiology, Pharmacology & Neuroscience, Biomedical Sciences Faculty, Bristol, United Kingdom; ^3^Case Western Reserve University, School of Medicine, Department of Medicine, Cleveland, United States of America

##### **Correspondence:** William Barnett (wbarnett2@gsu.edu)

*BMC Neuroscience* 2019, **20(Suppl 1)**:P213

Rett syndrome (RTT) is a neurodevelopmental disorder that includes pyramidal and extrapyramidal motor system dysfunction. Individuals with RTT breathe abnormally having apnea, apneusis, hyperventilation and irregular respiratory rate. The neurological mechanism of breathing dysrhythmia is not fully understood, but accumulating evidence indicates that disruption of inhibitory transmission in the pontine nuclei mediates disrupted respiration. For example, the blockade of GABAAor 5-HT1Areceptors in the Kӧlliker-Fuse nuclei (KFn) induces periodic apnea in rats and mice. Augmentation of inhibition in the KFn by application of a 5-HT1Areceptor agonist or the blockade of GABA reuptake suppresses periodic apneas in mouse models of RTT. Here, we use mathematical modeling to investigate the role of inhibition in the pontine respiratory circuitry as well as the physiological consequences of perturbations to the pontine inhibitory tone.

The respiratory central pattern generator (rCPG) is a neuronal network in the mammalian medulla that provides the timing and amplitude of descending respiratory drive to respiratory-modulated motoneurons. This neuronal circuitry is distributed across the ventrolateral respiratory column (VRC). During normal breathing neurons in these nuclei produce activity, which results in inspiratory motor output to the diaphragm. Under increased ventilatory drive (*e.g.*high pCO2) an additional population of neurons is recruited in the parafacial respiratory group (pFRG) to activate abdominal muscles during expiration. Interestingly, manipulating inhibitory tone in the KFn modulates the emergence of this activity. Weak activation of GABAAreceptors with low doses of isoguvacine in the KFn facilitates abdominal expiratory activity during hypercapnia, and activation of 5-HT1Areceptors in the KFn induces active expiration during normocapnia. Consistent with inhibition facilitating expiratory motor activity, weak dis-inhibition of the KFn with gabazine, a GABAAreceptor antagonist, suppresses expiration during hypercapnia.

We extend our established computational model of the respiratory central pattern generator by including a more detailed pontine circuit. This model is consistent with the experimental results produced by manipulation of GABA and 5HT1Areceptor-mediated transmission. We propose that a glutamatergic KFn population provides excitation to the expiratory neurons in VRC that in turn inhibit the expiratory oscillator in pFRG. The activity of KFn neurons is under external or local GABAergic inhibitory control. With reduced inhibition, a subpopulation of neurons in the KFn produces seizure-like activity periodically. During these seizures, KFn drives expiratory activity in VRC to periodically produce central apnea characteristic to RTT. When inhibition in the KFn is strong and the output of the KFn is weak, this ‘inhibitory’ balance in the KFN reduces the drive to post-inspiratory neurons of the Bötzinger Complex, which dis-inhibits pFRG population, allowing it to be active at eucapnic CO2levels. Integrating these results, we suggest that periodic apnea and modulation of active expiration are products of specific interactions between the medullary and pontine structures determined the excitatory-inhibitory balance in the KFn. Moreover, our modeling results support the concept that inhibitory receptors in the KFn are potential therapeutic targets to treat periodic apnea and abnormal emergence of active expiration.

## P214 Interlimb coordination during split-belt locomotion: a modeling study

### Elizaveta Latash^1^, William Barnett^2^, Robert Capps^1^, Simon Danner^3^, Ilya Rybak^3^, Yaroslav Molkov^1^

#### ^1^Georgia State University, Department of Mathematics & Statistics, Atlanta, GA, United States of America; ^2^Georgia State University, Georgia State University, Atlanta, GA, United States of America; ^3^Drexel University College of Medicine, Department of Neurobiology and Anatomy, Philadelphia, PA, United States of America

##### **Correspondence:** Elizaveta Latash (elizaveta.latash@gmail.com)

*BMC Neuroscience* 2019, **20(Suppl 1)**:P214

The rhythmic movement of limbs during locomotion is controlled by central pattern generators (CPGs)–neural networks that can produce rhythmic output in the absence of rhythmic inputs. These locomotor CPGs are located in the spinal cord and each limb is controlled by a separate CPG. Thus, interlimb coordination depends on the activity of the CPGs and interactions between them. The rhythm generated by the locomotor CPG network depends on the endogenous dynamics of CPG cells, interaction between these cells, supraspinal inputs to the CPGs, and sensory feedback.

The dynamics of limb coordination has been recently experimentally investigated with spinalized cats walking on a split-belt treadmill [1]. The speed of each belt was varied in a tied- or split-belt fashion while recording the continuous transformation of walking gait. In tied-belt experiments, with an increase of belt speed the duration of the swing phase of stepping remained relatively constant, whereas the duration of the stance phase decreased. Moreover, as belt speed increased, the walking gait takes on an antiphase pattern in which the swing phases on each side of the body were separated by epochs of dual support when both left and right limbs were on the ground. In split-belt experiments, the belt speed was fixed on one (slower) side and increased on the other (faster) side. In these experiments, the walking pattern was perturbed in an asymmetric fashion. As belt speed on the fast side increased, the cycle duration of steps on the slow side generally did not change. However, on the fast side the duration of the stance phase decreased. At high ratios of belt speeds the cats produced multiple steps on the fast side for each step on the slow side. In such 1-to-N gaits, the first swing phase on the fast side had greater duration than subsequent swing phases. In both 1-to-1 and 1-to-N gaits, split-belt experiments revealed an asymmetric mode of synchronization in which the stance phase on the slow side was initiated at the time when stance on of fast side ended. In this manner, the swing phase on the slow side was immediately succeeded by the swing phase on the fast side without an interceding interval of dual support.

We adopted these experimental results to elucidate the potential neuronal mechanisms of interlimb coordination using a tractable mathematical model of interacting locomotor CPGs. Our model contains two pairs of flexor and extensor neuronal populations mutually inhibiting each other representing two interacting CPGs. The endogenous dynamics of rhythmic activity in these populations was governed by the slow inactivation of a persistent sodium current. The oscillation frequency of each CPG is controlled by an external drive implemented as an excitatory synaptic conductance in a flexor population. Each flexor population received inhibition from the contralateral flexor and extensor populations. The split-belt locomotion was simulated by setting different frequencies of oscillations in left and right CPGs. The model could reproduce and proposed explanation to the above experimental results as the consequence of both specific organization of the circuits mediating interactions between CPGs on the left and right sides of the cord and dynamical properties of the CPGs.

**Reference**

1. Frigon A, Desrochers É, Thibaudier Y, Hurteau MF, Dambreville C. Left–right coordination from simple to extreme conditions during split‐belt locomotion in the chronic spinal adult cat. *The Journal of physiology* 2017 Jan 1;595(1):341–61.

## P215 Sympathetic and parasympathetic mechanisms of enhanced respiratory modulation of blood pressure and heart rate during slow deep breathing

### Robert Capps^1^, William Barnett^1^, Elizaveta Latash^2^, Erica Wehrwein^3^, Thomas Dick^4^, Yaroslav Molkov^2^

#### ^1^Georgia State University, Atlanta, GA, United States of America; ^2^Georgia State University, Department of Mathematics & Statistics, Atlanta, GA, United States of America; ^3^Michigan State University, Physiology, East Lansing, United States of America; ^4^Case Western Reserve University, School of Medicine, Department of Medicine, Cleveland, United States of America

##### **Correspondence:** Robert Capps (rocapp@gmail.com)

*BMC Neuroscience* 2019, **20(Suppl 1)**:P215

Human health and wellbeing are maintained by numerous homeostatic processes mediated by the cardiorespiratory systems. These processes do not operate independently. In fact, coupling between respiratory and autonomic processes may be a biomarker of health. Interactions between cardiovascular and respiratory systems are evident in sympathetic and parasympathetic nerve activities. The sympathetic nervous system regulates vascular tone and controls blood flow, and its activity is modulated by respiration. Vagal nerve activity regulates the heart rate, and the cardiac vagal preganglionic neurons of the nucleus ambiguous are modulated by respiration. Physiologically these systems interact to control tissue oxygenation. Individually, the cardiovascular and respiratory systems are well studied, but less is known about the neural mechanisms that underlie their interaction.

Previously, we reported that slow deep breathing (SDB) evoked an increase in the respiratory modulation of blood pressure. Respiratory modulation of the blood pressure could emerge from modulation of the heart rate via the parasympathetic nervous system. This respiratory modulation of the heart rate is known as the respiratory sinus arrhythmia (RSA), which is characterized as the tendency of the heart rate to increase during inspiration and decrease during expiration. In the published SDB dataset, the magnitude of RSA increased significantly during SDB. We applied our computational model to test whether the increased RSA was sufficient to explain an enhanced respiratory modulation of the blood pressure. We found that the baseline RSA— prior to SDB— could be replicated by choosing an appropriate combination of modulatory inputs from respiratory neurons to the nucleus ambiguous. The enhanced RSA during SDB could be reproduced by an increase in activity of the respiratory neurons modulating the parasympathetic tone to the heart. However, the parasympathetic modulation alone was not sufficient to reproduce either the baseline or the enhanced blood pressure oscillations. To reproduce the Traube-Hering waves successfully, our model required respiratory input to the pre-sympathetic populations, which enabled both the experimentally observed baseline and the amplified respiratory modulation of blood pressure during SDB.

In summary, we considered the effects of slow deep breathing on the respiratory modulation of the heart rate and blood pressure. In addition to the previously identified enhancement of the blood pressure modulation, we found that the respiratory modulation of the heart rate is comparably increased during slow deep breathing. Using computational modeling we inferred the parasympathetic and sympathetic mechanisms of these effects. Results from our modeling suggest that the enhanced heart rate modulation can be explained by increased inputs from the respiratory neurons to nucleus ambiguous. However, the enhanced modulation of the heart rate alone does not explain the respiratory modulation visible in the blood pressure traces. Based on model simulations, we propose that blood pressure oscillations at the respiratory frequency originate from respiratory modulation of the pre-sympathetic neuronal activity in ventrolateral medulla. The amplification of these oscillations during slow deep breathing is associated with longer respiratory phases and/or stronger inputs from the respiratory neurons.

**Acknowledgments:** Supported by NIH grantsR01AT008632 and U01EB021960.

## P216 Pairwise models inferred from hippocampal data generate states typical of low-dimensional attractors

### Lorenzo Posani^1^, Simona Cocco^1^, Rémi Monasson^1,2^

#### ^1^Ecole Normale Supérieure, Physics, Paris, France; ^2^CNRS, Paris, France

##### **Correspondence:** Lorenzo Posani (lorenzo.posani@gmail.com)

*BMC Neuroscience* 2019, **20(Suppl 1)**:P216

Max-entropy (Ising) models allow for probabilistic modeling of neural population activity directly from data. Recently, approaches based on max-entropy assumptions have been applied to retinal, cortical, and hippocampal recordings, showing that they can provide powerful tools for characterizing and decoding the neural state [2, 3]. An important feature of these statistical models is that novel neural patterns can be generated by sampling from the corresponding probability distribution. A crucial question concerns the generative power of these models, i.e., how the generated patterns relate to the functionality of neural activity for the cognitive task. In the case of the Ising model, it is unclear which observables matter functionally, so the question of whether these statistical models are generative or not for neural data remains open.

Here, we address this issue in the case of hippocampal place cells activity recorded during open navigation. Place cells encode for the spatial location of the rat, as well as for contextual information; therefore, in the case of place-cells, functionality has a precise meaning regarding the spatial correlate of the activity. We infer an Ising model from reference CA3 data of [1], recorded during free exploration of a familiar environment. We then generate activity patterns by sampling from the inferred model, and test them on their spatial correlate.

We first show that generated patterns are novel yet statistically coherent with real data, see Fig. 1a. To assess the functionality (spatial selectivity) of these patterns we use a Bayesian decoder to retrieve a position in the environment from each pattern s of the generated session. The standard deviation of the spatial posterior P(x,y | s), measured in cm, is used as a proxy for the dispersion of the bump of activity, while the distance between consecutive decoded positions quantifies the coherence of the virtual trajectory in time. As shown in Fig. 1b, both bump dispersion and time-coherence are comparable in generated vs. real data. Moreover, the forced activation of a single neuron, in the model, resulted in a clustered activation of cells that have nearby place fields in the environment, effectively pinning the activity bump at the corresponding position (Fig. 1c), coherently with theoretical attractor models [3].

**Fig. 1:**
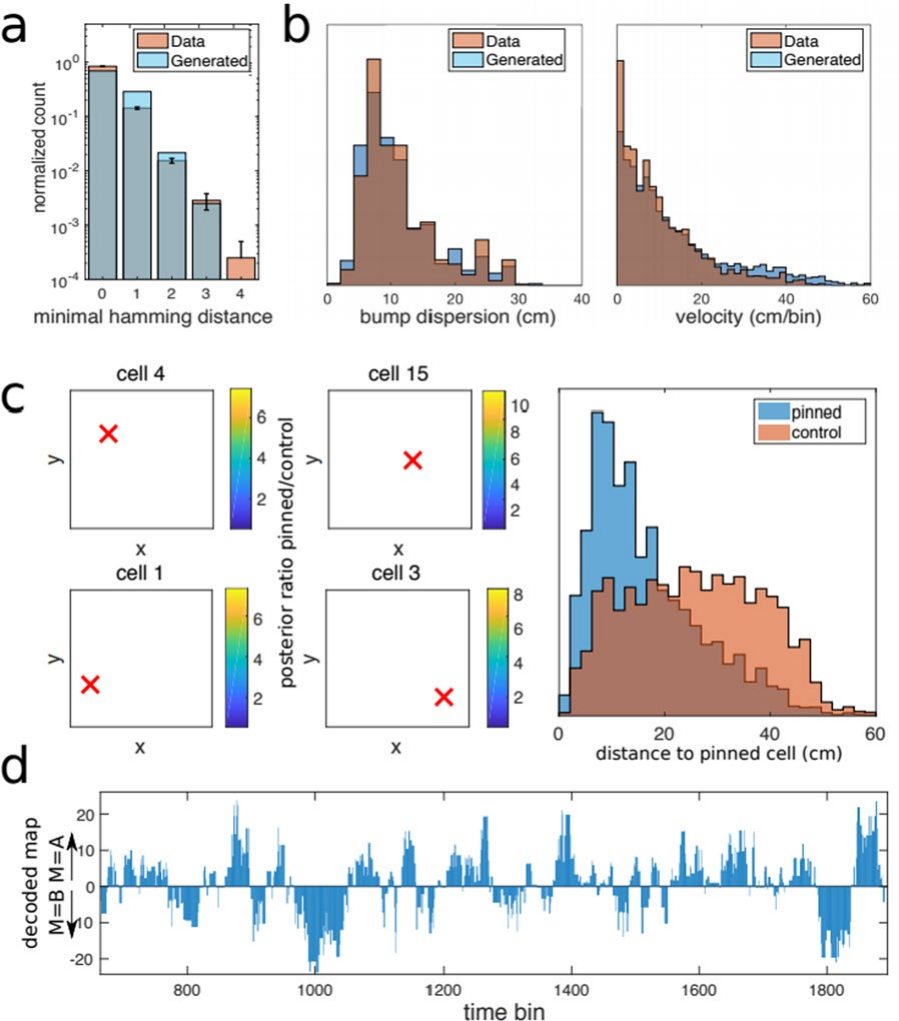
**a** Likelihood consistence and novelty (hamming distance>0) of Ising-generated vs. real data. **b** same comparison in term of positional variance and speed **c** pinning test on generated data **d** cognitive-map oscillations in data generated by a single model inferred from data recorded from two cognitive maps

We then perform the same analysis on a model inferred from the joint collection of patterns from two distinct open environments. Strikingly, this model generates bimodal activity configurations, coding for well-defined position in one of the two environments only, with spontaneous stochastic transitions from one map to the other (Fig. 1d), recalling the flickering oscillations observed in the “teleportation” session of the original training data [1].

Combined, these findings show that Ising-generated activity configurations are meaningful, in that they code for positions of the “simulated” animal in the familiar environment. Therefore, the inferred model captures and preserves the fundamental functional relation between neurons. These results highlight the tight relation between functionality and dimensionality reduction in the hippocampal cognitive map.

**References**

1. Jezek K, Henriksen EJ, Treves A, Moser EI, Moser MB. Theta-paced flickering between place-cell maps in the hippocampus. *Nature* 2011 Oct;478(7368):246.
2. Posani L, Cocco S, Ježek K, Monasson R. Functional connectivity models for decoding of spatial representations from hippocampal CA1 recordings. *Journal of Computational Neuroscience* 2017 Aug 1;43(1):17–33.
3. Posani L, Cocco S, Monasson R. Integration and multiplexing of positional and contextual information by the hippocampal network. *PLoS computational biology* 2018 Aug 14;14(8):e1006320.

## P217 Information transfer in modular spiking networks

### Barna Zajzon^1,2^, Renato Duarte^1,2^, Abigail Morrison^2^

#### ^1^Jülich Research Center, Institute of Neuroscience and Medicine (INM-6), Jülich, Germany; ^2^Jülich Research Centre, Institute for Advanced Simulation (IAS-6), Jülich, Germany

##### **Correspondence:** Barna Zajzon (b.zajzon@fz-juelich.de)

*BMC Neuroscience* 2019, **20(Suppl 1)**:P217

Topographic maps, comprising ordered projections among distinct neuronal populations, form an important component of the brain’s anatomical repertoire. Particularly prominent in sensory systems, such connectivity scheme has the potential to underlie important functional roles, ranging from information segregation and transmission to spatiotemporal feature aggregation. While the anatomical feature itself has been the subject of many studies, its computational significance remains relatively unexplored.

Here, we study the features enabling the reliable transfer of information across multiple recurrent, spiking neural network modules, tuned to operate in a balanced, asynchronous irregular regime. We exploit the complex input-driven dynamics that such networks exhibit during active processing and probe the systems’ computational proficiency with simple tasks. We show that, in a sequential setup, structured projections between the modules are strictly necessary for information to propagate to sufficient depths. Such mapping is shown to not only improve computational performance and efficiency, but also reduce response variability, increase robustness against noise and interference effects, and boost memory capacity. This suggests that, while random projections might be sufficient for communication between a few populations, signal propagation over longer distances and across several modules requires topographic precision for accurate, robust and reliable transmission.

Given that topographic specificity is assumed to decrease with hierarchical depth in cortical networks, we explore how the variation in the specificity of topographic projections influences the systems’ properties by manipulating key structural parameters such as modularity, map size and degree of overlap. Doing so, we identify a condition where the global population statistics converges towards a stable asynchronous irregular regime, the networks exhibit denoising properties and the overall discrimination capability improves with hierarchical depth. These results extend the relevance of topographic precision and suggest that it plays an important role in the control and modulation of population responses towards computationally advantageous regimes.

We further investigate the ability of the modular circuit to extract, integrate and propagate information from two concurrent input streams in a more complex, nonlinear fashion. Using the XOR task, we demonstrate that it is more advantageous to perform computations locally, within a given module, and subsequently transfer the results downstream than to transfer intermediate information and perform the computation downstream. Additionally, depending on how the input streams are mixed in the early processing stages, the networks employ different strategies to encode information with similar accuracy.

Apart from demonstrating and quantifying the functional benefits of topographic projections, the results and insights gained from this work can shed new light on important requirements for designing biologically inspired, functional hierarchical spiking networks.

## P218 Modelling the micro-structure of the mouse whole-neocortex connectome

### Michael Reimann, Michael Gevaert, Ying Shi, Huanxiang Lu, Henry Markram, Eilif Muller

#### École Polytechnique Fédérale de Lausanne, Blue Brain Project, Switzerland

##### **Correspondence:** Michael Reimann (michael.reimann@epfl.ch)

*BMC Neuroscience* 2019, **20(Suppl 1)**:P218

An understanding of the structure of synaptic connectivity in the brain is fundamental and necessary to ultimately understand brain function. Yet, the relationship between macro-scale connectomics—the study of connectivity between regions—and ultimately their implementation at the micro-scale—the motifs, rules and principles for how connectivity manifests at the neuronal level—remain largely unexplored. We have combined these two complementary views of connectomics to build a first draft statistical model of the neuron-to-neuron micro-connectome of a whole mouse neocortex.

We started by analyzing two recently published high quality datasets: The whole brain axon reconstructions of Janelia Mouselight [2], and the Allen Institute Mouse Meso-Connectome [3, 4]) to model in addition to the meso-scale trends also the innervation of individual neurons by individual axons, within and across regions. In doing so, we have identified, parameterized and validated new principles underlying the topographical mapping of connectivity between regions and the efferent long-range connectivity of individual neurons.

By combining the principles with an openly available cell atlas [1] we generated statistical connectomes between all ~10 million neurons in the mouse neocortex at sub-cellular resolution. These connectomes are made openly available to the community, and provide a powerful null model, i.e. a quantitative context in which experimental results can be understood and appreciated, for example retrograde tracer experiments (Fig. 1). The connectome instances can also serve as the basis of large-scale simulations of neuronal activity, ranging from mean-field to point-neuron or even morphologically detailed models, and allowing the study for example of emergent EEG dynamics or hierarchical interactions in cortex.

**Fig. 1:**
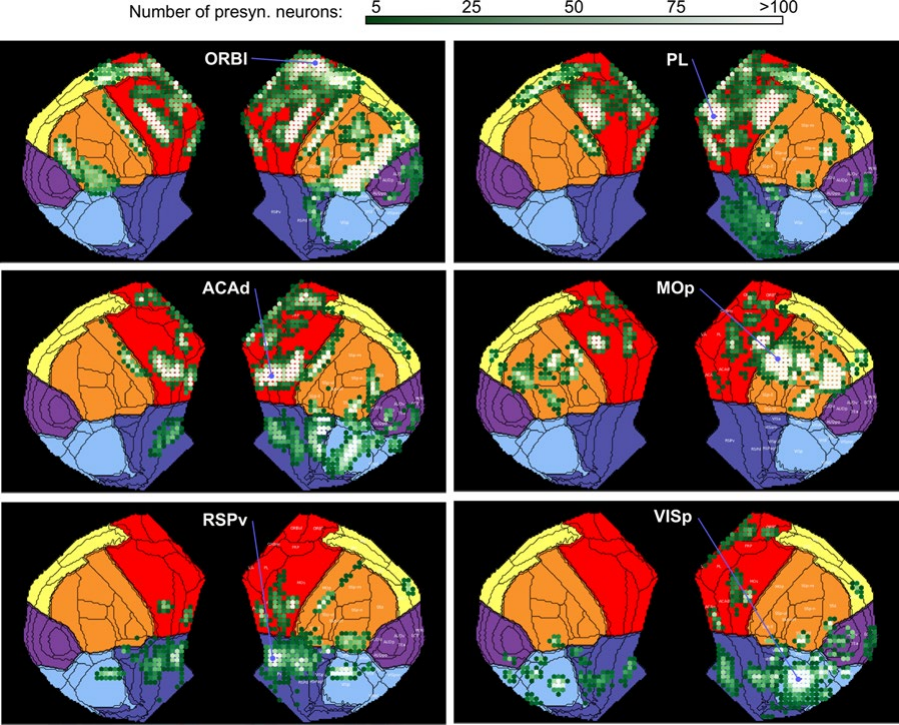
In-silico retrograde tracer experiments. Locations and numbers of neurons innervating around 100 neurons in a small volume (1.8·10^−4^ mm^3^) at the indicated locations in various representative brain regions

**References**

1. Erö C, Gewaltig MO, Keller D, Markram H. A cell atlas for the mouse brain. *Frontiers in neuroinformatics* 2018;12:84.
2. Gerfen CR, Economo MN, Chandrashekar J. Long distance projections of cortical pyramidal neurons. *Journal of neuroscience research* 2018 Sep;96(9):1467–75.
3. Harris JA, Mihalas S, Hirokawa KE, et al. The organization of intracortical connections by layer and cell class in the mouse brain. *BioRxiv* 2018 Jan 1:292961.
4. Oh SW, Harris JA, Ng L, et al. A mesoscale connectome of the mouse brain. *Nature* 2014 Apr;508(7495):207.

## P219 Impact of higher-order network structure on emergent cortical activity

### Max Nolte^1^, Michael Reimann^1^, Eyal Gal^2^, Henry Markram^1^, Eilif Muller^1^

#### ^1^École Polytechnique Fédérale de Lausanne, Blue Brain Project, Lausanne, Switzerland; ^2^The Hebrew University of Jerusalem, Edmond and Lily Safra Center for Brain Sciences, Jerusalem, Israel

##### **Correspondence:** Max Nolte (max.nolte@epfl.ch)

*BMC Neuroscience* 2019, **20(Suppl 1)**:P219

Synaptic connectivity between neocortical neurons is highly structured, including first-order structure, such as strengths of connections between different neuron types and distance-dependent connectivity, and higher-order structure, such as an abundance of cliques of all-to-all connected neurons and small-world topology. The relative impact of first- and higher-order structure on emergent cortical network activity is unknown. Here, we compared network topology and emergent activity in two neocortical microcircuit models with different null-models of synaptic connectivity, both with similar first-order structure, but with higher-order structure arising from morphological diversity within neuronal types removed in one model. We found that morphological diversity within neuronal types creates heterogeneous degree distributions with hub neurons, raises in-degrees at the bottom of layer six, contributes to the abundance of cliques, and increases small-world topology. The increase in higher-order network structure was accompanied by more nuanced changes in neuronal firing patterns, including increased activity and response reliability at the bottom of layer six. Without morphological diversity, the dependence of pairwise correlations on the positions of neurons in directed cliques was strongly reduced. Our study shows that circuit models with very similar first-order structure of synaptic connectivity can have a drastically different higher-order network topology, and that the higher-order topology imposed by morphological diversity within neuronal types has a clear impact on emergent activity.

## P220 Biophysical modeling of LTP and LTD in the somatosensory cortex

### Giuseppe Chindemi^1^, Vincent Delattre^1^, Michael Doron^2^, Michael Graupner^3^, James King^1^, Pramod Kumbhar^1^, Rodrigo Perin^4^, Srikanth Ramaswamy^1^, Michael Reimann^1^, Christian A Rössert^1^, Werner Alfons Hilda Van Geit^1^, Idan Segev^2^, Henry Markram^1^, Eilif Muller^1^

#### ^1^École Polytechnique Fédérale de Lausanne, Blue Brain Project, Lausanne, Switzerland; ^2^Hebrew University of Jerusalem, Department of Neurobiology, Jerusalem, Israel; ^3^Université Paris Descartes, Laboratoire de Physiologie Cérébrale - UMR 8118, CNRS, Paris, France; ^4^École Polytechnique Fédérale de Lausanne, Laboratory of Neural Microcircuitry, Lausanne, Switzerland

##### **Correspondence:** Giuseppe Chindemi (giuseppe.chindemi@epfl.ch)

*BMC Neuroscience* 2019, **20(Suppl 1)**:P220

Long-term potentiation (LTP) and long-term depression (LTD) of synaptic responses are often thought to be among the fundamental building block of learning and memory in the brain. These plastic changes are largely heterogeneous in the neocortex, even within the same region. Due to the complexity of the experimental procedures involved, a complete map of LTP and LTD dynamics is still missing. It has been shown in vitro that such heterogeneity could be explained, at least in part, by the specificity of synaptic innervation rather than of plastic mechanisms [1, 2]. In this work, we tested the previous hypothesis by integrating in vitro data on LTP and LTD at layer 5 thick-tufted pyramidal cell connections [2, 3] into a morphologically detailed model of somatosensory cortex [4]. Our results suggest that indeed innervation specificity could account for a large portion of the available experimental evidence at different connection types [5-8]. We then generalized our results to the non-experimentally characterized connection types, obtaining a first predicted map of LTP and LTD in the somatosensory cortex.

**References**

1. Froemke RC, Poo MM, Dan Y. Spike-timing-dependent synaptic plasticity depends on dendritic location. *Nature* 2005 Mar;434(7030):221.
2. Sjöström PJ, Häusser M. A cooperative switch determines the sign of synaptic plasticity in distal dendrites of neocortical pyramidal neurons. *Neuron* 2006 Jul 20;51(2):227–38.
3. Markram H, Lübke J, Frotscher M, Sakmann B. Regulation of synaptic efficacy by coincidence of postsynaptic APs and EPSPs. *Science* 1997 Jan 10;275(5297):213–5.
4. Markram H, et al. Reconstruction and simulation of neocortical microcircuitry. *Cell* 2015 Oct 8;163(2):456–92.
5. Egger V, Feldmeyer D, Sakmann B. Coincidence detection and changes of synaptic efficacy in spiny stellate neurons in rat barrel cortex. *Nature neuroscience* 1999 Dec;2(12):1098.
6. Rodríguez-Moreno A, Paulsen O. Spike timing–dependent long-term depression requires presynaptic NMDA receptors. *Nature neuroscience* 2008 Jul;11(7):744.
7. Zilberter M, et al. Input specificity and dependence of spike timing–dependent plasticity on preceding postsynaptic activity at unitary connections between neocortical layer 2/3 pyramidal cells. *Cerebral Cortex* 2009 Feb 4;19(10):2308–20.
8. Banerjee A, et al. Distinct mechanisms of spike timing‐dependent LTD at vertical and horizontal inputs onto L2/3 pyramidal neurons in mouse barrel cortex. *Physiological reports* 2014 Mar 1;2(3).

## P221 Realistic models of molecular layer inhibitory interneurons of the cerebellar cortex

### Martina Francesca Rizza^1^, Stefano Masoli^1^, Egidio D’Angelo^1^, Francesca Locatelli^1^, Francesca Prestori^1^, Diana Sánchez Ponce^2^, Alberto Muñoz^2^

#### ^1^University of Pavia, Department of Brain and Behavioural Sciences, Pavia, Italy; ^2^Universidad Politécnica, Centro de Tecnología Biomédica, Madrid, Spain

##### **Correspondence:** Martina Francesca Rizza (marti.rizza@gmail.com)

*BMC Neuroscience* 2019, **20(Suppl 1)**:P221

Stellate (SC) and basket cells (BC) are inhibitory interneurons located in the molecular layer (ML) of the cerebellum. These cells receive excitatory inputs from parallel fibers and their branched axons make synapses onto Purkinje cells (PC), providing feed-forward inhibition. SCs make synaptic contacts with PC dendrites, while BCs with PC soma and axon initial segment (AIS). Although these interneurons are generated by the same progenitor and, during their migration, are morphologically indistinguishable, they achieve different localization in the ML. SCs are placed at the top of the ML, whereas BCs are located in the deepest part [1].To investigate SC and BC electrophysiological properties, we elaborated multi-compartmental biophysically realistic models in Python-NEURON (Python 2.7; NEURON 7.5) [2]. 3D morphologies of mouse neurons were reconstructed into Neurolucida format from fluorescent images obtained with a confocal microscope. The morpho-electrical equivalents, composed of dendritic tree, soma, AIS and axon, were reconstructed with NEURON. Ionic channels were located on the morphological compartments according to immunohistochemistry [3] and their gating kinetics were modeled following the HH formulation or Markov-chains [4]. The maximum ionic conductances (Gi-max) were tuned to match the firing pattern revealed by electrophysiological recordings in mice cerebellar slices using patch-clamp techniques. SC and BC discharges elicited by step current injections were used as templates to extract the features needed to assess the fitness function for optimization. Gi-max tuning was performed by automatic parameter estimation algorithms, using multi-objective genetic algorithm [5] implemented in Blue Brain Python Optimization Library (BluePyOpt) [6]. The optimized models reproduce the spontaneous firing of both cells and the firing patterns in response to positive and negative current injections. Cellular responses to synaptic activity modulation and to gap junction synchronization (in the case of SCs), will be investigated to study the electrophysiological microcircuit dynamics. The optimization technique gave satisfactory results, reproducing interneurons biophysical properties. The model provided a valuable tool to examine the neuronal function involved in cerebellar network activity.

**Acknowledgments:** The research has received funding from the EU Horizon 2020 under the Specific Grant Agreement No. 720270 (HBP SGA1) and 785907 (HBP SGA2).

**References**

1. Pouzat C, Hestrin S. Developmental regulation of basket/stellate cell→ Purkinje cell synapses in the cerebellum. *Journal of Neuroscience* 1997 Dec 1;17(23):9104–12.
2. Hines M, Davison AP, Muller E. NEURON and Python. *Frontiers in neuroinformatics* 2009 Jan 28;3:1.
3. Masoli S, Solinas S, D’Angelo E. Action potential processing in a detailed Purkinje cell model reveals a critical role for axonal compartmentalization. *Frontiers in cellular neuroscience* 2015 Feb 24;9:47.
4. D’Angelo E, Nieus T, Maffei A, et al. Theta-frequency bursting and resonance in cerebellar granule cells: experimental evidence and modeling of a slow k+-dependent mechanism. *Journal of Neuroscience* 2001 Feb 1;21(3):759–70.
5. Druckmann S, Banitt Y, Gidon AA, Schürmann F, Markram H, Segev I. A novel multiple objective optimization framework for constraining conductance-based neuron models by experimental data. *Frontiers in neuroscience* 2007 Oct 15;1:1.
6. Van Geit W, Gevaert M, Chindemi G, et al. BluePyOpt: leveraging open source software and cloud infrastructure to optimise model parameters in neuroscience. *Frontiers in neuroinformatics* 2016 Jun 7;10:17.

## P222 Excitability differences between small and large sensory nerve fibers may be explained by different ion channel distribution

### Jenny Tigerholm, Aida H. Poulsen, Ole K. Andersen, Carsten D. Mørch

#### Aalborg university, Department of Health Science and Technology, Aalborg, Denmark

##### **Correspondence:** Jenny Tigerholm (tige@kth.se)

*BMC Neuroscience* 2019, **20(Suppl 1)**:P222

During neuropathy, the excitability of cutaneous nerve fibers is altered, which has been associated with voltage-gated ion channels abnormalities. It is technically difficult to measure the excitability in nerve fibers, particularly in small fibers. However, the perception threshold tracking technique can be used to indirectly assess the excitability in nerve fibers. Small surface electrodes (Pin electrodes) have been developed to preferentially activate small fibers with superficial innervation while conventional surface electrodes (Patch electrodes) activate large fibers around perception threshold. The aim of this study was to develop a computational model in order to understand how subtypes of voltage-gated ion channels influence the excitability of nerve fibers.

A two-part computational model has been developed in order to study nerve fiber activation by cutaneous electrical stimulation. The first part of the model is a 3D finite element model, calculating the electrical field generated by a cutaneous electrode. A pin electrode and a patch electrode were developed in COMSOL version 5.2. The skin model consists of four rectangular layers: stratum corneum, epidermis, dermis, and adipose tissue. In order to understand how the electrical field influences the nerve fibers, two multi-compartment nerve fiber models were developed in the simulator environment NEURON (version 7.6). Two different fibers were implemented: one large fiber (length: 5.0 cm, diameter: 9 µm, compartments: 27 120) and one small fiber (length: 5.5 cm, diameter: 3.5 µm, compartments: 25 940). The small fiber model terminated in the middle of the epidermis whereas the large fibers model terminated in the middle of the dermis. The axon models included the voltage-gated ion channels: Nav1.6-Nav1.9, KDr, KM, KA and HCN channel. Experimentally assessed perception threshold of the strength-duration relationship, threshold electrotonus, and slowly increasing pulse forms were set as constraints for the two nerve fiber models.

The computational model reproduced the experimentally assessed perception thresholds for the three protocols; the strength-duration relationship, the threshold electrotonus, and the slowly increasing pulse forms. The small nerve fiber model showed a higher increase in activation threshold for shorter square pulses compared to the large nerve fiber model. This is consistent with the experimental results showing a significantly higher time constant for small nerve fibers (computational model: 1170 µs, experiment obtained: 1060 µs ± 690 µs) than for large nerve fibers (computational model = 420 µs, experiment obtained: 580 µs ± 160 µs). For long hyperpolarizing prepulses (duration>30 ms), the small nerve fiber model had a substantial increase in the activation threshold compared to the large nerve fiber model. The computational model showed that difference in excitability between the two fiber types could be explained by the different distribution of TTX sensitive- and TTX resistant sodium channels as well as the M-current.

In conclusion, assessments of perception thresholds using the three protocols may be an indirect measurement of the membrane excitability, and computational models may have the possibility to link voltage-gated ion channel activation to perception threshold measurements.

**Acknowledgements**: Funded by Center for Neuroplasticity and Pain (CNAP). CNAP is supported by the Danish National Research Foundation (DNRF121).

## P223 A new spectral graph model of brain oscillations

### Ashish Raj, Xihe Xie, Chang Cai, Pratik Mukherjee, Eva Palacios, Srikantan Nagarajan

#### University of California San Francisco, Radiology and Biomedical Imaging, San Francisco, CA, United States of America

##### **Correspondence:** Ashish Raj (ashish.raj@ucsf.edu)

*BMC Neuroscience* 2019, **20(Suppl 1)**:P223

One of the most important questions in computational neuroscience is how the brain’s structural wiring gives rise to its function and its patterns of activity. Although neural mass models that involve large coupled non-linear differential equations have previously been proposed for capturing these structure-function relationships, they are unwieldy, difficult to compute, and are only accessible via simulations. Here we present for the first time a linear and analytically accessible model that incorporates local oscillations and long-range connectivity, fully accounts for the axonal conductance delays, and obtains closed form equations for each of the graph Laplacian’s eigen-modes. The proposed model incorporates all frequencies of oscillations, is fast and in a closed form, given by the eigen-modes of the graph Laplacian. Since the model does not require spatially-varying model parameters, its success could imply that network topology alone is capable of producing spatial patterning in brain activity.

## P224 Controlling burst activity allows for a multiplexed neural code in cortical circuits

### Filip Vercruysse^1^, Henning Sprekeler^1^, Paola Suárez^1^, Richard Naud^2^

#### ^1^Technical University Berlin, Berlin, Germany; ^2^University of Ottawa, Ottawa, Canada

##### **Correspondence:** Filip Vercruysse (vercruysse@tu-berlin.de)

*BMC Neuroscience* 2019, **20(Suppl 1)**:P224

The existence of specialized mechanisms for burst generation in pyramidal cells (PCs) suggests that bursts are likely to be an important temporal feature of neural signals. Bursts appear to be correlated with sensory processing and perception [1], are effective inducers of synaptic plasticity [2], and have been proposed as a cellular mechanism to combine external and internal information [3]. In layer 5 PCs, bursts occur at a low, but consistent rate, and are thought to arise from active dendritic processes. Because burst activity relies on dendritic threshold mechanisms [4], it appears likely that low burst activity requires an intricate homeostatic control. We hypothesized that this control is mediated by inhibitory plasticity of connections from Martinotti cells, which are known to control dendritic activity [5]. To investigate this hypothesis, we studied a computational network model comprising layer 5 pyramidal cells with a somatic and dendritic compartment that was fitted to in vitro data [6], as well as different classes of interneurons. Our results show that a simple Hebbian plasticity rule on inhibitory synapses leads to robust and self- organized control of dendritic and burst activity. The dendritic learning rule we propose is inspired by a homeostatic rule that was previously proposed to control somatic spiking activity [7] and therefore inherits properties such as a balance of excitation and inhibition. We demonstrate that this E/I balance is necessary for realistic burst firing patterns in biologically inspired cortical microcircuits with inhibitory neurons and recurrent connections. Furthermore, we show that the self-organized control of somatic and dendritic activity in pyramidal cells enables a multiplexed burst code suggested recently [6], by alleviating the need to tune input or noise levels. Finally, we show in simulations that the self-organising properties of inhibitory plasticity rules can be used to multiplex sensory and decision-related signals in decision-making networks [8], allowing us to decode behavioral decisions from burst activity in populations of sensory neurons.

**References**

1. Takahashi N, Oertner TG, Hegemann P, Larkum ME. Active cortical dendrites modulate perception. *Science* 2016 Dec 23;354(6319):1587–90.
2. Sjostrom PJ, Rancz EA, Roth A, Hausser M. Dendritic excitability and synaptic plasticity. *Physiological reviews* 2008 Apr;88(2):769–840.
3. Larkum M. A cellular mechanism for cortical associations: an organizing principle for the cerebral cortex. *Trends in neurosciences* 2013 Mar 1;36(3):141–51.
4. Larkum ME, Zhu JJ, Sakmann B. A new cellular mechanism for coupling inputs arriving at different cortical layers. *Nature* 1999 Mar;398(6725):338.
5. Murayama M, Pérez-Garci E, Nevian T, Bock T, Senn W, Larkum ME. Dendritic encoding of sensory stimuli controlled by deep cortical interneurons. *Nature* 2009 Feb;457(7233):1137.
6. Naud R, Sprekeler H. Sparse bursts optimize information transmission in a multiplexed neural code. *Proceedings of the National Academy of Sciences* 2018 Jul 3;115(27):E6329–38.
7. Vogels TP, Sprekeler H, Zenke F, Clopath C, Gerstner W. Inhibitory plasticity balances excitation and inhibition in sensory pathways and memory networks. *Science* 2011 Dec 16;334(6062):1569–73.
8. Wimmer K, Compte A, Roxin A, Peixoto D, Renart A, De La Rocha J. Sensory integration dynamics in a hierarchical network explains choice probabilities in cortical area MT. *Nature communications* 2015 Feb 4;6:6177.

## P225 Reinforcement-mediated plasticity in a spiking model of the drosophila larva olfactory system

### Anna-Maria Jürgensen^1^, Afshin Khalili^1^, Martin Paul Nawrot^2^

#### ^1^University of Cologne, Cologne, Germany; ^2^University of Cologne, Computational Systems Neuroscience, Institute of Zoology, Cologne, Germany

##### **Correspondence:** Anna-Maria Jürgensen (a.juergensen@uni-koeln.de)

*BMC Neuroscience* 2019, **20(Suppl 1)**:P225

The mushroom body is a center for the integration of sensory input and reinforcement information in insects [1]. In Drosophila larva it consist of less than two hundred neurons [2], yet the animal is able to identify and learn about odors and their context to guide behavior [3]. This spiking model is based on a generic insect model [4] and takes advantage of the recently released full synaptic connectome of the mushroom body [2]. Leaky integrate-and-fire neurons with an emphasis on biologically realistic parameters for neurons and synaptic connectivities are modeled in Brian2. Sparse coding has been demonstrated a key feature of insect sensory systems. In our model, temporal sparseness is achieved through spike frequency adaptation at the cellular level, while population sparseness is caused by lateral inhibition [6] and feedback inhibition via the anterior paired lateral neuron [7]. Reinforcement-mediated plasticity between the intrinsic and the output neurons of the mushroom body is sufficient to account for fundamental features of memory formation. The model performs well in one-trial and multiple-trial learning, solely based on plasticity at a single synaptic site (Fig. 1). It captivates with its exact implementation of the network size and the connectivities [2] along with the biological plausibility of the neuron parameters and the naturalistic behavior of cells including spike frequency adaptation [8].

**Fig. 1:**
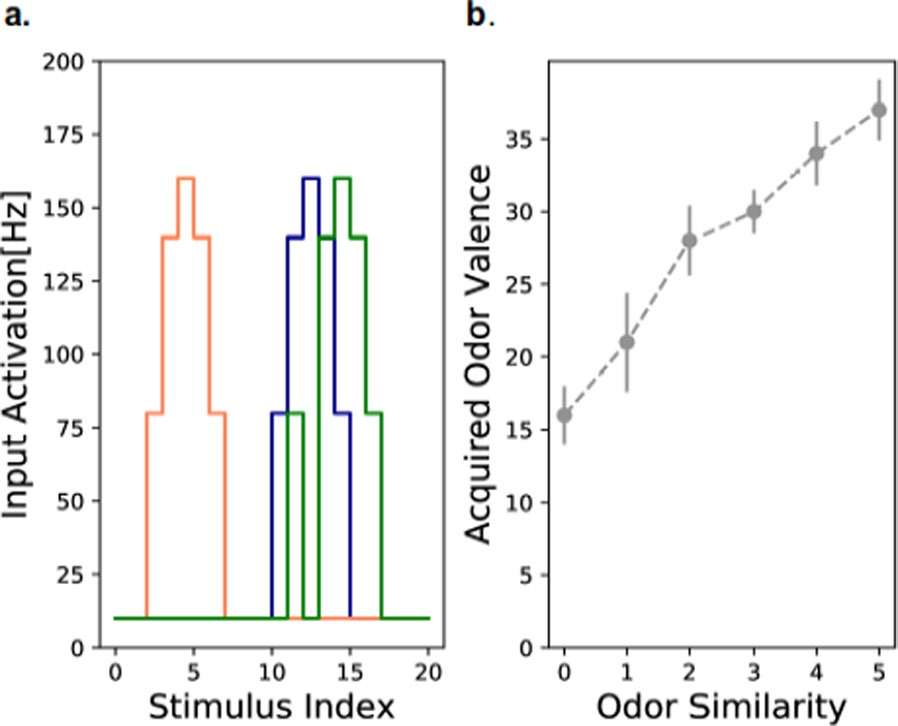
Absolute odor conditioning in Drosophila larva. **a**, exemplary virtual odors used as stimuli during training. Input activation of different sets of receptors as denoted by the stimulus index. Overlap codes similarity of odors. **b**, acquired odor valence depends on the similarity of tested with trained odor for n = 10 independent experiments

**Acknowledgements**: This research was funded by the DFG through FOR 2705 (grant no. 403329959) “Dissection of a Brain Circuit: Structure, Plasticity and Behavioral Function of the Drosophila mushroom body”

**References**

1. Heisenberg M. Mushroom body memoir: from maps to models. *Nature Reviews Neuroscience* 2003 Apr;4(4):266.
2. Eichler K, Li F, Litwin-Kumar A, et al. The complete connectome of a learning and memory centre in an insect brain. *Nature* 2017 Aug;548(7666):175.
3. Schleyer M, Fendt M, Schuller S, Gerber B. Associative learning of stimuli paired and unpaired with reinforcement: evaluating evidence from maggots, flies, bees, and rats. *Frontiers in psychology* 2018;9.
4. Betkiewicz RL, Nawrot MP, Lindner B. Circuit and cellular mechanisms facilitate the transformation from dense to sparse coding in the insect olfactory system. *bioRxiv* 2017 Jan 1:240671.
5. Farkhooi F, Froese A, Muller E, Menzel R, Nawrot MP. Cellular adaptation facilitates sparse and reliable coding in sensory pathways. *PLoS computational biology* 2013 Oct 3;9(10):e1003251.
6. Schmuker M, Pfeil T, Nawrot MP. A neuromorphic network for generic multivariate data classification. *Proceedings of the National Academy of Sciences* 2014 Feb 11;111(6):2081–6.
7. Lin AC, Bygrave AM, De Calignon A, Lee T, Miesenböck G. Sparse, decorrelated odor coding in the mushroom body enhances learned odor discrimination. *Nature neuroscience* 2014 Apr;17(4):559.
8. Demmer H, Kloppenburg P. Intrinsic membrane properties and inhibitory synaptic input of kenyon cells as mechanisms for sparse coding? *Journal of neurophysiology* 2009 Sep;102(3):1538–50.

## P226 Inhibitory clustering and adaptation: critical features in modeling the neocortex

### Vahid Rostami^1^, Thomas Rost^1^, Alexa Riehle^2^, Sacha van Albada^3^, Martin Paul Nawrot^1^

#### ^1^University of Cologne, Computational Systems Neuroscience, Institute of Zoology, Cologne, Germany; ^2^CNRS - Aix-Marseille Université, Institut de Neurosciences de la Timone (INT), Marseille, France; ^3^Jülich Research Centre, Institute of Neuroscience and Medicine (INM-6) and Institute for Advanced Simulation (IAS-6), Jülich, Germany

##### **Correspondence:** Vahid Rostami (vhdrostami@gmail.com)

*BMC Neuroscience* 2019, **20(Suppl 1)**:P226

Recent studies [1-3] have extended the balanced random network model to incorporate clusters of strongly interconnected neurons. This network topology can exhibit a functionally desired attractor dynamics [3-5]. In the regime repeated attractor stimulation results in a temporal reduction of an initially increased trial-to-trial spike count variability [1-3] that qualitatively matches the experimentally observed variability dynamics [6-8] (Fig. 1A). However, we recently showed that in the regime the firing rate of inactivated clusters tends quickly towards firing rate saturation [9], which is inconsistent with experimental observations and strongly limits the dynamic working range. Moreover, the regime is highly sensitive even to small changes in network parameters which strongly limits robustness. Moreover, when the stimulus is weak the spiking network model fails to capture the reduction of trial-to-trial variability during stimulation (Fig. 1B).

**Fig. 1:**
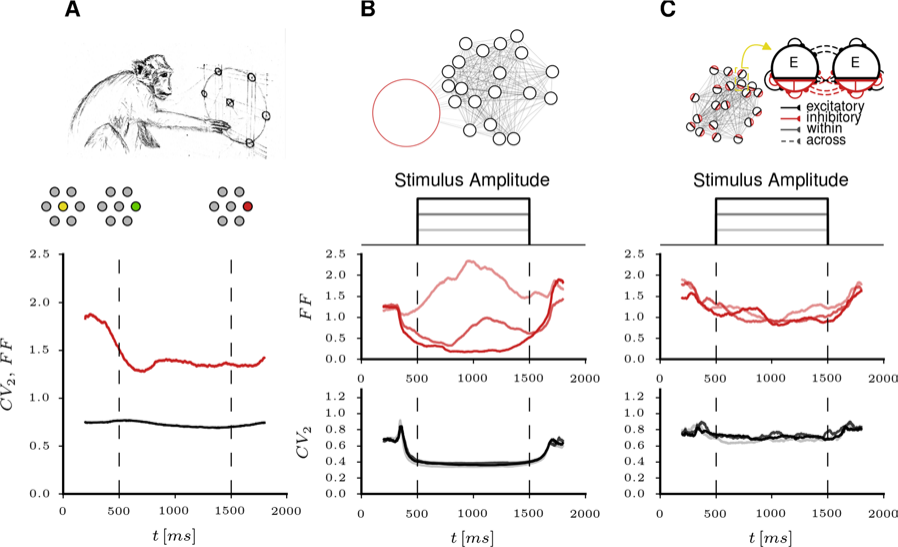
Variability dynamics in **a** experimental data recorded from the motor cortex of a macaque monkey during a delayed center-out reach task [6], **b** network model with purely excitatory clusters and global inhibition, and **c** network model with excitatory and inhibitory clusters

To improve on these aspects we incorporated two biologically plausible mechanisms in our cortical network models. We first implemented clustering of inhibitory neuron pools (Fig. 1C) as motivated by an increasing number of anatomical and physiological studies that suggest stimulus and choice selectivity of inhibitory neurons [10-11].

We find that the network model with inhibitory clustering achieves biologically realistic spiking activity with respect to the firing rate regime, spiking regularity and trial-to-trial spike count variability. As shown in Fig. 1C the temporal dynamics of the Fano factor (FF) shows a realistic reduction even for weak stimulation (light colors) while spike train regularity remains constant, in line with the experimental observation. We further added the cellular mechanisms of spike frequency adaptation (SFA) to all neurons in the network consistent with its importance in accounting for cortical network dynamics found in a recent study of large-scale recordings [12]. We previously demonstrated a strong regularizing impact of SFA on cortical variability dynamics [13, 14]. Including the cellular mechanism of SFA adds a second transient temporal component to variability dynamics and enhances the robustness of attractor dynamics against variation in network parameters. We propose that both mechanisms—inhibitory clustering at the network level and spike frequency adaptation at the cellular level—are crucial features of functional processing units inneocortex.

**Acknowledgments:** This work is supported by the German Science Foundation under the Institutional Strategy of the University of Cologne within the German Excellence Initiative (DFG-ZUK 81/1).

**References**

1. Litwin-Kumar A, Doiron B. Slow dynamics and high variability in balanced cortical networks with clustered connections. *Nature neuroscience* 2012 Nov;15(11):1498.
2. Mazzucato L, Fontanini A, La Camera G. Dynamics of multistable states during ongoing and evoked cortical activity. *Journal of Neuroscience* 2015 May 27;35(21):8214–31.
3. Deco G, Hugues E. Neural network mechanisms underlying stimulus driven variability reduction. *PLoS computational biology* 2012 Mar 29;8(3):e1002395.
4. Wang XJ. Probabilistic decision making by slow reverberation in cortical circuits. *Neuron* 2002 Dec 5;36(5):955–68.
5. Roudi Y, Latham PE. A balanced memory network. *PLoS computational biology* 2007 Sep 7;3(9):e141.
6. Rickert J, Riehle A, Aertsen A, Rotter S, Nawrot MP. Dynamic encoding of movement direction in motor cortical neurons. *Journal of Neuroscience* 2009 Nov 4;29(44):13870–82.
7. Churchland MM, Byron MY, Cunningham JP, et al. Stimulus onset quenches neural variability: a widespread cortical phenomenon. *Nature neuroscience* 2010 Mar;13(3):369.
8. Riehle A, Brochier T, Nawrot M, Gruen S. Behavioral context determines network state and variability dynamics in monkey motor cortex. *Frontiers in neural circuits* 2018;12.
9. Rost T, Deger M, Nawrot MP. Winnerless competition in clustered balanced networks: inhibitory assemblies do the trick. *Biological cybernetics* 2018 Apr 1;112(1–2):81–98.
10. Khan AG, Poort J, Chadwick A, et al. Distinct learning-induced changes in stimulus selectivity and interactions of GABAergic interneuron classes in visual cortex. *Nature neuroscience* 2018 Jun;21(6):851.
11. Najafi F, Elsayed GF, Pnevmatikakis E, Cunningham J, Churchland AK. Inhibitory and excitatory populations in parietal cortex are equally selective for decision outcome in both novices and experts. *bioRxiv* 2018 Jan 1:354340.
12. Stringer C, Pachitariu M, Steinmetz NA, et al. Inhibitory control of correlated intrinsic variability in cortical networks. *Elife* 2016 Dec 2;5:e19695.
13. Farkhooi F, Froese A, Muller E, Menzel R, Nawrot MP. Cellular adaptation facilitates sparse and reliable coding in sensory pathways. *PLoS computational biology* 2013 Oct 3;9(10):e1003251.
14. Farkhooi F, Muller E, Nawrot MP. Adaptation reduces variability of the neuronal population code. *Physical Review E* 2011 May 19;83(5):050905.

## P227 Representation of isometric wrist movement in the motor and somatosensory cortices of primates

### Nasima Sophia Razizadeh^1^, Marita Metzler^1^, Yifat Prut^2^, Martin Paul Nawrot^1^

#### ^1^University of Cologne, Computational Systems Neuroscience, Institute of Zoology, Cologne, Germany; ^2^The Hebrew University, Department of Medical Neurobiology, IMRIC, Hadassah Medical School, Jerusalem, Israel

##### **Correspondence:** Nasima Sophia Razizadeh (nasima.razizadeh@t-online.de)

*BMC Neuroscience* 2019, **20(Suppl 1)**:P227

In primate motor cortices (MC) it is well established that neurons encode kinematic parameters of voluntary arm movements such as movement, speed and direction [1, 2, 3]. Recurrent pathways between MC and somatosensory cortices (SS) provide a substrate for somatosensory integration during movement planning and execution. However, little is known about the encoding of movement parameters in SS. This project compares neuronal representation of movement direction in MC and SS during a multi-directional isometric wrist task [4]. The delayed task paradigm allows for a separation of preparation and movement epochs. Simultaneous recording of multiple single units from MC and SS enable us to study a potential temporal delay between MC and SS. For MC we expect to replicate published results in similar experiments [5, 4]. For MC and SS we quantitatively compare the task-related dynamics of (i) directional tuning [3] quantified by the signal-to-noise ratio [6], and (ii) trial-by-trial spiking variability [7, 8, 9]. So far obtained results indicate tuning in both cortices and a delay of directionally tuned activity in SS relative to MC (Fig. 1). An increase in directional tuning can already be observed in the preparatory phase in MC but not in SS. Average tuning strength in MC is greater than in SS. We anticipate that our findings will improve the understanding of cortico-cortical somatosensory interactions.

**Fig. 1:**
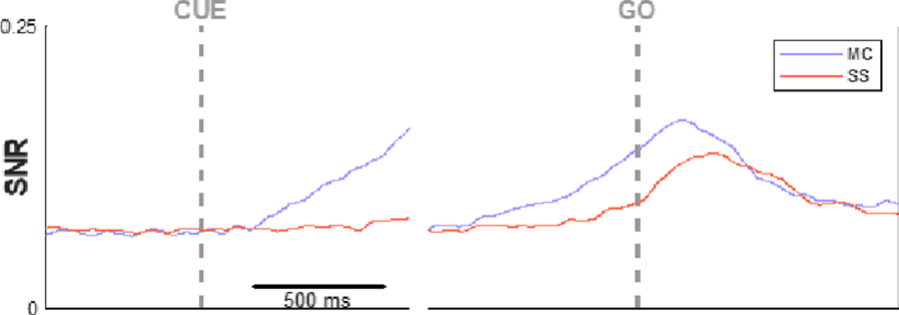
Time-resolved average signal to noise ratio (SNR) for motor cortex (MC) and somatosensory cortex (SS) during preparation and movement. The go signal was presented with a delay of 970 ms after the directional cue. The SNR presents the extent of directional tuning strength of a neuron. Red and blue curves show the average time-resolved SNR of 276 M1 and 484 SS neurons respectively

**Acknowledgements**: Funding was received from the German Israeli Foundation (GIF, Grant I-1224-396.13/2012 to MN and YP), the Israel Science Foundation (ISF-1787/13 to YP), and the German Science Foundation (DFG-ZUK 81/1 to MN).

**References**

1. Georgopoulos SP, Kalaska JF, Caminiti R, Massey JT. On the relations between the direction of two-dimensional arm movements and cell discharge in primate motor cortex. *Journal of Neuroscience* 1982, 2:1527–37.
2. Johnson MT, Mason CR, Ebner TJ. Central processes for the multiparametric control of arm movements in primates. *Current Opinions in Neurobiology* 2001, 11:684–688.
3. Rickert J, Riehle A., Aertsen A., Rotter S, Nawrot MP. Dynamic encoding of movement direction in motor cortical neurons. *Journal of Neuroscience* 2009, 29:13870-13882.
4. Yanai Y, Adamit N, Harel R, Israel Z, Prut Y. Connected corticospinal sites show enhanced tuning similarity at the onset of voluntary action. *Journal of Neuroscience* 2007, 27:12349–57.
5. Mahan MY, Georgopoulos AP. Motor directional tuning across brain areas: directional resonance and the role of inhibition for directional accuracy. *Frontiers in Neural Circuits* 2013, 7:92.
6. Mehring C, Nawrot MP, de Oliveira SC, et al. Comparing information about arm movement direction in single channels of local and epicortical field potentials from monkey and human motor cortex. *Journal of Physiology Paris* 2004, 98:498–506.
7. Nawrot MP, Boucsein C, Molina VR, Riehle A, Aertsen A, Rotter S. Measurement of variability dynamics in cortical spike trains. *Journal of Neuroscience Methods* 2008, 169:374–390.
8. Churchland MM, Yu BM, Cunningham JP, et al. Stimulus onset quenches neural variability: a widespread cortical phenomenon. *Nature Neuroscience* 2010, 13:369–78.
9. Riehle A, Brochier T, Nawrot MP, Grün S. Behavioral context determines network state and variability dynamics in monkey motor cortex. *Frontiers in Neural Circuits* 2018, 12:52.

## P228 Temporal credit-assignment in a detailed spiking model of the fly mushroom body can solve olfactory learning in dynamic odor environments.

### Hannes Rapp, Martin Paul Nawrot

#### University of Cologne, Computational Systems Neuroscience, Institute of Zoology, Cologne, Germany

##### **Correspondence:** Hannes Rapp (hannes.rapp@smail.uni-koeln.de)

*BMC Neuroscience* 2019, **20(Suppl 1)**:P228

Olfaction is a vital sense for insects and underlies innate as well as adaptive behaviors. During foraging flights, insects locate odor sources by navigating through airborne odor plumes. It has been shown experimentally that odor plumes evolve in fine filaments that form a complex, spatial landscape of volatile compounds. When flying through such plumes with intermittent structure, the animal’s sensory system thus encounters a temporally highly dynamic input with almost discrete stimulation peaks at a high temporal rate. Here we analyze a fully spiking multi-layered neural network model of the insect olfactory system of the fruit fly Drosophila melanogaster with realistic connectivity. Peripheral input from olfactory sensory neurons is processed in the antennal lobe generating a dense odor code in the population of projection neurons, which in turn transforms into a high-dimensional sparse representation [7] in the mushroom body (see Fig. 1). Population sparseness [5] and temporal sparseness [6] in the large population of Kenyon cells are achieved by lateral inhibition and spike frequency adaptation, respectively [1]. Our model is able to sense odor fluctuations on very short time scales in agreement with experimental studies [2, 9, 8]. We finally demonstrate that performing temporal credit-assignment within a single plastic [4] mushroom body output neuron (MBON) can rapidly solve the hard problem of odor source identification when navigating environments composed of turbulent mixtures [3] of odors that are emitted from separate distant sources. We suggest that our model for overcoming the delayed reward problem in the olfactory system is generic and applicable in different systems across taxa.

**Fig. 1:**
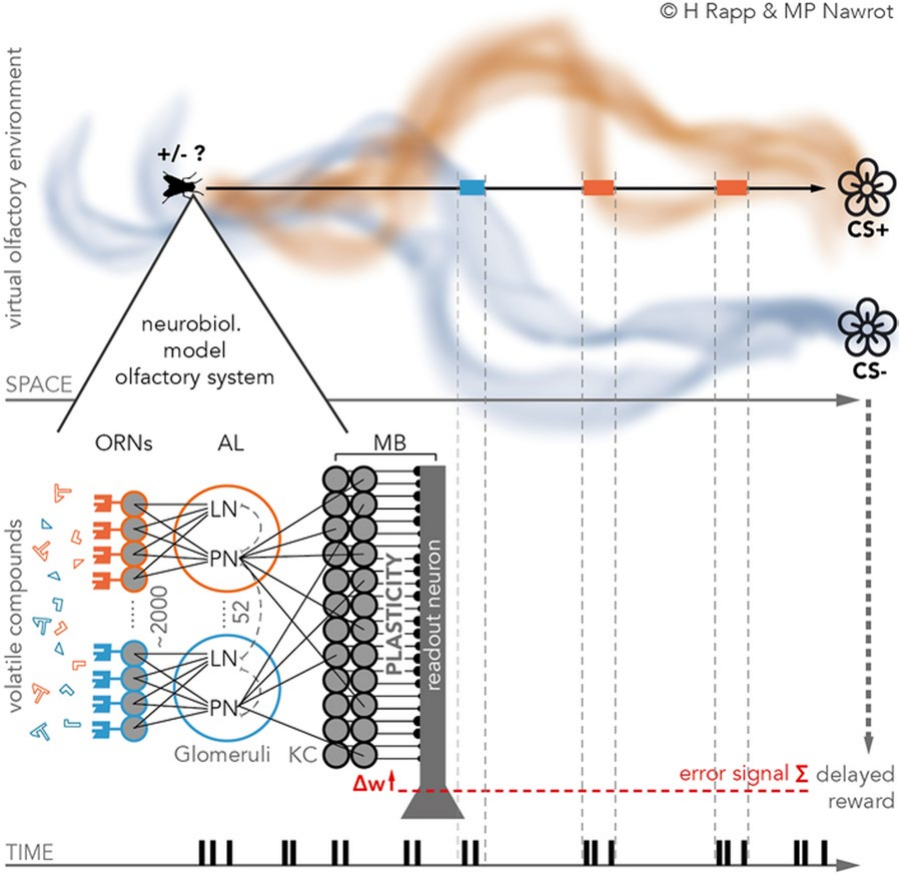
Top: Our simulated environment using fluid dynamics of turbulent odors from two sources. Subject travels upwind towards the sources. Reward delivery is delayed until after completion of a flight path at one of the two sources. Bottom: Sketch of spiking network model of the olfactory pathway, mushroom body (MB) of Drosophila and a plastic readout neuron to solve the temporal credit-assignment

**Acknowledgements**: Supported by DFG (grant no. 403329959) within the Research Unit FOR 2705.

**References**

1. Betkiewicz RL, Nawrot MP, Lindner B. Circuit and cellular mechanisms facilitate the transformation from dense to sparse coding in the insect olfactory system. *bioRxiv* 2017 Jan 1:240671.
2. Egea-Weiss A, Renner A, Kleineidam CJ, Szyszka P. High precision of spike timing across olfactory receptor neurons allows rapid odor coding in Drosophila. *IScience* 2018 Jun 29;4:76–83.
3. Farrell JA, Murlis J, Long X, Li W, Cardé RT. Filament-based atmospheric dispersion model to achieve short time-scale structure of odor plumes. *Environmental fluid mechanics* 2002 Jun 1;2(1–2):143–69.
4. Gütig R. Spiking neurons can discover predictive features by aggregate-label learning. *Science* 2016 Mar 4;351(6277):aab4113.
5. Honegger KS, Campbell RA, Turner GC. Cellular-resolution population imaging reveals robust sparse coding in the Drosophila mushroom body. *Journal of Neuroscience* 2011 Aug 17;31(33):11772–85.
6. Ito I, Ong RC, Raman B, Stopfer M. Sparse odor representation and olfactory learning. *Nature neuroscience* 2008 Oct;11(10):1177.
7. Kloppenburg P, Nawrot MP. Neural coding: sparse but on time. *Current Biology* 2014 Oct 6;24(19):R957–9.
8. Krofczik S, Menzel R, Nawrot MP. Rapid odor processing in the honeybee antennal lobe network. *Frontiers in computational neuroscience* 2009 Jan 15;2:9.
9. Szyszka P, Gerkin RC, Galizia CG, Smith BH. High-speed odor transduction and pulse tracking by insect olfactory receptor neurons. *Proceedings of the National Academy of Sciences* 2014 Nov 25;111(47):16925–30.

## P229 Model of the fruit fly’s mushroom body reproduces olfactory extinction learning

### Magdalena Anna Springer^1^, Johannes Felsenberg^2^, Martin Paul Nawrot^1^

#### ^1^University of Cologne, Computational Systems Neuroscience, Institute of Zoology, Cologne, Germany; ^2^Friedrich Miescher Institute for Biomedical Research, Basel, Switzerland

##### **Correspondence:** Magdalena Anna Springer (magdalena.springer@yahoo.de)

*BMC Neuroscience* 2019, **20(Suppl 1)**:P229

Adapting to an ever-changing world is crucial for survival and reproduction of all animals. When learned information turn out to be incorrect, the underlying memory needs to be updated. Memory extinction, the ability to reprocess previously learned information by integrating contradictory information, is a key player in this adaptation process. Insight from experimental research on *D. melanogaster* suggest that after memory extinction two parallel but opposing memory traces coexist at different sites in the mushroom body (MB) [1] (Fig. 1A). To validate this hypothesis, we designed and investigated a simple connectionist model (based on [2]) integrating the fly’s olfactory and reinforcement pathway. The multi-layered model employs plastic synaptic connections in separate appetitive and aversive learning pathways [3]. In this context we focus on two specific MBONs, (i) the MVP2 neuron mediating approach; its inhibition is associated with avoidance behavior [4]; (ii) the M6 neuron mediating avoidance behavior; blocking M6 induces appetitive behavior [5].

**Fig. 1:**
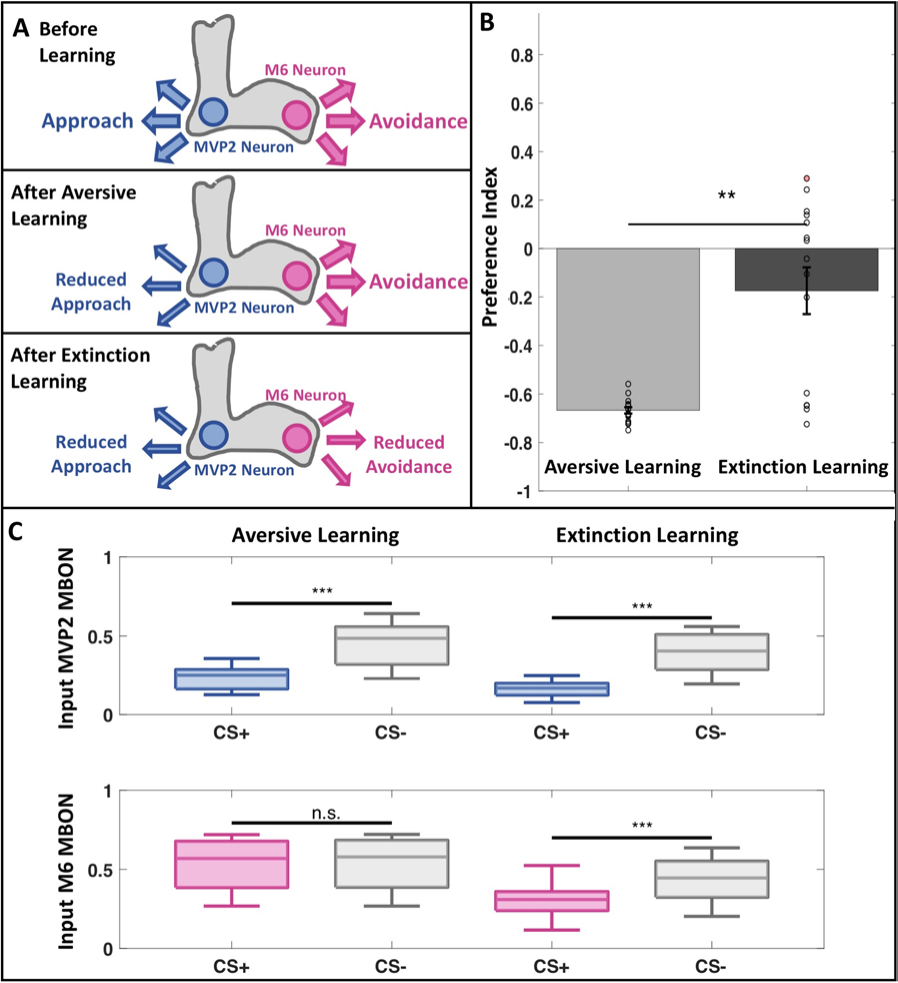
**a** The mechanisms underlying memory extinction in the fruit fly’s MB. (modified from [6]). **b** The model reproduces olfactory extinction learning (p<.01, n = 15). **c** Aversive conditioning leads to a reduced CS+ response in MVP2 MBON (p<.001), but not in M6 MBON (p>.05). After extinction, the MVP2 trace remains (p<.001) and there is an additional decrease in the CS+ response in M6 MBON (p<.05, n = 15)

We subjected our model to an aversive learning protocol. In the training phase, one odor (CS+) was paired with punishment. During the test phase the CS+ odor was presented alone to measure the conditioned response (CR). In the experiments investigating extinction learning, a second training phase in which the CS+ odor was presented without punishment was included.

After initial absolute conditioning the model showed an aversive CR for the CS+ odor test (Fig. 1B). However, the reactivation during the extinction phase resulted in a robust suppression of aversive memory (Fig. 1B). Thus, our model is suitable to qualitatively and quantitatively reproduce olfactory extinction experiments in *D. melanogaster*.

To test whether two separate memory traces are formed in the model, we compared the total synaptic drive received by the MBONs in response to stimulation with CS+ and a control odor (CS−) during different experimental phases (Fig. 1C). In the MVP2 MBON, there was a significant decrease in CS+ response relative to CS− evoked activation after the initial training. This effect was still visible after the extinction learning. In contrast, aversive training did not alter the M6 neuron response. However, extinction training led to a reduction of the CS+ specific M6 drive. The presented findings match the recent behavioral and *in vivo* neurophysiological results in *D. melanogaster* [1]. Our model supports the hypothesis of a parallel appetitive memory trace that is formed during aversive extinction learning.

**Acknowledgements**: Supported by the German Research Foundation (grant no. 403329959) within the Research Unit DFG-FOR 2705, https://www.uni-goettingen.de/de/601524.html

**References**

1. Felsenberg J, Jacob PF, Walker T, et al. Integration of parallel opposing memories underlies memory extinction. *Cell* 2018 Oct 18;175(3):709–22.
2. Peng F, Chittka L. A simple computational model of the bee mushroom body can explain seemingly complex forms of olfactory learning and memory. *Current Biology* 2017 Jan 23;27(2):224–30.
3. Bouzaiane E, Trannoy S, Scheunemann L, Plaçais PY, Preat T. Two independent mushroom body output circuits retrieve the six discrete components of Drosophila aversive memory. *Cell reports* 2015 May 26;11(8):1280–92.
4. Perisse E, Owald D, Barnstedt O, Talbot CB, Huetteroth W, Waddell S. Aversive learning and appetitive motivation toggle feed-forward inhibition in the Drosophila mushroom body. *Neuron* 2016 Jun 1;90(5):1086–99.
5. Owald D, Felsenberg J, Talbot CB, et al. Activity of defined mushroom body output neurons underlies learned olfactory behavior in Drosophila. *Neuron* 2015 Apr 22;86(2):417–27.
6. Felsenberg J, Owald D. Making Memories. On the fly. *e-Neuroforum* 2018 May 25;24(2):A53–60.

## P230 Modelling a biologically realistic microcircuit of the Drosophila mushroom body calyx

### Miriam Faxel^1^, Gaia Tavosanis^2^, Philipp Ranft^3^, Martin Paul Nawrot^4^

#### ^1^University of Cologne, Köln, Germany; ^2^German Center for Neurodegenerative Diseases, Dendrite Differentiation Unit, Bonn, Germany; ^3^German Center for Neurodegenerative Diseases, Dynamics of Neuronal Circuits, Bonn, Germany; ^4^University of Cologne, Computational Systems Neuroscience, Institute of Zoology, Cologne, Germany

##### **Correspondence:** Miriam Faxel (mfaxel@smail.uni-koeln.de)

*BMC Neuroscience* 2019, **20(Suppl 1)**:P230

Odour detection and odour learning are crucial for insect survival. The anatomy of the olfactory system of the fruit fly Drosophila melanogaster has been described in great detail and is thus suitable for biologically detailed modelling of odor processing and synaptic plasticity. The insect mushroom body (MB) is a central brain neuropil, that integrates different sensory modalities and is an essential site for learning-induced plasticity. The olfactory input region (calyx) of the MB is organized into a large number of microglomeruli (MGs). Each MG constitutes a recurrent microcircuit of high synaptic density (cf. Fig. 1) [1-5]. It involves recurrent connections between one central large bouton of a single projection neuron (PN, providing excitatory olfactory input from the antennal lobe), the primarily postsynaptic Kenyon cells (KCs, the excitatory primary intrinsic neurons of the MB) and the inhibitory anterior-paired lateral neuron (APL).

**Fig. 1:**
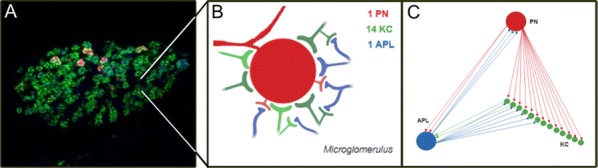
A: Optical section through calyx of adult fly, synaptic markers define the microglomerular complexes. B: Schematic drawing of MG microcircuit (postsynaptic endings represented by curved structures). The model is realised in Brian2, uses realistic neuronal parameters, consists of 1 PN,14 KCs (both modelled as LIF neurons) and one non-spiking APL neuron. The exact connections are visualized in C

Here we investigate a model for local sensory processing within a single MG based on detailed structural data from a fly connectome [6, 7]. We hypothesize that the inhibitory APL is a non-spiking neuron, that can be locally activated within the MG and recursively, provides local feedback inhibition within the same MG as well as lateral inhibition to neighbouring MGs. This circuit motive leads to a reduction of the overall KC activity and we quantify the effect on population sparseness in the KC population. Experimental studies have indicated that the MGs are also a locus of learning-induced long-term and short-term memory [7, 5]. Inspired by our previous model in the honeybee MB [8] we explore how plasticity at the inhibitory synapses could play a role in reinforcement learning.

**Acknowledgments:** This research is supported by the German Research Foundation (grant no. 403329959) within the Research Unit ‘Structure, Plasticity and Behavioral Function of the Drosophila mushroom body’ (DFG-FOR 2705) to GT and MPN.

**References**

1. Butcher NJ, et al. Different classes of input and output neurons reveal new features in microglomeruli of the adult Drosophila mushroom body calyx. *Journal of Comparative* *Neurology* 2012, 520 (10), 2185–2201
2. Leiss F, et al. Synaptic organization in the adult Drosophila mushroom body calyx. *Journal of Comparative* *Neurology* 2009, 517(6),808–824.
3. Kremer MC, et al. Structural long-term changes at mushroom body input synapses. *Current Biology* 2010,20(21),1938–1944
4. Groh C, Rössler W. Comparison of microglomerular structures in the mushroom body calyx of neopteran insects. *Arthropod structure & development* 2011 Jul 1;40(4):358–67.
5. Haenicke J, et al. Neural Correlates of Odor Learning in the Presynaptic Microglomerular Circuitry in the Honeybee Mushroom Body Calyx. *eNeuro* 2018,5(3), ENEURO.0128-18.2018.
6. Zheng et al. A Complete Electron Microscopy Volume of the Brain of Adult Drosophila melanogaster. *Cell* 2018, 174(3), 730–743.
7. Blatruschat et al. in revision
8. Haenicke J. Modeling insect inspired mechanisms of neural and behavioral plasticity. *Freie Universität Berlin* 2015, urn:nbn:de:kobv:188-fudissthesis000000100699-0.

## P231 Relevance of non-synaptic interactions in the neural encoding of odorants: a good start is half the battle

### Mario Pannunzi^1^, Paul Szyszka^2^, Thomas Nowotny^3^

#### ^1^University of Sussex, Department of Informatics, Brighton, United Kingdom; ^2^University of Otago, Dunedin, New Zealand; ^3^University of Sussex, School of Engineering and Informatics, Brighton, United Kingdom

##### **Correspondence:** Mario Pannunzi (mario.pannunzi@gmail.com)

*BMC Neuroscience* 2019, **20(Suppl 1)**:P231

In many insect species, including Drosophila melanogaster, olfactory receptor neurons (ORNs) are housed in hair-like sensilla in a stereotypical manner. Each sensillum contains two or more ORNs of different types. ORNs within the same sensillum interact, without synaptic connection, mostly inhibiting each other. As suggested previously [1, 2], these non-synaptic interactions (NSIs) could be crucial for insects capability to resolve concentration ratio or timing between different odorants at high resolution, and they are the focus of our study.

Here we test the hypothesis that NSIs could improve the spatiotemporal resolution of odor recognition in mixed odor plumes (see Fig. 1): If a single source emits an odor mixture (multiple odorants), odorants arrive in close synchronization, NSIs take effect and both ORNs responses are diminished. If separate sources emit odorants, their concentrations are less correlated, and NSIs have almost no effect, resulting in larger ORN responses. This fast mechanism could provide an essential advantage because behavioural relevant temporal and spatial scales of plumes can be very fine, on the order of tens of milliseconds and tens of millimeters.

**Fig. 1:**
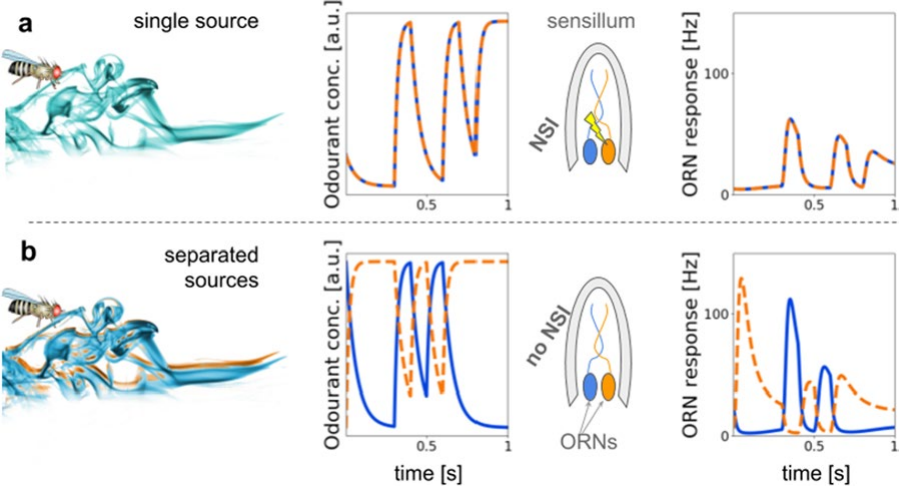
Driving hypothesis: **a** If a single source emits an odor mixture (multiple odorants), odorants arrive in close synchronization, NSIs take effect and both ORNs responses diminished. **b** If separate sources emit odorants, their concentrations are less correlated, and NSIs have almost no effect, resulting in larger ORN responses. ORN response data shown is based on our model

Here, we analyze this hypothesis in a computational model. Firstly, we generate a model of the early olfactory system of insects—a simple circuit model with two ORNs and their corresponding projection neurons (PNs) and local neurons (LNs) in the antennal lobe (AL)—and reproduce the responses to simple odor stimuli reported in the literature. Secondly, we analyze the advantages of having NSIs (compared to not having them) for detecting target odors within complex mixtures.

The system reproduces most of the response dynamics reported in the literature: ORN responses adapt and follow ‘intensity invariant’ trends as recently reported [3]; a linear-nonlinear model well describe ORN responses for long periods of stimulation [3]; and when the stimulus is a step, the function relating the activity of ORNs to PN responses is the same sigmoidal function as reported by Olsen and Wilson [4]. This latter effect is a consequence of the assumptions of the model and no parameter was tuned to obtain it.

We then studied how the presence or absence of NSIs between ORNs modifies the ability of the model to detect a target odorant in an odorant mixture. Our results lead us to speculate that NSIs and lateral inhibition may implement two different functions: NSIs have a high spatiotemporal resolution and they generate selective inhibition between ORNs; Local LN networks take effect later to decorrelate PN activities and normalize them with respect to the average input from ORNs [5].

**Acknowledgments:** This research was funded by the European Union under grant agreement 785907(HBP SGA2), and the Human Frontiers Science Program, grant RGP0053/2015 (Odor Objects).

**References**

1. Todd JL, Baker TC. Function of peripheral olfactory organs. *Insect olfaction* 1999: p. 67-–6;
2. Su CY et al. Non-synaptic inhibition between grouped neurons in an olfactory circuit. *Nature* 2012: 492(7427) p. 66.
3. Martelli C, et al. Intensity invariant dynamics and odor-specific latencies in olfactory receptor neuron response. *Journal of Neuroscience* 2013, 33(15): p. 6285–6297.
4. Olsen SR, et al. Divisive normalization in olfactory population codes. *Neuron* 2010: 66.2 p. 287–299.
5. Wilson RI. Early olfactory processing in Drosophila: mechanisms and principles. *Annual review of neuroscience* 2013, 36: p. 217–241.

## P232 Learning a reward distribution with reward prediction errors in a model of the Drosophila mushroom body

### James Bennett, Thomas Nowotny

#### University of Sussex, School of Engineering and Informatics, Brighton, United Kingdom

##### **Correspondence:** James Bennett (james.bennett@sussex.ac.uk)

*BMC Neuroscience* 2019, **20(Suppl 1)**:P232

Drosophilae exhibit matching behavior, whereby their relative preferences for different foods are determined by the relative nutritional and energetic values of the respective food sources [1]. Key to this behavior is their ability to learn and accurately evaluate the valence of different options that elicit unique sensory cues. An important site of learning in Drosophila is the mushroom body (MB) [2]. Current models posit that distinct regions of the MB encode the valence of reward information and actions (Fig. 1A): DANs in the PAM cluster (hereafter called D+) are excited by positive (+ve) rewards (R+),depressing active Kenyon cell (KC) synapses onto MBONs that bias actions toward retreat (M-); DANs in the PPL1 cluster (D-) are excited by punishments, or negative (−ve) rewards (R), and depress active (+). Some MBONs provide excitatory feedback to DANs [3, 4], such that the learned reduction in MBON firing can offset the excitatory reward signal arriving at that DAN. Thus, D (D +ve (−ve) reward valence. Here, we postulate that the difference between M+ and M-firing rates signals the net learned valence, i.e. a reward prediction, associated with a particular sensory cue. We capture these details in a reduced, computational MB model (Fig. 1B), in which the tens of neurons comprising each cell type mentioned above are modeled with just a single rate-based, point neuron.

**Fig. 1:**
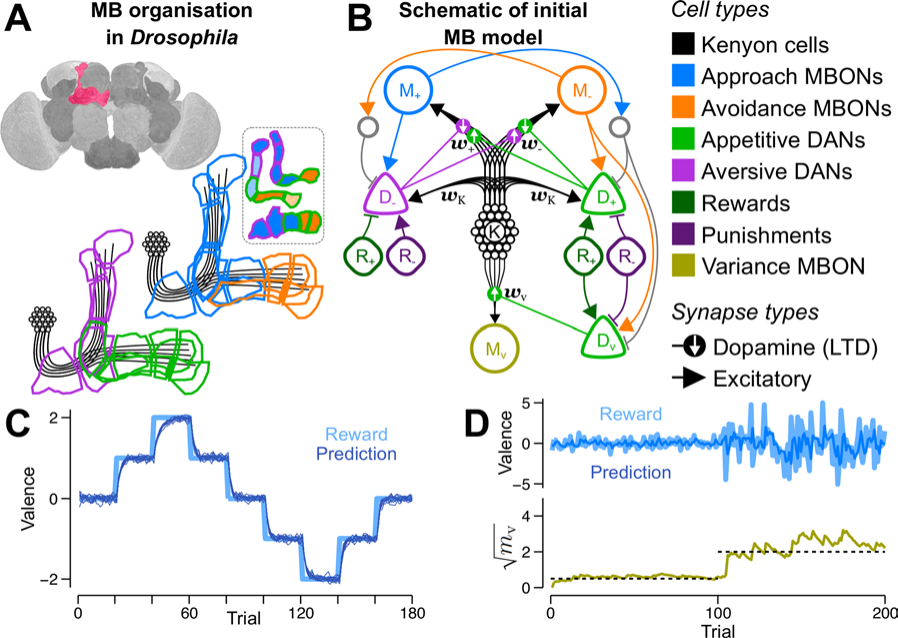
**a** Anatomical and functional organization of the MB. Colors indicate cell types as in **b**. **b** Schematic of the MB model presented in this work. **c** The MB model is able to accurately learn and update predictions for the true reward mean with a dynamic reward schedule. **d** The variance encoding MBON in the extended MB model learns and updates accurate predictions of the true reward variance

We investigate the ability of the MB to track multiple dynamic cue-specific rewards. Learning accurate estimates of rewards amounts to minimizing the error between the reward prediction and the net reward (the difference between R+ and R−). We capture this objective with a cost function that penalizes RPEs over all cued options. A Rescorla-Wagner like plasticity rule can then be obtained by gradient descent on the cost functions with respect to the KC-MBON synaptic weights. However, we show that the known MB circuitry cannot learn under this normative approach, as all synaptic weights are driven to zero. We therefore propose additional circuitry in the MB (Fig. 1B) that not only enables learning (Fig. 1C), but also captures several additional physiological and behavioral phenomena observed in experiments. We then demonstrate how the model performs as a function of the number of options in a multi-armed bandit task.

We next extend the model to address how the MB may learn second order moments of a reward distribution, which provide a measure of reward reliability, given that unreliable rewards carry risks that an organism may want to avoid. We therefore formulate a new cost function and derive a corresponding plasticity rule that minimizes a reward-variance prediction error (Fig. 1D). We then demonstrate how scaling reward predictions by variance predictions can explain the relative utility of different flower species, as determined by the foraging behavior of bees and wasps, and the dependence of utility on the mean and variance in the flowers’ respective nectar yields [5].

**References**

1. Beshel J, Zhong Y. Graded encoding of food odor value in the Drosophila brain. *Journal of Neuroscience* 2013 Oct 2;33(40):15693–704.
2. Cognigni P, Felsenberg J, Waddell S. Do the right thing: neural network mechanisms of memory formation, expression and update in Drosophila. *Current opinion in neurobiology* 2018 Apr 1;49:51–8.
3. Felsenberg J, Barnstedt O, Cognigni P, Lin S, Waddell S. Re-evaluation of learned information in Drosophila. *Nature* 2017 Apr;544(7649):240.
4. Felsenberg J, Jacob PF, Walker T, et al. Integration of parallel opposing memories underlies memory extinction. *Cell* 2018 Oct 18;175(3):709–22.
5. Real LA. Animal choice behavior and the evolution of cognitive architecture. *Science* 1991 Aug 30;253(5023):980–6.

## P233 Does structure in neural correlations match anatomical structure?

### Thomas Delaney, Cian O’Donnell

#### University of Bristol, Computer Science, Bristol, United Kingdom

##### **Correspondence:** Thomas Delaney (td16954@bristol.ac.uk)

*BMC Neuroscience* 2019, **20(Suppl 1)**:P233

Information in the brain is carried in correlated network activity. Decades of research has established that these correlations play a crucial role in representing sensory information. For example, the onset of visual attention has been shown to have a greater effect on the correlations in the macaque V4 than on the firing rates in that region [1]. In order to understand the representation of sensory information we must understand the interactions between neurons.

Because of limitations in recording technology almost all research has explored correlations between neurons within a given brain region. Relatively little is known about correlations between neurons in different brain regions. However, the recent development of ‘Neuropixels’ probes [2] has allowed extracellular voltage measurements to be collected from multiple brain regions simultaneously routinely, and in much larger numbers than traditional methods. In this project we used a publicly-available Neuropixels dataset to analyze correlations between different brain regions.

Using two probes, spiking activity was simultaneously collected from over 800 neurons in an awake mouse brain for a period of 84 minutes. During this period, the mouse and was shown various visual stimuli. The 800 neurons were distributed across 5 different brain regions: V1, hippocampus, thalamus, motor cortex, and striatum. Using these data, we examined pairwise correlations between neurons within the same region, and between neurons in different regions (see Fig. 1). We also compared the distribution of regional pairwise correlations across regions. As well as measuring pairwise correlations, we took an information theoretic approach and measured the ‘incremental mutual information’ (IMI) between pairs of neurons [3]. Again we measured the IMI for pairs of neurons within regions and for pairs of neurons in different regions.

**Fig. 1:**
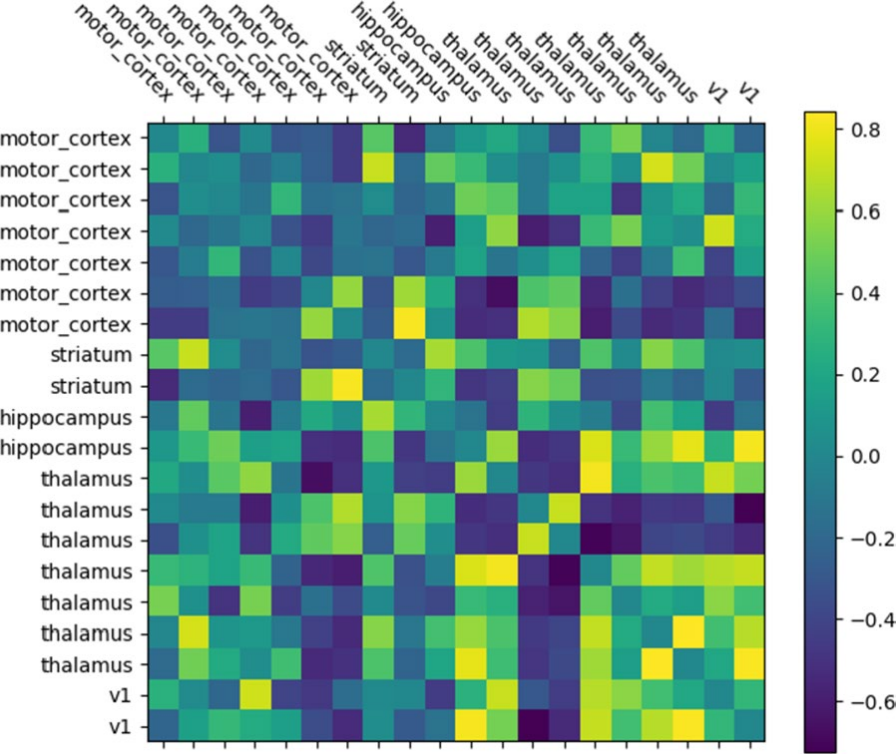
Spike count correlations between 20 randomly chosen neurons in 5 different brain regions. Spiking activity was measured while the mouse was exposed to a moving bar visual stimulus

Our main objective was to see if, using these quantities, the neurons could be clustered such that the clustering matched their anatomical partition. We first clustered the neurons based on their spike responses to stimuli. Then we clustered pairs of neurons based on their pairwise correlations. Then we clustered pairs of neurons based on their IMI. Finally we compared these clustering results to the anatomical partition of the cells.

**Acknowledgements**: I would like to thank Dr. Nick Steinmetz (University of Washington, Seattle) for making the dataset used in this project publicly available.

**References**

1. Cohen MR, Maunsell JH. Attention improves performance primarily by reducing interneuronal correlations. *Nature Neuroscience* 2009 Dec;12(12):1594.
2. Jun JJ, Steinmetz NA, Siegle JH, et al. Fully integrated silicon probes for high-density recording of neural activity. *Nature* 2017 Nov;551(7679):232.
3. Singh A, Lesica NA. Incremental mutual information: a new method for characterizing the strength and dynamics of connections in neuronal circuits. *PLoS Computational Biology* 2010 Dec 9;6(12):e1001035.

## P234 The structure of the population code in V1 and V4 microcircuits responding to natural stimuli.

### Veronika Koren^1^, Ariana Andrei^2^, Ming Hu^3^, Valentin Dragoi^2^, Klaus Obermayer^1^

#### ^1^Technische Universität Berlin, Institute of Software Engineering and Theoretical Computer Science, Berlin, Germany; ^2^University of Texas Medical School, Department of Neurobiology and Anatomy, Houston, Texas, United States of America; ^3^Massachusetts Institute of Technology, Picower Institute for Learning and Memory, Cambridge, Massachusetts, United States of America

##### **Correspondence:** Veronika Koren (koren@ni.tu-berlin.de)

*BMC Neuroscience* 2019, **20(Suppl 1)**:P234

In visual areas of primates, neurons activate in parallel while the animal is engaged in a behavioral task. In this study, we examine the structure of the population code while the animal performs delayed match to sample task on complex natural images. The macaque monkey visualizes two consecutive stimuli that can be either the same or different, while we record the activity of neural populations that span the cortical depth in its V1 and V4 cortical areas. We decode correct choices on binary stimulus classes (“same” and “different”) in single neurons as well as in the population of simultaneously recorded units. The use of a linear decoder allows to compute population decoding weights that also take into account inter-neuron interactions. Such weights have a straightforward biological interpretation as the synaptic weights between a population of projecting neurons and a read-out neuron. We show that population decoding weights are uncorrelated with absolute firing rate of single neurons, their trial-to-trial variability and the coupling of single neurons with the population. The population code is therefore not merely a byproduct of decoding properties of single neurons, but instead has its own intrinsic structure.

Comparing the predictive power of the activity of single neurons with the high-dimensional model of the population activity, we find that typically, the high-dimensional read-out of the population activity predicts correct choices better than an average single neuron from the same recording session. This increase in performance is attributed to more information in the population response, compared to single neurons, and is in general not due to the correlation structure of simultaneously active units. Considering the population decoding weights, we divide the population in two mutually exclusive coding pools. We find that correlations between neurons from the same coding pool decrease the performance of the decoder, while correlations between neurons from different coding pools do not affect the performance. Even though correlations within the same coding pool slightly decrease the accuracy of discrimination, neurons within the coding pool are more strongly correlated than neurons across the two coding pools. The difference in pairwise interactions for neurons within and across coding pools appears to be a robust structural feature of the population code. It is present in the correlation of trial-to-trial variability, correlation of spike counts on shorter time scales as well as in synchrony of spiking. This effect is robust across the two brain areas and across different time windows. It persists also when we replace optimal decoding weights with non-optimal ones and divide the population in two coding pools according to the preference of each single neuron for one of the two mutually exclusive stimulus classes. In summary, our results point out the existence of functional subnetworks that also differ in pairwise interactions on different time scales.

## P235 Diversity of networks activity is provided by chemical plus electrical synapses

### Kesheng Xu^1^, Jean Paul Maidana^2^, Patricio Orio^1^

#### ^1^Universidad de Valparaíso, Centro Interdisciplinario de Neurociencia de Valparaíso, Valparaiso, Chile; ^2^Universidad de Valparaíso, Estadística, Valparaíso, Chile

##### **Correspondence:** Kesheng Xu (kesheng.xu@cinv.cl)

*BMC Neuroscience* 2019, **20(Suppl 1)**:P235

Many experiments have evidenced that electrical and chemical synapses coexist in most organisms and brain structures (For reviews, see [1, 2]). The role of electrical and chemical synapse connection in diversity of neural activity generation has been investigated separately in networks of varying complexities. Nevertheless, theoretical understanding of mixed synapses in diverse dynamical states of neural networks for self-organization and robustness still has not been fully studied. We here present a model of neural network built with both types of synapse connections to investigate the emergence of global and collective dynamics states. These neural networks consist of excitatory and inhibitory populations interacting together. The excitatory population is connected by excitatory synapses in small world topology and adjacent neurons are also connected by gap junctions. The inhibitory population is only connected by chemical inhibitory synapses with all-to-all interaction. Our numerical simulations show that in the networks with weak electrical coupling, the synchrony states generated by this architecture are mainly controlled by heterogeneity among neurons and the balance of its excitatory and inhibitory inputs. More importantly, we show that the boundary between sub-threshold regime and firing regimes of excitatory populations is linear. In networks with strong electrical coupling, diverse dynamical states arise from different combinations of excitatory and inhibitory weights. We show that the synchronous firing, cluster synchrony, and various ripples events (such as traveling waves) emerge by slight modification of chemical coupling weights. For large enough electrical synapse coupling, the whole neural networks become synchronized. Our results pave a way in the study of the dynamical mechanisms and computational significance of the contribution of mixed synapses in the neural functions

**Acknowledgments:** We thank the funding from Fondecyt Project Nos. 1181076 (P.O.) and 3170342 (K.X.) from CONICYT, Chile. PO is partially funded by the Advanced Center for Electrical and Electronic Engineering (FB0008 Conicyt, Chile). The Centro Interdisciplinario de Neurociencia de Valparaíso (CINV) is a Millennium Institute supported by the Millennium Scientific Initiative of the Ministerio de Economía (Chile).

**References**

1. Nagy JI, Pereda AE, Rash JE. On the occurrence and enigmatic functions of mixed (chemical plus electrical) synapses in the mammalian CNS. *Neuroscience letters* 2017 Sep 11.
2. Nagy JI, Pereda AE, Rash JE. Electrical synapses in mammalian CNS: past eras, present focus and future directions. *Biochimica et Biophysica Acta (BBA)-Biomembranes* 2018 Jan 1;1860(1):102–23.

## P236 Is human connectome optimized to enhance dynamic cortical ignition?

### Samy Castro^1^, Wael El-Deredy^1^, Demian Battaglia^2^, Patricio Orio^3^

#### ^1^Universidad de Valparaiso, Centro de Investigacion y Desarrollo en Ingenieria en Salud, Valparaiso, Chile; ^2^Aix-Marseille Université, Institut de Neurosciences des Systèmes, Marseille, France; ^3^Universidad de Valparaíso, Instituto de Neurociencia, Valparaiso, Chile

##### **Correspondence:** Samy Castro (samy.castro@cinv.cl)

*BMC Neuroscience* 2019, **20(Suppl 1)**:P236

The map of the neural connections or connectome has been proposed as a key to understanding cortical activity. Indeed, network analysis of the whole-brain activity has shown that the fluctuations of cortical activity correlate with structural hubs and sub-network cores. Hubs underscore the influence of single areas in the network activity whereas the cores point out the contribution of shells of well-interconnected areas on the collective dynamics. Some authors postulate that structural cores rather than hubs enhance the propagation of activity in the networks [1]. However, the contribution of structural hubs and cores on the cortical activity needs to be disentangled by a computational perspective, with a focus on its collective dynamics. In this work, we simulated cortex-like dynamics using a mean-field model for cortical areas [2], wired by the Human cortex connectome (**HC**), obtained from diffusion imaging database [3]. Using the coupling strength between cortical areas (**G**) as a control parameter, we study the onset of the bistable regime, at which some cortical areas can develop a high or low firing rate activity, depending on the initial conditions. This bistable regime has been often associated with a functional role in working memory or input integration, and we call G- the minimum G value at which it is observed. At G-, high activity in the network is observed only in a unique subset of the cortical areas, that we call the ignition core. This subset corresponds with the critical s-core structure of the network but not with the hubs. Next, we studied the onset of the bistable regime in structural models, having either similar small-world index, degree or weight distribution of the HC. We find that the strength of global excitation needed to trigger ignition at G- is substantially larger for these models compared to the empirical HC. Notably, only the HC shows a tight core relationship with the ignited areas at G-, not observed in any of the structural models. Furthermore, when increasing the strength of excitation (or G), the propagation of ignition outside of this ignition core –which can self-sustain its high activity– is more gradual in the HC than for any of the randomized connectomes, allowing for better control of the number of ignited regions. We explain both these assets in terms of the exceptional weighted core-shell organization of the empirical connectome, speculating that this topology of HC may be optimized to support an enhanced ignition dynamic [4].

**Acknowledgments:** This work was supported by the Advanced Center for Electrical and Electronic Engineering (FB0008 CONICYT, Chile) and the supercomputing infrastructure of the NLHPC (ECM-02). The Centro Interdisciplinario de Neurociencia de Valparaiso (Chile, ICM09-022-P). SC was funded by Beca Doctorado Nacional CONICYT 21140603 and Programa de Doctorado en Neurociencia, Universidad de Valparaíso. PO was funded by FONDECYT 1181076.

**References**

1. Kitsak M, et al. Identification of influential spreaders in complex networks. *Nature Physics* 2010, 6 (11).
2. Hansen EC, et al. Functional connectivity dynamics: modeling the switching behavior of the resting state. *NeuroImage* 2015, 105, 525–535
3. Hagmann P, et al. Mapping the structural core of human cerebral cortex. *PLoS Biol* 2008, 6, e159.
4. Deco G, Kringelbach ML. Hierarchy of Information Processing in the Brain: A Novel ‘Intrinsic Ignition’ Framework. *Neuron* 2017, 94, 961–968.

## P237 Comparing the effects of adaptation and synaptic filtering on the timescale of recurrent networks

### Manuel Beiran, Srdjan Ostojic

#### Ecole Normale Supérieure, Department of Cognitive Studies, Paris, France

##### **Correspondence:** Manuel Beiran (manuel.beiran@ens.fr)

*BMC Neuroscience* 2019, **20(Suppl 1)**:P237

Neural activity in awake behaving animals exhibits a vast range of timescales that can be several fold larger than the membrane time constant of individual neurons. Two types of mechanisms have been proposed to explain the origin of these large timescales. One possibility is that large timescales are generated by a network mechanism based on positive feedback, but this hypothesis requires fine-tuning of the strength or structure of the synaptic connections. A second possibility is that large timescales in the neural dynamics are inherited from large timescales of underlying biophysical processes, two prominent candidates being intrinsic adaptive ionic currents and synaptic transmission. How the timescales of adaptation or synaptic transmission influence the timescale of the network dynamics has however not been fully explored.

To address this question, here we analyze large networks of randomly connected excitatory and inhibitory units with additional degrees of freedom that correspond to adaptation or synaptic filtering. We determine the fixed points of the systems, their stability to perturbations and the related dynamical timescales (Fig. 1 A-B). Furthermore, we apply dynamical mean field theory to study the temporal statistics of the activity beyond the bifurcations, and examine how the effects of adaptation and synaptic timescales transfer from individual units to the whole population (Fig. 1 C).

**Fig. 1:**
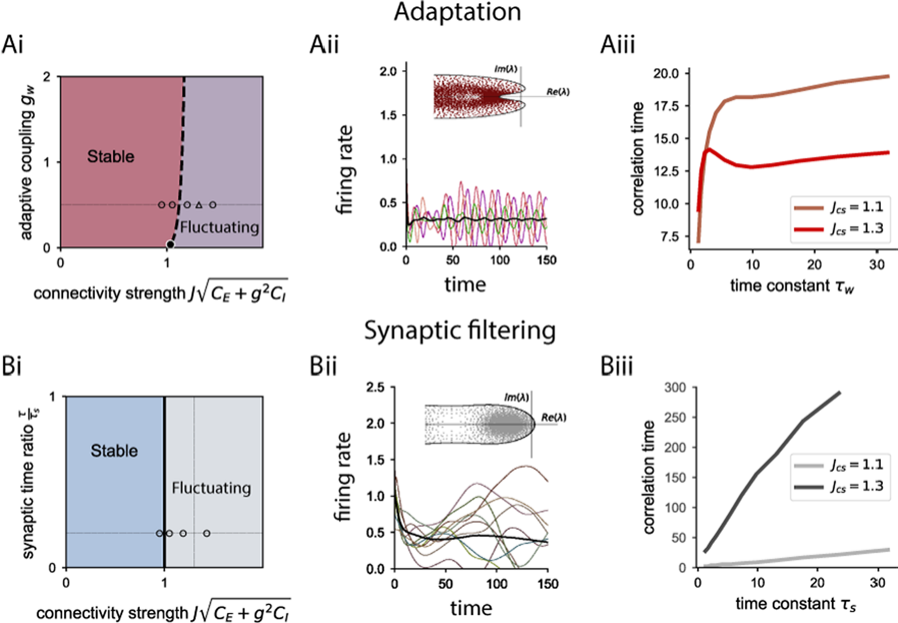
Diagram of the different dynamical regimes of the recurrent neural network (i), illustration of the dynamics of ten units in the fluctuating regime (ii) with the eigenspectrum of the linearized dynamics (inset) and timescale of the activity of single units in the network as a function of the timescale of the slow process (iii)

Our overarching finding is that synaptic filtering and adaptation in single neurons have very different effects at the network level. Unexpectedly, the macroscopic network dynamics do not inherit the large timescale present in adaptive currents. In contrast, the timescales of network activity increase proportionally to the time constant of the synaptic filter. Altogether, our study demonstrates that the timescales of different biophysical processes have different effects on the network level, so that the slow timescales of different biophysical processes within individual neurons do not necessarily induce slow activity in large recurrent neural networks.

## P238 Computations of inhibition of return mechanisms by modulating V1 dynamics

### David Berga^1^, Xavier Otazu^1,2^

#### ^1^Universitat Autonoma de Barcelona, Computer Vision Center, Barcelona, Spain; ^2^Dept. of Computer Science, Barcelona, Spain

##### **Correspondence:** David Berga (dberga@cvc.uab.es)

*BMC Neuroscience* 2019, **20(Suppl 1)**:P238

In this study we present a unified model of the visual cortex for predicting visual attention using real image scenes. Feedforward mechanisms from RGC and LGN have been functionally modeled using wavelet filters at distinct orientations and scales for each chromatic pathway (Magno-, Parvo-, Konio-cellular) and polarity (ON-/OFF-center), by processing image components in the CIE Lab space. In V1, we process cortical interactions with an excitatory-inhibitory network of firing rate neurons, initially proposed by [1], later extended by [2]. Firing rates from model’s output have been used as predictors of neuronal activity to be projected in a map in superior colliculus (with WTA-like computations), determining locations of visual fixations. These locations will be considered as already visited areas for future saccades, therefore we integrated a spatiotemporal function of inhibition of return mechanisms (where LIP/FEF is responsible) to feed to the model with spatial memory for next saccades. Foveation mechanisms have been simulated with a cortical magnification function, which distort spatial viewing properties for each fixation. Results show lower prediction errors than with respect no IoR cases (Fig. 1), and it is functionally consistent with human psychophysical measurements. Our model follows a biologically-constrained architecture, previously shown to reproduce visual saliency [3], visual discomfort [4], brightness [2] and chromatic induction [5].

**Fig. 1:**
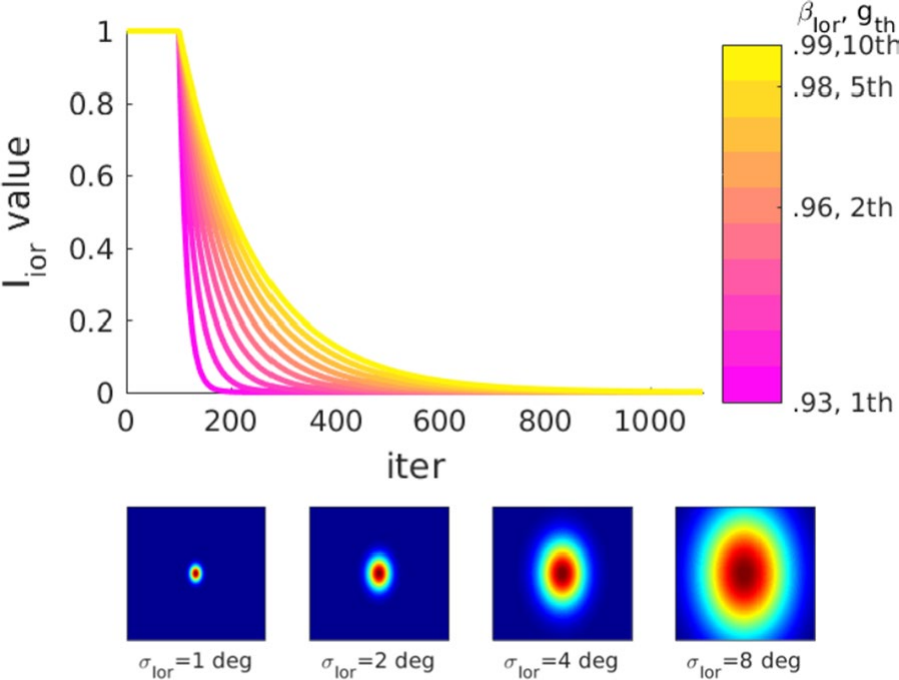
Evolution of inhibition factor for 100 mem.time (about 1000 iterations), corresponding approximately to performing 10 saccades to the model (top). Spatial representation of the IoR with distinct size (bottom)

**References**

1. Li Z. A neural model of contour integration in the primary visual cortex. Neural computation. 1998 May 15;10(4):903–40.
2. Penacchio O, Otazu X, Dempere-Marco L. A neurodynamical model of brightness induction in v1. PloS one. 2013 May 22;8(5):e64086.
3. Berga D, Otazu X. A Neurodynamical model of Saliency prediction in V1. arXiv preprint arXiv:1811.06308. 2018 Nov 15.
4. Penacchio O, Wilkins AJ, Otazu X, Harris JM. Inhibitory function and its contribution to cortical hyperexcitability and visual discomfort as assessed by a computation model of cortical function. InPerception 2016 Aug 1 (Vol. 45, pp. 272–272).
5. Cerda-Company, Xim, Otazu X. A Multi-Task Neurodynamical Model of Lateral Interactions in V1: Chromatic Induction. InPERCEPTION 2016 Aug 1 (Vol. 45, pp. 52–53).

## P239 Long-range recurrent connectivity – cost-effective circuit for natural image perception

### Seungdae Baek, Youngjin Park, Se-Bum Paik

#### Korea Advanced Institute of Science and Technology, Department of Bio and Brain Engineering, Daejeon, South Korea

##### **Correspondence:** Seungdae Baek (seung7649@kaist.ac.kr)

*BMC Neuroscience* 2019, **20(Suppl 1)**:P239

Both the human brain and recent deep neural networks (DNNs) perform exceptionally well in natural image recognition [1]. However, the visual cortex (ventral stream: 8 layers) is composed of a much smaller number of layers and more sparse connections than DNNs (ResNet: 152 layers), presumably due to the limited volume of the brain [2]. What is the strategy of the brain to construct a high-functioning network for natural image perception using a much smaller number of inter-neural connections than those in a DNN? Here, we suggest that long-range horizontal connectivity, which has been observed in the early visual cortex of various mammals, is a critical factor in the cost-effectiveness for natural image perception. We hypothesize that long-range connections can be spontaneously evolved in the network under the condition that minimizing the number of connections while maintaining the ability to accurately recognize natural images. To validate our hypothesis, we designed a three-layer neural network that has lateral connections and convergence feedforward connectivity implementing the receptive field (RF), as a simplified model of the visual pathway. The objective function of the system was defined as an error minimization term for image classification added by a connection length-penalty term, which represents the visual recognition function and physical length constraint of the brain, respectively. After optimizing the network using natural images, we observed that most of the initial connections had disappeared, but a few long-range connections (LRCs), which are defined as the connections longer than the diameter of RF, remained. The statistics of the developed connectivity were similar to those observed in primate’s V1 [3]. Remarkably, the number of LRCs optimized for natural images was significantly larger than the number of LRCs optimized for random images. Disconnecting the LRCs in the trained network noticeably reduced performance for the natural image classification compared to that for random connections. Taken together, these results imply that LRCs play an important role in natural image perception in networks with limited connection length. To find which features of natural images are extracted by LRCs, we designed new image sets in which a pair of numeric images (local feature) are placed in different positions (global feature). We found that the ratio of LRCs to optimal recurrent connectivity increased when the network was trained by images with longer feature distance. When LRCs were deleted on the optimized networks, the accuracy of image classification was further reduced for networks trained by images with longer feature distance. This suggests that long-range connections contribute to image classification by extracting the low-frequency components of natural images. Overall, we propose that long-range recurrent connectivity found in the visual pathway is a critical component to optimize the balance between maximizing natural image perception performance and minimizing wiring cost. We believed that long-range recurrent connectivity can be used as a new module of DNN architecture with high connection efficiency.

**References**

1. He K, Zhang X, Ren S, Sun J. Deep residual learning for image recognition. *In Proceedings of the IEEE conference on CVPR* 2016 (pp. 770–778).
2. Samu D, Seth AK, Nowotny T. Influence of wiring cost on the large-scale architecture of human cortical connectivity. *PLoS computational biology* 2014 Apr 3;10(4):e1003557.
3. Horvát S, Gămănuț R, Ercsey-Ravasz M, et al. Spatial embedding and wiring cost constrain the functional layout of the cortical network of rodents and primates. *PLoS biology* 2016 Jul 21;14(7):e1002512.

## P240 Extracting whole-brain functional subnetworks for the prediction of cognitive and clinical conditions

### Andrea Insabato^1^, Matthieu Gilson^2^, Vicente Pallarés^1^

#### ^1^Universty Pompeu Fabra, Barcelona, Spain; ^2^Universitat Pompeu Fabra, Center for Brain and Cognition, Barcelona, Spain

##### **Correspondence:** Andrea Insabato (a.insabato@gmail.com)

*BMC Neuroscience* 2019, **20(Suppl 1)**:P240

Discovering functional networks underlying different cognitive states has become a crucial goal in neuroimaging over the last decade. The urgency is even larger for clinical studies where knowing the specific links involved in pathological conditions can push forward our understanding of brain diseases and provide better diagnostic and prognostic tools. Brain atlases have become more detailed with an increasingly larger number of brain regions. As a consequence functional brain networks have become larger and the problem of finding sets of links (subnetworks) related to cognitive or pathological conditions have become more complex.

Here we propose a method to extract such subnetworks based on Machine Learning tools. In particular, we frame the problem as a *feature selection* [1] in the context of supervised learning. Each functional connectivity matrix can be considered as one sample in the high dimensional space of functional links. Supervised learning can be used to fit a model to map from the links space to the cognitive or pathological labels associated to each matrix. In this context each link can be ranked in order of relevance for the prediction of labels. Here we compare different ranking methods: univariate information filter [1], infinite feature selection [2] and recursive feature elimination [3]. Finally we propose a method to automatically select the subset of links that best support the prediction of labels. We show applications of the method with controlled synthetic datasets, where the performance of the method can be easily understood and with a real dataset to distinguish different cognitive states.

**References**

1. Guyon I, Elisseeff A. An introduction to variable and feature selection. *Journal of machine learning research* 2003;3(Mar):1157–82.
2. Roffo G, Melzi S, Cristani M. Infinite feature selection. *In Proceedings of the IEEE International Conference on Computer Vision* 2015 (pp. 4202–4210).
3. Pallarés V, Insabato A, Sanjuán A, et al. Extracting orthogonal subject-and condition-specific signatures from fMRI data using whole-brain effective connectivity. *Neuroimage* 2018 Sep 1;178:238–54.

## P241 State transition network analysis of the resting state human brain cortex

### Jiyoung Kang, Hae-Jeong Park

#### Yonsei University, College of Medicine, Seoul, South Korea

##### **Correspondence:** Jiyoung Kang (jiyoungkang01@gmail.com)

*BMC Neuroscience* 2019, **20(Suppl 1)**:P241*BMC Neuroscience* 2019, **20(Suppl 1)**:P24

The resting state brain is often modelled as a dynamic system transitioning among multiple coexisting stable states. Despite the increasing number of studies on the multistability of the brain system, the state transition processes of the brain, which are essential to understanding the dynamic characteristics of the brain system, have rarely been explored. Thus, in the present study, we investigated the state transition processes of the large-scale cortical brain system by constructing state transition networks. The state transition processes were analyzed based on the graph-theoretical perspective.

To obtain state transition pathways and their transition rates, we performed energy landscape analysis, adopting a pairwise maximum entropy model (MEM). For the estimation of the model parameters of the MEM, the resting state fMRI (rs-fMRI) data from the Human Connectome Project (HCP) database were used. We extracted local minima and optimal pathways among them in the energy landscape, explored the characteristics of the brain state transition processes from the graph-theoretical perspective. In the state transition network, brain microstates, i.e., brain activity patterns, were assigned as nodes, and transitions and transition rates between two states (nodes) as edges were assigned as edges and their weights.

Three types of state transition networks were introduced in this study; 1) a state transition network among full states (STN-FS), 2) a state transition network from local minima toward the global minimum (STN-GM), and 3) a state transition network among rate-determining transition states and local minima states (STN-LM). The STN-FS includes all states that were utilized in paths, the STN-GM is a reduced network in that all remained states are connected to the global minimum, and the STN-LM is the simplified network that only includes stable and transition states, which is determine the transition rate. Finally, we constructed perturbed systems to evaluate robustness of the dynamic properties of the state transition processes in the resting state.

As a result, we found that the cortical brain system at rest contains multiple stable states that are clustered into two major state groups. The transition between brain states in the two groups was mediated by a frequent transition state, which operated as a hub of the transition network. State transition in the brain appears to involve multi-step state transitions, with some stable states serving as intermediate states for the complete transition. We also found that the baseline cortical brain system at rest shows a more complex and organized state transition network than those of artificially perturbed systems. This network approach to the state transition in the brain may provide a new framework for exploring the brain dynamics.

**Acknowledgements:** This research was supported by Brain Research Program through the National Research Foundation of Korea (NRF) funded by the Ministry of Science and ICT (NRF-2017M3C7A1049051).

## P242 Optimization and validation of a point neuron model to simulate the activity of olivocerebellar neurons

### Alice Geminiani^1^, Egidio D’Angelo^2^, Claudia Casellato^2^, Alessandra Pedrocchi^1^

#### ^1^Politecnico di Milano, Department of Electronics, Information and Bioengineering, Milan, Italy; ^2^University of Pavia, Dept. of Brain and Behavioral Sciences - Unit of Neurophysiology, Pavia, Italy

##### **Correspondence:** Alice Geminiani (alice.geminiani@polimi.it)

*BMC Neuroscience* 2019, **20(Suppl 1)**:P242

Introducing realistic firing properties of single neurons in large-scale simulations of Spiking Neural Networks is a fundamental challenge in computational neuroscience. In fact, not only connectivity and plasticity but also the variety of neuron spiking patterns has been proved fundamental for eliciting complex network activity, neural signal transmission and, eventually, behavior generation. This is particularly important for the cerebellum, where a rich variety of neurons is present, each one with a specific set of electroresponsive features [1]. In view of large-scale simulations with limited computational load and high biological plausibility, an extended generalized leaky integrate-and-fire (E-GLIF) point neuron has been developed [2], using NEST. Thanks to the intrinsic currents (endogenous and spike-triggered—depolarizing; adaptive—mainly hyperpolarizing) and the coupling between membrane potential and adaptive current, E-GLIF is able to reproduce autorhythm, linear slope between response frequency and input current (f-Iin), adaptation and bursting mechanisms, phase reset, intrinsic self-sustained oscillations of the membrane potential and resonance. Based on the gradient-based optimization tool developed for cerebellar Golgi cells [2], we here optimized E-GLIF to reproduce the f-Iin relationship and the post-inhibitory response of olivo-cerebellar neurons (Molecular layer interneurons, Granule, Purkinje, Cerebellar nuclei and Inferior olive neurons). The cell-specific E-GLIFs were then validated in *PyNEST* simulations, where different input current steps were provided to test the remaining spiking patterns. Specifically, Granule cell E-GLIF showed theta-band subthreshold oscillations, causing resonance in the same frequency band when stimulated with periodic spike trains. Molecular layer interneuron E-GLIF exhibited low-frequency autorhythm with zero-input current, while Purkinje cells were characterized by high-frequency spontaneous simple spikes, and complex spikes were triggered by high-amplitude current pulses. Post-inhibitory rebound excitation was reproduced in cerebellar nuclei E-GLIF, before returning to the 30-Hz autorhythm. Inferior olive neurons exhibited intrinsic subthreshold sinusoidal oscillations of membrane potential, phase reset and bursts of two/three spikes with depolarizing input currents.

The E-GLIF point neuron was able to capture the variety of firing patterns of olivocerebellar neurons. For each neuron we identified a set of model parameters allowing to generate cell-specific spiking responses depending on the provided input stimulus, like in experimental conditions. Thus, the optimized neurons can be used in large-scale simulations of the olivocerebellar circuit, eventually embedded in closed-loop control systems to reproduce sensorimotor behaviors [1]. The resulting multiscale tool would allow to link detailed single neuron dynamics to network and behavioral properties.

**Acknowledgements**: This work was developed within the HBP Partnering Project *CerebNEST* and was supported by European Union’s Horizon 2020 under Specific Grant Agreement No. 785907 (Human Brain Project SGA2).

**References**

1. D’Angelo E, et al. Modeling the Cerebellar Microcircuit: New Strategies for a Long-Standing Issue. *Frontiers in Cellular Neuroscience* 2016, 10, 1–29
2. Geminiani A, et al. Complex Dynamics in Simplified Neuronal Models: Reproducing Golgi Cell Electroresponsiveness. *Frontiers in Neuroinformatics* 2018, 12, 1–19

## P243 Modelling complex cells of early visual cortex using predictive coding

### Angelo Franciosini, Victor Boutin, Laurent Perrinet

#### CNRS - Aix-Marseille Université, Institut de Neurosciences de la Timone, Marseille, France

##### **Correspondence:** Angelo Franciosini (angelo.franciosini@univ-amu.fr)

*BMC Neuroscience* 2019, **20(Suppl 1)**:P243

Predictive Coding (PC) is an influential framework introduced by [1] to model neural processes in the primary visual cortex of mammals (V1). PC exploits the hierarchical structure of sensory information into a bi-directional update scheme: Higher-level cortical layers predict at best the activity of the lower-level ones and send the prediction through feedback connections. This prediction is compared to the activity of the lower-level layers to generate a prediction error that is sent to the upper layer through feed-forward connections. Interestingly, PC gives a possible explanation to extra-classical receptive fields effects in V1, this is also in line with the abundance of feedback connectivity in the brain. Additionally, this model has provided an elegant way to model task-driven learning in the brain by approximating error back-propagation, commonly used in deep neural networks, only by means of Hebbian plasticity and local computations. When implemented in a recurrent neural network, with the addition of sparsity constraints, PC can explain the emergence of edge sensitive cells in low-level visual areas as well as more specific descriptors in higher cortical areas. We show that such a model, called Sparse Deep Predictive Coding network (SDPC), can also account for the topological organization of the primary visual cortex when imposing a max-pooling operator across small groups of neurons. Moreover, we show that the resulting model encodes for edges of specific orientation independently of their phase, a behaviour analogous to the one observed in neural recordings of complex cells.

**Reference**

1. Rao RP, Ballard DH. Predictive coding in the visual cortex: a functional interpretation of some extra-classical receptive-field effects. *Nature Neuroscience* 1999 Jan;2(1):79.

## P244 Distributed representations and learning in neuronal networks

### Matthieu Gilson^1^, David Dahmen^2^, Ruben Moreno-Bote^3^, Moritz Helias^2^, Andrea Insabato^1^, Jean-Pascal Pfister^4^

#### ^1^Universitat Pompeu Fabra, Center for Brain and Cognition, Barcelona, Spain; ^2^Jülich Research Centre, Institute of Neuroscience and Medicine (INM-6), Jülich, Germany; ^3^Universitat Pompeu Fabra, Center for Brain and Cognition & DTIC, Barcelona, Spain; ^4^ETH Zurich, Zurich, Switzerland

##### **Correspondence:** Matthieu Gilson (matthieu.gilson@upf.edu)

*BMC Neuroscience* 2019, **20(Suppl 1)**:P244

Many efforts in the study of the brain have focused on representations of stimuli by neurons and learning thereof. This presentation will build on two of our recent works to propose a novel perspective for the processing of spike-based representations in neuronal networks.

The first work [1] provides a mathematical description of the propagation of high-order moments of spiking activity in Hawkes processes, also known as Poisson neurons. Our approach describes the spatio-temporal filtering induced by the afferent and recurrent connectivities using operator theory. Our algebraic viewpoint provides intuition about how the network ingredients shape the input-output mapping for moments, as well as cumulants.

The second work [2] focuses on learning an input-output mapping in a network based on second-order statistics, namely spatio-temporal covariances. Relying on the multivariate autoregressive (MAR) dynamics, our theory derives the weight update such that input covariance patterns are mapped to given objective output covariance patterns. It can be as an extension of the classical perceptron [3], a central concept that has brought many fruitful theories in the fields of neural coding and learning in networks. As an example, it performs the categorization of fluctuating time series determined by their hidden dynamics. Conceptually, variability in the time series is the basis for information to be learned, via the co-fluctuations that result in second-order statistics. Our approach is thus a radical change of perspective compared to classical approaches that typically transform time series into a succession of static patterns where fluctuations are noise.

After presenting the key aspects on these two studies, we will envisage future steps that join them toward a novel theory where information is conveyed by high-orders in the spike trains. In particular, we will illustrate the concepts with examples in spiking networks.

**References**

1. Gilson M, Pfister JP. Propagation of moments in Hawkes networks. *(preprint) arXiv* http://arxiv.org/abs/1810.09520
2. Gilson M, Dahmen D, Moreno-Bote R, Insabato A, Helias M. The covariance perceptron: A new framework for classification and processing of time series in recurrent neural networks. *(preprint) bioRxiv* http://doi.org/10.1101/562546
3. Bishop CM. Pattern Recognition and Machine Learning. Springer 2006.

## P245 Electrical synapses shape responses to transient inputs to canonical circuits

### Julie Haas^1^, Tuan Pham^2^

#### ^1^Lehigh University, Dept. of Biological Sciences, Bethlehem, PA, United States of America; ^2^University of Chicago, Graduate Program in Computational Neuroscience, Chicago, IL, United States of America

##### **Correspondence:** Julie Haas (julie.haas@gmail.com)

*BMC Neuroscience* 2019, **20(Suppl 1)**:P245

As information about the world traverses the brain, the signals exchanged between neurons are passed and modulated by synapses, or specialized contacts between neurons. While neurotransmitter-based synapses tend to exert either excitatory or inhibitory pulses of influence on the postsynaptic neuron, electrical synapses, composed of plaques of gap junction channels, continuously transmit signals that can either excite or inhibit a coupled neighbor. A growing body of evidence indicates that electrical synapses, similar to their chemical counterparts, are modified in strength during physiological neuronal activity. The synchronizing role of electrical synapses in neuronal oscillations has been well established, but their impact on transient signal processing in the brain is much less understood.

To investigate the impact of electrical synapses on transient signals, we constructed computational models based on the canonical feedforward neuronal circuit, wherein two principal neurons, connected by an excitatory synapse, are also connected by disynaptic feedforward inhibition (See Fig. 1). We used Izhikevich-type neurons for all cell types. We progressively expanded models and analysis from a single circuit to a pair of circuits, and finally to network composed of canonical circuits. We provided these models with single closely timed inputs, in order to determine how the embedded electrical and inhibitory synaptic connections between interneurons influence subthreshold integration and spiking statistics at the output stage of the model.

**Fig. 1:**
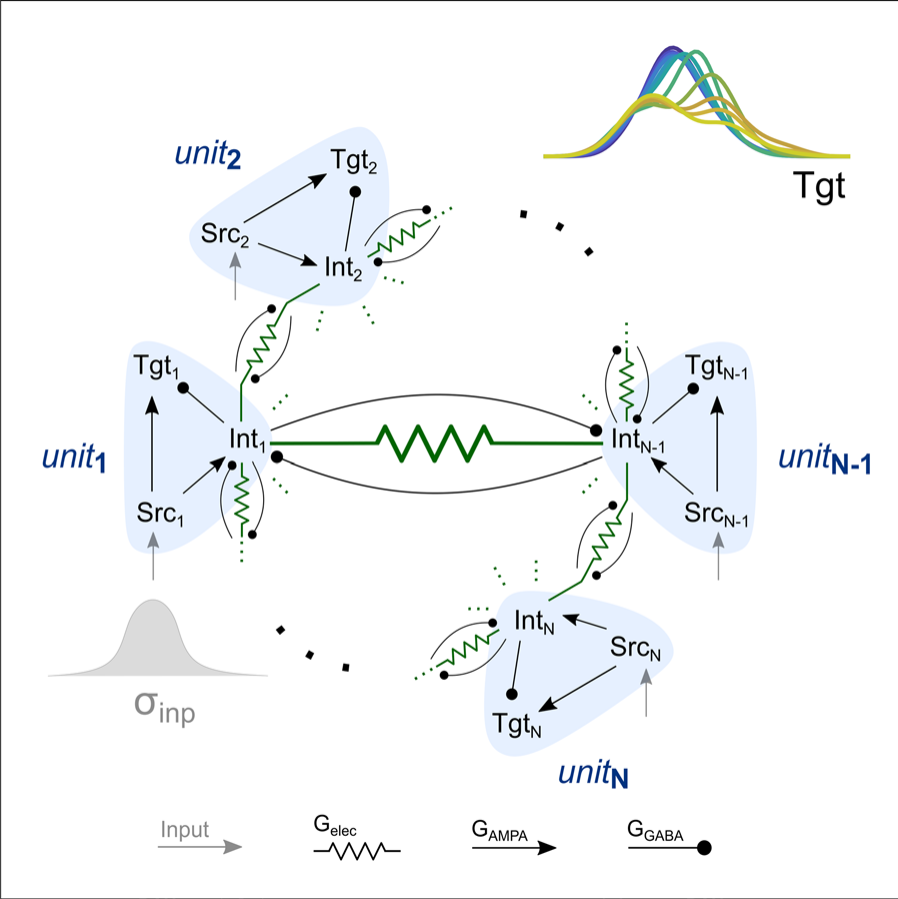
Network of coupled canonical circuits

Our simulations highlight the diverse and powerful roles that electrical synapses play even in simple circuits. In pairs of canonical circuits, electrical synapses either delay interneuron spiking by increasing leak or act to coordinate interneuron spiking, with substantial impacts in both cases on output principal neuron summation. The presence or absence of chemical synapses between electrically coupled interneurons also shapes subthreshold summation at the output stage. In networks of canonical circuits, electrical coupling between interneurons can delay, accelerate, and sharpen spiking of output principal neurons, depending on connection strength.

Because these canonical circuits are represented widely throughout the brain, we expect that these are general principles for the influence of electrical synapses on transient signal processing across the brain.

**Acknowledgements**: NSF IOS 1557474, the Whitehall Foundation (JSH).

## P246 Sensory processing and abstract categorization in cortical and deep neural networks

### Dimitris Pinotsis

#### City–University of London and MIT, London, United Kingdom

##### **Correspondence:** Dimitris Pinotsis (pinotsis@mit.edu)

*BMC Neuroscience* 2019, **20(Suppl 1)**:P246

Many recent advances in artificial intelligence (AI) are rooted in neuroscience. For example, a large body of AI work has used ideas from neuroscience to build neural networks that can perform sensory information processing and reinforcement learning tasks. However, ideas from more complicated paradigms like perceptual decision-making tasks are less used. Although automated decision-making systems are ubiquitous in modern applications (driverless cars, pilot support systems, medical diagnosis algorithms etc.) achieving human-level performance is still a challenge. Humans can effortlessly accomplish decision making tasks that are deemed difficult for artificial systems. Thus, understanding complex decision-making dynamics in the brain and modeling them using deep neural networks could open new avenues to tackle these difficulties. Here we used multivariate methods and deep recurrent neural networks to model some of the complex neural interactions during sensorimotor decision making in primate brain. We investigated how brain dynamics flexibly represented and distinguished between sensory processing and categorization in two different sensory domains: motion direction and color. We found that representations changed depending on context. Selectivity of each brain area depended not only on the stimulus represented but also on the domain of categorization. We trained deep recurrent neural networks with monkey LFP recordings and found that they could process sensory information and perform categorization in the motion and color domains similarly to the animals. By comparing brain dynamics with network predictions, we found that computations in different brain areas also changed flexibly between the two tasks. Color computations appeared to rely more on sensory processing, while motion computations more on categorization. Overall, our results shed light to the biological basis of categorization and differences in selectivity and computations in different brain areas.

## P247 Substantia nigra pars compacta dopamine axons die back; a bioenergetic model to explain mechanisms of degeneration in Parkinson’s disease

### Teresa Ruiz Herrero^1^, Eleftheria Pissadaki^2^

#### ^1^Biogen Inc, Quantitative Medicine and Clinical Technologies, Research and Development, Cambridge, MA, United States of America; ^2^Biogen Inc, Quantitative Medicine and Clinical Technologies, Cambridge, MA, United States of America

##### **Correspondence:** Eleftheria Pissadaki (eleftheria.pissadaki@biogen.com)

*BMC Neuroscience* 2019, **20(Suppl 1)**:P247

The cellular hallmark of Parkinson’s disease is the selective and progressive loss of dopaminergic neurons in the substantia nigra *pars compacta* (SNc). While their degeneration is associated with the appearance of the cardinal motor symptoms of the disease, the etiopathology of their loss remains unknown. The massive, profusely branched and unmyelinated axons of dopamine neurons, has given rise to the hypothesis that their selective vulnerability is a consequence of the axon structure and morphological characteristics. Indeed, detailed biophysical modeling has shown that bioenergetic requirements elevate in a power law fashion as a function of the size of the axon arborization and the number of branches (complexity) that an SNc dopamine axon establishes. Furthermore, it has been shown that the distal terminals are energy inefficient, meaning that sodium channels can conduct superfluous sodium current during the upstroke phase of the action potential, requiring extra Na+/K+ATP-ase activity to recover membrane potential. Here we sought to examine the hypothesis that the thin caliber axon segments render an increased surface to volume ratio, associated with elevated Na+/K+ATP-ase activity, that leads to incomplete sodium and calcium gradient replenishment inducing toxicity that could lead to axonal and cellular degeneration. We implemented a biophysical model of ion and mitochondrial diffusion coupled to channel activity at the axon compartment level to examine the above hypothesis. A successful validation of the hypothesis may shed light to the enigma as to why SNc dopamine neurons and their axon terminals degenerate in Parkinson’s disease.

## P248 Breakdown of spatial coding and neural synchronization in epilepsy using a computational model

### Spyridon Chavlis^1^, Ioanna Pandi^2^, Tristan Shuman^3^, Daniel Aharoni^4^, Denise Cai^3^, Peyman Golshani^4^, Panayiota Poirazi^5^

#### ^1^Foundatuion for Research and Technology-Hellas, Institute of Computer Science, Heraklion, Greece; ^2^University of Crete, School of Medicine, Heraklion, Greece; ^3^Icahn School of Medicine at Mount Sinai, Department of Neuroscience and Friedman Brain Institute, New York, United States of America; ^4^University of California, Department of Neurology, David Geffen School of Medicine, Los Angeles, United States of America; ^5^FORTH, IMBB, Heraklion, Greece

##### **Correspondence:** Spyridon Chavlis (schavlis@ics.forth.gr)

*BMC Neuroscience* 2019, **20(Suppl 1)**:P248

Temporal lobe epilepsy causes significant cognitive deficits in both humans and rodents, yet the specific circuit mechanisms underlying these deficits remain unknown. There is profound and selective interneuron death and axonal reorganization within the hippocampus of both humans and animal models of temporal lobe epilepsy.

To assess the specific contribution of these mechanisms on spatial coding, we developed a biophysically constrained network model of the CA1 region that consists of different subtypes of cells [1]. More specifically, our network consists of 150 cells, 130 excitatory pyramidal cells and 20 interneurons (Fig. 1). To simulate place cell formation in the network model, we generated grid cell and place cell inputs from the Entorhinal Cortex (ECLIII) and CA3 regions, respectively, activated in a realistic manner as observed when an animal transverses a linear track. Realistic place fields emerged in a subpopulation of pyramidal cells (40–50%), in which similar EC and CA3 grid cell inputs converged onto distal/proximal apical and basal dendrites. The tuning properties of these cells are very similar to the ones observed experimentally in awake, behaving animals

**Fig. 1:**
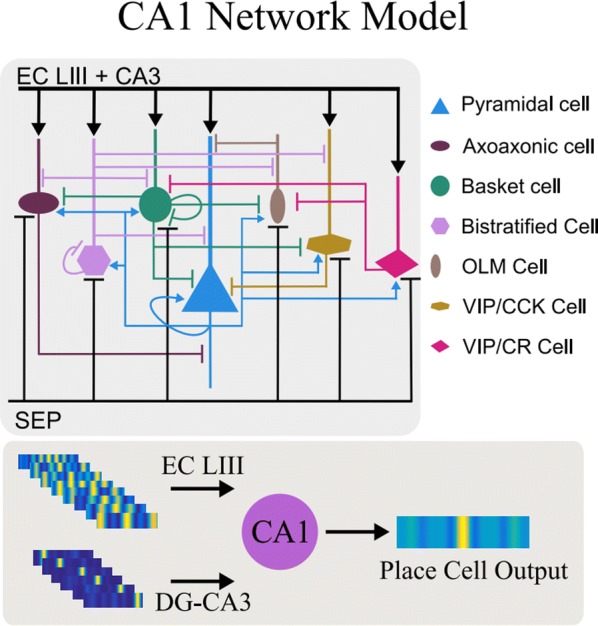
(Top) Schematic diagram of CA1 computational network model, (bottom) Grid-like and place-like inputs to CA1 pyramidal cells

To examine the role of interneuron death and axonal reorganization in the formation and/or tuning properties of place fields we selectively varied the contribution of each interneuron type and desynchronized the two excitatory inputs. We found that desynchronized inputs were critical in reproducing the experimental data, namely the profound reduction in place cell numbers, stability and information content [2]. These results demonstrate that desynchronized firing of hippocampal neuronal populations contributes to poor spatial processing in epileptic mice, during behavior. Given the lack of experimental data on the selective contributions of interneuron death and axonal reorganization in spatial memory, our model findings predict the mechanistic effects of these alterations at the cellular and network levels.

**Acknowledgments:** We thank Stavros Niarchos Foundation – FORTH Fellowships for supporting Spyridon Chavlis with ARCHERS project: Advancing Young Researchers’ Human Capital in Cutting Edge Technologies in the Preservation of Cultural Heritage and the Tackling of Societal Challenges.

**References**

1. Turi GF, Li W-K, Chavlis S, et al. Vasoactive Intestinal Polypeptide-Expressing Interneurons in the Hippocampus Support Goal-Oriented Spatial Learning. *Neuron* 2019, In Press.
2. Shuman T, Aharoni D, Cai DJ, et al. Breakdown of spatial coding and neural synchronization in epilepsy. *bioRxiv* 2018.

## P249 Structured connectivity exploits NMDA-non-linearities to induce diverse responses in a PFC circuit.

### Stefanos S. Stefanou, Athanasia Papoutsi, Panayiota Poirazi

#### FORTH, IMBB, Heraklion, Greece

##### **Correspondence:** Athanasia Papoutsi (athpapoutsi@yahoo.gr)

*BMC Neuroscience* 2019, **20(Suppl 1)**:P249

Prefrontal Cortex (PFC) exerts control on action selection and mediates behavioral flexibility. This flexibility is imperative during working memory (WM), when stimuli retention and integration takes place. Neurons of the PFC exhibit mixed selectivity to stimuli, yet the mechanisms that enable them to rapidly modify their response properties in a context-dependent manner remain poorly understood. How can a recurrently and sparsely connected network perform both stimulus selection and integration in a way that appears highly dynamic at the single neuron level, yet is stable and separable at the population level remains an open question.

To answer this question we hypothesize that: a) it is possible to have neurons displaying this highly dynamic behavior (e.g. via encoding both ‘sensory’ and ‘memory’ signals) in a recurrently connected network and b) rapid synaptic facilitation via NMDARs enables this behavior in a network constrained by the highly reciprocal and clustered connectivity that is prominent in PFC.

We test this hypothesis using detailed biophysical models of PFC neuronal networks, which are constrained in their physiological and connectivity properties and reproduce the key features of single neuron and population processing. Our simulations show that NMDA properties of L5 PFC neurons are appropriate for exploiting the structured connectivity profile in order to implement highly dynamic, yet discrete WM representations in the PFC. Specifically, we predict that both NMDA nonlinearities and a structured connectivity are needed to produce multiple, flexible states thus maintaining WM information in a dynamical way while also exhibiting robustness in time.

Our results (Fig. 1) are summarized in: a) a network model that reproduces the complexity of population responses as well as the emergence of low energy stable states during the WM period. b) This model can respond with different stable states, in response to different stimulus applied, through a rapid, internal connectivity reconfiguration. c) This reconfiguration is mediated by dendritic nonlinearities, since eliminating them abolishes the discrimination abilities of the network. d) This reconfiguration is observed more prominently in a network connected as the PFC anatomy indicates, compared to a randomly connected network. e) We note that the aforementioned stable states can in principle be attributed to dynamically recruited neuronal ensembles or to combinations of them, that respond stably in time.

**Fig. 1:**
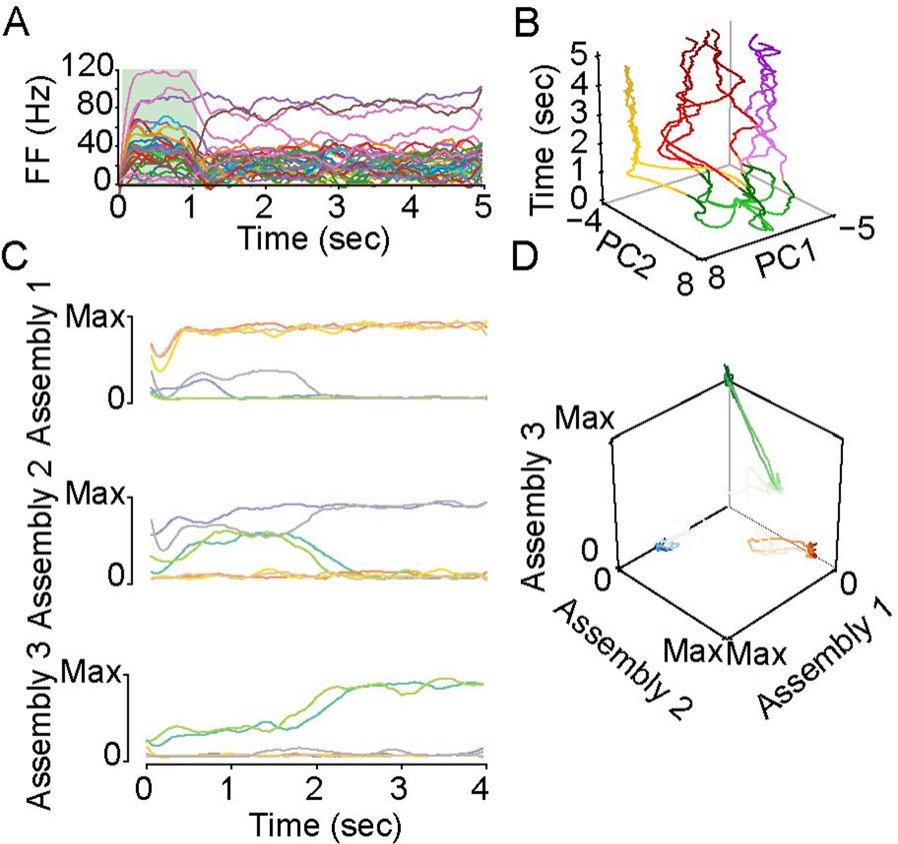
**a** Dynamic single neuron responses. **b** Activity induced by different stimuli settles in discernable states (PCA space). **c** Activity decomposed into three assemblies, color coded per stimulus. **d** Same as **c**, color coded for different states

**Acknowledgments:** This work was supported by the Fondation Santé, the Hellenic Foundation for Research and Innovation (HFRI) and the General Secretariat for Research and Technology (GSRT), under grant agreement 1357 and by the European Research Council, ERC Starting Grant dEMORY (ERC-2012-StG-311435).

## P250 Spiking patterns in the zebrafish larvae

### Nicolas Doyon^1^, Patrick Desrosiers^2^, Simon V. Hardy^3^, Jean-Christophe Rondy-Turcotte^1^, Jasmine Poirier^4^

#### ^1^Université Laval, Mathematics and Statistics, Quebec, Canada; ^2^Laval University, Département de physique, de génie physique et d’optique, Quebec, Canada; ^3^Laval University, Département d’informatique et de génie logiciel, Quebec, Canada; ^4^Laval University, Physics, Quebec, Canada

##### **Correspondence:** Nicolas Doyon (nicolas.doyon@mat.ulaval.ca)

*BMC Neuroscience* 2019, **20(Suppl 1)**:P250

A difficult challenge in neuroscience is to determine the functional connectivity of neural networks. Nature provides us a with a small but valuable ally in the shape of the zebrafish larvae. Thanks to the transparent skull of the animal, the brain of zebrafish larvae is amenable to optic investigation. Once the neurons are filled with a calcium sensitive dye, the in vivo activity of these neurons become measurable without any alteration to the natural behaviour. We have built a multidisciplinary team to investigate the structure of the zebrafish neural network. We’re establishing a pipeline running from animal care and data acquisition to spike inference and network reconstruction (Fig. 1). First, fast scanning microscopes allow real time data acquisition in large 3D brain structures with high frequency (around 30 Hz). Then, a homemade flexible segmentation program gives the time series of fluorescence measured simultaneously in (for now) more than 300 neurons. Such an approach has been pioneered by the group of Ahrens [1]. We believe that our original data set will provide insights on the properties of neural networks. Connectivity can be determined either directly from the correlations in calcic activity or from correlations in the inferred spike trains. To infer individual spike trains from the fluorescence, we tailored an algorithm developed by Deneux et al. [2]. Given an experimental time series, this algorithm recursively searches for the spike train most likely to explain it, using a hidden layer Markov model. The fluorescence data presents difficulties: slow drift in the baseline signal, presence of noise and acquisition frequency all limit the accuracy of spike detection. To estimate the failure rate, we simulated synthetic data sharing the most relevant aspects of experimental data such as noise amplitude and signal strength. The estimated error is a 30 percent probability of failing to detect an actual spike and a 7 percent probability that a detected spike isn’t an actual one. The aggregated mean spike rate is 4 Hz with important oscillations and spike rates above 15 Hz often sustained for periods of more than 1 second. The inferred spike trains lead to a natural classification of neurons into oscillating, noisy and one time active. Important correlations are observed between the activity of oscillating neurons. Our next goal is to use the inferred spike trains to deduce functional connections between neurons. We hope to uncover network statistics such as clustering coefficients or small world properties that may help explain the robustness and resilience of natural neural networks.

**Fig. 1:**
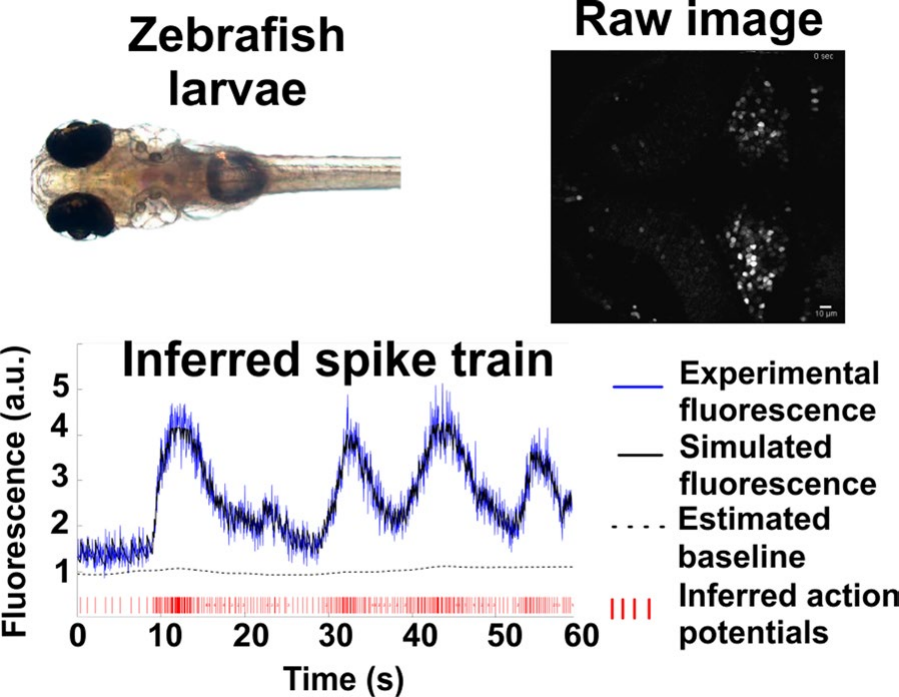
Top left: A picture of a zebrafish larvae. Top right: An example of raw fluorescence data. Bottom: An instance of spike inference

**Acknowledgements:** We thank the program Sentinel North, Canada First Research Excellence Fund.

**References**

1. Chen X, Mu Y, Hu Y, et al. Brain-wide organization of neuronal activity and convergent sensorimotor transformations in larval zebrafish. *Neuron* 2018 Nov 21;100(4):876–90.
2. Deneux T, Kaszas A, Szalay G, et al. Accurate spike estimation from noisy calcium signals for ultrafast three-dimensional imaging of large neuronal populations in vivo. *Nature communications* 2016 Jul 19;7:12190.

## P251 Impact of brain parcellation on parameter optimization of the whole-brain dynamical models

### Thanos Manos^1^, Sandra Diaz-Pier^2^, Felix Hoffstaedter^1^, Jan Schreiber^3^, Alexander Peyser^2^, Simon B. Eickhoff^1^, Oleksandr V. Popovych^1^

#### ^1^Research Centre Jülich, Institute of Neuroscience and Medicine (INM-7: Brain and Behaviour), Jülich, Germany; ^2^Jülich Research Centre, SimLab Neuroscience, Jülich, Germany; ^3^Research Centre Jülich, Institute of Neuroscience and Medicine, Structural and functional organization of the brain (INM-1), Jülich, Germany

##### **Correspondence:** Thanos Manos (t.manos@fz-juelich.de)

*BMC Neuroscience* 2019, **20(Suppl 1)**: P251

Recent progress in neuroimaging techniques has advanced our understanding of structural and functional properties of the brain. Resting-state functional connectivity (FC) analysis has brought new insights to the inter-individual variability [1]. Using diffusion-weighted magnetic resonance imaging, one can retrieve the basic features of the anatomical architecture of brain networks, i.e. structural connectivity (SC) [2]. Empirical SC (eSC) and FC (eFC) can be used to build and validate large-scale mathematical models of the brain dynamics being in the focus of research nowadays [3, 4]. In this work, we set out to investigate the impact of different brain atlases on the dynamics of the whole-brain computational models and their optimal parameters fitted to the neuroimaging data, resulting in the optimal agreement between empirical and simulated data. We considered a sample of 23 healthy subjects from the Human Connectome Project database [5] and 2 different brain atlases, the Harvard-Oxford structural atlas and the Schaefer functional atlas [6]. The large-scale network model of brain activity is based on an informed by eSC Kuramoto model [8] and is simulated using The Virtual Brain (TVB) platform [7], with an optimized code from TVB-HPC adequate for high-performance clusters computing. We found that the two considered atlases are in good agreement with respect to the optimal parameters (e.g. global coupling strength K) and the corresponding values of the correlation coefficient of the best correspondence between sFC and eSC. Moreover, the considered model can demonstrate relatively strong correlations between eSC and sFC matrices whereas the correspondence between eFC and sFC matrices is, however, weaker for both atlases [9].

**References**

1. Park HJ, Friston K. Structural and functional brain networks: from connections to cognition. *Science* 2013 Nov 1;342(6158):1238411.
2. Maier-Hein KH, Neher PF, Houde JC, et al. The challenge of mapping the human connectome based on diffusion tractography. *Nature communications* 2017 Nov 7;8(1):1349.
3. Popovych OV, Manos T, Hoffstaedter F, Eickhoff SB. What Can Computational Models Contribute to Neuroimaging Data Analytics? *Frontiers in systems neuroscience* 2018;12:68.
4. Deco G, Kringelbach ML. Metastability and coherence: extending the communication through coherence hypothesis using a whole-brain computational perspective. *Trends in neurosciences* 2016 Mar 1;39(3):125–35.
5. McNab JA, Edlow BL, Witzel T, et al. The Human Connectome Project and beyond: initial applications of 300 mT/m gradients. *Neuroimage* 2013 Oct 15;80:234–45.
6. Schaefer A, Kong R, Gordon EM, et al. Local-global parcellation of the human cerebral cortex from intrinsic functional connectivity MRI. *Cerebral Cortex* 2017 Jul 18;28(9):3095-114.
7. Sanz Leon P, Knock SA, Woodman MM, et al The Virtual Brain: a simulator of primate brain network dynamics. *Frontiers in neuroinformatics* 2013 Jun 11;7:10. (TVB-HPC: https://gitlab.thevirtualbrain.org/tvb/hpc).
8. Kuramoto Y. Chemical oscillations, waves, and turbulence. *Courier Corporation* 2003.
9. Manos T, Diaz-Pier S, Hoffstaedter F, Schreiber J, Eickhoff SB, Popovych OV. (*in preparation).*

## P252 Ion channel correlations emerge from the simultaneous regulation of multiple neuronal properties

### Jane Yang^1^, Steve Prescott^2^

#### ^1^University of Toronto & The Hospital for Sick Children, Institute of Biomaterials and Biomedical Engineering, Toronto, Canada; ^2^University of Toronto, Neurosciences and Mental Health & Dept. Physiology, Institute of Biomaterials and Biomedical Eng, Toronto, Canada

##### **Correspondence:** Jane Yang (strawcup@gmail.com)

*BMC Neuroscience* 2019, **20(Suppl 1)**: P252

Equivalent neuronal excitability can be achieved through different ion channel combinations. This is an example of degeneracy. Ion channel diversity is greater than required to regulate any one aspect of excitability, but multiple aspects of excitability plus other aspects of cell function (e.g. energy usage) must be regulated. Here we show that the number of tunable ion channels (*N*) must exceed the number of regulated outputs (*M*) to ensure degenerate solutions. We argue that degeneracy cannot be inferred from *N* alone, but, rather, that it depends on *N* relative to *M*. In other words, the dimensionality of the solution is defined by *N*–*M*.

Experimental work has revealed that the levels of certain ion channels are correlated. Theoretical work has revealed that such correlations can be explained based on the relative rates of underlying processes like transcription. Yet the existence of strong pairwise correlations is surprising if dimensionality of the solution is high. Consistent with this prediction, we show that correlations weaken as the number of tunable ion channels (*N*) is increased (Fig. 1A and B). On the other hand, correlations strengthen as the number of regulated outputs (*M*) is increased (Fig. 1B and C). Hence, the existence of ion channel correlations suggests that the dimensionality of the solution (*N*–*M*) is low. Since *N* is known to be high, this suggests that *M* is also high, or, in other words, that neurons are regulating multiple properties simultaneously.

**Fig. 1:**
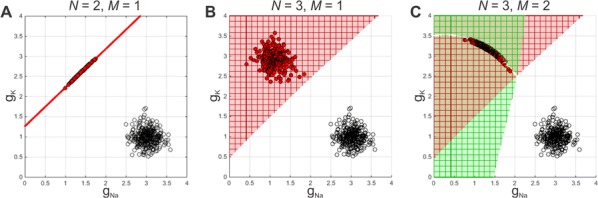
**a** Red dots show ion channel combinations (N = 2) producing a target rheobase of 100 microamps/cm^2^ (M = 1) found using a toy homeostatic rule. White dots show starting values. **b** Same as **a**, but N increased to 3; Red grid = iso-rheobase surface. **c** Same as **b**, but M increased to 2 for rheobase and ATP per spike (energy usage); Green grid = iso-ATP surface; dashed line = intersection

## P253 A new framework for modelling neural-ECM signaling and interaction

### Nicolangelo Iannella^1^, Anders Malthe-Sørensen^2^, Gaute Einevoll^3^, Marianne Fyhn^1^, Kristian Prydz^1^

#### ^1^University of Oslo, Department of Biosciences, Oslo, Norway; ^2^University of Oslo, Department of Physics, Oslo, Norway; ^3^Norwegian University of Life Sciences, Faculty of Science and Technology, Aas, Norway

##### **Correspondence:** Nicolangelo Iannella (nicolangelo.iannella@gmail.com)

*BMC Neuroscience* 2019, **20(Suppl 1)**: P253

Electrical activity in cortical networks and their composite neurons provides the basis for understanding the information processing in single neurons and interconnected neural populations [1]. To this end, numerous theoretical/computational studies have focused on how networks and their composite neurons operate from an electrical perspective [2, 3]. With the advent of molecular techniques, the underlying molecular activity also plays important role, where neurons and glia are now appreciated to be complex molecular machines, adapting their response properties over short and long-time scales [4]. Recent experiments have implicated the Extracellular Matrix (ECM) and its molecular components in neural signalling and information processing, by virtue that it strategically occupies the synaptic cleft [5, 6]. Recent evidence suggests the ECM is involved in Life-long learning and memory and significantly, in the recall of fear memory [7, 8]. Some have also shown that the expression of certain ECM molecules can modulate the efficacy of neural transmission on multiple time scales [9]. Currently, a handful of computational studies have focused on understanding the role of the ECM through bidirectional signalling and interaction between neurons and the ECM [10]. How this interaction impacts network behaviour and information processing has yet to be explored from a computational perspective.

We present a new biologically inspired framework and accompanying mathematical model that captures the nature of bidirectional neuron-ECM signalling. The framework considers the various roles played by various ECM molecules through their activity-driven influence on neural transmission and impact on neural responses. Our model is computationally tractable and can be applied to study the bidirectional nature of neural-ECM signalling in different brain areas and their collective influence on both single neuron responses and network activity.

**Acknowledgments:** The authors thank the Research Council of Norway for financial support via BRAINMATRIX NRF250259.

**References**

1. Kandel ER, Schwartz JH, Jessell TM. Principles of Neural Science (5th Ed), *McGraw-Hill New York* 2012.
2. Tuckwell HC. Introduction to theoretical neurobiology: Vol 1 and Vol 2. *Cambridge University Press*; 1988.
3. Brette R, Rudolph M, Carnevale T, et al. Simulation of networks of spiking neurons: a review of tools and strategies. *Journal of computational neuroscience* 2007 Dec 1;23(3):349–98.
4. Mataga N, Mizuguchi Y, Hensch TK. Experience-dependent pruning of dendritic spines in visual cortex by tissue plasminogen activator. *Neuron* 2004 Dec 16;44(6):1031–41.
5. Dityatev A, Schachner M, Sonderegger P. The dual role of the extracellular matrix in synaptic plasticity and homeostasis. *Nature Reviews Neuroscience* 2010 Nov;11(11):735.
6. Ferrer-Ferrer M, Dityatev A. Shaping synapses by the neural extracellular matrix. *Frontiers in Neuroanatomy* 2018;12.
7. Tsien RY. Very long-term memories may be stored in the pattern of holes in the perineuronal net. *Proceedings of the National Academy of Sciences* 2013 Jul 23;110(30):12456–61.
8. Thompson EH, Lensjø KK, Wigestrand MB, et al. Removal of perineuronal nets disrupts recall of a remote fear memory. *Proceedings of the National Academy of Sciences* 2018 Jan 16;115(3):607–12.
9. Huntley GW. Synaptic circuit remodelling by matrix metalloproteinases in health and disease. *Nature Reviews Neuroscience* 2012 Nov;13(11):743.
10. Kazantsev V, Gordleeva S, Stasenko S, Dityatev A. A homeostatic model of neuronal firing governed by feedback signals from the extracellular matrix. *PloS One* 2012 Jul 27;7(7):e41646.

## P254 In silico Spinal cord model shows the viability of targeting segmental foci along rostrocaudal axis for eliciting a variety of movement types

### Madhav Vinodh, Raghu Iyengar, Mohan Raghavan

#### Indian Institute of Technology, Hyderabad, Biomedical Engineering, Hyderabad, India

##### **Correspondence:** Mohan Raghavan (mohan.raghavan.s@gmail.com)

*BMC Neuroscience* 2019, **20(Suppl 1)**: P254

The motor plan generated in the brain is relayed to specific centers in the spinal cord via descending commands. These specific centers, in turn, recruit spinal modules consisting of neuronal circuits that are capable of producing and or modulating specific movement type independent of supraspinal input. This emphasizes the idea that descending commands can interact with spinal modules mostly to modulate and alter network dynamics while spinal modules work independently to produce activations in the array of muscles that are required by the movement type. Hence the modular organization of motor circuits is a smart and efficient design strategy by which the nervous system is capable of achieving complex motor activation. These spinal neuronal modules are spread across the rostrocaudal axis of the spinal cord and evidence from intraspinal stimulations on anaesthetized monkeys and cats explain the presence of such anatomical localizations. For e.g. Specific spinal foci responsible for particular movement types have been observed in animals. In decerebrate cats stimulating particular spinal foci resulted in activation of all muscles required for swinging the limb forward. Somatotopic maps thus generated are essential in understanding spinal circuitry in conjunction with spinal anatomy. Understanding circuit localizations specific to movement types is essential in designing stimulation therapies or rehabilitation planning after spinal injury. Although strong evidential models of spinal modules responsible for movement types are available but are restricted to rodent, monkey or cat models. Models on human spinal cord are rare owing to the experimental constraints. In the current study we tried to build an in-silico spinal cord model based on anatomical motor maps obtained from [1] study and combined with elementary spinal circuits proposed by [2-5]. The elementary spinal circuits generated within the model are based on movement synergy and circuit topology encompasses spinal reflex components and interneuronal pathways. The setup contains spinal anatomy + circuit model interacting with an external biomechanical model of the lower limb. Preliminary stimulation studies on the model revealed the segmental segregation of movement types along the rostrocaudal axis. While this has been established for some movement types in previous studies, we show that the same exists as a general principle covering all degrees of freedom in the lower limb. The movements that are strongly opposed or are major antagonists are found to be separated along the axis. For instance, hip_flexion, knee_extension, hip_adduction are localized in more rostral segments compared to hip_extension, knee_flexion, hip_abduction. This pattern is borne out in the figure (Fig. 1) where the diameters indicate the combined activity of all motor neurons responsible for a movement type in response to a stimulation along various segments. This organization has an important implication for descending connections and stimulation therapies namely that movement types may be triggered by selectively stimulating different spinal segments. The error probabilities of triggering antagonistic movements simultaneously are automatically controlled by the fact that strongly antagonistic joints/movement types are farther away along the rostrocaudal axis of the spinal cord. Open and closed loop model stimulation studies strengthen the claim of this general principle.

**Fig. 1:**
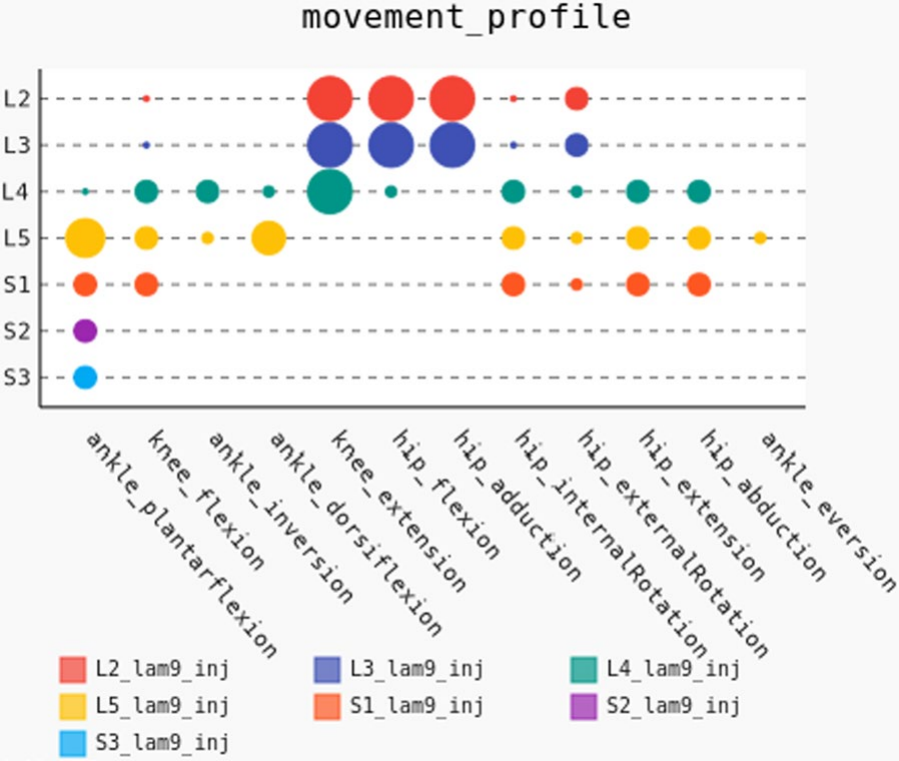
Movement types vs spinal segmental foci

**References**

1. Sharrard WJ. The distribution of the permanent paralysis in the lower limb in poliomyelitis: a clinical and pathological study. *The Journal of bone and joint surgery. British volume* 1955 Nov;37(4):540–58.
2. Jankowska E, Hammar I. Spinal interneurones; how can studies in animals contribute to the understanding of spinal interneuronal systems in man? *Brain Research Reviews* 2002 Oct 1;40(1–3):19–28.
3. Jankowska E. Interneuronal relay in spinal pathways from proprioceptors. *Progress in neurobiology* 1992 Apr 1;38(4):335–78.
4. Jankowska E. Spinal interneuronal systems: identification, multifunctional character and reconfigurations in mammals. *The Journal of physiology* 2001 May 1;533(1):31–40.
5. Pierrot-Deseilligny E, Burke D. The circuitry of the human spinal cord: spinal and corticospinal mechanisms of movement. *Cambridge University Press*; 2012 Apr 26.

## P255 Slow-gamma frequencies are optimally guarded against neurodegenerative impairments

### Pedro Maia^1^, J. Nathan Kutz^2^, Ashish Raj^1^

#### ^1^University of California San Francisco, Radiology and Biomedical Imaging, San Francisco, CA, United States of America; ^2^University of Washington, Department of Applied Mathematics, Seattle, WA, United States of America

##### **Correspondence:** Pedro Maia (pedro.doria.maia@gmail.com)

*BMC Neuroscience* 2019, **20(Suppl 1)**: P255

Neurodegeneration and traumatic brain injuries generate a broad array of pathologies across multiple spatial scales. At the most fundamental level, neuronal impairments distort the information encoded in spike trains, ultimately undermining the neuronal network’s functionality. We demonstrate that slow-gamma frequencies in the 38–41 Hz range are most robust to injury, independent of parameter and methodological choices in common neuron models. Our theory provides a unified signal-processing framework that accounts for major cellular-level pathologies that affect spike train propagation, including axonal swellings and demyelination. Specifically, we model with a family of discrete-time filters eight experimentally observed transformations that occur to injured spike trains, including increasing refractoriness and intermittent blockage. We also derive continuous counterparts for the filters by convolving neuronal firing rates from spike trains with Causal and Gaussian kernels. Our filters align with the spectrum of dynamic memory fields; Thus, working memory, visual consciousness, and other higher cognitive functions seem to operate in a frequency band that is optimally guarded against common types of pathological effects. In contrast, higher-frequency neural encoding, such as is observed with short-term memory, is greatly compromised by neurodegeneration and injury. We also propose impaired tuning curves for a few classic visual/motor studies to facilitate experimental validation of our results.

All impaired neurons seem to distort, confuse and/or block the information encoded in spike trains. Thus, it is possible to draw commonalities between the demyelinating effects of Multiple Sclerosis, some neurodegenerative developments in Alzheimer’s disease, and the axonal swellings following traumatic brain injuries. These incurable brain disorders are of great societal interest since they are respectively the most common type of autoimmune disease, dementia, and a leading source of death/disability among youngsters. We show that neural response frequencies in the slow-gamma range of 38—41 Hz are most insulated against common spike-train distortions [1]. This frequency band is involved in key functions: (a) the hippocampus is known to split its gamma oscillations in two distinct components, a fast band (@ 120 Hz) and slow band (@ 40 Hz). This separation is important to reliably distinguish the perceptions of ongoing experiences from the internally evoked memories [2]. Our results show that the slow band is optimally robust. (b) F. Crick and C. Koch made the 40-Hz oscillation the centerpiece of their theory of visual consciousness and awareness after several studies reported neuronal phase-locking in this frequency in the visual cortex [3]. (c) Changes in slow-gamma oscillations have been observed in several neurological disorders, but most prominently in rat models for Alzheimer’s Disease (AD). Iaccarino et al [4] showed that external stimulation of CA1 neurons at 40 Hz- and at 40 Hz only- attenuates pathologies associated with AD. We conjecture that if more neurons synchronize at robust frequencies, the overall capability of the system to transmit information should increase. Overall, we provide a compelling hypothesis as to why so many higher cognitive functions are encoded in the slow-gamma range.

**References**

1. Maia PD, Raj A, Kutz JN. Slow-gamma frequencies are optimally guarded against effects of neurodegenerative diseases and traumatic brain injuries. *Journal of computational neuroscience* 2019 Jun 4:1–6. 10.1007/s10827-019-00714-8
2. Colgin LL, Denninger T, Fyhn M, et al. Frequency of gamma oscillations routes flow of information in the hippocampus. *Nature* 2009 Nov;462(7271):353.
3. Crick F, Koch C. The problem of consciousness. *Scientific American* 1992 Sep 1;267(3):152–9.
4. Iaccarino HF, Singer AC, Martorell AJ, et al. Gamma frequency entrainment attenuates amyloid load and modifies microglia. *Nature* 2016 Dec;540(7632):230.

## P256 A mathematical investigation of chemotherapy induced peripheral neuropathy

### Parul Verma^1^, Achim Kienle^2^, Dietrich Flockerzi^2^, Doraiswami Ramkrishna^1^

#### ^1^Purdue University, Chemical Engineering, West Lafayette, IN, United States of America; ^2^Max Planck Institute for Dynamics of Complex Technical Systems, Magdeburg, Germany

##### **Correspondence:** Parul Verma (verma38@purdue.edu)

*BMC Neuroscience* 2019, **20(Suppl 1)**: P256

Chemotherapy-induced peripheral neuropathy (CIPN) is a prevalent, painful, dose-limiting toxicity which arises due to a number of chemotherapy drugs, such as vincristine, paclitaxel, and cisplatin. CIPN can be prolonged, affecting the quality of life of cancer patients. Moreover, sometimes, chemotherapy treatment is stopped altogether because of the severe pain, thereby affecting the treatment efficacy. Currently, there are no FDA-approved agents to prevent it. The lack of an established CIPN treatment stems from an incomplete understanding of its mechanism. The aim of this study is to fundamentally understand CIPN mechanism with a focus on voltage-gated ion channels.

The aforementioned chemotherapy drugs alter the expression of peripheral sensory neuron ion channels, which can lead to hyperexcitability [1]. Hyperexcitability is an abnormal discharge of action potentials and is a potential indicator of peripheral neuropathy *in vitro* [2]. To understand the role of voltage-gated ion channels in the onset of hyperexcitability, we analyzed a mathematical model that represents voltage dynamics in a small dorsal root ganglia neuron. We performed nonlinear dynamical systems analysis to obtain a comprehensive view on the role of various voltage-gated ion channels and external current in the onset of hyperexcitability. Keeping external current as a bifurcation parameter, we found a subcritical Hopf and cyclic limit point bifurcations separating the steady state and full-blown action potential oscillations respectively, and several period-doubling and limit point bifurcations describing the mixed-mode oscillations in between. Specific ion channels’ conductance and gating kinetics were most significant in inducing hyperexcitability since they were highly sensitive to the Hopf and limit point bifurcations. Hence, selectively targeting these ion channels can be a potential strategy to reverse hyperexcitability and subsequently provide relief from CIPN. This study outlines a promising approach to understand CIPN mechanism and explore therapeutic agents to prevent it. Further collaborative effort among clinicians, experimentalists, and mathematicians can lead to determining a robust solution to this long-standing problem.

**References**

1. Aromolaran KA, Goldstein PA. Ion channels and neuronal hyperexcitability in chemotherapy-induced peripheral neuropathy: Cause and effect? *Molecular Pain* 2017, 13, 1744806917714693.
2. Chung JM, Chung K. Importance of hyperexcitability of DRG neurons in neuropathic pain. *Pain Practice* 2002, 2.2, 87–97.

## P257 Estimating the readily-releasable vesicle pool size at synaptic connections in neocortex

### Natali Barros Zulaica^1^, Giuseppe Chindemi^1^, Rodrigo Perin^2^, Henry Markram^1^, Srikanth Ramaswamy^1^, Eilif Muller^1^, John Ramon^1^

#### ^1^École Polytechnique Fédérale de Lausanne, Blue Brain Project, Lausanne, Switzerland; ^2^École Polytechnique Fédérale de Lausanne, Laboratory of Neural Microcircuitry, Lausanne, Switzerland

##### **Correspondence:** Natali Barros Zulaica (natali.barroszulaica@epfl.ch)

*BMC Neuroscience* 2019, **20(Suppl 1)**: P257

Previous studies based on the ‘Quantal Model’ for synaptic transmission suggested that neurotransmitter release is mediated by a single release site at individual synaptic contacts in the neocortex. However, recent studies seem to contradict this hypothesis and indicate that multi-vesicular release (MVR) could better explain the synaptic response variability observed *in vitro*.

In this study we present a novel method to estimate the number of release sites per synapse, also known as the size of the readily-releasable pool (NRRP), from paired whole-cell recordings of layer 5 thick tufted pyramidal cell (L5-TTPC) connections in the somatosensory neocortex. Our approach extends the work of Loebel and colleagues to take advantage of a recently reported data-driven biophysical model of neocortical tissue .

From a collection of 33 paired whole cell patch-clamp recordings of L5-TTPC in the P14 rat somatosensory cortex, we extracted the synaptic parameters for the Tsodyks-Markram model of synapse dynamics (TM-model)and estimated the maximal synaptic conductance through matching experimental EPSPs values with equivalently sampled pairs simulated in the neocortical tissue model *in silico*. Finally, the size of the readily releasable pool, NRRP, was adjusted to obtain the best match between the coefficient of variation (CV) profile of the EPSPs for the *in vitro* data and *in silico* simulations. Using this approach, we estimated NRRPto be between two to three for connections between layer 5 thick tufted pyramidal cells. To constrain NRRPvalues for other connections in the microcircuit, we developed and validated a generalization approach using data on EPSP CVs from literature and matching to *in silico* experiments.

Our study shows that synaptic connections in the neocortex generally are mediated by MVR and provides a data-driven approach to constrain the MVR model parameters of the microcircuit. These findings have important implications for synaptic transmission, biophysical models of synaptic plasticity, and when considering synaptic noise sources for information processing in the neocortex.

## P258 Extracellular synaptic and action potential signatures in the hippocampal formation : a modelling study

### Amelie Aussel^1,2^, Harry Tran^1^, Laure Buhry^2^, Steven Le Cam^1^, Louis Maillard^1,3^, Sophie Colnat-Coulbois^1,4^, Valérie Louis Dorr^2^, Radu Ranta^1^

#### ^1^Université de Lorraine, CRAN UMR 7039, Nancy, France; ^2^Université de Lorraine, LORIA UMR CNRS 7503, Nancy, France; ^3^Université de Lorraine, CHRU Nancy Service de Neurologie, Nancy, France; ^4^Université de Lorraine, CHRU Nancy Service de Neurochirurgie, Nancy, France

##### **Correspondence:** Amelie Aussel (amelie.aussel@loria.fr)

*BMC Neuroscience* 2019, **20(Suppl 1)**: P258

Simulating extracellular recordings of neuronal populations is a challenging task for understanding the nature of extracellular field potentials (LFPs), investigating specific brain structures and mapping cognitive functions. It is well known that recording devices like microelectrodes record a mixture of high-frequency components reflecting action potentials (APs) activity and low frequency patterns, mainly attributed to the synaptic currents. Simulating such is a key step to understand the brain functioning, however it often requires a high computational burden due to the multicompartmental neuron models used [1]. This could be an issue for producing and validating computational models of neural activity of large populations such as hippocampal oscillations [3]. Here, we propose a method to reproduce brain oscillations by taking into account the APs and the synaptic currents contributions.

A model of the hippocampal structure was previously proposed to reproduce various oscillatory rhythms within the sleep–wake cycle and neurons were positioned and connected in an anatomically realistic manner [4]. Based on [5], we were able to set the neurons morphologies and the axon orientations for each hippocampus region in accordance with the literature. The model dynamic was modelled as a Poisson process with variable intensity (firing rate). This firing rate was extracted from the envelopes of real depth EEG recordings from cerebral areas projecting onto the Entorhinal cortex of human epileptic patients (recorded during presurgical evaluation in the Neurology Service of the Nancy University Hospital - CHRU Nancy, France). Contributions of the APs and the synaptic currents to the LFP were balanced by a pair of coefficients.

First of all, it was found that the frequency properties of the simulated signal were consistent with the ones of the real recorded signals. It was also found that the APs contribution was a key parameter for gamma band however it is less significant for lower frequency bands.

## P259 Neural architecture for representing sound in cortex

### Alex Reyes

#### New York University, Center for Neural Science, New York, NY, United States of America

##### **Correspondence:** Alex Reyes (ar65@nyu.edu)

*BMC Neuroscience* 2019, **20(Suppl 1)**: P259

Most sensory systems are organized topographically so that features of the sensory input are represented in an orderly fashion in the brain. How topography can be used to encode sensory information requires a firm understanding of how sensory features are mapped onto neural elements. A stumbling block is that the fundamental processing unit of cortex is ill-defined. Proposed motifs have included single neurons, canonical microcircuits, and large columns. Early experiments suggested that somatosensory and visual cortices are divided into columns, which consists of neurons with similar tuning properties. Over the years, the definition of columns has evolved to include micro- and hypercolumns and the requirement that the borders be sharp was relaxed. Moreover, other motifs emerged such as canonical microcircuits and ensembles. The dimensions and organization of proposed units have important theoretical implications as each impose limits on the computations that networks may perform.

To explore how these abstract notions of cortical organization affect function, the representation of sound in auditory cortex is examined. A salient feature of auditory cortex is that the characteristics frequencies of neurons vary systematically along one axis. This tonotopic organization, which originates in the cochlea and preserved via precise topographic afferent projections between stages of the auditory pathway, is the substrate for a ‘place code’ for representing tone frequency. Despite conceptual problems, the importance of this tonotopy-based coding scheme is undeniable: cochlear implants, the most successful brain-machine interface, enable the deaf to discriminate pitch simply by stimulating different points of the cochlea with brief electrical pulses. To gain insights into the functional organization of cortex that underlie sensory representation, a mapping from the acoustic space to neural space is constructed, adhering strictly to the constraints imposed by mathematics and the biology. The analyses indicate that the functional unit of cortex for a place code is unlikely to be a cortical column. Rather, the cortex is organized as overlapping clusters of neurons with flexible borders: tone frequency is represented by the location of the active clusters along the tonotopic axis and intensity by the cluster size.

## P260 Firing rate of neurons with dendrites, soma and axon in the fluctuation-driven, low-rate limit

### Robert Gowers^1^, Yulia Timofeeva^2^, Magnus Richardson^3^

#### ^1^University of Warwick, Centre for Doctoral Training in Mathematics for Real-World Systems, Coventry, United Kingdom; ^2^University of Warwick, Department of Computer Science, Coventry, United Kingdom; ^3^University of Warwick, Warwick Mathematics Institute, Coventry, United Kingdom

##### **Correspondence:** Robert Gowers (r.gowers@warwick.ac.uk)

*BMC Neuroscience* 2019, **20(Suppl 1)**: P260

Neurons *in-vivo* are subject to an input where the arrival time and strength of synaptic pulses is highly variable. The integration of this stochastic synaptic drive has been modelled extensively from the late 1960s. Since then, significant analytical progress has been made in the understanding of how class-specific membrane properties and synaptic dynamics affect neuronal integration. Due to the constraints of tractability, the majority of analytical work has been directed at neurons that can be approximated as electrotonically compact or as comprising a small number of connected, discrete compartments.

Here we consider continuous models of neurons comprising dendrites, soma and axon driven by spatially distributed fluctuating synaptic drive. We demonstrate that in the fluctuation-driven, low firing-rate limit a level-crossing approach can be used to approximate the steady-state firing rate. The low-rate limit in which Rice’s level-crossing becomes accurate is applicable to pyramidal neurons in which average firing rates have been measured to be low, when compared to the reciprocal of the effective membrane time constant.

We apply this approach to some very simple neuronal morphologies that can be considered as “toy models” of spatiotemporal synaptic integration. First, we demonstrate the interesting result that certain dendritic morphologies have firing-rate functions of the input drive that are independent of the electrotonic length but, nevertheless, distinct to that seen in point-like integrate-and-fire models. Second, when an axon is added, we demonstrate that the firing rate varies non-monotonically with the axonal radius, with the peak firing rate corresponding to an axonal radius similar to that found in pyramidal cells. Third, we observe that adding dendrites driven by fluctuating drive does not always increase the firing rate of the neuron. This last effect can also be captured quantitatively by discrete, compartment-based models; however, the advantage of the continuous approach is that the spatial variables are approximated in controlled fashion. Finally, we show that soma size and moving the position of the spike-initiation site on the axon alter both these non-monotonic relationships, with a larger soma causing the firing rate to be maximised for a higher number of dendrites.

Though applied to toy models of neuronal structure, these initial results show that it is possible to obtain analytical results, at least in the low-rate limit, that capture the effects of spatially distributed synaptic drive. This framework can also be straightforwardly extended to quasi-active neuronal membranes to include the effects of the h-current present in apical dendrites of pyramidal cells and thus provides a basis for future studies that include greater biophysical detail.

## P261 Noise can counterintuitively synchronize dynamics on the human connectome

### James Pang^1^, Leonardo L Gollo^2^, James Roberts^1^

#### ^1^QIMR Berghofer Medical Research Institute, Brain Modelling Group, Brisbane, Australia; ^2^QIMR Berghofer Medical Research Institute, Brisbane, Australia

##### **Correspondence:** James Pang (james.pang@qimrberghofer.edu.au)

*BMC Neuroscience* 2019, **20(Suppl 1)**: P261

Cortical coherence is important for the communication of functionally specific brain regions to establish transient networks that accomplish cognitive function [1]. Meanwhile, stochastic effects have been shown to induce optimal system responses, termed stochastic resonance (SR) [2, 3]. Thus, we ask whether stochastic inputs can produce an SR-like coherent dynamics on the human connectome. We implement a large-scale model of noisy Kuramoto oscillators on a human connectome with 513 interconnected nodes. Each oscillator has a local phase and a natural frequency and is driven by a Gaussian noise. Based on findings that a hierarchy of timescales exists across the primate cortex [4], we take the natural frequency from a hierarchical distribution within the bandwidth of fMRI [5] (Fig. 1b inset). This allows the cortical hierarchy to be mapped into a gradient of timescales with slow hubs and fast peripheral regions. We use the order parameter *R* to quantify global phase coherence (*R* = 0 for incoherent state and *R* = 1 for coherent state). We also calculate the network synchronization from the time average of *R* after discarding transient dynamics.

**Fig. 1:**
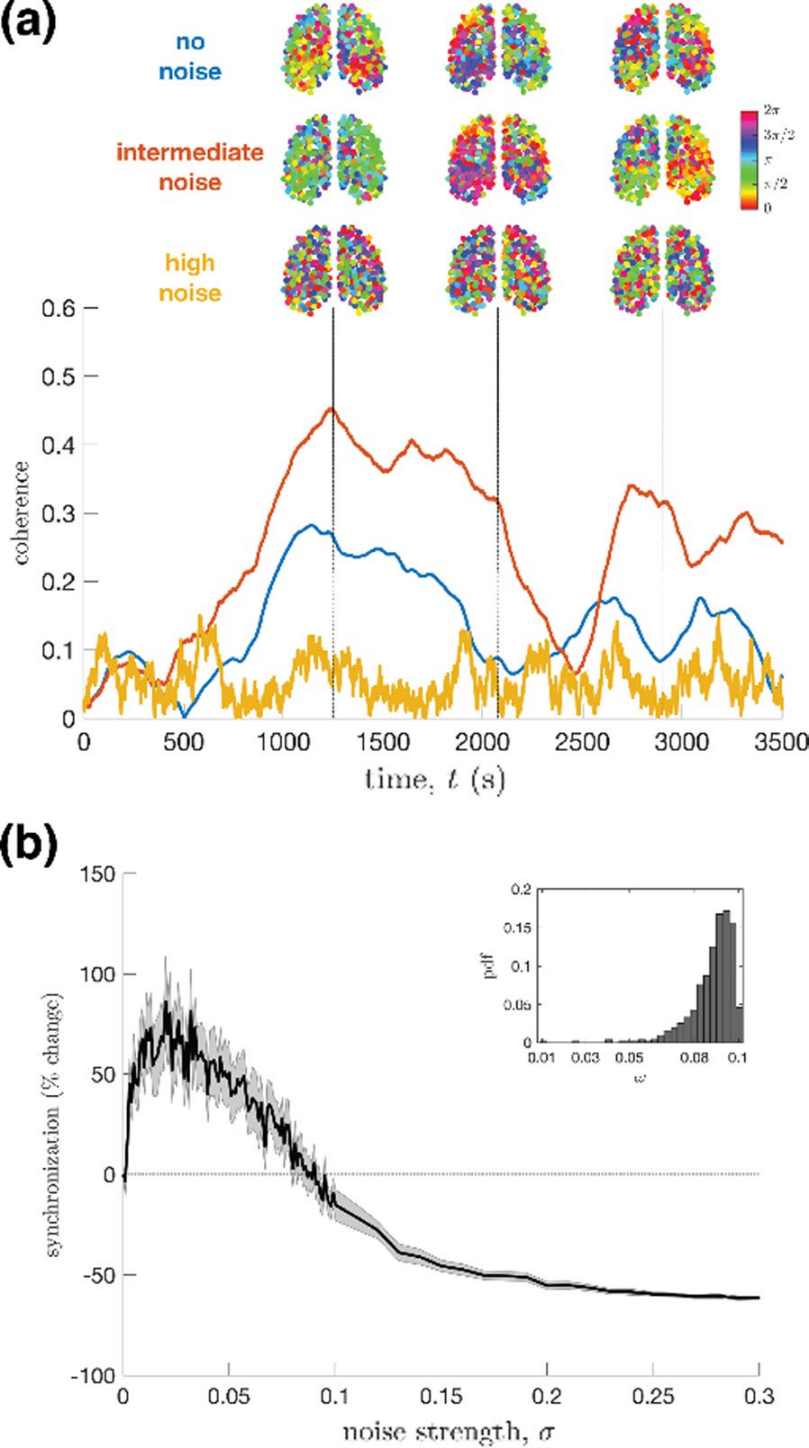
**a** Top: Node dynamics for cases with no noise, with intermediate noise, and with high noise. Bottom: Coherence for the three noise cases. **b** Percent change of synchronization as a function of noise strength. The shaded region is the SEM. The inset shows the hierarchical distribution. The results clearly demonstrate the counterintuitive SR effect

Fig. 1a shows that coherence paradoxically improves for an intermediate noise level but breaks down for a higher level. This is reminiscent of SR, which is also found in experiments on human perception and cognition [6] and on metastable transitions of brain waves [2]. Fig. 1b further demonstrates SR where an intermediate noise level optimizes synchronization. We also found that SR disappears by destroying the structure of the connectome (e.g., random connectivity) and/or choosing other frequency distributions (e.g., random distribution). This proves that the hierarchical structure of the human connectome and the hierarchy of time scales are both key ingredients to produce and drive the paradoxical SR effect.

The significance of this work can be summarized as follows: (i) a counterintuitive effect is found where addition of disorder leads to a more ordered state; (ii) the formulation is general because the Kuramoto model applies to many neural and non-neural systems with coupled oscillatory units; (iii) the results emphasize the role of hierarchy and heterogeneity in brain dynamics, which is a topic of current interest in neuroscience; and (iv) the results disentangle the contributions of the network and local node dynamics to the emergence of large-scale dynamics.

**References**

1. Ward LM. Synchronous neural oscillations and cognitive processes. *Trends in cognitive sciences* 2003 Dec 1;7(12):553–9.
2. Roberts JA, Gollo LL, Abeysuriya RG, et al. Metastable brain waves. *Nature communications* 2019 Mar 5;10(1):1056.
3. Collins JJ, Chow CC, Imhoff TT. Stochastic resonance without tuning. *Nature* 1995 Jul;376(6537):236.
4. Murray JD, Bernacchia A, Freedman DJ, et al. A hierarchy of intrinsic timescales across primate cortex. *Nature neuroscience* 2014 Dec;17(12):1661.
5. Cocchi L, Sale MV, Gollo LL, et al. A hierarchy of timescales explains distinct effects of local inhibition of primary visual cortex and frontal eye fields. *Elife* 2016 Sep 6;5:e15252.
6. Landry N, Ward BJ, Trépanier S, et al. Preclinical and clinical development of plant-made virus-like particle vaccine against avian H5N1 influenza. *PloS one* 2010 Dec 22;5(12):e15559.

## P262 Neural heterogeneity in the gain control mechanism of antennal lobe improves odorant classification

### Aaron Montero, Jessica Lopez-Hazas, Francisco B Rodriguez

#### Universidad Autónoma Madrid, Ingeniería Informática, Madrid, Spain

##### **Correspondence:** Aaron Montero (aaron.montero.m@gmail.com)

*BMC Neuroscience* 2019, **20(Suppl 1)**: P262

The antennal lobe (AL) of insects receives the stimuli from the olfactory receptor neurons (ORNs), grouping them in glomeruli related to the different ORNs. Once the odor information is collected by the glomeruli, it is sent to the mushroom body (MB) by the projection neurons (PNs) located in AL. However, despite the fact that odor concentration affects the ORNs, the signal transmitted by the PNs is invariant to it [1]. This is due to the gain control mechanism realized by the local neurons (LNs) of AL that control the activity of PNs by inhibiting them. This gain control mechanism may be due to the heterogeneity [2] found in the LNs since two types of neurons are observed: homoLNs, which innervate most if not all glomeruli uniformly, and heteroLNs, which innervate only a few of them [3]. The aim of this study is to simulate this gain control mechanism and analyze its ability to modulate the stimulus signal compared to normalization.

To implement the gain control mechanism, we took into account that the proportion of the LNs is approximately one third of the PNs (830 PNs and 300 LNs in locust, 150-200 PNs among the 250 AL neurons in Drosophila) and made the following assumptions: (i) all PNs are uniglomerular (as it happens in the majority of PNs of the Drosophila), (ii) homoLNs are connected to all the glomeruli and use the activity received by one of them to inhibit the rest and (iii) heteroLNs are connected to a few glomeruli and use their average activity to inhibit them.

This gain control is introduced in the input layer of our computational model that simulates the main parts of insect olfactory system [4, 5]. In this model, the input corresponds to AL, the hidden layer to the Kenyon cells and the output to the MB output neurons, where odor stimuli are classified by a Hebbian learning between the connections of these last two layers.

In order to simulate the odor patterns for different concentration levels, we used Gaussian distributions with variable heights and widths, which centers encode the odor identity. The concentration variability of these patterns generates in our gain control mechanism an input-output relationship similar to the one observed in AL. Furthermore, the classification error using our heterogeneous gain control mechanism had an improvement of 2.4% compared to a homogeneous gain control such as normalization of patterns. These preliminary results give a possible computational explanation of why neural heterogeneity can perform a suitable gain control mechanism in the insect olfactory system.

**Acknowledgements**: This research was supported by the Spanish Government project TIN2017-84452-R.

**References**

1. Stopfer M, Jayaraman V, Laurent G. Intensity versus identity coding in an olfactory system. *Neuron* 2003, 39:991–1004
2. Mejias JF, Longtin A. Differential effects of excitatory and inhibitory heterogeneity on the gain and asynchronous state of sparse cortical networks. *Frontiers in Computational Neuroscience* 2014, 8:107
3. Girardin CC, Kreissl S, Galizia CG. Inhibitory connections in the honeybee antennal lobe are spatially patchy. *Journal of Neurophysiology* 2013, 109:332–343
4. Montero A, Huerta R, Rodriguez FB. Regulation of specialists and generalists by neural variability improves pattern recognition performance. *Neurocomputing* 2015, 151:69–77
5. Montero A, Huerta R, Rodriguez FB. Stimulus space complexity determines the ratio of specialist and generalist neurons during pattern recognition. *Journal of the Franklin Institute* 2018, 355:2951–2977

## P263 Tuning a computational model of the electromotor system to patterns of interpulse intervals recorded from Gnathonemus petersii specimens

### Angel Lareo^1^, Pablo Varona^2^, Francisco B Rodriguez^2^

#### ^1^Universidad Autónoma de Madrid, Grupo de Neurocomputación Biológica, Madrid, Spain; ^2^Universidad Autónoma de Madrid, Ingeniería Informática, Madrid, Spain

##### **Correspondence:** Angel Lareo (angel.lareo@uam.es)

*BMC Neuroscience* 2019, **20(Suppl 1)**: P263

The electromotor system of pulse mormyrids, a family of weakly electric fish, is a neural network which encodes information using a multiplexed temporal coding scheme [1]. The output of the system is correlated with the generation of electrical discharges by the electric organ of these fish. The electrical activity can be recorded non-invasively during long-time periods by monitoring a freely-behaving specimen using an appropriate registration setup [2]. Recordings of such electrical activity typically show a large temporal variability. In this context, a set of stereotyped temporal patterns (which are related to overall fish behavior) has been described by previous neurothological studies [3]. Furthermore, the topology of the network has been described by several physiological studies [4]. These characteristics make this neural network an interesting case for studying information encoding and processing in the nervous system.

A model of the electromotor system which replicates temporal firing patterns described by previous studies was developed using genetic algorithms (GA) for multi-objective parameter optimization [5]. The model was tuned to reproduce a set of artificially-constructed stereotyped temporal firing patterns. Patterns of the electrical interpulse intervals detected from real recordings of several Gnathonemus Petersii specimens were used as target patterns to readjust the parameters of the model. Accordingly, several changes in the GA evaluation function were introduced to consider the larger variability of pattern examples observed in living fish recordings.

Output results from the model showed a proper adjustment to the temporal structure of the real patterns. Nevertheless, automated parameter optimization techniques result in disparate parameter configurations which almost equally adjust to experimental data. The robustness of each GA-adjusted configuration was evaluated in this study. In particular, the reproducibility of the patterns was tested by adding disturbances to the model inputs in terms of intensity and duration.

**Acknowledgments:** This work has been supported by Spanish grants MINECO (http://www.mineco.gob.es/) TIN2017-444 84452-R, DPI2015-65833-P.

**References**

1. Baker CA, Kohashi T, Lyons-Warren AM, Ma X, Carlson BA. Multiplexed temporal coding of electric communication signals in mormyrid fishes. *Journal of Experimental Biology* 2013 Jul 1;216(13):2365–79.
2. Lareo A, Forlim CG, Pinto RD, Varona P, Rodriguez FD. Temporal code-driven stimulation: definition and application to electric fish signaling. *Frontiers in Neuroinformatics* 2016 Oct 6;10:41.
3. Carlson BA, Hopkins CD. Stereotyped temporal patterns in electrical communication. *Animal Behaviour* 2004 Oct 1;68(4):867–78.
4. Carlson BA. Neuroanatomy of the mormyrid electromotor control system. *Journal of Comparative Neurology* 2002 Dec 23;454(4):440–55.
5. Lareo A, Varona P, Rodríguez FB. Evolutionary Tuning of a Pulse Mormyrid Electromotor Model to Generate Stereotyped Sequences of Electrical Pulse Intervals. *In International Conference on Artificial Neural Networks* 2018 Oct 4 (pp. 359–368). Springer, Cham.

## P264 Kenyon Cells threshold distribution adapts to pattern complexity in a bioinspired computational learning model of the locust olfactory system

### Jessica Lopez-Hazas, Aaron Montero, Francisco B Rodriguez

#### Universidad Autónoma Madrid, Ingeniería Informática, Madrid, Spain

##### **Correspondence:** Aaron Montero (aaron.montero.m@gmail.com)

*BMC Neuroscience* 2019, **20(Suppl 1)**: P264

A mechanism that controls the generation of neural spikes and, therefore, how information is encoded in biological systems is the neural threshold. Some studies have pointed out that neural threshold dynamics could cause certain emerging properties, like the regulation of the sensitivity of neurons [1]. Previous research on the locust olfactory system showed that Kenyon Cells (KCs) in the mushroom body use sparse coding to represent the odorants that arrive at the system [4] and that there is variability in the sensitivity of these neurons towards the input they receive [2].

To study the implications of this hypothesis, we have used the locust olfactory system model presented in [3], which includes a learning algorithm capable of finding the best neural threshold distribution for KCs in order to resolve a classification problem. The model also imitates the effects of the inhibition KCs receive from the giant GABAergic neuron [5] through an activity regulation term. We have examined the threshold distributions found by the algorithm for Gaussian patterns of two different complexity levels, according to the measure developed in [1]: (i) Gaussian patterns with moderate noise (easier to classify) and (ii) Gaussian patterns with high noise (more difficult to classify).

We have found that the distributions adjusted by the learning algorithm are always heterogeneous and that there is a relationship between the complexity of the patterns and the ratio between generalist neurons (lower thresholds) and specialist ones (higher thresholds). When the classification problem is easy, the solution found by the learning algorithm contains a majority of generalist neurons with lower thresholds (3.33 ± 1.95 arbitrary units in the model), while, when complexity is high, specialist neurons with higher thresholds (9.52 ± 2.84) predominate after the learning process.

A plausible explanation to this behavior is that, as complexity increases, using sparse coding to separate the patterns of different classes is more advantageous. This is why a greater amount of specialist neurons that only respond to certain inputs are preferred. This is also consistent with previous studies where neural thresholds were estimated by a brute-force search algorithm [1]. The results obtained show the important influence of neural threshold in order to regulate neural sensitivity and provide energetic efficient ways of encoding information like sparse coding, which could be potentially applied to artificial neural networks.

**Acknowledgments:** This research was supported by the Spanish Government projects TIN2017-84452-R.

**References**

1. Montero A, Huerta R, Rodriguez FB. Stimulus space complexity determines the ratio of specialist and generalist neurons during pattern recognition. *Journal of the Franklin Institute* 2018;355:2951–2977.
2. Rodriguez FB, Huerta R. Techniques for temporal detection of neural sensitivity to external stimulation. *Biological Cybernetics* 2009;100:289–297.
3. Lopez-Hazas J, Montero A, Rodriguez FB. Strategies to enhance pattern recognition in neural networks based on the insect olfactory system. *Lecture Notes in Computer Science* 2018;11139 LNCS:468–475.
4. Perez-Orive J, Mazor O, Turner GC, Cassenaer S, Wilson RI, Laurent G. Oscillations and sparsening of odor representations in the mushroom body. *Science* 2002;297:359–65.
5. Gupta N, Stopfer M. Olfactory coding: Giant inhibitory neuron governs sparse odor codes. *Current Biology* 2011;21:R504–R506.

## P265 Biologically realistic mean-field models of conductance-based networks of spiking neurons

### Matteo di Volo^1^, Cristiano Capone^2^, Alain Destexhe^3^, Alberto Romagnoni^4^

#### ^1^CNRS, Paris, France; ^2^INFN, sezione di Roma, INFN, sezione di Roma, Rome, Italy; ^3^CNRS, Department of Integrative and Computational Neuroscience (ICN), Gif-sur-Yvette, France; ^4^Ecole normale superieure ENS, Departement d’informatique, Paris, France

##### **Correspondence:** Matteo di Volo (m.divolo@gmail.com)

*BMC Neuroscience* 2019, **20(Suppl 1)**: P265

Accurate population models are needed to build very large-scale neural models. Nevertheless, their derivation is difficult for realistic networks of neurons, in particular when nonlinear properties are involved such as conductance-based interactions and spike-frequency adaptation. Here, we consider such models based on networks of Adaptive Exponential Integrate and Fire excitatory and inhibitory neurons. Using a Master Equation formalism, we derive a mean-field model of such networks and compare it to the full network dynamics. The mean-field model is capable to correctly predict the average spontaneous activity levels in asynchronous irregular regimes similar to in vivo activity. It also captures the transient temporal response of the network to complex external inputs. Finally, the mean-field model is also able to quantitatively describe regimes where high and low activity states alternate (UP-DOWN state dynamics), leading to slow oscillations. We conclude that such mean-field models are “biologically realistic” in the sense that they can capture both spontaneous and evoked activity, and they naturally appear as candidates to build very large-scale models involving multiple brain areas.

## P266 Interplay between network structure and synaptic strength on information transmission in hierarchical modular cortical networks

### Rodrigo Pena^1^, Vinicius Lima^2^, Renan O. Shimoura^2^, Joao-Paulo Novato^2^, Antônio C. Roque^2^

#### ^1^New Jersey Institute of Technology, Federated Department of Biological Sciences, Newark, NJ, United States of America; ^2^University of Sao Paulo, Dept. Physics, FFCLRP, Ribeirao Preto, Brazil

##### **Correspondence:** Antônio C. Roque (antonior@usp.br)

*BMC Neuroscience* 2019, **20(Suppl 1)**: P266

The cerebral cortex is organized in a hierarchical modular (HM) way, from cellular microcircuits at the lowest level to cortical areas at the intermediate level to brain regions at the highest level. The effect of this complex structure on activity propagation across its multiple spatial scales is still not well understood. Here, we use simple HM spiking network models to study information transmission (IT) in these networks as a function of two parameters, hierarchical level H of the network, which determines the number of modules, and overall strength J of synaptic coupling, and show that there is an optimal range of H values which maximizes IT. We start with a random network of N = 217 neurons connected with probability 0.01. The ratio of excitatory to inhibitory neurons is 4:1. Neurons are described by the LIF model with parameters as in [1]. The arrival of a synaptic input causes an instantaneous postsynaptic voltage increment J (excitatory synapse) or −gJ (inhibitory synapse). We fix g = 5, so synaptic strength is parameterized by J (in the range (0,1]). We create HM networks from the random network using the same scheme as in [2], so networks with hierarchical level H have 2H modules. We considered networks with H in the range from 1 (2 modules) to 9 (512 modules). We simulated networks generated by all combinations of parameters (H,J) in their respective ranges for simulation times of 2 sec. We quantified IT in the networks by the mean transfer entropy (TE) between firing rates of adjacent modules [3]. Our simulations show that for each value of J there is always a single value of H that optimizes IT: TE increases with H up to a certain value of H, and then decreases as H increases further on. The H values that maximize IT are always in the range 5-7. So, for the networks considered by us the number of modules that maximize IT is neither too small nor too large. We observed that the increase of IT caused by the increase of H is associated to an increase in the cross-correlation of the spike trains of network neurons. So, we propose the following mechanism to explain why there is an optimal intermediate range of H values for IT in the network: while H is below the optimal range, each increase in H causes modules to fire in a more correlated way; but after H passes the optimal range, each further increase in H causes modules to behave more independently from each other, gradually decreasing inter-modular communication. We also observed that for networks with their H value set within the optimal range, the effect of varying the coupling strength J is to cause small changes in IT, demonstrating robustness with respect to J in this range. Our results suggest that there is an interplay between the topological structure of the cortical network and the synaptic strength level, which affects information transmission in the network.

**Acknowledgments:** Research supported by FAPESP (grants 2013/07699-0 CEPID NeuroMat and 2015/50122-0). VL and RS are supported by FAPESP scholarships (grants 2017/05874-0 and 2017/07688-9 respectively). ACR is partially supported by a CNPq fellowship (grant 306251/2014-0).

**References**

1. Ostojic S. Two types of asynchronous activity in networks of excitatory and inhibitory spiking neurons. *Nature neuroscience* 2014 Apr;17(4):594.
2. Tomov P, Pena RF, Zaks MA, Roque AC. Sustained oscillations, irregular firing, and chaotic dynamics in hierarchical modular networks with mixtures of electrophysiological cell types. *Frontiers in computational neuroscience* 2014 Sep 2;8:103.
3. Schreiber T. Measuring information transfer. *Physical review letters* 2000 Jul 10;85(2):461.

## P267 Wavenet identification and input estimation from single voltatge traces

### Toni Guillamon^1^, Carlos A. Claumann^2^, Enric Fossas^3^, Nestor Roqueiro^4^

#### ^1^Universitat Politecnica de Catalunya, Mathematics, Barcelona, Spain; ^2^Researcher, Florianopolis, Brazil; ^3^Universitat Politecnica de Catalunya, Automatic Control, Barcelona, Spain; ^4^Federal University of Santa Catarina, Automação e Sistemas, Florianopolis, Brazil

##### **Correspondence:** Toni Guillamon (antoni.guillamon@upc.edu)

*BMC Neuroscience* 2019, **20(Suppl 1)**: P267

We explore a new computational procedure devised both to obtain a heuristic neuron model and to estimate input parameters only from a single voltage trace. Our proposal is based on an efficient use of artificial neural networks based on wavelets (wavenet). On one side, given an appropriately designed input and the resulting voltage trace, we obtain a black-box model that reproduces neuron’s dynamics (“identification” process); it is intended for cases where electrophysiological exploration is discarded, but still one seeks for predictions on the neuron’s response under different stimuli or neighbouring activity. On the other side, we show how the wavenet methodology can also be applied to the reverse situation, that is, to provide input estimations from voltage traces (by means of an inverse network). We use this reverse procedure to estimate the synaptic input with the ultimate goal of estimating synaptic conductances, an active current research line with no complete solutions yet, see for instance [1].

The efficiency of the wavenet methodology comes from a modification in the representation of dynamical systems by wavenets which decreases the number of used functions, see [2]. This approach combines localized and global scope functions in a network with a specially chosen target function.

In this communication, we focus on a proof-of-concept of the whole methodology instantiated by conductance-based neuron models, although it is clearly extendable to experimental data. We first perform the identification from voltage traces obtained by simulation of the specific neuron models. We show that, after training our network with biologically plausible input currents, the network is able to identify the neuron’s behaviour with high accuracy. Interestingly, the input currents used for training the wavenet span both quiescent and spiking regimes (Fig. 1, left), thus showing the ability of identifying abrupt changes in the bifurcation diagram, from almost linear input-output relationships to highly nonlinear ones. These findings open new avenues to provide heuristic models for real neurons by stimulating them in closed-loop experiments, using for instance dynamic-clamp.

**Fig. 1:**
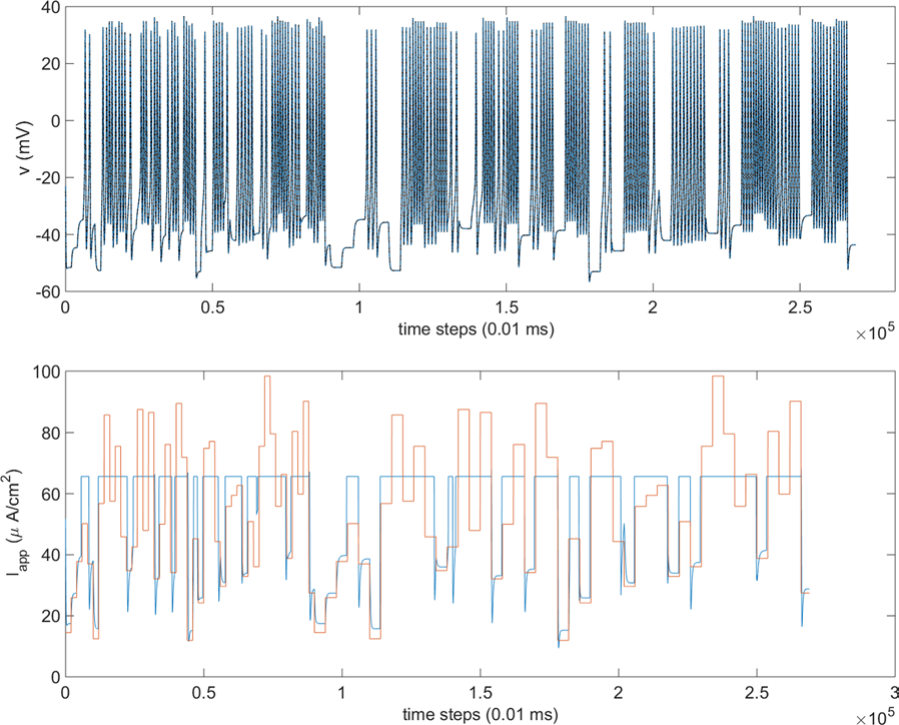
(Up) Solid line: the original current input; light dot-dashed line: estimation of current input (a mask, not explained in the abstract, has been applied). (Down) Voltages obtained from the direct wavenet mimicking a Morris-Lecar model. Solid line: voltatge elicited by the original current input; light dot-dashed line: voltatge reconstruction using the estimated current trace. Time step is 0.01 ms

Concerning the estimation of synaptic inputs, we train the inverse wavenet with voltage traces (obtained from direct wavenet) and their corresponding inputs. Given a voltage trace, the output of the inverse wavenet turns out to be the estimation of the input current received by the cell. In (Fig. 1), we show both estimations of the input for the Morris-Lecar model and the corresponding voltage reconstruction whose high accuracy validates the test. Note that from the synaptic input we can further estimate the synaptic conductances. Remarkably, our method overcomes typical shortcomings that are persistent in the literature on estimation of synaptic conductances: it works with a single trial and resists the presence of nonlinear currents (spiking or specific subthreshold currents). Apart from single experimental data, the proposed method can also be used for rate models and population data.

**Acknowledgements**: We thank Spanish grant MINECO-FEDER-UE MTM-2015-71509-C2-2-R (AG) and Catalan Grant 2017SGR1049 (AG) 2017SGR0872 (EF). This work has been done in a long-term stay of Prof. Roqueiro at the UPC.

**References**

1. Chizhov AV, Amakhin DV. Method of experimental synaptic conductance estimation: Limitations of the basic approach and extension to voltage-dependent conductances. Neurocomputing. 2018 Jan 31;275:2414–25.
2. Claumann CA. Desenvolvimento e aplicações de redes neurais wavelets e da teoria de regularização na modelagem de processos. *PhD Thesis* 2003. https://repositorio.ufsc.br/handle/123456789/86310

## P268 A biophysical model for the tripartite synapse under metabolic stress

### Manu Kalia^1^, Stephan van Gils^1^, Michel J.A.M. van Putten^2^, Christine R. Rose^3^

#### ^1^University of Twente, Applied Mathematics, Enschede, Netherlands; ^2^University of Twente, Department of Clinical Neurophysiology, Enschede, Netherlands; ^3^Heinrich Heine University Duesseldorf, Institute of Neurobiology, Duesseldorf, Germany

##### **Correspondence:** Manu Kalia (m.kalia@utwente.nl)

*BMC Neuroscience* 2019, **20(Suppl 1)**: P268

At the tripartite synapse astrocytes are increasingly recognized as major modulators of synaptic transmission. However, there are significant gaps in our understanding of the early changes in neuronal and astrocyte function during reduced energy availability. In this work we introduce a detailed single-cell biophysical model of the glutamatergic tripartite synapse to study dynamics of five relevant ions during metabolic stress. We calibrated the model with recent experimental findings on early events of metabolic failure. The model explains previously unclear mechanisms of early synaptic failure and cellular swelling during low energy conditions, as a function of ion clearance pathways by the astrocyte and extracellular space. We show that smaller extracellular spaces correspond to higher vulnerability to metabolic stress. Using sensitivity and bifurcation analysis, we explain the transitions between physiological and pathological states, parameterized by initial extracellular ratio and maximum ATPase activity. We also discuss key mechanisms associated with recovery from a pathological state upon restoring physiological conditions, thereby providing directions for future experiments.

**Acknowledgements**: Supported by the German Research Foundation (DFG), Research Unit 2795 ‘Synapses under stress’ (Ro2327/14-1 to CR Rose and Mercator fellowhips to SA van Gils and Michel JAM van Putten).

## P269 Fluctuation-driven plasticity allows for flexible rewiring of neuronal assemblies

### Federico Devalle^1^, Ernest Montbrió^1^, Alex Roxin^2^

#### ^1^Universitat Pompeu Fabra, Department of Information and Communication Technologies, Barcelona, Spain; ^2^Centre de Recerca Matemàtica, Barcelona, Spain

##### **Correspondence:** Alex Roxin (aroxin@crm.cat)

*BMC Neuroscience* 2019, **20(Suppl 1)**: P269

Cortical circuitry is shaped through ongoing synaptic plasticity. However, network models in which recurrent synaptic connections change via Hebbian plasticity rules are unstable: synapses become maximally potentiated or depressed, effectively erasing all nontrivial structure in the connectivity. One solution to this dilemma is to include additional mechanisms to offset Hebbian instabilities. Here we consider an alternative scenario in which, given constant firing rates, the rates of potentiation and depression are equal and opposite. Net potentiation or depression only occurs when the firing rates of neurons covary in time. We show that standard heuristic STDP (spike-timing dependent plasticity) rules can have this property. Furthermore, we show how external time-varying signals can be used to flexibly control the network structure. As an example, neuronal assemblies can be strongly coupled, decoupled, or uni-directionally coupled by driving them with oscillatory signals with distinct phase lags. Alternatively, the connectivity between assemblies driven by stochastic inputs can be flexibly shaped via the covariance matrix of the inputs.

Recent work [1] shows that model networks with hierarchically organized clusters can fit all relevant connectivity statistics reported in slice experiments from rat cortex [2]. Our work here suggests a mechanism to account for the formation of such a clustered network structure. Namely, when sensory stimuli drive a time-varying response in a network with heterogeneous feature selectivity, the recurrent connectivity will be shaped by the cross-correlation in the firing rates. Our analysis indicates that neurons with similar time-varying response (selectivity) will form strongly interconnected clusters, while the connectivity between any pair of clusters will depend on the cross-correlation and time-lag in the sensory drive that each receives. Importantly, this rewiring only occurs due to stimulus-driven fluctuations of the neuronal activity about the baseline rates; constant rates result in no overall plasticity.

**Acknowledgments:** Grant MTM2015-71509-C2-1-R of the Spanish Ministry of Economics and Competitiveness.

FLAG-ERA Grant HIPPOPLAST of the HBP and Proyecto I+i+D Programación Conjunta Internacional PCI2018-093095.

This work was partially funded by the CERCA network of centers of the Generalitat de Catalunya.

**References**

1. Vegue M, Perin R, Roxin A. On the structure of cortical microcircuits inferred from small sample sizes. *Journal of Neuroscience* 2017 Aug 30;37(35):8498–510.
2. Perin R, Berger TK, Markram H. A synaptic organizing principle for cortical neuronal groups. *Proceedings of the National Academy of Sciences* 2011 Mar 29;108(13):5419–24.

## P270 Exploring mechanisms of intermittent patterns of neural synchrony

### Joel Zirkle^1^, Leonid Rubchinsky^1,2^

#### ^1^Indiana University Purdue University Indianapolis, Department of Mathematical Sciences, Indianapolis, IN, United States of America; ^2^Indiana University School of Medicine, Stark Neurosciences Research Institute, Indianapolis, IN, United States of America

##### **Correspondence:** Leonid Rubchinsky (lrubchin@iupui.edu)

*BMC Neuroscience* 2019, **20(Suppl 1)**: P270

Synchrony of neural oscillations is believed to play important role in a variety of brain functions. Too excessive or too weak synchrony is associated with several neurological and neuropsychiatric dysfunctions. However, the synchrony in cortical and subcortical circuits may not necessarily stay perfect for a prolong intervals of time. This implies that for some intervals of time synchrony may be stronger, while for other intervals of time it may be weaker. Few long intervals of desynchronized dynamics may be functionally different from many short desynchronized intervals, although the synchrony may be the same on the average [1].

Recent developments in time-series analysis allowed exploring the temporal patterning of synchrony on very short time-scales [2]. These methods were recently used to study neural synchronization in several different systems and signal types (humans and rodents, spikes, LFP, and EEG). All these studies had one common feature: the observed synchrony level was reached by potentially very frequent, but short desynchronizations [3, 4].

This study explores the effect of synaptic plasticity and channel noise on the temporal patterning of intermittent synchronization on short temporal scales. Small networks of a conductance-based models (of a Morris-Lecar type) are used similarly to a prior study [5], the neurons are connected via excitatory synapses. STDP plasticity rule is implemented in the model to consider the plastic effects. Channel noise is introduced con consider how noise affects the patterns of intermittent synchrony. with implemented STDP rule. Presence of oscillatory activity is confirmed via SNR criterion.

Temporal patterning of synchronization was characterized by the distribution of desynchronization durations. Since some level of phase synchronization is present, there is a preferred value of the phase difference. Dynamics is considered to be desynchronized, when the actual phase difference deviates from the preferred phase difference by more than a pre-selected threshold value. This approach distinguishes between a large number of short desynchronizations, a small number of long desynchronizations, and the spectrum of possibilities in between even if they all yield the same average value of synchronization strength.

We found that both STDP plasticity and channel noise have a substantial potential in promoting short desynchronizations dynamics. These model-based observations fit with earlier experimental observations of the changes in the distribution of desynchronization durations.

**Acknowledgement**: This work was supported by NSF grant DMS 1813819.

**References**

1. Ahn S, Zauber SE, Worth RM, Witt T, Rubchinsky LL. Neural synchronization: average strength vs. temporal patterning. *Clinical Neurophysiology* 2018, 129, 842–844.
2. Ahn S, Park C, Rubchinsky LL. Detecting the temporal structure of intermittent phase locking. *Phys Rev E.* 2011, 84, 016201.
3. Ahn S, Rubchinsky LL. Short desynchronization episodes prevail in synchronous dynamics of human brain rhythms. *Chaos* 2013, 23, 013138.
4. Ratnadurai-Giridharan S, Zauber SE, Worth RM, Witt T, Ahn S, Rubchinsky LL. Temporal patterning of neural synchrony in the basal ganglia in Parkinson’s disease. *Clinical Neurophysiology* 2016, 127, 1743–1745.
5. Ahn S, Rubchinsky LL. Potential mechanisms and functions of intermittent neural synchronization. *Frontiers in Computational Neuroscience* 2017, 11, 44.

## P271 Substantia Nigra pars reticulata responses to direct and indirect pathway GABAergic projections depend on intracellular chloride dynamics

### Ryan Phillips, Jonathan Rubin

#### University of Pittsburgh, Department of Mathematics, Pittsburgh, PA, United States of America

##### **Correspondence:** Ryan Phillips (ryan.sean.phillips@gmail.com)

*BMC Neuroscience* 2019, **20(Suppl 1)**: P271

In the mammalian central nervous system, post-synaptic activation of ionotropic GABAA-receptors underlies a primary form of fast synaptic transmission. The resulting GABAA-current (IGABA), while typically considered inhibitory, may also be shunting or excitatory. Moreover, IGABA may exhibit biphasic inhibitory-to-excitatory responses mediated by intracellular chloride dynamics [1]. In this computational study, we show that detailed consideration of GABAergic dynamics may be critical for understanding neurocircuit function in brain regions that depend heavily on GABAA synaptic transmission.

We simulated GABAergic synaptic transmission in a model of the substantia nigra pars reticulata (SNr). The SNr is the primary output nucleus of the rodent basal ganglia (BG) and receives converging GABAA-receptor mediated synaptic inputs from the two major transmission channels through the BG, the direct and indirect pathways. Due to the convergence of these inputs, we predict that GABAergic signals to SNr will induce the range of atypical responses described above, depending on conditions and input properties. The details of these responses will be shaped by the firing rates and patterns of the presynaptic neurons and the short-term plasticity of the associated synapses. Moreover, direct pathway projections synapse on the distal dendrites whereas indirect pathway projections form basket-like synapses around the somas of SNr neurons [2]. The functional significance of these synapse locations is unclear; however, due to differences in compartment size and the distribution of the Cl extruder KCC2, dendritic and somatic compartments may have different susceptibilities to Cl accumulation and to breakdown of the GABAA reversal potential (EGABA) [1]. Distinct changes in EGABA due to these factors may lead to significant disparities in the properties of direct and indirect pathway synapses on SNr neurons.

To test the roles of EGABA and the Cl extruder KCC2 in shaping SNr responses to synaptic inputs, we constructed a novel conductance-based model of an SNr neuron that includes dendritic and somatic compartments. After showing that the model’s dynamics matches a range of experimental findings on SNr firing patterns, we used the model to study the effects of [Cl]i dynamics on EGABA and short-term ionic plasticity. We show that GABAA- and KCC2-mediated fluctuations in [Cl]i can explain many aspects of the SNr spiking responses to GABAergic inputs from the direct and indirect pathways. We also explore the predictions of our model relating to SNr activity patterns in functionally relevant settings involving inputs from both pathways. We provide a possible explanation for motor rescue in akinetic dopamine-depleted mice during optogenetic stimulation of indirect pathway subpopulations published by the Gittis lab. Integration of GABAA receptor-mediated synaptic inputs to somatic and dendritic compartments is not unique to SNr neurons; thus, these results may have implications for other brain regions as well.

**References**

1. Raimondo J, et al. Short-term ionic plasticity at GABAergic synapses. *Front Synaptic Neuroscience* 2012, 4: 5.
2. Von Krosigk M, et al. Synaptic organization of GABAergic inputs from the striatum and the globus pallidus onto neurons in the substantia nigra and retrorubral field which project to the medullary reticular formation. *Neuroscience* 1992, 50.3: 531–549.

## P272 Comparing spikes and the local field potential (LFP) in V1 between experimental data and a comprehensive biophysical model

### Espen Hagen^1^, Alexander J Stasik^1^, Yazan Billeh^2^, Joshua Siegle^2^, Kael Dai^2^, Torbjørn V Ness^3^, Marianne Fyhn^4^, Torkel Hafting^5^, Christof Koch^2^, Shawn Olsen^2^, Gaute Einevoll^3^, Anton Arkhipov^2^, Atle E. Rimehaug^6^, Malin B. Røe^4^

#### ^1^University of Oslo, Department of Physics, Oslo, Norway; ^2^Allen Institute for Brain Science, Modelling, Analysis and Theory, Seattle, WA, United States of America; ^3^Norwegian University of Life Sciences, Faculty of Science and Technology, Ås, Norway; ^4^University of Oslo, Department of Biosciences, Oslo, Norway; ^5^University of Oslo, Institute of Basic Medical Sciences, Oslo, Norway; ^6^Norwegian University of Science and Technology, Trondheim, Norway

##### **Correspondence:** Espen Hagen (espen.hagen@fys.uio.no)

*BMC Neuroscience* 2019, **20(Suppl 1)**: P272

Several large-scale data-collection efforts have been undertaken to characterize different brain areas in different species, including their underlying neuronal composition and circuitry down to the cellular, synaptic and molecular levels [1, 2]. Parallel efforts also aim to develop neuronal circuit models incorporating this vast biological detail, to explain/match various features observed in vivo and in vitro (e.g., [3, 4]). Such data-driven computer models can be used as frameworks for hypothesis testing and experimentation. Traditionally, comparison of experimental activity data and equivalent neuronal network models have been conducted at the level of spiking activity. Here, we aim to substantiate such a model by also comparing the commonly recorded local field potential (LFP), that is, the low frequency part of extracellularly recorded potentials, and the corresponding current source density (CSD).

Here, we use a model of the mouse primary visual cortex (area V1), currently in development at the Allen Institute for Brain Science (that will be made publicly available when it has been validated). The model incorporates ~230,000 neurons across each cortical layer, 21 neuronal classes, over 100 unique cell models, and over 200 connection classes. ~52,000 of the neurons are morphologically detailed multicompartment models, while remaining neurons are single-compartment models. The neuron model responses were fit to in vitro experimental measurements [5]. The use of multicompartment neuron models facilitates LFP predictions using electrostatic forward modeling [6,7]. Visual inputs are mediated by a feed-forward, filter-based model representing the retina and lateral geniculate nucleus (LGN) V1 pathway [8]. The V1 and LGN models are set up with the Brain Modeling ToolKit (BMTK, github.com/AllenInstitute/bmtk) in Python, and simulated using NEURON [9].

The experimental validation datasets are obtained using high-density multi-electrode arrays (Neuropixels [10]) inserted in V1 and LGN, and conventional laminar probes (NeuroNexus) inserted in V1. The awake, head-fixed mice are subjected to various visual stimuli (flashes, drifting gratings etc.).

We systematically compare activity observed in the V1 model with that recorded experimentally, at the level of spikes, LFP and CSD, for different types of visual stimuli. We also explore the use of laminar population analysis (LPA) [11] as a means to decompose laminar recordings of spikes and LFP/CSD into contributions from layer-specific populations. The LPA method is first validated on model output, before application on corresponding experimental data. By characterizing activity in multiple animals, we find which features of spiking activity, LFP CSD are conserved across all animals and which reflect individual variability and investigate whether these features are reproduced in simulations.

**References**

1. Koch C, Reid RC. Neuroscience: Observatories of the mind. *Nature* 2012 Mar 21;483(7390):397.
2. Kandel ER, Markram H, Matthews PM, Yuste R, Koch C. Neuroscience thinks big (and collaboratively). *Nature Reviews Neuroscience* 2013 Sep;14(9):659.
3. Potjans TC, Diesmann M. The cell-type specific cortical microcircuit: relating structure and activity in a full-scale spiking network model. *Cerebral cortex* 2012 Dec 2;24(3):785–806.
4. Markram H, Muller E, Ramaswamy S, et al. Reconstruction and simulation of neocortical microcircuitry. *Cell* 2015 Oct 8;163(2):456-92.
5. Gouwens NW, Berg J, Feng D, et al. Systematic generation of biophysically detailed models for diverse cortical neuron types. *Nature communications* 2018 Feb 19;9(1):710.
6. Holt GR, Koch C. Electrical interactions via the extracellular potential near cell bodies. *Journal of computational neuroscience* 1999 Mar 1;6(2):169-84.
7. Hagen E, Næss S, Ness TV, Einevoll GT. Multimodal Modeling of Neural Network Activity: Computing LFP, ECoG, EEG, and MEG Signals With LFPy 2.0. *Frontiers in neuroinformatics* 2018;12.
8. Arkhipov A, Gouwens NW, Billeh YN, et al. Visual physiology of the layer 4 cortical circuit in silico. *PLoS computational biology* 2018 Nov 12;14(11):e1006535.
9. Hines ML, Carnevale NT. The NEURON simulation environment. *Neural computation* 1997 Aug 15;9(6):1179-209.
10. Jun JJ, Steinmetz NA, Siegle JH, et al. Fully integrated silicon probes for high-density recording of neural activity. *Nature* 2017 Nov;551(7679):232.
11. Einevoll GT, Pettersen KH, Devor A, Ulbert I, Halgren E, Dale AM. Laminar population analysis: estimating firing rates and evoked synaptic activity from multielectrode recordings in rat barrel cortex. *Journal of neurophysiology* 2007 Mar;97(3):2174–90.

## P273 Impact of intrinsic neuronal properties in cortical network-dynamics

### Leonardo Dalla Porta^1^, Almudena Barbero^1^, Albert Compte^1^, Maria V. Sanchez-Vives^1,2^

#### ^1^IDIBAPS, Systems Neuroscience, Barcelona, Spain; ^2^ICREA, Systems Neuroscience, Barcelona, Spain

##### **Correspondence:** Leonardo Dalla Porta (leonardodallaporta@gmail.com)

*BMC Neuroscience* 2019, **20(Suppl 1)**: P273

The cerebral cortex exhibits a rich dynamic repertoire of activity, ranging from highly synchronized states (e.g. in slow wave sleep) to desynchronized states (e.g. awake). These collective phenomena are the product of the interaction between single neurons that are endowed with diverse ionic channels with complex biophysical properties. The study of the mechanisms that control emergent network activity in the cortex is often focused on synaptic properties and network connectivity. Our objective here is to further elucidate the impact of ion channels on cortical dynamics, bridging the divide between the individual neuron and the network. To this end, we investigated here a model of spontaneous activity in the form of slow oscillations (SO), which consist of periods of high neuronal firing (Up states) interleaved with periods of near-silence (Down states). SO dynamics and in particular Down states have been associated with activity-dependent K+ channels, such as Ca2+-dependent K+-current (KCa) and Na+-dependent K+-current (KNa) channels [1].

*In vitro* extracellular multiunit recordings were obtained from cortical slices under physiological conditions and bath-application of apamin, a KCa channel blocker. Also, in a data-based computational network model of Up and Down states, we parametrically varied the fraction of neurons—as well as their conductance—that includes KCa and KNa channels. Experimentally we found that properties of the network, such as Up and Down state duration and the frequency of SO and its firing rate, were affected when KCa was partially blocked. These findings were also reproduced by the computational model, which also predicted that KNa is essential for maintaining the network in a bistable state. Furthermore, we found that the ionic properties of individual neurons become network properties through synaptic recurrency, such that the extracellular and intracellular recordings of neurons without these ionic currents are indistinguishable from those with them. Together our findings highlight the challenge of disentangling the link between intrinsic and synaptic mechanisms derived from the dynamics of emergent oscillatory activity. They also suggest these off-periods caused by potassium currents share properties with those described in the brain of unresponsive wakefulness syndrome patients that have been found to disrupt causality and complexity [2].

**Acknowledgments:** Funded by the Spanish Ministry of Science (BFU2017-85048-R) and EU H2020 Research and Innovation Programme, Grant 720270 (HBP SGA2).

**References**

1. Compte A, et al. Cellular and network mechanisms of slow oscillatory activity (<1 Hz) and wave propagations in a cortical network model. *Journal of Neurophysics* 2003, 89, 2707.
2. Rosanova M, et al. Sleep-like cortical OFF-periods disrupt causality and complexity in the brain of unresponsive wakefulness syndrome patients. *Nature Communications* 2018, 9, 4427.

## P274 Wave propagation, dynamical richness and predictability under different brain states

### Alessandra Camassa^1^, Miguel Dasilva^1^, Maurizio Mattia^2^, Maria V. Sanchez-Vives^1,3^

#### ^1^IDIBAPS, Systems Neuroscience, Barcelona, Spain; ^2^Istituto Superiore di Sanità, National Center for Radiation Protection and Computational Physics, Roma, Italy; ^3^ICREA, Systems Neuroscience, Barcelona, Spain

##### **Correspondence:** Alessandra Camassa (camassa@clinic.cat)

*BMC Neuroscience* 2019, **20(Suppl 1)**: P274

Propagating waves of cortical activity are dynamical patterns that occur across different brain states and are also present in unconscious states [1]. In this study we aimed to characterize different brain states characterizing the changes occurring to the spatiotemporal dynamics of slow-wave activity in multichannel data. During the sleep-like slow oscillations (SOs,<1Hz), activation waves propagate across the cortical network both in vitro [2] and in vivo in anesthetized animals [3]. By varying the anesthesia levels, it is possible to vary the brain state [4]. Here, we varied the anesthesia levels without departing from the slow-wave activity regime. The emergent oscillatory activity ranged from lower (0.12 Hz) to higher (1.15 Hz) frequency for low to high anesthesia levels respectively. We recorded the extracellular local field potential (LFP) with a superficial 32-channel electrode-array placed on the surface of the brain of eight mice anesthetized at three different levels. To reconstruct the spatiotemporal dynamics of propagating waves in multichannel recordings we developed a phase-based method. We computed the instantaneous phase at each electrode via the analytic signal obtained using the Hilbert transform of our time series, and used the latency in absolute time to a given phase crossing [5] to reconstruct the spatiotemporal profile of the waves across the space covered by the electrode grid. By considering each electrode as a node of a network, we estimated the synchrony at the network-level as a function of time by computing the Kuramoto order parameter [6]. We then evaluated the differences in the synchronization dynamics across the different anesthesia levels. As a result, we were able to define the patterns of propagating activity under each anesthesia level, to study their dynamical evolution in time, and estimate, for each brain state, the overall dynamical richness and sequence predictability. Overall, our findings allowed us to characterize the evolution of the cortical spatiotemporal dynamics under different brain states within the SO regime, revealing that the wave propagation patterns change together with the brain state showing higher dynamical richness and lower predictability in lighter anesthesia states, supporting the idea of an increasingly complex brain activity that varies when we move from deeply unconscious states towards wakefulness.

**Acknowledgements**: Funded by the Spanish Ministry of Science (BFU2017-85048-R) and by EU H2020 Research and Innovation Programme, Grant 720270 (HBP SGA2)

**References**

1. Sanchez-Vives MV, Massimini M, Mattia M. Shaping the default activity pattern of the cortical network. *Neuron* 2017 Jun 7;94(5):993–1001.
2. Sanchez-Vives MV, McCormick DA. Cellular and network mechanisms of rhythmic recurrent activity in neocortex. *Nature neuroscience* 2000 Oct;3(10):1027.
3. Ruiz-Mejias M, Ciria-Suarez L, Mattia M, Sanchez-Vives MV. Slow and fast rhythms generated in the cerebral cortex of the anesthetized mouse. *Journal of neurophysiology* 2011 Aug 31;106(6):2910–21.
4. Tort-Colet N, Capone C, Sanchez-Vives MV, Mattia M. Attractor competition enriches cortical dynamics during awakening from anesthesia. *BioRxiv* 2019 Jan 1:517102.
5. Muller L, Reynaud A, Chavane F, Destexhe A. The stimulus-evoked population response in visual cortex of awake monkey is a propagating wave. *Nature communications* 2014 Apr 28;5:3675.
6. Arenas A, Díaz-Guilera A, Kurths J, Moreno Y, Zhou C. Synchronization in complex networks. *Physics reports* 2008 Dec 1;469(3):93–153.

## P275 Classification of brain states across the awake-sleep transition in the cortex of rats.

### Belen de Sancristobal^1^, Ruben Moreno-Bote^1,2^, Melody Torao^3^, Maria V. Sanchez-Vives^3,4^

#### ^1^Universitat Pompeu Fabra, Department of Information and Communication Technologies, Barcelona, Spain; ^2^Universitat Pompeu Fabra, Center for Brain and Cognition, Barcelona, Spain; ^3^IDIBAPS, Barcelona, Spain; ^4^ICREA, Systems Neuroscience, Barcelona, Spain

##### **Correspondence:** Belen de Sancristobal (belen.sancristobal@upf.edu)

*BMC Neuroscience* 2019, **20(Suppl 1)**: P275

The electrophysiological signals recorded both in the cortex and in the thalamus exhibit clearly distinct dynamical features during wakefulness and sleep, particularly during nonrapid eye movement (NREM) sleep, or anesthesia. The former is characterized by high frequencies and low voltage activity. The latter shows high-amplitude slow oscillatory activity and a reduction of behavioral responses to sensory stimuli. However, it remains unclear how the different stages of sensory processing are modulated by each brain state (and its idiosyncratic dynamics), leading to different perceptual thresholds. Recent findings have revealed that changes in consciousness levels correspond to changes in the interactions between functionally specialized brain regions [1]. Therefore, differences across brain states not only arise locally, but are also manifested in the effective connectivity among widespread cortical areas. Indeed, local dynamics fail to explain differences between wakefulness and rapid eye movement (REM) sleep, since, at this stage, cortical activity exhibits wake-like dynamics but the sensory threshold is raised above waking levels similar to NREM sleep. In this study we have focused on natural sleep, and we have recorded cortical local field potentials (LFP) of rats while they naturally transitioned from wakefulness to sleep and vice-versa [2]. Scoring arousal levels is a time-consuming task that, in many laboratories, is manually performed by experts. Besides, the wake-sleep cycle of rodents is more fragmented than in humans and the presence of REM epochs is lower [3]. Here we present a classification algorithm that can facilitate state detection in chronic animal experiments that last several days. Automatic scoring programs that have been published to date are mainly based on decision trees that require threshold criteria for the choice of sleep-wake states [4,5] or on supervised learning algorithms [6]. Here we present a multinomial logistic regression where the labels are manually assigned based on visual inspection of the LFP, EMG and behavior. Freely-moving rats are monitored during the awake-sleep cycle until enough data is collected to train the classifier and to accurately predict (> 80%) wake, REM and NREM. Rats were implanted in the visual, motor, prefrontal, parietal association cortex and the auditory cortex. The animal movement is videotaped to later track its position within the cage. We found that power spectral density ratios of beta-gamma and theta-delta in 5-s epochs can be successfully used as predictors to classify the three states (A). Training of the classifier is performed offline and individually for each subject. The optimal weights are then used for an online detection of states. We tested the performance of the classifier under its different operation modes: homogeneous versus heterogeneous weighting of the LFP channels, EMG versus video tracking (B and C), different temporal epochs and different choices of predictors. In summary, we present a fully-characterized classifier, which can use different neuronal/behavioral signals for detecting awake, REM and NREM. Moreover, reliable online identification of brain states allows simultaneous computations of effective connectivity estimates across the recording sites, which provide an online large-scale description of those states.

**Fig. 1:**
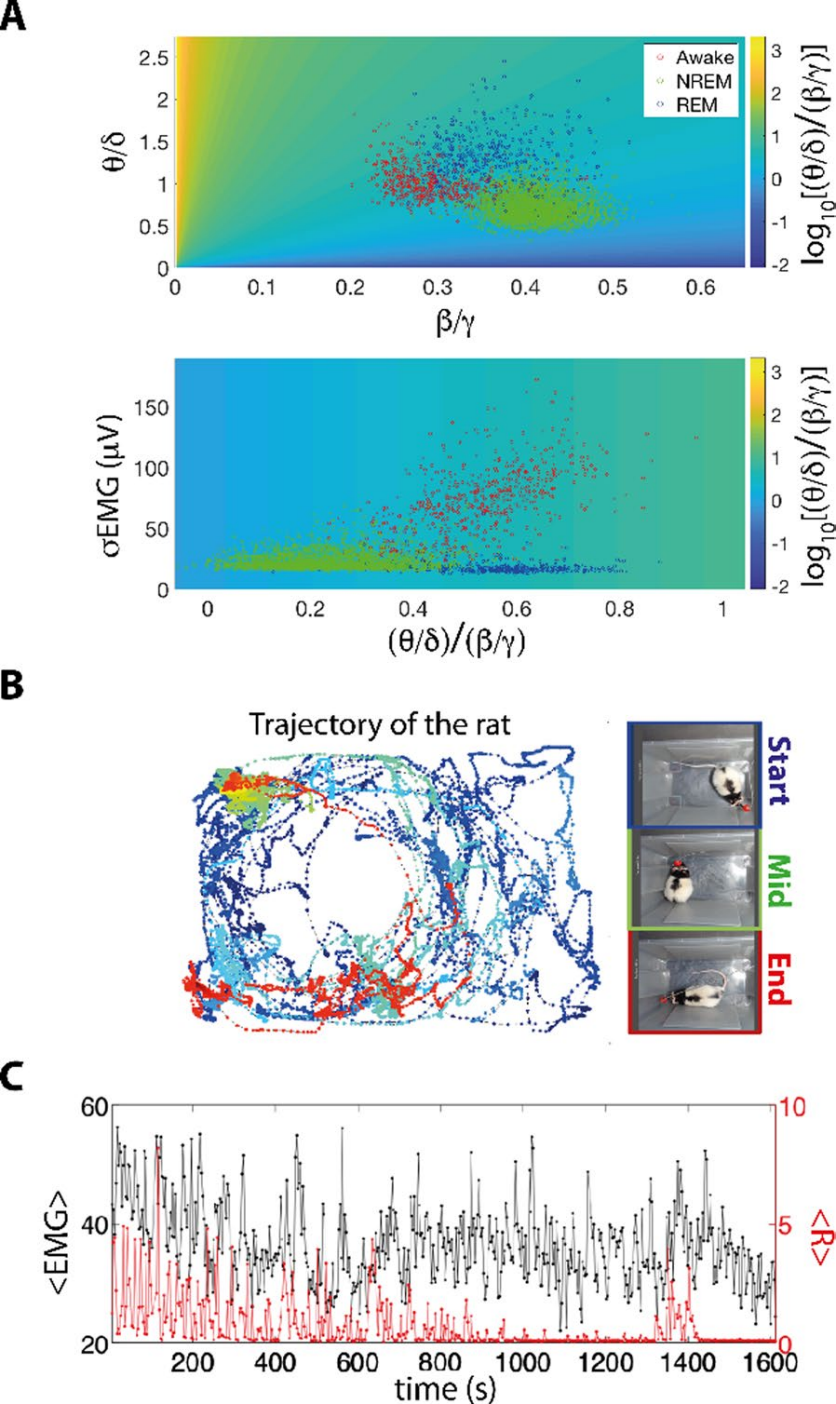
Characterization of a single rat’s LFP, EMG and location. **a** 2D map of the predictors, theta-delta ratio versus beta-gamma ratio, computed over 5s-windows of the averaged LFP (upper plot). The standard deviation of the EMG computed in the same temporal window as a function of the ratios of the predictors (bottom plot). **b** Trajectory of the rat during a recording session obtained from a video trac

**References**

1. Stitt I, Hollensteiner KJ, Galindo-Leon E, et al. Dynamic reconfiguration of cortical functional connectivity across brain states. *Scientific reports* 2017 Aug 18;7(1):8797.
2. Nir Y, Vyazovskiy VV, Cirelli C, Banks MI, Tononi G. Auditory Responses and Stimulus-Specific Adaptation in Rat Auditory Cortex are Preserved Across NREM and REM Sleep. *Cerebral Cortex* 2015 May;25(5):1362.
3. Bastianini S, Berteotti C, Gabrielli A, Martire VL, Silvani A, Zoccoli G. Recent developments in automatic scoring of rodent sleep. *Archives italiennes de biologie* 2015 Sep 30;153(2–3):58–66.
4. Hamrahi H, Stephenson R, Mahamed S, Liao KS, Horner RL. Selected Contribution: Regulation of sleep-wake states in response to intermittent hypoxic stimuli applied only in sleep. *Journal of Applied Physiology* 2001 Jun 1;90(6):2490–501.
5. Louis RP, Lee J, Stephenson R. Design and validation of a computer-based sleep-scoring algorithm. Journal of neuroscience methods 2004 Feb 15;133(1–2):71–80.
6. Rytkönen KM, Zitting J, Porkka-Heiskanen T. Automated sleep scoring in rats and mice using the naive Bayes classifier. *Journal of neuroscience methods* 2011 Oct 30;202(1):60–4.

## P276 Propagating densities of spontaneous activity in cortical slices

### Roman Arango^1^, Pedro Mateos-Aparicio^2^, Emili Balaguer-Ballester^1^, Maria V. Sanchez-Vives^2,3^

#### ^1^Bournemouth University, Department of Computing and Informatics, Bournemouth, United Kingdom; ^2^IDIBAPS, Systems Neuroscience, Barcelona, Spain; ^3^ICREA, Systems Neuroscience, Barcelona, Spain

##### **Correspondence:** Roman Arango (rcabrera@bournemouth.ac.uk)

*BMC Neuroscience* 2019, **20(Suppl 1)**: P276

Slow oscillations (SO) of neural activity emerge spontaneously in the neocortex during functionally-disconnected brain states (e.g. deep anaesthesia or slow-wave sleep). A multi-scale phenomenon consisting of the alternation (ca. 1 Hz) of high- (UP) and low-responsive (DOWN) periods, the SO propagate spatio-temporally as a travelling wave, which reveals properties of the underlying cortical network. Even though the SO would act as its preferred global state, the network can be driven into other richer dynamical states by neuromodulation, inducing for example the transition from sleep to wakefulness. How such a highly-synchronized state (the SO) may give rise spontaneously to an awake-like interdependent state is something that some modelling approaches have partially addressed. Yet identifying the way the network dynamics spatially evolve when the SO breaks down is still an open question. For example, when the SO weakens, faster and irregular oscillations appear interleaved with asynchronous periods, whose dynamics are poorly known.

We analysed extracellular multi-electrode array recordings that were carried out on acute slices from the ferret’s V1 cortex. The slices exhibited a robust SO activity and were then subjected to neurochemical modulations aimed at eliciting an awake-like state (AS). In order to capture population firing rates of the nearby neuronal ensembles, we proposed an enhanced estimation of the multi-unit activity (MUA). We suggest that locally sampled probability densities of the MUA reflect the state of the network at different spatial and temporal scales, within and beyond the SO state.

By determining shape parameters of these MUA densities, we provided a data-driven definition of UP/DOWN states from a neuronal-ensemble perspective, without further assumptions on their properties. While DOWN, and in general, low-activity states are well fitted by gamma probability densities, residual upper-tail subdensities account for UP-like higher-activity states. Thus, our new technique allows to identify alternating states other than UP/DOWN when the SO regime weakens.

Furthermore, the embedding of the MUA densities in a suitable metric space enabled us to compare the network activity at different periods and locations. Our approach reveals the emergence, during the AS, of a particular spatial clustering pattern that seems to follow the laminar structure of the slice. More generally, we have devised a theoretical procedure to evaluate the propagation of the MUA densities based on cross-correlation analyses of the Kolmogorov-Smirnov statistic. This way we could capture transient spatiotemporal changes in the underlying distributions.

This study has again confirmed that the SO propagate longitudinally across the network in waves, although in a rather more complex way as previously reported. Interestingly, our method can also be applied to the AS case, in which a propagating wave-front cannot be identified like in the SO regime. Preliminary results suggest that, during the AS, UP-like bursts of activity emerge and propagate amid short asynchronous periods.

## P277 Recovery time after stimulation in mice characterizes brain complexity under different levels of anesthesia

### Manel Vila-Vidal^1^, Ane López-González^1^, Miguel Dasilva^2^, Gustavo Deco^3^, Maria V. Sanchez-Vives^2,4^

#### ^1^Universitat Pompeu Fabra, Center for Brain and Cognition, Barcelona, Spain; ^2^Institut d’Investigacions Biomèdiques August Pi i Sunyer (IDIBAPS), Barcelona, Spain; ^3^Universitat Pompeu Fabra, Barcelona, Spain; ^4^ICREA, Systems Neuroscience, Barcelona, Spain

##### **Correspondence:** Manel Vila-Vidal (m@vila-vidal.com)

*BMC Neuroscience* 2019, **20(Suppl 1)**: P277

High cognitive functions critically rely on the ability of the brain to sustain complex activity patterns that are characterized by an optimal balance between functional integration and segregation in cortical circuits, a feature also known as brain complexity [1, 2]. Over the last decade, different metrics have been proposed to assess brain complexity based on the analysis of graph theoretical properties of spontaneous activity [1] or on the algorithmic complexity of perturbation-evoked responses [2]. These indices have proven to discriminate different levels of consciousness both in humans and animals.

Here, we aim to investigate the temporal fading of the perturbation-evoked high-correlation states in cortical circuits as a function of the level of consciousness. To this end, three levels of anesthesia (deep, mid and light) were selectively induced in mice by means of varying isoflurane levels (2% to 0). Spontaneous extracellular LFP activity was recorded with 32 channel multielectrode arrays covering almost entirely the right hemisphere (Fig. 1A). We subsequently delivered electrical stimulation through a bipolar electrode aimed at the infragranular layers of the frontal or occipital cortex at a distance of 500 µm from the edge of the electrode array. In all cases, we computed the time course of the correlation between the broadband LFP signals using a sliding window approach (width of 200 ms and step of 100 ms). For each window, we extracted the functional integration as proposed in [1] (Fig. 1B).

**Fig. 1:**
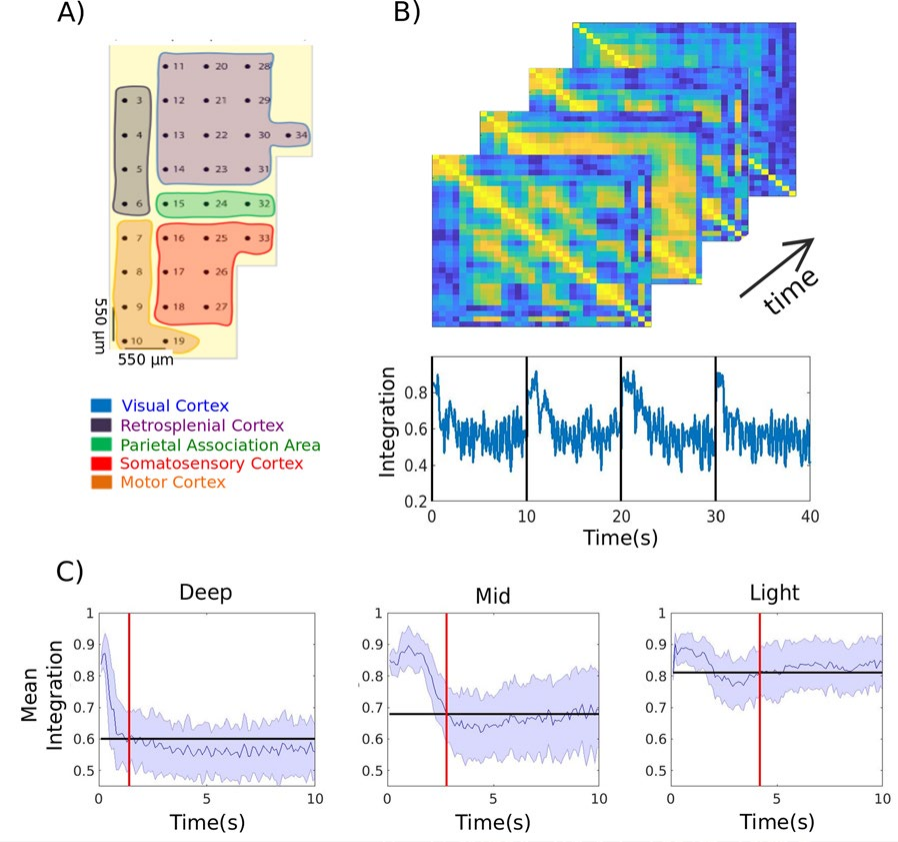
**a** Location of the electrodes used for the recordings (left hemisphere, from the top when placed on the brain). **b** FC matrices and global integration are computed across time using a sliding window approach. Black bars correspond to stimulations. **c** Stimulus-evoked integration is averaged across trials (blue) and the recovery time, defined as the time it takes for the network integration to reach stable values, is extracted (marked in red)

The average integration of spontaneous activity was found to be higher in lighter anesthesia, in accordance with previous studies [3]. We then analyzed the effect of the electrical stimulation, finding a significant increase of the integration after stimulation in all cases. Preliminary results show that the duration of the stimulus-evoked response in the network depends on the level of anesthesia. In particular, we showed that the recovery time, defined as the time it takes for the network integration to reach stable values after the stimulus is delivered, is larger in states of light anesthesia, suggesting a more complex processing of the delivered stimulus.

Overall, we proposed a novel measure to define brain complexity based on the recovery time of the brain network integration after a perturbation is delivered, being this time significantly longer in light than in deep anesthesia.

**Acknowledgements**: M.V. is supported by ‘la Caixa’ Foundation (ID 100010434, fellowship code: LCF/BQ/DE17/11600022). G.D. is supported by the Spanish Ministry Research Project PSI2016-75688-P (AEI/FEDER) and by the EU H2020 FET Flagship Human Brain Project 785907 (HBP SGA2). M.V.S.V is supported by the Spanish Ministry of Science (BFU2017-85048-R) to MVSV and by EU H2020 Research and Innovation Programme, Grant 720270 (HBP SGA2).

**References**

1. Casali AG, Gosseries O, Rosanova M, et al. A theoretically based index of consciousness independent of sensory processing and behavior. *Science translational medicine*. 2015, *5*(198), 198ra105
2. Deco G, Tononi G, Boly M, et al. Rethinking segregation and integration: contributions of whole-brain modelling. *Nature Reviews Neuroscience*. 2015, *16*(7), 430.
3. Bettinardi RG, Tort-Colet N, Ruiz-Mejias M, et al. Gradual emergence of spontaneous correlated brain activity during fading of general anesthesia in rats: evidences from fMRI and local field potentials. *Neuroimage*. 2015, *114*, 185–198.

## P278 Unsupervised learning of sparse spatio-temporal receptive fields through inhibitory plasticity; A model of the mammalian early visual system

### Samuel Sutton, Volker Steuber, Michael Schmuker

#### University of Hertfordshire, Biocomputation Research Group, Hatfield, United Kingdom

##### **Correspondence:** Samuel Sutton (s.sutton3@herts.ac.uk)

*BMC Neuroscience* 2019, **20(Suppl 1)**: P278

Receptive fields of V1 simple cells in mammalian visual cortex are characterised as localized, oriented bandpass filters that respond to oriented contrast edges of a specific spatial scale in a particular area of the visual fields. Olshausen and Field [1] showed that V1 receptive fields can be obtained with an algorithm that learns basis functions from natural image patches by maximising sparsity whilst prioritising the preservation of information. However, Olshausen & Field’s algorithm is lacking biorealism on several fronts. First, it uses static image patches, whereas real brain activity is dynamic and input is constantly changing. Second, it assumes a rate code, assigning real numbers to the activity of neurons, instead of timed events as in spike trains.

Our goal is to develop a spiking network that employs event-based synaptic update rules to learn dynamic receptive fields. Input to our system will be provided by an event-based camera that transmits “spikes” whenever the brightness at a pixel crosses a threshold (Dynamic Vision Sensor (DVS), Inivation, Zurich, Switzerland) [2]. In addition, we aim to learn receptive fields in real-time by leveraging the neuromorphic SpiNNaker platform to accelerate the spiking network simulation [3]. We are using a balanced E/I network with spike-timing dependent plasticity at inhibitory synapses, with 800 excitatory (E) neurons and 200 inhibitory (I) ones. The first step was to parameterise the network in the asynchronous-irregular (AI) regime to ensure it is reactive to input [4]. We achieved an AI state, even under temporally dynamic input. The next step was to expose the network to defined stimuli where the ground truth is known for the sparse basis functions that the network is supposed to learn. To this end, we generated surrogate data consisting of oriented spike fronts travelling across the receptive field at a set of predefined orientations, mimicking what the DVS sensor would output in response to moving oriented lines.

We exposed the network to up to 1 hour of such oriented contrast edges, with 5 edges per second, and orientation picked randomly from one of 0, 45,90, 135, 180, 225, 270 and 315 degrees. For each spike in the E population, we computed the average reverse correlation with each input pixel at different time points. Although some cells clearly showed a preference to certain orientations and angles, no clear pattern has yet emerged that would demonstrate learning of V1-like receptive fields (Fig. 1). Further work will thus concentrate on exploring the parameter space of the network and learning rule, as well as the presentation statistics of the stimuli to support receptive field formation. Ultimately, the network will ideally learn V1-like receptive fields from long exposure to DVS recordings. Our results will hopefully aid further understanding of the mechanisms of receptive field emergence and efficient event-based vision in general.

**Fig. 1:**
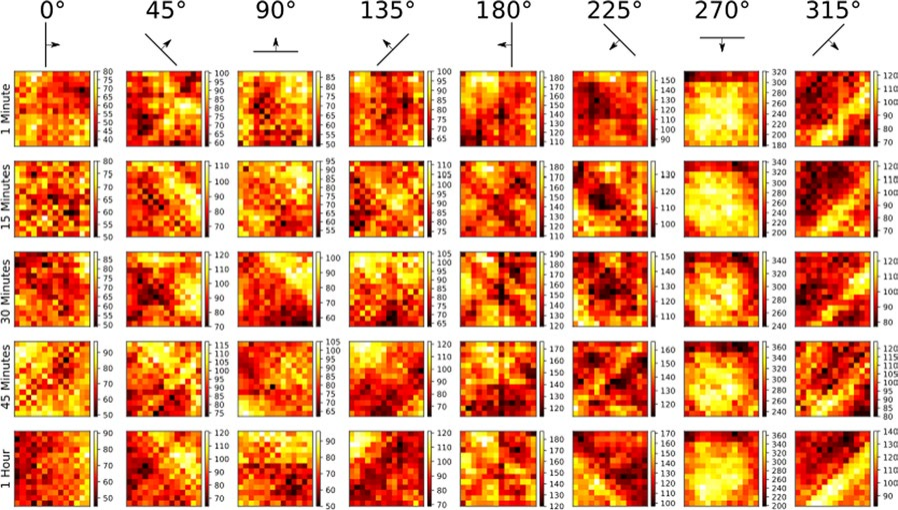
Reverse correlated activity receptive fields of 8 selected neurons after 1 minute, 15 minutes, 30 minutes, 45 minutes and 1 hour of being exposed to randomized oriented contrast edges. The color represents the number of correlated spikes that occurred in the input population during 4ms before any spike of the selected neuron over a simulated minute

**References**

1. Olshausen BA, Field DJ. Wavelet-like receptive fields emerge from a network that learns sparse codes for natural images. *Nature* 1996, 381, 607–609.
2. Posch C, et al. Retinomorphic Event-Based Vision Sensors: Bioinspired Cameras with Spiking Output. *Proceedings of the IEEE* 2014, 102, 1470–1484
3. Furber S, et al. Overview of the SpiNNaker System Architecture. *IEEE Transactions on Computers* 2013, 62, 2454–2467
4. Deneve S, Machens CK, Efficient codes and balanced networks. *Nature Neuroscience* 2016, 19, 375–382

## P279 Capacitance clamp

### Paul Pfeiffer^1^, Federico José Barreda Tomás^2^, Jiameng Wu^3^, Jan-Hendrik Schleimer^1^, Imre Vida^2^, Susanne Schreiber^1^

#### ^1^Humboldt-Universität zu Berlin, Institute for Theoretical Biology, Berlin, Germany; ^2^Charité-Universitätsmedizin Berlin, Institute for Integrative Neuroanatomy, Berlin, Germany; ^3^Humboldt-Universität zu Berlin, Bernstein Center for Computational Neuroscience, Berlin, Germany

##### **Correspondence:** Paul Pfeiffer (pfeifferpaul90@gmail.com)

*BMC Neuroscience* 2019, **20(Suppl 1)**: P279

A basic time scale in neural dynamics from single cells to the network level is the membrane time constant—set by a neuron’s input resistance and its capacitance. Interestingly, the membrane capacitance appears to be more dynamic than previously assumed with implications for neural function and pathology. Indeed, altered capacitance has been observed in reaction to neural swelling [1], but also in ageing and Alzheimer’s disease [2]. Importantly, according to theory, even small capacitance changes can induce a qualitative switch in spike generation and affect neuronal signal processing, e.g. increased network synchronization [3]. In experiment, robust methods to modify the capacitance of a neuron have been missing. Here, we present the capacitance clamp—an electrophysiological method for capacitance control based on an unconventional application of the dynamic clamp.

In its original form, dynamic clamp mimics additional synaptic or ionic conductances by injecting their respective currents [4]. Whereas a conductance directly governs a current, the membrane capacitance determines how fast the voltage responds to a current. Accordingly, capacitance clamp mimics an altered capacitance by injecting a dynamic current that slows down or speeds up the voltage response (Fig 1 A). For the dynamic current, the experimenter only has to specify the original cell and the desired target capacitance. In particular, capacitance clamp requires no detailed model of present conductances and thus can be applied in every excitable cell.

**Fig. 1:**
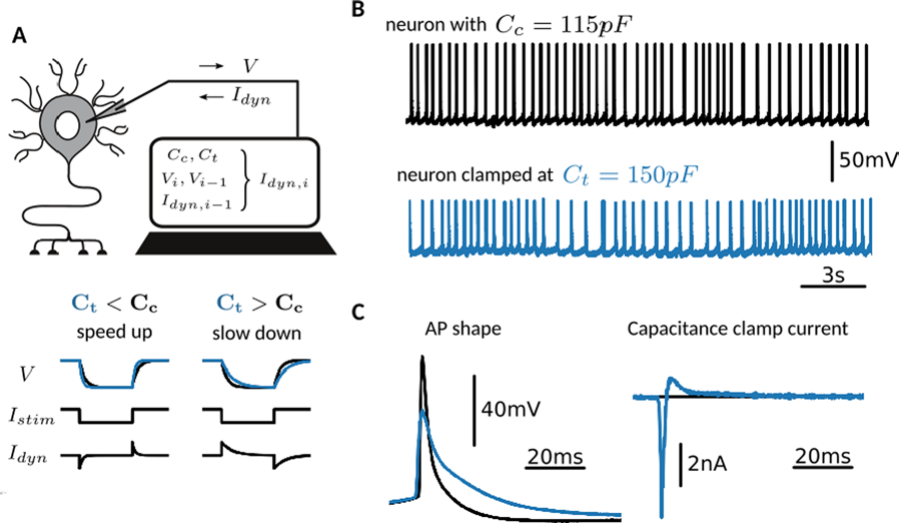
The capacitance clamp. A With the original cell and the desired target capacitance (Cc, Ct), the dynamic clamp adjusts the current I_dyn to mimic the change of capacitance. B Spike trains of a cultured neuron: original at Cc = 115pF (black) and clamped at Ct = 150pF (blue). More irregular spiking suggests passing of the SNL bifurcation. C Spike shapes from B (l.) and capacitance clamp currents (r.)

To validate the capacitance clamp, we performed numerical simulations of the protocol and applied it to modify the capacitance of cultured neurons. First, we simulated capacitance clamp in conductance-based neuron models to verify altered capacitance. Second, in cultured hippocampal neurons from rats, we could reliably control the capacitance in a range of 75 to 200% of the original capacitance and observed pronounced changes in the shape of the action potentials: increasing the capacitance reduced after-hyperpolarization amplitudes and slowed down repolarization. Third, we studied the saddle-node loop (SNL) bifurcation, a particular switch in spike onset generation that leads to stuttering and increased network synchronization [3]. Our preliminary results indicate that we may reversibly shift neurons over this SNL point via capacitance clamp (Fig. 1 B, C). With robust control over capacitance, we aim next to map how close different neuron types are to this critical switch.

To conclude, we present a novel tool for electrophysiology: the capacitance clamp provides reliable control over the capacitance of a neuron and thereby opens a new way to study the temporal dynamics of excitable cells.

**Acknowledgements**: The work was supported by BMBF (01GQ0901, 01GQ1403) and DFG (GRK 1589/2). We are grateful to Jan Benda for support with dynamic clamp.

**References**

1. Amzica F, et al. Membrane Capacitance of Cortical Neurons and Glia During Sleep Oscillations and Spike-Wave Seizures. *J. Neurophysiol*. 2017, 82, 2731–2746.
2. Brown JT, et al. Altered intrinsic excitability of hippocampal CA1 pyramidal neurons in aged PDAPP mice. *Front. Cell. Neurosci*. 2015, 9, 1–14.
3. Hesse J, et al. Qualitative changes in phase-response curve and synchronization at the saddle-node-loop bifurcation. *Phys. Rev. E* 2017, 95.
4. Sharp AA, et al. The dynamic clamp: artificial conductances in biological neurons. *Trends Neurosci*. 1993, 16, 389–394.

## P280 Biologically realistic behaviors from a superconducting neuron model

### Patrick Crotty^1^, Kenneth Segall^1^, Daniel Schult^2^

#### ^1^Colgate University, Department of Physics and Astronomy, Hamilton, NY, United States of America; ^2^Colgate University, Department of Mathematics, Hamilton, United States of America

##### **Correspondence:** Patrick Crotty (pcrotty@colgate.edu)

*BMC Neuroscience* 2019, **20(Suppl 1)**: P280

We have introduced [1] and discussed the synchronization properties [2, 3] of a circuit containing superconducting Josephson junctions that in many respects mimics the dynamics of biological neurons. In this study, we explore computationally whether our “Josephson junction neuron” can reproduce the different dynamical behaviors tabulated by Izhikevich [4] and reproducible by his model. For different choices of the circuit and input current parameters, our model can indeed reproduce several of these behaviors like phasic spiking, tonic spiking, and tonic bursting, as shown in (Fig. 1), as well as others. This suggests that Josephson junction neurons could be constructed to simulate different classes of biological neurons. As these circuits have picosecond-scale dynamics and operate fully in parallel, they would potentially allow for much faster simulation of neural circuits and brain regions than is achievable with conventional computers, even with massively parallel architectures.

**Fig. 1:**
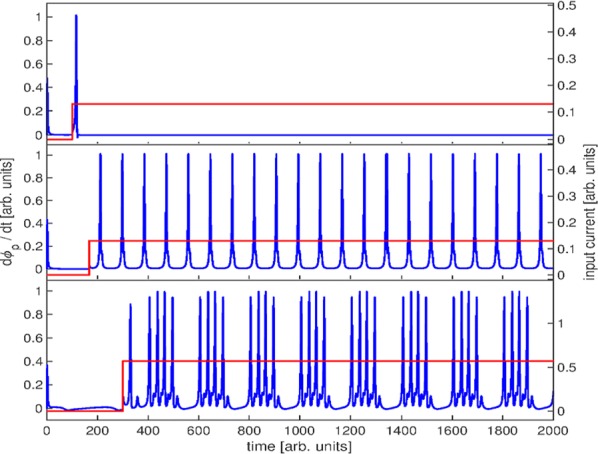
Response (blue) of simulated Josephson junction neuron to current step (red) for different circuit parameters, showing phasic spiking, tonic spiking, and tonic bursting. DC bias current is used for first two cases, AC for third. Membrane potential is represented by derivative of quantum-mechanical phase across one of the junctions (which produces a voltage). All quantities nondimensionalized

**References**

1. Crotty P, Schult D, Segall K. Josephson junction simulation of neurons. *Physical Review E* 2010 Jul 19;82(1):011914.
2. Segall K, Guo S, Crotty P, Schult D, Miller M. Phase-flip bifurcation in a coupled Josephson junction neuron system. *Physica B: Condensed Matter* 2014 Dec 15;455:71–5.
3. Segall K, LeGro M, Kaplan S, et al. Synchronization dynamics on the picosecond time scale in coupled Josephson junction neurons. *Physical Review E* 2017 Mar 22;95(3):032220.
4. Izhikevich EM. Which model to use for cortical spiking neurons? *IEEE transactions on neural networks* 2004 Sep;15(5):1063–70.

## P281 Calculating local field potential from spiking neural network model

### Rahmi Elibol^1,2^, Neslihan Serap Sengor^2^

#### ^1^Erzincan University, Istanbul, Turkey; ^2^Istanbul Technical University, Electronics and Communication Engineering Dept., Istanbul, Turkey

##### **Correspondence:** Rahmi Elibol (rahmielibol@itu.edu.tr)

*BMC Neuroscience* 2019, **20(Suppl 1)**: P281

Local field potential (LFP) is electrophysiological signal that is used as a marker of cognitive processes and malfunctioning of neural structures in neuroscience. LFP is formed by currents and dipoles, and measured with array electrodes. In other words, they are created by the effect of synaptic currents due to the synaptic inputs to the dendrites, regardless of the neurons producing the spikes. The measurement results are generally interpreted using frequency analysis, as in EEG data [1, 2].

In order to obtain these empirical results with computational models, there has to be a method to calculate the LFP. Although there are some methods given in the literature, these methods are mainly focused on networks composed of morphologically modeled neurons [3, 4]. While some methods are designed for point neurons, they also try to model LFP data by segmenting the neurons [5, 6].

Here, we focus on obtaining results from computational models that can be compared with empirical LFP data. The computational model is formed as a spiking neural network consisting of point neurons. The spiking point neuron model is created in 3 dimensions. A random coordinate is assigned for each neuron that corresponds to their physiological dimensions. The distance of the neurons from the electrodes placed in the structure is determined. Then based on the distance of each neuron to electrodes and the total synaptic current for each neuron LFP values are obtained.

The proposed model for calculating LFP is tested on a computational model of nucleus accumbens where the role of dopamine from ventral tegmental area on LFP is investigated. The LFP and the frequency analysis related are given in (Fig. 1). As it can be followed from beta band is observed in resting state as mentioned in literature [7, 8].

**Fig. 1:**
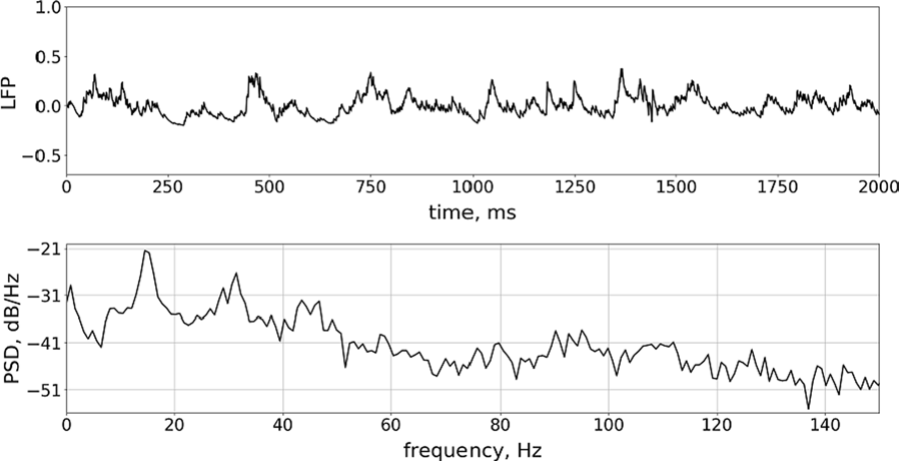
LFP and Power spectrum (PSD) of Nucleus Accumbens model which is obtained using modified Izhikevich neuron model

**References**

1. Buzsáki G, Anastassiou CA, Koch C. The origin of extracellular fields and currents — EEG, ECoG, LFP and spikes. *Nature Reviews Neuroscience* 2012, 13, 407–420.
2. Bédard C, Destexhe, A. Local field potentials. In: Brette R, Destexhe A, editors. Handbook of Neural Activity Measurement. Cambridge University Press 2012, 136–191.
3. Lindén H, Hagen E, Leski S, et al. LFPy: a tool for biophysical simulation of extracellular potentials generated by detailed model neurons. *Frontiers in Neuroinformatics* 2014,7, 41.
4. Parasuram H, Nair B, D’Angelo E, at al. Computational Modeling of Single Neuron Extracellular Electric Potentials and Network Local Field Potentials using LFPsim. *Frontiers in Computational Neuroscience* 2016,10,65.
5. Hagen E, Dahmen D, Stavrinou ML, et al. Hybrid Scheme for Modeling Local Field Potentials from Point-Neuron Networks. *Cerebral Cortex* 2016, 26(12), 4461–4496.
6. Mazzoni A, Lindén H, Cuntz H, et al. Computing the Local Field Potential (LFP) from Integrate-and-Fire Network Models. *PLOS Computational Biology* 2015, 11(12), e1004584.
7. Cohen MX, Axmacher N, Lenartz D, et al. Good Vibrations: Cross-frequency Coupling in the Human Nucleus Accumbens during Reward Processing. *Journal of Cognitive Neuroscience* 2009, 21(5), 875–889.
8. Stenner MP, Dürschmid S, Rutledge RB, et al. Perimovement decrease of alpha/beta oscillations in the human nucleus accumbens. *Journal of Neurophysiology* 2016,116(4), 1663–1672.

## P282 Lagrangian neurodynamics for real-time error-backpropagation across cortical areas

### Dominik Dold^1^, Akos Ferenc Kungl^1^, João Sacramento^2^, Mihai A. Petrovici^3^, Kaspar Schindler^4^, Jonathan Binas^5^, Yoshua Bengio^5^, Walter Senn^3^

#### ^1^Heidelberg University, Kirchhoff Institute for Physics, Germany; ^2^UZH / ETH, Institute of Neuroinformatics, Zurich, Switzerland; ^3^University of Bern, Department of Physiology, Bern, Switzerland; ^4^Inselspital Bern, University of Bern, Department of Neurology, Bern, Switzerland; ^5^University of Montreal, MILA, Montreal, Canada

##### **Correspondence:** Dominik Dold (dominik.dold@kip.uni-heidelberg.de)

*BMC Neuroscience* 2019, **20(Suppl 1)**: P282

The hierarchical structure of the cortex raises the question how plasticity in the brain is able to shape such a structure in the first place. The distant cousins of biological neurons, deep abstract neural networks, are commonly trained with the backpropagation-of-errors algorithm (backprop), which solves the credit assignment problem for deep neural networks and is behind many of the recent achievements of deep learning. Despite its effectiveness in abstract neural networks, it remains unclear whether backprop might represent a viable implementation of cortical plasticity. Here, we present a new theoretical framework that uses a least-action principle to derive a biologically plausible implementation of backprop.

In our model, neuronal dynamics are derived as Euler-Lagrange equations of a scalar function (the Lagrangian). The resulting dynamics can be interpreted as those of multi-compartment neurons with apical and basal dendrites, coupled with a Hodgkin-Huxley-like activation mechanism allowing neurons to phase-advance their somatic input and hence undo temporal delays introduced by somatic and dendritic low-pass filtering. We suggest that a neuron’s apical potential encodes a local prediction error arising from the difference between top-down feedback from higher cortical areas and bottom-up predictions represented by activity in its home layer. This computation is enabled by a stereotypical cortical microcircuit, projecting from pyramidal neurons to interneurons back to the pyramidal neurons’ apical compartments. When a subset of output neurons is slightly nudged towards a target behavior that cannot be explained away by bottom-up predictions, an error signal is induced that propagates back throughout the network via feedback connections. By defining synaptic dynamics as gradient descent on the Lagrangian, we obtain a biologically plausible plasticity rule that acts on the forward projections of pyramidal neurons in order to reduce this error.

The presented model incorporates several features of biological neurons that cooperate towards approximating a time-continuous version of backprop, where plasticity acts at all times to reduce local prediction errors, thereby minimizing a global output error or cost function. Finally, the model is not only restricted to supervised learning, but can also be applied to unsupervised and reinforcement learning schemes, as demonstrated in simulations.

**Acknowledgments:** This work has received funding from the European Union under grant agreements 720270, 785907 (HBP) and the Manfred Stärk Foundation. Calculations were performed on UBELIX (University of Bern HPC) and bwHPC (state BaWü HPC, supported by the DFG through grant no INST 39/963-1 FUGG).

## P283 Spatiotemporal discrimination in attractor networks with short-term synaptic plasticity

### Benjamin Ballintyn^1^, Paul Miller^2^, Benjamin Shlaer^3^

#### ^1^Brandeis University, Graduate Program in Neuroscience, Waltham, MA, United States of America; ^2^Brandeis University, Dept of Biology, Waltham, MA, United States of America; ^3^University of Auckland, Physics, Auckland, New Zealand

##### **Correspondence:** Benjamin Ballintyn (bbal@brandeis.edu)

*BMC Neuroscience* 2019, **20(Suppl 1)**: P283

We demonstrate that a randomly connected attractor network with dynamic synapses can discriminate between similar general basis for neural computations in the brain. The network contains units representing assemblies of pools of neurons, with preferentially strong recurrent excitatory connections rendering each unit bi-stable. Weak interactions between units leads to a multiplicity of attractor states, within which information can persist beyond stimulus offset. When a new stimulus arrives, the prior state of the network impacts the encoding of the incoming information, with short-term synaptic depression ensuring an itinerancy between sets of active units. We assess the ability of such a network to encode the identity of sequences of stimuli, so as to provide a template for sequence recall, or decisions based on accumulation of evidence. Across a range of parameters, such networks produce the primacy (better final encoding of the earliest stimuli) and recency (better final encoding of the latest stimuli) observed in human recall data and can retain the information needed to make a binary choice based on total number of presentations of a specific stimulus. Similarities and differences in the final states of the network produced by different sequences lead to predictions of specific errors that could arise when an animal or human subject generalizes from training data, when the training data comprises a subset of the entire stimulus repertoire. We suggest that such networks can provide the general-purpose computational engines needed for us to solve many cognitive tasks.

## P284 Time-dependent dopamine modulation of projection neurons in the mosquito olfactory system

### Suh Woo Jung^1^, Eli Shlizerman^1,2^

#### ^1^University of Washington, Department of Electrical Engineering, Seattle, WA, United States of America; ^2^University of Washington, Departments of Applied Mathematics, Seattle, WA, United States of America

##### **Correspondence:** Eli Shlizerman (shlizee@uw.edu)

*BMC Neuroscience* 2019, **20(Suppl 1)**: P284

Mosquitoes are known for their tendency in selecting host species as certain individuals are preferred over the other. How do mosquitoes determine and alter their host preferences? One important factor that affects host selection is olfaction, and requires a key neurotransmitter, dopamine, that enforces learning [1].

The major goal of this study is to investigate how dopamine modulates the neuronal activity in Aedes aegypti mosquitoes. Superfusing dopamine on the olfactory center of the brain, the antennal lobe (AL), is known to modulate the activities of neurons and increase the ability to learn new hosts. In our work, we process the multi-neuronal recordings of the projection neurons (PNs) in the AL under three different phases: before (P1), during (Dop), and after (P2) the superfusion of dopamine. The firing-rates of PNs were recorded for six different odor stimuli for several trials in each of the phases.

The dynamics of PNs projected to lower dimensional odor space reveal converging to fixed points as in [2, 3]. Comparison of the locations of the fixed points in each of the phases indicates that the superfusion of dopamine causes fixed points to dislocate indicating that dopamine has longer-term effects on the AL neural encoding (Fig. A right). Furthermore, dislocation of the fixed point appears to be specific for each odor. In order to investigate how dopamine modulation could rearrange neural connectivity in the AL, we employ a network model which infers the connectome of neurons in the AL from recorded data [3]. In particular, the model that employs lateral inhibition where both inhibitory and excitatory neurons receive common input and interact to mediate the response of the PNs. We then further extend the neural dynamics model and propose a new learning rule to accommodate the roles of dopamine neurons.

In particular, we assume that the connectome matrices are time-invariant during the phase when no learning is occurring (P1) and use the representation of the fixed point in an orthogonal odor space to calibrate the connectivity matrices [3, 4]. We then examine recordings in the Dop phase and re-calibrate the inhibition matrix B while keeping the other matrices fixed. The calibration is using the new dynamics to the fixed point in the Dop phase as the target. Specifically, we enforce a learning rule that at each time point takes the difference between P1 and Dop dynamics and solves a convex optimization problem for the elements of B. We observe that modulation of the elements of B oscillate during and after the stimulus in the Dop phase with the most salient burst observed between 500ms and 600ms These oscillating patterns are consistent throughout all neurons, which suggests that superfusion of dopamine has global impact on the neural population.

**References**

1. Vinauger C, Lahondère C, Wolff GH, et al. Modulation of host learning in Aedes aegypti mosquitoes. *Current Biology* 2018 Feb 5;28(3):333–44.
2. Riffell JA, Shlizerman E, Sanders E, et al. Flower discrimination by pollinators in a dynamic chemical environment. *Science* 2014 Jun 27;344(6191):1515–8.
3. Shlizerman E, Riffell JA, Kutz JN. Data-driven inference of network connectivity for modeling the dynamics of neural codes in the insect antennal lobe. *Frontiers in computational neuroscience* 2014 Aug 13;8:70.
4. Blaszka D, Sanders E, Riffell JA, Shlizerman E. Classification of fixed point network dynamics from multiple node timeseries data. *Frontiers in neuroinformatics* 2017 Sep 20;11:58.

## P285 Identifying functional pathways of oxygen sensation in caenorhabditis elegans using systematic computational ablation

### Jimin Kim^1^, Eli Shlizerman^1,2^

#### ^1^University of Washington, Electrical and Computer Engineering, Seattle, WA, United States of America; ^2^University of Washington, Applied Mathematics, Seattle, WA, United States of America

##### **Correspondence:** Jimin Kim (jk55@u.washington.edu)

*BMC Neuroscience* 2019, **20(Suppl 1)**: P285

Caenorhabditis elegans (C. elegans) is known to perform aerotaxis, behavior in which locomotion is modulated according to the composition of air in the environment. Sensory neurons related to O2aerotaxis have been proposed through experimental research [1]. In particular, it was shown that the sensory AQR neuron, located in the anterior part of the body, is a possible trigger for changing oxygen level. Here we use a recently introduced in-silico model, which emulates nervous system responses to stimuli [2] and simulates the body to investigate functional pathways of O2sensation [3]. Our approach is to introduce neural stimuli continuously varying according to the location of the body. Specifically, we develop spatial gradients of AQR stimulation proportional to the level of O2concentration. We utilize such stimuli to search for downstream neurons which play a key role in mediating O2aerotaxis response.

We first validate that when AQR gradient is placed as an obstacle for forward locomotion the stimulus changes the forward bearing causingC. elegansto steer away from the gradient, as observed in experiments (see Fig. 1). Consequently, we propose a computational ablation approach for examination and testing of the effective functionality of sensorimotor neural pathways, such as pathways from AQR to motor neurons. In particular, we propose aCombinatorial Neighbor Ablation (CNA) algorithm, in which all possible combinations of neurons connected via synaptic or gap connection to a set of neurons is enlisted and ablation is performed for each combination. We identify 64 of such combinations for the AQR neuron. Application of CNA to the AQR neuron allows us to sort pathways into strongly or loosely correlated with AQR response. Indeed, the analysis identifies a command neuron, PVPL as an interneuron that has the highest correlation with AQR induced oxygen sensation, i.e., its ablation masks motor response driven by oxygen sensation functionality. In addition, we identify alternative sensorimotor pathways from AQR which include neurons (PVPR, DVC, AVKL). Upon triple ablation of these neurons, the ventral turning inside the AQR gradient is significantly diminished.

**Fig. 1:**
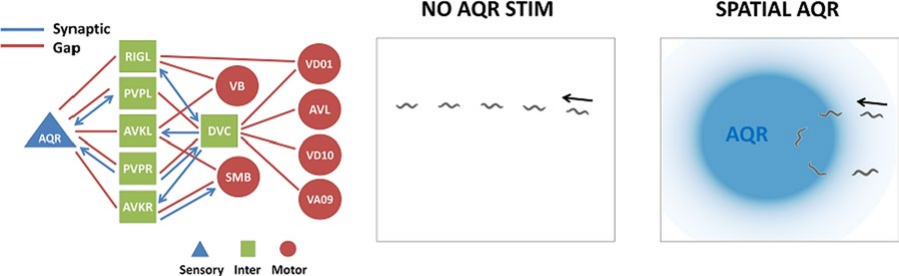
Left: Diagram of O2 sensation circuit induced by AQR sensory neuron compiled from connectome and circuitry identifies from vulnerability analysis of Kim et al [4]. Middle: Snapshots of baseline forward locomotion. Right: Snapshots of forward locomotion with the presence of a spatial AQR gradient

With this study we show that *C. elegans* in-silico model in conjunction with spatial stimulation and systematic ablation can assist in identifying behavioral functional pathways and circuits, even in a complex model that incorporates connectomics, dynamics and body postures, exhibiting intricate behaviors such as aerotaxis.

**References**

1. Gray JM, Karow DS, Lu H, et al. Oxygen sensation and social feeding mediated by a C. elegans guanylate cyclase homologue. *Nature* 2004 Jul;430(6997):317.
2. Kim J, Leahy W, Shlizerman E. Neural interactome: Interactive simulation of a neuronal system*. Frontiers in Computational Neuroscience* 2019;13.
3. Kim J, Shlizerman E. Dynamic worm: Computational investigation of locomotion through integration of connectomics, neural dynamics and biomechanics in caenorhabditis elegans. *Submitted*
4. Kim S, Kim H, Kralik JD, Jeong J. Vulnerability-based critical neurons, synapses, and pathways in the Caenorhabditis elegans connectome. *PLoS computational biology* 2016 Aug 19;12(8):e1005084.

## P286 A circuit model for temporal sequence learning

### Ian Cone^1^, Harel Shouval^2^

#### ^1^Rice University, Applied Physics, Houston, TX, United States of America; ^2^UTHealth, Neurobiology and Anatomy, Houston, United States of America

##### **Correspondence:** Ian Cone (iancone@rice.edu)

*BMC Neuroscience* 2019, **20(Suppl 1)**: P286

Sequence representation is an essential part of many kinds of learning and memory, and as such there may be common design principles which describe the circuits that mediate it. My work proposes a substrate for such representations, via a biophysically realistic network model that can robustly learn and recall sequences of variable order and duration. This work is in agreement with recent experimental results, which have shown that visual temporal sequence representations may be stored and recalled by local neural circuits in visual cortex. While this model is designed specifically to account for these observations in V1, it can also be thought of as a general circuit model for sequence representation, regardless of cortical modality.

The network consists of spiking leaky-integrate-and-fire model neurons placed in a modular architecture designed to mimic cortical microcolumns. The network is stimulated from an input layer designed to mimic LGN inputs. Learning is performed via competitive LTP and LTD like “eligibility traces”, which hold a history of synaptic activity before being converted into changes in synaptic strength upon the presentation of reward. This short term synaptic history solves the temporal credit assignment problem that arises from traditional Hebbian rules. A recent study has found evidence that these eligibility traces indeed exist and are consistent with the theoretically proposed mechanism that can be used to associate distal events.

Before training, the network only produces naïve responses to incoming stimuli, and contains no memory of any particular sequence. During training, a particular temporal sequence of visual stimuli is repeatedly presented to the network. After training, presentation of only the first element in that sequence is sufficient for the network to recall its entire learned representation of the sequence (Fig. 1). This capability provides a sufficient framework for biologically realistic sequence based learning and memory.

**Fig. 1:**
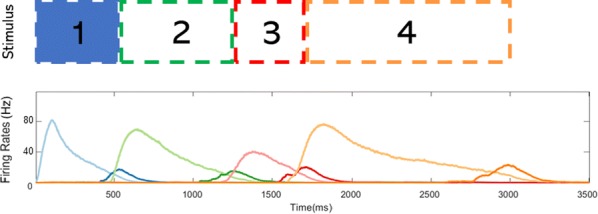
Top, representation of stimuli presented during training (dotted boxes) and after training (solid boxes). Bottom, time averaged firing rates of neuronal subpopulations in response to only stimulus 1 (after training). The network recalls the entire learned sequence 1-2-3-4

## P287 Exploring interneuron specific control of oriens lacunosum moleculare (OLM) interneuron recruitment in CA1 hippocampus

### Alexandre Guet-McCreight, Frances Skinner

#### Krembil Research Institute, Division of Fundamental Neurobiology, Toronto, Canada

##### **Correspondence:** Alexandre Guet-McCreight (agmccrei@gmail.com)

*BMC Neuroscience* 2019, **20(Suppl 1)**: P287

Consideration of interneuron subtypes is critical to understanding brain function and behavior [4]. However, deciphering the contributions of intrinsic cell properties and cell firing to network population output and behavior is extremely challenging given the large diversity of cell types, connectivity rules, and ion channels present in neurons. Certain interneuron subtypes synapse only onto other interneurons, exerting disinhibitory control over circuits [1]. One such example is work from [6], where photoactivation of interneuron specific 3 (IS3) cells in CA1 hippocampus at a 10 Hz frequency was sufficient to control the spiking of oriens lacunosum moleculare (OLM) cells *in vitro*. This frequency is notable since it is in the theta frequency range, a brain rhythm important in memory [2], which OLM cells are known to be phase-locked to *in vivo*. It remains unclear whether this finding is due to direct connections from IS3 cells, resultant activity from other interneuron types in CA1 (e.g. feedforward inhibition/excitation), or contributions of post-inhibitory rebound (PIR)-related currents in OLM cells. To explore this question computationally, we first used BluePyOpt [7] and the neuroscience gateway [5], to develop new, spiking multi-compartment models of OLM cells that were constrained by morphologies and electrophysiologies from the same cell. We then used these models to simulate the *in vitro* conditions in [6]. For this we chose three different presynaptic populations to simulate a virtual network on our OLM cell models: IS3 cells, bistratified cells, and local pyramidal cells. Synaptic conductance for each compartment in the model were fit to approximately match the current amplitudes seen experimentally for these inputs to OLM cells. We first tested the effect of IS3 cell inputs alone on the OLM cell models spiking across a range of frequencies and numbers of synapses. While IS3 cell inputs alone could time OLM cell spiking, this was preferentially at lower spike frequencies with larger numbers of synapses. We also did not observe any substantial contributions of PIR-currents relative to other intrinsic properties in the OLM cell models, suggesting that OLM cell spiking was timed through spike suppression rather than PIR. We next looked at the effects of adding feedforward inhibitory inputs, which generated similar results to having IS3 cell inputs alone. Given these results, we also added feedforward excitatory inputs, and found much more stereotyped spiking in the OLM cell models, similar to the experimental results obtained by [6]. Thus, our simulations lead us to predict that *in vitro* photoactivation of IS3 cells causes a feedforward disinhibition of pyramidal cell spiking and recurrent excitation onto OLM cells, which leads to more phase-locked OLM cell spiking. Our computational studies help untangle interacting inhibitory and excitatory interactions controlling the various cell subtypes. Moving forward, these interactions can be examined in *in vivo*-like scenarios [3].

**References**

1. Chamberland S, Topolnik L. Inhibitory control of hippocampal inhibitory neurons. *Frontiers in neuroscience* 2012 Nov 14;6:165.
2. Colgin LL. Rhythms of the hippocampal network. *Nature Reviews Neuroscience* 2016 Apr;17(4):239.
3. Guet-McCreight A, Skinner FK. Using computational models to predict in vivo synaptic inputs to interneuron specific 3 (IS3) cells of CA1 hippocampus that also allow their recruitment during rhythmic states. *PloS one* 2019 Jan 8;14(1):e0209429.
4. Kepecs A, Fishell G. Interneuron cell types are fit to function. *Nature* 2014 Jan;505(7483):318.
5. Sivagnanam S, et al. Introducing the Neuroscience Gateway 2013 IWSG, volume 993 of CEUR Workshop *Proceedings. CEUR-WS. org*. 2013.
6. Tyan L, et al. Dendritic inhibition provided by interneuron-specific cells controls the firing rate and timing of the hippocampal feedback inhibitory circuitry. *Journal of Neuroscience* 2014 Mar 26;34(13):4534–47.
7. Van Geit W, Gevaert M, Chindemi G, et al. BluePyOpt: leveraging open source software and cloud infrastructure to optimise model parameters in neuroscience. *Frontiers in neuroinformatics* 2016 Jun 7;10:17.

## P288 Shaping connectivity and dynamics of neuronal networks with physical constraints

### Adriaan Ludl, Jordi Soriano

#### Universitat de Barcelona, Departament de Física de la Matèria Condensada, UB Institute of Complex Systems, Barcelona, Spain

##### **Correspondence:** Adriaan Ludl (ludl@ub.edu)

*BMC Neuroscience* 2019, **20(Suppl 1)**: P288

Scaffolds and patterned substrates are among the most successful devices to tailor the connectivity between neurons in culture. We compare simulations of networks with known ground truth topology to inferred functional networks from experimental data obtained by calcium fluorescence imaging of *in vitro* cultures.

We model axonal growth as a random walk where connections are established with a given probability where an axon crosses the dendritic tree of another neuron as in [1]. Physical obstacles have been observed to guide the growth of neurites in experiments [2], axons tend to follow rounded obstacles.

The impact of obstacles and their shape on connectivity of the network is examined. Fig. 1 illustrates that box like scaffolds reduce the in-degree and enhance the clustering coefficient. The effect of an array of obstacles is discussed and the results are compared to those obtained for cultures of varying density [3].

**Fig. 1:**
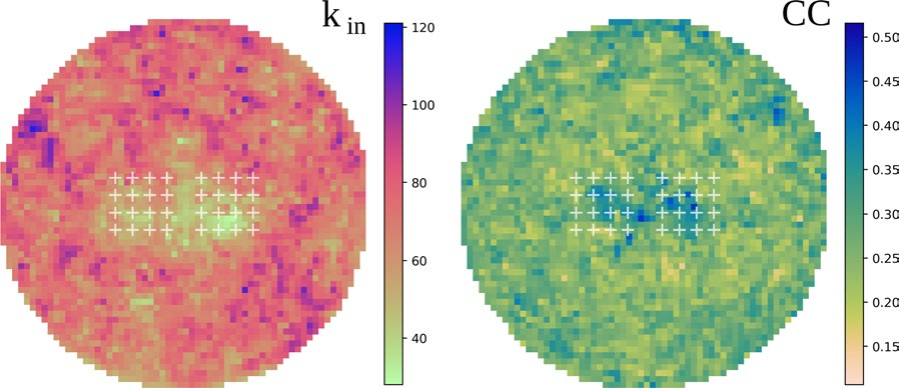
Heatmaps of in-degree (left) and clustering coefficient (right) for a simulation of 2500 neurons in a circle of radius 2.0 mm with two 4x4 scaffold arrays

**Acknowledgements**: This research is part of MESOBRAIN. MESOBRAIN has received funding from the European Union’s Horizon 2020 research and innovation programme under grant agreement No 713140. [4]

**References**

1. Orlandi JG, Soriano J, Alvarez-Lacalle E, et al. Noise focusing and the emergence of coherent activity in neuronal cultures. *Nature Physics* 2013, 9(9), p.582–590.
2. Tibau E, Bendiksen C, Teller S, et al. Interplay activity-connectivity: dynamics in patterned neuronal cultures. *In AIP Conference Proceedings* 2013, 1510(1), p. 54–63.
3. Martorell ET, Ludl AA, Rudiger S, et al. Neuronal spatial arrangement shapes effective connectivity traits of in vitro cortical networks. *IEEE Transactions on Network Science and Engineering* 2018, 10.1109/tnse.2018.2862919.
4. The MesoBrain Project, http://www.mesobrain.eu.

## P289 Application of control theory to neural learning in the brain

### Catherine Davey^1^, David Grayden^1^, Anthony Burkitt^1^, Bastian Oetemo^2^, Artemio Soto-Breceda^1^

#### ^1^University of Melbourne, Department of Biomedical Engineering, Melbourne, Australia; ^2^University of Melbourne, School of Computing and Information Systems, Melbourne, Australia

##### **Correspondence:** Catherine Davey (cedavey@unimelb.edu.au)

*BMC Neuroscience* 2019, **20(Suppl 1)**: P289

Neural plasticity describes the process by which the brain learns, primarily in response to environmental inputs. Supervised multisensory learning in the biological context can be considered a subset of plasticity that describes how one sensory system trains a second sensory modality to achieve a specific goal [1]. This sensory integration requires multimodal neurons and is often performed in higher processing areas. Consequently, while there are several small-scale examples of supervised learning, more general cases require a complex system of interconnected neurons from multiple brain regions [2]. Supervised learning in artificial neural networks has historically been modelled using iterative gradient evaluation techniques [3], typically using backpropagation of error through the network to enable local updating of synaptic connection strengths. In biological neural systems, this assumes that the network can propagate the error backwards, which is a significant assumption that is not biologically plausible at the level of individual synapses [4].

In this work, we pose supervised learning within a control framework, with the primary objective to capitalise on the success of control theory in managing large scale, complex systems [5]. We design and build a biologically plausible supervisory system that is scalable in both size and complexity. Feedback control, in particular, has many desirable properties, such as the ability to converge to a specified output, stable performance in a noisy environment, and a framework for modelling complex systems [6]. We develop a proof-of-concept for the use of control theory in supervised multi-modal learning, showing how the visual system can ‘teach’ the auditory system to identify the direction of a sound source, demonstrating superior performance to existing techniques. Our prototype system learns to transform the interaural time difference (ITD) (delay between the arrival of a sound to the left and right ears [7]) into an estimate of angle to the source. The auditory feature map generated from the ITD is transformed to a source angle feature map in the superior colliculus, though exactly how this is achieved is the subject of ongoing research.

The primary challenge in employing control theory for biological neural networks is in the calculation of the control signal. In a control system, the controller calculates the optimal signal to be fed into the system to minimise the difference between the system’s output and the supervisor’s output. The controller is limited in the operations available to it to ensure it is feasibly implemented in a neural network. In this work, we show that it is possible to design a controller that complies with the requirements of the control system as well as being biologically feasible. Application of control theory has the potential to resolve complexity limitations inherent in current approaches in addition to addressing the biological plausibility issues associated with current techniques.

**Fig. 1:**
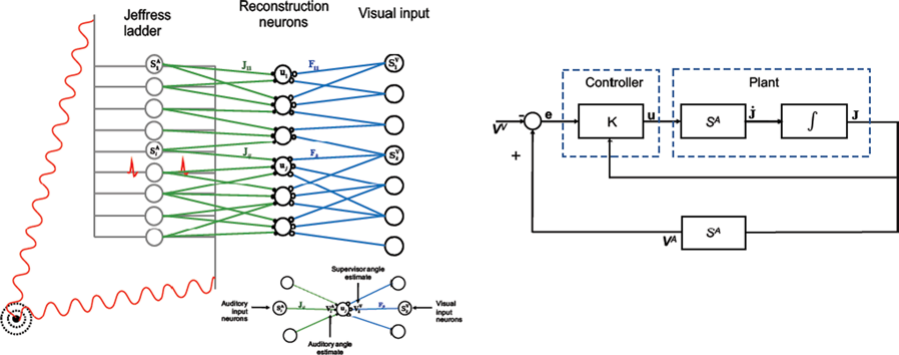
Schematic showing the integration of auditory angle estimate from a Jeffress ladder, with visual angle estimate, in a reconstruction neuron. The reference signal from the visual system, V^V, is combined with the sum of the output of the Jeffress ladder, S^A, weighted by synaptic weights, J. Update of the weights is determined by the controller, which calculates an optimal gain signal, K

## P290 From episodic to semantic memory: A computational model

### Denis Alevi^1^, Richard Kempter^2^, Henning Sprekeler^1^

#### ^1^Technical University Berlin, Berlin, Germany; ^2^Humboldt University of Berlin, Institute for Theoretical Biology, Berlin, Germany

##### **Correspondence:** Denis Alevi (denis.alevi@campus.tu-berlin.de)

*BMC Neuroscience* 2019, **20(Suppl 1)**: P290

Systems memory consolidation transfers declarative memories that initially depend on the hippocampal formation into long-term memory traces in neocortical networks. Over the last decades, multiple phenomenological theories of systems memory consolidation have been proposed. While it appears clear that episodic memories undergo a semantization over time, no consensus on why and how this arises has been found as of now, partially because the mechanistic basis for systems consolidation on the level of neurons and synapses is largely unresolved.

Here, we study how episodic memories change over time in a recently suggested computational model for the neuronal basis of systems memory consolidation. The model suggests that systems memory consolidation could arise from Hebbian synaptic plasticity in networks with parallel synaptic pathways (Fig. 1). In the model, memories are initially stored as cue-response associations in a multisynaptic pathway. During consolidation, these associations are reactivated and allow the multisynaptic pathway to act as a teacher for a shortcut pathway. This transfer into shortcut pathways—which are widely encountered throughout the brain—can be hierarchically iterated to achieve a transfer of memories from the hippocampus to neocortex.

**Fig. 1:**
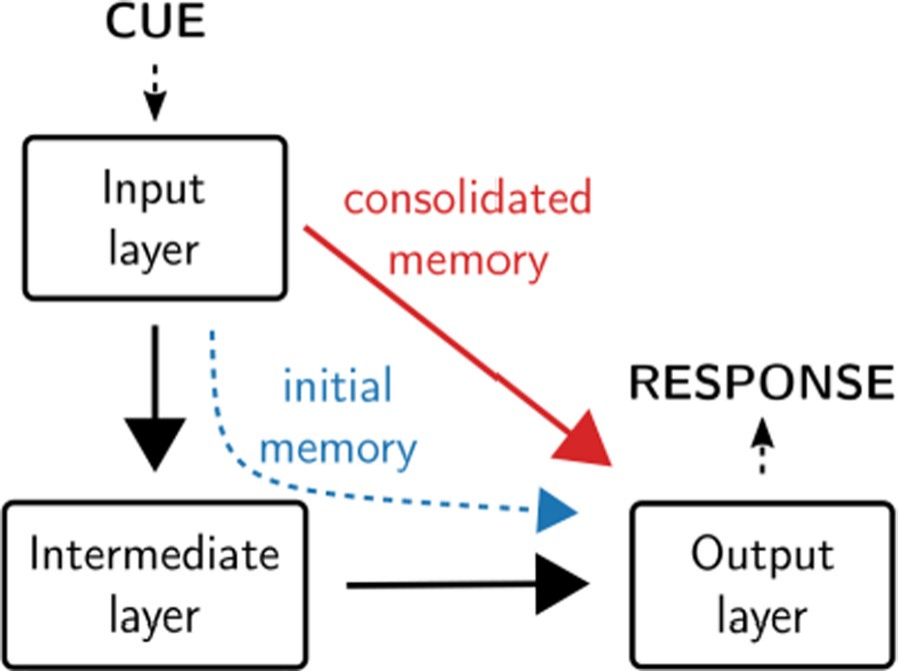
Schematic of the network motif consisting of two parallel synaptic pathways. Memories, conceptualized as cue-response associations, are initially stored in a multisynaptic pathway (blue) and later consolidated into a parallel shortcut connection (red)

In the present work, we implement the proposed mechanism in artificial neural networks to study how the characteristics of episodic memories change over time. Given that episodic memories contain details of individual autobiographic events, we conceptualize the formation of an episodic memory in neural networks as overfitting to a single event—thereby learning all of its details. Semantic memory on the other hand is abstract and factual knowledge that allows to generalize across experiences and is hence largely independent of any specific event. To model memory consolidation, we simulate the acquisition of new episodic memories during the day by storing their associations in the multisynaptic pathway of the shortcut mechanism. Consolidation during the night is then modelled by reactivating these associations and thereby learning them in the parallel shortcut pathway. Memories are therefore transferred from a multi-layer neural network into a two-layer neural network, and hence from a higher capacity learning system to a lower capacity learning system. We show that in such a model, the transfer of memory associations into the shortcut pathway facilitates the forgetting of random episodic detail in memories and enhances the extraction of semantic generalizations. Moreover, we show that (i) the amount of episodic detail that is transferred into the shortcut pathway depends on the speed of learning in the shortcut pathway and (ii) that neural replay enhances the speed of consolidation and can in certain situations be necessary for the extraction of semantic memories. The latter appears to be the case specifically for the extraction of semantic content from a rapidly learning hippocampal system. Finally, we hypothesize that the previously suggested hierarchical iteration of the mechanism may provide a mechanistic model for the spatial and temporal gradients of episodic and semantic memories observed in lesion studies, which suggest that episodic memory content decreases and semantic memory content increases with distance from the hippocampus.

## P291 Short-term facilitation and neurotransmitter spillover counteract each other in neuronal information transmission

### Mehrdad Salmasi^1,2,3^, Stefan Glasauer^2,3,5^, Martin Stemmler^2,4^

#### ^1^Graduate School of Systemic Neurosciences, Ludwig-Maximilians-Universität München, Munich, Germany; ^2^Bernstein Center for Computational Neuroscience, Munich, Germany; ^3^German Center for Vertigo and Balance Disorders, Ludwig-Maximilians-Universität, Munich, Germany; ^4^Department of Biology II, Ludwig-Maximilians-Universität, Munich, Germany; ^5^Chair of Computational Neuroscience, Brandenburg University of Technology Cottbus-Senftenberg, Senftenberg, Germany

##### **Correspondence:** Mehrdad Salmasi (mehrdad.salmasi@lrz.uni-muenchen.de)

*BMC Neuroscience* 2019, **20(Suppl 1)**: P291

Chemical synapses mediate neuronal information transmission through the release of vesicles. The neurotransmitter molecules of a released vesicle diffuse in the synaptic cleft and attach to the postsynaptic receptors located opposite to the release site. Through diffusion, the neurotransmitters can activate the receptors of the neighboring release sites, a phenomenon known as neurotransmitter spillover [1]. Such spillover causes the number of open receptor channels to rise faster than linearly with the number of vesicles released, thereby altering synaptic integration. How the nonlinear transformations of the postsynaptic potential affect synaptic information efficacy has not been fully understood yet.

We present an analytical model of spillover in excitatory and inhibitory synapses, and derive the postsynaptic potential as a function of the number of released vesicles for different levels of spillover (Fig. 1A). Each release site is modeled by a binary asymmetric channel which distinguishes between synchronous spike-evoked release and asynchronous release of the synapse. The short-term plasticity of the synapse is incorporated into the memory of the communication channel; the release probability of the synapse is determined based on the history of vesicular release and the spiking activity of the presynaptic neuron [2, 3].

**Fig. 1:**
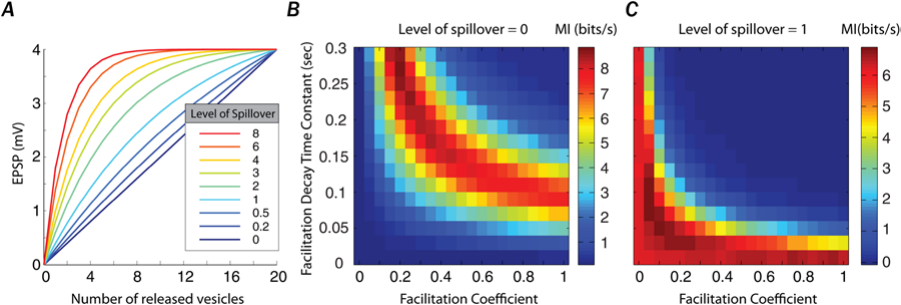
**a** Excitatory postsynaptic potential depending on the number of released vesicles for various levels of spillover. **b** The mutual information rate, MI, between an input neuron and the target neuron as a function of facilitation dynamics in the absence of spillover. **c** Similar to **b** for synapses with spillover

We have shown that neurotransmitter spillover changes the functional role of short-term depression. In the presence of spillover, short-term depression can increase the rate of information transmission between the neurons [4]. Here we study the interplay between short-term synaptic facilitation and neurotransmitter spillover and its modulatory effect on information transmission.

Short-term facilitation increases the release probability of both synchronous spike-evoked release and asynchronous release, which represent two distinct modes of release regulated by different synaptotagmins. We first identify the regime of facilitation dynamics for which neurons (without spillover) transfer information optimally (Fig. 1B). Interestingly, both spillover and facilitation can separately improve synaptic information transmission, but asynchronous release limits the benefits of having both. Indeed, in the presence of spillover, the optimal rate of information transmission is attained for facilitation that is weaker and decays faster (Fig. 1C). Many synapses manifest not only short-term facilitation, but also short-term depression on slightly longer time-scales. We determine the optimal joint dynamics of depression and facilitation that results in the maximum rate of information transmission between the neurons, and investigate the dependency of the optimal regime on the level of spillover.

**Acknowledgment:** Supported by DSGZ (BMBF grant 01EO1401).

**References**

1. DiGregorio DA, Nusser Z, Silver RA. Spillover of glutamate onto synaptic AMPA receptors enhances fast transmission at a cerebellar synapse. *Neuron* 2002.
2. Salmasi M, Stemmler M, Glasauer S, Loebel A. Information rate analysis of a synaptic release site using a two-state model of short-term depression. *Neural Computation* 2017.
3. Salmasi M, Loebel A, Glasauer S, Stemmler M. Short-term synaptic depression can increase the rate of information transfer at a release site. *PLoS Computational Biology* 2019.
4. Salmasi M, Glasauer S, Stemmler M. Neurotransmitter spillover redresses the information loss caused by synaptic depression. *Abstract for Cosyne* 2019.

## P292 Reconstructing connectome of the cortical column with biologically-constrained associative learning

### Danke Zhang, Chi Zhang, Armen Stepanyants

#### Northeastern University, Department of Physics, Boston, MA, United States of America

##### **Correspondence:** Danke Zhang (d.zhang@northeastern.edu)

*BMC Neuroscience* 2019, **20(Suppl 1)**: P292

Cortical connectome develops in an experience-dependent manner under the constraints imposed by the morphologies of axonal and dendritic arbors of numerous classes of neurons. In this study, we describe a theoretical framework which makes it possible to construct the connectome of the cortical column by loading associative memory sequences into its structurally (potentially) connected network [1]. To generate the structural connectivity of the column, we put together axonal and dendritic arbors of 55 neuron classes reconstructed as part of the Blue Brain project [2] and created a network containing 28,156 neurons interconnected with 1.9 × 108 potential synapses [3]. By loading associative memory sequences into this network [4, 5], we generated its functional connectivity. Learning in the model is accompanied with biologically inspired constraints imposed by structural connectivity, constraints on connection signs (excitatory or inhibitory), homeostatic constraints on connection weights, and the requirement of reliable memory storage [4, 5]. We solved the associative learning problem analytically (replica theory) and numerically, showing that at close to maximum memory storage capacity many properties of connectivity in the model column are in good agreement with the available experimental measurements. These include connection probabilities for 14 types of local excitatory and inhibitory projections, dependence of connection probability on distance between neurons, correlations between structural and functional connectivity, overrepresentations of specific excitatory and inhibitory 3-neuron motifs, and volume densities of inhibitory synapses in different cortical layers (see Fig. 1). Our results contain predictions regarding intra- and inter-laminar connectivity between specific neuron classes that can be tested in future experiments. We conclude that basic properties of the connectome of the cortical column may have resulted from biologically-constrained associative learning in a morphologically constrained neural network.

**Fig. 1:**
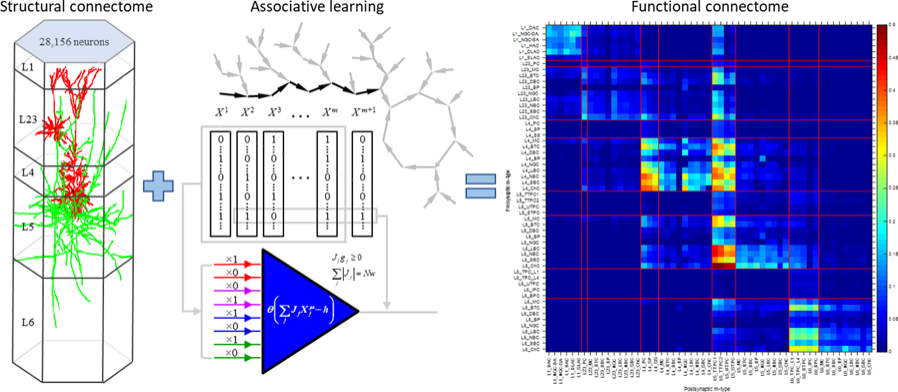
Functional connectivity results from biologically-constrained associative learning in a morphologically constrained neural network

**Acknowledgements:** This work is supported by Air Force grant FA9550-15-1-0398 and NSF grant IIS-1526642.

**References**

1. Markram H, Muller E, Ramaswamy S, et al. Reconstruction and simulation of neocortical microcircuitry. *Cell* 2015 Oct 8;163(2):456–92.
2. The Blue Brain Project, 2019. https://bbp.epfl.ch/nmc-portal/welcome
3. Stepanyants A, Hirsch JA, Martinez LM, Kisvárday ZF, Ferecskó AS, Chklovskii DB. Local potential connectivity in cat primary visual cortex. *Cerebral Cortex* 2007 Apr 9;18(1):13–28.
4. Brunel N. Is cortical connectivity optimized for storing information? *Nature Neuroscience* 2016 May;19(5):749.
5. Zhang D, Zhang C, Stepanyants A. Robust associative learning is sufficient to explain structural and dynamical properties of local cortical circuits. *bioRxiv* 2018 Jan 1:320432.

## P293 Phase dependence of the termination of absence seizures by cerebellar input to thalamocortical networks

### Julia Goncharenko^1^, Lieke Kros^2^, Reinoud Maex^1^, Neil Davey^1^, Christoph Metzner^3^, Chris de Zeeuw^2^, Freek Hoebeek^2^, Volker Steuber^1^

#### ^1^University of Hertfordshire, Biocomputation Research Group, Hatfield, United Kingdom; ^2^Erasmus MC, Department of Neuroscience, Rotterdam, Netherlands; ^3^Technische Universität Berlin, Department of Software Engineering and Theoretical Computer Science, Berlin, Germany

##### **Correspondence:** Julia Goncharenko (i.goncharenko@herts.ac.uk)

*BMC Neuroscience* 2019, **20(Suppl 1)**: P293

Absence seizures are the most common form of epilepsy in children. They start and finish abruptly, last for 10–20 seconds and can be detected by generalised spike-and-wave discharges (GSWDs) in the electroencephalogram. These GSWDs are based on neuronal oscillations in thalamocortical networks, which can be caused by excessive inhibition in the thalamus or excessive cortical activity. Absence seizures can be triggered by switching of the normal asynchronous neuronal activity in thalamocortical networks to synchronised oscillations, and terminated by the reverse process, switching from synchronised oscillations to asynchronous activity.

Experimental studies have shown that thalamic stimulation can disrupt oscillatory activity in thalamocortical networks. More recently, it was also found that optogenetic activation of neurons in the cerebellar nuclei (CN) can stop epileptic absence seizures and reset the oscillatory activity, for example in a closed loop system [1]. However, the underlying mechanism of the termination of absence seizures by CN stimulation is not yet clear.

To investigate the mechanism of the termination of absence seizures by thalamic input from the CN we used computer simulations. We simulate a thalamocortical network model with adaptive exponential integrate-and-fire neurons, displaying complex intrinsic properties such as low-threshold spiking, regular spiking, fast spiking and adaptation [2]. The network activity can exhibit oscillatory or asynchronous irregular (AI) dynamics, depending on the time constants of the inhibitory synaptic conductance, which are 5 ms (AI) and 15 ms (oscillatory), respectively. An increase in the inhibitory decay time constant reflects a change from GABAA dominated inhibition to more GABAB, which can result in GSWDs, given that the “wave” components of GSWDs are related to slow GABAB -mediated K+ currents [3].

We provide CN input to all thalamocortical neurons to analyse the mechanism of reverting from abnormal oscillatory activity to the normal AI state. Our results confirm that input from the CN can control oscillatory activity in thalamocortical networks. Furthermore, they show that the effectiveness of this input exhibits phase-dependence. In our simulations, CN input terminates epileptic absence seizures most effectively when it arrives at the peak of GSWDs, while seizure termination is least efficient for input between the GSWD bursts. This finding is potentially relevant for therapeutic applications of CN stimulation to terminate seizures. However, the simulations in silico did not take several biological factors such as indirect pathways from the CN to the thalamus into account that may explain differences with animal models of epilepsy [1].

**References**

1. Kros L, Eelkman Roda OHJ, Spanke JK, et al. Cerebellar Output Controls Generalised Spike-and-Wave Discharge Occurrence. *Annals of neurology* 2015, 77(6):1027–1049.
2. Destexhe A. Self-sustained asynchronous irregular states and up-down states in thalamic, cortical and thalamocortical networks of nonlinear integrate-and-fire neurons. *Journal of Computational Neuroscience* 2009, 27:493–506.
3. Destexhe A. Spike-and-Wave Oscillations Based on the Properties of GABAB Receptors. *Journal of Neuroscience* 1998, 18(21):9099–9110.

## P294 Growth rules for repair of asynchronous irregular network models following peripheral lesions

### Ankur Sinha^1^, Christoph Metzner^2^, Neil Davey^1^, Rod Adams^1^, Michael Schmuker^1^, Volker Steuber^1^

#### ^1^University of Hertfordshire, Biocomputation Research Group, Hatfield, United Kingdom; ^2^Technische Universität Berlin, Department of Software Engineering and Theoretical Computer Science, Berlin, Germany

##### **Correspondence:** Ankur Sinha (a.sinha2@herts.ac.uk)

*BMC Neuroscience* 2019, **20(Suppl 1)**: P294

Several homeostatic mechanisms enable the brain to maintain desired levels of neuronal activity after disruptive changes to synaptic inputs. One of these homeostatic mechanisms, structural plasticity, can restore activity levels after peripheral lesions and deafferentation by altering neuronal connectivity over extended time periods [2]. Several experimental lesion studies have investigated the temporal evolution of network rewiring by structural plasticity in detail. However, the underlying mechanisms and growth rules that underlie these homeostatic rewiring processes are still not known [3].

We have used computer simulations of a network model that exhibits biologically realistic Asynchronous Irregular (AI) activity [1] in order to study the growth rules and processes that could explain homeostatic rewiring based on structural plasticity after peripheral lesions. In our simulations, we observe network rewiring after loss of peripheral input to a localised part of the network, the Lesion Projection Zone (LPZ).

Our simulation results indicate that the homeostatic re-establishment of activity in neurons both within and outside the LPZ requires opposite activity dependent growth rules for excitatory and inhibitory post-synaptic elements. As a consequence, the reduction of activity in the LPZ results in ingrowth of novel excitatory inputs and a retraction of inhibitory input connections, whilst an increase in activity due to the loss of inhibition outside the LPZ causes a retraction of excitatory input connections and an increase in inhibitory ones. Our growth rules maintain desired activity levels in the network as well as in individual neurons.

Furthermore, we show that these growth rules replicate the directional formation of connections that is observed in lesion experiments. After deafferentation of the LPZ, the simulated network exhibits the sprouting of excitatory axons from areas next to the LPZ into the LPZ that has been reported in experiments, and the outgrowth of inhibitory axons from the LPZ into neighbouring areas that has been found experimentally. Further predictions of our model that could be tested experimentally are (1) that the ingrowth of excitatory axons into the LPZ requires that the growth of excitatory axons is triggered by an increase in neuronal activity, and (2) that the sprouting of inhibitory axons needs to be caused by a decrease in neuronal activity.

**References**

1. Vogels TP, Sprekele, H, Zenke F, Clopath C, Gerstner W. Inhibitory plasticity balances excitation and inhibition in sensory pathways and memory networks. *Science* 2011, 334, 1569–1573.
2. Butz M, van Ooyen A. A Simple Rule for Dendritic Spine and Axonal Bouton Formation Can Account for Cortical Reorganization after Focal Retinal Lesions. *PLoS Computational Biology* 2013, 9, e1003259.
3. Sammons RP, Keck T. Adult plasticity and cortical reorganization after peripheral lesions. *Current Opinion in Neurobiology 35. Circuit plasticity and memory*, 2015, 136–141. ISSN: 0959-4388.

## P295 The effect of alterations of schizophrenia-associated genes on gamma band auditory steady-state responses

### Christoph Metzner^1^, Gili Karni^1^, Hana McMahon-Cole^1^, Tuomo Mäki-Marttunen^2^, Volker Steuber^3^

#### ^1^Technische Universität Berlin, Department of Software Engineering and Theoretical Computer Science, Berlin, Germany; ^2^Simula Research Laboratory, Oslo, Norway; ^3^University of Hertfordshire, Biocomputation Research Group, Hatfield, United Kingdom

##### **Correspondence:** Christoph Metzner (cmetzner@ni.tu-berlin.de)

*BMC Neuroscience* 2019, **20(Suppl 1)**: P295

Recent GWAS have identified more than 100 risk genes for schizophrenia (SCZ) [1]. Many of these encode ion channels. While their function has been well characterized, the contributions of common variation in these channels to SCZ pathology remain elusive. Here, we explored the effects of altered kinetics of voltage-gated ion channels on gamma range auditory steady-state (ASSR) deficits, a common biomarker for SCZ [2]. We used a network model of coupled E and I neurons [3].We modified the parameters of single cells in a way that mimics the expected effects of common variants associated with SCZ [4, 5].We included a total of 86 variants of the following genes: CACNA1C, CACNA1D, CACNB2, SCN1A, and HCN1 [5]. We then simulated a click train paradigm with stimulation at 40 Hz, to investigate gamma ASSR deficits. Overall, not surprisingly, we found that almost all genetic variants had a small effect on gamma power (72/86 had gamma power change<15%). However, we identified few variants that either strongly reduced or strongly increased gamma power. Interestingly, these were exclusively variants of genes encoding Ca currents subunits. Furthermore, the variants resulting in reduced gamma power also produced a strong component in the theta range. These changes in spectral composition were caused by changes in the offset and the slope of parameter ‘m’ of the high-voltage activated Ca channel. Our results deepen the understanding of gamma range ASSR deficits in patients suffering from SCZ. All scripts will be freely available (https://github.com/ChristophMetzner) and integrated into the ASSRUnit software package [6].

**References**

1. Ripke S, Neale BM, Corvin A, Walters JT, et al. Biological insights from 108 schizophrenia-associated genetic loci. *Nature* 2014, 511(7510), pp. 421–427.
2. Thune H, Recasens M, Uhlhaas PJ. The 40-Hz auditory steady-state response in patients with schizophrenia: a meta-analysis. *JAMA Psychiatry* 2016, 73(11), pp. 1145–1153.
3. Vierling-Claassen D, Cardin J, Moore CI, Jones SR. Computational modeling of distinct neocortical oscillations driven by cell-type selective optogenetic drive: separable resonant circuits controlled by low-threshold spiking and fast-spiking interneurons. *Frontiers in Human Neuroscience* 2010, 4, 198.
4. Mäki-Marttunen T, Halnes G, Devor A, et al. A stepwise neuron model fitting procedure designed for recordings with high spatial resolution: Application to layer 5 pyramidal cells. *Journal of Neuroscience Methods* 2018, 293, 264–283.
5. Mäki-Marttunen T, Krull F, Bettella F, et al. Alterations in Schizophrenia-Associated Genes Can Lead to Increased Power in Delta Oscillations. *Cerebral Cortex* 2018, 29(2), 875–891.
6. Metzner C, Mäki-Marttunen T, Zurowski B, Steuber V. Modules for Automated Validation and Comparison of Models of Neurophysiological and Neurocognitive Biomarkers of Psychiatric Disorders: ASSRUnit—A Case Study.*Computational Psychiatry 2018*,*2*, 74–91.

## P296 Conservation and change of organizational features during the evolution of neocortical circuits in mammals

### Rodrigo Suarez

#### The University of Queensland, Brisbane, Australia

##### **Correspondence:** Rodrigo Suarez (rsuarezsaa@gmail.com)

*BMC Neuroscience* 2019, **20(Suppl 1)**: P296

The six-layered cerebral cortex, o neocortex, mediates sensory-motor integration and higher order associative and cognitive functions. It constitutes a hallmark of mammalian evolution; other vertebrates, such as birds or reptiles, lack a neocortex, and homologue inputs, outputs and internal circuits are organized in neuronal clusters. The neocortex is extensively interconnected within and between hemispheres via axons that form the anterior commissure in egg-laying monotremes and marsupials, while eutherian mammals (also known as placental) evolved a new route: the corpus callosum. Studies of the cortical connectome have focused on rodents and humans, and have described conserved features of interhemispheric communication via the corpus callosum. However, whether these features arose exclusively in eutherians with callosum origin, or instead they represent ancient features of neocortical organization shared by all mammals have remained unanswered [1]. Here, I will present recent findings of a pan-mammalian map of cortical connections between hemispheres [2]. These include findings in platypus (a monotreme) and dunnarts (a marsupial), combining magnetic resonance imaging, cell-level anatomical mapping and in-pouch electroporation of fluorescent markers in vivo. Despite lacking a corpus callosum, monotremes and marsupials share with eutherians a pattern of connections between similar areas of both hemispheres (homotopy), as well as some hyper-connected regions (hubs). Moreover, contralateral axons are spatially segregated as they cross the midline, and parcellation of the midsagittal anterior commissure is sufficient to reconstruct the main homotopic domains (Fig. 1). Additional features shared between eutherian and non-eutherian mammals include the layer-of-origin of commissural neurons (layers 5 and 2/3), topographic representation of fibers according to the position of the cell bodies within the cortex, and hyperconnected hubs at regions bordering the neocortex on its dorsal extent (cingulate and motor cortices) and its lateral extent (insula and claustrum). These results suggest that ancient principles of neocortical connectivity arose at least 80 million years before the origin of the corpus callosum. Because these features have been conserved throughout mammalian evolution in species with or without a corpus callosum, they likely represent key principles of neocortical development and organization.

**Fig. 1:**
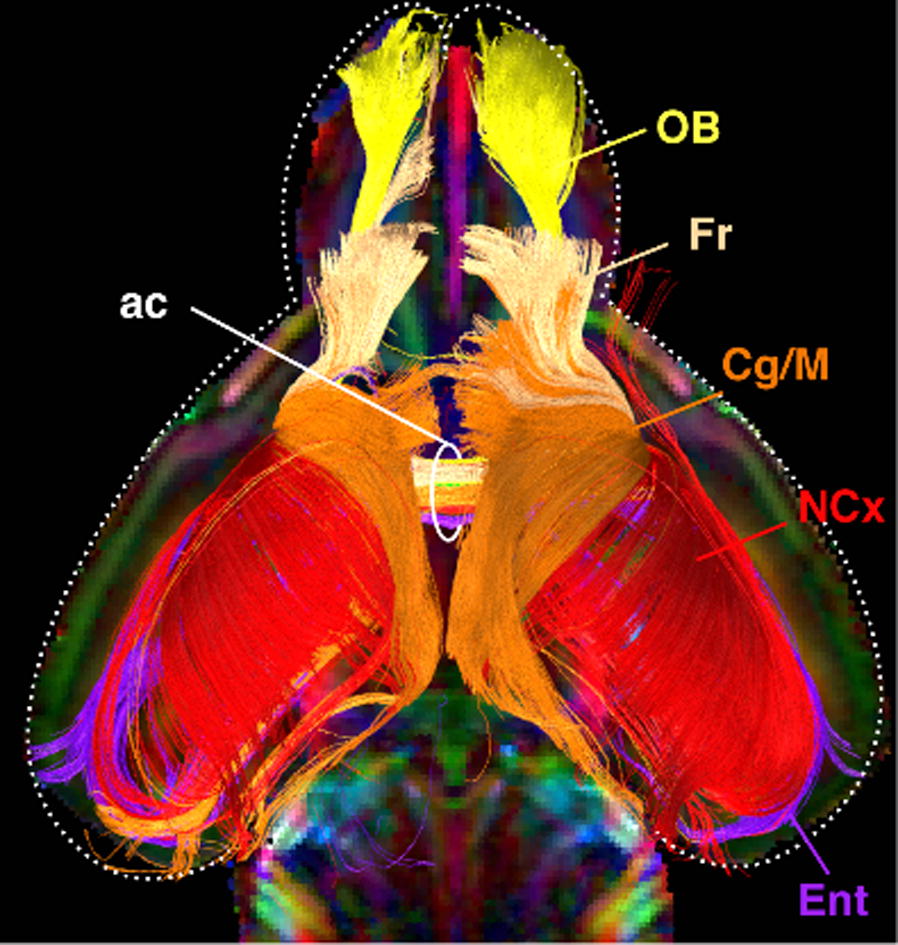
Dorsal view of a fat-tailed dunnart brain, after magnetic resonance imaging and tractography reconstruction. Tracts were generated by parcellating the midsagittal anterior commissure (ac) into five segregated color-coded domains, each labeling homotopic contralateral connections between the olfactory bulbs (OB), frontal cortex (Fr), neocortex (NCx) and entorhinal cortex (Ent)

**Acknowledgements**: This work was supported by the Australian Research Council (DP160103958 and DE160101394).

**References**

1. Suárez R. Evolution of telencephalic commissures: Conservation and change of developmental systems in the origin of brain wiring novelties. *In: Kaas JH, editor. Evolution of Nervous Systems. Volume 2. 2nd edition. Academic: Oxford*; 2017, p205–223.
2. Suárez R, et al. A pan-mammalian map of interhemispheric brain connections predates the evolution of the corpus callosum. *PNAS USA* 2018, 115(38), 9622–9627.

## P297 Integrating classifiers and electrophysiology to better understand hearing loss

### Samuel Smith^1^, Mark Wallace^1^, Joel Berger^2^, Christian Sumner^3^

#### ^1^University of Nottingham, Hearing Sciences, Nottingham, United Kingdom; ^2^University of Iowa, Neurosurgery, Iowa City, United States of America; ^3^Nottingham Trent University, Psychology, Nottingham, United Kingdom

##### **Correspondence:** Samuel Smith (samuel.smith@nottingham.ac.uk)

*BMC Neuroscience* 2019, **20(Suppl 1)**: P297

A moderate hearing loss can pose major challenges for speech identification, principally in noisy environments. This is despite most features of speech remaining audible. It is not yet clear how the neural representation of speech changes following hearing loss, and whether it is in fact worse. Here, in order to quantify this, we present a framework that integrates a Bayesian classifier with neural data.

Neural responses to the presentation of vowel-consonants (VCs) overlapping one another, with onset asynchronies between 0 ms and ±262.5 ms, were recorded from the midbrain of anaesthetised guinea pigs. Animals either had normal hearing (NH) or a moderate high frequency hearing loss imposed (HL). A naïve Bayesian-inspired classifier was implemented to predict auditory perception. The classifier was trained and tested on neural data recorded from either the NH or HL animals, with additional zero-mean Gaussian noise (variance was the classifier’s only fitting parameter). The stimuli used to train the model were VCs in quiet, but it was tested with or without prior knowledge of overlapping VCs. Collectively this protocol has the potential to highlight how hearing loss alters speech identification.

The classifier was set to predict VCs in quiet with a 95% accuracy. When presented with the overlapping VC stimuli the classifier’s performance reduced to as low as 55% (0 ms offset). When this classifier was instead fed the HL data, identification of the VCs in quiet and the overlapping VCs reduced further. Crucially this reduction was more than 10x larger for some temporal overlaps of the competing VC stimuli (e.g. +37.5 ms offset) relative to VCs in quiet. Despite predicted errors mainly resulting from consonant confusions it was vowel confusions that became more prominent following hearing loss. This remained the case when the classifier was run for neurons maximally responsive to frequencies below 3 kHz where there was no measurable hearing loss. Classifier performance improved when tested with prior knowledge of overlapping VCs, particularly for the HL data. Hearing loss may however adversely affect whether such prior information can be used optimally.

Overall, this work offers evidence for a degraded representation of speech in complex acoustic backgrounds at the midbrain level, following hearing loss. The qualitative changes to speech identification that were predicted are not solely attributable to a simple loss of auditory information in the frequencies most affected by hearing loss. Finally, the principle of applying a machine classifier to the neural coding of speech appears to be a promising method for understanding the real-world problems associated with hearing loss.

## P298 Transition probability in decision-making process

### Nicoladie Tam

#### University of North Texas, Department of Biological Sciences, Denton, TX, United States of America

##### **Correspondence:** Nicoladie Tam (nicoladie.tam@unt.edu)

*BMC Neuroscience* 2019, **20(Suppl 1)**: P298

Decision-making process is often considered as a self-decision-making process, independent of the decisions of others. Yet, in social interactions, decisions are often dependent on the decisions of others, especially in social reciprocity. In order to determine the underlying factors contributing to the decision-making process, we propose a computational model in which the decision is determined by computing the conditional probability depending on the decision of the opponent. To account for social reciprocity [1, 2], decisions are dependent on trust, which requires prediction of the opponent’s decision and fairness, which requires reciprocation of decision.

In this computational model, a decision-space diagram of transition probability diagram is used to represent the conditional probability of decision dependent on the prior opponent’s decision. Using such transition probability diagram, equal-reciprocating decision is represented by the diagonal line in which the current decision is the exactly same as the prior decision of the opponent, i.e., a tit-for-tat decision. The decision-space in the upper-triangular region represents decisions that are reciprocated with more generous offers than the prior opponent’s offers. This indicates reciprocating decisions that are nice. On the other hand, decision-space in the lower-triangular region represents decisions that are reciprocated with less generous offers than the prior opponent’s offers. This indicates reciprocating decisions that are mean. Thus, we provided a computational model of transition probability of reciprocating decision-making process based on the prior opponent’s decision, which is dependent on the decisions of others, not just based on self.

**References**

1. Tam ND. Computational social interaction in reciprocity and empathic behavior as behavioral economics and risk tasking behavior. *BMC Neuroscience* 2017, 18(Suppl 1):P103.
2. Tam N. Computational model of the conditional probability of decision-making process as an optimization process. *BMC Neuroscience* 2018, 19(Suppl 2):P167.

## P299 Figure-ground detection by a population of neurons with a variety of receptive-field structures in monkey V4

### Ko Sakai^1^, Kouji Kimura^1^, Yukako Yamane^2,3^, Hiroshi Tamura^3,4^

#### ^1^University of Tsukuba, Department of Computer Science, Tsukuba, Japan; ^2^Japan Society for Promotion of Science, Tokyo, Japan; ^3^Osaka University, Graduate School of Frontiers Bioscience, Osaka, Japan; ^4^Center for Information and Neural Networks, Osaka, Japan

##### **Correspondence:** Ko Sakai (sakai@cs.tsukuba.ac.jp)

*BMC Neuroscience* 2019, **20(Suppl 1)**: P299

Segregation of images into figures and background (FG) is a crucial step for understanding scenes and recognizing objects. As a step towards understanding the formation of FG in intermediate-level ventral visual cortical areas, we focused on the local information contained in natural images and investigated the potential of neuronal activity to signal figures and grounds in the absence of global context and scene analysis. We recorded spiking activities of a population of macaque V4 neurons in response to a variety of natural image patches that extended approximately three times larger than the extent of the classical receptive field (cRF) of the cells. We examined whether the activity of V4 neurons depend on the local FG configuration of stimulus images, and intended to evaluate how a population of V4 neurons represents figure-ground information.

Around one third of the visually responsive neurons showed response modulation depending on the positional relation between the cRF of the neuron and the figural region of the stimulus. First, we estimated the spatial structure of the RFs using spike triggered stimulus average (STSA). With our aim of detecting the spatial organization of the RFs in response to FG configuration, we linked the FG to luminance contrast. We grouped the stimulus patches based on their luminance contrast of the figure region with respect to the ground region. We then generated STSAs for each group and took the difference between the two. This subtraction cancelled out the contrast dependence of the cell. We compensated nonuniformity of luminance in the natural images by subtracting the simple ensemble average of the stimuli from the STSA (equivalent to weight = 1 for all stimuli). The estimated STASs exhibited antagonistic structures; facilitative and suppressive sub-regions on the preferred and non-preferred figure sides, respectively, as shown in Fig. 1. The antagonistic structure might be suitable in the detection of FG from local structures. The sub-regions showed a wide variety in shape and spatial extent, suggesting the limitation of individual neurons in the detection of FG from a variety of natural images and the necessity of the integration of multiple-cell responses.

**Fig. 1:**
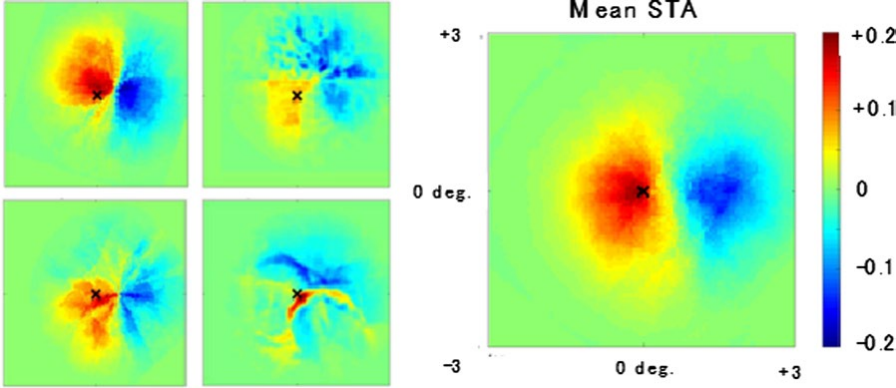
(Left) The computed STSA of four example cells in response to figures and grounds. Antagonistic structures are observed (reddish and blueish colors show facilitation and suppression, respectively). The shape and extent of sub-regions differ across cells. (Right) The mean STSA across FG-responsive cells, with the preferred sides of each cell aligned toward the left of the cRF center

A wide variety of the shape and extent of sub-regions led us to predict population coding in FG judgement. Because the natural image patches include a wide variety of figure shapes and extent, we expected that individual neurons exhibit low consistency in FG determination across a variety of image patches. The measured consistency was approximately 55%, indicating a low capability of individual neurons in judging FG across a variety of natural patches. We then hypothesized that an integration of responses across a small population of FG-modulated neurons would be capable of correctly judging FG across a variety of images. To examine the power of integration and the number of neurons necessary for accurate FG discrimination across a variety of stimuli, we trained a support vector machine to classify figures and grounds of stimuli on the basis of firing activity. The integration of the activities of 40 to 50 neurons yielded the discrimination consistency far greater than that of single neurons (up to 72%), suggesting a distributed representation of FG information in V4.

**Acknowledgments:** This work was supported by JSPS and MEXT, Japan (KAKENHI 26280047, 17H01754, 16K01962, 71J40112, JP15H05921) and RIEC, Tohoku University (H29/A13).

## P300 Brief mindfulness training induces structural plasticity within brain hub

### Rongxiang Tang^1^, Yi-Yuan Tang^2^

#### ^1^Washington University in St. Louis, Psychological and Brain Sciences, St. Louis, WA, United States of America; ^2^Texas Tech University, Lubbock, TX, United States of America

##### **Correspondence:** Yi-Yuan Tang (yiyuan.tang@ttu.edu)

*BMC Neuroscience* 2019, **20(Suppl 1)**: P300

Research in large-scale brain networks has shown that brain structural core includes regions such as posterior medial/cingulate and parietal cortex. These regions have high degree, strength and betweenness centrality, and constitute connector hubs that link major structural modules [1]. Our RCTs indicated that the integrative body-mind training (IBMT) induces brain functional and structural changes related to self-control networks such as anterior cingulate cortex following about 2-10 h of practice [2-4]. However, whether brief mindfulness could induce structural plasticity within brain structural core remains unexplored. Here we targeted the brain structural core—posterior cingulate cortex (PCC) as ROI to examine potential volumetric changes in 40 healthy adults who either received 1 month (about 10 h in total) of IBMT or relaxation training.Structural data (T1-weighted images) were acquiredin a 3-Telsa Siemens Skyraat pre- /post-training and automatically processed using FreeSurfer for cortical reconstruction and segmentation (http://surfer.nmr.mgh.harvard.edu/). Similar to the literature [5-7], a standard longitudinal processing pipelinewas employedto extract reliable volume and thickness estimates. Specifically,an unbiased within-subject template space and image was created using robust, inverse consistent registration. Several processing steps such as skull stripping, Talairach transforms, atlas registration, spherical surface maps and parcellations were then initialized with common information from the within-subject template.The white and pial surfaces were visually inspected and manually edited to correct for errors when necessary. We calculated symmetrized percent change (spc) in volumes for our ROI using long_stats_slopes command lines. The spc is the rate of change with respect to the average volume: spc = 100 * rate / avg. For longitudinal design, this is a more robust measure than the rate of change from time point 1 to 2. Our results indicated a significantly positive spc of the ventral PCC volume in the IBMT group, suggesting that brief mindfulness training can lead to increase in grey matter volume and plasticity withinbrain structural core/hub. Such increases may serve as underlying mechanisms of behavioral improvements in emotion regulation, conscious awareness and attention control that are often observed following mindfulness [4].

**References**

1. Hagmann P et al. Mapping the structural core of human cerebral cortex. *PLoS Biol*. 2008, 6:e159.
2. Tang YY et al. Central and autonomic nervous system interaction is altered by short term meditation. *Proc Natl Acad Sci USA* 2009, 106: 8865–70
3. Tang YY et al. Short-term meditation induces white matter changes in the anterior cingulate. *Proc Natl Acad Sci USA* 2010, 107: 15649–52
4. Tang YY, Holzel BK, Posner MI. The neuroscience of mindfulness meditation. *Nat Rev Neurosci* 2015, 16: 213-225
5. Reuter M, Fischl B. Avoiding asymmetry-induced bias in longitudinal image processing. *Neuroimage* 2011, 57: 19–21.
6. Reuter M, Rosas HD, Fischl B. Highly accurate inverse consistent registration: a robust approach. *Neuroimage* 2010, 53: 1181–1196.
7. Reuter M et al. Within-subject template estimation for unbiased longitudinal image analysis. *Neuroimage* 2012, 61: 1402–1418.

## P301 Short-term mindfulness meditation changes grey matter in insula

### Rongxiang Tang^1^, Yi-Yuan Tang^2^

#### ^1^Washington University in St. Louis, Psychological and Brain Sciences, St. Louis, WA, United States of America; ^2^Texas Tech University, Lubbock, TX, United States of America

##### **Correspondence:** Yi-Yuan Tang (yiyuan.tang@ttu.edu)

*BMC Neuroscience* 2019, **20(Suppl 1)**: P301

Mindfulness meditation induces brain plasticity such as the prefrontal cortex, anterior cingulate cortex (ACC) and insula [1]. Among the areas related to self-control, the insula is a key node of the salience network that plays the key role in integrating external sensory info with internal bodily states and awareness [2]. Research has shown that short-term mindfulness changes insula activity whereas long-term mindfulness changes insula structure [3-5].

Our RCTs showed that the integrative body-mind training (IBMT) induces brain functional and structural changes related to self-control networks such as ACC after 2-10 h of practice [3, 4]. However, whether short-term mindfulness could induce insula structural plasticity remains unexplored. Here, we targeted the anterior insula (AI) as the ROI to examine potential volumetric changes in 40 healthy adults who either received1 month (about 10 h in total)of IBMT or relaxation training. Structural data (T1-weighted images) were acquired in a 3-Telsa Siemens Skyraat pre-/post-training and automatically processed using FreeSurfer for cortical reconstruction and segmentation (http://surfer.nmr.mgh.harvard.edu/).Similar to the literature [6- 8],a standard longitudinal processing pipeline was employed to extract reliable volume and thickness estimates. Specifically, an unbiased within-subject template space and image was created using robust, inverse consistent registration. Several processing steps such as skull stripping, Talairach transforms, atlas registration, spherical surface maps and parcellations were then initialized with common info from the within-subject template.

The white and pial surfaces were visually inspected and manually edited to correct for errors when necessary. We calculated symmetrized percent change (spc) in volumes for our ROI using long_stats_slopes command lines. The spc is the rate of change with respect to the average volume: spc = 100 * rate / avg. For longitudinal design, this is a more robust measure than the rate of change from time point 1 to 2. Our results showed a significantly positive spc of AI volume in the IBMT group. This result suggests that brief mindfulness leads to greater grey matter volume and plasticity in the AI and that such insula plasticity may indicate that IBMT works through executive control and salient networks supported by ACC and insula [1, 3, 4].

**References**

1. Tang YY, Hölzel BK, Posner MI. The neuroscience of mindfulness meditation. Nature Reviews Neuroscience. 2015 Apr;16(4):213.
2. Uddin LQ. Salience processing and insular cortical function and dysfunction. *Nature Reviews Neuroscience* 2015 Jan;16(1):55.
3. Tang YY, Ma Y, Fan Y, et al. Central and autonomic nervous system interaction is altered by short-term meditation. *Proceedings of the national Academy of Sciences* 2009 Jun 2;106(22):8865–70.
4. Tang YY, Lu Q, Feng H, Tang R, Posner MI. Short-term meditation increases blood flow in anterior cingulate cortex and insula. *Frontiers in psychology* 2015 Feb 26;6:212.
5. Hernández SE, Barros-Loscertales A, Xiao Y, Gonzalez-Mora JL, Rubia K. Gray matter and functional connectivity in anterior cingulate cortex are associated with the state of mental silence during Sahaja yoga meditation. *Neuroscience* 2018 Feb 10;371:395–406.
6. Reuter M, Fischl B. Avoiding asymmetry-induced bias in longitudinal image processing. *Neuroimage* 2011 Jul 1;57(1):19–21.
7. Reuter M, Rosas HD, Fischl B. Highly accurate inverse consistent registration: a robust approach. *Neuroimage* 2010 Dec 1;53(4):1181–96.
8. Reuter M, Schmansky NJ, Rosas HD, Fischl B. Within-subject template estimation for unbiased longitudinal image analysis. *Neuroimage* 2012 Jul 16;61(4):1402–18.

## P302 Ultrasonic neuromodulation in multi-compartmental neuron models

### Thomas Tarnaud, Wout Joseph, Luc Martens, Timothy Van Renterghem, Emmeric Tanghe

#### University of Ghent, Waves - Information technology (INTEC), Ghent, Belgium

##### **Correspondence:** Thomas Tarnaud (thomas.tarnaud@ugent.be)

*BMC Neuroscience* 2019, **20(Suppl 1)**: P302

Transcranial focused ultrasound (tFUS) has recently gained attention, due to its capability to modulate brain activity non-invasively, reversibly and with high spatial accuracy. However, the underlying mechanism of ultrasonic neuromodulation (UNMOD) is not well understood. Although several possible mechanisms have been proposed (e.g. acoustic radiation pressure, mechanosensitive ion channels, extracellular cavitation…), only the bilayer sonophore model (BLS) of intramembrane cavitation has been able to provide a comprehensive mathematical framework, predicting how ultrasound can induce action potentials [1-2]. However, numerical modeling studies with the BLS-model have been restricted to single-compartment point-neurons (Fig. 1) [1-4], leaving important questions with respect to UNMOD in the BLS-framework unanswered (e.g. location of the excitation node, importance of the spatial features of the pressure field (phase and intensity distribution), sensitivity of model predictions on spatially distributed parameters…). Furthermore, a spatially extended (multi-compartmental) UNMOD-model would streamline the coupling of neuronal and ultrasonic propagation simulations, benefiting future neural engineering studies concentrating on the design of the ultrasonic transducer or transducer array. In this study, the BLS-model is implemented in NMODL (NEURON) as a distributed-mechanism, and subsequently coupled with modifications of Blue Brain Project multi-compartmental pyramidal cells and interneurons [5]. We investigate the importance of the spatial variations in the pressure field (e.g. subsequent nodes of Ranvier will be subjected to different ultrasonic intensity and phase) and the predicted location of excitation in the BLS-framework. Finally, we determine the quantitative and qualitative importance of multi-compartmental neuron models for UNMOD, by comparing our results with previous studies using single-compartment BLS-simulations. We conclude that the extension of the intramembrane cavitation model to spatially extended neurons is an important step to improve understanding of the underlying mechanism of ultrasonic neuromodulation and enable the combined simulation of the acoustic field and neuronal response.

**Fig. 1:**
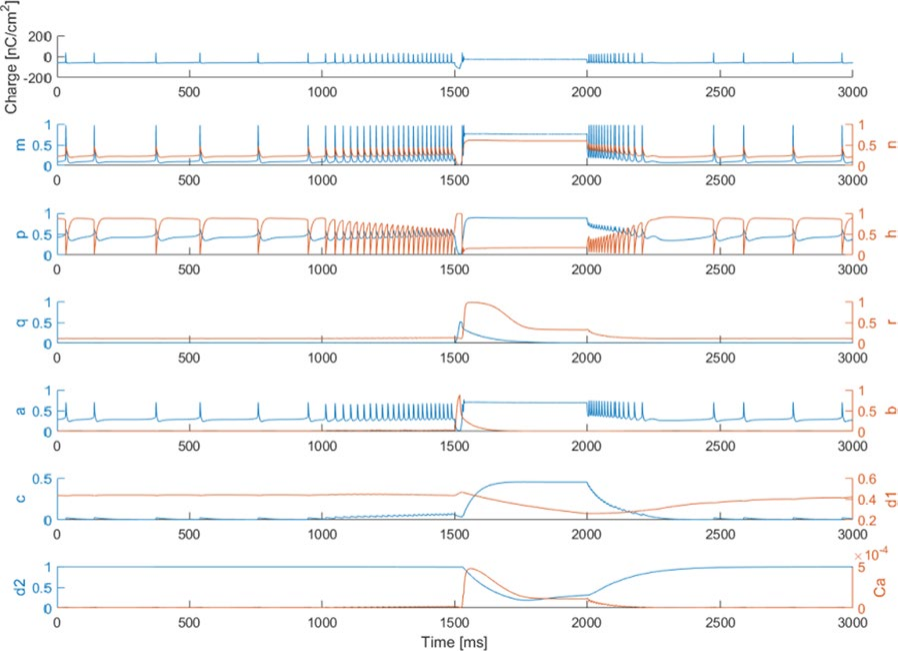
Example simulation of ultrasonic neuromodulation within the BLS-framework. Subthalamic nucleus neuron (Otsuka-model) insonicated with continuous-wave ultrasound (690 kHz, pulse onset at 1000 ms, pulse duration of 1000 ms and inhibitory perturbation at 1500 ms). Different traces represent the membrane charge and gating parameters

**References**

1. Plaksin M, Shoham S, Kimmel E. Intramembrane cavitation as a predictive bio-piezoelectric mechanism for ultrasonic brain stimulation. *Physical review X* 2014 Jan 21;4(1):011004.
2. Plaksin M, Kimmel E, Shoham S. Cell-type-selective effects of intramembrane cavitation as a unifying theoretical framework for ultrasonic neuromodulation. *eNeuro* 2016 May;3(3).
3. Tarnaud T, Joseph W, Martens L, Tanghe E. Computational modeling of ultrasonic subthalamic nucleus stimulation. *IEEE Transactions on Biomedical Engineering* 2018 Sep 6;66(4):1155–64.
4. Tarnaud T, Joseph W, Martens L, Van Renterghem T, Tanghe E. Interaction of electrical and ultrasonic neuromodulation: a computational study. *Brain Stimulation: Basic, Translational, and Clinical Research in Neuromodulation* 2019 Mar 1;12(2):563.
5. Aberra AS, Peterchev AV, Grill WM. Biophysically realistic neuron models for simulation of cortical stimulation. *Journal of Neural Engineering* 2018 Oct 16;15(6):066023.

## P303 Universal automated seizure focus prediction consistent with post-operative outcome

### Manel Vila-Vidal^1^, Carmen Pérez Enríquez^2^, Rodrigo Rocamora^2^, Gustavo Deco^3^, Adrià Tauste Campo^1^

#### ^1^Universitat Pompeu Fabra, Center for Brain and Cognition, Barcelona, Spain; ^2^Hospital del Mar Medical Research Institute, Epilepsy Monitoring Unit, Department of Neurology, Barcelona, Spain; ^3^Universitat Pompeu Fabra, Barcelona, Spain

##### **Correspondence:** Manel Vila-Vidal (m@vila-vidal.com)

*BMC Neuroscience* 2019, **20(Suppl 1)**: P303

Over the last decades, an increasing computational effort has been undertaken to develop quantitative tools for intracranial EEG analysis to better identify the brain regions involved in seizure generation and propagation in patients with drug-resistant epilepsy. Yet, the development of automated methods for seizure onset zone (SOZ) spatial delineation remains challenging due to a number of reasons. The complex localization of the SOZ, the variable number and typology of seizures during the monitoring period and the variety of electrophysiological seizure-onset patterns that may occur even within one patient [7] represent major challenges to design a detection algorithm that is universally valid for all patients. While several algorithms have been proposed to characterize the epileptogenicity of different brain structures [1-6, 8], most of them are based on preselected spectral features of the SEEG signals and might fall short to capture the broad diversity of seizure-specific onset patterns.

Here, we report a fully unsupervised and automatic algorithm for SOZ identification that maximizes the amount of information that can be extracted to this aim from short epochs comprising seizure events (peri-ictal period). The current approach builds upon the previously introduced mean activation (MA) measure [11], which quantifies the average spectral activation of each targeted brain structure for pre-defined frequency and time windows of interest. The methodology developed in this study relies on finding the time-frequency windows of interest where the MA is maximal with respect to a baseline pre-ictal period, while being spatially confined to a few contacts. Central to this approach is the definition of two novel measures, the global activation (GA) and the activation entropy (AE), that are used to monitor the magnitude of spectral changes with respect to the pre-ictal epoch and the spread of these spectral activations, respectively, at different frequencies and as time progresses from seizure onset (Fig. 1). By setting appropriate conditions on the two measures, it is possible to find time-frequency windows where SOZ regions can be optimally discriminated. Selected windows characterize the seizure-specific seizure-onset spectral properties in each case. Within those windows, the most active channels are selected and accumulated across seizures to output a single SOZ per patient.

**Fig. 1:**
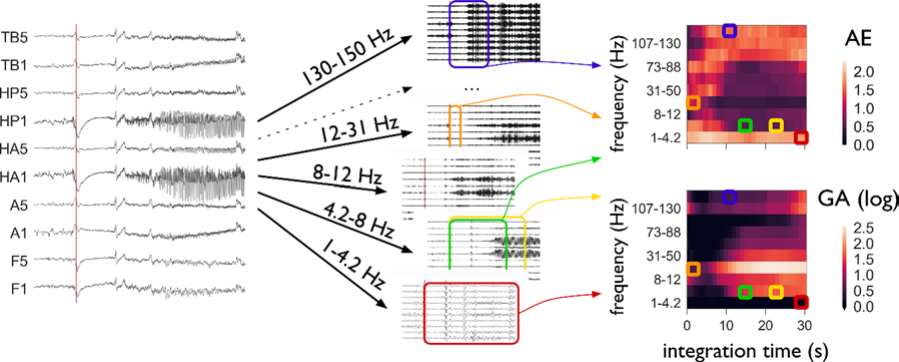
Identification of the most relevant seizure onset windows (SOW) and seizure onset zone (SOZ) detection is illustrated with one exemplary seizure. Signals are band-pass filtered in pre-defined bands of interest spanning the whole spectrum. For each recording site, a mean activation index (MA) is obtained over different time-frequency windows of interest using the Hilbert transform method and averaging the instantaneous power across time. For each window, the MA profile is characterized by two summary measures: the global activation (GA) and the activation entropy (AE), that quantify the magnitude of spectral changes with respect to the pre-ictal epoch and the spread of these spectral activations, respectively. SOW detection is then achieved by finding time-frequency windows that maximize GA under the constraint of low AE. For each SOW, most active regions are considered to be part of the SOZ

Our method was successfully tested with a cohort of 10 patients with clinically assessed SOZ featuring heterogeneous patterns and validated with the post-operative outcome of resective surgery and radio-frequency thermo-coagulation (RF-TC) after follow-up periods from 2 to 6 years. Significantly, we provided evidence that seizure onset is characterized by a shift towards higher values of GA and lower values of AE, regardless of the specific seizure onset spectral features. Furthermore, we showed that time-frequency windows selected by the method reliably characterize a variety of electrophysiological seizure onset patterns described in the literature [7]. Overall, the application of the method on the ictal epoch across all patients yielded an average sensitivity of 0.94 ± 0.09, with an average specificity of 0.90 ± 0.09. SOZ detection was not complete only in three patients but missed SOZ sites lied at most 1.5 mm apart from the delineated region. Complementary to the main results, we further investigated the effect of the recording referencing on the method performance. While no significant differences were found in terms of specificity, the algorithm was more sensitive to SOZ regions in the monopolar than in the bipolar configuration. Additionally, we independently applied the method in a short pre-ictal window (30s before seizure onset), which yielded a lower but still notable accuracy (sensitivity: 0.77 ± 0.32; specificity: 0.77 ± 0.12) supporting the view that the pre-ictal period already carries information that might be of interest for SOZ localization, in agreement with previous studies [1, 10] Finally, cross-validation of the method outputs with postresective information revealed the predictive power of the core variable of the study (MA) as a putative biomarker of the resected zone in long follow-up seizure-free patients.

Overall, we proposed a novel methodology to automatically estimate SOZ regions from intracranially recorded signals that extracts the most relevant time-frequency windows for SOZ detection from the spectral properties of the signals. In practice, this approach can be easily integrated as a complementary diagnostic tool with minimal computational costs for surgical planning, reducing time-consuming SEEG revisions and improving the clinician decision after pre-surgical evaluation.

**Acknowledgements**: M.V. is supported by “la Caixa” Foundation 100010434 (LCF/BQ/DE17/11600022). G.D. is supported by the Spanish Ministry Research Project PSI2016-75688-P (AEI/FEDER), by the EU H2020 FET Flagship Human Brain Project 785907 (HBP SGA2), by the Catalan Research Group Support 2017 SGR 1545, and by the Swiss National Foundation (CRSII5_170873).

**References**

1. Andrzejak RG, et al. Localization of epileptogenic zone on pre-surgical intracranial EEG recordings: toward a validation of quantitative signal analysis approaches. *Brain Topography* 2015, 28(6), 832–837
2. Bartolomei F, Chauvel P, Wendling F. Epileptogenicity of brain structures in human temporal lobe epilepsy: a quantified study from intracerebral EEG. *Brain* 2008; 131 (7): 1818–1830.
3. David O, Blauwblomme T, Job AS, et al. Imaging the seizure onset zone with stereo-electroencephalography. *Brain* 2011; 134(10): 2898–2911.
4. Geertsema EE, Visser GH, Velis DN, Claus SP, Zijlmans M, Kalitzin SN. Automated seizure onset zone approximation based on nonharmonic high-frequency oscillations in human interictal intracranial EEGs. *International Journal of Neural Systems* 2015, 25(05): 1550015.
5. Gnatkovsky V, Francione S, Cardinale F, et al. Identification of reproducible ictal patterns based on quantified frequency analysis of intracranial EEG signals. *Epilepsia* 2011; 52(3): 477–488.
6. Gnatkovsky V, de Curtis M, Pastori C, et al. Biomarkers of epileptogenic zone defined by quantified stereo-EEG analysis. *Epilepsia* 2014; 55(2): 296–305.
7. Lagarde S, et al. Seizure-onset patterns in focal cortical dysplasia and neurodevelopmental tumors: Relationship with surgical prognosis and neuropathologic subtypes. *Epilepsia* 2016, 57(9), 1426–1435
8. Liu S, Sha Z, et al. Exploring the time–frequency content of high frequency oscillations for automated identification of seizure onset zone in epilepsy. *Journal of Neural Engineering* 2016, 13(2), 026026
9. Murphy PM, von Paternos AJ, Santaniello S. A novel HFO-based method for unsupervised localization of the seizure onset zone in drug-resistant epilepsy. *In: Engineering in Medicine and Biology Society (EMBC), 2017 39th Annual International Conference of the IEEE. IEEE,* 2017. P. 1054–1057.
10. Tauste Campo A, Principe A, Ley M, Rocamora R, Deco G. Degenerate time-dependent network dynamics anticipate seizures in human epileptic brain. *PLoS Biology* 2018; 16(4): e2002580.
11. Vila-Vidal M, Principe A, et al. Detection of recurrent activation patterns across focal seizures: Application to seizure onset zone identification. *Clinical Neurophysiology* 2017; 128(6): 977–985.

## P304 Linearly optimal Fisher discriminant analysis of neuronal activity

### Matias Calderini, Nareg Berberian, Jean-Philippe Thivierge

#### University of Ottawa, Department of Psychology, Ottawa, Canada

##### **Correspondence:** Matias Calderini (mcald052@uottawa.ca)

*BMC Neuroscience* 2019, **20(Suppl 1)**: P304

The effects of noise correlations on neuronal stimulus discrimination have been the subject of sustained debate. Both experimental and computational work suggest beneficial and detrimental contributions of noise correlations [1]. The aim of this study is to develop an analytically tractable model of stimulus discrimination that reveals the conditions leading to improved or impaired performance from model parameters and levels of noise correlation.

We begin with a mean firing rate integrator model as an approximation of underlying spiking activity in neuronal circuits [2]. We consider two independent units receiving constant input and time fluctuating noise. Synaptic connectivity between units is not modeled explicitly, but is accounted for through correlated activity that can be tuned independently of firing rate. We implement a perceptron-like readout with Fisher Linear Discriminant Analysis (LDA) [3]. We exploit its closed form solution to find explicit expressions for discrimination error as a function of network parameters (leak, shared inputs, and noise gain) as well as the strength of noise correlation.

First, we derive equations for discrimination error as a function of noise correlation. We find that two sets of results exist, based on the ratios of the difference of means and variance of the distributions of neural activity. In one set, an increase in noise correlation can only cause higher overlap between distributions and a monotonic increase in error. However, under a second set, the distributions stretch along parallel axes, and their resulting overlap leads to error rates that can evolve non-monotonically as a function of correlation. These results provide a potential explanation for previously reported contradictory effects of noise correlation.

Second, we derive functions of network parameters that allow us to examine the diverse behaviour of error rates. Particularly, when the noise gains of a pair of units is increased, the error rate as a function of noise correlation increases multiplicatively. However, when the noise gain of a single unit is increased, the effect of noise can be beneficial to stimulus discrimination. This arises from the stretching of the variance in neural activity on an axis different from that of correlation, thus counterbalancing the overlap caused by the latter.

In sum, we present a framework of analysis that explains a series of non-trivial properties of neuronal discrimination via a simple linear classifier. We show explicitly how different configurations of parameters can lead to drastically different conclusions on the impact of noise correlations. These effects shed light on abundant experimental and computational results reporting conflicting effects of noise correlation. The derived analyses rely on few assumptions and may therefore be applicable to a broad class of neural models whose activity can be approximated by a multivariate distribution.

**References**

1. Averbeck BB, Latham PE, Pouget A. Neural correlations, population coding and computation. *Nature Reviews Neuroscience* 2006, *7*(5), 358–366.
2. Cain N, Barreiro AK, Shadlen M, Shea-Brown E. Neural integrators for decision making: A favorable tradeoff between robustness and sensitivity. *Journal of Neurophysiology* 2013, 109(10), 2542–2559.
3. Calderini M, Zhang S, Berberian N, Thivierge JP. Optimal readout of correlated neural activity in a decision-making circuit. *Neural Computation* 2018, 30, 1573–1611.

## P305 Temporal processing with oscillation-driven balanced spiking reservoirs with conductance based synapses

### Philippe Vincent-Lamarre, Jean-Philippe Thivierge

#### University of Ottawa, Department of Psychology, Ottawa, Canada

##### **Correspondence:** Philippe Vincent-Lamarre (pvinc058@uottawa.ca)

*BMC Neuroscience* 2019, **20(Suppl 1)**: P305

Many cognitive and behavioral tasks—such as interval timing, speech or bird song production—requirethe execution ofprecisely-timed series of behaviors. These behaviors rely on temporal sequences of neural activation that are produced in the absence of external inputs, and therefore cannot be explained by a succession of external stimuli. To produce these sequences, neural circuits must act as autonomous systems (that is, systems whose ongoing dynamics are not driven by fluctuating inputs) whose activation is triggered by a brief external “GO” cue or an intrinsic cue. The objective of this study is to examine the autonomous production of sequences of neural activity using a reservoir computing framework [1, 2]. In this model, an output unit is taught to produce a complex time-varying series as it reads the activity of a recurrent network (reservoir) [2, 3]. The reservoir is a balanced network of leaky integrate-and-fire units with synaptic connections mediated by conductance-based synapses. A subset of cells from the reservoir receives oscillating inputs (Fig. 1A,B). Only the connections between the reservoir and the output unit are trained. Our results show that reservoirs driven with periodic inputs produce robust, meaningful and repeatable neural patterns. In addition, our model displays two key features observed experimentally: i) the emergence of temporal tuning (Fig. 1C), where some neurons consistently elevate their firing rate after a given time delay; and ii) after learning a task at a given speed, the network is able to scale the speed of the execution of the task without additional training (Fig. 1D). When trained on an interval timing task, the model yielded superior performance compared with other implementations while being resistant to relatively large perturbations of its state or parameters, as shown previously [4]. The model performed well when we replaced the artificially generated sine wave driving the network with subnetworks within the reservoir that have slower inhibitory kinetics. In this regime, these subnetworks produce asynchronous spontaneous activity, but a simple step function switches their activity from asynchronous and chaotic to a fully synchronized and repeatable regime (Fig. 1E). These subnetworks can be embedded in the reservoir to create a plausible source of repeatable oscillating inputs (Fig. 1F). In sum, our work proposes a novel role for neuronal oscillations found in cortical circuits, where they may serve as a collection of inputs from which a network can robustly generate complex dynamics and implement rich computations.

**Fig. 1:**
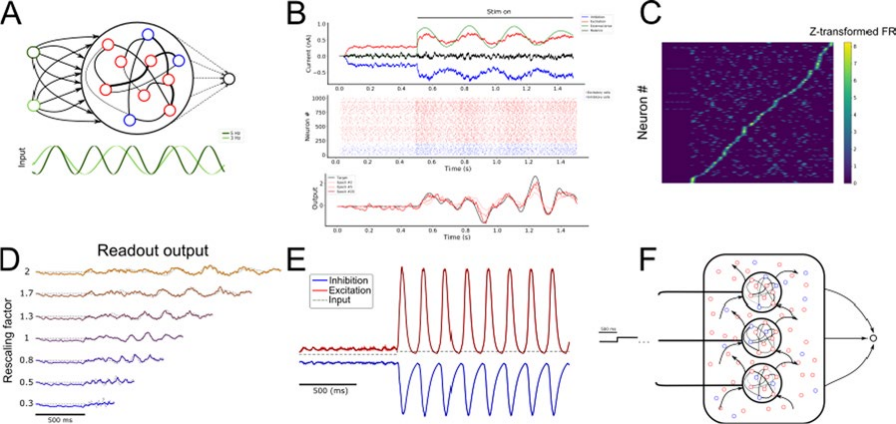
**a** Architecture of the model. **b** Sample activity of a network. **c** Cells showing a time-locked increase in activity during a trial. **d** Temporal rescaling of the output of a network trained at only one speed. **e** Synchronized activity in a network with increased inhibitory decay. **f** Architecture of the model with oscillating subnetworks embedded in the reservoir

**References**

1. Laje R, Buonomano DV. Robust timing and motor patterns by taming chaos in recurrent neural networks. *Nature neuroscience* 2013 Jul;16(7):925.
2. Sussillo D, Abbott LF. Generating coherent patterns of activity from chaotic neural networks. *Neuron* 2009 Aug 27;63(4):544–57.
3. Nicola W, Clopath C. Supervised learning in spiking neural networks with FORCE training. *Nature communications* 2017 Dec 20;8(1):2208.
4. Vincent-Lamarre P, Lajoie G, Thivierge JP. Driving reservoir models with oscillations: a solution to the extreme structural sensitivity of chaotic networks. *Journal of computational neuroscience* 2016 Dec 1;41(3):305–22.

## P306 Emergence of gamma rhythms in V1 during the critical period requires balance of activity between interneuron subtypes

### Justin Domhof, Paul Tiesinga

#### Radboud University, Neuroinformatics, Nijmegen, Netherlands

##### **Correspondence:** Paul Tiesinga (p.tiesinga@science.ru.nl)

*BMC Neuroscience* 2019, **20(Suppl 1)**: P306

The development of the primary visual cortex (V1) of mice is characterized by distinct time windows with enhanced plasticity. During each of these periods, neurons develop feature selective spiking patterns with respect to a variety of features of visual stimuli. The critical period (CP), for example, is the time interval wherein ocular dominance is established and concurrently gamma rhythms emerge in the network’s spontaneous activity. In contrast, visual stimulation results in enhancement of beta rhythms at the expense of these gamma rhythms. So far, models have been presented for each rhythm in isolation. Here, we, therefore, propose a spiking neuron network model that unites these individual aspects and allows for the inference of mechanisms and the exploration of the network’s synchronization properties. It comprises excitatory pyramidal cells and two types of inhibitory interneurons. The first is the parvalbumin expressing (PV) interneuron, which produces gamma oscillations, while the other interneuron group, the somatostatin expressing cell, was found to generate beta rhythms. Our results indicate that for both types of oscillations a pyramidal-interneuron gamma (PING) mechanism was at play. In addition, our findings show that prominent gamma and beta oscillations in respectively the spontaneous and visually evoked activity of the mouse V1 can only occur within the same network configuration if there is a balance between both types of interneurons (Fig. 1). This harmony requires that PV and SOM neurons have a similar influence on pyramidal cells and are both active in the spontaneous state of the network. Taken together, our results demonstrate that PV and SOM cell inhibitions must be balanced for a proper functioning of the V1. Since spontaneous gamma rhythms emerge during the CP, our findings furthermore support the idea that PV cells become integrated in the circuit of this cortical area during this time window. This concurrently activates new plasticity mechanisms, which in their own turn can be exploited to restore developmental visual deficits.

**Fig. 1:**
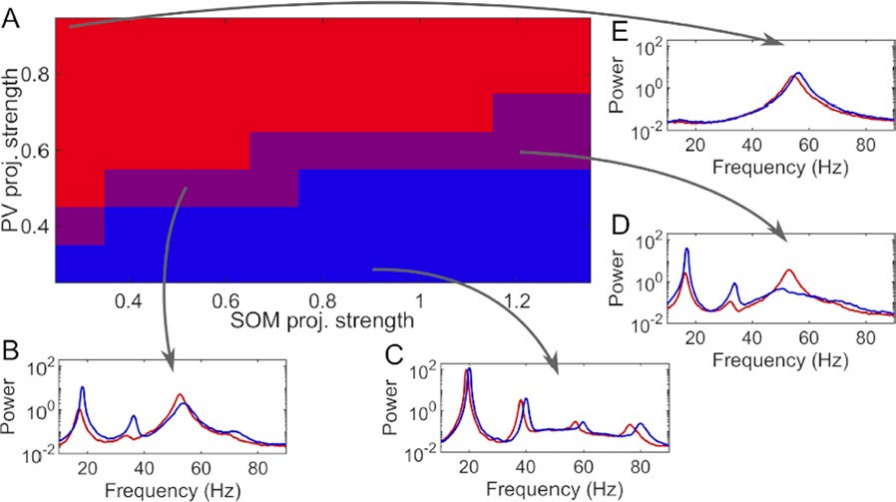
A balance between the inhibitory contributions of parvalbumin and somatostatin expressing cells is required for experimentally observed changes in network synchronization to occur. **a** Network synchronization types as a function of the parvalbumin and somatostatin expressing cell projection strengths. **b**–**e** Corresponding frequency spectra

## P307 Rodent whisking enters the third dimension

### Benoit Meynart, Charl Linssen, Paul Tiesinga

#### Radboud University, Neuroinformatics, Nijmegen, Netherlands

##### **Correspondence:** Paul Tiesinga (p.tiesinga@science.ru.nl)

*BMC Neuroscience* 2019, **20(Suppl 1)**: P307

Rodents have an array of whiskers that aids in navigating their environment. We are interested in the characteristics of sensory information that enters the brain via the whiskers and aim to determine the resulting spatiotemporal properties of activity patterns in barrel cortex. As a first step towards this goal we modeled the structural and dynamical properties of the whiskers using a mechanical model. A rod can be modeled according to the nonlinear equations of elasticity theory [3] which can be linearized to obtain the Euler-Bernoulli equations [3], however, the latter are not appropriate for the typical bending angles of rodent whiskers. Current state of the art for nonlinear whisker modeling is therefore essentially limited to 2D [2], which presents a problem because due to the intrinsic curvature of the whisker in combination with applied contact forces from objects in the environment, the shape of the whisker is not constrained to be in the plane. We have therefore formulated the full 3D equations for whisker structure in a way that can be solved either directly using a standard integrator for boundary value problems or by optimizing an objective function generated by integrating the equations directly. The key innovation was to use the Bishop coordinate frame [1] to parallel transport the intrinsic curvature along the deformed whisker shape and to model the intrinsic curvature using a form for which the path length could be calculated analytically.

To validate our approach we first compared the results for the 3D equations to published results for the 2D equations [2], for the case when contact force is in the plane defined by the intrinsic curvature, which constrains the shape to be in the same 2D plane. We successfully reproduced the bifurcation diagram as a function of push angle. We then repeated the same analysis in 3D which again resulted in a saddle node bifurcation. In addition, we compared the results of this static model to a dynamical model, comprised of discrete segments coupled through torsion springs, which provided suggestions for how to adjust the parameter settings to match resonance frequencies to experimental measurements.

**References**

1. Bishop RL. There is more than one way to frame a curve. *The American Mathematical Monthly* 1975 Mar 1;82(3):246–51.
2. Hires SA, Pammer L, Svoboda K, Golomb D. Tapered whiskers are required for active tactile sensation. *Elife* 2013 Nov 19;2:e01350.
3. Landau LD, Lifshitz EM, Kosevich AM, Pitaevskiĭ LP. Theory of elasticity. *Pergamon Press* 1986, Oxford Oxfordshire; New York.

## P308 Finite element method pipeline to optimize electrical properties of ECoG

### Meron Vermaas^1^, Maria Carla Piastra^2,3^, Nick Ramsey^4^, Paul Tiesinga^1^

#### ^1^Radboud University, Donders Institute for Brain, Neuroinformatics, Nijmegen, Netherlands; ^2^Radboud University Nijmegen Medical Centre, Nijmegen, Netherlands; ^3^Radboud University, Donders Institute for Brain, Cognitive Neuroscience, Nijmegen, Netherlands; ^4^University Medical Center Utrecht, Brain Center Rudolf Magnus, Department of Neurology and Neurosurgery, Utrecht, Netherlands

##### **Correspondence:** Meron Vermaas (m.vermaas@science.ru.nl)

*BMC Neuroscience* 2019, **20(Suppl 1)**: P308

Brain computer interfaces require recordings of relevant neuronal population activity with high precision and/or stimulate a spatially restricted set of neurons. In order to achieve that, both the precise placement of the electrode grid on the cortex and the electrode properties, such as the electrode size and material, need to be optimized with regard to the subject’s head anatomy. For this, it is crucial to have a reliable method and tool able to calculate the extracellular potential generated by realistic population activity patterns and to incorporate the properties of the electrodes explicitly in the model. In this study, this need is addressed by introducing a new open-source pipeline based on the finite element method implemented in FEniCS.

We have tested our pipeline using several standard scenarios and source configurations such as a monopole source in two infinite half-spaces with different conductivities and a multi-layer sphere model, each layer having a different conductivity. We report on the heuristics for picking the number of mesh elements and their sizes to obtain accurate results. This can primarily be described as function of the distance of a source to a surface across which accurate values need to be simulated. In addition, as a proof of concept, we simulated the extracellular potential in an individualized realistically shaped head model.

We were interested in the influence the electrode properties have on the measurements, as well as on the distribution of the extracellular potential itself. This effect is expected to be especially relevant in electrocorticography (ECoG) because of the large electrode surface relatively close to the current source. Therefore, most electrode placements in the geometries were made comparable to ECoG electrodes. We considered four models: (1) a virtual electrode that records the extracellular potential at the center of the electrode, which implies a zero-flux boundary condition (homogeneous Neumann boundary condition); (2) an electrode that absorbs currents and that is an equipotential (Dirichlet boundary condition); (3) an electrode explicitly modeled as a conductive sheet either having a thickness in the geometry or using a thin-layer approximation (mixed boundary condition); (4) an electrode modeled as a sheet with a pseudocapacitive constant phase angle impedance and a charge transfer resistance, which includes the effects of the boundary layer formed at electrolyte-conductor interfaces and for which we need to do a frequency-resolved calculation. Furthermore, we included a tissue model with a dielectric constant in addition to a conductivity, with parameters taken from the literature. Overall, the first electrode model (virtual electrode) and pure conductivity tissue model yields an excellent approximation to the more realistic electrode models. When the electrodes are large, they affect the field itself which can deviate from model 1 by averaging the underlying potential distribution.

Taken together, these results demonstrate this pipeline is an appropriate tool to simulate the signals generated on ECoG grids by the spatiotemporal electrical activity patterns produced by cortical neurons and thereby allows to optimize grids for brain computer interfaces including exploration of more exotic electrode materials/properties.

## P309 A burst motif for attentional control

### Pablo Casaní Galdón, Paul Tiesinga

#### Radboud University, Donders Institute for Brain, Neuroinformatics, Nijmegen, Netherlands

##### **Correspondence:** Pablo Casaní Galdón (pablocasani@gmail.com)

*BMC Neuroscience* 2019, **20(Suppl 1)**: P309

Spatiotemporally coordinated neuronal activity is key for inter-areal information transfer. Specifically, the strength of synchronous activity between brain regions has been related to many cognitive processes, often in a frequency-band specific way. Recent experiments show that attentional processes modulate communication between frontal brain regions. During a selective attention task, recordings from macaque prefrontal cortex (PFC) and the anterior cingulate cortex (ACC) were obtained. Spike train analysis showed an increase in the proportion of high-frequency burst events, during the attention period, relative to baseline [4]. The combination of a decrease of non-burst spikes together with an increase of burst events was responsible for the increase of burst proportion (burst-events/non-burst-spikes). This effect is especially evident in broad-spiking cells, although also apparent in narrow-spikers [3].

Larkum et al. [1] showed that a burst of somatic action potentials of a Layer 5 (L5) pyramidal cell can be triggered as the result of a calcium spike traveling from dendrite to soma [1]. These calcium waves are triggered by the coincidence of back-propagating action potentials and distal dendritic excitatory inputs near the dendritic initiation site. This mechanism—BAC firing—could be relevant for integration of top-down and bottom-up information. Low-threshold spiking cells called Martinotti cells inhibit L5 pyramidal cells on their tuft in the superficial layers. Hence, it is hypothesised that the coordinated activity of these cells could modulate BAC firing.

To test this hypothesis, we used a reduced biophysical model, comprised of two leaky integrate-and-fire compartments with a somatic spike-triggered afterhyperpolarization current and a voltage-dependent dendritic calcium current, respectively, connected by an ohmic resistance [2]. The input current to the somatic compartment was modelled as an Ornstein-Uhlenbeck (O-U) process with correlation time (τ) = 3 ms, whereas rhythmic inhibitory synaptic inputs drove the dendritic compartment. We measured the burst proportion for two conditions in which the excitatory inputs to the dendrite had similar statistics but the degree of precision of the inhibitory inputs to the dendritic compartment was varied. When the inhibitory inputs changed from low precision (a Poisson spike train with constant instantaneous probability of spiking, Fig. 1A) to high precision (an oscillatory spike train with period 100 ms and standard deviation 18 ms, Fig. 1B), both the non-burst firing rate and burst proportion increased, even though the mean number of inhibitory inputs remained the same.

**Fig. 1:**
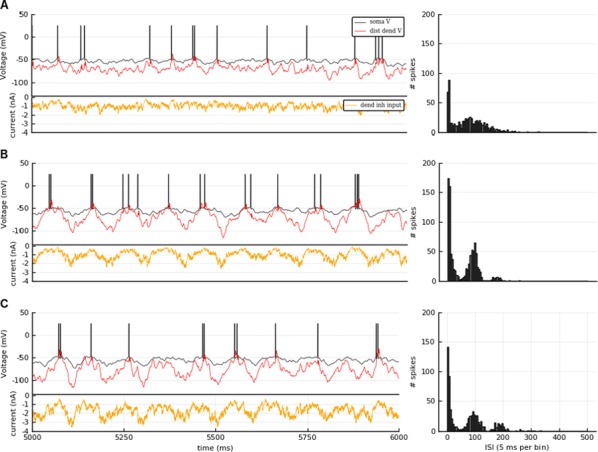
Synchronous inhibition modulates burst fraction. On the left, the model’s voltage traces and inhibitory synaptic current. On the right, the interspike interval histogram

Further, when the input rate of the high precision inhibition and the excitatory dendritic input increased simultaneously relative to the low precision case the non-burst firing rate decreased (Fig. 1C). Nevertheless the fraction as well as the absolute number of bursting events increased. This effect only occurred within a restricted frequency band of 5–20 Hz.

These simulations show that we could reproduce the experimental results on burst proportion in a single neuron model, the next step is to build a multi-area network based on this motif.

**References**

1. Larkum ME, Zhu JJ, Sakmann B. A new cellular mechanism for coupling inputs arriving at different cortical layers. *Nature* 1999 Mar;398(6725):338.
2. Larkum ME, Senn W, Lüscher HR. Top-down dendritic input increases the gain of layer 5 pyramidal neurons. *Cerebral cortex* 2004 Oct 1;14(10):1059–70.
3. Voloh B, Womelsdorf T. Cell-type specific burst firing interacts with theta and beta activity in prefrontal cortex during attention states. *Cerebral Cortex* 2017 Nov 9;28(12):4348–64.
4. Womelsdorf T, Ardid S, Everling S, Valiante TA. Burst firing synchronizes prefrontal and anterior cingulate cortex during attentional control. *Current Biology* 2014 Nov 17;24(22):2613–21.

## P310 A combined volume conduction and nerve fiber model for assessment of electrode design in relation to preferential activation of small cutaneous nerve

### Aida H. Poulsen, Ole K. Andersen, Carsten D. Mørch, Jenny Tigerholm

#### Aalborg University, Health Science and Technology, Aalborg, Denmark

##### **Correspondence:** Aida H. Poulsen (ahp@hst.aau.dk)

*BMC Neuroscience* 2019, **20(Suppl 1)**310

Small fiber neuropathy is an increasing health care issue and there is a current need for an easy, fast, non-invasive and safe assessment of small fiber function. Electrical stimulation meets these requirements; however, conventional surface electrical stimulation activates large fibers at lower intensities than small fibers. As small fibers terminate more superficially than large fibers, three electrodes have achieved some degree of preferential activation of small fibers, by producing a higher current density in the superficial skin layers than in the deeper tissues. However, the selectivity of the electrodes is highly debated. The electrodes were developed empirically and have not been directly compared. Hence, the aim of the present study was to develop a computational model to compare the nerve fiber activation for the different electrodes and identify important design features for achieving preferential small fiber activation.

Three electrodes for small fiber activation: intra-epidermal, pin, and planar concentric as well as a regular patch electrode were modeled in two steps. The first step was a finite element model of the skin (COMSOL, version 5.3) estimating the electrical field. The model consisted of four horizontal skin layers: stratum corneum, epidermis, dermis, and hypodermis, with tissue thickness, conductance and permittivity adopted from literature. The second step was two axon models (NEURON, version 7.6), describing the cutaneous nerve fiber activation. Both a small fiber (Aδ-fiber) and a large fiber (Aβ-fiber) models were developed with a diameter of 3.5 µm and 9 µm, respectively. The models included the following ion channels: Nav1.6, Nav1.7, Nav1.8, Nav1.9, Kdr, KM, KA, and the HCN channel. Experimental validation of the models included measurements of impedance and reaction times, for all electrodes. Impedances were obtained for frequencies between 10 Hz and 100 kHz, with 10 measuring points per decade. Reaction times were acquired at perception threshold intensities for rectangular pulses of 0.1, 0.5, 1, 10, 25, and 50 ms duration.

Impedance measurements corresponded well with the computational model. The current density in the epidermis was higher for electrodes with smaller cathode area, while current spread into deeper tissues was more prominent in electrodes with larger anode-cathode distance and larger anode area. The largest difference in the current density between the epidermal and dermal layer was observed for the intra-epidermal electrode, which also produced the largest difference in activation threshold between the nerve fiber models. Reaction times were 6.8%, 10.6% and 17.3 % shorter for the patch electrode (p<0.05), compared to the pin, planar concentric, and intra-epidermal electrode, respectively.

The intra-epidermal electrode was the most selective for small fiber activation. The cathode-anode distance and the anode area are important design features in order to limit the current spread to the dermis, while a small cathode area is the most important feature for producing high current density in the epidermis.

## P311 Possible mechanisms for gamma-nested oscillations in the hippocampus

### Marco Segneri^1^, Simona Olmi^2^, Alessandro Torcini^1^

#### ^1^Université de Cergy-Pontoise, Laboratoire de Physique Théorique et Modélisation, Pontoise, France; ^2^Inria Sophia Antipolis Mediterranee Research Centre, MathNeuro Team, Sophia Antipolis, France

##### **Correspondence:** Marco Segneri (marco.segneri.1991@hotmail.it)

*BMC Neuroscience* 2019, **20(Suppl 1)**: P311

The CA1 area of the hippocampus exhibits multiple types of gamma oscillations in vivo, which can be segregated based on their spectral, temporal and spatial profiles [1-4], however the exact nature and origin of the intrinsic CA1 gamma oscillations is still under debate. Recently, Butler et al. in [5] have demonstrated that the CA1 in vitro is capable of generating intrinsic gamma oscillations in response to optogenetic theta stimulation, thus demonstrating that CA1 can produce its own gamma oscillation in addition to inheriting activity from the upstream regions. In particular, sinusoidal optical stimulation of CA1 at theta frequency was found to induce robust theta-nested gamma oscillations with a temporal and spatial profile similar to CA1 gamma in vivo. We can define a theta-nested gamma oscillation, a gamma oscillation which is phase-amplitude coupled to the theta stimulation, occurring at the peak of each sinusoidal stimulation cycle. Further measurements suggest that this intrinsic CA1 gamma oscillation is generated via a PING mechanism.

In order to reproduce the phenomenon revealed by [5], we considered spiking neural networks of quadratic integrate and fire neurons (QIF) as well as the corresponding exact mean field description, recently developed in [6]. In particular, we considered a two networks setup to investigate whether PING or ING mechanisms can be at the origin of the gamma-nested oscillations reported in [5]. Regarding the PING case, we studied a setup composed of an excitatory and an inhibitory network, coupled with fast synapses; moreover excitatory neurons are subject to an excitatory theta forcing. On the other hand, for the ING setup, we examined a self-coupled inhibitory network with exponential post-synaptic potentials driven by an excitatory theta stimulation [7]. We have been able to obtain in both cases theta-nested gamma oscillations and to reproduce to a large extent the experimental observations (see Fig. 1).

**Fig. 1:**
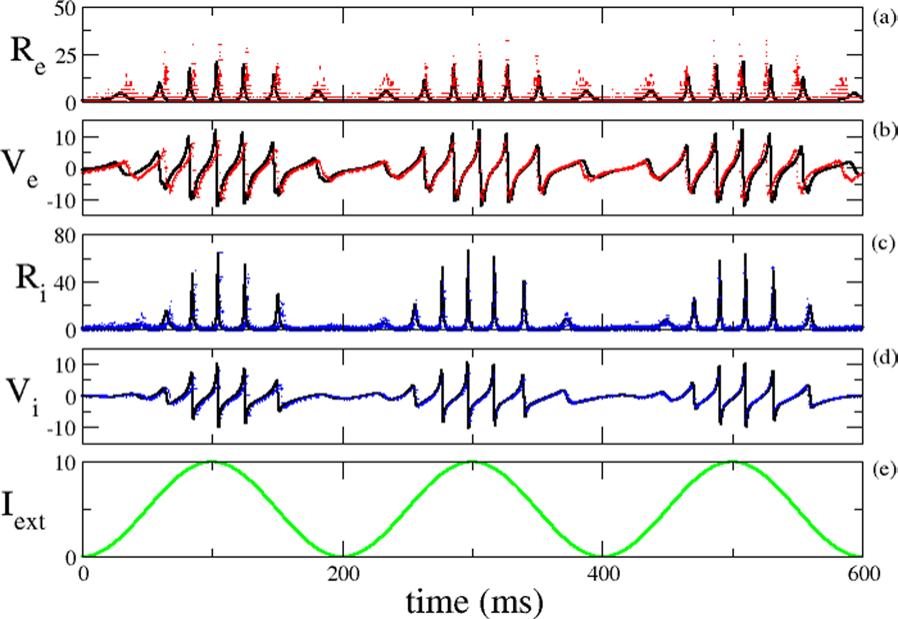
PING and ING mechanisms originating gamma-nested oscillations. The response of the excitatory (inhibitory) population in the PING (ING) configuration to the theta-forcing **e** are shown in panels **a**, **b** (**c**, **d**): average rates in **a**, **d** and average membrane potentials in **b**, **c**. Solid lines refer to the mean field, while symbols to the spiking network simulations

Furthermore, the bifurcation analysis of the mean field revealed that the origin of the theta-nested gamma oscillations can be in both cases associated to the proximity of the non-oscillatory resting state to a supercritical Hopf bifurcation, that in presence of the theta forcing is excited, giving rise to gamma oscillations super imposed on the theta rhythm, somehow similarly to what previously found in [8].

**References**

1. Csicsvari J, Jamieson B, Wise KD, Buzsáki G. Mechanisms of gamma oscillations in the hippocampus of the behaving rat. *Neuron* 2003 Jan 23;37(2):311–22.
2. Colgin LL, et al. Frequency of gamma oscillations routes flow of information in the hippocampus. *Nature* 2009 Nov;462(7271):353.
3. Belluscio MA, Mizuseki K, Schmidt R, Kempter R, Buzsáki G. Cross-frequency phase–phase coupling between theta and gamma oscillations in the hippocampus. *Journal of Neuroscience* 2012 Jan 11;32(2):423–35.
4. Schomburg EW, Fernández-Ruiz A, Mizuseki K, et al. Theta phase segregation of input-specific gamma patterns in entorhinal-hippocampal networks. *Neuron* 2014 Oct 22;84(2):470–85.
5. Butler JL, Mendonça PR, Robinson HP, Paulsen O. Intrinsic cornu ammonis area 1 theta-nested gamma oscillations induced by optogenetic theta frequency stimulation. *Journal of Neuroscience* 2016 Apr 13;36(15):4155–69.
6. Montbrió E, Pazó D, Roxin A. Macroscopic description for networks of spiking neurons. *Physical Review X.* 2015 Jun 19;5(2):021028.
7. Devalle F, Roxin A, Montbrió E. Firing rate equations require a spike synchrony mechanism to correctly describe fast oscillations in inhibitory networks. *PLoS computational biology* 2017 Dec 29;13(12):e1005881.
8. Onslow AC, Jones MW, Bogacz R. A canonical circuit for generating phase-amplitude coupling. *PloS one* 2014 Aug 19;9(8):e102591.

## P312 Coexistence of fast and slow gamma oscillations in one population of inhibitory spiking neurons

### Hongjie Bi^1^, Matteo di Volo^2^, Marco Segneri^1^, Alessandro Torcini^1^

#### ^1^Université de Cergy-Pontoise, Laboratoire de Physique Théorique et Modélisation, Pontoise, France; ^2^Unite de Neuroscience, Information et Complexité (UNIC), Gif sur Yvette, France

##### **Correspondence:** Hongjie Bi (hongjiebi@gmail.com)

*BMC Neuroscience* 2019, **20(Suppl 1)**: P312

Gamma rhythms are the most studied brain oscillations due to their crucial role for attention, memory formation and for their relevance for focal seizures. Recently two different experiments have shown the coexistence of fast and slow gamma oscillations in the hippocampus of rodents (see reference [1] and [2]). These studies have analyzed the modulation of gamma oscillations induced by the theta rhythm, present during locomotory actions and rapid eye movement sleep, and have revealed clear phase locking between theta and gamma oscillations in the two coexisting bands, thus suggesting their functional relevance. The mechanisms behind the emergence of these two distinct gamma bands are not yet clarified, in particular it is not clear if these are induced by external inputs [2] or generated locally [3].

In our study, we report for the first time the coexistence of fast and slow gamma oscillations in a single inhibitory neural population. Furthermore, we show that the two rhythms have different “physical” origin: one being driven by coordinated tonic firing of the neurons the other by endogenous noise due to irregular neural firing. As discussed in [4] these are the only two possible mechanisms able to generate gamma oscillations in purely inhibitory networks, for the first time we show that both mechanisms can coexist in the same model and that theta forcing can activate one or the other mechanism at different theta phases and give rise to different phase locking with the two gamma rhythms during the same stimulation session (see Fig. 1), in agreement with experimental results reported in [1] and [2]. In particular, we have shown that fast gamma are locked to a strong excitatory input, resembling that generated in the CA1 by the activity of the place cells evoked from direct inputs from the medial enthorinal cortex (MEC) as reported in [2], while low gamma COs emerge when excitation and inhibition balance, somehow indicating that these COs can occur when the place cell activity is mostly inhibited by inputs originating from the CA3 region of the hippocampus as suggested in [1, 2].

**Fig. 1:**
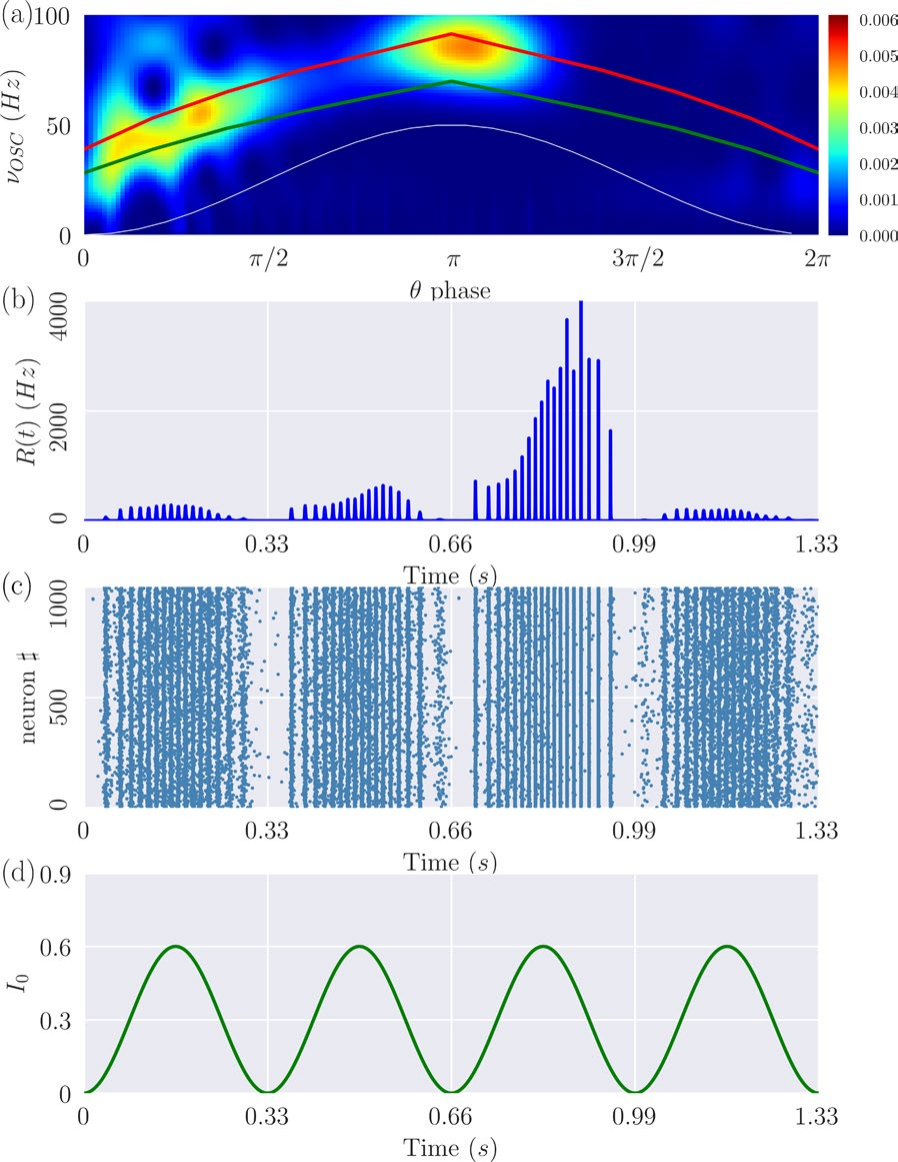
Fast and slow gamma oscillations entrainment with the theta phase **a** Spectrogram of the collective oscillations as a function of the phase of the theta forcing I0, which is reported in **a** as a white solid line in arbitrary units. **b** Instantaneous firing rate; **c** raster plot and the profile of the forcing current I0 **d** versus time

Finally, to gain analytic insights on the dynamics besides direct simulations of the spiking network we employ an effective mean field model (which is an extension of that introduced in [5] for fully coupled networks) and that despite its extreme simplicity can reproduce the dynamics of sparse balanced networks [6], which are commonly believed to be a realistic representation of cortical dynamics dominated by endogenous fluctuations.

**References**

1. Belluscio MA, Mizuseki K, Schmidt R, Kempter R, Buzsáki G. Cross-frequency phase–phase coupling between theta and gamma oscillations in the hippocampus. *Journal of Neuroscience* 2012 Jan 11;32(2):423–35.
2. Colgin LL, Denninger T, Fyhn M, et al. Frequency of gamma oscillations routes flow of information in the hippocampus. *Nature* 2009 Nov;462(7271):353.
3. Craig MT, McBain CJ. Fast gamma oscillations are generated intrinsically in CA1 without the involvement of fast-spiking basket cells. *Journal of Neuroscience* 2015 Feb 25;35(8):3616-24.
4. Buzsáki G, Wang XJ. Mechanisms of gamma oscillations. *Annual review of neuroscience* 2012 Jul 21;35:203–25.
5. Montbrió E, Pazó D, Roxin A. Macroscopic description for networks of spiking neurons. *Physical Review X* 2015 Jun 19;5(2):021028.
6. di Volo M, Torcini A. Transition from asynchronous to oscillatory dynamics in balanced spiking networks with instantaneous synapses. *Physical review letters* 2018 Sep 17;121(12):128301.

## P313 Transition from asynchronous to oscillatory dynamics in balanced spiking networks with instantaneous synapses

### Matteo di Volo^1^, Alessandro Torcini^2^

#### ^1^CNRS, Paris, France; ^2^Université de Cergy-Pontoise, Laboratoire de physique théorique et modélisation, Pontoise, France

##### **Correspondence:** Matteo di Volo (m.divolo@gmail.com)

*BMC Neuroscience* 2019, **20(Suppl 1)**: P313

We report a transition from asynchronous to oscillatory behavior in balanced inhibitory networks for class I and II neurons with instantaneous synapses. Collective oscillations emerge for sufficiently connected networks. Their origin is understood in terms of a recently developed mean-field model, whose stable solution is a focus. Microscopic irregular firings, due to balance, trigger sustained oscillations by exciting the relaxation dynamics towards the macroscopic focus. The same mechanism induces in balanced excitatory-inhibitory networks quasiperiodic collective oscillations.

## P314 A simple model of neural autapse reconciles diverse experimental observations

### Hazem Toutounji

#### Institute of NeuroInformatics, UZH/ETH, Zurich, Switzerland

##### **Correspondence:** Hazem Toutounji (hazem@ini.ethz.ch)

*BMC Neuroscience* 2019, **20(Suppl 1)**: P314

An autapse is a synapse connecting the neuron to itself. Previous anatomical studies suggest that autapses in excitatory cortical neurons known as pyramidal cells (PCs) are rare, irrelevant errors in neuronal wiring. However, a recent electrophysiological study challenges this view by showing that autapses are a common phenomenon, specifically in layer 5 PCs, that are also functionally relevant to neural computation [1]. Among other findings, Yin and colleagues [1] observed that autapses enhance bursting in PCs, which may suggest a role in boosting neural information transmission. Furthermore, previous studies on the aplysia demonstrate that autapses in motor neurons facilitate persistent activity (necessary for memory) in the absence of sufficiently strong input [2], a phenomenon that was not observed in layer 5 PCs according to [1].

The aim of this work is to reconcile these disparate experimental observations within a single theoretical framework, namely, dynamical systems theory. I augmented a simple, biophysically motivated neural model with autaptic excitation. My initial simulations show that, for different parameter choices, the presence of an autapse either enhances bursting (Fig. 1, left) or leads to persistent activity in absence of strong external excitation (Fig. 1, right). This suggests that this autaptic neuron model can support diverse modes of operation that correspond to different neuro-computational properties of functionally specific cell types. Furthermore, augmenting the autapse with delay results in chaotic dynamics within a biophysically plausible parameter regime. This suggests autaptic neurons as potential contributors to neuronal firing variability.

**Fig. 1:**
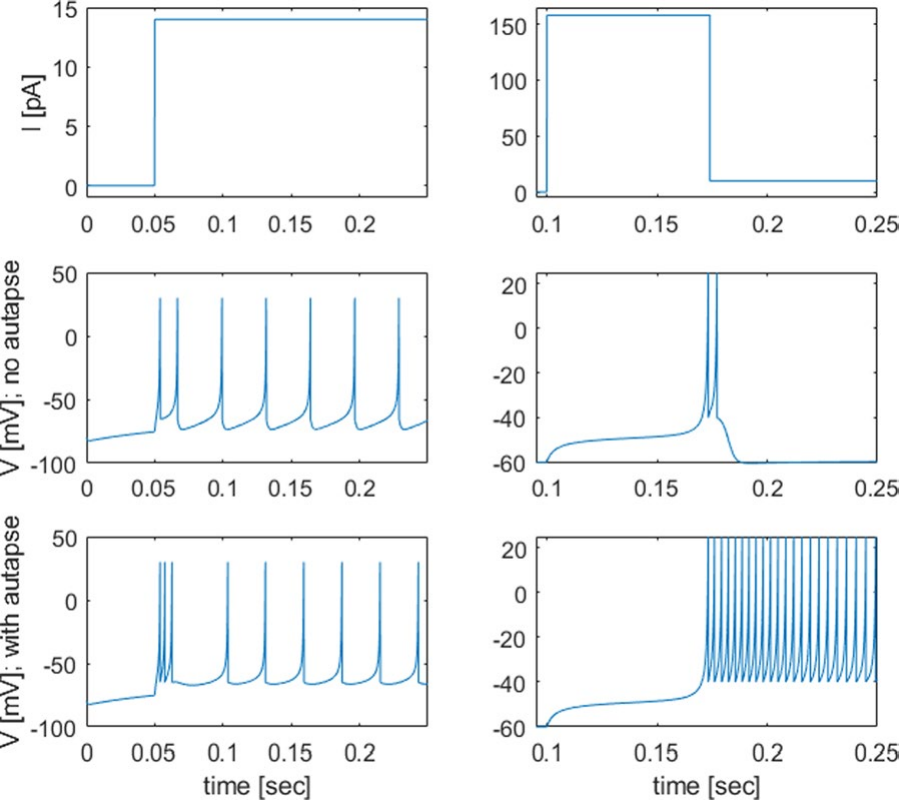
Depending on its parameters, a model neuron with an autapse enhances bursting (left) or leads to persistent activity in absence of strong external excitation (right)

The current contribution presents mathematical analyses of the dynamics of the model, involving numerical simulations, linear stability analysis, Lyapunov exponent estimation and bifurcation analysis. This will lead toward identifying model parameter ranges and bifurcations that underlie transition to bursting, persistent activity, chaos and other neural phenomena the model can support and, by extension, a better understanding of the neuro-computational roles of autapses.

**References**

1. Yin L, Zheng R, Ke W, et al. Autapses enhance bursting and coincidence detection in neocortical pyramidal cells. *Nature Communications* 2018,*9*(4890).
2. Saada R, Miller N, Hurwitz I, et al. Autaptic Excitation Elicits Persistent Activity and a Plateau Potential in a Neuron of Known Behavioral Function. *Current Biology* 2009,*19*(6), 479–484.

**Editor’s note:** This abstract has been retracted. Please handle as discussed with the copy editor.

## P315 A Bayesian model explains how individual and mutual properties of action and outcome affect sense of agency

### Roberto Legaspi, Taro Toyoizumi

#### Rikem Center for Brain Science, Neural Computation and Adaptation, Wakoshi, Saitama, Japan

##### **Correspondence:** Roberto Legaspi (roberto.legaspi@riken.jp)

*BMC Neuroscience* 2019, **20(Suppl 1)**: P315

Although sense of agency (SoA) is a significant concept in the human sciences, the literature still lacks a mathematical elucidation of its underlying principles. SoA, i.e., the experience that oneself initiated an action that caused an outcome, grounds our sense of self and all kinds of causally efficacious self-world interactions mediated by our actions. We have introduced a theoretical model of SoA [1] that adapted a Bayesian inference model originally used to explain the ventriloquism effect as an effort to estimate a common cause behind its multisensory integration. However, we have yet to explain through our model how action and outcome signals, in isolation or interacting with each other, affect SoA.

Our Bayesian model exhibits the following properties for high SoA: (A) the perceived action-outcome effect is consistent (e.g., spatially or temporally) with the causation of the outcome by the action, (B) the prior belief that the action caused the outcome is strong, and (C) the perceived action or outcome signals are reliable when they happen in isolation, i.e., the amplitudes of sensory jitters are small enough not to increase sensory uncertainty. Given that human perception is inherently noisy, the brain resolves ambiguity by drawing on prior expectation of action-outcome consistency, which can be cognitively modulated by prior experience of cause-outcome pairing. These joint priors inform SoA only when precise estimates of action and outcome attributes are shifted towards each other.

The above properties accounted for the intentional binding (IB) effect in two pertinent experiments. IB refers to the perceived compression of the temporal interval of a volitional action and its sensory outcome. The first is the seminal experiment by Haggard et al. [2] that showed voluntary actions produced IB but involuntary actions produced a repulsion effect, i.e., a prolonged opposite perception of the action-outcome interval. Both binding and repulsion effects were accounted for by property (A) of our Bayesian model. The second study is by Wolpe et al. [3] that showed the contribution of sensory uncertainty to IB, which was accounted for by (C). Moreover, as accounted for by (B), the strength of a prior belief of the action causing the outcome moves the perception of the action and outcome towards each other, and weakening it consequently diminishes the action-outcome effect similar to when their signals are unrelated. This accounted for both the repulsion effect from involuntary actions reported by Haggard et al. and the diminished action binding reported by Wolpe et al. However, our model also explains action and outcome binding even for unintended actions given high confidence in the action causing the outcome, which suggests that intentionality is not strictly necessary. Finally, our Bayesian model also explains that if the sensory cues are reliable, SoA can emerge even for unintended actions.

**Acknowledgement**: This study was supported by Brain/MINDS from AMED under Grant Number JP18dm020700 and JSPS KAKENHI Grant Number JP18H05432.

**References**

1. Legaspi R, Toyoizumi T. A Bayesian psychophysics model of sense of agency. *BMC Neuroscience* 2018, 19(suppl 2):P248.
2. Haggard P, Clark S, Kalogeras J. Voluntary action and conscious awareness. *Nature Neuroscience* 2002, 5(4), 383v385.
3. Wolpe N, Haggard P, Siebner HR, Rowe JB. Cue integration and the perception of action in intentional binding. *Experimental Brain Research* 2013, 229, 467–474.

## P316 A map-based model for the membrane potential of healthy and unhealthy neurons and cardiac cells

### Mauricio Girardi-Schappo^1^, Patrick A. Morelo^2^, Rafael V. Stenzinger^2^, Marcelo H. R. Tragtenberg^2^

#### ^1^University of Sao Paulo, Department of Physics FFCLRP, Ribeirao Preto, SP, Brazil; ^2^Federal University of Santa Catarina, Department of Physics, Florianopolis, SC, Brazil

##### **Correspondence:** Mauricio Girardi-Schappo (girardi.s@gmail.com)

*BMC Neuroscience* 2019, **20(Suppl 1)**: P316

A good compromise between conductance-based and integrate-and-fire models is found in map-based neuron or cardiac cell models [1]. Maps describe the actual membrane potential of the neurons, allowing for studies in many levels of detail: from single-cell dynamics to entire networks [1-3]. Recently, we studied a simplified model that captures many different experimental behaviors of single cells: the logistic KTz map [4]. It is computationally efficient and displays from class I and II excitability, to adaptation, to many more dynamical regimes. The model has only five parameters and three dynamic variables, allowing for the computation of all the fixed-point bifurcations analytically [4].

Here, we focus on the dynamics of the logistic KTz map that describes the action potential (AP) of cardiac cells. By tuning its parameters, we may reproduce the activity of many heart cells, including the sinoatrial node, the atrioventricular node, the Purkinje cell, and the myocytes. We characterize the dynamics of three different mechanisms through which the AP of the map shows impaired oscillations: autonomous spikes with early afterdepolarization (EAD), excitable spikes with EAD (related to the long QT syndrome), and nonchaotic aperiodic cardiac spiking. EADs are pathological voltage oscillations during the repolarization of the AP that may disrupt the healthy heart rhythm, ultimately leading to lethal ventricular fibrillation [5].

The healthy regime of autonomous cardiac spiking is bordered by two separate unhealthy regimes: aperiodic cardiac spiking and bursting [4]. Autonomous spikes displaying EAD appear in the model on the frontier between cardiac spikes and bursting. They look like a spike with a plateau immediately followed by a long burst of thin spikes. The Lyapunov exponent in this transition is greater than 1, making this type of oscillations chaotic. The nonchaotic aperiodic cardiac spiking appears when, in the fast subsystem, an unstable node turns into an unstable spiral, causing the orbit to spiral for a few timesteps before the rise of the spike. This mechanism makes the rise of the spike a stochastic event. The EAD in excitable spikes occurs because the fast subsystem displays a subcritical Neimark-Sacker (NS) bifurcation followed closely by a Saddle-Node (SN) of an unstable node causing a stretch of the duration of the spike. When the spike is too long, it encounters the limit cycle of the NS bifurcation, causing the spontaneous depolarization during the repolarization phase of the AP, and delaying the T wave in electrocardiograms.

Our next step is to plug many logistic KTz cells into a lattice to determine how the behavior of an unhealthy cell can disrupt the activity of the entire lattice, causing synchrony and fibrillation. The study of conductance-based models is sometimes too confusing due to both the massive amount of parameters and the complexity of the calculations involved in the determination of its dynamics. Maps turn out to be useful (and essential) in these situations, allowing for an integrative understanding of complex phenomena, like heart and brain diseases. Thus, provided that the bifurcations of simple map neurons and conductance-based models match, the latter may often be replaced by maps.

**Acknowledgements:** MGS, PAM and RVS acknowledge financial support from Brazilian agencies FAPESP, CAPES and FAPESC, respectively.

**References**

1. Girardi-Schappo M, Kinouchi O, Tragtenberg MHR. A brief history of excitable map-based neurons and neural networks. *Journal of Neurosci Methods* 2013, 220(2):116–130.
2. Girardi-Schappo M, Kinouchi O, Tragtenberg MHR. Critical avalanches and subsampling in map-based neural networks coupled with noisy synapses. *Physical Review E* 2013, 88:024701.
3. Izhikevich EM, Edelman GM. Large-scale model of mammalian thalamocortical systems. *PNAS USA* 2008, 105(9): 3593–3598.
4. Girardi-Schappo M, Bortolotto GS, Stenzinger RV, Gonsalves JJ, Tragtenberg MHR. Phase diagrams and dynamics of a computationally efficient map-based neuron model. *PLoS ONE* 2017, 12(3):e0174621.
5. Katz AM. Physiology of the heart, 5th Edition. *Philadelphia: Lippincott Williams & Wilkins*; 2011

## P317 Deterministic search process underlies memory recall

### Michelangelo Naim, Mikhail Katkov, Misha Tsodyks

#### Weizmann Institute of Science, Neurobiology, Rehovot, Israel

##### **Correspondence:** Michelangelo Naim (michelangelonaim@gmail.com)

*BMC Neuroscience* 2019, **20(Suppl 1)**: P317

It is believed that human cognitive abilities could not be theoretically predicted because their neuronal mechanisms are poorly understood. For example, it is hard to imagine how one may predict the performance in memory task. The standard experimental paradigm to address the memory for random material is free recall. Typical experiments involve recalling randomly assembled lists of words in arbitrary order after a brief exposure. It was observed over the years that when the presented list becomes longer, the average number of recalled words grows but in a sublinear way, such that the fraction of words recalled steadily decreases. We have recently proposed a model for recalling random unstructured information that gives an analytical prediction for the number of words that can be recalled without any free parameters to tune. More specifically, the number of recalled items is predicted to be the square root of the number of acquired items. We conjectured that recall performance may be affected by variability in the words acquisition. Therefore, we performed a number of experiments where each participant performed both free recall and recognition experiment. From recall experiment we estimated the number of words recalled, while from recognition experiments we estimated the number of words acquired. The data show a remarkable agreement between theoretical prediction and experimental observations. This level of precision of an analytical model is common for physical theories but is believed to be impossible for biological systems. The result (Fig. 1) provides the first analytical description of a high-level cognitive process. Moreover, the theoretical model is based on neuronal representation of material and potentially may be used to gain a neuron-level understanding of recall process. In conclusion, human memory recall operates according to deterministic search process, which results in a fundamental limit on the number of items that can be successfully recalled.

**Fig. 1:**
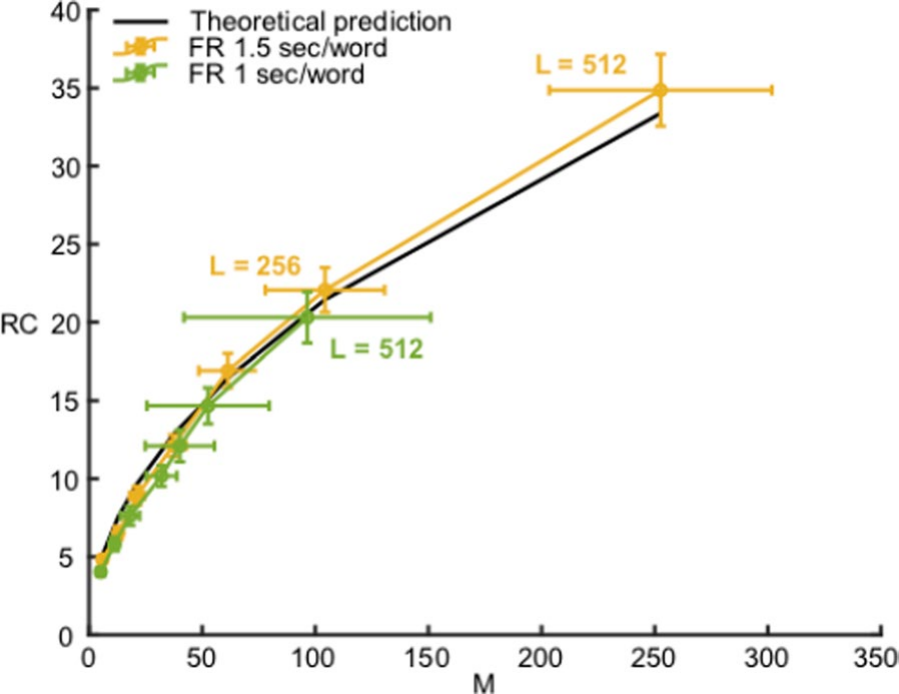
Average number of words recalled (RC) as a function of the average number of acquired words (M) for different list length (L). Black line: theoretical prediction. Yellow line: experimental results for presentation rate 1.5 sec/word. Green line: experimental results for presentation rate 1 sec/word. The error in RC is a standard error of the mean, while the error in M is computed with bootstrap

**Acknowledgments:** This work is supported by the EU-H2020-FET 1564 and EU-M-GATE 765549 and Foundation Adelis.

## P318 Geometry of sensory response extremizes correlations in a phenomenological model of autism

### Rashid Williams-Garcia^1,2^, G. Bard Ermentrout^1^, Nathan Urban^2^

#### ^1^University of Pittsburgh, Department of Mathematics, Pittsburgh, PA, United States of America; ^2^University of Pittsburgh, Department of Neurobiology, Pittsburgh, PA, United States of America

##### **Correspondence:** Rashid Williams-Garcia (rwgarcia@pitt.edu)

*BMC Neuroscience* 2019, **20(Suppl 1)**: P318

Experimental evidence suggests that autistic neural networks exhibit reduced inhibition, weaker synapses, and higher response variability. Using these findings, we develop a phenomenological model of autism to better understand sensory deficits associated with the disorder. In the model, neuronal responses in autistic networks are emulated by weakening the synaptic strength and increasing the neuronal excitability relative to typical model neuron parameter values. Our results agree with empirical findings above a certain firing rate and generate testable predictions about the variability of neuronal responses at different stimulus intensities. To validate these results, we perform analytical calculations of the equal-time cross-correlation and examine the relationship between the stimulus intensity, neuronal excitability, and neuronal response variability. We show that the calculated contour geometry extremizes the cross-correlation, suggesting anunderlying conservation law and variational principlein the study of autism.

## P319 Modeling implicates inhibitory network bistability as an underpinning of seizure initiation

### Scott Rich^1^, Homeira Chameh^1^, Marjan Rafiee^1^, Katie Ferguson^1^, Frances Skinner^1^, Taufik Valiante^2^

#### ^1^Krembil Research Institute, Division of Fundamental Neurobiology, Toronto, Canada; ^2^Krembil Research Institute and University of Toronto, Fundamental Neurobiology; Departments of Surgery (Neurosurgery) and ECE, Toronto, Canada

##### **Correspondence:** Scott Rich (sbrich@umich.edu)

*BMC Neuroscience* 2019, **20(Suppl 1)**: P319

For decades, the study of seizure initiation has focused on either over-excitation or dis-inhibition as the underlying cause of the synchronous and hyper-excitable dynamics hallmarking seizure. However, a variety of recent findings in the experimental literature implicate the synchronous activity of GABAergic interneurons in driving this pathology [1, 3]. Given the novelty of these results, little computational research into a “GABAergic initiation hypothesis” of seizure has been performed. Correspondingly, few mechanisms explaining the predisposition of ictogenic inhibitory networks toward abrupt transitions into synchrony have been articulated.

This research [4] proposes such a mechanism by comparing simulated inhibitory networks containing control interneurons (a modification of an existing PV+ interneuron model [2]) and networks containing hyper-excitable interneurons modeled to mimic treatment with 4-Aminopyridine (4-AP), an agent used to model seizures *in vivo* and *in vitro*. The differences in these models were informed by experimental literature describing the effects of 4-AP [5], as well as in-house experiments recording spiking properties of the same cortical interneuron in both a control and 4-AP setting. The*in silico*study revealed that 4-AP networks are more prone than their control counterparts to exhibit bistability, where the two stable states are sparse, asynchronous firing and faster, synchronous activity. In the 4-AP setting, it is significantly more likely that a perturbation to the network, modeled as a brief synchronizing current pulse, will force an asynchronous network into stable synchronous dynamics. Analogous dynamical changes arise when an Ornstein-Uhlenbeck process modeling background excitatory synaptic dictates the perturbation, indicating that this transition might be feasible*in vivo*. These results propose a mechanism by which a cortically-inspired inhibitory network can shift from incoherent to coherent dynamics, which in turn might initiate seizure according to a “GABAergic initiation hypothesis”. Moreover, this mechanism specifically explains how inhibitory networks containing hyper-excitable, and in turn potentially ictogenic, interneurons can undergo this transition without relying upon a permanent change in the drive to the system. This potentially explains such networks’ increased vulnerability to seizure initiated by GABAergic activity. These mechanistic ideas will be expanded upon via application to excitatory-inhibitory (E-I) network systems based on recordings from human cortical cells.

**References**

1. Chang M. et. al. Brief activation of GABAergic interneurons initiates the transition to ictal events through post-inhibitory rebound excitation. *Neurobiology of disease* 2018 Jan 1;109:102–16.
2. Ferguson, K.A. et. al. Experimentally constrained CA1 fast-firing parvalbumin-positive interneuron network models exhibit sharp transitions into coherent high frequency rhythms. *Frontiers in computational neuroscience* 2013 Oct 22;7:144.
3. Miri ML, Vinck M, Pant R, Cardin JA. Altered hippocampal interneuron activity precedes ictal onset. *Elife* 2018 Nov 2;7:e40750.
4. Rich S. et. al. Modeling implicates inhibitory network bistability as an underpinning of seizure initiation. *Manuscript to be submitted* 2019.
5. Williams SB, Hablitz JJ. Differential modulation of repetitive firing and synchronous network activity in neocortical interneurons by inhibition of A-type K+ channels and Ih. *Frontiers in cellular neuroscience* 2015 Mar 18;9:89.

## P320 High frequency neurons help routing information in brain networks

### Claudio Mirasso^1^, Aref Pariz^1^, Santiago Canals^2^, Alireza Valizadeh^3^

#### ^1^Universitat de les Illes Balears, Instituto de Física Interdisciplinar y Sistemas Complejos, Palma de Mallorca, Spain; ^2^Instituto de Neurociencias de Alicante, Sant Joan d’Alacant, Spain; ^3^Institute for Advanced Studies in Basic Sciences, Zanjan, Iran

##### **Correspondence:** Claudio Mirasso (claudio@ifisc.uib-csic.es)

*BMC Neuroscience* 2019, **20(Suppl 1)**: P320

The emergence of flexible information channels in brain networks is a fundamental question in neuroscience. Understanding the mechanisms of dynamic routing of information would have far-reaching implications in a number of disciplines ranging from biology and medicine to information technologies and engineering.

In this presentation, we study how signals transmit in bidirectionally-coupled networks. In these networks, each node represents a unit composed of excitatory and inhibitory neurons and all nodes, except one, produce a local oscillation with frequency ν0in the gamma range. An inhomogeneity is introduced by placing the remaining node –called source node– to oscillate at a different intrinsic frequency ν = ν0+ Δν. We find that the presence of this particular node leads to reliable transmission of signals and establishes a preferential direction of information flow.

Our results show that slowly varying local signals can better propagate along the network if the receiving node has a higher intrinsic firing rate. Moreover, we find that high frequency units determine the direction of signal propagation, so the effective connectivity in such a network.

To gain insights into the mechanisms that favor the preferable direction of information flow, we study in depth the simple case of two mutually-coupled Hodgkin-Huxley neurons and compare it results with the analytical predictions of two coupled Kuramoto models.

## P321 Hybrid circuits to assess sequential neural rhythms from low dimensional observations

### Irene Elices Ocon^1^, Manuel Reyes-Sanchez^1^, Rodrigo Amaducci^1^, Rafael Levi^2^, Francisco B Rodriguez^1^, Pablo Varona^1^

#### ^1^Universidad Autónoma Madrid, Ingeniería Informática, Madrid, Spain; ^2^University of Southern California, Department of Biological Sciences, Los Angeles, CA, United States of America

##### **Correspondence:** Irene Elices Ocon (irene.elices@uam.es)

*BMC Neuroscience* 2019, **20(Suppl 1)**: P321

Functional sequential activity underlying neural rhythms requires the synergistic interaction of both intrinsic and circuit dynamics. In experimental setups, information of rhythmic activity is obtained through low dimensional observations, i.e., only some aspects of neurons’ rich dynamics are accessible as the number of simultaneous recordings is restricted. On the other hand, variability and flexibility present in living circuits are not captured by commonly used models and since they are usually simplified by reducing the number of neurons or the variables in the model. These restrictions in experimental and computational studies hinder the understanding and assessment of the origin and mechanisms responsible for specific cycle-by-cycle temporal properties of sequential activity.

Hybrid circuits connect living cells to model neurons typically by means of dynamical clamp protocols [1]. Hybrid circuits allow to manipulate parameters or features under study with a similar degree of precision and freedom than in a model, and modulate the living neural circuit dynamics through a more realistic interaction. Thus, they combine the best features of computer modeling and experimental electrophysiology in particular in the context of the study of neural sequential dynamics, and they also provide insight of the origin of such properties.

In this work, we use hybrid circuits to study the origin of cycle-by-cycle temporal relationships between pivotal time intervals that build the sequence of the pyloric CPG rhythm. In particular, we characterized time intervals defined using the LP and PD neuron bursting activity. These temporal relationships are preserved under various conditions, and thus can be identified as dynamical invariants [2]. Hybrid circuits were built by coupling the LP neuron and neuron and graded synapse models using our recently developed RTHybrid software [3, 4]. Results show that dynamical invariants could be interpreted as an intrinsic balance between flexibility and robustness of the neural sequences. We assess the conditions under which the neuron and synapse models provide the minimum elements to build robust invariants in the hybrid circuits. The analysis is also relevant to design models that include such relevant aspects of sequential dynamics. We argue that these invariants can be a universal feature of many sequences shaping neural rhythms in the nervous system.

**Acknowledgements**: Funded by MINECO/FEDER DPI2015-65833-P/TIN2017-84452-R (http://www.mineco.gob.es/) and ONRG grant N62909-14-1-N279.

**References**

1. Prinz AA, Abbott LF, Marder E. The dynamic clamp comes of age. *Trends in Neurosciences* 2004, 27:218–224.
2. Elices I, Levi R, Arroyo D, Rodriguez FB, Varona P. Robust dynamical invariants in sequential neural activity. *BioRxiv* 2018. 10.1101/379909.
3. Amaducci R, Reyes-Sanchez M, Elices I, Rodriguez FB, Varona P. RTHybrid: a standardized and open-source real-time software model library for experimental neuroscience. *Frontiers in Neuroinformatics* 2019;13:11. 10.3389/fninf.2019.0001.
4. Reyes-Sanchez M, Amaducci R, Elices I, Rodriguez FB, Varona P. Automatic adaptation of model neurons and connections to build hybrid circuits with living networks. *BioRxiv* 2018. 10.1101/419622

## P322 Robotic locomotion driven by the flexible rhythm of a living Central Pattern Generator

### Rodrigo Amaducci^1^, Irene Elices Ocon^2^, Manuel Reyes-Sanchez^2^, Rafael Levi^3^, Francisco B Rodriguez^2^, Pablo Varona^2^

#### ^1^Universidad Autónoma de Madrid, Grupo de Neurocomputacion Biologica (GNB), Madrid, Spain; ^2^Universidad Autónoma de Madrid, Ingeniería Informática, Madrid, Spain; ^3^University of Southern California, Department of Biological Sciences, Los Angeles, CA, United States of America

##### **Correspondence:** Irene Elices Ocon (irene.elices@uam.es)

*BMC Neuroscience* 2019, **20(Suppl 1)**: P322

Bio-inspired central pattern generators have been widely used to control robot locomotion, see for review [1]. Some efforts use neurons with intrinsic rich dynamics as observed in several experimental works of CPGs [2, 3]. However, most studies use simplified oscillator paradigms with some bioispiration from the connectivity of living CPGs. We have recently revealed the presence of dynamical invariants in the pyloric CPG in the form of cycle-by-cycle linear relations among specific time intervals and the instantaneous period [4]. Such invariants may underlie effective locomotion programs. In this work we show that flexible robot locomotion with cycle-by-cycle invariants can be directly driven by the living CPG rhythm.

A hexapod robotic platform was built using printable parts from the BQ DIWO PrintBot Crab, whose schematics are open-source. A BQ Zum BT-328 board was used, programmed with Arduino code, with three TG9e microservomotors. Activity from both the LP and PD neurons of the stomatogastric ganglion of a crab was recorded using intracellular electrodes and sent to a computer through a DAQ device. The computer then performed online event detection on the signals and forwarded this information to the robot via Bluetooth connection, accurately preserving the temporal structure of the intervals building the CPG sequence. We tested two different strategies to control the robot. The first one consisted of using the neuron oscillations to drive the robot directly, for example moving the legs when a specific neuron fires. In the second case, the robot was moved by artificial oscillators from the ArduSnake library, whose parameters were set cycle-by-cycle based on the dynamical properties of the living circuit rhythm, such as the instantaneous inter-burst intervals duration. These trials were also carried out using a model CPG rhythms instead of living recordings to compare the robot performance in both cases.

Our results show that a direct translation of the properties that shape the sequences and dynamical invariants of the living CPG can efficiently drive the hexapod robot. Moreover, this platform can also be used to incorporate feedback to the living system by using the sensory information collected by the robot. This will be implemented in future studies to assess the functional role of CPG dynamical invariants.

**Acknowledgments:** This work was supported by MINECO/FEDER DPI2015-65833-P, TIN2017-84452-R and ONRG grant N62909-14-1-N279

**References**

1. Ijspeert AJ. Central pattern generators for locomotion control in animals and robots: a review. *Neural Networks* 2008;21:642–53. 10.1016/j.neunet.2008.03.014
2. Lee YJ, Lee J, Kim KK, Kim Y-B, Ayers J. Low power CMOS electronic central pattern generator design for a biomimetic underwater robot. *Neurocomputing* 2007;71:284–96. 10.1016/j.neucom.2006.12.013
3. Herrero-Carrón F, Rodríguez FB, Varona P. Bio-inspired design strategies for central pattern generator control in modular robotics. *Bioinspiration and Biomimetics* 2011;6:16006. 10.1088/1748-3182/6/1/016006
4. Elices I, Levi R, Arroyo D, Rodriguez FB, Varona P. Robust dynamical invariants in sequential neural activity. *bioRxiv* 2018. 10.1101/37990

## P323 Transfer of rich living circuit dynamics to neuron models through graded synapses

### Manuel Reyes-Sanchez, Rodrigo Amaducci, Irene Elices Ocon, Francisco B Rodriguez, Pablo Varona

#### Universidad Autónoma Madrid, Ingeniería Informática, Madrid, Spain

##### **Correspondence:** Manuel Reyes-Sanchez (manuel.reyes@uam.es)

*BMC Neuroscience* 2019, **20(Suppl 1)**: P323

While in most neurons propagation of neural signaling occurs via all-or-none action potentials, graded synapses are also present in many nervous circuits such as the pyloric CPG of crustacean [1, 2]. We have recently discovered the presence of dynamical invariants in the form of preserved cycle-by-cycle relationships between specific time intervals of the rhythm and the instantaneous period [3]. In this work, we assess how the parameters of a graded synapse can influence the propagation of rich dynamics of living circuits to neuron models.

We performed long recordings of the sequential activations of the LP and PD neurons corresponding to several waveform patterns and frequencies. The time series of each living neuron was used to drive the input to a set of neuron models through a graded chemical synapse using the RTHybrid software [4]. The automatic amplitude, temporal scaling and adaptation of the living neuron signals to each model’s working regime was performed by the algorithms described in [5]. We then mapped the regions of the parameter space that produced a linear relationship between the instantaneous period and the intervals defining the sequence of this hybrid circuit. In these high resolution maps, the linear relationship was quantified by the calculation of the R2 of the regression.

The analysis of the maps generated from these hybrid circuits shows that there are regions of the synaptic parameter space that allow the transfer of the dynamics that give rise to the dynamical invariants between the living and the model neurons. The maps help identifying the specific role of synaptic parameters in sustaining the invariants. They show that not only the expected maximum conductance, but also the threshold of the graded release and the time constants that control the waveform and dynamics of the synapse affect the existence of the invariant. Furthermore, the maps can also be used to study how the features of the neuron model influence the presence and quality of the invariant.

**Acknowledgements**: Funded by MINECO/ FEDER DPI2015-65833-P, TIN2017-84452-R and ONRG grant N62909-14-1-N279.

**References**

1. Graubard K, Raper JA, Hartline DK. Graded synaptic transmission between identified spiking neurons. *J Neurophysiol* 1983;50:508–21. 10.1152/jn.1983.50.2.508.
2. Kurtz R, Warzecha A-K, Egelhaaf M. Transfer of Visual Motion Information via Graded Synapses Operates Linearly in the Natural Activity Range. *J Neurosci* 2001;21:6957 LP- 6966. 10.1523/jneurosci.21-17-06957.2001.
3. Elices I, Levi R, Arroyo D, Rodriguez FB, Varona P. Robust dynamical invariants in sequential neural activity. *BioRxiv* 2018. 10.1101/379909.
4. Amaducci R, Reyes-Sanchez M, Elices I, Rodriguez FB, Varona P. RTHybrid: a standardized and open-source real-time software model library for experimental neuroscience. Frontiers in *Neuroinformatics* 2019;13:11. 10.3389/fninf.2019.0001.
5. Reyes-Sanchez M, Amaducci R, Elices I, Rodriguez FB, Varona P. Automatic adaptation of model neurons and connections to build hybrid circuits with living networks. *BioRxiv* 2018. 10.1101/419622.

## P324 Lost order and rhythm in a mouse model for neurodegenerative motor neuron disease

### Soju Seki^1^, David H Terman^2^, Antonios Pantazis^3^, Riccardo Olcese^4^, Martina Wiedau-Pazos^5^, Scott H Chandler^1^, Sharmila Venugopal^1^

#### ^1^University of California Los Angeles, Integrative Biology and Physiology, Los Angeles, United States of America; ^2^The Ohio State University, Department of Mathematics, Columbus, OH, United States of America; ^3^Linköping University, Clinical and Experimental Medicine, Linköping, Sweden; ^4^University of California, Los Angeles, Departments of Anesthesiology and Physiology, Los Angeles, CA, United States of America; ^5^University of California, Los Angeles, Department of Neurology, Los Angeles, CA, United States of America

##### **Correspondence:** Sharmila Venugopal (svenugopal10@gmail.com)

*BMC Neuroscience* 2019, **20(Suppl 1)**: P324

Neurodegenerative diseases involve a protracted pre-symptomatic phase, during which, many structural, functional and molecular abnormalities precede imminent neuronal loss and overt symptoms. Our research focuses on understanding the cellular and molecular dynamics of disease development using experimental and computational approaches. Here, we report early abnormalities of brainstem sensorimotor neurons controlling jaw function, in a mouse model for neurodegenerative Lou Gehrig’s disease, also known as Amyotrophic Lateral Sclerosis (ALS). In this fast-progressing, devastating disease, alpha motor neurons (MNs) degenerate, resulting in muscle atrophy, paralysis and death. Complementing our previous report on early hyperexcitability of MNs1, and recent evidence that proprioceptive inputs to alpha-MNs could play a trigger role in the neurodegenerative process2, we directly tested whether proprioceptive sensory neurons are abnormal early during disease development. Using diverse approaches we establish early circuit-specific proprioceptive sensory abnormalities in a well-characterized Super-Oxide-di-Mutase-1 (SOD1) transgenic mouse model for ALS at postnatal age P11±3. Our results suggest circuit-specific early dysregulation of proprioceptive neurons and predicts consequent sensorimotor dysfunction in ALS.

## P325 Do human brain functional networks obey power law scaling?

### Riccardo Zucca^1^, Xerxes Arsiwalla^2^, Hoang Le^3^, Mikail Rubinov^4^, Paul Verschure^1,5^

#### ^1^IBEC, SPECS, Barcelona, Spain; ^2^Institute for Bioengineering of Catalonia, Barcelona, Spain; ^3^California Institute of Technology, Pasadena, United States of America; ^4^Vanderbilt University, Department of Biomedical Engineering, Vanderbilt, United States of America; ^5^Catalan Institute of Advanced Studies (ICREA), Barcelona, Spain

##### **Correspondence:** Riccardo Zucca (rzucca@ibecbarcelona.eu)

*BMC Neuroscience* 2019, **20(Suppl 1)**: P325

Do human brain functional connectivity networks obey power law scaling in their degree distributions? Initial claims to the affirmative, based on least-squares fitting, have been challenged on methodological grounds [1, 2]. Subsequently, estimators based on maximum-likelihood and non-parametric tests involving surrogate data have been proposed [2]. However, no clear consensus has emerged as results have varied across studies [3], especially, showing a dependence on the resolution of the data used [4]. Addressing this question calls for a closer examination of methodological issues. In this study, we analyze high-resolution fMRI data from the Human Connectome Project at six different resolutions: 1, 5, 10, 20, 50 and 80 thousand regions of interest. We test for the power law, exponential, log normal, Weibull and generalized Pareto distributions. Our results show that the statistics generally do not support a power law. These degree distributions are not fat-tailed. Instead, they tend towards the short-tailed limit of the generalized Pareto model. This has clear implications for the organization of hubs in human fMRI data. Furthermore, working across several resolutions of the data and performing cross-model comparisons, we establish robustness of the generalized Pareto model in explaining the data. An interesting by-product of this analysis is the observation that down-sampling the data affects statistical tests in a systematic manner. The statistical plausibility of every model systematically increases up to a limit. Lower resolutions make it harder to discern between models, leading to the appearance of multiple models being significant. This is why one sees more power laws there than at higher resolutions, but those tests fail cross-model comparisons. This is particularly relevant for studies that involve down-sampling fMRI data into anatomical parcellations. The down-sampling effects we report here bear significance for the broader discussion of scientific reproducibility.

**Acknowledgments:** This work has been supported by the ERC (341196) to PV. The data used in this study was made available by the Human Connectome Project, WU-Minn Consortium (PIs: D. Van Essen and K. Ugurbil; 1U54MH091657)

**References**

1. Barabási AL. Scale-free networks: a decade and beyond. *Science* 2009 Jul 24;325(5939):412–3.
2. Broido AD, Clauset A. Scale-free networks are rare. *Nature communications* 2019 Mar 4;10(1):1017.
3. Zucca R, Arsiwalla XD, Le H, Rubinov M, Verschure PF. Scaling properties of human brain functional networks. *In International Conference on Artificial Neural Networks* 2016 Sep 6 (pp. 107–114). Springer, Cham.
4. Zalesky A, Fornito A, Harding IH, et al. Whole-brain anatomical networks: does the choice of nodes matter? *Neuroimage* 2010 Apr 15;50(3):970–83.

## P326 Self beyond the body: self-generated task-relevant distal cues modulate performance and the body ownership

### Klaudia Grechuta^1^, Laura Ulysse^1^, Belen Rubio^2^, Paul Verschure^3^

#### ^1^Universitat Pompeu Fabra (UPF), Barcelona, Spain; ^2^Institute for Bioengineering of Catalonia (IBEC), Barcelona, Spain; ^3^Institute for BioEngineering of Catalonia (IBEC), Catalan Institute of Advanced Studies (ICREA), SPECS Lab, Barcelona, Spain

##### **Correspondence:** Laura Ulysse (lau.ulysse@gmail.com)

*BMC Neuroscience* 2019, **20(Suppl 1)**: P326

Body Ownership (BO) was originally considered a sensory state deriving reactively as a consequence of bottom-up integration of multi-sensory cues when exafferent inputs are presented synchronously [1]. Interestingly, recent evidence shows that BO is also actively modulated by top-down prediction of both externally [2] and self-generated sensory events [3]. Thus, BO seems to be a product of bottom-up integration and top-down prediction of both self and externally-generated sensory events, which occurs when sensory prediction errors are low [4, 5, 6]. In motor control, Forward Models (FM) driving goal-oriented behavior integrate any task-relevant sensory cues to minimize sensory prediction errors and optimize performance with the world, however, inevitably triggers processing of not only proximal (TRPC)(i.e. proprioceptive)but also distal (TRDC) (i.e. vision, audition)task-relevant cues, and these, in turn, might influence TRPC establishing a feedback loop [7].In that direction, Forward Models (FM) which leads goal-oriented behavior will combine each sensory modality that pertains to the goal at hand and the performance of an embodied self. The study aimed to find if BO depends on the consistency of FMs driving goal-oriented action, where congruent relationship between TRPC and TRDC influence BO and even define the borders of the embodied self. To do so, we created an embodied VR-based goal-oriented task where action outcomes were driven by diverse auditory cues. By manipulating the cues with respect to their spatiotemporal congruency and valence, we show that action-driven distal feedback (TRDC) which breaks predictions about the environment settle both BO and performance. Our results demonstrate that feedback cues related to not only the body itself but also by feedback around it within a task might influence BO.

**References**

1. Botvinick M, Cohen J. Rubber hands ‘feel’ touch that eyes see. *Nature* 1998 Feb;391(6669):756.
2. Ferri F, Chiarelli AM, Merla A, Gallese V, Costantini M. The body beyond the body: expectation of a sensory event is enough to induce ownership over a fake hand. *Proceedings of the Royal Society B: Biological Sciences* 2013 Aug 22;280(1765):20131140.
3. Dummer T, Picot-Annand A, Neal T, Moore C. Movement and the rubber hand illusion. *Perception* 2009 Feb;38(2):271-80.
4. Blanke O. Multisensory brain mechanisms of bodily self-consciousness. *Nature Reviews Neuroscience* 2012 Aug;13(8):556.
5. Seth AK. Interoceptive inference, emotion, and the embodied self. *Trends in cognitive sciences* 2013 Nov 1;17(11):565–73.
6. Apps MA, Tsakiris M. The free-energy self: a predictive coding account of self-recognition. *Neuroscience & Biobehavioral Reviews* 2014 Apr 1;41:85–97.
7. Miall RC, Wolpert DM. Forward models for physiological motor control. *Neural networks* 1996 Nov 1;9(8):1265–79.

## P327 Integrated information as a measure of volitional control in intracranial functional connectivity networks

### Xerxes Arsiwalla^1^, Daniel Pacheco^2^, Paul Verschure^3^

#### ^1^IBEC, Barcelona, Spain; ^2^IBEC, Neural Engineering, Barcelona, Spain; ^3^Institute for BioEngineering of Catalonia (IBEC), Catalan Institute of Advanced Studies (ICREA), SPECS Lab, Barcelona, Spain

##### **Correspondence:** Xerxes Arsiwalla (xarsiwalla@ibecbarcelona.eu)

*BMC Neuroscience* 2019, **20(Suppl 1)**: P327

An important challenge in computational neuroscience is to quantify information complexity [1] of the brain at different temporal and spatial scales, especially given the surge in high-throughput imaging and recording technologies. The question we address here is: How can we quantify temporal informational complexity of the brain to distinguish states of volition? To investigate this, we work with a new formulation of network integrated information that is based on the Kullback-Leibler divergence between the multivariate distribution on the set of network states versus the corresponding factorized distribution over its parts [2, 3]. We extend this formulation for temporal networks and then apply it to human brain data obtained from intracranial recordings in epilepsy patients. We test subjects on a virtual-reality based navigation task, where one group performs active exploration of visual information, while the other passive. Computing the temporal integrated information on these datasets shows that our measure captures statistical differences in volitional versus passive navigation modes. Moreover, compared to random re-wirings, functional connectivity networks constructed from human brain data, score consistently higher in the above measure of integrated information. This work suggests that temporal integrated information may indeed function as a useful measure of volition and more generally cognitive complexity.

**References**

1. Tononi G, Boly M, Massimini M, Koch C. Integrated information theory: from consciousness to its physical substrate. *Nature Reviews Neuroscience* 2016 Jul;17(7):450.
2. Arsiwalla XD, Verschure PF. The global dynamical complexity of the human brain network. *Applied network science* 2016 Dec;1(1):16.
3. Arsiwalla XD, Verschure PF. Integrated information for large complex networks. *In The 2013 International Joint Conference on Neural Networks (IJCNN)* 2013 Aug 4 (pp. 1–7). IEEE.

## P328 Learning joint attention in reinforcement learning agents through intrinsic rewards

### Oriol Corcoll Andreu^1^, Abdullah Makkeh^1^, Dirk Oliver Theis^1^, Raul Vicente Zafra^1^

#### ^1^Tartu University, Institute of Computer Science, Tartu, Estonia

##### **Correspondence:** Oriol Corcoll Andreu (oriol.corcoll.andreu@ut.ee)

*BMC Neuroscience* 2019, **20(Suppl 1)**: P328

Humans and other species face the challenge of navigating through complex social interactions. They critically depend on developing appropriate social interactions with their conspecifics for survival, reproduction, and even regulation of their own body physiology [1]. Recent evidence from neuroimaging and developmental studies indicates that human’s social competencies are learnt and developed from experience with other individuals of their social group (e.g. caregivers) through a joint agency [2]. In particular, the ability of individuals to focus on a common goal (joint attention) and share psychological states (intention sharing) have been proposed to be foundational skills for many of our social competencies like theory of mind, and support general cognitive development [2].

In this work we address the question of how joint attention develops between agents during social cooperation. In particular, we employ causal influence and information measures in a deep reinforcement learning setting to study how intrinsic rewards (in addition to the task’s extrinsic rewards) guide agents to detect and manipulate the attention of other agents to achieve their own goals. For this purpose, we use environments inspired by social dilemmas, including Stag-Hunt, with a rich variety of goals that promote the specialisation of agents in particular tasks, and where joint attention is an advantageous skill to learn. To characterise the learning process we measure the degree of joint attention achieved by different agents using counterfactual methods [3] and asses the importance of this skill at different stages of learning using evolution-inspired algorithms such as population-based training [4]. Finally, based on our studies, we discuss the conditions that are necessary for joint attention to emerge between computational agents and how it can be used to develop further social skills.

**References**

1. Atzil S, Gao W, Fradkin I, Barrett LF. Growing a Social Brain. *Nature Human Behaviour* 2018 Aug 6:1.
2. Tomasello M. Becoming Human: A theory of ontogeny. Belknap Press; 2019 Jan 7.
3. Pearl J, Mackenzie D. The Book of Why: the New Science of Cause and Effect. *Basic Books*; 2018 May 15.
4. Jaderberg M, Dalibard V, Osindero S, et al. Population based training of neural networks. *arXiv preprint* arXiv:1711.09846. 2017 Nov 27.

## P329 Desperately seeking a computational model for wide-cognition approaches

### Oscar Vilarroya

#### Universitat Autonoma de Barcelona, Barcelona, Spain

##### **Correspondence:** Oscar Vilarroya (oscar.vilarroya@gmail.com)

*BMC Neuroscience* 2019, **20(Suppl 1)**: P329

The brain, as any other biological system, is an open system in interaction with the environment, which means, among other things, that there is an interchange of matter and energy between the brain and the environment. However, as a cognitive system, the brain is generally considered to be a closed system. There is a traditional characterization of neural systems as cognitively closed, where cognition must be understood as an internal process. According to these views, cognition is created by intrinsic processes within the brain. In contrast with this approach, insights from different lines of research have emphasized the view of cognition as a coupled continuum between the neural system and the environment. Some authors have used the term “wide cognition” (Milkowski et al., 2018) to characterize such views. According to the wide-cognition approaches, which I have explored elsewhere (Vilarroya, 2012, 2014, 2017), the neural system should be considered to be linked with an external element of the environment, creating a “coupled” system that can be seen as a cognitive unit in its own right. All the components in such a system play an active causal role, and together they govern behavior. If we remove the external component, the system’s behavioral competence decline, just as it would do so if we removed part of the neural system. The assumption is that such a coupled process must be characterized as a genuine cognitive computation, whether or not it is wholly part of the neural system. In other words, the environment should be considered to have not only an active role in the internal activity of the neural system, but as a necessary part of what enables cognition. As far as I have been able to assess, there are few computational approaches that model wide-cognition approaches. It is true, that there are computational models of, for example, object grasping that have begun to integrate the environment in their formalization. However, we need an alternative view that does not require encoding the bodily and environmental elements. Such an alternative view should assume parameters that are environmentally maintained, by relying on the use of the properties of external elements without internally coding for them. My aim with this communication is to open a discussion with participants at the CNS*2019 in order to discuss the possible approaches that could model the wide-cognition view. More specifically, my aim is to be able discuss the possibility of a computational model that can rely on properties of the environment without coding for them. My goal is to use such a proposal to assess the possibility of modelling the cognitive properties of a minimal cognitive system, as well as to provide evidence that such a model shows some of the core properties of a cognitive system, such as learning, generalization, flexibility and context-sensitivity.

## P329 Catecholaminergic contributions to asynchronous broadband activity driven by top-down attention in the human cortex

### Vicente Medel^1^, Samy Castro^2^, Martín Irani^1^, Brice Follet^1^, Jean-Philippe Lachaux^3^, Nicolás Crossley^1^, Tomás Ossandón^1^

#### ^1^Pontificia Universidad Católica de Chile, Psychiatry, Santiago, Chile; ^2^Universidad de Valparaíso, CINV, Valparaiso, Chile; ^3^Inserm U1018, Lyon, France

##### **Correspondence:** Vicente Medel (vimedel@uc.cl)

*BMC Neuroscience* 2019, **20(Suppl 1)**: P330

Brain fluctuations can be indexed in the spectrum of the field potentials by the asynchronous background activity—possibly representing the “noise” of cortical neurons-. It has been shown that cortical computation under neuromodulatory control affect intrinsic variability. In macro scale, it has been shown that by pharmacology, catecholamine modulates fMRI network dynamics in a spatially distributed manner, depending on the its receptors density.

Brain fluctuations have a distributed “scale-free” behaviour, where power scales by frequency, according to a power law, P(f) ∝ fβ. This asynchronous component has been suggested to reflect background noise and catecholaminergic modulation and it has been proposed as a correlate of the BOLD signal. This makes asynchronous activity a candidate to link micro and macro scale dynamics with plausible mechanism. Here, we extend the spatial distribution of catecholaminergic network modulations seen in fMRI to intracortical field potential during a top-down visuospatial working memory task.

We will show that the slope of the power spectrum covaries differently depending on attentional recruitment (see Fig. 1). By correlating spatial distribution of asynchronous activity with catecholaminergic receptor densities, we show regions under neuromodulatory control of intrinsic background activity.

**Fig. 1:**
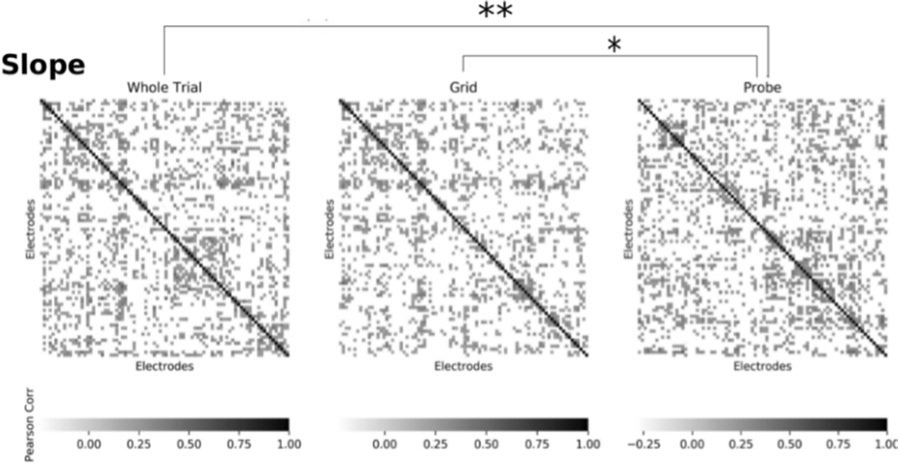
PSD slope covariance matrices of 86 electrodes taken pairwise. Significative differences using t-test were found between whole trial and top-down attention (probe) conditions (p<0.005) and between top-down attention (probe) and rest (grid) conditions (p<0.05)

## P330 Not all dendrites are equal: location-dependent dendritic integration of inputs

### Everton Agnes, Chaitanya Chintaluri, Tim P. Vogels

#### University of Oxford, Centre for Neural Circuits and Behaviour, Oxford, United Kingdom

##### **Correspondence:** Everton Agnes (everton.agnes@cncb.ox.ac.uk)

*BMC Neuroscience* 2019, **20(Suppl 1)**: P331

Neurons often feature complex dendritic morphology that host intricate connectivity motifs. These compartmentalised structures are thought of as independent functional units, and recent experimental and computational studies have helped to elucidate the impact and function of dendritic dynamics on somatic activity [1-3]. However, dendritic responses to complex input patterns have still not been explored in detail. Specifically, we still do not fully understand how slow and fast synaptic currents interact in response to varying spike trains, and how single neurons can perform pattern detection on the many spike patterns that it experiences throughout the day. Towards this goal, we build a simplified dendritic compartment model with conductance-based synapses such that we can quantify how dendrites integrate synaptic inputs under various conditions. In our model, the dendritic dynamics results from synaptic input patterns and the strength of their coupling to the soma, i.e., electrotonic distance. To assess voltage responses, we explore the impact of distinct input spike train statistics: uniform Poisson, non-uniform Poisson with bursts, and non-uniform Poisson following an Ornstein-Uhlenbeck process. Additionally, we test how correlated excitatory and inhibitory spike trains affect excitatory-inhibitory balance, and dendritic responses. We find that four parameters can diversify the dendritic dynamics: soma-dendrite coupling strength (or distance); number of synapses connected onto the dendritic region; ratio between synapse types; and correlation between excitatory and inhibitory inputs. The first is related to the neuronal morphology, and the latter three to connectivity motif (branch location). We show that inhibition tightly balances excitation even for uncorrelated excitatory and inhibitory inputs when enough incoming synapses are present, and that non-linear integration of inputs allows for the emergence of up- and down-states [4], that depends on the soma-dendrite coupling. Furthermore, we show that, depending on the correlation between excitatory and inhibitory inputs, the dendrite responds to either the onset or the offset of a strong stimulation (burst), like ON and OFF cells [5], which also depends on its coupling to the soma. Our work strengthens the hypothesis that dendritic branches can be understood as computational units that give rise to diverse responses based on the location, and synaptic configuration of its input.

**Acknowledgements**: This project was supported by a Leverhulme Trust Project grant (RPG-2016-446; EJA), a BBSRC grant (BB/N019512/1; CC), and a Sir Henry Dale Wellcome Trust and Royal Society grant (WT100000; TPV).

**References**

1. Poirazi P, Brannon T, Mel, BW. Pyramidal neuron as two-layer neural network. *Neuron* 2003, 37, 989–999.
2. Schmidt-Hieber C, Toleikyte G, Aitchison L, et al. Active dendritic integration as a mechanism for robust and precise grid cell firing. *Nature Neuroscience* 2017, 20, 1114–1121.
3. Ujfalussy BB, Makara JK, Lengyel M, Branco T. Global and Multiplexed Dendritic Computations under In Vivo-like Conditions. *Neuron* 2018, 100, 579–592.
4. Cheng-yu TL, Poo M, Dan Y. Burst spiking of a single cortical neuron modifies global brain state. *Science* 2009, 324, 643–646.
5. Qin L, Chimoto S, Sakai M, et al. Comparison between offset and onset responses of primary auditory cortex ON-OFF neurons in awake cats. *Journal of Neurophysiology* 2007, 97, 3421–3431.

## P331 Codependent synaptic plasticity: interacting synapses for stable learning in spiking networks

### Everton Agnes, Tim P. Vogels

#### University of Oxford, Centre for Neural Circuits and Behaviour, Oxford, United Kingdom

##### **Correspondence:** Everton Agnes (everton.agnes@cncb.ox.ac.uk)

*BMC Neuroscience* 2019, **20(Suppl 1)**: P331

Synaptic plasticity is the main mechanism for learning, allowing for versatile synaptic changes that are required to shape the rich variety of cortical dynamics and, consequently, define its functional flexibility [1]. Classically, plasticity is thought to be Hebbian, i.e., a local phenomenon in which changes in synaptic weights depend solely on pre- and post-synaptic quantities [2]. Recently, plasticity has been shown to be modulated by additional pre-synaptic activity, e.g., from neighbouring synapses connecting onto the same post-synaptic dendritic region [3]. It is therefore plausible to re-think the concept of locality and consequently expand it from synapse-specific to dendritic–specific. Furthermore, the functional consequences of this extra modulation are unexplored due to lack of models that incorporate the direct interaction among synapses, especially different synaptic types. Here we present a model of codependent synaptic plasticity including this direct interaction. Based on experimental data, our model adjusts its synapses so that the total excitatory inputs onto a dendritic region is the desired fixed-point; different from the standard firing-rate fixed-point dynamics. Inhibitory plasticity has a complementary role that is also independent of post-synaptic firing-rate. GABAergic synapses change so that the total synaptic input onto a dendritic region is precisely balanced. Together, excitatory and inhibitory synapses dictate the state of excitability of the post-synaptic neuron, while slow homeostatic processes could control its activity, without any unstable dynamics. Additional flexibility is achieved by the modulation of the learning rate of excitatory plasticity via inhibitory inputs. Codependent excitatory plasticity learning-rate is directly affected by inhibitory input currents, which creates a powerful control of “when” to learn through disinhibition. We show that, when connected in a simplified feed-forward network, a post-synaptic neuron dynamically modifies its receptive field-like input weights due to this disinhibitory effect, as reported for auditory cortex [4]. When connected in a recurrent network we show that, by controlling the total excitability of neurons, the network learns to be highly sensitive to external inputs, producing strong transient dynamics, similarly to what is seen in motor cortex [5]. Finally, we show that synaptic clustering emerges naturally in dendrites, as a function of initial number of correlated synapses and distance to the soma. Codependent plasticity is the next step in plasticity modelling by including synaptic interactions for efficient, quick and stable learning of multiple brain functions.

**Acknowledgements**: This project was supported by a Leverhulme Trust Project grant (RPG-2016-446; EJA) and a Sir Henry Dale Wellcome Trust and Royal Society grant (WT100000; TPV).

**References**

1. Poo MM, Pignatelli M, Ryan TJ. What is memory? The present state of the engram. *BMC Biology* 2016, 14, 40.
2. Markram H, Gerstner W, Sjostrom PJ. A history of spike-timing-dependent plasticity. *Frontiers in Synaptic Neuroscience* 2011, 3, 4.
3. Hennequin G, Agnes EJ, Vogels TP. Inhibitory Plasticity: Balance, Control, and Codependence. *Annual Reviews Neuroscience* 2017, 40, 557–579.
4. Froemke RC, Merzenich MM, Schreiner CE. A synaptic memory trace for cortical receptive field plasticity. *Nature* 2007, 450, 425–429.
5. Churchland MM, Cunningham JP, Kaufman MT, et al. Neural population dynamics during reaching. *Nature* 2012, 487, 51–56.

## P332 A canonical model for explaining heterogeneous behavior in perceptual decision making

### Genis Prat-Ortega^1^, Klaus Wimmer^2^, Tobias Donner^3^, Alex Roxin^4^, Jaime de la Rocha^5^, Cristina Pericas^1^, Niklas Wilming^6^

#### ^1^IDIBAPS, Cortical circuits Dynamics, Barcelona, Spain; ^2^Centre de Recerca Matemàtica, Computational Neuroscience Group, Barcelona, Spain; ^3^University Medical Center Hamburg-Eppendorf/University of Amsterdam, Department of Neurophysiology & Pathophysiology/Department of Psychology, Hamburg/Amsterdam, Germany; ^4^Centre de Recerca Matemàtica, Barcelona, Spain; ^5^IDIBAPS, Theoretical Neurobiology, Barcelona, Spain; ^6^UKE, Hamburg, Germany

##### **Correspondence:** Genis Prat-Ortega (gprat@crm.cat)

*BMC Neuroscience* 2019, **20(Suppl 1)**: P332

Our brains interpret ambiguous streams of information to make decisions and guide our behavior. Canonical approaches to model this cognitive function are based on diffusion processes that assume bounded or unbounded perfect integration of the stimulus [1]. Here we study the integration process in neurobiological models with winner-take-all dynamics that can be reduced to a diffusion process of the decision variable x in a double well (DW) potential: dx/dt = -dφ/dX+S(t) with φ = -aX2+bX4 where the stimulus is a gaussian signal (S(t) = μ+ ση(t)) with mean μ and std. deviation σ [2, 3].

To show the key mechanisms that differentiate this model from canonical ones, we characterize the integration process by quantifying the shape and magnitude of the Psychophysical Kernel (PK) . The PKs allow us to characterize the time course of the stimulus impact in both psychophysics experiments and models of perceptual decision making. With this approach and increasing the magnitude of the stimulus fluctuations (σ), we find different integration regimes: i) For small σ, there is transient integration (i.e. primacy) because once the system reaches one well, there are no transitions. iii) For medium σ, we find flexible categorization as initial decisions can be reversed by the stimulus fluctuations (changes of mind). In this regime, the stimulus can impact the choice during the whole trial iii) When σ is large, it can drive several transition between wells. Thus the first visited attractor is irrelevant for the decision and only the late fluctuations in the trial can have an impact on the choice, i.e. the integration is leaky (recency).

We test this prediction in a visual discrimination task (N = 16 humans) and in an auditory discrimination task (N = 5 rats). We find that subjects show heterogeneous psychophysical kernels from primacy to recency. To quantify these different strategies we develop an algorithm to fit a general potential to each subject φ = -aX2+bX4+cX6. Adding a third term allows the potential to take different shapes, from a double well shape to shapes that allow bounded or unbounded nearly perfect integration of the evidence. We test the generality and power of the algorithm using synthetic data. First, we confirm that the algorithm recovers the original parameter set used to generate the data. Second, we can include different mechanisms in the algorithm and test whether they improve the fit to the data. Such mechanisms include an urgency signal, variability at the initial condition or a bias towards one of the sides. This algorithm could be a powerful tool to find the neural mechanisms that explain subject heterogeneity.

**References**

1. Gold JI, Shadlen MN. The neural basis of decision making. *Annual Reviews in Neuroscience* 2007 Jul 21;30:535–74.
2. Wang XJ. Probabilistic decision making by slow reverberation in cortical circuits. *Neuron* 2002 Dec 5;36(5):955–68.
3. Roxin A, Ledberg A. Neurobiological models of two-choice decision making can be reduced to a one-dimensional nonlinear diffusion equation. *PLoS Computational Biology* 2008 Mar 28;4(3):e1000046.

## P333 Prefrontal circuit specialization underlying task-triggered changes in population codes in spatial working memory

### Nicolás Pollán Hauer^1^, Joao Barbosa^2^, Adrià Galán^2^, Bijan Pesaran^3^, Christos Constantinidis^4^, Gianluigi Mongillo^5^, Albert Compte^6^, Klaus Wimmer^1^

#### ^1^Centre de Recerca Matemàtica, Computational Neuroscience Group, Barcelona, Spain; ^2^Institut d’Investigacions Biomèdiques August Pi i Sunyer, Theoretical Neurobiology, Barcelona, Spain; ^3^Center for Neural Science, New York Univ., New York, United States of America; ^4^Wake Forest Univ., School of Medicine, Winston Salem, NC, United States of America; ^5^Paris Descartes, CNRS, Neurophysics and Physiol, Paris, France; ^6^IDIBAPS, Systems Neuroscience, Barcelona, Spain

##### **Correspondence:** Nicolás Pollán Hauer (npollan@crm.cat)

*BMC Neuroscience* 2019, **20(Suppl 1)**: P333

Neural population activity recorded from primate prefrontal cortex carries information about the remembered stimulus, maintaining a stable representation throughout the delay period of working memory tasks. During cue presentation and in the beginning of the delay period the population code is dynamic [3]. Individual prefrontal neurons show heterogeneous activity including strong temporal dynamics in all task phases. It remains unresolved how individual neurons support the dynamic and stable population codes and what are the underlying circuit mechanisms.

Here we set out to investigate how prefrontal neurons with different trial-period dynamics contribute to population dynamics during an oculomotor delayed response task from two datasets [1, 2]. We used linear decoders on single neurons and compared stimulus information during different time points through the whole trial period. We identified a subpopulation of neurons with stable delay decoding at the level of individual neurons and those neurons were the main contributors to the stable population code during the delay. We consistently found stable population decoding, as accurate as the whole population, even for small neuronal subpopulations. These findings are consistent with attractor dynamics.

However, as observed previously (e.g. [3]), the population code and the underlying neuronal firing rates show transient dynamics during cue presentation and in the early delay, apparently at odds with attractor dynamics. We show that this can be explained by a circuit model composed of two coupled ring models. External input impinges on a ring model with weaker recurrence, which is in turn coupled to a second ring model with stronger recurrence that generates the sustained code. The model predicts the existence of two distinct neuronal subpopulations: Firing rates of neurons in the first ring quickly increase in response to the stimulus and decay relatively quickly after stimulus offset (~100 ms). Firing rates in the second ring show a transient increase in firing rate during the cue and early delay periods before the network reaches a stable fixed point.

The experimental data [2] provides support for this model. Changes of the firing rate of functionally different neurons (cue-responsive vs. delay-responsive) are characterized by different time constants. Cue-responsive neurons show faster firing rate dynamics with a narrow distribution of time constants, and the time constants of the delay-responsive population are higher on average. They are also more widely distributed due to ramping activity until the end of the delay period in a fraction of neurons. Finally, an analysis of spike cross-correlation of pairs of simultaneously recorded neurons reveals stronger synaptic coupling within than across the cells of each subpopulation, thus supporting the notion of specialized circuits.

In sum, our findings suggest that the presence of highly dynamic activity during the initial memory storage originates mainly from different neuronal subpopulations. After this initial transient, a stable state is reached, and memory maintenance is achieved through attractor dynamics.

**Acknowledgments:** Funded by the Spanish Ministry of Science (RYC-2015-17236, BFU2017-86026-R, BFU2015-65315-R), by AGAUR (2017 SGR 1565) and La Caixa Foundation.

**References**

1. Constantinidis C, Franowicz MN, Goldman-Rakic PS. Coding specificity in cortical microcircuits: a multiple-electrode analysis of primate prefrontal cortex. *Journal of Neuroscience* 2001 May 15;21(10):3646–55.
2. Markowitz DA, Curtis CE, Pesaran B. Multiple component networks support working memory in prefrontal cortex. *Proceedings of the National Academy of Sciences* 2015 Sep 1;112(35):11084–9.
3. Spaak E, Watanabe K, Funahashi S, Stokes MG. Stable and dynamic coding for working memory in primate prefrontal cortex. *Journal of Neuroscience* 2017 Jul 5;37(27):6503–16.

## P334 Interactions between feedback signals for the modulation of border ownership selective neurons

### Nobuhiko Wagatsuma^1^, Ernst Niebur^2^, Brian Hu^3^, Rüdiger von der Heydt^4^

#### ^1^Toho University, Funabashi, Japan; ^2^Johns Hopkins, Neuroscience, Baltimore, MD, United States of America; ^3^Allen Institute for Brain Science, United States of America; ^4^Johns Hopkins University, Krieger Mind/Brain Institute, Baltimore, United States of America

##### **Correspondence:** Nobuhiko Wagatsuma (nwagatsuma@is.sci.toho-u.ac.jp)

*BMC Neuroscience* 2019, **20(Suppl 1)**: P334

The activity of an individual border ownership selective (BOS) neuron indicates where a foreground object is located relative to its (classical) receptive field [4]. Collectively, BOS neurons thus provide an important component of perceptual grouping, the organization of the visual scene into objects. Previous theoretical work has proposed that this grouping mechanism is implemented by dedicated neuronal population of grouping (“G”) cells and that, furthermore, these G-cells also serve as “handles” for attention to objects [1]. Recent experimental studies have investigated correlations between BOS neurons [2]. A previous theoretical study showed that modulatory common feedback may underlie the physiologically observed synchrony between BOS neurons with consistent border ownership preferences, i.e. when both neurons in the pair respond to the same visual object [3]. Here, we extended this model to explain synchrony observed between neurons with non-consistent BOS. In our model, the responses of BOS neurons are modulated by the activity of G-cells which receive their input from BOS neurons and mediate selective attention. We assume two distinct types of G-cells: spatial-attention (Gsp) and object-based G-cells (Gobj) (Fig. 1).Whereas Gsp-cells provide a fast sketch of object locations, Gobj-cells mediate object-based attention. The G-cells provide modulatory feedback to BOS neurons via N-methyl-D-aspartate (NMDA) receptors. Common feedback from G-cells modulates activity of BOS neuron and underlies the synchrony. Simulations of the network model are in overall agreement with the physiological findings reported by [2]. Our results suggest that the interactions between feedback signals from top-down and recurrent pathways play a critical role to modulate the responses of BOS neurons.

**Fig. 1:**
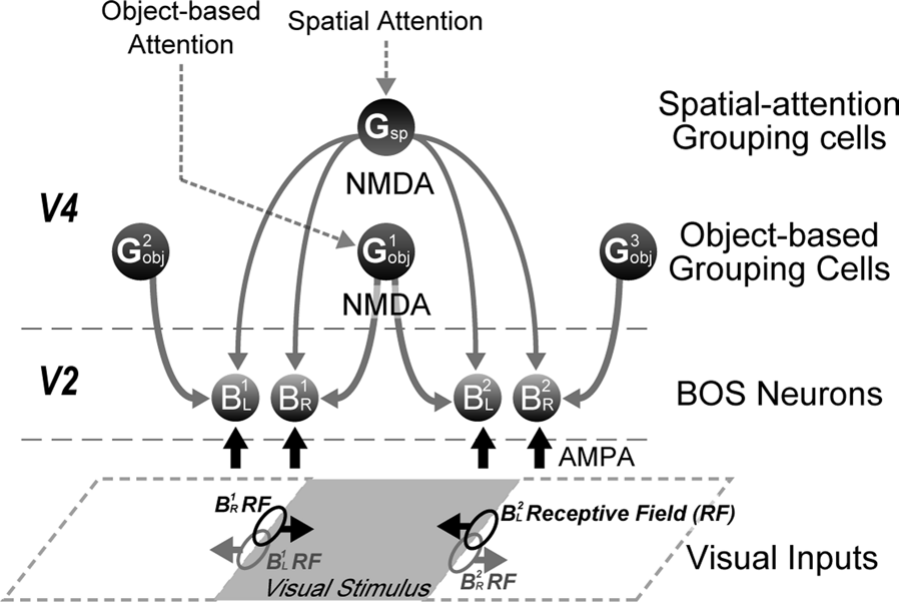
Architecture used in this study. Two distinct types of G-cells (balls with “G”) were assumed. Feedback signals from these G-cells modulate activity of BOS neuron (balls with “B”) by NMDA-type connections (gray downward pointing arrows). Black and gray ellipses represent the location of (classical) receptive fields of BOS neurons and arrows point toward the preferred side of a BOS neuron

**References**

1. Craft E, Schütze H, Niebur E, von der Heydt. A neural model of figure-ground organization. *J Neurophysiol* 2007, 97: 4310–-4326.
2. Martin AB, von der Heydt R: Spike synchrony reveals emergence of proto-objects in visual cortex. *J Neurosci* 2015, 35: 6860–6870.
3. Wagatsuma N, von der Heydt R, Niebur E: Spike synchrony generated by modulatory common input through NMDA-type synapses. *J Neurophysiol* 2016, 116: 1418–1433.
4. Zhou H, Friedman HS, von der Heydt R: Coding of border ownership in monkey visual cortex. *J Neurosci* 2000, 20: 6594–6611.

## P335 Neural variability quenching during decision-making: Neural individuality and its prestimulus complexity

### Annemarie Wolff

#### University of Ottawa, Institute of Mental Health Research, Ottawa, Canada

##### **Correspondence:** Annemarie Wolff (awolf037@uottawa.ca)

*BMC Neuroscience* 2019, **20(Suppl 1)**: P335

The spontaneous activity of the brain interacts with stimulus-induced activity which is manifested in event-related amplitude and its trial-to-trial variability (TTV). TTV describes the variability in the amplitude of the stimulus-evoked response across trials, and it is generally observed to be reduced, or quenched. While such TTV quenching has been observed on both the cellular and regional levels, its exact behavioral relevance and neuronal basis remains unclear. Applying a novel paradigm for testing neural markers of individuality in internally-guided decision-making, we here investigated whether TTV (i) represents an individually specific response by comparing individualized vs shared stimuli; and (ii) is mediated by the complexity of prestimulus activity as measured by the Lempel-Ziv Complexity index (LZC). We observed that TTV- and other electrophysiological markers such as ERP, ERSP, and ITC—showed first significant differences between individualized and shared stimuli (while controlling for task-related effects) specifically in the alpha and beta frequency bands, and secondly that TTV in the beta band correlated significantly with reaction time and eLORETA activity. Moreover, we demonstrate that the complexity (LZC) of neuronal activity is higher in the prestimulus period while it decreases during the poststimulus period, with the former also correlating specifically with poststimulus individualized TTV in alpha (but not with shared TTV). Together, our results show that the TTV represents a marker of ‘neural individualization’ which, being related to internal processes on both neural and psychological levels, is mediated by the information complexity of prestimulus activity. More generally, our results inform the pre- and post-stimulus dynamics of rest-stimulus interaction, which is a basic and ubiquitous neural phenomenon in the brain and highly relevant for mental features including their individuality. Fig. 1 shows Lempel-Ziv complexity (LZC) in the prestimulus and poststimulus periods. To examine complexity as a mechanism for TTV, as shown in Fig. 1A, 500ms prior to and after stimulus onset were investigated for complexity using the LZC measure. In paired samples *t*-tests, it was found that stimulus onset had a significant effect on complexity in both groups of stimuli and conditions. In contrast, there was no significant difference in the pseudotrials, which acted as surrogates. The time-resolved LZC seen here in Fig. 1A was computed for visualization only using a window of 500ms, overlap of 90%, and step of 50ms. Line curves were smoothed in MATLAB using the function *spline*. Two paired-samples *t*-tests was conducted comparing the difference in LZC related to stimulus onset between the individualized and shared stimuli in both conditions, as shown in Fig. 1B. There was a significant effect of stimulus in the IDM condition, but not in the EDM condition. P-values are Benjamini-Hochberg FDR corrected for multiple comparisons.

**Fig. 1:**
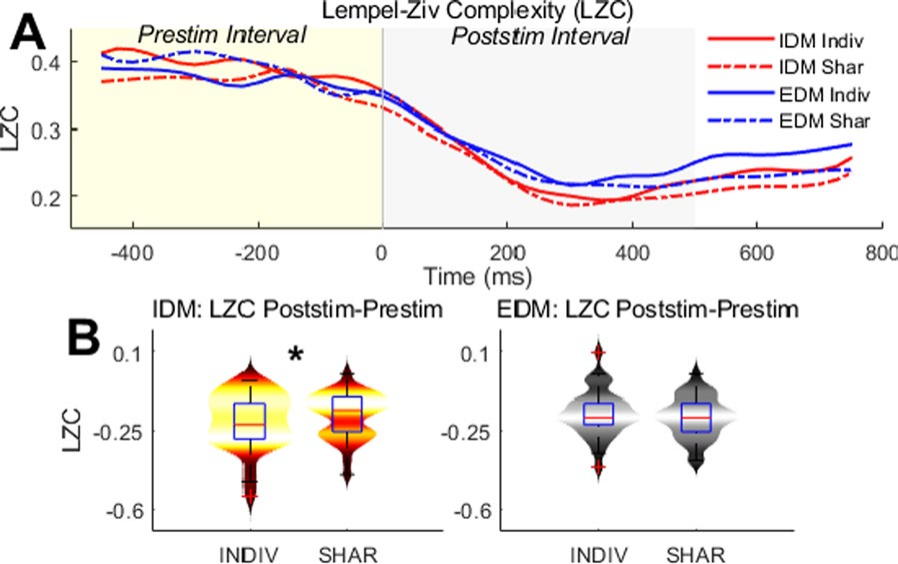
Lempel-Ziv complexity (LZC) in the prestimulus and poststimulus periods

## P336 The retina predicts information in inertial stochastic dynamics

### Min Yan^1^, Yiko Chen^2^, ChiKeung Chan^2^, K. Y. Michael Wong^3^

#### ^1^Hong Kong University of Science and Technology, Hong Kong, Hong Kong; ^2^Institute of Physics, Academia Sinica, Taipei, Taiwan, Physics, Taiwan, Taiwan; ^3^Hong Kong University of Science and Technology, Physics, Hong Kong, Hong Kong

##### **Correspondence:** Min Yan (myanaa@connect.ust.hk)

*BMC Neuroscience* 2019, **20(Suppl 1)**: P336

Vision is one of the most vital sensory modalities for animals to receive information from the surrounding world. Visual stimuli are first received by the retina, but the processing of visual signals begins first at the retina, instead of the visual cortex [1]. Experiments showed that the retina not only receives, but also preprocesses, the visual information. In experiments on the salamander retina, the mutual information between the visual signals and the responses of the retina with various time differences showed that responses of the retina actually have correlations with subsequent visual inputs [2]. Hence, not only can the retina transmit information, but it can also anticipate future signals based on what it has received. The anticipative mechanism in the retina is useful, especially when animals need to make quick responses and decisions to survive. The earlier the processing of the information, the higher the efficiency of signal analysis. Recently, experiments on the bullfrog retina showed that visual stimuli applied in the forms of Hidden Markov Model (HMM) and Ornstein-Uhlenbeck (OU) process resulted in different behaviors [3]. Predictive ability is present for HMM but effectively disappears in OU processes. To model these predictive behaviors, we propose a neural network model to simulate the dynamics of the amacrine cells and ganglion cells [4]. Since the stochastic dynamics is driven by inertia (or momentum) in HMM but not in OU, our model incorporates elements that accommodate inertia, an example being the inhibitory feedback in [5]. We found that when HMM stimuli are applied, single ganglion cells can realize anticipating tasks well, in accordance with experimental results. Besides, the population of ganglion cells can also achieve the predictive task as a whole (Fig. 1). On the other hand, for inputs produced by the OU process, the mutual information computed from single neurons or the whole network is effectively non-predictive, also in accordance with experimental results.

**Fig. 1:**
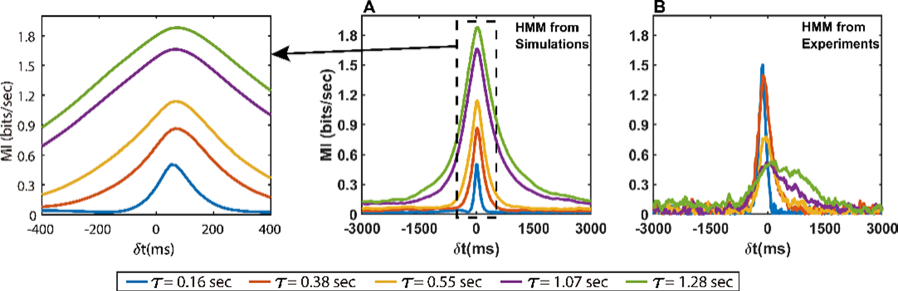
The mutual information (MI) curves for various correlation times τ of HMM. Positive δt denotes prediction. **a** The mutual information calculated from simulations. **b** The mutual information measured from experiments. Amplified part of ‘predictive MI’ in **a** is in the left figure

**Acknowledgements**: This work is supported by the Research Grants Council of Hong Kong (grant numbers 16322616 and 16306817) and the MOST 105-2112-M-001 -017 -MY3K.

**References**

1. Thomas Jessell, Siegelbaum S, Hudspeth AJ. *Principles of neural scienc*e 2000, Jan (Vol 4, pp 1227–1246) New York: McGraw-Hill;
2. Palmer SE, Marre O, Berry MJ, Bialek W. Predictive information in a sensory population. *Proceedings of the National Academy of Sciences* 2015 Jun 2;112(22):6908–13.
3. Chen KS, Chen CC, Chan CK. Characterization of predictive behavior of a retina by mutual information. *Frontiers in computational neuroscience* 2017 Jul 20;11:66.
4. Zhang AJ, Wu SM. Responses and receptive fields of amacrine cells and ganglion cells in the salamander retina. *Vision research* 2010 Mar 17;50(6):614–22.
5. Berry II MJ, Brivanlou IH, Jordan TA, Meister M. Anticipation of moving stimuli by the retina. *Nature* 1999 Mar;398(6725):334.

## P337 Robustness to the removal of connections in spiking neural networks evolved for temporal pattern recognition

### Muhammad Yaqoob^1^, Neil Davey^2^, Volker Steuber^2^, Borys Wrobel^1^

#### ^1^Adam Mickiewicz University Poznan, Evolving Systems Laboratory, Poznan, Poland; ^2^University of Hertfordshire, Biocomputation Research Group, Hatfield, United Kingdom

##### **Correspondence:** Volker Steuber (v.steuber@herts.ac.uk)

*BMC Neuroscience* 2019, **20(Suppl 1)**: P337

In order to understand how memory and robustness (to altered parameters and missing connections) emerges in spiking neural networks, we have evolved very small spiking neural networks for temporal pattern recognition in presence of membrane potential noise. The network consists of three input channels, a maximum of three interneurons and one output neuron. Interneurons and output neurons are represented by adaptive exponential integrate and fire models which generate a variety of spiking behaviors with different sets of parameters. For the current study we use the set of parameters which generates tonic spiking in response to constant input current [1]. If we consider the input signals as letters A, B and C, the task of the network would be to recognise input signals in a particular order, for example A followed by B followed by C in a continuous sequence of signals such as ACCBCCBABCACBBABCAC. Each signal lasts for 6 ms followed by a silent interval of 16 ms. The evolved network should not respond to any other possible combination of A, B and C. We use a genetic algorithm to evolve the topology and weights of the connections of the network. The initial population consists of 300 randomly created individuals encoded as a linear genome [2]. The fitness function rewards for a spike response after occurrence of a correct pattern (for example ABC) and penalises spikes elsewhere. The evolved network is robust to changes in neuronal parameters [3]. We are currently investigating which connections can be removed from the network without compromising performance.

**References**

1. Brette R, Gerstner W. Adaptive exponential integrate-and-fire model as an effective description of neuronal activity. *Journal of Neurophysiology* 2005, 94, 3637–3642.
2. Wrobel B, Abdelmotale A, Joachimczak M. Evolving networks processing signals with a mixed paradigm, inspired by gene regulatory networks and spiking neurons. *In: Di Caro, G.A., Theraulaz, G. (eds.) BIONETICS 2012. LNICST* 2014, vol. 134, pp. 135–149. Springer, Cham.
3. Yaqoob M, Wrobel B. Robust very small spiking neural networks evolved with noise to recognize temporal patterns. *In: ALIFE 2018: Proceedings of the 2018 Conference on Artificial Life* 2018, pp. 665–672. MIT Press

## P338 Neuronal hyperactivity by age-dependent alternations of brain structural connectivity in APOE4 carriers: Preliminary computational study

### Yasunori Yamada

#### IBM Research, Tokyo, Japan

##### **Correspondence:** Yasunori Yamada (ysnr@jp.ibm.com)

*BMC Neuroscience* 2019, **20(Suppl 1)**: P338

The epsilon 4 allele of apolipoprotein E (APOE) is the major genetic risk factor for Alzheimer’s disease (AD), but the reason APOE4 carriers have a higher incidence of AD than non-carriers is still not fully understood. Most studies have investigated APOE-mediated AD pathology by focusing on the ability of APOE4 to increase the aggregation and decrease the clearance of amyloid β (Aβ) [1]. In contrast, recent studies have suggested APOE4 also contributes to AD pathogenesis by Aβ-independent mechanisms [1-3]. One study investigated APOE mice lacking overt Aβ pathology and identified APOE4-associated neuronal hyperactivity driven by decreased background inhibition [3]. Because neuronal hyperactivity has been shown to be an early phenotype and accelerate AD pathology, this APOE4-associated hyperactivity has been suggested as a causative factor driving increased risk of AD [3]. However, the mechanism that links APOE4 to the decreased background inhibition driving neuronal hyperactivity remains unclear. On the other hand, other neuroimaging studies also investigated APOE4 carriers who were Aβ negative and suggested the age-dependent and Aβ-independent effects on structural connectivity [2]. However, whether and how the alternations of brain structural connectivity contribute to the increased risk of AD is not clear. In this study, I investigate the association between age-dependent alternations of structural connectivity in APOE4 carriers and neuronal hyperactivity by using computer simulations. Specifically, I built large-scale brain models consisting of 2.5 million excitatory and inhibitory spiking neurons and 5.0 billion synaptic connections. For determining inter-areal connectivity of each model, I used structural connectivity data of APOE4 carriers of different ages in which age-dependent alternations characterized by local interconnectivity loss had been reported [4, 5]. I then simulated and compared resting-state brain activities. Consequently, I found that while intrinsic cortical activities of each model matched typical patterns and quantitative indices from biological observations [6], the models based on the data of older APOE-4 carriers significantly increased excitatory neuronal activity along with reduced inhibitory activity and increased complexity of neural ensembles. This preliminary result suggests that age-dependent alternations of brain structural connectivity in APOE4 carriers might contribute to increased risk of AD by driving neuronal hyperactivity.

**References**

1. Liu CC, Kanekiyo T, Xu H, Bu G. Apolipoprotein E and Alzheimer disease: risk, mechanisms and therapy. *Nature Reviews Neurology* 2013 Feb;9(2):106.
2. Pievani M, Filippini N, Van Den Heuvel MP, Cappa SF, Frisoni GB. Brain connectivity in neurodegenerative diseases—from phenotype to proteinopathy. *Nature Reviews Neurology* 2014 Nov;10(11):620.
3. Nuriel T, Angulo SL, Khan U, et al. Neuronal hyperactivity due to loss of inhibitory tone in APOE4 mice lacking Alzheimer’s disease-like pathology. *Nature communications* 2017 Nov 13;8(1):1464.
4. Brown JA, Terashima KH, Burggren AC, et al. Brain network local interconnectivity loss in aging APOE-4 allele carriers. *Proceedings of the National Academy of Sciences* 2011 Dec 20;108(51):20760-5.
5. Brown JA, Rudie JD, Bandrowski A, Van Horn JD, Bookheimer SY. The UCLA multimodal connectivity database: a web-based platform for brain connectivity matrix sharing and analysis. *Frontiers in neuroinformatics* 2012 Nov 28;6:28.
6. Ikegaya Y, Sasaki T, Ishikawa D, et al. Interpyramid spike transmission stabilizes the sparseness of recurrent network activity. *Cerebral Cortex* 2012 Feb 7;23(2):293-304.

## P339 Integration of a reinforcement learning module in REACH model for adaptive arm control

### Taro Sunagawa, Tadashi Yamazaki

#### The University of Electro-Communications, Graduate School of Informatics and Engineering, Tokyo, Japan

##### **Correspondence:** Taro Sunagawa (sunagawa.taro@uec.ac.jp)

*BMC Neuroscience* 2019, **20(Suppl 1)**: P339

The recurrent error-driven adaptive control hierarchy (REACH) model [1] is a spiking network model for brain-style motor learning and control. The model can control a nonlinear multi-link arm adaptively against perturbation such as an unknown force field. The model, however, must be preprogrammed to move the arm appropriately under perturbation-free condition. Thus, the model does not have ability to learn from scratch. Reinforcement learning (RL) is a learning mechanism such that an agent learns appropriate behavior from scratch. RL is thought to be mediated by the basal ganglia (BG) in the brain. Therefore, we integrated BG model into REACH model to learn to move arm from scratch.

REACH model contains premotor cortex (PMC), primary motor cortex (M1) and cerebellum (CB). PMC generates abstract trajectories from start position to goal position, M1 transforms forces from abstracted representation to joint torques, and CB generates error correcting signals. We have implemented an RL algorithm called actor-critic model and integrated it into REACH model as a BG model. An actor-critic model consists of two networks, an actor network and a critic network. The critic network learns a value function that represents how good a given state is, whereas the actor network learns a policy that represents which action should be taken in a given state [2]. The learning is driven by the temporal-difference (TD) error, which is computed by the value function and external rewards. In BG, dopamine neurons are thought to provide the reward signals and thus trigger long-term synaptic plasticity. In the arm reaching task the BG model provides the predicted goal position and joint torques to move the arm to the goal, based on the current estimation of the goal position, current joint angles and angular velocities, the current hand position, and reward which is the distance between the predicted and actual goal positions.

Using this integrated model, we simulated arm reaching task in which the model moves the arm to the given target. Fig. 1 shows the sequence of predicted goal positions estimated by the BG model during 3,500 trials of arm movement. The accuracy of prediction is improved across trials. This result suggest that the BG model learn learns to predict the goal position gradually, which in turn allow the entire model to move the arm to the and the entire model learns moving arm to the goal position from scratch.

**Fig. 1:**
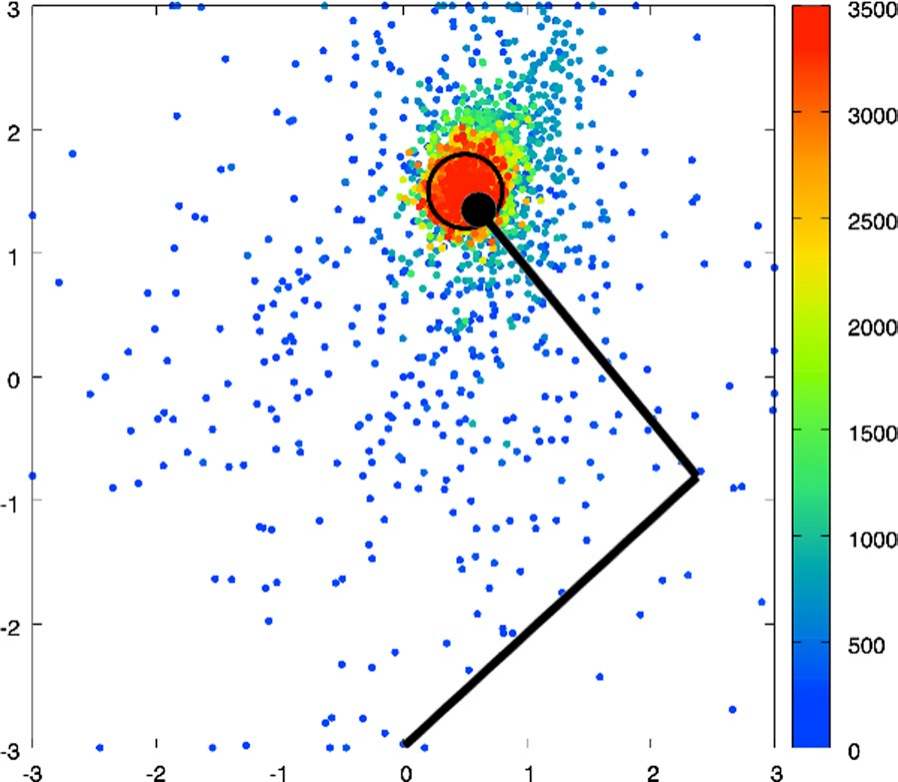
Predicted goal positions during 3500 trials of reaching. Each point represents the predicted target position for each trial. Color shows the trial number. The black circle represents the actual goal. The arm has two links and two joints (black thick lines with a small filled circle). It is controlled by joint torques exerted by the M1 module

We confirmed that the model successfully learns to predict the goal position and move the arm to the predicted position. We expect implementing more biologically plausible models would improve the performance of motor control, and analyzing neuron activities would provide better understanding of brain functions during motor control.

**Acknowledgments:** We would like to thank Dr. Hiroshi Yamakawa and the members at Dwango AI laboratory for their comments on this study.

**References**

1. DeWolf T, Stewart TC, Slotine JJ, Eliasmith C. A spiking neural model of adaptive arm control. *Proceedings of the Royal Society B: Biological Sciences* 2016, 283, 1843
2. Sutton RS, Barto AG. Reinforcement learning: An introduction (Vol. 1). *MIT press Cambridge*, 1998.

## P340 Building a spiking network model of the cerebellum on K computer using NEST and MONET simulators

### Hiroshi Yamaura^1^, Jun Igarashi^2^, Tadashi Yamazaki^3^

#### ^1^The University of Electro-Communications, Tokyo, Japan; ^2^Riken, Computational Engineering Applications Unit, Saitama, Japan; ^3^The University of Electro-Communications, Graduate School of Informatics and Engineering, Tokyo, Japan

##### **Correspondence:** Hiroshi Yamaura (yamaurahiroshi@uec.ac.jp)

*BMC Neuroscience* 2019, **20(Suppl 1)**: P340

The cerebellum plays important roles in motor control, learning, and cognitive functions. However, the computational mechanism is not fully understood. The human cerebellum holds 80% of all neurons in the human brain [1]. We believe that dynamics of the neural network composed of huge neurons contributes to the functions. Furthermore, the cerebellum is interconnected with the cerebral cortex and the basal ganglia. To understand the computational mechanism of the cerebellum, it is necessary to consider the interaction with the other parts of the whole brain. Simulation of a realistic neural network model is a useful tool to examine how neural network dynamics contributes to the brain function. Under the support of Post-K Exploratory Challenge #4, we built a spiking network model of the cerebellum based on our previous study [2] on Japanese flagship K computer. First, we used NEST simulator running on K computer [3]. We carried out computer simulation of optokinetic response (OKR), which is one of the simplest form of cerebellum-dependent eye movement task, and confirmed that the NEST version exhibited qualitatively the same results as the previous version. In response to sinusoidally modulated mossy fiber input signals, granule cells exhibited complex reservoir-like activity (Fig. 1A). Further, simulated neuronal activity patters of Purkinje cells (Fig. 1B) and vestibular nuclear neurons (Fig. 1C) are consistent with electrophysiological findings. Moreover, with the other groups in the same Post-K project, we have been connecting the present model with the other models for the cerebral cortex and the basal ganglia solely on NEST simulator. On the other hand, we implemented the same model on a different simulator called MONET (Mille-feuille like Organization NEural neTwork), which has been developed by the other group of this project. The MONET simulator partitions a stacked two-dimensional sheets of a network structure into a number of smaller square tiles and then performs calculation in parallel for the tiles. The MONET version produced qualitatively similar results of OKR simulation with the NEST version as well as the original version. We examined whether the network size can be further extended with the MONET version. We succeeded to build a very large-scale network model composed of more than 68 billion spiking neurons. The size is almost the same with the human entire cerebellum. In other words, we succeeded to build a human-scale cerebellar model on K computer. We expect that our cerebellar network model on NEST and MONET simulators would allow us to explore how such large-scale model and interaction with the cerebral cortex model play roles in complex voluntary motor tasks as well as higher-order cognitive tasks in which the cerebellum is involved.

**Fig. 1:**
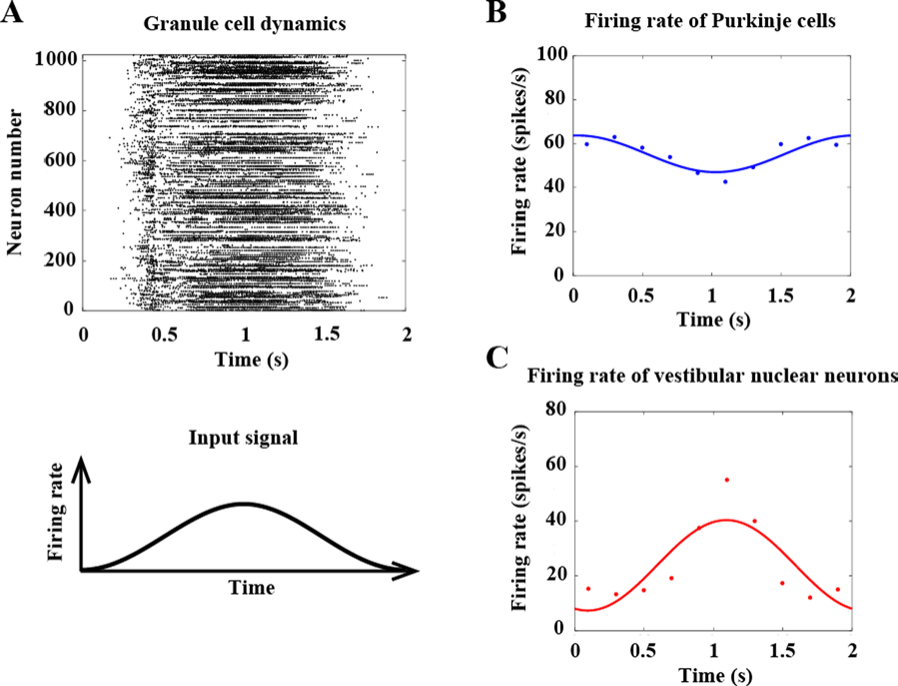
Network dynamics of the cerebellar network model on NEST simulator. **a** Top: Activity pattern of 1,024 granule cells in response to sinusoidally modulating input signal, which represents sinusoidally rotated visual world movements. Dot represents a spike. Bottom: Firing rate of the input signal. Firing rate of **b** Purkinje cells and **c** vestibular nuclear neurons

**Acknowledgements**: This work was supported by MEXT Post-K Exploratory Challenge #4.

**References**

1. Herculano-Houzel S. The human brain in numbers: a linearly scaled-up primate brain. Front. Hum. *Neuroscience* 2009, 3, 31.
2. Yamazaki T, Igarashi J, Makino J, et al. Real-time simulation of a cat-scale artificial cerebellum on PEZY-SC processors. *The International Journal of High Performance Computing Applications* 2019, 33, 155–168.
3. Kunkel S, Schmidt M, Eppler JM, et al. Spiking network simulation code for petascale computers. *Frontiers in Neuroinformation* 2014, 8, 78.

## P341 Neuromusculoskeletal model for bipedal locomotion under a pathological condition

### Daisuke Ichimura, Tadashi Yamazaki

#### The University of Electro-Communications, Graduate School of Informatics and Engineering, Tokyo, Japan

##### **Correspondence:** Daisuke Ichimura (dai.dai.dai.1014@hotmail.co.jp)

*BMC Neuroscience* 2019, **20(Suppl 1)**: P341

The motor module hypothesis [1] proposes that the nervous system groups a number of muscles into smaller number of modules to simplify motor control such as locomotion [2]. In locomotion of healthy adults, four or five motor modules are recruited, whereas patients with neural disorders such as stroke exhibit abnormal locomotion patterns, and two or more motor modules are merged into one [3]. However, how the nervous system of patients with alters the organization of motor modules is unclear. In this study, we focused on how motor modules are altered in pathological locomotion by building a two-dimensional neuromusculoskeletal model and carrying out its dynamical simulation.

The two-dimensional neuromusculoskeletal model consists of seven rigid links and nine principal muscles for each leg, a neural system with hierarchical central pattern generator (CPG), and various feed backs from sensor organs (Fig. 1A). The CPG contains a rhythm generator (RG) and a pattern formation (PF) network. The RG is composed of four neurons mutually inhibited to generate rhythm, whereas the PF network contains 10 neurons with mutual- and self-inhibition that correspond to five motor modules for each leg. 62 parameters of the neural system were tuned by genetic algorithms (GAs). After the parameter estimation, the model successfully walked for five sec. We call this a normal locomotion model. Then, we weakened the amplitude of neural inputs to muscles on one side leg to simulate a pathological condition such as stroke. We call this a pathological locomotion model. In the pathological model, we ran GAs for searching parameters only for proprioceptive feedback.

**Fig. 1:**
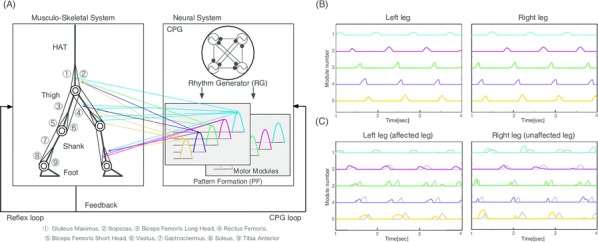
**a** Schematic figure of the proposed neuromusculoskeletal model. The proposed neural system with a CPG model composed of a rhythm generator (RG) network and a pattern formation (PF) network. **b** Patterns of the motor modules in the normal locomotion model. **c** Patterns of the motor modules in the pathological locomotion model. Gray lines show the motor modules in the normal locomotion model

After, 3000 generations of GAs, the normal locomotion model acquired a stable bipedal locomotion. The locomotion pattern resembles that of the human biped quantitatively. Next, we changed the model to the pathological condition. Initially, the model failed to walk. Then, after 1000 generations of GAs, the model once again succeeded to walk. The locomotion pattern showed more variations in the step size. Figure 1B shows the activity of 10 motor modules, five for left and five for right legs, in the normal locomotion model for three steps, showing that these modules are activated one by one sequentially with different phases. Figure 1C illustrates the same plots in the pathological locomotion model. In the affected leg, the first module does not exhibit marked activity due to the weakening of neural inputs, and the second and third modules tended to become active closely in time, suggesting that these modules are merged. These results suggest that alterations of proprioceptive feedback affect motor modules for stroke patients.

**Acknowledgments:** We thank Dr. Kazunori Hase (Tokyo Metropolitan University) for helpful discussions on building the neuromusculoskeletal model.

**References**

1. Ting LH, et al. Neuromechanical principles underlying movement modularity and their implications for rehabilitation. *Neuron* 2015, 86, 38–54.
2. Ivanenko YP, et al. Five basic muscle activation patterns account for muscle activity during human locomotion. *Journal of Physiology* 2004, 556, 267–282.
3. Clark DJ, et al. Merging of healthy motor modules predicts reduced locomotor performance and muscle coordination complexity post-stroke. *Journal of Neurophysiology* 2010, 103, 844–857.

## P342 Developing a spiking neuron network model of the basal ganglia performing reinforcement learning

### Hideyuki Yoshimura, Tadashi Yamazaki

#### The University of Electro-Communications, Graduate School of Informatics and Engineering, Tokyo, Japan

##### **Correspondence:** Hideyuki Yoshimura (y1831169@edu.cc.uec.ac.jp)

*BMC Neuroscience* 2019, **20(Suppl 1)**: P342

In the brain, the basal ganglia (BG) is thought to be the site for Reinforcement Learning (RL) [1] using dopaminergic system. RL plays important roles in several brain functions such as decision making and motor control. A precise model of BG performing RL would contribute to realize brain-style artificial intelligence with advanced functions like human beings. On the other hand, spiking neuron networks are paid much attention by its computational power and low power consumption of neuromorphic hardware. Therefore, we developed a spiking neuron network model of the basal ganglia.

The network structure of our model consisted of 9 layers, including the pre-frontal cortex (PFC), patch of striatum, matrix of striatum-D1/D2, globus pallidus pars interna/externa, subthalamic nucleus, thalamus, and premotor cortex (PMC). We did not implement the substantia nigra pars compacta (SNc) as spiking neuron layer. Each layers had 100 spiking neurons, except PFC and PMC. For PFC and PMC, we set the number of neurons in accordance with a task. A neuron model we used was a simplified spike response model (SRM 0) [2], which emit spikes stochastically according to its membrane potential. For synaptic plastisity rule realizing Reinforcement Learning, we applied TD-LTP [3] method to synapses, from PMC to striatum. In our model, cortico-striatal synaptic weights were modulated by the difference of pre-post spike time and reward prediction error (RPE) signal like dopaminergic neuron activities of SNc. The RPE signal were calculated from activity of neurons in the patch of the striatum.

We implemented the model on NVIDIA GeForce GTX TITAN Z in C++ and CUDA to accelerate numerical calculation. Then, to verify the performance of the model, we conducted numerical simulation of two standard RL tasks: arm reaching and water maze. In reaching task, an agent moves its arm, and obtains reward if the arm reaches a target. The agent learns to move the arm towards the target. In the case of water maze task, an agent is put on the starting point and explores the maze. When the agent reaches the goal area, the agent obtains a reward. On the other hand, the agent is given a punishment on touching obstacles or walls in the maze. The agent learns to go to the goal while avoiding obstacles and walls.

As a result, our BG model successfully learned to move the arm smoothly to the target, and to find a goal in the maze, respectively. Furthermore, all the numerical simulation ran faster than real-time.

These results suggest that our BG model implemented on GPU allows us to study the detailed dynamics and learning process of the BG in a real-world environment. Moreover, we believe that we could implement the same model on neuromorphic processors, and the online RL capability would be useful to solve various real-world and AI problems.

**Fig. 1:**
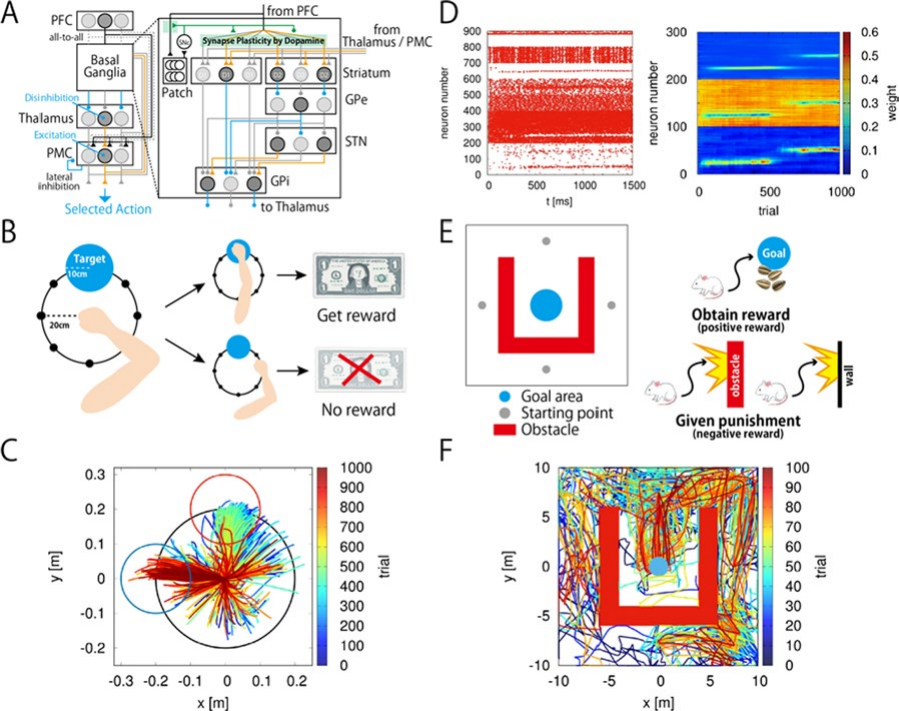
**a** Network structure. **b** Reaching task overview. **c** Arm trajectory of the reaching task. The target was shown at 12 o’clock in the first 500 trials, and the target was moved to 9 o’clock in the next 500 trials. **d** Raster plot of the last trial in **c** (left). Cortico-striatal synaptic weights changing in 1000 trials (right). **e** Maze task overview. **f** Trajectories of the agent for each trial of maze t

**Acknowledgements**: This presentation is based on results obtained from a project commissioned by the New Energy and Industrial Technology Development Organization (NEDO).

**References**

1. Sutton RS, Barto AG. Reinforcement learning: An introduction. *MIT press;* 1998.
2. Gerstner W, Kistler WM. Spiking neuron models: Single neurons, populations, plasticity. *Cambridge university press;* 2002 Aug 15.
3. Frémaux N, Sprekeler H, Gerstner W. Reinforcement learning using a continuous time actor-critic framework with spiking neurons. *PLoS computational biology* 2013 Apr 11;9(4):e1003024.

## P343 Parallel computing of a cortico-thalamo-cerebellar circuit using tile partitioning parallelization method by MONET simulator

### Jun Igarashi^1^, Hiroshi Yamaura^2^, Tadashi Yamazaki^3^

#### ^1^Riken, Computational Engineering Applications Unit, Saitama, Japan; ^2^The University of Electro-Communications, Tokyo, Japan; ^3^The University of Electro-Communications, Graduate School of Informatics and Engineering, Tokyo, Japan

##### **Correspondence:** Jun Igarashi (jigarashi@riken.jp)

*BMC Neuroscience* 2019, **20(Suppl 1)**: P343

The next generation supercomputers with exaflops levels of computational performance in the 2020s are estimated to be able to perform human-scale whole-brain spiking neural networks. However, it remains unclear how we can efficiently perform communication of increasing spike data among compute nodes, load balancing for the heterogeneous structure of the brain, and construction of neural networks with huge amounts of neurons and connections in whole-brain scale simulation.

We conducted a feasibility study of efficient parallelization and communication methods of a brain model using supercomputer K.

In the mammalian brain, the cortex and the cerebellum include 99 % of neurons and have layered sheet types of structures. They are densely wired within the regions and sparsely across distant regions. Therefore, an efficient parallel computing of layered sheet types of spiking neural networks with the dense-neighbor and long-range-sparse distant connections is essential for realizing human-scale whole-brain simulation from a viewpoint of calculation amount.

Taking into account the anatomical features of the brain, we chose to use tile partitioning parallelization, which assigns compute nodes with partitioned tiles of layered sheet types of neural networks. The tile partitioning method works for load balancing in both simulation and construction of networks. In addition, the communication method to reduce communication frequency of spike data that exploits signal transmission delay, which has been used in NEST and NEURON simulators, was expanded for tile partitioning method and used.

We tested the parallelization method by applying it to a realistic spiking neural network simulation of the cortico-thalamo-cerebellar circuit using our in-house simulator, MONET (Mille-feuille like Organization NEural neTwork). The cortico-thalamo-cerebellar circuit was developed based on anatomical and electrophysiological data including neural density and spatial extent of connections. We used a leaky Integrate-and-Fire neuron model for all neuron types and conductance-based synaptic models for all synapses. We used the K computer that has 88128 CPUs and 1.28 PB DRAM memory. In programming of parallel computing, we used C language, MPI and, OpenMP libraries.

We measured elapsed times of simulations for 1 second of biological time in various sizes of cortico-thalamo-cerebellar models with fixing the assigned tile size per compute node, where the numbers of neurons per compute node were 45 thousand neurons for a cortical tile, 2 thousand neurons for a thalamic tile, and 200 thousand neurons for a cerebellar tile. The total numbers of neurons and compute nodes ranged from 63 million to 1 billion neurons and from 768 to 12288 compute nodes, respectively. The elapsed times were 614 to 620 sec, which means that the sizes of the neural network scaled up with keeping the same range of elapsed time.

We also checked elapsed time of construction of the neural network. The elapsed time kept in the same range irrelevant of the network sizes.

These results demonstrated that the parallelization method realized efficient computing of cortico-thalamo-cerebellar circuit, which may contribute to human-scale whole-brain simulation on the next generation exascale supercomputers.

## P344 Dendritic discrimination of temporal input sequences in cerebellar Purkinje cells

### Yuki Yamamoto^1^, Tadashi Yamazaki^2^

#### ^1^The University of Electro-Communications, Yokohama, Japan; ^2^The University of Electro-Communications, Graduate School of Informatics and Engineering, Tokyo, Japan

##### **Correspondence:** Yuki Yamamoto (yafbf07e5961f51@gmail.com)

*BMC Neuroscience* 2019, **20(Suppl 1)**: P344

Discrimination of temporal sequences is thought as a fundamental function of the brain. Cortical pyramidal neurons exhibit sensitivity to the sequence of synaptic activation [2]. In particular, these neurons can emit spikes only when the synapses are stimulated in a certain order.

Cerebellar Purkinje cells have remarkably large dendrites. Thus, we investigated whether Purkinje cells can emit spikes in response to a certain sequential activation of synapses.

We used a Purkinje cell model built by De Schutter and Bower [1] (Fig. 1A). This is an elaborated biophysical model composed of 1,600 compartments and 10 types of voltage-dependent ion channels. The model receives excitatory parallel fiber inputs from granule cells and inhibitor inputs from stellate and Golgi cells. We stimulated multiple parallel fiber synapses sequentially in various orders, and examined the effect of sequential stimulation on the dendrites of the Purkinje cell. For efficient numerical stimulation, we reimplemented the model on GPU.

**Fig. 1:**
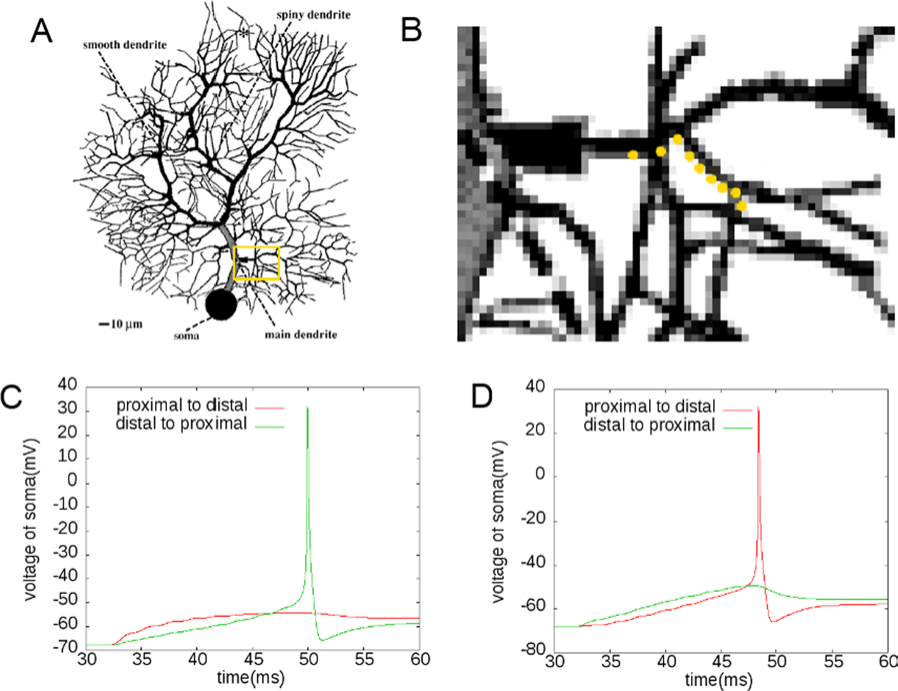
**a** Detail of the shape of Purkinje cell model built by De Schutter & Bower [1]; yellow box is Fig. B. **b** Places of synapses on dendrite we stimulated. **c** Voltage of soma when the dendrite is stimulated by parallel fiber synapses in the order from distal to proximal and from proximal to distal. **d** Voltage of soma when the dendrite is stimulated by parallel fiber synapses, adding synapse weights

We selected 9 parallel fiber synapses, all of which connected to one straight dendrite from Purkinje cell soma (Fig. 1B). We activated them in any orders, and we found that the order of stimulation from distal to proximal can produce larger somatic response than from proximal to distal (Fig. 1C). The larger directionality index of the order of stimulation was, the more likely somatic spike was emitted. We also confirmed that by assigning synaptic weights appropriately, the stimulation order for emitting spike can be reversed (Fig. 1D).

Branco et al. [2] suggest that two factors are important for the discrimination of temporal input sequences in cortical pyramidal neurons: the spatial gradient of input impedances along the dendrites and the highly nonlinear voltage dependence of the NMDAR conductances. On the other hand, the Purkinje cell model in our study suffices only the former factor, because the model does not have NMDA channels. Nevertheless, we could reproduce sequence-selective response of the cell. This suggests that either (a) spatial gradient of input impedances along dendrites was sufficient, or (b) some other mechanisms substituted the functional role of the NMDAR conductances. For (b), a potential candidate is the intracellular Ca2+ dynamics, because both NMDAR and Ca2+ exhibit voltage dependence and slow temporal dynamics. Additional simulation studies using other types of neuron models will be necessary to clarify this issue. Moreover, the reverse-order stimulation for emitting spike suggests that Purkinje cells could learn a specific sequence of parallel fiber stimulation specified by the climbing fiber inputs via long-term depression at parallel fiber-Purkinje cell synapses.

Overall, the sequential discrimination ability may enhance the computational capability of the cerebellar cortex beyond the standard notion as a perceptron. We are particularly interested in the capacity of information on the sequence such as the lengths and the variations.

**Acknowledgement**: This study was supported by JSPS Kakenhi (17K07049).

**References**

1. De Schutter ER, Bower JM. An active membrane model of the cerebellar Purkinje cell. *Journal of neurophysiology* 1994 Jan 1;71(1):375–419.
2. Branco T, Clark BA, Häusser M. Dendritic discrimination of temporal input sequences in cortical neurons. *Science* 2010 Sep 24;329(5999):1671–5.

## P345 Arbor – a morphologically-detailed neural network simulation library for contemporary high-performance computing architectures

### Nora Abi Akar^1^, Ben Cumming^1^, Felix Huber^2^, Vasileos Karakasis^3^, Anne Küsters^4^, Wouter Klijn^4^, Alexander Peyser^4^, Stuart Yates^1^

#### ^1^Swiss Supercomputing Centre, Scientific Software & Libraries, Zürich, Switzerland; ^2^University of Stuttgart, Institute of Applied Analysis and Numerical Simulation, Stuttgart, Germany; ^3^Swiss Supercomputing Centre, Scientific Computing Support, Lugano, Switzerland; ^4^Jülich Research Centre, SimLab Neuroscience, Jülich, Germany

##### **Correspondence:** Alexander Peyser (a.peyser@fz-juelich.de)

*BMC Neuroscience* 2019, **20(Suppl 1)**: P345

We introduce Arbor [1, 2], a performance portable library for the simulation of large networks of multicompartment neurons on HPC systems. Arbor is an active open source project, developed based on an open-development model with code, bug reports, and issues hosted on GitHub under the auspices of the Human Brain Project. The performance portability is by virtue of back-end specific optimizations for x86 multicore, Intel KNL, and NVIDIA GPUs. The development of Arbor has focused on tackling issues of vectorization and emerging hardware architectures by using modern C++ and automated code generation. When coupled with low memory overheads, these optimizations make Arbor an order of magnitude faster than the most widely-used comparable simulation software. The single-node performance can be scaled out to run very large models at extreme scale with efficient weak scaling (Fig. 1). Examples of new features that will be released soon include but are not limited to: a python wrapper for user-friendly model building and execution; accurate and efficient treatment of gap junctions; and a GPU solver for Hines matrices exposing more fine-grained parallelism. Arbor’s released features, its performance as well as current development work on the python front-end and the GPU solver are shown within the scope of this poster.

**Fig. 1:**
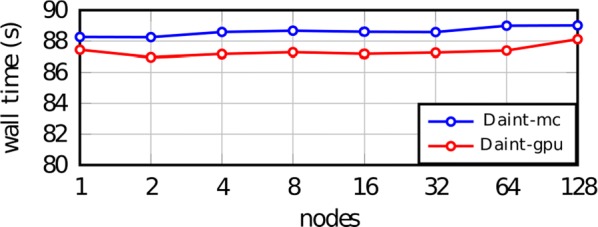
Simulation time for the weak scaling tests with 8,192 cells per node, 1 to 128 nodes. Each cell is connected to 10,000 random cells with no self-connections

**Acknowledgments:** This research has received funding from the European Union’s Horizon 2020 Framework Programme for Research and Innovation under the Specific Grant Agreement No. 720270 (Human Brain Project SGA1), and Specific Grant Agreement No. 785907 (Human Brain Project SGA2).

**References**

1. Akar NA, Cumming B, Karakasis V, et al. Arbor—A Morphologically-Detailed Neural Network Simulation Library for Contemporary High-Performance Computing Architectures. *In 2019 27th Euromicro International Conference on Parallel, Distributed and Network-Based Processing (PDP)* 2019 Feb 13 (pp. 274–282). IEEE.
2. Akar NA, Biddiscombe J, Cumming B, et al. arbor-sim/arbor: Arbor Library v0.2 (Version v0.2). *Zenodo* 2019, March 4. http://doi.org/10.5281/zenodo.2583709

## P346 Predictive coding in the drosophila antennal lobe

### Aurel A. Lazar, Chung-Heng Yeh

#### Columbia University, Department of Electrical Engineering, New York, NY, United States of America

##### **Correspondence:** Chung-Heng Yeh (chyeh@ee.columbia.edu)

*BMC Neuroscience* 2019, **20(Suppl 1)**: P346

The early olfactory system of the fruit fly encodes odorant *identity* and odorant *concentration* into a combinatorial neural code that is further processed in higher brain centers for recognition, associative learning, and other cognitive tasks. The combinatorial neural code is transformed along the olfactory system across two stages, the antenna and the antennal lobe. The antenna encodes an odorant stimulus into a concentration-dependent combinatorial code, and the antennal lobe encodes the output of antenna into a concentration-independent code. The combinatorial code at the input to the antennal lobe is concentration-dependent. It is transformed into a concentration-independent code within the antennal lobe of the fruit fly.

To interrogate the transformation of the combinatorial code, we devised a full-scale computational model of the antennal lobe at the molecular level as a predictive coding circuit. The *in silico* antennal lobe is comprised of inhibitory and excitatory local neurons (LNs), the projection neurons (PNs), synapses from olfactory sensory neurons (OSNs) to PNs, synapses from OSNs to LNs, and synapses from LNs to PNs. The predictive encoding circuit consists of LNs that presynaptically inhibit the axonal terminals of the OSNs. We advance biophysical models of the molecular processing in the axonal terminals of the OSNs and in the dendritic tree of the PNs, and biologically validate our modeling approach with data obtained from electrophysiological experiments for a subset of OSNs and PNs.

By modeling the odorant identity and concentration as an odorant-receptor affinity tensor modulated by the odorant concentration profile, we first show that the OSN spike train input to the predictive coding circuit is concentration dependent, and the affinity value characterizing different OSN types results in shifts of the OSN odorant concentration versus PSTH response curve. Second, we demonstrate that the LNs by inhibiting the axonal terminals drive the OSN axonal terminals to encode the odorant-receptor affinity value independently of the odorant concentration amplitude. We further show that the temporal response curves are contrast invariant across orders of magnitude of concentration amplitude values.

Our approach demonstrates for the first time that olfactory processing in the antennal lobe of the fruit fly is based upon predictive coding. The predictive coding circuit reproduces key properties of the olfactory processing, including divisive normalization and concentration invariant combinatorial coding. It strongly suggests that the contrast of the odorant concentration is preserved across a wide range of mean concentration amplitudes. Our work shows that predictive coding provides a new theoretical frontier for investigating the neural code in the antennal lobe and its role in memory and learning in fruit flies.

**Acknowledgments:** The research reported here was supported, in part, by NSF under grant #1544383 and in part by AFOSR under grant #FA9550-16-1-0410.

## P347 Unraveling intra- and intersegmental neural network architectures controlling an insect walking system

### Silvia Daun^1^, Azamat Yeldesbay^2^

#### ^1^Research Centre Jülich, Institute of Neuroscience and Medicine (INM-3), Jülich, Germany; ^2^University of Cologne, Institute of Zoology, Cologne, Germany

##### **Correspondence:** Azamat Yeldesbay (azayeld@gmail.com)

*BMC Neuroscience* 2019, **20(Suppl 1)**: P347

Locomotion of an animal requires a precise coordinated movement of all part of the legs. This coordination is achieved by the interaction between groups of neurons, the central pattern generators (CPGs), which drive the motoneurons and muscles. In the absence of any sensory input this network creates a stable rhythmic motor activity that is essential for a successful coordination between limbs. Hence, it is of particular interest to know the structure of this central neural circuit and the interaction between different parts of the CPG network.

This work is motivated by recent experimental results reported by [1]. By chemically activating both isolated and interconnected deafferented thoracic segments (ganglia) of the stick insect [1] analysed the interactions between contralateral networks that drive the levator-depressor muscle pairs, which are responsible for the upward-downward movement of the legs. The results of the experimental analysis showed that intrasegmental phase relationships differ between isolated segments. In particular, in isolated segments where the control networks of the middle and hind legs reside, i.e. in meso- and metathoracic ganglia, the phase relation between activities of the contralateral depressor motorneurons were in-phase and anti-phase, respectively. Moreover, the phase relations switched to in-phase and stabilized when the ganglia were interconnected.

Using the phase reduction of an intersegmental network model of stick insect locomotion presented in our previous work [2], we built a reduced model of the intra- and intersegmental network controlling levator-depressor activity in the meso- and metathoracic ganglia. By examining the intra- and intersegmental phase differences in the model we identified the properties of the couplings of the network that replicate the results observed in the experiments. We applied the theoretical analysis to escape type central pattern generators and revealed a set of possible contra- and ipsilateral synaptic connections. Finally, we defined general features of the synaptic couplings between central pattern generators of any type that maintain the phase relationships observed in the experiments.

**References**

1. Mantziaris C, Bockemühl T, Holmes P, Borgmann A, Daun S, Büschges A. Intra- and intersegmental influences among central pattern generating networks in the walking system of the stick insect. *J. Neurophysiol.* 2017, 118, 2296–2310.
2. Yeldesbay A, Tóth T, Daun S. The role of phase shifts of sensory inputs in walking revealed by means of phase reduction. *J. Comput. Neurosci.* 2018, 44, 313–339.

## P348 Effects of chaotic activity on the first-spike latency dynamics of Hodgkin-Huxley neurons

### Veli Baysal^1^, Zehra Saraç^2^, Ergin Yılmaz^1^

#### ^1^Zonguldak Bülent Ecevit Üniversitesi, Department of Biomedical Engineering, Zonguldak, Turkey; ^2^Zonguldak Bülent Ecevit Üniversitesi, Department of Electrical and Electronics Engineering, Zonguldak, Turkey

##### **Correspondence:** Veli Baysal (veli.baysal@beun.edu.tr)

*BMC Neuroscience* 2019, **20(Suppl 1)**: P348

It is a common appreciation that neurons code incoming stimulus into spike or spike train. There are two ideas on how neurons encode information in the spike train: spike time and rate. One of the encodings with spike time is latency coding in which neurons code information in their first spike times [1]. It is thought that first-spike latency conveys much of the information about the stimulus [1]. On the other hand, first spike latency could be increase due to various factors such as noise. In this context, [2] studied the effects of noise on the first-spike latency dynamics of excitable Hodgkin-Huxley (H-H) system subjected to a strong periodic forcing, and observed that appropriate noise induces a time delay on the occurrence time of the first spike of neuron which was called as “noise delayed decay” (NDD) [2]. Later, this finding has been extensively studied by using complex neuronal networks [3]. However, the origin of the neuron’s response variability is still unclear. Although it has been primarily thought that such variability in the neuron’s response is due to accumulating noise, it could be due to the chaotic activities [4]. Chaotic oscillations have been investigated in the various neuron models. The presence of chaotic activity in neurons has been shown in the numerical and experimental studies [5]. Here, we aim to present the effects of the chaotic activity on the first spike latency of single H–H neuron. To do this, we set the parameters of the Lorenz system for ensuring chaotic oscillations and obtained this chaotic signal is injected to H–H neuron along with the suprathreshold periodic signal. To measure the mean latency and jitter of the H–H neuron, the first-spike times of H–H are recorded. Then, by averaging these values mean latency time and jitter is computed. Obtained results are presented in Fig. 1.

**Fig. 1:**
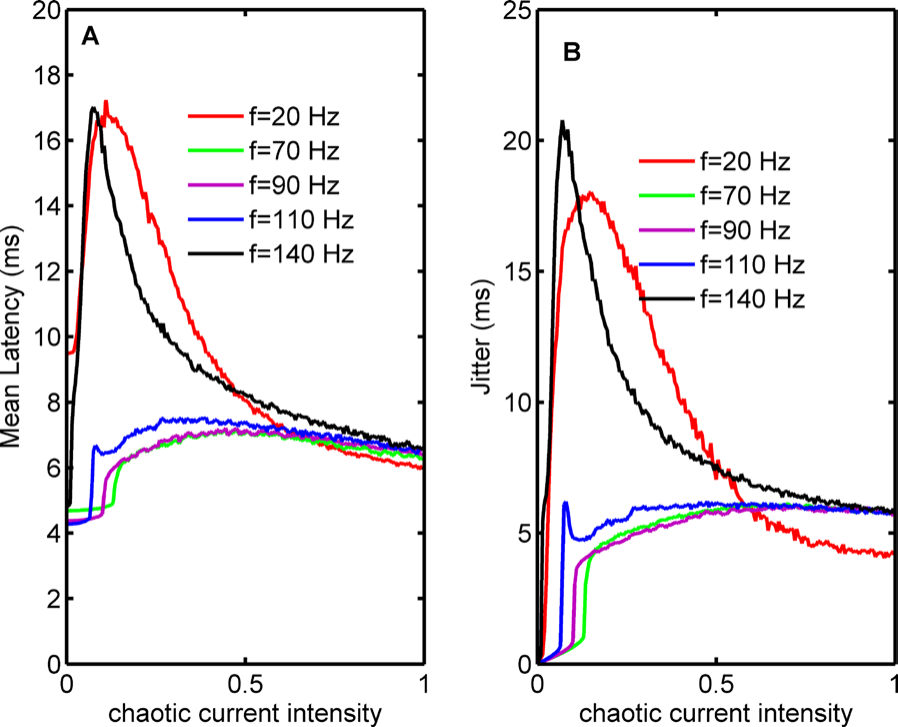
The statistics of the first-spike occurrence times depending on chaotic current intensity for various frequency of suprathreshold signal (amplitude of suprathreshold signal A = 4μA/cm^2^) **a** Mean latency of the H–H neuron. **b** Jitter of the H–H neuron

**Conclusions:** It is seen that mean latency and jitter of the first-spike times exhibit a bell-shaped dependence on the chaotic activity intensity. This finding is similar to the NDD phenomenon in ref. [2], but in our study, the effect is induced by chaotic activity instead of noise. In this context, our new finding can be called as “chaos delayed decay” (CDD).

**References**

1. Vanrullen R, Guyonneau R, Thorpe SJ. Spike times make sense. *Trends in Neuroscience* 2005, 28:1–4.
2. Pankratova EV, Polovinkin AV, Mosekilde E. Resonant activation in a stochastic Hodgkin–Huxley model: interplay between noise and suprathreshold driving effects. *European Physical Journal B* 2005, 45:391–397.
3. Yilmaz E. Impacts of hybrid synapses on the noise-delayed decay in scale-free neural networks. *Chaos, Solitons & Fractals* 2014, 66: 1–8.
4. Chacron MJ, Longtin A, Pakdaman K. Chaotic firing in the sinusoidally forced leaky integrate-and-fire model with threshold fatigue. *Physica D: Nonlinear Phenomena* 2004, 192:138–160.
5. Korn H, Faure P. Is there chaos in the brain? II. Experimental evidence and related models. *Comptes rendus biologies* 2003 Sep 1;326(9):787–840.

## P349 Stochastic resonance in feed-forward-loop neuronal network motifs of Hodgkin-Huxley neurons

### Veli Baysal, Ergin Yılmaz

#### Zonguldak Bülent Ecevit Üniversitesi, Department of Biomedical Engineering, Zonguldak, Turkey

##### **Correspondence:** Veli Baysal (veli.baysal@beun.edu.tr)

*BMC Neuroscience* 2019, **20(Suppl 1)**: P349

Information coding and transmission in brain are fulfilled via neuronal networks. It is shown that neuron communities have some of the characteristics of complex network structures [1-2]. On the other hand, it is indicated that complex networks include some micro topologies called “network motifs,” which are believed to be basic building blocks of these networks [3]. Therefore, it is important to demonstrate the dynamics and functions of this motif networks in order to understand the behavior of complex networks. With this motivation, in this study, we investigate the Stochastic Resonance (SR) phenomenon in a triple-neuron feed-forward-loop (FFL), shown in Fig. 1. Here, FFL motifs have eight possible structural configurations depending on the synapse type in the FFL motifs (Fig. 1C). We found that Q (Fourier coefficients, performance of weak signal detection of neuron 3) exhibits SR in all FFL motifs. Also, the weak signal transmission in networks via SR mechanism is dependence on synapse type. There are two ways to efficiently transmitted the weak signal to neuron 3: Synapse 3 should be excitatory, type of other synapses is unimportant, or Synapse 1 and Synapse 2 should be excitatory, type of synapse 3 is unimportant.

**Fig. 1:**
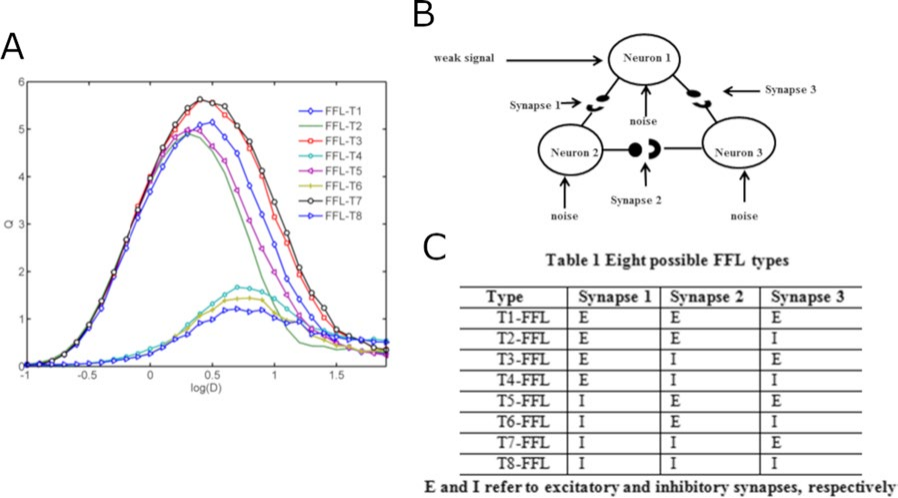
**a** The weak signal transmission dependence on noise intensity in different FFL motifs. **b** Schematic illustration of the considered feed-forward-loop motif. The weak signal is applied only neuron 1. Neuron 1 and 3 is considered as input and output neurons, respectively. **c** Table 1

**References**

1. Kaiser M. A tutorial in connectome analysis: Topological and spatial features of brain networks. *NeuroImage* 2011, 57:892–907.
2. Pastor-Satorras R, Vespignani A. Epidemic Spreading in ScaleFree Networks. *Physical Review Letters* 2001, 86:3–3200.
3. Reigl M, Alon U, Chklovskii DB. Search for computational modules in the C. elegans brain. *BMC biology* 2004 Dec;2(1):25.

## P350 Visual perception of spatial objects and textures in flying pigeons

### Margarita Zaleshina^1^, Alexander Zaleshin^2^

#### ^1^Moscow Institute of Physics and Technology, Moscow, Russia; ^2^Institute of Higher Nervous Activity and Neurophysiology, Moscow, Russia

##### **Correspondence:** Margarita Zaleshina (zaleshina@gmail.com)

*BMC Neuroscience* 2019, **20(Suppl 1)**: P350

Many studies examine different cases of perception of visual elements as significant objects, borders, textures, as well as filtration of many of the observed elements as just noise. Spatial perception can be considered at the level of behavior and at the level of brain activity.

To understand the complex spatial orientation it is necessary to study not only short-distance movements (indoors locomotion), but also medium-distance movements, such as pigeon flights. Pigeon trajectories during medium-distance flights are determined, in particular, by the visual perception of the terrain.

This work considers hypothesis that visual perception of external environment affects reactions of birds during medium-distance flights, which is reflected in birds’ trajectories.

Simultaneous comparison of data on external environment, on pigeon trajectories and on activities in the brains of birds helps to determine which elements of landscape can be a stimulus for bird navigation. Responses to basic spatial elements appear at the level of place cells, head direction cells, grid cells and a boundary cells [1]. Visual perception of more complex scenes is represented in total brain activity [2]. GPS tracks are often used to examine pigeon’s ability to consider visual landmarks and to change navigational behavior [3]. Analysis of power changes in high-frequency bands of the pigeon EEG allows to identify the response of bird’s brain to significant previously known visual navigational landmarks [4].

In this work, data on the flights of pigeons and remote sensing data for terrain over which these flights took place were used as primary source materials. Data packages were collected from Dryad Digital Repository (https://datadryad.org). Satellite images in the form of OpenLayers (http://openlayers.org) were used to obtain surface information.

This work showed that pigeon’s flight paths may reflect specific areas and objects in terrain. Here, we calculated typical time delays in pigeon responses after perception of visual stimuli during flights, and described characteristic reactions to visual stimuli for the intervals +/- 10 seconds (shown in Fig. 1). As a result, it was shown that the response characteristics vary depending on the ability of the pigeon to visually detect separate elements of the terrain during flight. So, it is possible to identify the features of birds’ response both to single landmarks and to boundaries of different surfaces.

**Fig. 1:**
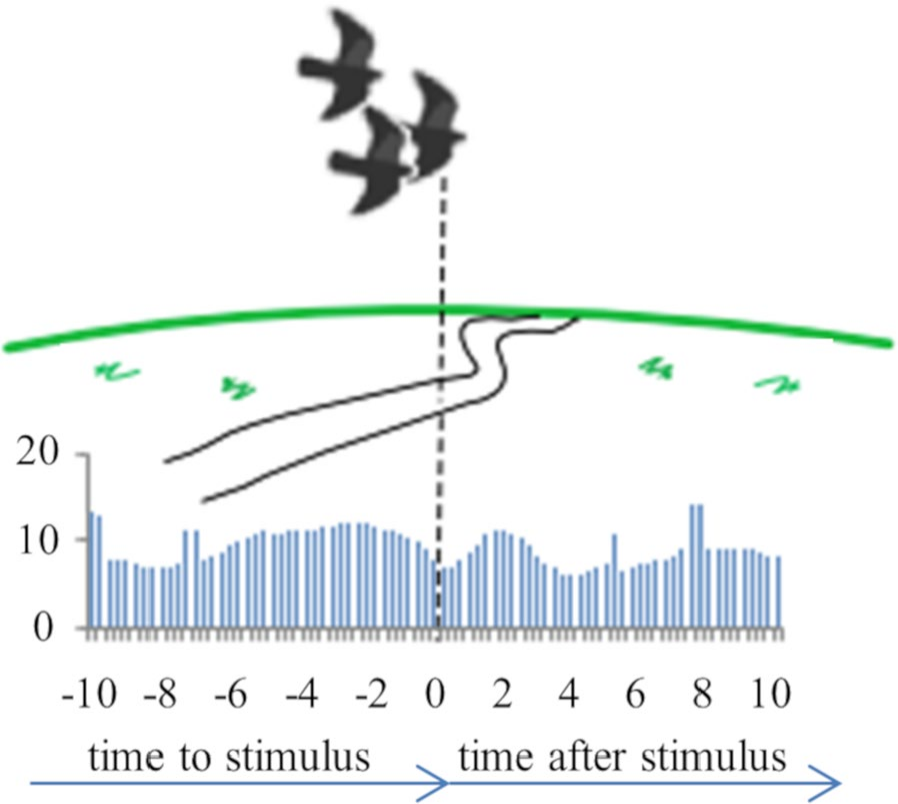
Stimulus-response correspondence in flying pigeons

Analysis of visual perception of landscapes, textures and landmarks in flying pigeons helps to better understand how spatial features are represented in the mind during motion.

**References**

1. Hartley T, Lever C, Burgess N, O’Keefe J. Space in the brain: how the hippocampal formation supports spatial cognition. *Philos Trans R Soc Lond B Biol Sci* 2013, 369:20120510.
2. Leeds DD, Pyles JA, Tarr MJ. Exploration of complex visual feature spaces for object perception. *Front Comput Neurosci* 2014, 8:106.
3. Biro D, Meade J, Guilford T. Familiar route loyalty implies visual pilotage in the homing pigeon. *Proc Natl Acad Sci USA* 2004, 101:17440–17443.
4. Vyssotski AL, Dell’Omo G, Dell’Ariccia G, et al. EEG Responses to Visual Landmarks in Flying Pigeons. *Curr Biol* 2009, 19:1159–1166.

## P351 A coarse-graining framework for spiking neuronal networks: from local, low-order moments to large-scale spatiotemporal activities

### Yuxiu Shao^1^, Louis Tao^2^, Jiwei Zhang^3^

#### ^1^Peking University, School of Life Sciences, Beijing City, China; ^2^Peking University, Center for Bioinformatics, National Laboratory of Protein Engineering and Plant Genetic Engineering, Beijing, China; ^3^Wuhan University, School of Mathematics and Statistics, Beijing City, China

##### **Correspondence:** Yuxiu Shao (shaoyx@mail.cbi.pku.edu.cn)

*BMC Neuroscience* 2019, **20(Suppl 1)**: P351

Emergent nonlinear dynamics within the primary visual cortex (V1) may determine how information is encoded and processed in the early visual pathway and have been shown to affect visual perception. A major goal of visual systems neuroscience is to understand how complex visual functions can arise from the collective nonlinear dynamics of the V1 network. This challenge has been partly met through electrophysiological recordings, optical imaging and neural population models. But a full account of how the multi-scale population-dynamics emerges from the detailed biophysical properties of individual neurons and the network architecture remains elusive. Previously, working on a homogeneously coupled network, using a partitioned ensemble average (PEA), we derived a series of population dynamics models, ranging from Master equations, to Fokker-Planck equations, and culminating in an augmented system of spatially-coupled ODEs [1]. This reduced model can describe the many dynamical regimes of a strongly coupled neuronal network, ranging from homogeneity to synchronous avalanche-like activity patterns.

Here we present an application of our reduction to a realistic integrate-and-fire network model of V1 [2]. The spatially-coupled ODE system, with locally organized visual feature maps and long-range orientation specific couplings, recapitulates the cortical wave generation and propagation induced by visual illusory stimuli [3]. We also found that the temporal dynamics of individual patches can be well captured by a low-dimensional set of voltage moments, demonstrating the effectiveness of our reduction (Fig. 1 A). Furthermore, this coarse-graining reveals the importance of the temporal differences between on-/off-pathways (Fig. 1 B), that may account for the directional motion perception from dark to bright [4].

**Fig. 1:**
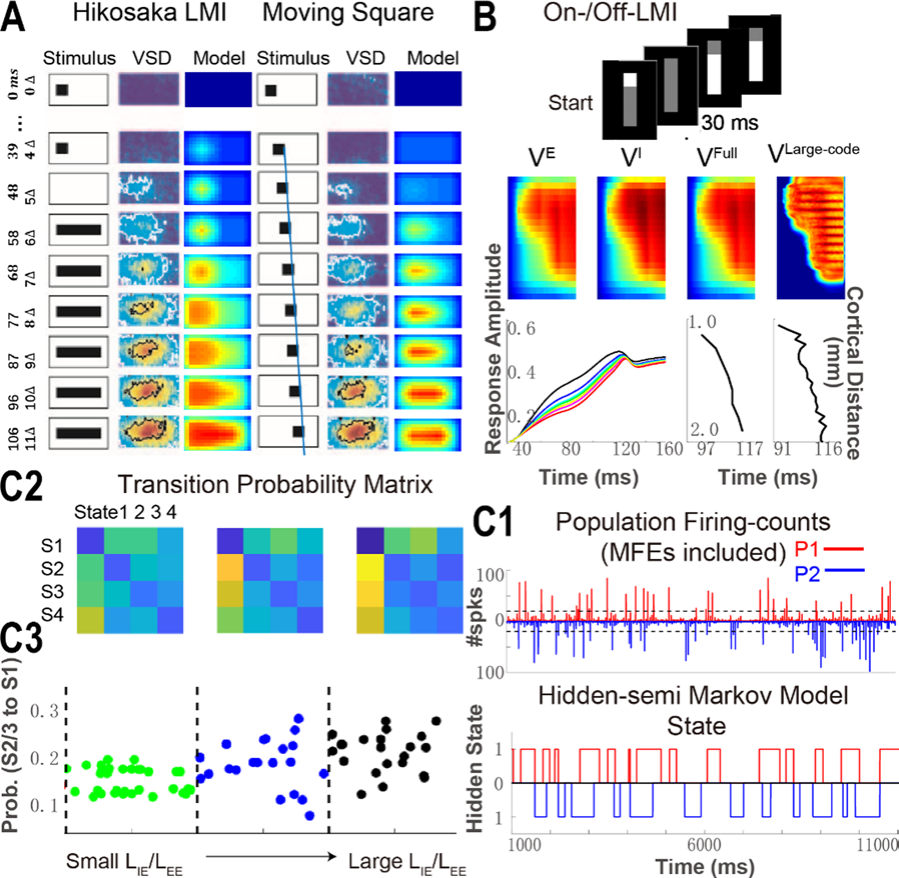
**a** Demonstrates the effectiveness of our reduced model from various visual illusory stimuli, In **b**, our model reveals the importance of temporal properties between on-/off-pathway, **c**1,2,3 presents the transient dynamic MFEs, and the interplay between network architecture and the emergent cortical dynamics with these MFEs

We further extend our framework to model other heterogeneous dynamics, e.g., [5]. Our coarse-grained dynamical models successfully capture a particular type of transient dynamics, multiple-firing events (MFEs) (Fig. 1 C1). This type of neural activity pattern emerges naturally in fluctuation-driven networks of strongly coupled neurons and contributes to the overall heterogeneous dynamics. The mechanisms underlying these MFEs, their generation, evolution, and contribution to large-scale cortical activity patterns (Fig. 1 C2,3), cannot easily be understood. Using our PEA formalism, we can address and account for this type of transition, leading to a more compact and efficient coarse-grained cortical model. We present simulations and analyses illustrating the utility of our framework, making explicit the connection between the emergent macroscopic dynamics to the underlying network architecture.

**Acknowledgments:** We thank the Natural Science Foundation of China: grants 31771147 (YS, LT), 91232715 (LT), 11771035 (JZ), 91430216 (JZ), and U1530401 (JZ), and the Open Research Fund of the State Key Laboratory of Cognitive Neuroscience and Learning grant CNLZD1404 (YS, LT).

**References**

1. Zhang J, Shao Y, Rangan AV, Tao L. A coarse-graining framework for spiking neuronal networks: from strongly-coupled conductance-based integrate-and-fire neurons to augmented systems of ODEs. *Journal of computational neuroscience* 2019 Feb 16:1–22.
2. Rangan AV, Cai D, McLaughlin DW. Modeling the spatiotemporal cortical activity associated with the line-motion illusion in primary visual cortex. *Proceedings of the National Academy of Sciences* 2005 Dec 27;102(52):18793–800.
3. Jancke D, Chavane F, Naaman S, Grinvald A. Imaging cortical correlates of illusion in early visual cortex. *Nature* 2004 Mar;428(6981):423.
4. Rekauzke S, Nortmann N, Staadt R, Hock HS, Schöner G, Jancke D. Temporal asymmetry in dark–bright processing initiates propagating activity across primary visual cortex. *Journal of Neuroscience* 2016 Feb 10;36(6):1902–13.
5. Cai D, Rangan AV, McLaughlin DW. Architectural and synaptic mechanisms underlying coherent spontaneous activity in V1. *Proceedings of the National Academy of Sciences* 2005 Apr 19;102(16):5868–73.

## P352 Reconstruction of sparse neuronal network connectivity in the balanced operating regime

### Victor Barranca^1^, Douglas Zhou^2^

#### ^1^Swarthmore College, Mathematics and Statistics, Swarthmore, PA, United States of America; ^2^Shanghai Jiao Tong University, Institute of Natural Sciences, Shanghai, China

##### **Correspondence:** Victor Barranca (vbarran1@swarthmore.edu)

*BMC Neuroscience* 2019, **20(Suppl 1)**: P352

Determining the structure of a network is of central importance to understanding its function in both neuroscience and applied mathematics. However, recovering the structural connectivity of neuronal networks remains a fundamental challenge both theoretically and experimentally. While neuronal networks function in certain dynamical regimes, which may influence their connectivity reconstruction, there is widespread experimental evidence of a balanced neuronal operating state in which strong excitatory and inhibitory inputs are dynamically adjusted such that neuronal voltages primarily remain near resting potential. Utilizing the firing dynamics of neurons in such a balanced regime in conjunction with the ubiquitous sparse connectivity structure of neuronal networks, we develop a theoretical framework using compressive sensing theory for efficiently reconstructing network connections by measuring individual neuronal dynamics in response to a relatively small ensemble of random stimuli injected over a short time scale. By tuning the network dynamical regime, we determine that the highest fidelity of reconstructions is achievable in the balanced state. We hypothesize the balanced dynamics observed in vivo may therefore be a result of evolutionary selection for optimal information encoding and expect the methodology developed to be tractable for alternative model networks as well as experimental paradigms.

## P353 Divisive normalization circuits faithfully represent visual, olfactory and auditory stimuli

### Aurel A. Lazar, Tingkai Liu, Yiyin Zhou

#### Columbia University, Department of Electrical Engineering, New York, NY, United States of America

##### **Correspondence:** Tingkai Liu (tl2747@columbia.edu)

*BMC Neuroscience* 2019, **20(Suppl 1)**: P353

Divisive normalization has long been proposed as a canonical neural computation employed by the brain, particularly for the purpose of adaptation and attention modulation [1]. In photoreceptors and olfactory sensory neurons, for example, divisive normalization enables adaptation to a wide range of input intensity and contrast values. Adaptation to the mean and variance of the amplitude of the stimuli has also been observed in the auditory system.

It is often considered that processing by neural circuits leads to loss of information. However, it has been rigorously demonstrated that 1) sensory neural circuits with linear receptive fields and biophysical spike generators can faithfully represent encoded sensory stimuli [3], and 2) visual stimuli encoded by an ensemble of complex cells, classically considered to have (quadratically) nonlinear receptive fields, can be perfectly recovered [4].

Divisive normalization is highly nonlinear by nature. While the presence of it is beneficial for downstream processing, the question remains as to whether this highly nonlinear transformation could also faithfully represent the entire information content of the sensory input and convey them to downstream neurons.

In the present work, we leverage the functional framework of divisive normalization proposed in [4], and explore the problem of input recovery given output samples. We formulate the stimulus encoding with divisive normalization circuits as generalized sampling. The reconstruction of stimuli from irregularly sampled outputs of divisive normalization circuits can be solved via an optimization problem with linear constraints. We provide novel algorithms based on semidefinite programming and alternating direction method of multipliers [5] for faithful recovery of sensory inputs, and demonstrate that (Fig. 1), under conditions akin to the Shannon-Nyquist Rate, divisive normalization is indeed capable of faithfully representing the input stimuli for downstream processing. We provide theoretical and simulation results regarding the performance of the algorithm in terms of sample efficiency. Finally, we demonstrate the ability to faithfully recovery the input of a model auditory circuit.

**Fig. 1:**
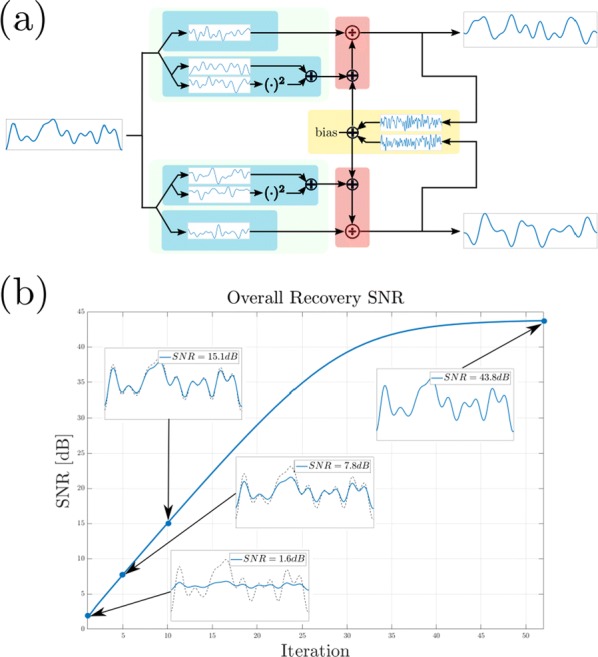
Schematic of Divisive Normalization Circuit (DNC). **a** Components of the circuit. **b** Convergence of the recovery algorithm for a different number of iterations

**Acknowledgments:** The research reported here was supported by AFOSR under grant #FA9550-16-1-0410.

**References**

1. Carandini M, Heeger DJ. Normalization as a canonical neural computation. *Nature Reviews Neuroscience* 2012 Jan;13(1):51.
2. Lazar AA, Zhou Y. Reconstructing natural visual scenes from spike times. *Proceedings of the IEEE* 2014 Oct;102(10):1500–19.
3. Lazar AA, Ukani NH, Zhou Y. Sparse Functional Identification of Complex Cells from Spike Times and the Decoding of Visual Stimuli. *The Journal of Mathematical Neuroscience* 2018 Dec;8(1):2.
4. Lazar AA, Ukani NH, Zhou Y. Modeling Contrast Gain Control of Fly Photoreceptors. Computational Neuroscience Meeting, CNS*2018, 2018 Seattle, WA.
5. Wen Z, Goldfarb D, Yin W. Alternating direction augmented Lagrangian methods for semidefinite programming. *Mathematical Programming Computation* 2010 Dec 1;2(3–4):203–30.

## P354 Movement directional maps for optical imaging with fNIRS (functional near-infrared spectroscopy)

### Nicoladie Tam^1^, Luca Pollonini^2^, George Zouridakis^2^

#### ^1^University of North Texas, Department of Biological Sciences, Denton, TX, United States of America; ^2^University of Houston, Department of Engineering Technology, Houston, TX, United States of America

##### **Correspondence:** Nicoladie Tam (nicoladie.tam@unt.edu)

*BMC Neuroscience* 2019, **20(Suppl 1)**: P354

Using invasive single-unit spike recording techniques, it has been shown that intentional movement direction can be decoded computationally by the population vector of an ensemble of cortical motor neurons [1]. In this study, we aim to estimate movement direction using noninvasive fNIRS (functional near-infrared spectroscopy) optical imaging. Since fNIRS records the hemodynamic activity of an ensemble of neurons, it correlates well with the scalar sum of neural firings rather than the vector sum of individual neural firings. Therefore, the population vector cannot be computed from individual neurons, since individual neural firings are not captured by the hemodynamic responses.

We propose to use the combined locations in the cortex to reconstruct a map of the intentional movement direction. More specifically, considering that fNIRS can record different parts of the cortex simultaneously, we can obtain the differential activation of distinct locations in the cortex to reconstruct the movement direction. We analyzed recordings obtained from human subjects who were instructed to make arm movements in different orthogonal directions while hemodynamic responses were recorded using fNIRS from the premotor and motor cortex simultaneously. We then correlated the movement direction with the differential hemodynamic activation at different locations in the motor cortex to determine the intentional movement direction.

Our computational method revealed that the differential activation of distinct ensembles of neurons could be accurately correlated with different movement directions. These results augment our previous findings [2–6] that intentional movement direction can be accurately reconstructed from different computational methods using the hemodynamic responses and distinct activation patterns of neural ensembles in the motor cortex.

**References**

1. Georgopoulos AP, Schwartz AB, Kettner RE. Neuronal population coding of movement direction. *Science (New York, NY)* 1986, 233(4771):1416–1419.
2. Tam ND, Zouridakis G. Decoding movement direction from motor cortex recordings using near-infrared spectroscopy. In: *Infrared Spectroscopy: Theory, Developments and Applications* 2014. edn. Hauppauge, NY: Nova Science Publishers, Inc.;
3. Tam ND, Pollonini L, Zouridakis G. Decoding movement direction using phase-space analysis of hemodynamic responses to arm movements based on functional near-infrared spectroscopy. In: *Proceedings of IEEE Engineering in Medicine & Biology Society* 2016: 1580–1583.
4. Tam N, Zouridakis G, Pollonini L. Population vector decoding for optical imaging with fNIRS (functional near-infrared spectroscopy). *BMC Neuroscience* 2018, 19(Suppl 2):P133.
5. Tam ND, Zouridakis G. Temporal decoupling of oxy- and deoxy-hemoglobin hemodynamic responses detected by functional near-infrared spectroscopy (fNIRS). *Journal of Biomedical Engineering and Medical Imaging* 2014, 1(2):18–28.
6. Tam ND, Zouridakis G, Pollonini L. Phase space analysis of hemodynamic responses to intentional movement directions using functional near-infrared spectroscopy (fNIRS) optical imaging technique. *BMC Neuroscience* 2016, 17(Suppl 1):P3.

## P355 Applying the method of spectral decomposition to analyse the response of integrate-and-fire neurons to time varying input

### Lukas Deutz^1^, Hugh Osborne^2^, Marc de Kamps^3^

#### ^1^University of Leeds, School of Computing, Leeds, Germany; ^2^University of Leeds, Institute for Artificial and Biological Computation, School of Computing, United Kingdom; ^3^University of Leeds, School of Computing, Leeds, United Kingdom

##### **Correspondence:** Lukas Deutz (scld@leeds.ac.uk)

*BMC Neuroscience* 2019, **20(Suppl 1)**: P355

Understanding the behaviour of large recurrent networks of spiking neurons is one of the major challenges in computational neuroscience. A first step in understanding the dynamical properties of such networks is to determine the location and stability of equilibria and how they depend on the connectivity profile and single neuron properties. Integrate-and-fire neuron models are widely used for this purpose because they are simple enough to be studied analytically while still being able to capture important dynamical features observed on a single neuron level, or in a network context. In the diffusion approximation, the synaptic input is approximated by Gaussian white noise which gives rise to a Fokker–Planck equation describing how the neuron responds on average to given input statistics.

Even for simple models, solving the Fokker–Planck equation for time dependent input statistics is a difficult task. If the amplitude of the temporal modulation of the input rate is small compared to its baseline, then perturbation theory can be used. In first order approximation, the neuron acts as a linear filter called the transfer function. It determines the gain and the phase shift of the neuron response for a given frequency of the input modulation. Using spectral decomposition [1, 2], we propose a method to systematically extend the solution to higher orders.

To do so, the eigenfunctions and associated eigenvalues of the Fokker–Planck operator have to be known for a model of interest. So far, analytic solutions only exist for the perfect and leaky integrate-and-fire neuron (PIF, LIF). To overcome this problem, we developed a numerical method to determine a finite number of eigenfunctions and eigenvalues for a given neuron model and confirmed that it is able to reconstruct the theoretical results for the PIF and LIF neuron (Fig. 1). Using the simulation platform MIIND, we demonstrate that the neuron dynamics for the PIF and LIF neuron can be reliably explained in terms of eigenmodes even if the Gaussian input is replaced by a Poisson process if the synaptic efficacy is small. We started to investigate if the method of spectral decomposition can be extended to regimes with large synaptic efficacy by correcting eigenvalues and eigenfunctions in a systematic manner.

**Fig. 1:**
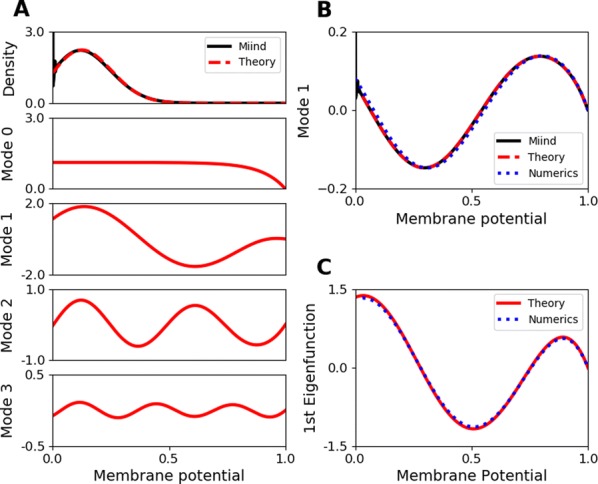
**a** Shows how the density function of the membrane potential for the PIF neuron can be decomposed into individual modes. **b** Compares the state of the first mode at a given point in time obtained by our numerical method (blue) to the theoretical result (red) and MIIND (black). **c** Compares the numerical reconstruction of the first eigenfunction (blue) to the theoretical result (red)

As a next step, we want to determine the spectrum for more complicated models including spike generating currents, adaptation and synaptic dynamics. Since theoretical results are lacking for these models, we use numerical results generated with MIIND for validation.

Once the eigenfunctions are known, the neuronal dynamics are solely captured by the time evolution of the weighting coefficients of individual modes, leading to an infinite linear system of coupled differential equations. Our hope is that a small number of modes is sufficient to describe the overall network dynamics. If so, then spectral decomposition could facilitate a very efficient way to simulate large scale networks on a population level.

**References**

1. Mattia M, Del Giudice P. Population dynamics of interacting spiking neurons. *Physical Review E.* 2002, Nov 26;66(5):051917.
2. Knight BW. Dynamics of encoding in neuron populations: some general mathematical features. Neural *Computation* 2000 Mar 1;12(3):473–518.

## P356 Temporal structures show different atrophy dynamics throughout the progression of cognitive impairment

### Meritxell Valentí, Jaime David Gómez-Ramírez, Miguel Ángel Fernández-Blázquez, Marina Ávila-Villanueva, Belén Frades-Payo, Teodoro del Ser-Quijano

#### CIEN Foundation, Madrid, Spain

##### **Correspondence:** Meritxell Valentí (merivalenti@gmail.com)

*BMC Neuroscience* 2019, **20(Suppl 1)**: P356

**Background:** The atrophy in the hippocampus is greater in subjects with AD than in subjects with mild cognitive impairment and greater than the atrophy thought to be produced during ageing. This atrophic pattern is furthermore asymmetric, being more pronounced in the left side [1]. A mechanistic understanding of the progression of atrophy is still lacking. Volumetric studies in structures in the temporal lobes other than the hippocampus show complex dynamics, including an increase in volume at the beginning of cognitive impairment [2].

**Objective:** To study whether the hippocampal atrophy observed in mild cognitive impairment of the amnesic type shows a linear decrease over time. We compare the pattern of atrophy in patients with Alzheimer’s disease, frontotemporal dementia and healthy older.

**Methods:** The Vallecas Project carries out the annual monitoring of healthy and cognitively impaired people through neurological evaluation, neuropsychological, blood analysis and neuroimaging for the early detection of cognitive impairment. We analyzed the hippocampal atrophy observed in an MRI 3 T scanner over a 7 years longitudinal study using Freesurfer 6.0 for automatic subcortical segmentation [3].

**Results:** A greater decrease in hippocampal volume was observed in people with Alzheimer’s disease and frontotemporal dementia compared to mild cognitive impairment. Fluctuation was observed in the volume of the hippocampus of the subject with mild cognitive impairment, increasing just before diagnosis and then descending, which is not seen in the rest of cases.

**Conclusions:** Temporal structures show different atrophy dynamics throughout the progression of cognitive impairment, and automatic subcortical segmentation is relevant to find possible fluctuations at its onset.

**References**

1. Shi F, Liu B, Zhou Y, Yu C, Jiang T. Hippocampal volume and asymmetry in mild cognitive impairment and Alzheimer’s disease: Meta-analyses of MRI studies. *Hippocampus* 2009;19[11]:1055–64.
2. Gispert JD, Rami L, Sánchez-Benavides G, et al. Nonlinear cerebral atrophy patterns across the Alzheimer’s disease continuum: impact of APOE4 genotype. *Neurobiol Aging* 2015 Oct 1;36[10]:2687–701.
3. Fischl B, Salat DH, Busa E, et al. Whole brain segmentation: automated labeling of neuroanatomical structures in the human brain. *Neuron* 2002 Jan 31;33[3]:341–55.

## P357 RNNs develop history biases in an expectation-guided two-alternative forced choice task

### Manuel Molano-Mazon, Ainhoa Hermoso-Mendizabal, Jaime de la Rocha

#### IDIBAPS, Theoretical Neurobiology, Barcelona, Spain

##### **Correspondence:** Manuel Molano-Mazon (molano@clinic.cat)

*BMC Neuroscience* 2019, **20(Suppl 1)**:P357

The role of prior expectations biasing perceptual decisions has been extensively studied in the context of two-alternative forced-choice tasks (2AFC). Here, we investigate this issue by training recurrent neural networks (RNNs) in a novel 2AFC task where both the current sensory evidence and the previous trial history provide information about the identity of the correct choice. Briefly, the probabilitypof repeating the previous stimulus category was varied between a low (e.g.p = 0.2, alternating block) and a high (e.g.p = 0.8, repeating block), in blocks of 200 trials [1].

We found that RNNs are able to learn the task and develophistory transition biases (a tendency to repeat/alternate the previous choice more often depending on the number of previous repetitions vs. alternations) similar to those found in rats performing the same task (Fig. 1a, left) [1]. Consistent with intuition, networks perform better with respect to an ideal observer as the correlation in the trial sequence is made stronger (Fig. 1b, left). Transition biases after correct trials are positively correlated with the probability of transitioning (orange dots), and are larger in magnitude than the biases after error trials (blue dots) (Fig. 1b, middle). In addition, sensitivity (slope of the curve) is negatively correlated with the transition probabilities (Fig. 1b, right).

**Fig. 1:**
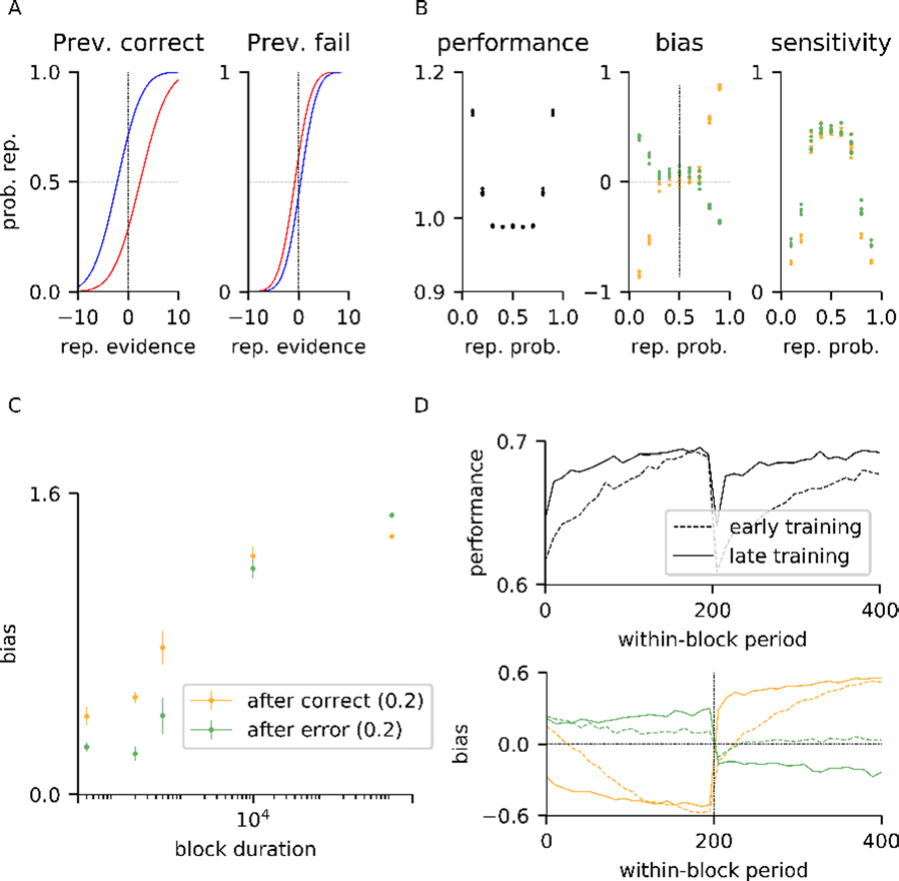
**a** RNNs develop transition biases similar to those found in rats, including the difference between the biases after correct and error trials. **b** Performance (left), bias (middle) and slope (right) for different transition probabilities. **c** The magnitude of the bias increases with the block sizes. **d** Evolution of performance and bias through training

Also in line with experimental results, the magnitude of the bias is stronger after correct trials than after errors (Fig. 1a, right). This could indicate that RNNs (and rats) develop different strategies for the after-correct and after-error scenarios. Indeed, while a rewarded response likely strengthens the confidence of the agent on the current strategy, a failed trial could indicate both an error caused by noise in the perception of the stimulus or by a change in the transition probability, revealing a flaw in the strategy. Consistently with this view, as the duration of the alternating/repeating blocks is increased and thus the change in the transition probability is made less probable, the asymmetry between the after-correct and after-error biases vanishes (Fig. 1c). Therefore, the different behaviors in the after-correct and after-error scenarios could reflect the fact that RNNs find a trade-off between the optimal strategy for each block and the need to rapidly adapt to a change of blocks. Indeed, throughout training networks learn to rapidly adapt to changes in transition probability (i.e. a block change): the bias after those changes adjusted faster during the late stages of the training than at earlier stages (Fig. 1d).

We also found that 75% of the network units presented activity that correlated with the number of repetitions during the last 5 trials, which is consistent with the estimation of the transition probabilities done by the networks during the task.

The present work demonstrates that RNNs can learn to perform a task which promotes the development of history transition biases and to rapidly adapt to new environments presenting different statistics.

**Acknowledgments:** Generalitat de Catalunya (grant 2017-BP-00305) and the European Research Council (ERC-2015-CoG – 683209_PRIORS).

**Reference**

1. Hermoso-Mendizabal A, Hyafil A, Rueda-Orozco PE, Jaramillo S, Robbe D, de la Rocha J. Response outcomes gate the impact of expectations on perceptual decisions. *bioRxiv* 2019 Jan 1:433409.

## P358 How cells in primary visual cortex combine filters for feature selectively and invariance

### Ali Almasi^1^, Shaun Cloherty^2^, Yan Wong^3^, Michael Ibbotson^1^, Hamish Meffin^1^

#### ^1^National Vision Research Institute, Melbourne, Melbourne, Australia; ^2^Monash University, Department of Physiology, Clayton, Australia; ^3^The University of Melbourne, Department of Biomedical Engineering, Parkville, Australia

##### **Correspondence:** Ali Almasi (aalmasi@student.unimelb.edu.au)

*BMC Neuroscience* 2019, **20(Suppl 1)**: P358

Object recognition in scenes develops across a hierarchy of visual areas. Robust recognition requires fine selectivity for particular features of relevance and invariance to irrelevant features. Deep convolutional neural networks have achieved near-human levels of performance in object recognition by iteratively applying filters that select features, followed by pooling of their outputs to generate invariance.

We applied a “filter-then-pool” model to recordings from neurons in cat primary visual cortex (V1) to investigate visual feature selectivity and invariance in the brain. Many neurons pooled the outputs of multiple filters, resulting in selectivity for feature characteristics that were preserved across filters, and invariance to feature characteristics that differed across filters. We found cells corresponding to the “energy model” of V1 complex cells that were invariant to spatial phase but selective to a combination of other feature characteristics. We also frequently found cells that showed only partial invariance to spatial phase, while exhibiting invariance to perturbations in peak orientation and spatial frequency. For each of these feature characteristics, some cells were more selective for the characteristic and others that were more invariant. To quantify the “selective-to-invariant” spectrum we used a bandwidth measuring the range of a characteristic over which a cell responded equally allowing for modest changes in contrast (< 2×). Peak orientation had the greatest portion of selective cells, followed by peak spatial frequency and then spatial phase, the latter showing the greatest portion of invariance. The bandwidth measure also allowed us to quantify how much the nonlinear pooling operation contributed to invariance by comparing it to the bandwidth expected from a linear operation. This showed that spatial phase invariance benefited the most from nonlinear pooling over multiple features, with the bandwidth frequently much greater than expected in the linear case, and sometimes reaching the maximum possible (360 deg). In contrast, orientation and spatial frequency had bandwidths that did not increase much with nonlinear pooling over that of linear pooling.

Thus, in V1 there is a diversity of cells that combine selectivity for some feature characteristics with invariance to perturbations in others. This diversity encompasses a variety of feature characteristics beyond spatial phase.

